# Reactivity of (*Z*)-4-Aryliden-5(4*H*)-thiazolones: [2 + 2]-Photocycloaddition, Ring-Opening Reactions, and Influence of the Lewis Acid BF_3_

**DOI:** 10.1021/acs.joc.1c01458

**Published:** 2021-08-16

**Authors:** Sonia Sierra, David Dalmau, Sheila Higuera, Darío Cortés, Olga Crespo, Ana I. Jimenez, Alexandra Pop, Cristian Silvestru, Esteban P. Urriolabeitia

**Affiliations:** † Instituto de Síntesis Química y Catálisis Homogénea, ISQCH (CSIC-Universidad de Zaragoza), Pedro Cerbuna 12, 50009 Zaragoza, Spain; ‡ Supramolecular Organic and Organometallic Chemistry Centre, Department of Chemistry, Faculty of Chemistry and Chemical Engineering, Babeş-Bolyai University, Str. Arany Janos 11, 400028 Cluj−Napoca, Romania

## Abstract

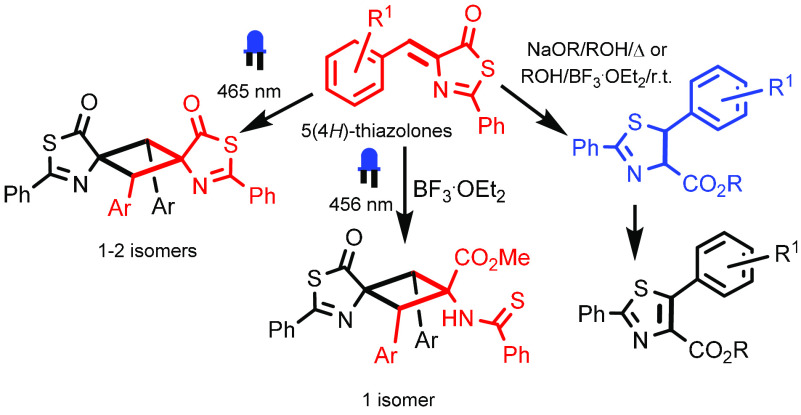

The irradiation of (*Z*)-2-phenyl-4-aryliden-5(4*H*)-thiazolones **2** with blue light (465 nm) in CH_2_Cl_2_ solution promotes [2 + 2]-photocycloaddition of the exocyclic C=C bonds and the formation of the dispirocyclobutanes **3**. This reaction takes place with high stereoselectivity, given that the ε-isomer (1,3 head-to-tail syn coupling) is formed in more than 90% yield in most of the cases. However, irradiation of 5(4*H*)-thiazolones **2** with blue light (456 nm) in dry MeOH in the presence of BF_3_·OEt_2_ leads to the monospirocyclobutanes **4** with full stereoselectivity, also affording the ε-isomer. A ring-opening reaction of only one of the thiazolone rings appears to have taken place in **4** upon methanolysis, leading to the corresponding ester and thioamide groups. The treatment of free 4-aryliden-5(4*H*)-thiazolones **2** with a base in alcohol (NaOR/ROH) also produces a ring-opening reaction of the heterocycle by methanolysis, although, under these reaction conditions, further intramolecular S-attack at the exocyclic C(H)=C bond and cyclization is observed, forming the dihydrothiazoles **5** or **6** as mixtures of *cis* (*RS*/*SR*)- and *trans* (*RR*/*SS*)-isomers with high diastereomeric excess. *trans*-(*RR/SS*)-Dihydrothiazoles **6** can be isolated as pure diastereoisomers by column chromatography. Surprisingly, dihydrothiazoles **5** can also be obtained by the treatment of 4-aryliden-5(4*H*)-thiazolones **2** with BF_3_·OEt_2_ in methanol in the absence of a base.

## Introduction

**Figure 1:**
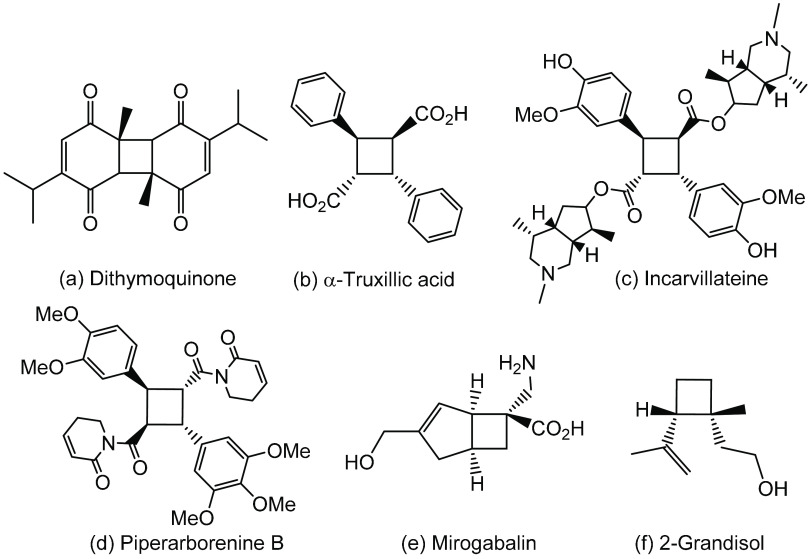
Cyclobutanes with important pharmacological activity.

The [2 + 2]-photocycloaddition reaction is a powerful synthetic tool for the tailored and versatile preparation of cyclobutanes by the C—C coupling of olefinic C=C bonds.^1^ The relevance of the cyclobutane ring resides in its presence as a common structural motif in natural products or synthetic compounds with strong pharmacological activity. Some examples of relevant cyclobutanes can be found in Figure 1.^2−7^ Moreover, cyclobutanes are also interesting synthetic intermediates as they show a particular reactivity due to the high ring strain.^8−11^

Figure 1 also shows that these cyclobutanes contain many chiral centers. The development of methods for control of the stereoselectivity during cyclobutane synthesis has attracted substantial attention.^12−15^ However, for photochemical processes, a high stereoselectivity is only achieved when the reactions take place in the solid state and topochemical Schmidt’s conditions are achieved. This is the case, for instance, for the synthesis of α-truxillic (Figure 1b) and β-truxinic acid derivatives.^16−18^ In general, the [2 + 2]-photocycloadditions performed in solution suffer a lack of stereoselectivity, and the use of auxiliary reagents such as chiral templates, sensitizers or catalysts, is mandatory.^19−31^

**Figure 2:**
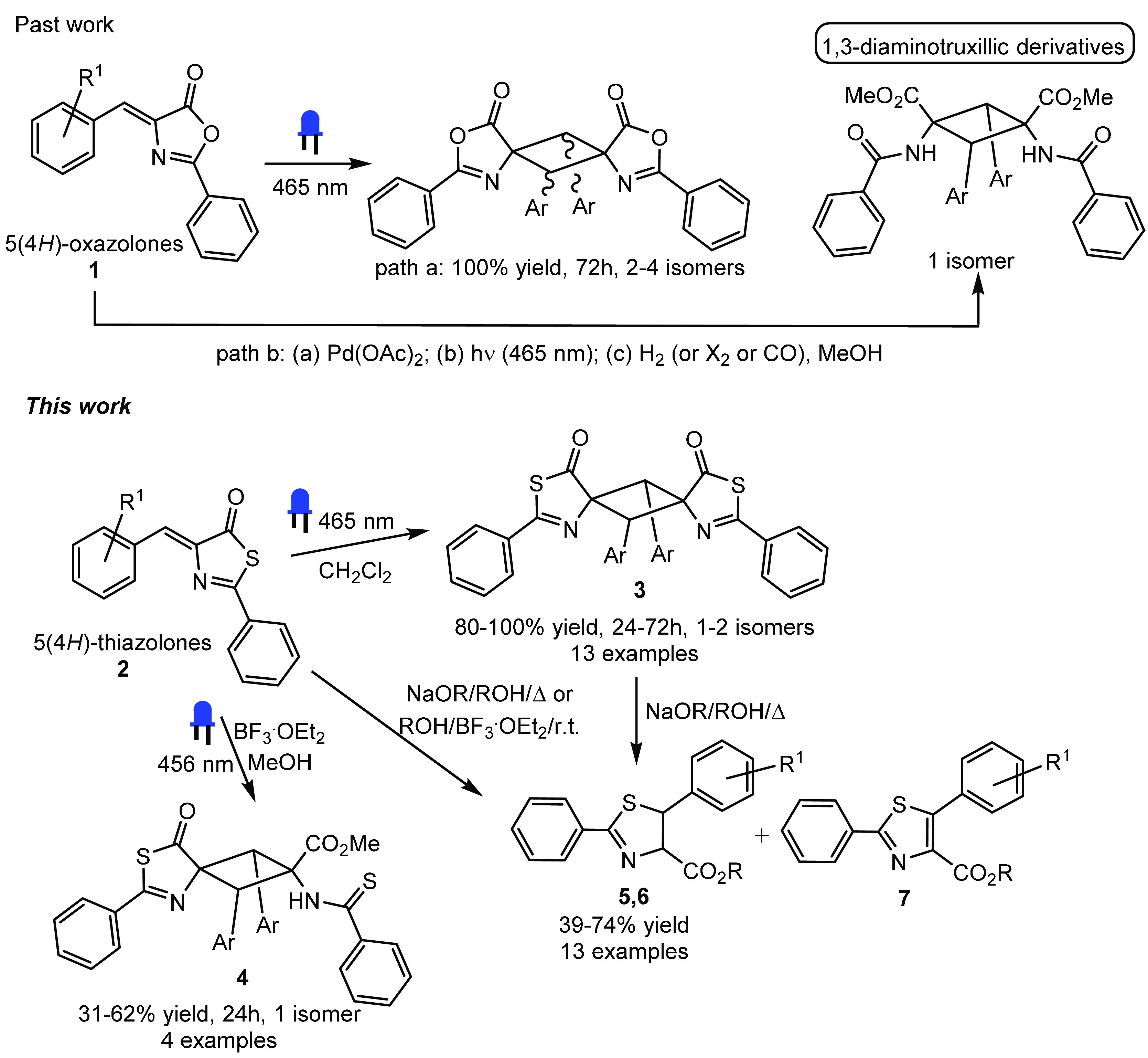
Context of this work, comparison with previous work and main achievements.

We are interested in a particular family of cyclobutanes, namely, 1,3-diaminotruxillic derivatives (Figure 2), which are well-known because of their antinociceptive activity. A renewed interest in these compounds has arisen over the past few years because truxillic derivatives have been shown to be FABP (fatty acid binding protein) inhibitors and are responsible for the cellular reuptake of anandamide (an endocannabinoid neurotransmitter).^32−36^ As a result, they are promising candidates in efficient treatments for chronic pain.^37^ However, this is not the only outstanding pharmacological activity of truxillic derivatives as they have also been recently shown to be the only nonpeptidic GLP-1R (glucagon-like peptide receptor) agonists for the treatment of type 2 diabetes mellitus.^38−40^

As a result of this interest, we have developed different methodologies for the stereoselective synthesis of 1,3-diaminotruxillic derivatives (Figure 2). Among these, the direct irradiation of (*Z*)-4-aryliden-5(4*H*)-oxazolones **1** shows high simplicity and versatility, together with some degree of stereoselectivity (Figure 2, past work, path a).^41^ Thus, we have shown that the [2 + 2]-photocycloaddition of oxazolones **1** can occur using low-power (less than 20W), blue light (465 nm) irradiation sources. This method works for oxazolones bearing electron-donating and electron-withdrawing substituents and gives quantitative yields of cyclobutanes in almost all cases studied. However, it requires long reaction times (up to 3 days) and affords up to four different stereoisomers, although one of them (ε) is obtained in 50–90% abundance with respect to the other isomers. This method has been complemented with another, three-step strategy in which a palladium complex behaves as a template, thus allowing the isolation of 1,3-diaminotruxillic derivatives with good yields as single isomers (Figure 2, past work, path b).^42−45^

The (*Z*)-4-arylidene-5(4*H*)-thiazolones **2** are the sulfur counterparts of 4-arylidene-5(4*H*)-oxazolones **1**. Despite the structural analogy, unsaturated 5(4*H*)-thiazolones are less well-known, and their synthetic potential is underdeveloped.^46,47^ Sulfur-containing drugs exhibit remarkable pharmacological activity, and as such, they are targets of particular interest from the point of view of pharmaceutical companies.^48^ Proof of this interest is the fact that there are at least 249 sulfur-containing drugs approved by the US Food and Drug Administration (FDA).^49^ Thiazolones have received some attention as sulfur-containing drugs, initially during the study of penicillin (it was believed that the active substance contained a thiazolone ring rather than a thiazolidine),^50,51^ and more recently as promising anticancer compounds.^52^ Due to the close relationship between 5(4*H*)-oxazolones **1** and 5(4*H*)-thiazolones **2**, the interesting reactivity of oxazolones to give 1,3-diaminotruxillic derivatives observed in our previous studies,^41−45^ the interest in sulfur-containing compounds due to their interesting pharmacological properties, and the complete absence of previous studies in this area, we have studied the reactivity of (*Z*)-4-arylidene-5(4*H*)-thiazolones **2** in [2 + 2]-photocycloaddition reactions and in ring-opening reactions upon alcoholysis (Figure 2, this work). With the aim of further exploring the chemical possibilities of these substrates, and taking into account the known influence of Lewis acids on photochemical reactions^26,28,53−62^ (acceleration and/or change in the orientation and selectivity of the reactions), we have examined both processes (ring opening and photocycloaddition) in the presence of a simple Lewis acid, namely BF_3_, and present the results obtained below.

## Results and Discussion

### Synthesis of (*Z*)-4-Arylidene-5(4*H*)-thiazolones **2** and [2 + 2]-Photocycloaddition by Direct Irradiation

**Figure 3:**
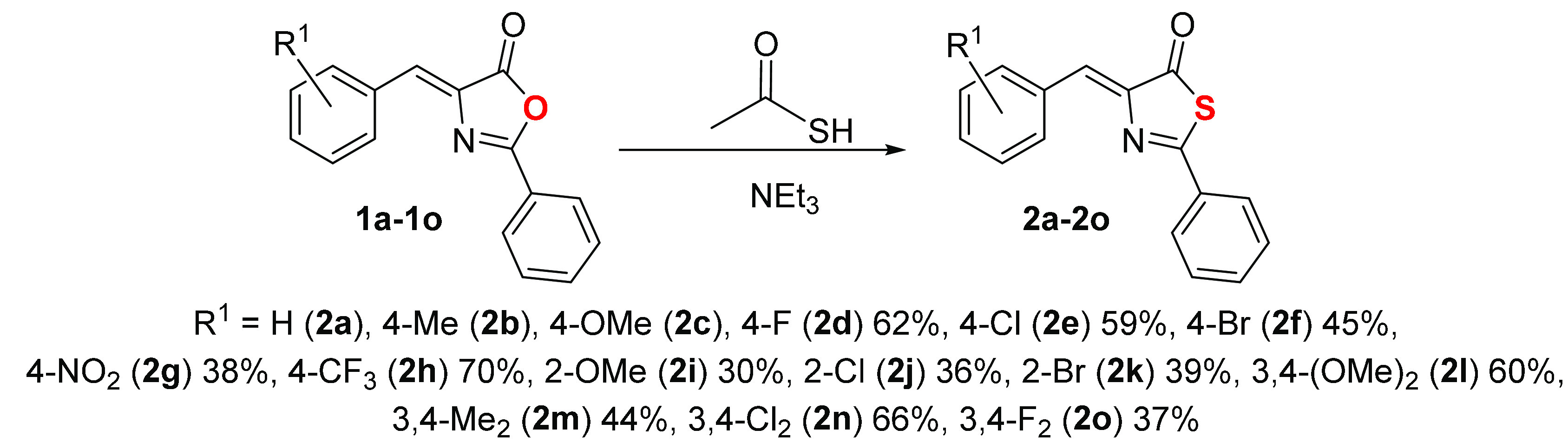
Thiazolones **2a–2o** used in this work and synthetic method.

The thiazolones **2a–2o** used in this work are shown in Figure 3. Synthesis was carried out following the same experimental procedure reported by Rao and Filler,^46^ which in turn were based on the original work of Behringer et al.^63,64^ Following this method, the treatment of oxazolones **1a–1o** with thioacetic acid in the presence of substoichiometric amounts of NEt_3_ gave the corresponding thiazolones **2a–2o** as air- and moisture-stable solids. Thiazolones **2a–2o** contain electron-withdrawing or electron-donating substituents at different positions of the 4-arylidene ring in order to cover the widest scope. Thiazolones **2a–2c** have been described previously, although **2b** was prepared using a different method,^65,66^ and although thiazolones **2e**, **2f**, **2g**, **2h**, and **2j** appear in Scifinder, there are either no references associated with their synthesis or no details can be found in the corresponding literature. As such, they are fully characterized here (see Supporting Information).

The HRMS (ESI^+^) spectra of **2a–2o** show peaks in agreement with the stoichiometries proposed in Figure 3. In addition, the ^1^H NMR spectra of **2a–2o** show a pattern of peaks quite similar to that of the oxazolone precursors **1a–1o**, with only the signal due to the *ortho*-H of the 2-Ph ring in **2a–2o** showing a downfield shift with respect to the same signal in **1a–1o**. The ^13^C NMR spectra of **2a–2o**, in which the signal due to the S—**C** (=O) carbon appears around 195 ppm, downfield shifted by more than 20 ppm with respect to the O—**C** (=O) carbonyl carbon peak (around 170 ppm), are much more informative.

**Figure 4:**
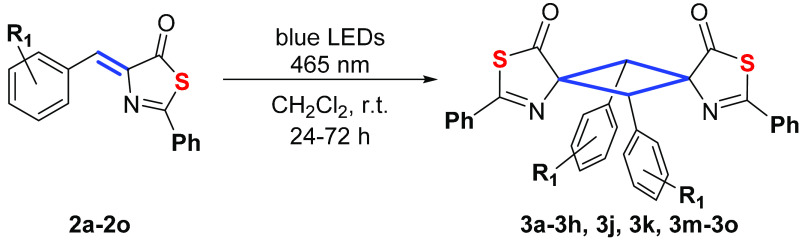
[2 + 2]-Photocycloaddition of 4-arylidene-5(4*H*)-thiazolones **2** to give cyclobutanes **3**.

Solutions of thiazolones **2a–2o** in CH_2_Cl_2_ were then irradiated with blue light (465 nm) at room temperature using the irradiation setup described in the Experimental Section (PCB with 24 blue LEDs). This irradiation promoted the [2 + 2]-photocycloaddition of the exocyclic C=C bond of thiazolones **2** and formation of the corresponding cyclobutanes **3** (Figure 4), which were isolated as air-stable solids after solvent evaporation and recrystallization from CH_2_Cl_2_/*n*-pentane.

**Figure 5:**
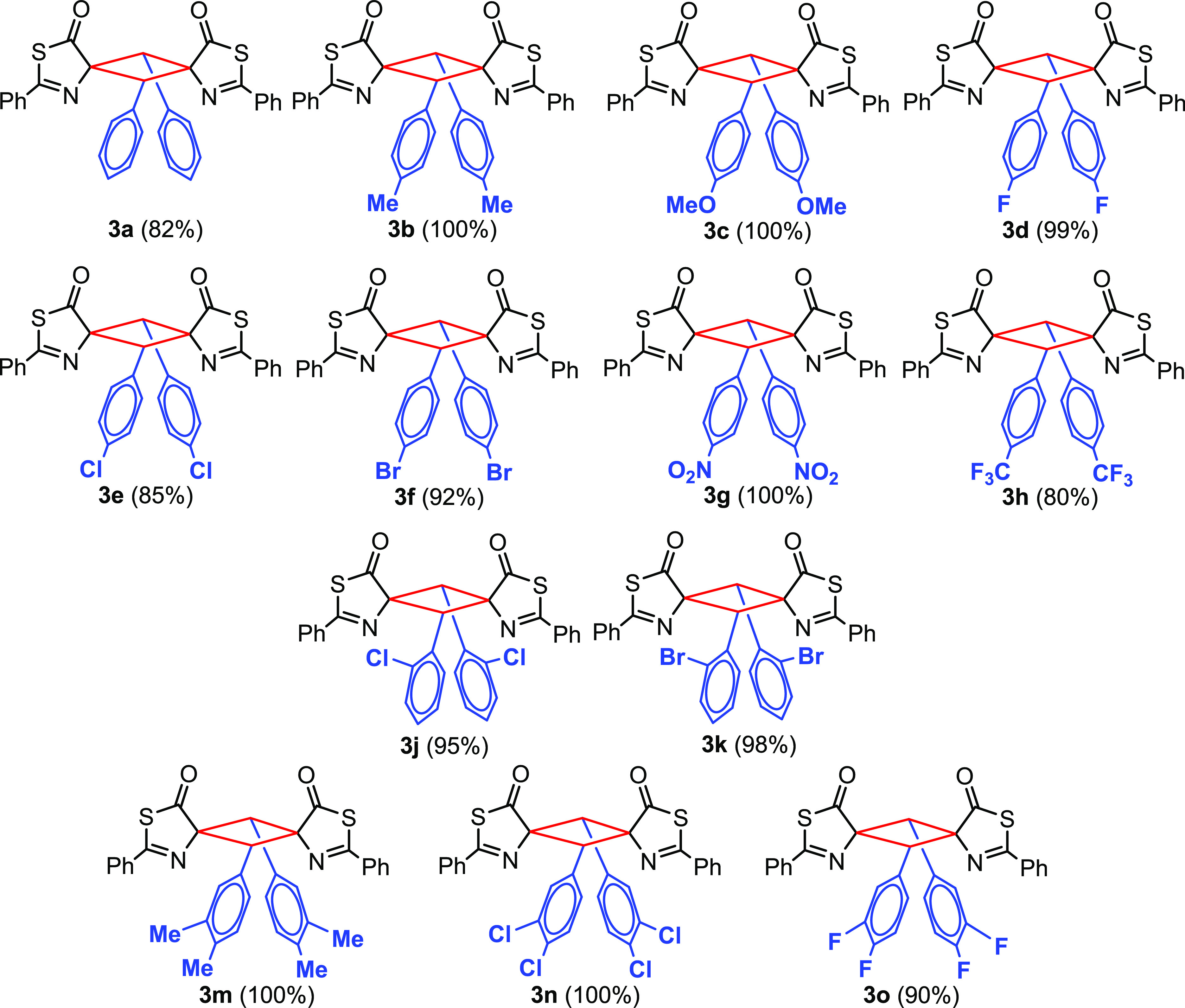
Scope of the [2 + 2]-photocycloaddition of thiazolones **2** to give cyclobutanes **3**.

The optimum reaction time for full conversion of **2** using the PCB, as determined by ^1^H NMR monitoring, was 72 h. This reaction time can be shortened to 24 h if a Kessil lamp (456 nm) is used instead, probably due to the higher photonic flux of the latter. The reaction also takes place in other solvents (for instance, methanol), giving the same yield of cyclobutanes **3**. Identical results were obtained in CH_2_Cl_2_ in the presence or absence of oxygen. No photocycloaddition was observed for **2i** and **2l**, and partial conversion was obtained for **2h**, despite the photonic flux used. The scope of the reaction (Figure 5) appears to be general as it takes place with full conversions and very good yields of isolated products in the presence of either electron-donating (Me, OMe) or electron-withdrawing (F, Cl, Br, NO_2_, CF_3_) substituents. A change in position of the substituents (*ortho* vs *para*) in the 4-arylidene ring is well tolerated (compare **3e** with **3j** or **3f** with **3k**), as is the presence of two substituents in the *meta*- and *para*-positions (**3m**–**3o**).

NMR analysis of the cyclobutanes **3** represented in Figures 4 and 5 showed that they were mainly obtained as single isomers (**3a**, **3g**, **3h**) or as mixtures of two isomers with molar ratios of 80:20 or higher (**3b**, **3d**, **3e**, **3f**, **3m**, **3n**, **3o**; see Table 1 and Experimental Section). Given that the photocycloaddition of thiazolones **2** can afford up to 11 different isomers, the stereoselectivity of the process presented here is remarkable. A comparison of these results with those obtained with related substrates shows that the use of thiazolones results in a more selective process. For instance, we have reported the synthesis of cyclobutanes by direct [2 + 2]-photocycloaddition of 4-arylidene-5(4*H*)-oxazolones **1** (Figure 2, past work, path a).^41^ This reaction takes place for only a narrow range of substituents, and the corresponding cyclobutanes were obtained as mixtures of four different isomers with similar molar ratios. However, in the case of the thiazolones studied here, the scope is much wider, and the stereoselectivity is markedly higher.

**Table 1:** Yields of Cyclobutanes **3**, Obtained as Mixtures of Isomers, and Composition of the Mixtures

	**3a**	**3b**	**3c**	**3d**	**3e**	**3f**	**3g**	**3h**	**3j**	**3k**	**3m**	**3n**	**3o**
yield	82	100	100	100	85	92	100	80^a^	95	98	100	100	90
ε (%)	100	90	59	91	96	83	100	100	71	65	85	91	94
α (%)	0	10	25	9	4	17	0	0	29	35	15	9	6
others (%)			9:7										

a Maximum conversion achieved.

**Figure 6:**
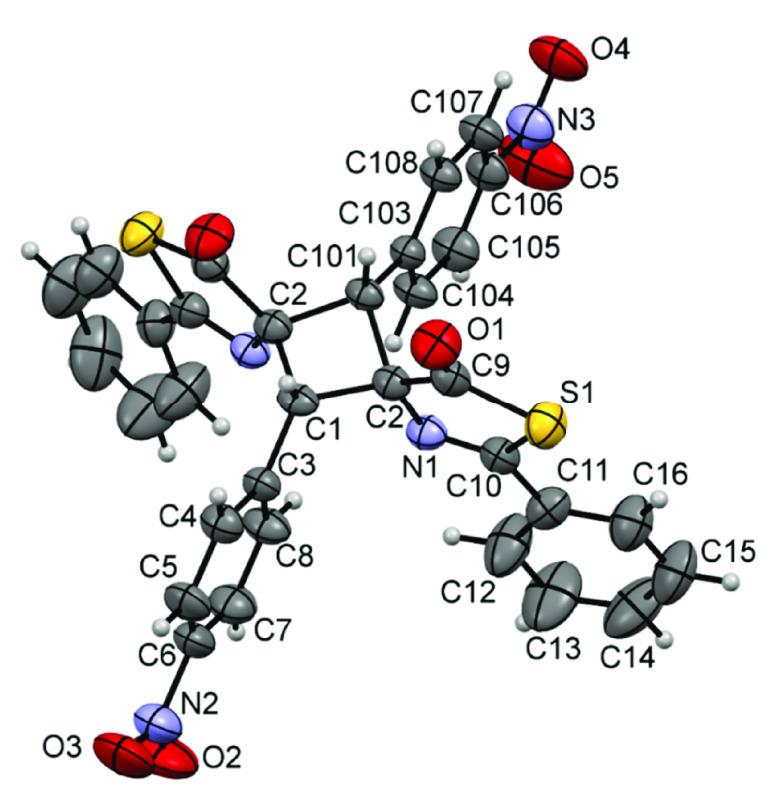
X-ray crystal structure of cyclobutane **3g.** Thermal ellipsoids are drawn at 50% probability.

**Figure 7:**
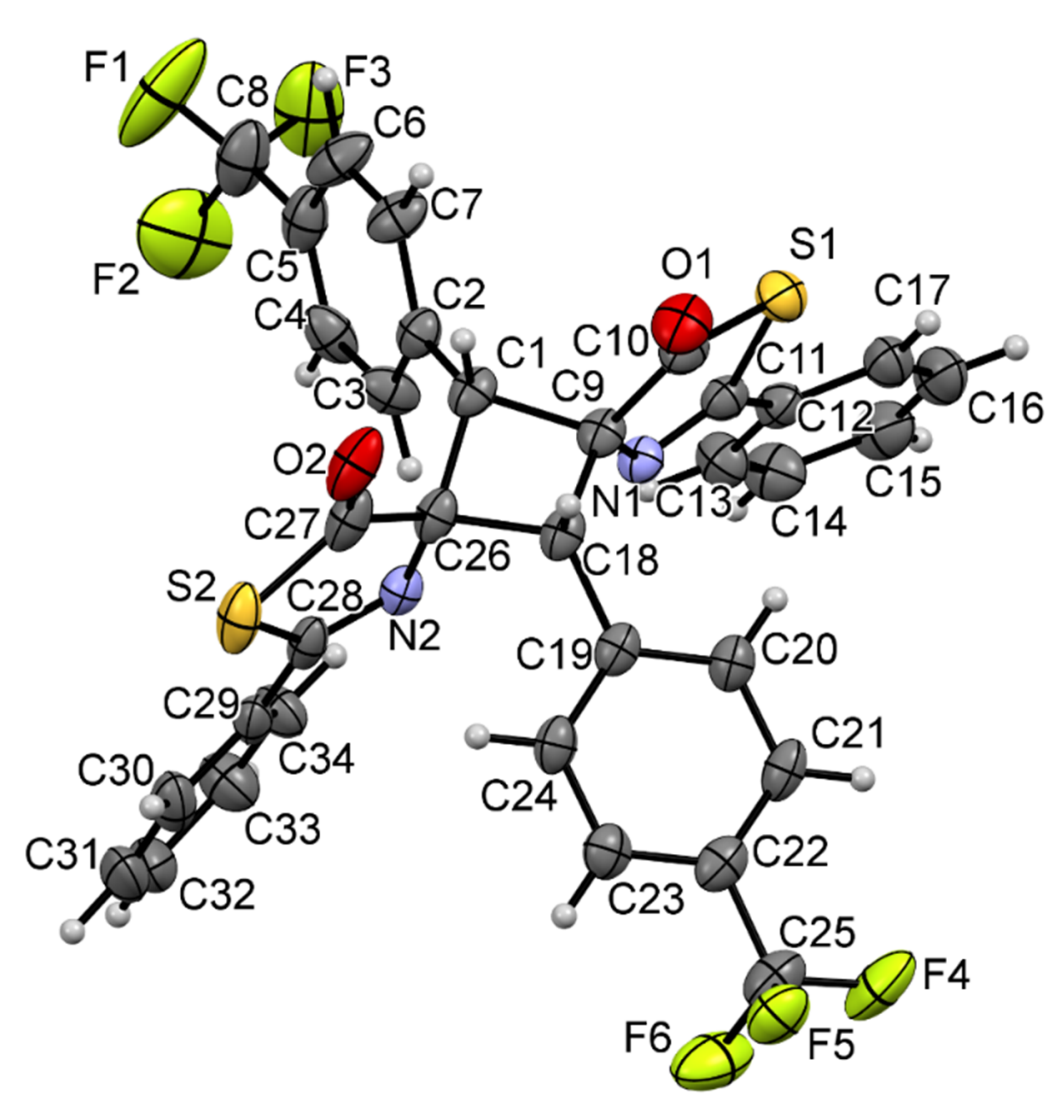
X-ray crystal structure of cyclobutane **3h**. Thermal ellipsoids are drawn at 50% probability.

**Figure 8:**
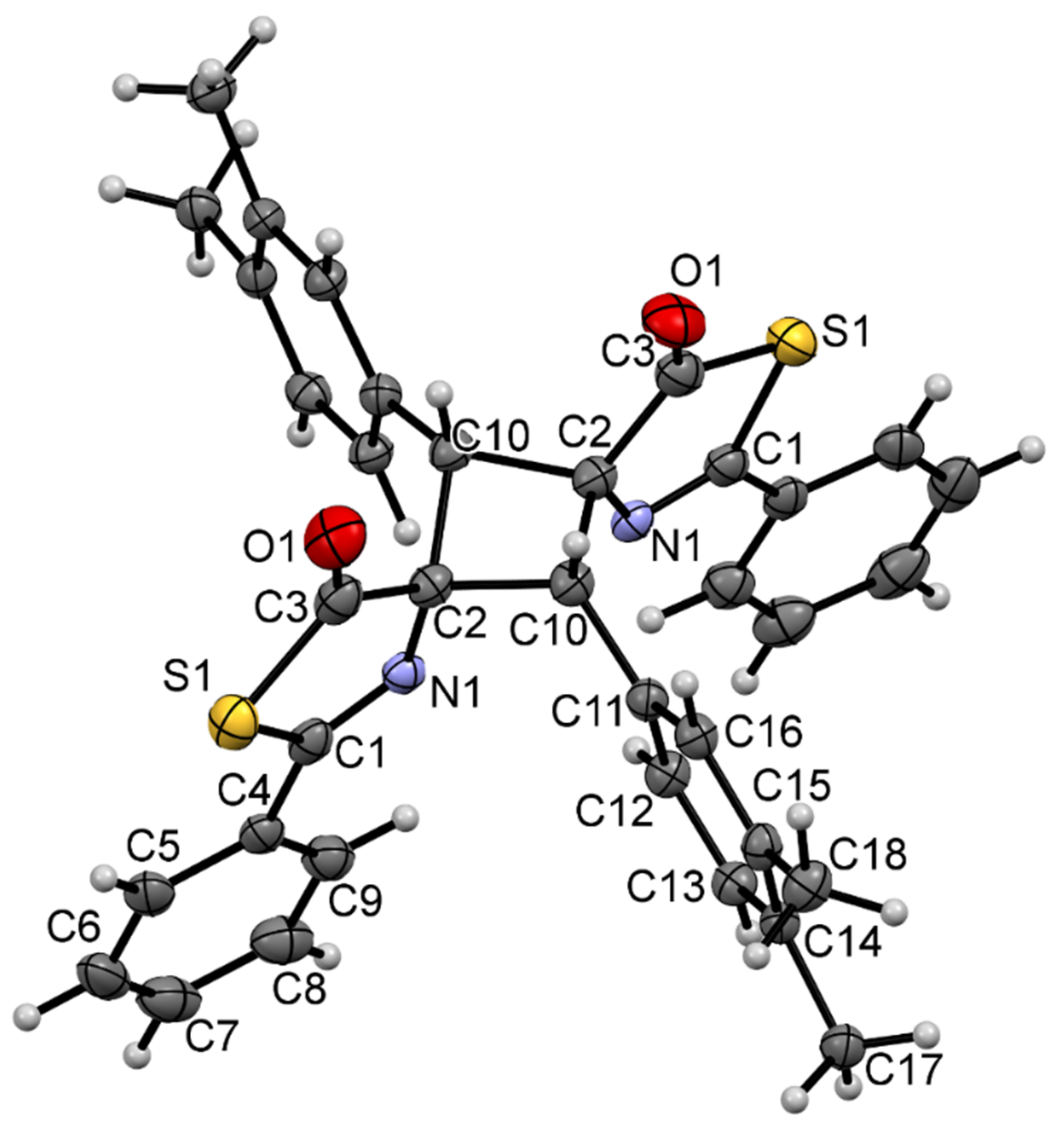
X-ray crystal structure of cyclobutane **3m**. Thermal ellipsoids are drawn at 50% probability.

The NMR data for all cyclobutanes **3** studied showed the presence of species with high symmetry but were not conclusive because several isomers could fit with the experimental NMR data. As such, full characterization of the main isomer for cyclobutanes **3** was achieved by determining the X-ray crystal structures of derivatives **3g**, **3h**, and **3m**, which are shown in Figures 6, 7, and 8, respectively.

All structures clearly show the formation of the cyclobutane core by [2 + 2]-cycloaddition of the respective thiazolones. The isomer characterized in all three cases is the ε-isomer (ε), according to the isomer assignment of Stoermer and Bachér,^67,68^ which is formed by the 1,3-head-to-tail coupling of two *Z*-thiazolones in a *syn* orientation. This ε-isomer is the same as that characterized as the major isomer in the [2 + 2]-photocycloaddition of oxazolones, thus suggesting that the dimerization of oxazolones and thiazolones follows the same orientation. The three structures are very similar, with the cyclobutane core showing the 1,2-*cis*-2,3-*cis*-3,4-*cis* configuration. The cyclobutane rings are not planar and exhibit dihedral angles of C1-C2-C101-C2 (**3g**) = 18.1(3)°, C1-C9-C18-C26 (**3h**) = 19.7(3)°, and C2-C10-C2-C10 (**3m**) = 22.4(3)°, which are similar to those found in related cyclobutanes.^41^ In addition, the values for the remaining bond distances (Å) and angles (°) are in the usual range of values found in the literature for related structural arrangements.^38,41,43,69−74^

The characterization of the main isomer in compounds **3g**, **3h**, and **3m** as the ε-isomer allows us to extrapolate this assignment to the remaining cyclobutanes **3** prepared here even though we were unable to characterize them all by X-ray diffraction. An additional argument in this direction is the comparison of the NMR chemical shifts for the ^1^H and ^13^C nuclei of the cyclobutane ring in **3**, which exhibit values that appear in very narrow ranges, thereby suggesting quite similar environments.

### Effect of Lewis Acids (BF_3_) on the [2 + 2]-Photocycloaddition of Thiazolones **2**

**Figure 9:**
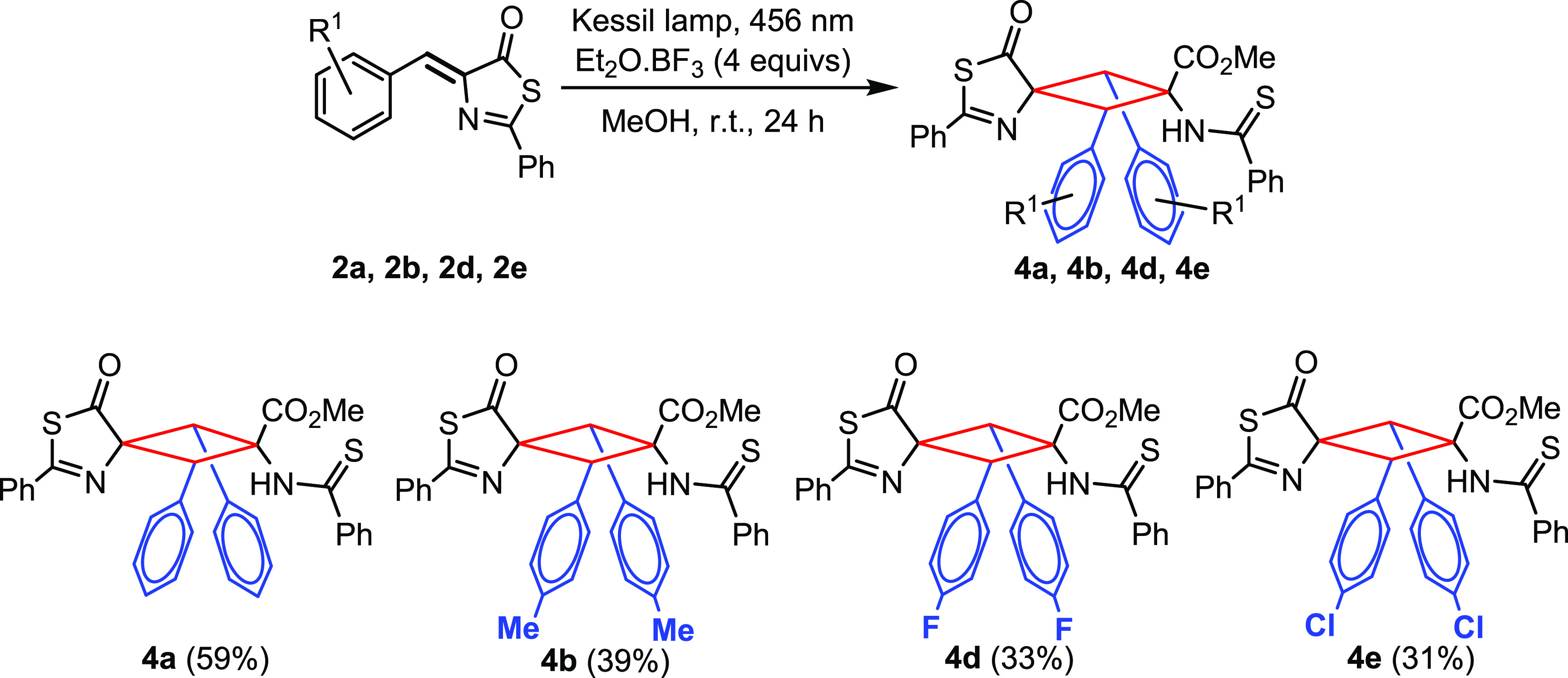
Photocycloaddition of thiazolones **2** in methanol in the presence of the Lewis acid BF_3_·OEt_2_

As mentioned in the Introduction, the presence of Lewis acids can change the rate, orientation, and/or selectivity of a given reaction.^23,28,53−62^ In order to determine the influence of Lewis acids in this particular photochemical reaction, we irradiated suspensions of selected examples of thiazolones **2** with blue light (Kessil lamp, 456 nm), in methanol, in the presence of BF_3_·OEt_2_. The optimized amount of BF_3_·OEt_2_ was four equivalents with respect to thiazolone **2**. After irradiation for 24 h, the conversion of **2** was complete, and cyclobutanes **4** (represented in Figure 9) were isolated by simple filtration of the resulting suspensions (see Experimental Section and Supporting Information). The full characterization of these cyclobutanes **4** showed that they were obtained as single isomers in all cases studied (see below). As such, the reaction in the presence of a Lewis acid (BF_3_) gives a cyclobutane with the same orientation as in its absence but with full stereoselectivity, thereby improving our previous results. However, no acceleration was observed. As a result, a more restricted scope was examined.

The characterization of compounds **4** by HRMS and NMR spectroscopy clearly shows the formation of a cyclobutane core in which one of the thiazolones remains unchanged while the other has undergone a ring-opening reaction by methanolysis, thus giving the corresponding ester and thioamide fragments. The HRMS data for cyclobutanes **4a**, **4b**, **4d**, and **4e** reflect the dimerization of the corresponding thiazolones **2** and the incorporation of a molecule of methanol for each dimer of cyclobutane. The ^1^H NMR spectra of **4a–4e** show signals corresponding to the presence of a single isomer in each case; in other words, the reaction is totally stereoselective for the cases studied. The ring-opening reaction is evidenced by the observation of peaks due to the ester fragment (at around 3.8 ppm) and the NH proton (a broad signal at around 8.5–8.7 ppm) with a relative intensity of 3:1. The presence of a single peak for the two chemically equivalent cyclobutane CH protons (4.8–4.9 ppm, relative intensity 2) shows that, despite the loss of symmetry produced by the ring-opening reaction, the two ArC(H) groups of the cyclobutane are still equivalent. The ^13^C NMR spectra of **4a–4e** also show signals in agreement with the ring-opening reaction of the only heterocycle. In this respect, peaks at around 205–206 (SC=O) and 166–167 ppm (SC=N) suggest the presence of a thiazolone ring, while peaks at around 198 (HNC=S) and 169 ppm (COO) confirm the presence of ester and thioamide groups. Moreover, three different signals are observed for the cyclobutane ring, one for the two chemically equivalent CH carbons (in the 55 ppm region) and two for the quaternary carbons (at about 90 and 67 ppm).

**Figure 10:**
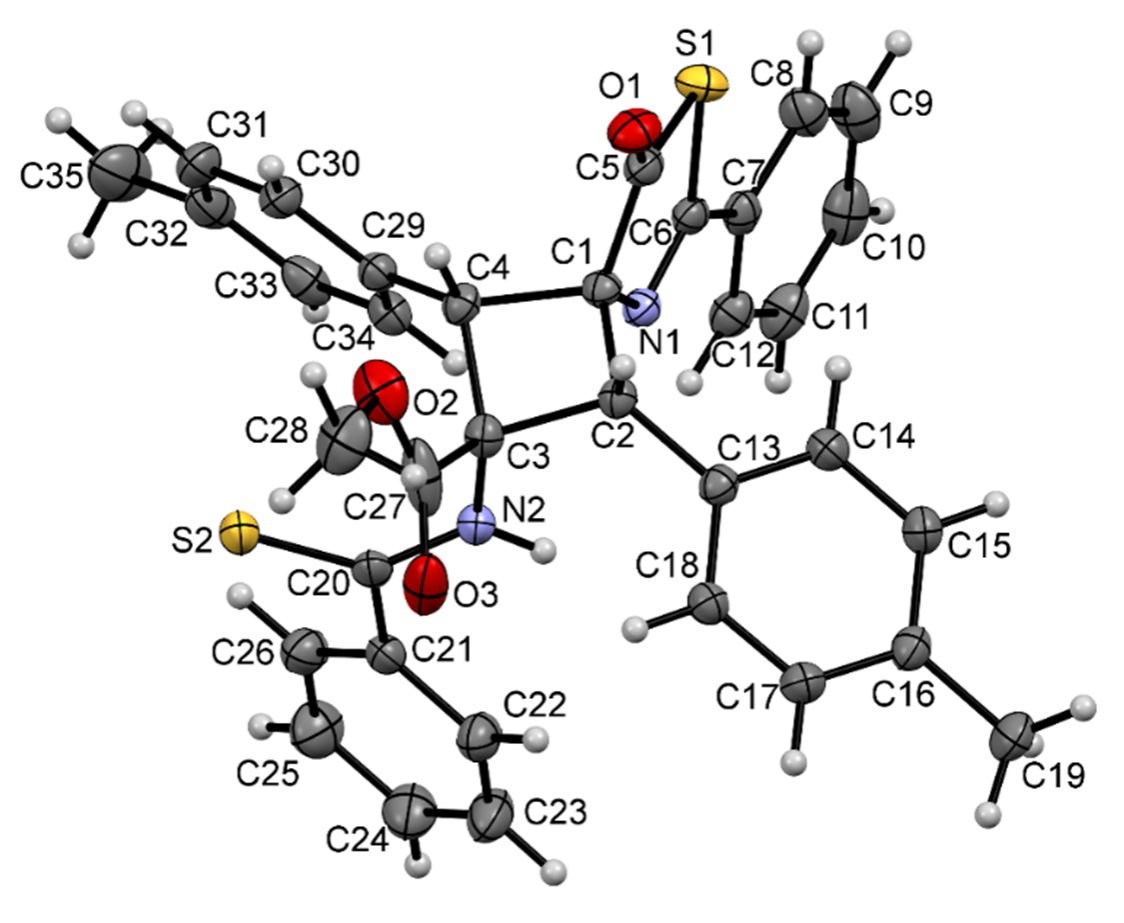
X-ray crystal structure of cyclobutane **4b**. Thermal ellipsoids are drawn at 50% probability.

As discussed for cyclobutanes **3**, there is more than one isomer whose structure fits with the HRMS and NMR data for cyclobutanes **4**. In this case, there are four possible structures (*syn*-ε, *syn*-epi, *anti*-epi, and *syn*-peri);^41^ therefore, the X-ray crystal structure of **4b** was determined to complete the structural characterization. Crystals were obtained by slow diffusion of *n*-pentane into a solution of crude **4b** in CH_2_Cl_2_ at −18 °C, and the structure obtained is shown in Figure 10.

The structure of cyclobutane **4b** shows that the isomer obtained is the ε-isomer, which is formed by the 1,3-head-to-tail coupling of two thiazolones in a *syn* orientation. The cyclobutane core of **4b** is not planar, showing a value for the C4-C1-C2-C3 dihedral angle of 19.1(1)°, which is identical to those found in the structures of **3g**, **3h**, and **3m**. Other internal parameters of the cyclobutane ring are also identical (within experimental error) to those found in **3g**, **3h**, and **3m**. The structure also confirms the ring-opening reaction by methanolysis at one of the thiazolones and the corresponding presence of ester and thioamide fragments, both of which show bond distance and angle values in agreement with those found in the literature for similar types of bonds.^74^

In light of the crystal structure of **4b**, it can be concluded that photocycloaddition in methanol in the presence of BF_3_ gives cyclobutanes **4**, and that cyclobutanes **3** are formed in the absence of BF_3_. This reaction takes place with exactly the same orientation because the same isomer out of the 11 possible cyclobutane isomers is obtained in both cases (ε-isomer). However, the reaction affords slightly different compounds as **3** contains two unaltered thiazolone rings, whereas **4** contains only one. This suggests that the Lewis acid BF_3_ has a small but important influence as, although neither the rate nor the orientation of the reaction are affected, the ring-opening reaction is favored in the presence of the Lewis acid.

**Figure 11:**
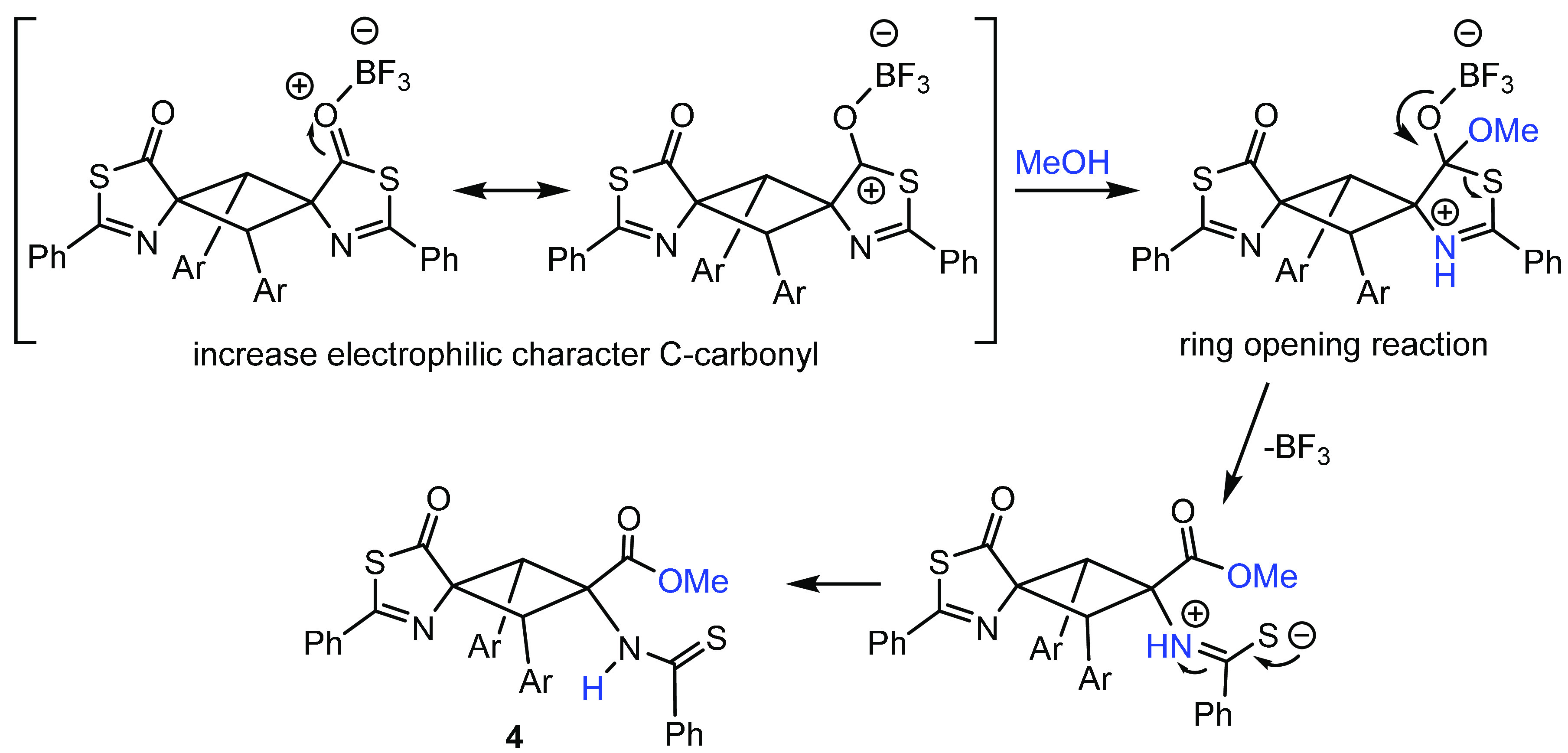
Proposal of the mechanism for the BF_3_-promoted ring-opening reaction.

We have studied the interaction between thiazolones **2** and BF_3_ in MeOH both in the ground state and in the excited state. Thiazolone **2b** was selected as a representative example. To analyze the interaction in the ground state, a solution of **2b** in CD_3_OD was treated with increasing amounts of BF_3_·OEt_2_ until the molar ratio 1:4 was reached, and the result of each addition was monitored by ^1^H NMR spectroscopy (see Supporting Information). A comparison of the NMR spectra showed that there is no detectable interaction on the NMR time scale as all spectra were identical, and no signal underwent a change in its chemical shift. This suggests that either the interaction is very weak or it occurs to such a small extent that it cannot be detected by NMR spectroscopy. As for the excited state, the fluorescence of thiazolone **2b** was examined in the absence and presence of BF_3_·OEt_2_ (Supporting Information) in methanol. Thiazolone **2b** is fluorescent and shows an emission maximum at 459 nm when excited at 390 nm. After the addition of BF_3_·OEt_2_ (1–4 equiv), no changes were detected in either the maximum of the emission or its intensity. These results suggest that the Lewis acid does not have a marked influence on either the excited state or the ground state. However, the different structure of cyclobutanes **3** and **4** shows that an interaction with the Lewis acid must occur at some point in the reaction, and given the lack of reaction observed between **2b** and BF_3_, this probably occurs after the formation of cyclobutane **3**. To check this, we treated cyclobutane **3a** with BF_3_·OEt_2_ in methanol for 24 h at room temperature and in the dark. The formation of **4a** was evident after this time, thus suggesting that the role of BF_3_ is related to the promotion of the ring-opening reaction, as shown in Figure 11, rather than to the [2 + 2]-photocycloaddition.

### Ring-Opening Reaction of Thiazolones **2**: Synthesis of 4,5-Dihydrothiazoles (**5**, **6**) and Thiazoles (**7**)

**Figure 12:**
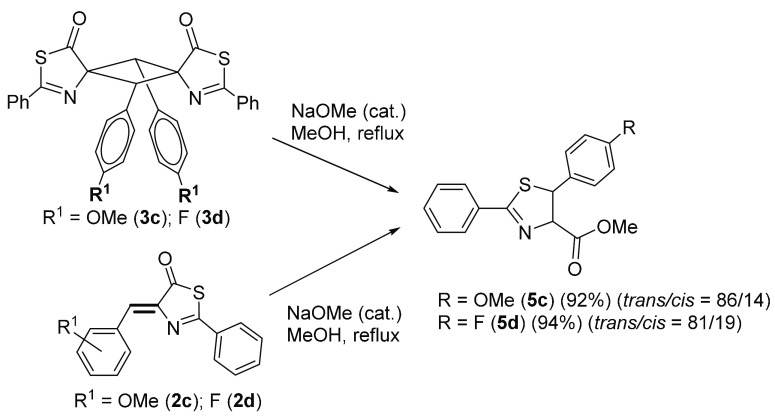
Synthesis of 4,5-dihydrothiazoles **5** by treatment of cyclobutanes **3** with NaOMe in MeOH.

The easy ring-opening reaction undergone by **3** to give **4** prompted us to study the opening of the remaining thiazolone ring in **4**. The opening of both heterocycles should produce cyclobutanes structurally analogous to diaminotruxillic bis-amino acids but containing sulfur in their structure. In a first attempt, we tested the classical process, namely, heating cyclobutanes **3c** and **3d** with a catalytic amount of a base (NaOMe) in alcohol (MeOH).^75^ Surprisingly, this reaction afforded the 4,5-dihydrothiazoles **5c** and **5d** as a mixture of *cis*- and *trans*-diastereoisomers, as shown in Figure 12 (upper reaction).

The transformation shown in the upper reaction of Figure 12 suggests that the reaction is most likely related to the intrinsic reactivity of the exocyclic C(H)=C bond in thiazolone **2** rather than to that of the cyclobutane skeleton in **3**. For that reason, we studied the reactivity of thiazolones **2c** and **2d** with NaOMe in MeOH as a solvent, and the results are also shown in Figure 12 (lower reaction). As expected, the treatment of **2c** and **2d** with base in alcohol gave the dihydrothiazoles **5c** and **5d** in virtually the same yields and diastereomeric excess as obtained when **3c** and **3d** were used as precursors instead. This suggests that, when heating under basic conditions, cyclobutanes **3** are not stable and a retro-[2 + 2] reaction can take place to regenerate thiazolones **2**, which subsequently react with a base to give **5**.

Compounds **5c** and **5d** were characterized by HRMS and NMR spectroscopy. The ^1^H NMR spectra show two characteristic AB spin systems centered around 5.4 ppm, assigned to the two aliphatic protons of the (N)CH—CH (S) moiety. The value of the ^3^*J*_HH_ coupling constant between these two protons is diagnostic for the determination of the configuration of each diastereoisomer. Thus, the major species shows a value for the ^3^J_HH_ coupling constant of 6.5 Hz, which is typical for *trans* geometries, whereas the value found for the minor isomer is around 10 Hz, thus suggesting a *cis* arrangement.^76^ The higher abundance of the *trans*-isomer is in good agreement with the lower intramolecular repulsions in this isomer. These two protons correlate (^1^H–^13^C HSQC spectra) with two C atoms at around 87 (CHN) and 55 ppm (CHS), thus showing their aliphatic character. In addition, the peak at around 194–196 ppm for thiazolones **2** (around 205–208 ppm in cyclobutanes **3**), assigned to the thiocarbonyl group (S—C=O), has disappeared, and a new peak is now observed in the 170–172 ppm region. This suggests the formation of a new heterocycle, namely the 4,5-dihydrothiazole shown in Figure 12.

**Figure 13:**
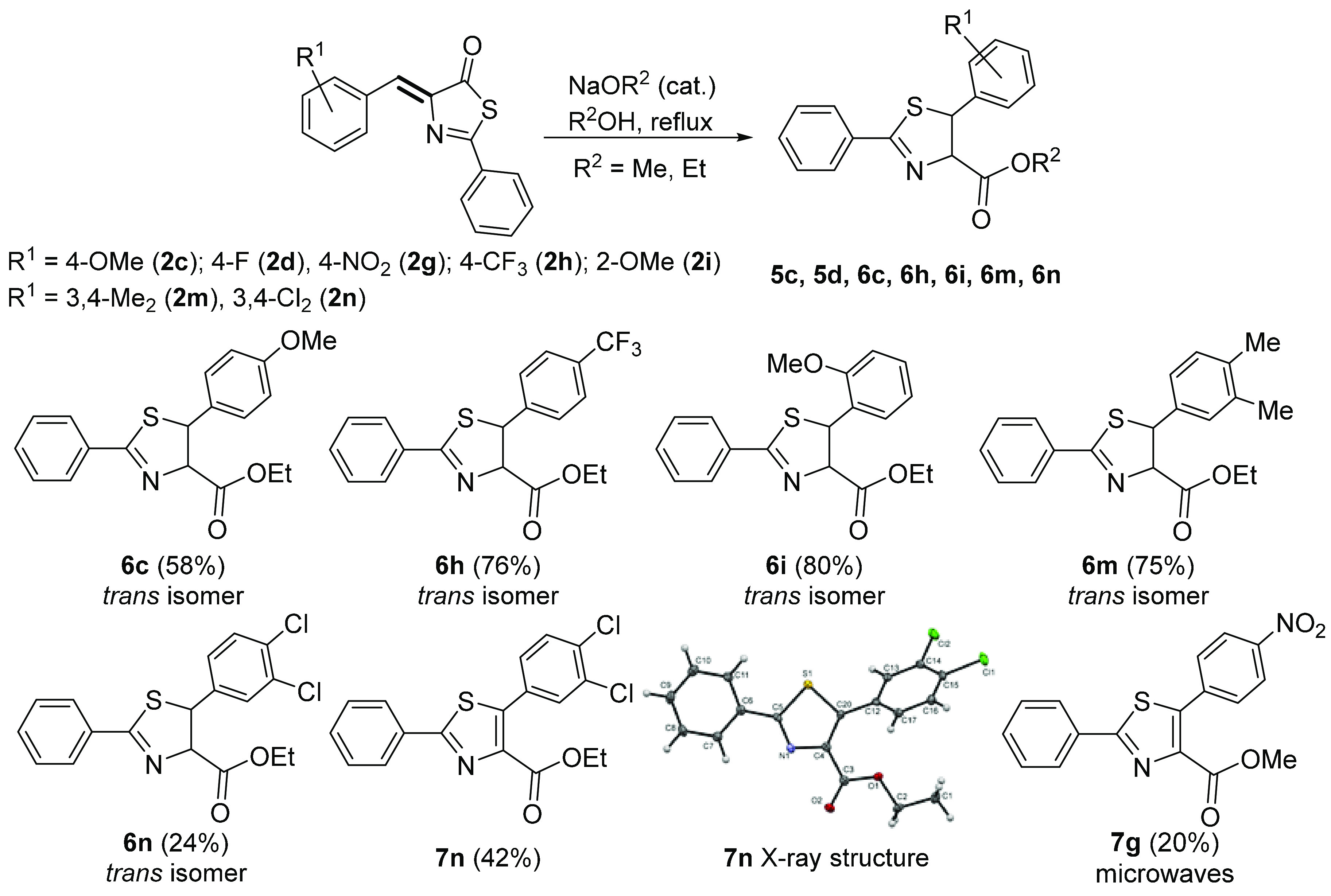
Reactivity of thiazolones **2** with base in alcohol to give dihydrothiazoles (**5** and **6**) and thiazoles (**7**). Yields (%) correspond to the pure isolated *trans*-isomer.

This synthetic method for the preparation of dihydrothiazoles from 4-arylidene-5(4*H*)thiazolones **2** is novel as, to the best of our knowledge, only one previous example has been reported in the literature.^77^ In that case, the reaction was performed using NaOH in water to afford the corresponding carboxylic acid.^77^ Dihydrothiazoles are interesting materials due to their properties, and different synthetic methods have been reported for their preparation, most of them starting from aminothiols.^78−89^ Dihydrothiazoles are versatile intermediates for the synthesis of high-value-added compounds, for instance β-cysteine and derivatives,^90−94^ are present in flavours^95^ and in natural products,^96−99^ and show a remarkable pharmacological activity.^100−102^ For all of these reasons, we decided to explore this almost unprecedented synthesis of dihydrothiazoles from thiazolones **2** in more detail. The results are presented in Figure 13.

The treatment of thiazolones **2** with NaEtO in ethanol at reflux temperature gave the corresponding dihydrothiazoles **6**, as shown in Figure 13. After the reaction, the ethanol was evaporated to dryness, the residue was extracted with CH_2_Cl_2_, and all insoluble materials were removed by filtration. Evaporation of the solvent gave an oily residue, which was shown by ^1^H NMR spectroscopy to be a mixture of the two diastereoisomers of dihydrothiazoles **6** in a *trans*/*cis* molar ratio in the range 86:14 to 90:10. As such, the reaction takes place with a remarkable diastereomeric excess, which is even higher than when methanol was used as a solvent. The major *trans*-isomer could be isolated in pure form from that mixture by column chromatography (see Experimental Section), while the minor *cis*-isomer could not be isolated in sufficient quantity to be characterized in most cases. The reaction shows an adequate scope, as it works in moderate to good yields for both electron-donating (**6c**, **6i**, **6m**) and electron-withdrawing (**6h**, **6n**) substituents in the 5-aryl ring. In addition, the reaction tolerates substituents at the *ortho*-, *meta*-, and *para*-positions of the aryl ring. In the case of thiazolone **2n**, the formation of the thiazole **7n** as the major reaction product was observed. This compound was also purified and separated from **6n** by column chromatography. The absence of the diagnostic AB spin system at around 5.3–5.4 ppm in the ^1^H NMR spectrum of **7n**, and the corresponding carbons at 86 ppm and 55 ppm in the ^13^C NMR spectrum, as well as the presence of two new quaternary C atoms at 142 ppm, confirms the formation of thiazole **7n**, the molecular structure of which determined by X-ray diffraction methods (Figure 13). The formation of thiazole **7n** in the reaction medium is likely due to aerobic oxidation of the precursor dihydrothiazole, which is a well-known reaction.^103^ When microwaves were used instead of a conventional heating source, as in the case of the 4-NO_2_-substituted thiazolone **2g**, complete transformation into thiazole **7g** was observed in just one minute, thus meaning that formation of the dihydrothiazole is accelerated, as is its oxidation. For that reason, only conventional heating was used.

**Figure 14:**
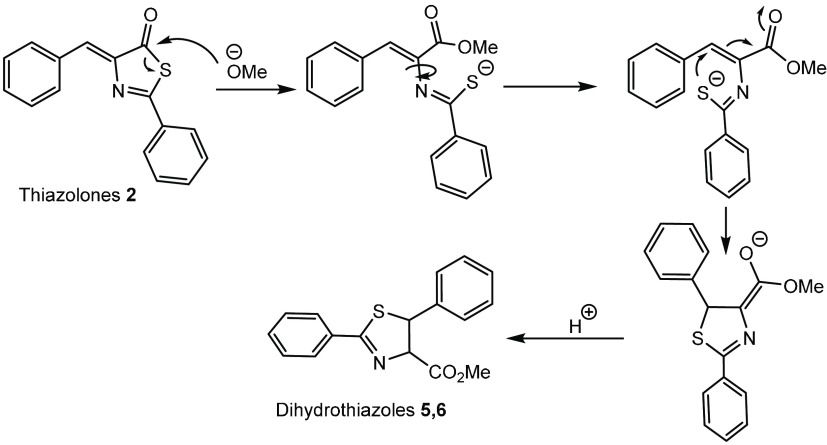
Mechanistic proposal for the synthesis of dihydrothiazoles **5** (or **6**) from thiazolones **2**.

The mechanism proposed for the synthesis of dihydrothiazoles **5** or **6** from thiazolones **2** is shown in Figure 14. The reaction seems to start with a nucleophilic attack of the alkoxide at the carbonyl carbon to form the corresponding ester group and the thioamidate fragment. Subsequent intramolecular S-attack of the sulfide at the C(H) carbon of the exocyclic C(H)=C double bond, followed by protonation of the stabilized enolate generated, results in the formation of the final dihydrothiazoles.

**Figure 15:**
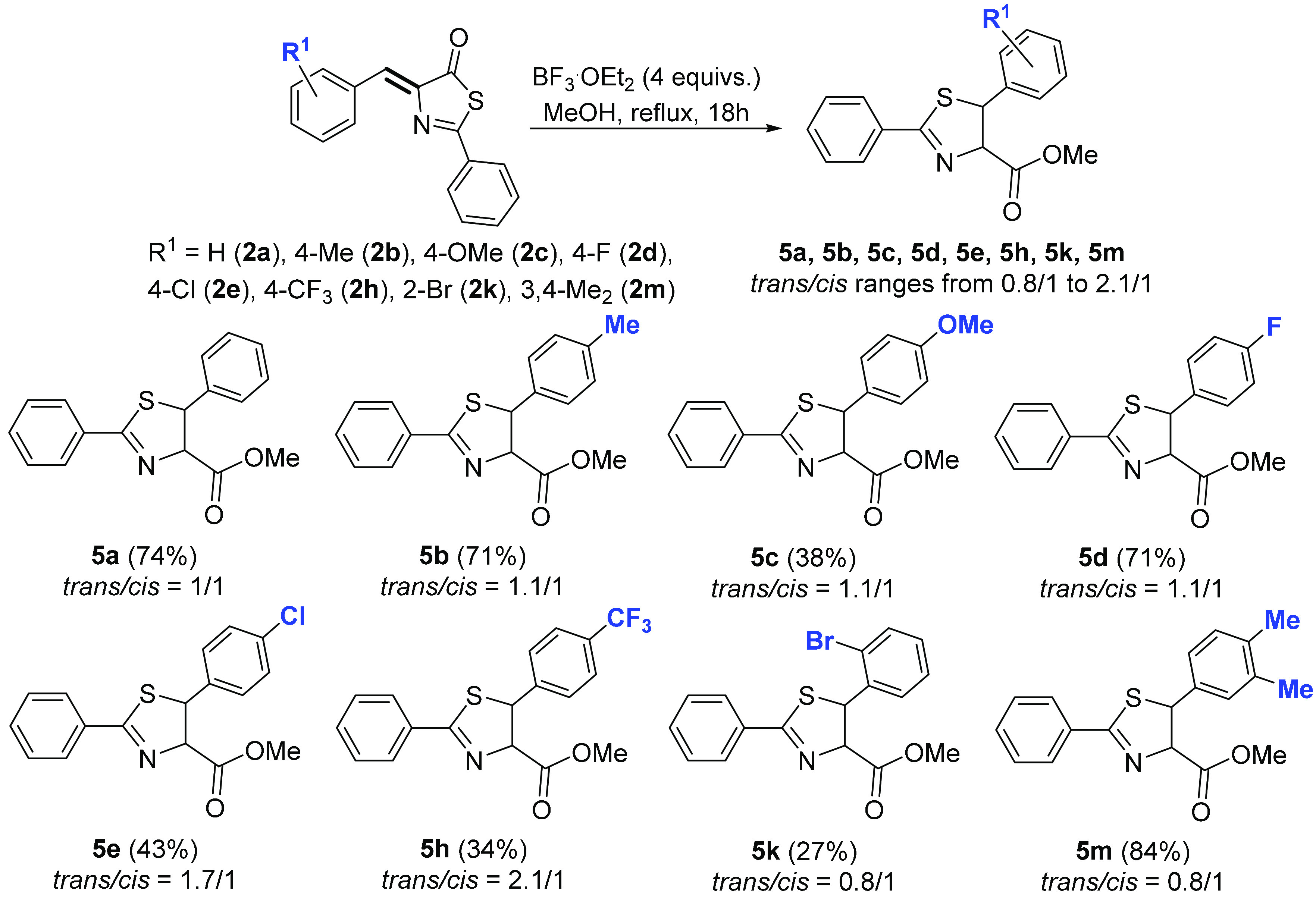
Reactivity of thiazolones **2** with MeOH in the presence of BF_3_; synthesis of dihydrothiazoles **5**.

The easy ring-opening reactions undergone by cyclobutanes **3** to give cyclobutanes **4** shown in Figures 9 and 11 suggest that the Lewis acid BF_3_ fosters the attack of nucleophiles at the carbonyl carbon, which should, in principle, favor the formation of dihydrothiazoles from thiazolones. With this in mind, we attempted the reaction of thiazolones **2** with methanol, in the presence of the Lewis acid BF_3_·OEt_2_ and in the absence of a base. The results of these reactions are shown in Figure 15.

**Figure 16:**
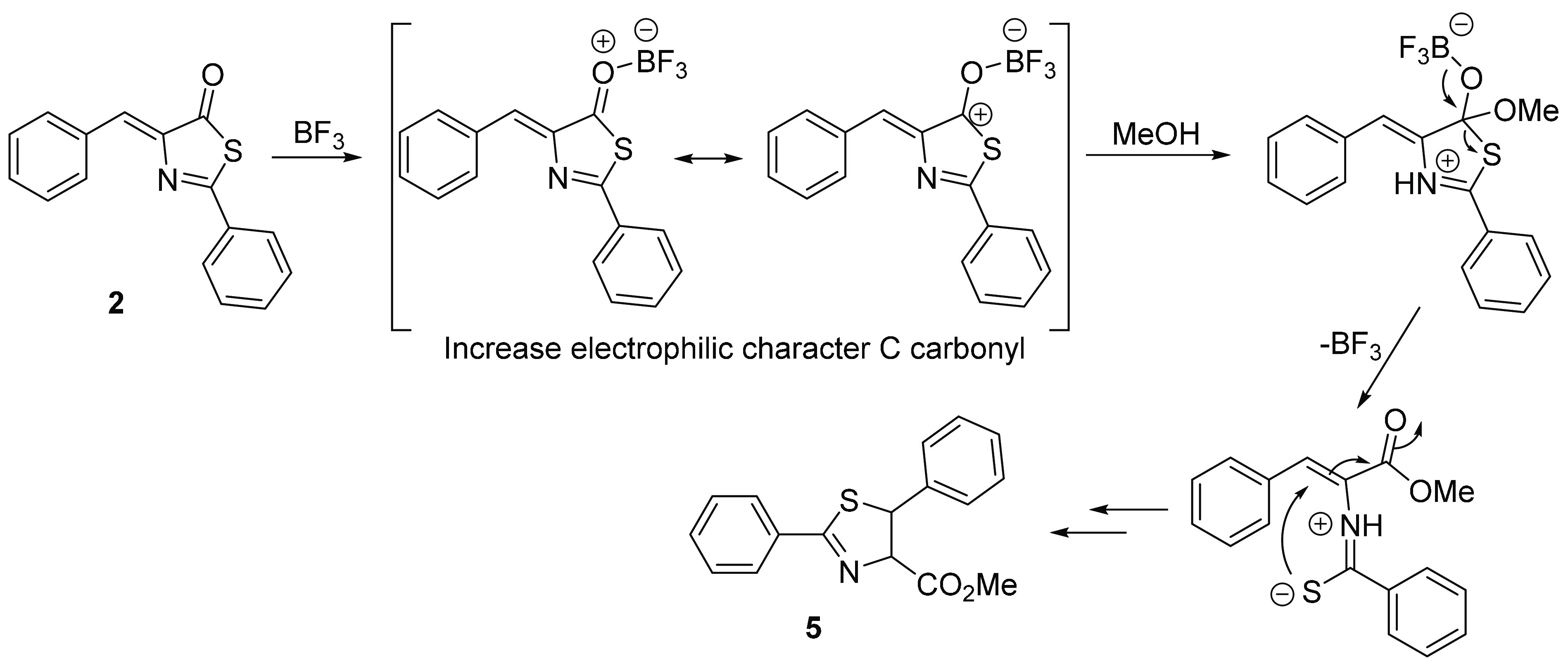
Mechanistic proposal for the formation of dihydrothiazoles in the presence of BF_3_.

The treatment of thiazolones **2** with BF_3_·OEt_2_ (4 equiv) in methanol at reflux temperature for 18 h resulted in the formation of the corresponding dihydrothiazoles **5** in good to moderate yields. The reaction does not take place at room temperature, with thiazolone **2** being recovered unchanged. At shorter reaction times (2 h) in refluxing methanol, we observed low conversions (less than 10%), along with the formation of **5** in a more or less equimolar mixture of the two diastereoisomers (range between 1.2:1 to 1.5:1). No reaction was observed in refluxing methanol in the absence of BF_3_. As such, in the presence of BF_3_, both high temperatures and long reaction times are needed to achieve full conversion of thiazolones **2** into dihydrothiazoles **5**, although a base is no longer required. The reaction is tolerant to the presence of electron-donating (Me, OMe) and electron-withdrawing (F, Cl, Br, CF_3_) substituents at different positions of the aryl ring, thus meaning that the reaction shows an adequate scope. The main difference between this process and the reaction performed in the presence of a base (Figures 12 and 13) is the diastereoselectivity, which is moderate to high in the presence of a base but very low or even nonexistent in the presence of BF_3_. Unfortunately, we have no reasonable explanation for this finding. Our mechanistic proposal to explain the role of the Lewis acid in this reaction is presented in Figure 16. Coordination of the carbonyl oxygen to BF_3_ increases the electrophilic character of the carbonyl carbon (as in Figure 11), which is, therefore, more susceptible to attack by methanol. The decoordination of BF_3_ is followed by the formation of the sulfide, which can attack the exocyclic C(H)=C bond (see Figure 14).

## Conclusion

In summary, new (*Z*)-4-aryliden-5(4*H*)-thiazolones **2** have been prepared by treatment of oxazolones with thioacetic acid. These thiazolones have been shown to be convenient and versatile precursors for the synthesis of a wide variety of carbo- and heterocycles with high selectivity. The irradiation of thiazolones **2** with blue light (465 nm) results in the formation of diaminotruxillic-type cyclobutanes **3** by head-to-tail 1,3-*syn* coupling [2 + 2]-photocycloaddition of thiazolones **2**. The reaction shows a high stereoselectivity as cyclobutanes **3** are obtained mostly as the ε isomer. When the photochemical reaction is performed in the presence of a Lewis acid (BF_3_·OEt_2_), the reaction follows the same orientation (head-to-tail 1,3-coupling), but an additional ring-opening reaction is observed, due to bonding of the BF_3_, thus giving a different family of truxillic cyclobutanes **4**. The role of the Lewis acid is to foster the electrophilic character of the carbonyl carbon, thereby favoring the ring-opening reaction. In addition, treatment of **2** with base NaOR in alcohol ROH affords dihydrothiazoles **5**, **6** via a ring-opening reaction followed by S-intramolecular attack at the exocyclic C(H)=C bond. The reaction is highly stereoselective, with the *trans*-isomer being obtained as a major isomer in all cases studied. Dihydrothiazoles **5** can also be obtained by treatment of the thiazolone **2** with alcohol in the presence of BF_3_ but in the absence of a base. In this case, the reaction shows a broader scope, and it seems that the role of BF_3_ is to increase the electrophilic character of both the carbonyl carbon and the vinyl carbon.

## Experimental Section

### General Methods

All solvents used are commercial-grade and were used as received. All reactions were performed at open-air without special caution against the moisture and oxygen, except the syntheses of compounds **4**, which were carried out under Ar atmosphere using dry and deoxygenated methanol. Flash column liquid chromatographies were performed on silica gel (70–230 μm) or aluminum oxide 90 neutral (50–200 μm), eluting with the solvents specified on each case. Elemental analyses (CHNS) were carried out on a Perkin-Elmer 2400 Series II microanalyzer. Infrared spectra (4000–380 cm^–1^) were recorded on a Perkin-Elmer Spectrum-100 IR spectrophotometer. ^1^H, ^13^C, and ^19^F NMR spectra were recorded in CDCl_3_ or CD_2_Cl_2_ solutions at 25 °C on Bruker AV300 or AV500 spectrometers (δ in ppm, *J* in Hz) at ^1^H operating frequency of 300.13 or 500.13 MHz, respectively. ^1^H and ^13^C spectra were referenced using the solvent signal as internal standard, while ^19^F spectra were referenced to CFCl_3_. The assignment of ^1^H NMR peaks has been performed through standard 2D ^1^H–COSY (2K points in *t*_2_ using a spectral width of 12 ppm; 128 *t*_1_ experiments were recorded and zero-filled to 1K; for each *t*_1_ value, two scans were signal-averaged using a recycle delay of 1 s) and selective 1D 1H-NOESY experiments. Typical mixing times in the case of selective 1D-NOESY experiments were in the range 1.0–2.0 s, as a function of the irradiated signal. These values of optimized mixing times were set equal to the longitudinal relaxation time *T*_1_, determined using the inversion–recovery sequence. The ^13^C NMR peaks were identified using standard ^1^H–^13^C edited-HSQC and ^1^H–^13^C HMBC 2D experiments. In both cases, 4K points in *t*_2_ using spectral widths of 10 ppm (^1^H) and 200 ppm (^13^C) were used, with averaged values of the coupling constants ^1^*J*_CH_ = 145 Hz and long-range ^*n*^*J*_CH_ = 10 Hz. Typically, 128 *t*_1_ experiments were recorded and zero-filled to 1 K. For each *t*_1_ value, 8 (HSQC) or 32 (HMBC) scans were signal-averaged using a recycle delay of 1 s. ESI (ESI^+^) mass spectra were recorded using an Esquire 3000 ion-trap mass spectrometer (Bruker Daltonic GmbH) equipped with a standard ESI/APCI source. Samples were introduced by direct infusion with a syringe pump. Nitrogen served both as the nebulizer gas and the dry gas. MALDI mass spectra were recorded using a Bruker MicroFlex^TM^ or a Bruker AutoFlex^TM^III spectrometer, equipped with a time-of-flight mass analyzer, and using DIT (dithranol) as a matrix. The sample was dissolved in CH_2_Cl_2_. The HRMS mass spectra were recorded using a MicroToF Q, API-Q-ToF ESI with a mass range from 20 to 3000 *m*/*z* and mass resolution of 15000 (fwhm). The absorption spectra in the UV–visible region were measured on a Thermo Scientific Evolution 600 UV–vis spectrophotometer using quartz SUPRAXIL cuvettes, light path 10 mm. The oxazolones **1a–1o** used as starting materials were synthesized according to published methods.^104−113^

### Irradiation Setup

The irradiation setup consists of a round-bottom flask irradiated by either a printed circuit board (PCB) formed by 24 LEDs bulbs (Topbright) of a 10 mm diameter, each irradiating at 465 nm, or a commercial Kessil lamp irradiating at 456 nm. The LEDs are serially connected in blocks of 6. The output power per LED unit (blue, 465 nm) is 250 kmcd. The optical output power of the PCB of LEDs measured with a photometer (PM100D, Thorlabs) was 1 W, so the maximal power provided by the PCB is 24 W. The PCB (dimensions: 7 cm × 6 cm) and the flask are placed inside a custom-built setup for fixing the light source and the sample container and dissipate the excess heating. A concave mirror is placed in front of the PCB to maximize the light that irradiates the LEDs. The Kessil lamp is the PR160L-456 nm model, with a maximal power of 40 W. The intensity of the lamp can be tuned, and different powers were tested. Those specified in each case gave the maximum yield.

### X-ray Crystallography

Single crystals of **3g**, **3h**, **4b**, and **7n** of suitable quality for X-ray diffraction measurements were grown by slow diffusion of *n*-pentane into CH_2_Cl_2_ solutions of the crude product at −18 °C for several weeks, while those of **3m** were obtained by slow evaporation of a solution of the product in CH_2_Cl_2_. A single crystal was mounted in each case at the end of a quartz fiber in a random orientation. The crystal was fixed to the fiber with epoxy resin (**3g**) or covered with perfluorinated oil and placed under a cold stream of N_2_ gas (**3h**, **3m**, **4b**, **7n**). The data collections were performed at 293(2) K on an Oxford Diffraction Xcalibur Sapphire3 diffractometer (**3g**) or at 100(2) K on Bruker D8 Venture (**3h**, **3m**) or Bruker APEX CCD (**4b**, **7n**) diffractometers using graphite-monochromated Mo Kα radiation (λ = 0.71073 Å). A hemisphere of data was collected based on ω-scan and φ-scan runs. The diffraction frames were integrated using the programs CrysAlis RED^114^ and SAINT,^115^ and the integrated intensities were corrected for absorption with SADABS.^116^ The structures were solved and developed by Fourier methods.^117^ All non-hydrogen atoms were refined with anisotropic displacement parameters. The H atoms were placed at idealized positions and treated as riding atoms. Each H atom was assigned an isotropic displacement parameter equal to 1.2 times the equivalent isotropic displacement parameter of its parent atom. The structures were refined to F_o_^2^, and all reflections were used in the least-squares calculations.^118^ CCDC 1912941 (**3g**), 1912942 (**7n**), 1958992 (**3h**), 1958993 (**3m**), and 1999650 (**4b**) contains the supplementary crystallographic data for this paper and can be obtained free of charge from The Cambridge Crystallographic Data Centre via www.ccdc.cam.ac.uk/data_request/cif.

#### General Procedure for the Synthesis of (Z)-2-Phenyl-4-aryliden-5(4*H*)-thiazolones **2a**–**2o**

The synthesis of the thiazolones **2a–2o** has been carried out following the same experimental procedure as that reported by Rao and Filler,^46^ which in turn is based in the original work of Behringer et al.^63,64^ Thiazolones **2a–2c** have been previously described, although **2b** was prepared using a different method.^65,66^ Thiazolones **2e**, **2f**, **2g**, **2h**, and **2j** appear on Scifinder, but either no references are associated with their synthesis, or no details of their preparation and characterization can be found in the literature associated. Therefore, we present here the full synthesis and characterization of thiazolones **2d–2o**.

##### (*Z*)-4-(4-Fluorobenzylidene)-2-phenyl-5(4*H*)-thiazolone **2d**

The oxazolone **1d** (2.5 g, 9.3 mmol), thioacetic acid (2.0 mL, 28.4 mmol) and NEt_3_ (0.1 mL) were heated in an oil bath at 70 °C while stirred for 18 h. During this time the oxazolone dissolved, giving a dark solution, and after some minutes a deep-colored solid precipitated. After the reaction time the mixture was left to reach room temperature and ethanol (30 mL) was added to complete the precipitation. The yellow solid thus formed was filtered, washed thoroughly with ethanol (120–150 mL) until the characteristic smell of thioacetic acid disappeared, dried by suction, and characterized as **2d**. Obtained: 1.64 g (62% yield). ^1^H NMR (CD_2_Cl_2_, 300.13 MHz): δ = 8.30 (dd, 2H, H_2_/H_6_, C_6_H_4_F, ^3^*J*_HH_ = 8.7 Hz, ^4^*J*_HF_ = 5.7 Hz), 8.00 (d, 2H, H_o_, Ph, ^3^*J*_HH_ = 6.5 Hz), 7.61–7.50 (m, 3H, H_p_+2H_m,_Ph), 7.18 (t, 2H, H_3_/H_5_, ^3^*J*_HH_≈ ^3^*J*_HF_ = 8.7 Hz), 7.17 (s, 1H, H_vinyl_). ^19^F NMR (CD_2_Cl_2_, 282.4 MHz): δ = −107.41 (tt, ^3^*J*_FH_ = 8.7 Hz, ^4^*J*_FH_ = 3.1 Hz). ^13^C{^1^H} NMR (CD_2_Cl_2_, 75.5 MHz): δ = 194.7 (CO), 167.6 (NCS), 165.0 (d, C_4_F, ^1^*J*_CF_ = 254 Hz), 146.4 (=C), 136.0 (d, 2CH, C_2_/C_6_, ^3^*J*_FC_ = 8.7 Hz), 133.9 (C, C_i_, Ph), 133.3 (CH, C_p_, Ph), 130.8 (d, C, C_1_, C_6_H_4_F, ^4^*J*_FC_ = 3.3 Hz), 130.0 (d, CH, C_vinyl_, ^5^*J*_FC_ = 1.7 Hz), 129.6 (2CH, C_m_, Ph), 128.8 (2CH, C_o_, Ph), 116.7 (d, 2CH, C_3_/C_5_, C_6_H_4_F, ^2^*J*_FC_ = 22.0 Hz). HRMS (ESI^+^) [*m*/*z*]: calcd for [C_16_H_10_FNNaOS]^+^ = [M + Na]^+^, 306.0359; found, 306.0349. IR (ν, cm^–1^): 1682 (CO, vs).

##### (*Z*)-4-(4-Chlorobenzylidene)-2-phenyl-5(4*H*)-thiazolone **2e**

Thiazolone **2e** was obtained following the same experimental procedure than the described for **2d**. Therefore, oxazolone **1e** (2.5 g, 8.8 mmol) was reacted with thioacetic acid (2 mL) and NEt_3_ (0.1 mL) for 18 h at 70 °C to give **2e** as a deep yellow solid. Obtained: 1.55 g (59% yield). ^1^H NMR (CD_2_Cl_2_, 300.13 MHz): δ = 8.25 (AA′BB′ spin system, 2H, H_3_/H_5_, C_6_H_4_Cl,), 8.04 (dd, 2H, H_o_, Ph, ^3^*J*_HH_ = 8.2 Hz, ^4^*J*_HH_ = 1.5 Hz), 7.64–7.55 (m, 3H, H_p_+2H_m,_Ph), 7.48 (AA′BB′ spin system, 2H, H_2_/H_6_, C_6_H_4_Cl), 7.18 (s, 1H, H_vinyl_). ^13^C{^1^H} NMR (CD_2_Cl_2_, 75.5 MHz): δ = 194.1 (CO), 167.5 (NCS), 146.4 (=C), 137.3 (C, C_4_, C_6_H_4_Cl), 134.2 (2CH, C_3_/C_5_, C_6_H_4_Cl), 133.3 (C_1_, C_6_H_4_Cl), 132.9 (CH, C_p_, Ph), 132.4 (C_i_, Ph), 129.2 (CH, C_vinyl_), 129.2 (2CH, C_2_/C_6_, C_6_H_4_Cl), 129.0 (2CH, C_m_, Ph), 128.3 (2CH, C_o_, Ph). HRMS (ESI^+^) [*m*/*z*]: calcd for [C_16_H_10_ClNNaOS]^+^ = [M + Na]^+^, 322.0064; found, 322.0053.

##### (*Z*)-4-(4-Bromobenzylidene)-2-phenyl-5(4*H*)-thiazolone **2f**

Thiazolone **2f** was obtained following the same experimental procedure than the described for **2d**. Therefore, oxazolone **1f** (2.5 g, 7.6 mmol) was reacted with thioacetic acid (2 mL) and NEt_3_ (0.1 mL) for 18 h at 70 °C to give **2f** as a deep yellow solid. Obtained: 1.19 g (45% yield). ^1^H NMR (CDCl_3_, 300.13 MHz): δ = 8.13 (d, 2H, H_3_ + H_5_, C_6_H_4_Br, ^3^*J*_HH_ = 8.5 Hz), 8.01 (d, 2H, H_o_, Ph, ^3^*J*_HH_ = 6.7 Hz), 7.61 (d, 2H, H_2_ + H_6_, C_6_H_4_Br, ^3^*J*_HH_ = 8.5 Hz), 7.57–7.51 (m, 3H, H_p_ + 2H_m_, Ph), 7.16 (s, 1H, H_vinyl_). ^13^C{^1^H} NMR (CDCl_3_, 75.5 MHz): δ = 194.5 (CO), 167.7 (NCS), 146.7 (=C), 134.5 (2CH, C_3_ + C_5_, C_6_H_4_Br), 133.5 (C_1_, C_6_H_4_Br), 133.0 (CH, C_p_, Ph), 132.8 (C_i_, Ph), 132.4 (2CH, C_2_ + C_6_, C_6_H_4_Br), 129.8 (CH, C_vinyl_), 129.2 (2CH, C_m_, Ph), 128.5 (2CH, C_o_, Ph), 126.3 (C_4_, C-Br, C_6_H_4_Br). HRMS (ESI^+^) [*m*/*z*]: calcd for [C_16_H_10_BrNNaOS]^+^ = [M + Na]^+^, 365.9559; found, 365.9551.

##### (*Z*)-4-(4-Nitrobenzylidene)-2-phenyl-5(4*H*)-thiazolone **2g**

Thiazolone **2g** was obtained following the same experimental procedure than the described for **2d**. Therefore, oxazolone **1g** (2.5 g, 8.5 mmol) was reacted with thioacetic acid (2 mL) and NEt_3_ (0.1 mL) for 18 h at 70 °C to give **2g** as a deep yellow solid. Obtained: 1.02 g (38% yield). ^1^H NMR (CDCl_3_, 300.13 MHz): δ = 8.45 (AA′BB′ spin system, 2H, H_3_/H_5_, C_6_H_4_NO_2_), 8.35 (AA′BB′ spin system, 2H, H_2_/H_6_, C_6_H_4_NO_2_), 8.07 (d, 2H, H_o_, Ph, ^3^*J*_HH_ = 7.2 Hz), 7.66–7.57 (m, 3H, H_p_+2H_m,_Ph), 7.24 (s, 1H, H_vinyl_). ^13^C{^1^H} NMR (CDCl_3_, 75.5 MHz): δ = 195.3 (CO), 169.1 (NCS), 148.4 (C_4_, C_6_H_4_NO_2_), 139.7 (C_1_, C_6_H_4_NO_2_), 133.5 (CH, C_p_, Ph), 133.3 (2CH, C_3_/C_5_, C_6_H_4_NO_2_), 129.2 (2CH, C_m_, Ph), 128.6 (2CH, C_o_, Ph), 127.0 (CH, C_vinyl_), 123.9 (2CH, C_2_/C_6_, C_6_H_4_NO_2_). Due to low solubility some quaternary ^13^C signals (Ci of Ph and = C of the arylidene fragment) were missing. HRMS (ESI+) [*m*/*z*]: calcd for [C_16_H_10_N_2_NaO_3_S]^+^ = [M + Na]^+^, 333.0310; found, 333.0317. IR (ν, cm^–1^): 1696 (vs).

##### (*Z*)-4-(4-Trifluoromethylbenzylidene)-2-phenyl-5(4*H*)-thiazolone **2h**

Thiazolone **2h** was obtained following the same experimental procedure than the described for **2d**. Therefore, oxazolone **1h** (2.5 g, 7.9 mmol) was reacted with thioacetic acid (2 mL) and NEt_3_ (0.1 mL) for 18 h at 70 °C to give **2h** as a pale-yellow solid. Obtained: 1.84 g (70% yield). ^1^H NMR (CDCl_3_, 300.13 MHz): δ = 8.34 (AA′BB′ spin system, 2H, H_2_/H_6_, C_6_H_4_CF_3_), 8.01 (dd, 2H, H_o_, Ph, ^3^*J*_HH_ = 8.2 Hz, ^4^*J*_HH_ = 1.5 Hz), 7.72 (AA′BB′ spin system, 2H, H_3_/H_5_, C_6_H_4_CF_3_), 7.60–7.50 (m, 3H, H_p_+2H_m,_Ph), 7.19 (s, 1H, H_vinyl_). ^19^F NMR (CDCl_3_, 282.4 MHz): δ = −62.97 (s). ^13^C{^1^H} NMR (CDCl_3_, 75.5 MHz): δ = 194.4 (CO), 168.9 (NCS), 147.7 (=C), 137.1 (q, C_1_, C_6_H_4_CF_3,_^5^*J*_CF_ = 1.4 Hz), 133.3 (CH, C_p_, Ph), 133.2 (C_i_, Ph), 133.1 (2CH, C_2_/C_6_, C_6_H_4_CF_3_), 132.2 (q, C, C_4_, C_6_H_4_CF_3,_^2^*J*_CF_ = 43 Hz), 129.2 (2CH, C_m_, Ph), 128.7 (CH, C_vinyl_), 128.6 (2CH, C_o_, Ph), 125.8 (2CH, C_3_/C_5_, C_6_H_4_CF_3,_^3^*J*_CF_ = 3.8 Hz), 120.4 (q, CF_3_, ^1^*J*_CF_ = 272 Hz). Anal. Calcd for C_17_H_10_F_3_NOS: C, 61.26; H, 3.02; N, 4.20; S, 9.62. Found: C, 60.94; H, 3.12; N, 3.91; S, 9.93.

##### (*Z*)-4-(2-Methoxybenzylidene)-2-phenyl-5(4*H*)-thiazolone **2i**

Thiazolone **2i** was obtained following the same experimental procedure than the described for **2d**. Therefore, oxazolone **1i** (2.5 g, 8.9 mmol) was reacted with thioacetic acid (2 mL) and NEt_3_ (0.1 mL) for 18 h at 70 °C to give **2i** as a pale-yellow solid. Obtained: 0.80 g (30% yield). ^1^H NMR (CDCl_3_, 300.13 MHz): δ = 8.95 (d, 1H, H_6_, C_6_H_4_OMe, ^3^*J*_HH_ = 7.8 Hz), 8.02 (d, 2H, H_o_, Ph, ^3^*J*_HH_ = 6.5 Hz), 7.90 (s, 1H, H_vinyl_), 7.57–7.42 (m, 4H, H_p_(Ph) + 2H_m_(Ph) + H_4_/H_5_ (C_6_H_4_OMe)), 7.12 (t, 1H, H_4_/H_5_, C_6_H_4_OMe, ^3^*J*_HH_ = 7.6 Hz), 6.94 (d, 1H, H_3_, C_6_H_4_OMe, ^3^*J*_HH_ = 8.3 Hz), 3.92 (s, 3H, OMe). ^13^C{^1^H} NMR (CDCl_3_, 75.5 MHz): δ = 194.6 (CO), 165.9 (NSC), 160.1 (C_2_, C-OMe, C_6_H_4_OMe), 145.7 (=C), 133.6 (C_i_, Ph), 133.5 (CH, C_6_, C_6_H_4_OMe), 133.2 (CH, C_4_/C_5_, C_6_H_4_OMe), 132.4 (CH, C_p_, Ph), 128.9 (2CH, C_m_, Ph), 128.2 (2CH, C_o_, Ph), 125.4 (CH, C_vinyl_), 122.9 (C_1_, C_6_H_4_OMe), 121.0 (CH, C_4_/C_5_, C_6_H_4_OMe), 110.8 (CH, C_3_, C_6_H_4_OMe), 55.7 (OMe). HRMS (ESI^+^) [*m*/*z*]: calcd for [C_17_H_13_NNaO_2_S]^+^ = [M + Na]^+^, 318.0559; found, 318.0555.

##### (*Z*)-4-(2-Chlorobenzylidene)-2-phenyl-5(4*H*)-thiazolone **2j**

Thiazolone **2j** was obtained following the same experimental procedure than the described for **2d**. Therefore, oxazolone **1j** (2.5 g, 8.8 mmol) was reacted with thioacetic acid (2 mL) and NEt_3_ (0.1 mL) for 18 h at 70 °C to give **2j** as a yellow solid. Obtained: 0.98 g (36% yield). ^1^H NMR (CDCl_3_, 300.13 MHz): δ = 8.95 (dd, 1H, H_6_, C_6_H_4_Cl, ^3^*J*_HH_ = 7.8 Hz, ^4^*J*_HH_ = 2.0 Hz), 8.01 (m, 2H, H_o_, Ph), 7.75 (s, 1H, H_vinyl_), 7.62–7.34 (m, 6H, H_p_(Ph) + 2H_m_(Ph) + H_3_/H_4_/H_5_ (C_6_H_4_Cl)). ^13^C{^1^H} NMR (CDCl_3_, 75.5 MHz): δ = 194.2 (CO), 168.3 (NSC), 147.2 (=C), 137.5, 131.8 (C_i_/C_2_, C_6_H_4_Cl), 133.9 (CH, C_6_, C_6_H_4_Cl), 133.3 (C_i_, Ph), 133.0 (CH, C_p_, Ph), 131.7, 130.0, 127.1 (CH, C_3_/C_4_/C_5_, C_6_H_4_Cl), 129.0 (2CH, C_m_, Ph), 128.4 (2CH, C_o_, Ph), 126.0 (CH, C_vinyl_). HRMS (ESI^+^) [*m*/*z*]: calcd for [C_16_H_10_ClNNaOS]^+^ = [M + Na]^+^, 322.0064; found, 322.0072.

##### (*Z*)-4-(2-Bromobenzylidene)-2-phenyl-5(4*H*)-thiazolone **2k**

Thiazolone **2k** was obtained following the same experimental procedure than the described for **2d**. Therefore, oxazolone **1k** (2.5 g, 7.6 mmol) was reacted with thioacetic acid (2 mL) and NEt_3_ (0.1 mL) for 18 h at 70 °C to give **2k** as a deep yellow solid. Obtained: 1.02 g (39% yield). ^1^H NMR (CDCl_3_, 300.13 MHz): δ = 8.92 (dd, 1H, H_3_, C_6_H_4_Br, ^3^*J*_HH_ = 8.0 Hz, ^4^*J*_HH_ = 1.7 Hz), 8.01 (dd, 2H, H_o_, Ph, ^3^*J*_HH_ = 8.2 Hz, ^4^*J*_HH_ = 1.5 Hz), 7.70 (s, 1H, H_vinyl_), 7.68 (dd, 1H, H_6_, C_6_H_4_Br, ^3^*J*_HH_ = 8.0 Hz, ^4^*J*_HH_ = 1.2 Hz), 7.60–7.45 (m, 4H, H_p_ + 2H_m_ (Ph) + H_4_(C_6_H_4_Br)), 7.30 (td, 1H, H_5_, C_6_H_4_Br, ^3^*J*_HH_ = 8.0 Hz, ^4^*J*_HH_ = 1.7 Hz). ^13^C{^1^H} NMR (CDCl_3_, 75.5 MHz): δ = 194.1 (CO), 168.3 (NCS), 147.2 (=C), 134.0 (CH, C_3_, C_6_H_4_Br), 133.4 (CH, C_5_/C_6_, C_6_H_4_Br), 133.3 (C_i_, Ph), 133.0 (CH, C_p_, Ph), 131.9 (CH, C_5_/C_6_, C_6_H_4_Br), 129.0 (2CH, C_o_, Ph), 128.8 (CH, C_vinyl_), 128.4 (2CH, C_m_, Ph), 128.3 (2 overlapped carbons, C_1_+C_2_, C_6_H_4_Br), 127.7 (CH, C_4_, C_6_H_4_Br). HRMS (ESI^+^) [*m*/*z*]: calcd for [C_16_H_10_BrNNaOS]^+^ = [M + Na]^+^, 365.9559; found, 365.9548.

##### (*Z*)-4-(3,4-Dimethoxybenzylidene)-2-phenyl-5(4*H*)-thiazolone **2l**

Thiazolone **2l** was obtained following the same experimental procedure than the described for **2d**. Therefore, oxazolone **1l** (2.5 g, 8.1 mmol) was reacted with thioacetic acid (2 mL) and NEt_3_ (0.1 mL) for 18 h at 70 °C to give **2l** as a deep orange solid. Obtained: 1.59 g (60% yield). ^1^H NMR (CDCl_3_, 300.13 MHz): δ = 8.29 (s, 1H, H_2_, C_6_H_3_), 7.97 (d, 1H, H_o_, Ph, ^3^*J*_HH_ = 7.2 Hz), 7.60 (d, 1H, H_6_, C_6_H_3_, ^3^*J*_HH_ = 8.4 Hz), 7.55–7.48 (m, 3H, 2H_m_ + H_p_(Ph)), 7.20 (s, 1H, H_vinyl_), 6.94 (d, 1H, H_5_, C_6_H_3_, ^3^*J*_HH_ = 8.4 Hz), 4.02 (s, 3H, OMe), 3.97 (s, 3H, OMe). ^13^C{^1^H} NMR (CDCl_3_, 75.5 MHz): δ = 194.4 (CO), 165.1 (NCS), 152.3, 149.1 (2C, C_3_+C_4_, C_6_H_3_), 144.5 (=C), 133.6 (C_i_, Ph), 132.3 (CH, C_6_, C_6_H_3_), 131.7 (CH, C_vinyl_), 129.0 (2CH, C_m,_Ph), 128.9 (CH, C_p_, Ph), 127.8 (2CH, C_o_, Ph), 127.0 (C, C_1_, C_6_H_3_), 114.5 (CH, C_2_, C_6_H_3_), 110.9 (CH, C_5_, C_6_H_3_), 56.0 (OCH_3_), 55.8 (OCH_3_). HRMS (ESI^+^) [*m*/*z*]: calcd for [C_18_H_15_NNaO_3_S]^+^ = [M + Na]^+^, 348.0665; found, 348.0659. IR (ν, cm^–1^): 1704 (vs), 1677.

##### (*Z*)-4-(3,4-Dimethylbenzylidene)-2-phenyl-5(4*H*)-thiazolone **2m**

Thiazolone **2m** was obtained following the same experimental procedure than the described for **2d**. Therefore, oxazolone **1m** (2.5 g, 9.0 mmol) was reacted with thioacetic acid (2 mL) and NEt_3_ (0.1 mL) for 18 h at 70 °C to give **2m** as a yellow solid. Obtained: 1.18 g (44% yield). ^1^H NMR (CDCl_3_, 300.13 MHz): δ = 8.09 (d, 1H, H_6_, C_6_H_3_, ^3^*J*_HH_ = 8.0 Hz), 8.05–8.02 (m, 3H, H_5_ (C_6_H_3_)+ 2H_o_(Ph)), 7.59–7.52 (m, 3H, H_p_ + 2H_m_, Ph), 7.29 (s, 1H, H_2_, C_6_H_3_), 7.23 (s, 1H, H_vinyl_), 2.37 (s, 3H, Me, C_6_H_3_(Me)_2_), 2.36 (s, 3H, Me, C_6_H_3_(Me)_2_). ^13^C{^1^H} NMR (CDCl_3_, 75.5 MHz): δ = 194.7 (CO), 165.7 (NCS), 145.5 (=C), 141.2 (C, C_6_H_3_), 137.2 (C, C_6_H_3_), 134.6 (CH, C_5_, C_6_H_3_), 133.6 (C_i_, Ph), 132.4 (CH, C_vinyl_), 131.9 (CH, C_p_, Ph), 131.5 (C, C_6_H_3_), 131.0 (CH, C_6_, C_6_H_3_), 130.4 (CH, C_2_, C_6_H_3_), 128.9 (2CH, C_m_, Ph), 128.1 (2CH, C_o_, Ph), 20.2 (CH_3_), 19.9 (CH_3_). HRMS (ESI^+^) [*m*/*z*]: calcd for [C_18_H_15_NNaOS]^+^ = [M + Na]^+^, 316.0767; found, 316.0771. IR (ν, cm^–1^): 1687 (vs), 1652.

##### (*Z*)-4-(3,4-Dichlorobenzylidene)-2-phenyl-5(4*H*)-thiazolone **2n**

Thiazolone **2n** was obtained following the same experimental procedure than the described for **2d**. Therefore, oxazolone **1n** (2.5 g, 7.9 mmol) was reacted with thioacetic acid (2 mL) and NEt_3_ (0.1 mL) for 18 h at 70 °C to give **2n** as a yellow solid. Obtained: 1.74 g (66% yield). ^1^H NMR (CDCl_3_, 300.13 MHz): δ = 8.49 (d, 1H, H_2_, C_6_H_3_Cl_2_, ^4^*J*_HH_ = 1.9 Hz), 8.06–8.02 (m, 3H, H_5_/H_6_(C_6_H_3_Cl_2_) + 2H_o_(Ph)), 7.63–7.55 (m, 4H, H_5_/H_6_(C_6_H_3_Cl_2_) + 2H_m_ + H_p_ (Ph)), 7.12 (s, 1H, H_vinyl_). ^13^C{^1^H} NMR (CDCl_3_, 75.5 MHz): δ = 194.1 (CO), 168.5 (NCS), 147.1 (=C), 145.1 (C, C_6_H_3_Cl_2_), 135.3 (C, C_6_H_3_Cl_2_), 134.2 (CH, C_6_, C_6_H_3_Cl_2_), 133.7 (2C overlapped, C_i_ (Ph) + C (C_6_H_3_Cl_2_)), 133.1 (CH, C_5_, C_6_H_3_Cl_2_), 131.9 (CH, C_2_, C_6_H_3_Cl_2_), 130.8 (CH, C_p,_Ph), 129.1 (2CH, C_o,_Ph), 128.4 (2CH, C_m,_Ph), 127.8 (CH, C_vinyl_). HRMS (ESI^+^) [*m*/*z*]: calcd for [C_16_H_9_Cl_2_NNaOS]^+^ = [M + Na]^+^, 355.9665; found, 355.9664. IR (ν, cm^–1^): 1699 (vs), 1683.

##### (*Z*)-4-(3,4-Difluorobenzylidene)-2-phenyl-5(4*H*)-thiazolone **2o**

Thiazolone **2o** was obtained following the same experimental procedure than the described for **2d**. Therefore, oxazolone **1o** (2.5 g, 8.8 mmol) was reacted with thioacetic acid (2 mL) and NEt_3_ (0.1 mL) for 18 h at 70 °C to give **2o** as a yellow solid. Obtained: 0.98 g (37% yield). ^1^H NMR (CDCl_3_, 300.13 MHz): 8.43 (ddd, 1H, H_2_, C_6_H_3_F_2_, ^3^*J*_FH_ = 11.7 Hz, ^4^*J*_FH_ = 8.0 Hz, ^4^*J*_HH_ = 1.9 Hz), 8.04 (d, 2H, H_o_, Ph, ^3^*J*_HH_ = 6.7 Hz), 7.81 (m, 1H, H_5_, C_6_H_3_F_2_), 7.63–7.54 (m, 3H, H_p_ + 2H_m_, Ph), 7.28 (t, 1H, H_6_, C_6_H_3_F_2_, ^3^*J*_HH_ ≈ ^4^*J*_FH_ = 8.5 Hz), 7.15 (s, 1H, H_vinyl_). ^19^F NMR (CDCl_3_, 282.40 MHz): δ = −131.30 (dddd, 1F, F_4_, ^3^*J*_FF_ = 21.0 Hz, ^3^*J*_FH_ = 14.1 Hz, ^4^*J*_FH_ = 8.0 Hz, ^4^*J*_FH_ = 6.0 Hz), −135.95 (ddd, 1F, F_3_, ^3^*J*_FF_ = 21.0 Hz, ^3^*J*_FH_ = 14.1 Hz, ^4^*J*_FH_ = 6.0 Hz). ^13^C{^1^H} NMR (CDCl_3_, 75.5 MHz): δ = 194.2 (CO), 168.1 (NCS), 152.2 (dd, C, C_3_/C_4_, C_6_H_3_F_2_, ^1^*J*_CF_ = 272 Hz, ^2^*J*_CF_ = 13.3 Hz), 150.2 (dd, C, C_3_/C_4_, C_6_H_3_F_2_, ^1^*J*_CF_ = 272 Hz, ^2^*J*_CF_ = 13.3 Hz), 146.5 (=C), 133.2 (C, C_i_, Ph), 133.0 (CH, C_p_, Ph), 131.0 (dd, C, C_1_, C_6_H_3_F_2_, ^3^*J*_CF_ = 4.1 Hz), 130.2 (dd, CH, C_5_, C_6_H_3_F_2_, ^2^*J*_CF_ = 6.6 Hz, ^3^*J*_CF_ = 3.4 Hz), 129.1 (2CH, C_m_, Ph), 128.4 (2CH, C_o_, Ph), 128.3 (CH, C_vinyl_), 121.1 (d, CH, C_2_, C_6_H_3_F_2_, ^2^*J*_CF_ = 18.7 Hz), 117.7 (d, CH, C_6_, C_6_H_3_F_2_, ^2^*J*_CF_ = 17.8 Hz). HRMS (ESI^+^) [*m*/*z*]: calcd for [C_16_H_10_F_2_NOS]^+^ = [M + H]^+^, 302.0445; found, 302.0446.

#### General Procedure for the [2 + 2]-Photocycloaddition of 4-Arylidene-2-phenyl-5(4*H*)-thiazolones **2**: Synthesis of Cyclobutanes **3a**–**3o**

A solution of the 4-arylidene-2-phenyl-5(4*H*)-thiazolones **2a–2o** (∼1 mmol) in 10 mL of CH_2_Cl_2_ was irradiated for 24–72 h with the blue light (465 nm) provided by the PCB of 24 LEDs while stirred at room temperature. The progress of the reaction was followed by ^1^H NMR. After 72 h, the conversion of thiazolones **2a–2o** into cyclobutanes **3a–3o** was complete (exceptions are indicated). The solvent was then evaporated to dryness, and the yellow solid residue was characterized by NMR as the cyclobutanes **3a–3o**. In almost all cases, the ε-isomer appeared as the major isomer (>90% molar ratio) with minor amounts (<10%) of other isomers (exceptions are indicated). For that reason, only the ε-isomer is fully characterized.
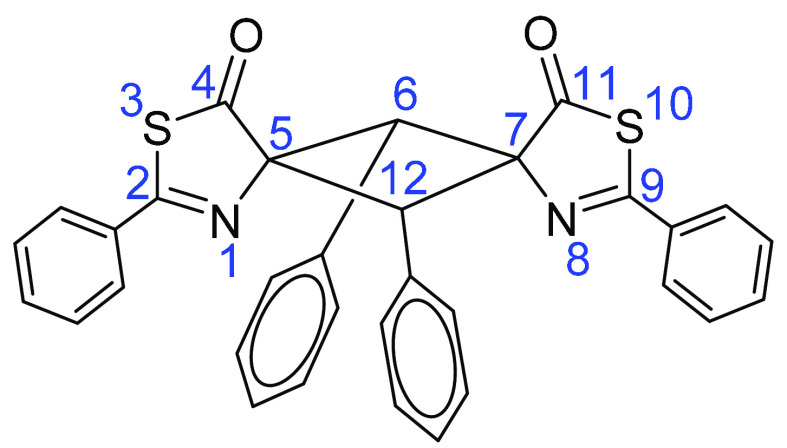


#### Numbering of Cyclobutanes **3a–3o**, Exemplified with **3a**

##### 2,6,9,12-Tetraphenyl-3,10-dithia-1,8-diazadispiro[4.1.4^7^.1^5^]-dodeca-1,8-diene-4,11-dione **3a**

Following the general method, thiazolone **2a** (300 mg, 1.13 mmol) was reacted in CH_2_Cl_2_ with blue light for 24 h to give **3a** as a yellow solid. Compound **3a** was recrystallized in a mixture CH_2_Cl_2_/*n*-pentane, the ε-isomer was obtained selectively. Obtained: 245.4 mg (82% yield). ^1^H NMR (CDCl_3_, 300.13 MHz): δ = 7.90 (m, 2H, H_o_, NCS-Ph), 7.54–7.48 (m, 5H, H_m_, H_p_, NCS-Ph; H_o_, Ph), 7.20 – 7.12 (m, 3H, H_m_, H_p_, Ph), 4.71 (s, 1H, CH_,_H-C(6,12)). ^13^C{^1^H} NMR (CDCl_3_, 75.5 MHz): δ = 208.0 (SC = O, C4,11), 164.9 (SC = N, C2,9), 133.6 (C_i_, Ph), 132.7 (C_i_, Ph), 132.3 (C_p_, CH, Ph), 131.1 (CH, Ph), 129.0 (CH, Ph), 128.5 (C_o_, CH, C2,9-Ph), 128.2 (C_p_, CH, Ph), 128.0 (CH, Ph), 91.3 (C_q_, C5,7), 58.5 (CH, C6,12). HRMS (ESI^+^) [*m*/*z*]: calcd for [C_32_H_23_N_2_O_2_S_2_]^+^=[M + H]^+^, 531.1195, found 531.1203.

##### 2,9-Diphenyl-6,12-di-*p*-tolyl-3,10-dithia-1,8-diazadispiro[4.1.4^7^.1^5^]-dodeca-1,8-diene-4,11-dione **3b**

Following the general method, thiazolone **2b** (300 mg, 1.07 mmol) was reacted in CH_2_Cl_2_ with blue light for 72 h to give **3b** as a yellow solid. Cyclobutane **3b** was obtained as a mixture of two isomers in 90:10 molar ratio. Obtained: 300 mg (100% yield). Only the major ε-isomer was fully characterized. ^1^H NMR (CD_2_Cl_2_, 300.13 MHz): δ = 7.98 (m, 2H, H_o_, Ph), 7.59 (m, 3H, H_m_, H_p_, Ph), 7.41 (AA′BB′ spin system, 2H, H_o_, C_6_H_4_), 7.02 (AA′BB′ spin system, 2H, H_m_, C_6_H_4_), 4.65 (s, 1H, H-C6,12), 2.21 (s, 3H, CH_3_). ^13^C{^1^H} NMR (CD_2_Cl_2_, 75.5 MHz): δ = 207.8 (SC = O, C4,11), 164.6 (SC = N, C2,9), 137.9 (C-CH_3_),133.3 (C_i_, Ph), 132.2 (C_p_, Ph), 130.6 (2C, C_m_, C_6_H_4_), 129.6 (C_i_, C_6_H_4_), 128.9 (2C, C_m_, Ph), 128.4 (2C, C_o_, C_6_H_4_), 128.3 (2C, C_o_, Ph), 91.2 (C_q_, C5,7), 58.1 (CH, C6,12), 20.7 (CH_3_). HRMS (ESI^+^) [*m*/*z*]: calcd for [C_34_H_26_N_2_NaO_2_S_2_]^+^ = [M + Na]^+^, 581.1333; found, 581.1329.

##### 6,12-Bis(4-methoxyphenyl)-2,9-diphenyl-3,10-dithia-1,8-diazadispiro-[4.1.4^7^.1^5^]dodeca-1,8-diene-4,11-dione **3c**

Following the general method, thiazolone **2c** (300 mg, 1.01 mmol) was reacted in CH_2_Cl_2_ with blue light for 72 h to give **3c** as a yellow solid. Cyclobutane **3c** was obtained as a mixture of four isomers in 59:25:9:7 molar ratio. Obtained: 300 mg (100% yield). Only the major ε-isomer was fully characterized. ^1^H NMR (CD_2_Cl_2_, 300.13 MHz): δ = 7.99 (m, 2H, H_o_, Ph), 7.60–7.58 (m, 3H, H_m_, H_p_, Ph), 7.52 (AA′BB′ spin system, 2H, H_o_, C_6_H_4_), 6.75 (AA′BB′ spin system, 2H, H_m_, C_6_H_4_), 4.64 (s, 1H, H-C6,12), 3.69 (s, 3H, -OCH_3_). ^13^C{^1^H} NMR (CD_2_Cl_2_, 75.5 MHz): δ = 207.8 (SC = O, C4,11), 164.6 (SC = N, C2,9), 159.4 (C-OCH_3_),133.4 (C_i_, Ph), 132.3 (2C, C_o_, C_6_H_4_), 131.9 (C_p_, Ph), 128.9 (2C, C_m_, Ph), 128.2 (2C, C_o_, Ph), 124.7 (C_i_, C_6_H_4_), 113.0 (2C, C_m_, C_6_H_4_), 91.7 (C_q_, C5,7), 57.9 (CH, C6,12), 55.0 (OCH_3_). HRMS (ESI^+^) [*m*/*z*]: calcd for [C_34_H_26_N_2_NaO_4_S_2_]^+^ = [M + Na]^+^, 613.1232; found, 613.1237.

##### 6,12-Bis(4-fluorophenyl)-2,9-diphenyl-3,10-dithia-1,8-diazadispiro-[4.1.4^7^.1^5^]dodeca-1,8-diene-4,11-dione **3d**

Following the general method, thiazolone **2d** (300 mg, 1.06 mmol) was reacted in CH_2_Cl_2_ with blue light for 72 h to give **3d** as a yellow solid. Cyclobutane **3d** was obtained as a mixture of two isomers in 91:9 molar ratio. Obtained: 298 mg (99% yield). Only the major ε-isomer was fully characterized. ^1^H NMR (CD_2_Cl_2_, 300.13 MHz): δ = 7.97 (m, 2H, H_o_, Ph), 7.63–7.55 (m, 2H, H_o_, C_6_H_4_ + 2H, H_m_, Ph + 1H, H_p_, Ph), 6.92 (t, 2H, H_m_, C_6_H_4_, ^3^*J*_HH_ = ^3^*J*_FH_ = 8.73 Hz), 4.69 (s, 1H, H-C6,12). ^13^C{^1^H} NMR (CD_2_Cl_2_, 75.5 MHz): δ = 207.3 (SC = O, C4,11), 165.5 (SC = N, C2,9), 162.5 (d, C-F, ^1^J_FC_ = 247 Hz), 133.2 (C_i_, C_6_H_4_), 132.9 (d, 2C, C_o_, C_6_H_4,_^3^J_FC_ = 8.3 Hz), 132.5 (C_p_, Ph), 129.0 (2C, C_m_, Ph), 128.4 (C_i_, Ph), 128.2 (2C, C_o_, Ph), 114.6 (d, 2C, C_m_, C_6_H_4,_^2^J_FC_ = 21.3 Hz), 90.9 (C_q_, C5,7), 57.4 (CH, C6,12). ^19^F NMR (CD_2_Cl_2_, 282.4 MHz) δ = −114.13 (tt, ^3^*J*_FH_ = 8.68 Hz, ^4^*J*_FH_ = 3.36 Hz). HRMS (ESI^+^) [*m*/*z*]: calcd for [C_32_H_20_F_2_N_2_NaO_2_S_2_]^+^ = [M + Na]^+^, 589.0826; found, 589.0812.

##### 6,12-Bis(4-chlorophenyl)-2,9-diphenyl-3,10-dithia-1,8-diazadispiro-[4.1.4^7^.1^5^]dodeca-1,8-diene-4,11-dione **3e**

Following the general method, thiazolone **2e** (300 mg, 1.00 mmol) was reacted in CH_2_Cl_2_ with blue light for 72 h to give **3e** as a yellow solid. Cyclobutane **3e** was obtained as a mixture of two isomers in a 96:4 molar ratio. Obtained: 255 mg (85% yield). Only the major ε-isomer was fully characterized. ^1^H NMR (CDCl_3_, 500.13 MHz): δ = 7.89 (m, 2H, H_o_, Ph), 7.59–7.52 (m, 3H, H_m_+H_p_, Ph), 7.47 (m, 2H, H_o_, C_6_H_4_), 7.14 (m, 2H, H_m_, C_6_H_4_), 4.63 (s, 1H, H-C6,12). ^13^C{^1^H} NMR (CDCl_3_, 125.76 MHz): δ = 207.3 (SC = O, C4,11), 165.6 (SC = N, C2,9), 134.3 (C-Cl, C_6_H_4_), 133.2 (C_i_, Ph), 132.5 (C_p_, Ph), 132.4 (2C, C_o_, C_6_H_4_), 130.7 (C_i_, C_6_H_4_), 129.0 (2C, C_m_, Ph), 128.3 (2C, C_o_, Ph), 128.1 (2C, C_m_, C_6_H_4_), 90.7 (C_q_, C5,7), 57.5 (CH, C6,12). HRMS (ESI^+^) [*m*/*z*]: calcd for [C_32_H_20_Cl_2_N_2_NaO_2_S_2_]^+^ = [M + Na]^+^, 621.0241; found, 621.0233.

##### 6,12-Bis(4-bromophenyl)-2,9-diphenyl-3,10-dithia-1,8-diazadispiro-[4.1.4^7^.1^5^]dodeca-1,8-diene-4,11-dione **3f**

Following the general method, thiazolone **2f** (300 mg, 0.87 mmol) was reacted in CH_2_Cl_2_ with blue light for 72 h to give **3f** as a yellow solid. Cyclobutane **3f** was obtained as a mixture of two isomers in 83:17 molar ratio. Obtained: 276 mg (92% yield). Only the major ε-isomer was fully characterized. ^1^H NMR (CDCl_3_, 300.13 MHz): δ = 7.89 (m, 2H, H_o_, Ph), 7.57–7.51 (m, 3H, H_m_ + H_p_, Ph), 7.41 (m, 2H, H_o_, C_6_H_4_), 7.30 (m, 2H, H_m_, C_6_H_4_), 4.63 (s, 1H, H-C6,12). ^13^C{^1^H} NMR (CDCl_3_, 75.5 MHz): δ = 207.3 (SC = O, C4,11), 165.8 (SC = N, C2,9), 133.3 (C_i_, Ph), 132.8 (2C, C_o_, C_6_H_4_), 132.6 (C_p_, Ph), 131.4 (C_i_, C_6_H_4_), 131.2 (2C, C_m_, C_6_H_4_), 129.2 (2C, C_m_, Ph), 128.4 (2C, C_o_, Ph), 122.8 (Br-C_p_, C_6_H_4_), 90.7 (C_q_, C5,7), 57.7 (CH, C6,12). Anal. Calcd for C_16_H_10_BrNOS: C, 55.83; H, 2.93; N, 4.07; S, 9.31. Found: C, 56.18; H, 2.63; N, 4.08; S, 9.67.

##### 6,12-Bis(4-nitrophenyl)-2,9-diphenyl-3,10-dithia-1,8-diazadispiro-[4.1.4^7^.1^5^]dodeca-1,8-diene-4,11-dione **3g**

Following the general method, thiazolone **2g** (300 mg, 0.94 mmol) was reacted in CH_2_Cl_2_ with blue light for 72 h to give **3g** as a yellow solid. Cyclobutane **3g** was obtained as a single isomer (ε-isomer). Obtained: 298 mg (100% yield). ^1^H NMR (CD_2_Cl_2_, 300.13 MHz): δ = 8.06 (m, 2H, H_m_, C_6_H_4_), 7.95 (m, 2H, H_o_, Ph), 7.77 (m, 2H, H_o_, C_6_H_4_), 7.65–7.56 (m, 3H, H_p_ + H_m_, Ph), 4.84 (s, 1H, H-C6,12). ^13^C{^1^H} NMR (CD_2_Cl_2_, 75.5 MHz): δ = 206.5 (SC = O, C4,11), 167.0 (SC = N, C2,9), 147.7 (C_6_H_4_, C-NO_2_), 139.2 (C_i_, C_6_H_4_), 132.9 (C_p_, Ph), 132.7 (C_i_, Ph), 131.9 (2C, C_o_, C_6_H_4_), 129.1 (2C, C_m_, Ph), 128.3 (2C, C_o_, Ph), 122.9 (2C, C_m_, C_6_H_4_), 89.7 (C_q_, C5,7), 57.1 (CH, C6,12). HRMS (ESI^+^) [*m*/*z*]: calcd for [C_32_H_20_N_4_NaO_6_S_2_]^+^ = [M + Na]^+^, 643.0716; found, 643.0708.

##### 6,12-Bis(4-trifluoromethylphenyl)-2,9-diphenyl-3,10-dithia-1,8-diazadispiro[4.1.4^7^.1^5^] dodeca-1,8-diene-4,11-dione **3h**

Following the general method, thiazolone **2h** (300 mg, 0.90 mmol) was reacted in CH_2_Cl_2_ with blue light for 72 h to give a maximal conversion of **3h** of 80% as a single isomer (ε-isomer). Further irradiation of the solution did not improved the conversion. Obtained: 241 mg. The remaining thiazolone **2h** proved to be difficult to be separated. ^1^H NMR (CDCl_3_, 500.13 MHz): δ = 7.89 (m, 2H, H_o_, Ph), 7.63 (m, 2H, H_o_, C_6_H_4_), 7.60–7.53 (m, 3H, H_p_ + H_m_, Ph), 7.44 (m, 2H, H_m_, C_6_H_4_), 4.75 (s, 1H, H-C6,12). ^13^C{^1^H} NMR (CDCl_3_, 125.7 MHz): δ = 207.1 (SC = O, C4,11), 166.3 (SC = N, C2,9), 136.2 (C_i_, C_6_H_4_), 133.1 (C_i_, Ph), 132.8 (C_p_, Ph), 131.4 (2C, C_o_, C_6_H_4_), 130.5 (q, C-CF_3_, ^2^*J*_CF_ = 66.6 Hz), 129.3 (2C, C_m_, Ph), 128.4 (2C, C_o_, Ph), 125.0 (q, 2C, C_m_, C_6_H_4_, ^3^*J*_CF_ = 3.7 Hz), 124.0 (q, CF_3_, ^1^*J*_CF_ = 272.2 Hz), 90.5 (C_q_, C5,7), 57.7 (CH, C6,12). ^19^F NMR (CDCl_3_, 282 MHz) δ = −62.72 (s, CF_3_). HRMS (ESI^+^) [*m*/*z*]: calcd for [C_34_H_20_F_6_N_2_NaO_2_S_2_]^+^ = [M + Na]^+^, 689.0752; found, 689.0768.

##### 6,12-Bis(2-chlorophenyl)-2,9-diphenyl-3,10-dithia-1,8-diazadispiro-[4.1.4^7^.1^5^]dodeca-1,8-diene-4,11-dione **3j**

Following the general method, thiazolone **2j** (300 mg, 1.00 mmol) was reacted in CH_2_Cl_2_ with blue light for 72 h to give **3j** as a yellow solid. Cyclobutane **3j** was obtained as the mixture of two isomers in 71:29 molar ratio. Obtained: 285 mg (95% yield). Only the major ε-isomer was fully characterized. ^1^H NMR (CDCl_3_, 500.13 MHz): δ = 8.19 (dd, 1H, H_6_, C_6_H_4_, ^3^*J*_HH_ = 7.0 Hz, ^4^*J*_HH_ = 2.3 Hz), 7.87 (m, 2H, H_o_, Ph), 7.58–7.46 (m, 3H, H_m_+H_p_, Ph, overlapped with minor isomer), 7.24 (dd, 1H, H_3_, C_6_H_4_, ^3^*J*_HH_ = 7.0 Hz, ^4^*J*_HH_ = 1.9 Hz), 7.06 (td, 2H, H_4_, H_5_, C_6_H_4_, ^3^*J*_HH_ = 7.0 Hz, ^4^*J*_HH_ = 1.9 Hz), 5.54 (s, 1H, H-C6,12). ^13^C{^1^H} NMR (CDCl_3_, 125.7 MHz): δ = 206.3 (SC = O, C4,11), 165.6 (SC = N, C2,9), 134.9 (C_1_, C_6_H_4_), 134.0 (C_6_, C_6_H_4_), 133.4 (C_i_, Ph), 132.3 (C_p_, Ph), 130.4 (C_2_, C_6_H_4_), 129.2, 125.8 (2C, C_4_, C_5_, C_6_H_4_), 129.1 (C_3_, C_6_H_4_), 128.9 (2C, C_m_, Ph), 128.2 (2C, C_o_, Ph), 90.4 (C_q_, C5,7), 53.0 (CH, C6,12). HRMS (ESI^+^) [*m*/*z*]: calcd for [C_32_H_20_Cl_2_N_2_NaO_2_S_2_]^+^ = [M + Na]^+^, 621.0241; found, 621.0238.

##### 6,12-Bis(2-bromophenyl)-2,9-diphenyl-3,10-dithia-1,8-diazadispiro-[4.1.4^7^.1^5^] dodeca-1,8-diene-4,11-dione **3k**

Following the general method, thiazolone **2k** (300 mg, 0.87 mmol) was reacted in CH_2_Cl_2_ with blue light for 72 h to give **3k** as a yellow solid. Cyclobutane **3k** was obtained as the mixture of two isomers in 65:35 molar ratio. Obtained: 295 mg (98% yield). In this case, well-separated peaks were observed for the two isomers, allowing their full characterization. Major isomer (ε-isomer) ^1^H NMR (CDCl_3_, 500.13 MHz): δ = 8.32 (dd, 1H, H_6_, C_6_H_4_, ^3^*J*_HH_ = 8.1 Hz, ^4^*J*_HH_ = 1.7 Hz), 7.87 (m, 2H, H_o_, Ph), 7.58–7.48 (m, 3H, H_p_+H_m_, Ph; overlapped with minor isomer), 7.43 (dd, 1H, H_3_, C_6_H_4_, ^3^*J*_HH_ = 7.9 Hz, ^4^*J*_HH_ = 1.2 Hz), 7.14 (td, 1H, H_5_, C_6_H_4_, ^3^*J*_HH_ = 7.8 Hz, ^4^*J*_HH_ = 1.3 Hz), 6.97 (td, 1H, H_4_, C_6_H_4_, ^3^*J*_HH_ = 7.8 Hz, ^4^*J*_HH_ = 1.7 Hz), 5.62 (s, 1H, H-C6,12). ^13^C{^1^H} NMR (CDCl_3_, 125.76 MHz): δ = 206.2 (SC = O, C4,11), 165.6 (SC = N, C2,9), 134.5 (C_6_, C_6_H_4_), 133.4 (C_i_, Ph),132.5 (C_3_, C_6_H_4_), 132.3 (C_p_, Ph), 132.1 (C_i_, C_6_H_4_), 129.5 (C_4_, C_6_H_4_), 128.9 (2C, C_m_, Ph; overlapped with minor isomer), 128.2 (2C, C_o_, Ph), 126.4 (C_5_, C_6_H_4_), 125.9 (Br-C_2_, C_6_H_4_), 90.5 (C_q_, C5,7), 55.4 (CH, C6,12). Minor isomer (α-isomer) ^1^H NMR (CDCl_3_, 500.13 MHz): δ = 8.62 (dd, H_6_, C_6_H_4_, ^3^*J*_HH_ = 8.0 Hz, ^4^*J*_HH_ = 2.2 Hz), 7.96 (m, H_o_, Ph), 7.58–7.48 (m, H_p_+H_m_, Ph; overlapped with major isomer), 7.46 (dd, 1H, H_3_, C_6_H_4_, ^3^*J*_HH_ = 8.0 Hz, ^4^*J*_HH_ = 1.1 Hz), 7.35 (td, 1H, H_5_, C_6_H_4_, ^3^*J*_HH_ = 7.7 Hz, ^4^*J*_HH_ = 1.3 Hz), 7.09 (td, 1H, H_4_, C_6_H_4_, ^3^*J*_HH_ = 7.8 Hz, ^4^*J*_HH_ = 1.7 Hz), 5.91 (s, 1H, H-C6,12). ^13^C{^1^H} NMR (CDCl_3_, 125.76 MHz): δ = 205.8 (SC = O, C4,11), 164.9 (SC = N, C2,9), 134.0 (C_6_, C_6_H_4_), 133.5 (C_i_, Ph),133.3 (C_i_, C_6_H_4_), 132.6 (C_3_, C_6_H_4_), 132.3 (C_p_, Ph), 129.5 (C_4_, C_6_H_4_), 128.9 (2C, C_m_, Ph, overlapped), 128.5 (2C, C_o_, Ph), 127.0 (C_5_, C_6_H_4_), 125.4 (Br-C_2_, C_6_H_4_), 88.7 (C_q_, C5,7), 54.9 (CH, C6,12). Anal. Calcd for C_16_H_10_BrNOS: C, 55.83; H, 2.93; N, 4.07; S, 9.31. Found: C, 55.79; H, 2.91; N, 4.05; S, 9.62.

##### 6,12-Bis(3,4-dimethylphenyl)-2,9-diphenyl-3,10-dithia-1,8-diazadispiro-[4.1.4^7^.1^5^] dodeca-1,8-diene-4,11-dione **3m**

Following the general method, thiazolone **2m** (300 mg, 1.02 mmol) was reacted in CH_2_Cl_2_ with blue light for 72 h to give **3m** as a yellow solid. Cyclobutane **3m** was obtained as the mixture of two isomers in 85:15 molar ratio. Obtained: 299 mg (100% yield). Only the major ε-isomer was fully characterized. ^1^H NMR (CDCl_3_, 500.13 MHz): δ = 7.93 (m, 2H, H_o_, Ph), 7.55–7.49 (m, 3H, H_p_+H_m_, Ph), 7.34–7.28 (m, 2H, H_2_ + H_6_, C_6_H_3_), 6.91 (d, 1H, H_5_, C_6_H_3_, ^3^*J*_HH_ = 7.83 Hz), 4.61 (s, 1H, H-C6,12), 2.11 (s, 3H, C_3_-Me), 2.09 (s, 3H, C_4_-Me). ^13^C{^1^H} NMR (CDCl_3_, 125.76 MHz): δ = 208.0 (SC = O, C4,11), 164.2 (SC = N, C2,9), 136.4 (C_1_, C_6_H_3_), 135.7 (C_4_, C_6_H_3_), 133.7 (C_i_, Ph), 132.5 (C_2_, C_6_H_3_), 132.0 (C_p_, Ph), 130.0 (C_3_, C_6_H_3_), 129.0 (C_5_, C_6_H_3_), 128.8 (2C, C_m_, Ph), 128.6 (C_6_, C_6_H_3_), 128.3 (2C, C_o_, Ph), 91.6 (C_q_, C5,7), 58.3 (CH, C6,12), 19.7 (C_3_-Me), 19.4 (C_4_-Me). HRMS (ESI^+^) [*m*/*z*]: calcd for [C_36_H_30_N_2_NaO_2_S_2_]^+^ = [M + Na]^+^, 609.1646; found, 609.1651.

##### 6,12-Bis(3,4-dichlorophenyl)-2,9-diphenyl-3,10-dithia-1,8-diazadispiro-[4.1.4^7^.1^5^]dodeca-1,8-diene-4,11-dione **3n**

Following the general method, thiazolone **2n** (300 mg, 0.90 mmol) was reacted in CH_2_Cl_2_ with blue light for 72 h to give **3n** as a yellow solid. Cyclobutane **3n** was obtained as the mixture of two isomers in 91:9 molar ratio. Obtained: 298 mg (100% yield). Only the major ε-isomer was fully characterized. ^1^H NMR (CD_2_Cl_2_, 300.13 MHz): δ = 7.99 (m, 2H, H_o_, Ph), 7.91 (d, 1H, H_2_, C_6_H_3,_^4^*J*_HH_ = 2.0 Hz), 7.66–7.56 (m, 3H, H_p_+H_m_, Ph), 7.42 (dd, 1H, H_6_, C_6_H_3_, ^3^*J*_HH_ = 8.4 Hz, ^4^*J*_HH_ = 2.1 Hz), 7.29 (d, 1H, H_5_, C_6_H_3,_^3^*J*_HH_ = 8.4 Hz), 4.62 (s, 1H, H-C6,12). ^13^C{^1^H} NMR (CD_2_Cl_2_, 75.5 MHz): δ = 206.7 (SC = O, C4,11), 166.6 (SC = N, C2,9), 133.6 (C_2_, C_6_H_3_), 132.8 (C_p_, Ph), 132.4 (C_4_, C_6_H_3_), 132.2 (2C, C_i_ (Ph)+C_1_(C_6_H_3_)), 131.6 (C_3_, C_6_H_3_), 130.6 (C_6_, C_6_H_3_), 129.7 (C_5_, C_6_H_3_), 129.1 (2C, C_m_, Ph), 128.3 (2C, C_o_, Ph), 89.9 (C_q_, C5,7), 56.7 (CH, C6,12). HRMS (ESI^+^) [*m*/*z*]: calcd for [C_32_H_18_Cl_4_N_2_NaO_2_S_2_]^+^ = [M + Na]^+^, 688.9461; found, 688.9466.

##### 6,12-Bis(3,4-difluorophenyl)-2,9-diphenyl-3,10-dithia-1,8-diazadispiro-[4.1.4^7^.1^5^] dodeca-1,8-diene-4,11-dione **3o**

Following the general method, thiazolone **2o** (300 mg, 1.00 mmol) was reacted in CH_2_Cl_2_ with blue light for 72 h to give **3o** as a yellow solid. Cyclobutane **3o** was obtained as the mixture of two isomers in 94:6 molar ratio. Obtained: 271 mg (90% yield). Only the major ε-isomer was fully characterized. ^1^H NMR (CDCl_3_, 500.13 MHz): δ = 7.91 (m, 2H, H_o_, Ph), 7.65 (td, 1H, H_6_, C_6_H_3_, ^3^*J*_HH_ = ^4^*J*_FH_ = 9.5 Hz, ^4^*J*_HH_ = 2.2 Hz), 7.61–7.53 (m, 3H, H_p_ + H_m_, Ph), 7.12 (dt, 1H, H_2_, C_6_H_3_, ^3^J_HF_ = 8.7 Hz, ^4^*J*_HH_ = 2.2 Hz), 6.94 (q, 1H, H_5_, C_6_H_3_, ^3^J_HF_ = ^4^J_HF_ = ^3^*J*_HH_ = 9.5 Hz), 4.59 (s, 1H, H-C6,12). ^13^C{^1^H} NMR (CDCl_3_, 125.76 MHz): δ = 206.9 (SC = O, C4,11), 166.3 (SC = N, C2,9), 150.4 (dd, C_4_-F, ^1^*J*_CF_ = 253.2 Hz, ^2^*J*_CF_ = 14.7 Hz), 149.5 (dd, C_3_-F, ^1^*J*_CF_ = 249.6 Hz, ^2^*J*_CF_ = 15.3 Hz), 132.9 (C_i_, Ph), 132.7 (C_p_, Ph), 129.2 (2C, C_m_, Ph), 128.9 (m, C_1_, C_6_H_3_), 128.3 (2C, C_o_, Ph), 127.1 (t, C_2_, C_6_H_3_, ^2^*J*_CF_ = ^3^*J*_CF_ = 5.3 Hz), 120.7 (t, C_6_, C_6_H_3_, ^2^*J*_CF_ = ^3^*J*_CF_ = 10.06 Hz), 116.6 (dd, C_5_, C_6_H_3_, ^2^J_FC_ = 14.13 Hz, ^3^*J*_CF_ = 3.64 Hz), 90.5 (C_q_, C5,7), 56.8 (CH, C6,12). ^19^F{^1^H} NMR (CDCl_3_, 282.4 MHz) δ = −137.37 (d, ^3^J_FF_ = 9.6 Hz), −137.33 (d, ^3^J_FF_ = 9.6 Hz). HRMS (ESI^+^) [*m*/*z*]: calcd for [C_32_H_18_F_4_N_2_NaO_2_S_2_]^+^ = [M + Na]^+^, 625.0644; found, 625.0640.

#### General Procedure for the [2 + 2]-Photocycloaddition of 4-Arylidene-2-phenyl-5(4*H*)-thiazolones **2** in the Presence of BF_3_: Synthesis of Cyclobutanes **4a**–**4e**

To a suspension of the thiazolones **2a–2e** (around 0.38 mmol) in dry deoxygenated methanol (3 mL) under an Ar atmosphere, BF_3_·OEt_2_ was added (200 μL, 1.621 mmol). The resulting suspension was irradiated for 24 h with the blue light provided by a Kessil lamp (PR160L, 40 W). The distance between the sample and the lamp is 5 cm, and the power of the lamp is fixed at 50% to avoid the overheating of the sample. After the reaction time, the solid in suspension is filtered, washed with MeOH, dried in vacuo, and characterized as cyclobutanes **4a–4e**.
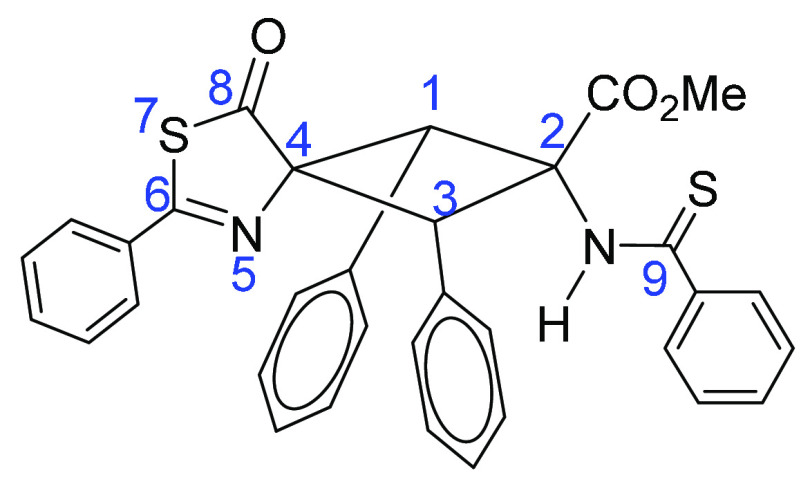


#### Numbering of Cyclobutanes **4a–4e**, Exemplified with **4a**

##### Methyl 8-Oxo-1,3,6-triphenyl-2-phenylthioamido-7-thia-5-azaspiro[3.4]-oct-5-ene-2-carboxylate **4a**

Following the general method, thiazolone **2a** (299.6 mg, 1.13 mmol) and BF_3_·OEt_2_ (600 μL, 4.863 mmol) were irradiated with blue light (456 nm) for 24 h in dry and deoxygenated methanol (9 mL) to give cyclobutane **4a** as a pale-yellow solid. Obtained: 185.1 mg (59% yield). ^1^H NMR (CDCl_3_, 300.13 MHz): δ = 8.66 (s, 1H, NH), 7.68 (d, 2H, H_o_, NCS-Ph), 7.65 (m, 2H, H_o_, NCS-Ph), 7.54 (t, 1H, H_p_, NCS-Ph), 7.50–7.42 (m, 3H, H_p_, H_m_, NCS-Ph), 7.42–7.33 (m, 6H, H_m_ NCS-Ph, H_o_ Ph), 7.24–7.15 (m, 6H, H_m_, H_p_, Ph), 4.94 (s, 2H, H-C1,3), 3.88 (s, 3H, OMe). ^13^C{^1^H} NMR (CDCl_3_, 75.5 MHz): δ = 206.3 (SC = O, C8), 199.0 (NC = S, C9), 169.7 (COO), 166.5 (SC = N, C6), 141.2 (C_i_, C6-Ph), 133.0 (C_i_, C9-Ph), 132.7 (C_p_, CH, C6-Ph), 132.5 (C_i_, C1,3-Ph), 131.4 (C_p_, CH, C9-Ph), 129.5 (C_o_, CH, C1,3-Ph), 129.2 (C_p_, CH, C1,3-Ph), 128.7 (C_m_, CH, C6-Ph), 128.7 (C_m_, CH, C1,3-Ph), 128.2 (C_m_, CH, C9-Ph), 128.2 (C_o_, CH, C6-Ph), 126.5 (C_o_, CH, C9-Ph), 90.8 (C_q_, C4), 67.8 (C_q_, C2), 55.0 (CH, C1,3), 53.1 (OMe). HRMS (ESI^+^) [*m*/*z*]: calcd for [C_33_H_27_N_2_O_3_S_2_]^+^ = [M + H]^+^, 563.1463; found, 563.1457.

##### Methyl 8-Oxo-6-phenyl-2-phenylthioamido-1,3-di-*p*-tolyl-7-thia-5-azaspiro[3.4]oct-5-ene-2-carboxylate **4b**

Following the general method, thiazolone **2b** (99.84 mg, 0.358 mmol) and BF_3_·OEt_2_ (200 μL, 1.621 mmol) were irradiated with blue light (456 nm) for 24 h in dry and deoxygenated methanol (3 mL) to give cyclobutane **4b** as a yellow solid. Compound **4b** was recrystallized from CH_2_Cl_2_/*n*-pentane. Obtained: 41.1 mg (39% yield). ^1^H NMR (CDCl_3_, 300.13 MHz): δ = 8.64 (s, 1H, NH), 7.72 (m, 2H, H_o_, NCS-Ph), 7.68 (m, 2H, H_o_, NCS-Ph), 7.55 (m, 1H, H_p_, NCS-Ph), 7.50–7.43 (m, 3H, H_p_, H_m,_ NCS-Ph), 7.37 (t, 2H, H_m_, NCS-Ph),7.27 (d, 4H, H_m_, C_6_H_4_Me, ^3^*J*_HH_ = 7.7 Hz), 7.00 (d, 4H, H_o_, C_6_H_4_Me, ^3^*J*_HH_ = 7.7 Hz), 4.87 (s, 2H, H-C1,3), 3.86 (s, 3H, OMe), 2.24 (s, 6H, Me). ^13^C{^1^H} NMR (CDCl_3_, 75.5 MHz): δ = 206.5 (SC = O, C8), 198.9 (NC = S, C9), 169.8 (COO), 166.1 (SC = N, C6), 141.4 (C_i_, C6/C9-Ph), 138.5 (C_i_, C1,3-C_6_H_4_Me), 138.0 (C_p_, C1,3-C_6_H_4_Me), 133.1 (C_i_, C6/C9-Ph), 132.6 (C_p_, CH, C6/C9-Ph), 131.3 (C_p_, CH, C6/C9-Ph), 129.4 (C_o,_C1,3-C_6_H_4_), 129.4 (C_m_, C1,3-C_6_H_4_), 129.2(C_m_, CH, C6/C9-Ph), 128.7 (C_m_, CH, C6/C9-Ph), 128.2 (C_o_, CH, C6/C9-Ph), 126.6 (C_o_, CH, C6/C9-Ph), 91.1 (C_q_, C4), 67.8 (C_q_, C2), 54.9 (CH, C1,3), 53.0 (OMe), 21.3 (Me). HRMS (ESI^+^) [*m*/*z*]: calcd for [C_35_H_30_N_2_O_3_S_2_Na]^+^ = [M + Na]^+^, 613.1590; found, 613.1588.

##### Methyl 1,3-Bis(4-fluorophenyl)-8-oxo-6-phenyl-2-phenylthioamido-7-thia-5-azaspiro[3.4]oct-5-ene-2-carboxylate **4d**

Following the general method, thiazolone **2d** (100.61 mg, 0.355 mmol) and BF_3_·OEt_2_ (200 μL, 1.621 mmol) in dry and deoxygenated methanol (3 mL) were irradiated with blue light (456 nm) for 24 h to give cyclobutane **4d** as a yellow solid. Compound **4d** was recrystallized from CH_2_Cl_2_/*n*-pentane. Obtained: 34.99 mg (33% yield). ^1^H NMR (CDCl_3_, 300.13 MHz): δ = 8.54 (s, 1H, NH), 7.67 (m, 2H, H_o_, NCS-Ph), 7.64 (m, 2H, H_o_, NCS-Ph), 7.57 (m, 1H, H_p_, NCS-Ph), 7.54–7.42 (m, 3H, H_p_, H_m,_ NCS-Ph), 7.39 (t, 2H, H_m_, NCS-Ph), 7.35 (m, 4H, H_o_, C_6_H_4_F), 6.90 (t, 4H, H_m_, C_6_H_4_F), 4.88 (s, 2H, H-C1,3), 3.88 (s, 3H, OMe). ^13^C{^1^H} NMR (CDCl_3_, 75.5 MHz): δ = 205.9 (SC = O, C8), 199.0 (NC = S, C9), 169.5 (COO), 167.2 (SC = N, C6), 162.7 (d, C-F, C1,3-C_6_H_4_F, ^1^J_FC_ = 248.21 Hz), 140.9 (C_i_, C6/C9-Ph), 133.0 (C_p_, CH, C6/C9-Ph), 132.7 (2C overlapped, C_i_, C6/C9-Ph + C_i_, C1,3-C_6_H_4_F), 131.7 (C_p_, CH, C6/C9-Ph), 131.2 (d, C_o_, C1,3-C_6_H_4_F, ^3^J_FC_ = 8.04 Hz), 129.4 (C_m_, CH, C6/C9-Ph), 128.8 (C_m_, CH, C6/C9-Ph), 128.1 (C_o_, CH, C6/C9-Ph), 126.4 (C_o_, CH, C6/C9-Ph), 115.7 (d, C_m_, C1,3-C_6_H_4_F, ^2^J_FC_ = 21.32 Hz), 90.6 (C_q_, C4), 67.7 (C_q_, C2), 54.2 (CH, C1,3), 53.2 (OMe). ^19^F NMR (CDCl_3_, 282.40 MHz) δ = −113.18 (tt, ^3^*J*_FH_ = 8.8 Hz, ^4^*J*_FH_ = 3.7 Hz). HRMS (ESI^+^) [*m*/*z*]: calcd for [C_33_H_25_F_2_N_2_O_3_S_2_]^+^ = [M + H]^+^, 599.1269; found, 599.1262.

##### Methyl 1,3-Bis(4-chlorophenyl)-8-oxo-6-phenyl-2-phenylthioamido-7-thia-5-azaspiro[3.4]oct-5-ene-2-carboxylate **4e**

Following the general method, thiazolone **2e** (100.37 mg, 0.336 mmol) and BF_3_·OEt_2_ (200 μL, 1.621 mmol) in dry and deoxygenated methanol (3 mL) were irradiated with blue light (456 nm; 100% intensity in this case to maximize the conversion) for 24 h to give cyclobutane **4e** as a yellow solid. Compound **4e** was recrystallized from CH_2_Cl_2_/*n*-pentane. Obtained: 32.27 mg (31% yield). ^1^H NMR (CDCl_3_, 300.13 MHz): δ = 8.53 (s, 1H, NH), 7.67 (m, 2H, H_o_, NCS-Ph), 7.64 (m, 2H, H_o_, NCS-Ph), 7.57 (m, 1H, H_p_, NCS-Ph), 7.53–7.44 (m, 3H, H_p_, H_m,_ NCS-Ph), 7.40 (t, 2H, H_m_, NCS-Ph), 7.29 (d, 4H, H_m_, C_6_H_4_Cl, ^2^*J*_HH_ = 8.5 Hz), 7.18 (d, 4H, H_o_, C_6_H_4_Cl, ^2^*J*_HH_ = 8.53 Hz), 4.86 (s, 2H, H-C1,3), 3.88 (s, 3H, OMe). ^13^C{^1^H} NMR (CDCl_3_, 75.5 MHz): δ = 205.8 (SC = O, C8), 198.1 (NC = S, C9), 169.4 (COO), 167.4 (SC = N, C6), 140.9 (C_i_, C6/C9-Ph), 134.4 (C_p_, C1,3-C_6_H_4_Cl), 133.1 (C_p_, CH, C6/C9-Ph), 131.7 (C_p_, CH, C6/C9-Ph), 132.6 (C_i_, C6/C9-Ph), 132.4 (C_i_, C1,3-C_6_H_4_Cl), 130.7 (C_o_, C1,3-C_6_H_4_Cl), 129.4 (C_m_, CH, C6/C9-Ph) 128.9 (C_m_, C1,3-C_6_H_4_Cl), 128.9 (C_m_, CH, C6/C9-Ph), 128.1 (C_o_, CH, C6/C9-Ph), 126.4 (C_o_, CH, C6/C9-Ph), 90.4 (C_q_, C4), 67.6 (C_q_, C2), 54.3 (CH, C1,3), 52.2 (OMe). HRMS (ESI^+^) [*m*/*z*]: calcd for [C_33_H_24_Cl_2_N_2_O_3_S_2_Na]^+^ = [M + Na]^+^, 653.0498; found, 653.0506.

#### Synthesis of Dihydrothiazoles (**5** and **6**) and Thiazoles (**7**) through a Ring-Opening Reaction Promoted by a Base

#### Reaction of Cyclobutanes **3c** and **3d** with NaOMe in MeOH

To a suspension of the cyclobutanes **3c** or **3d** (150 mg) in methanol (10 mL) was added NaOMe (10 mg). The resulting mixture was heated in an oil bath at 60 °C for 30 min. After the reaction time, the resulting solution was evaporated to dryness, and the residue was extracted with CH_2_Cl_2_ (2 × 15 mL). Any insoluble solid in the CH_2_Cl_2_ was removed by filtration. The organic phase was washed with H_2_O (10 mL), dried with anhydrous MgSO_4_, and evaporated to dryness, giving dihydrothiazoles **5c** or **5d** as yellow oils. Obtained: 168 mg (**5c**, 92% yield); 166 mg (**5d**, 94% yield). Compounds **5c** and **5d** were characterized by NMR methods as the mixture of the two possible diastereoisomers *trans* (*RR*/*SS*) and *cis* (*RS*/*SR*) in *trans*/*cis* = 86:14 (**5c**) and 81:19 (**5d**) molar ratios.

##### *trans*-(*RR*/*SS*) Methyl 5-(4-Methoxyphenyl)-2-phenyl-4,5-dihydrothiazole-4-carboxylate **5c**

^1^H NMR (CDCl_3_, 300.13 MHz): δ = 7.92 (d, 2H, H_o_, Ph, ^3^*J*_HH_ = 7 Hz), 7.52–7.46 (m, 3H, H_p_+H_m_, Ph), 7.35 (AA′BB′ spin system, 2H, H_o_, C_6_H_4_), 6.89 (AA′BB′ spin system, 2H, H_m_, C_6_H_4_), 5.45, 5.35 (AB spin system, 2H, H_4_+H_5_, ^3^*J*_HH_ = 6.6 Hz), 3.83 (s, 3H, CO_2_Me), 3.81 (s, 3H, OMe).). ^13^C{^1^H} NMR (CDCl_3_, 75.5 MHz): δ = 170.8 (CO_2_Me), 170.4 (SC = N), 159.5 (C_p_-OMe, C_6_H_4_), 132.7, 132.2 (2C, C_i_ (C_6_H_4_ + Ph)), 131.8 (C_p_, Ph), 128.7 (C_o_, C_6_H_4_), 128.6 (C_o_, Ph), 128.6 (C_m_, Ph), 114.3 (C_m_, C_6_H_4_), 86.6 (C4), 56.3 (C5), 55.3 (OMe), 52.8 (CO_2_Me). HRMS (ESI^+^) [*m*/*z*]: calcd for [C_18_H_18_NO_3_S]^+^ = [M + H]^+^, 328.1002; found, 328.1010.

##### *trans*-(*RR*/*SS*) Methyl 5-(4-Fluorophenyl)-2-phenyl-4,5-dihydrothiazole-4-carboxylate **5d**

^1^H NMR (CDCl_3_, 300.13 MHz): δ = 7.92 (dd, 2H, H_o_, Ph, ^3^*J*_HH_ = 6.8 Hz, ^4^*J*_HH_ = 1.2 Hz), 7.50–7.44 (m, 3H, H_p_+H_m_, Ph), 7.41 (m, 2H, H_o_, C_6_H_4_), 7.02 (dd, 2H, H_m_, C_6_H_4,_^3^*J*_HH_ = 8.7 Hz, ^4^*J*_FH_ = 2.4 Hz), 5.46, 5.33 (AB spin system, 2H, H_4_+H_5_, ^3^*J*_HH_ = 6.5 Hz), 3.81 (s, 3H, CO_2_Me). ^13^C{^1^H} NMR (CDCl_3_, 75.5 MHz): δ = 170.6 (CO_2_Me), 170.2 (SC = N), 162.4 (d, C_p_-F, C_6_H_4_, ^1^*J*_CF_ = 248.21 Hz), 136.1 (C_i_, C_6_H_4_), 132.5 (C_i_, Ph), 131.9 (C_p_, Ph), 129.2 (d, C_o_, C_6_H_4_, ^3^J_CF_=8.3 Hz), 128.7 (C_o_, Ph), 128.6 (C_m_, Ph), 115.9 (d, C_m_, C_6_H_4_, ^2^*J*_CF_ = 21.9 Hz), 86.7 (C4), 55.9 (C5), 52.9 (CO_2_Me). ^19^F NMR (CDCl_3_, 282 MHz) δ = −113.15 (tt, CF, ^3^*J*_FH_ = 8.09 Hz, ^4^*J*_FH_ = 4.86 Hz). HRMS (ESI^+^) [*m*/*z*]: calcd for [C_17_H_13_FNO_2_S]^+^ = [M – H]^+^, 314.0657; found, 314.0649.

#### General Procedure for the Synthesis of *trans*-(*RR*/*SS*) Ethyl 5-Aryl-2-phenyl-4,5-dihydrothiazole-4-carboxylates **6**

All syntheses of *trans*-(*RR*/*SS*) ethyl 5-aryl-2-phenyl-4,5-dihydrothiazole-4-carboxylates **6** were performed using the same experimental method, which is detailed here for the synthesis of **6c**. To a suspension of thiazolone **2c** (600 mg, 2.03 mmol) in 10 mL of ethanol was added NaOEt (10 mg, 0.09 mmol). The resulting mixture was refluxed (80 °C) in an oil bath for 2 h, then left to reach room temperature. The resulting solution was evaporated to dryness. The orange oily residue was extracted with CH_2_Cl_2_ (25 mL), removing all insoluble material by filtration. The clear solution was evaporated to dryness, and the residue was characterized by NMR as the dihydrotiazol **6c** (mixture of the two diastereoisomers), although impure. Therefore, *trans*-(*RR*/*SS*)-**6c** (the main component of the mixture) was purified by column chromatography using silica gel as support and a mixture of *n*-hexane/Et_2_O (8:1) as an eluent. The band collected under these conditions is *trans*-(*RR*/*SS*)-**6c**. Obtained: 411 mg (58% yield).

##### *trans*-(*RR*/*SS*) Ethyl 5-(4-Methoxyphenyl)-2-phenyl-4,5-dihydrothiazole-4-carboxylate **6c**

^1^H NMR (CDCl_3_, 300.13 MHz): δ = 7.92 (d, 2H, H_o_, Ph, ^3^*J*_HH_ = 6.9 Hz), 7.52–7.36 (m, 3H, H_p_ + H_m_, Ph), 7.34 (d, 2H, H_2_, H_6_, C_6_H_4_OMe, ^3^*J*_HH_ = 8.7 Hz), 6.89 (d, 2H, H_3_, H_5_, C_6_H_4_OMe, ^3^*J* = 8.7 Hz), 5.43 (AB spin system, 1H, SCH, ^3^*J*_HH_ = 6.7 Hz), 5.32 (AB spin system, 1H, NCH, ^3^*J*_HH_ = 6.7 Hz), 4.29 (q, 2H, OCH_2_CH_3_, ^3^*J*_HH_ = 7.1 Hz), 3.82 (s, 3H, OMe), 1.32 (t, 3H, OCH_2_CH_3_, ^3^*J*_HH_ = 7.1 Hz). ^13^C{^1^H} NMR (CDCl_3_, 75.5 MHz): δ = 170.3 (CO), 170.3 (NCS), 159.5 (C_4_, C_6_H_4_OMe), 132.8 (C_i_, Ph), 132.3 (C_1,_C_6_H_4_OMe), 131.7 (CH, C_p_, Ph), 128.7 (CH, C_o_, Ph), 128.7 (2CH, C_2_+C_6_, C_6_H_4_OMe), 128.6 (2CH, C_m_, Ph), 114.3 (2CH, C_3_+C_5_, C_6_H_4_OMe), 86.8 (CH, NC4H), 61.8 (CH_2_), 56.5 (CH, SC5H), 55.3 (OMe), 14.2 (CH_3_). HRMS (ESI^+^) [*m*/*z*]: calcd for [C_19_H_19_NNaO_3_S]^+^ = [M + Na]^+^, 364.0983; found, 364.0983.

##### *trans*-(*RR*/*SS*) Ethyl 5-(4-Trifluoromethylphenyl)-2-phenyl-4,5-dihydrothiazole-4-carboxylate **6h**

Following the general procedure, thiazolone **2h** (192 mg, 0.58 mmol) was reacted with NaOEt (10 mg, 0.09 mmol) for 2 h in refluxing EtOH (10 mL) to give, after chromatographic purification using silica gel as support and *n*-hexane/Et_2_O (8:1) as an eluent, *trans*-(*RR*/*SS*)-**6h** as a waxy orange solid. Obtained: 166.5 mg (76% yield). ^1^H NMR (CDCl_3_, 300.13 MHz): δ = 7.93 (d, 2H, H_o_, Ph, ^3^*J*_HH_ = 7.0 Hz), 7.65–7.46 (m, 7H, H_p_+H_m_, Ph; H_2,6_+H_3,5_, C_6_H_4_CF_3_), 5.49 (AB spin system, 1H, SCH, ^3^*J*_HH_ = 6.3 Hz), 5.36 (AB spin system, 1H, NCH, ^3^*J*_HH_ = 6.3 Hz), 4.31 (q, 2H, OCH_2_CH_3_, ^3^*J*_HH_ = 7.1 Hz), 3.87 (s, 3H, OMe), 1.33 (t, 3H, OCH_2_CH_3_, ^3^*J*_HH_ = 7.1 Hz). ^13^C{^1^H} NMR (CDCl_3_, 75.5 MHz): δ = 170.3 (NCS), 169.8 (CO), 144.4 (C_1_, C_6_H_4_CF_3_), 132.3 (C_i_, Ph), 132.1 (C_p_, Ph), 130.4 (q, C_4_-CF_3_, ^2^*J*_CF_ = 40 Hz), 128.8 (C_o_, Ph), 128.7 (C_m_, Ph), 128.0 (C_2_+C_6_, C_6_H_4_CF_3_), 126.0 (C_3_+C_5_, C_6_H_4_CF_3_, ^3^*J*_CF_ = 3.8 Hz), 123.5 (CF_3_, ^1^*J*_CF_ = 272 Hz), 86.4 (CH, NC4H), 62.1 (CH_2_), 55.9 (CH, SC5H), 14.1 (CH_3_). ^19^F NMR (CDCl_3_, 282.4 MHz): δ = −62.70 (s, CF_3_). HRMS (ESI^+^) [*m*/*z*]: calcd for [C_19_H_15_F_3_NO_2_S]^+^ = [M – H]^+^, 378.0781; found, 378.0797.

##### *trans*-(*RR*/*SS*) Ethyl 5-(2-Methoxyphenyl)-2-phenyl-4,5-dihydrothiazole-4-carboxylate **6i**

Following the general procedure, thiazolone **2i** (150 mg, 0.51 mmol) was reacted with NaOEt (10 mg, 0.09 mmol) for 2 h in refluxing EtOH (10 mL) to give, after chromatographic purification using silica gel as support and *n*-hexane/Et_2_O (11:1) as an eluent, *trans*-(*RR*/*SS*)-**6i** as a yellow oil. Obtained: 140 mg (80% yield). ^1^H NMR (CDCl_3_, 300.13 MHz): δ = 7.93 (dd, 2H, H_o_, Ph, ^3^*J*_HH_ = 8.2 Hz, ^4^*J*_HH_ = 1.4 Hz), 7.51 (tt, 1H, H_p_, Ph, ^3^*J*_HH_ = 8.2 Hz, ^4^*J*_HH_ = 1.4 Hz), 7.44 (t, 2H, H_m_, Ph, ^3^*J*_HH_ = 8.2 Hz), 7.39 (dd, 1H, H_6_, C_6_H_4_OMe, ^3^*J*_HH_ = 7.7 Hz, ^4^*J*_HH_ = 0.7 Hz), 7.29 (td, 1H, H_5_, C_6_H_4_OMe, ^3^*J*_HH_ = 7.7 Hz, ^4^*J*_HH_ = 0.7 Hz), 6.96 (td, 1H, H_4_, C_6_H_4_OMe, ^3^*J*_HH_ = 7.7 Hz, ^4^*J*_HH_ = 0.7 Hz), 6.92 (d, 1H, H_3_, C_6_H_4_OMe, ^4^*J*_HH_ = 0.7 Hz), 5.81 (AB spin system, 1H, NCH, ^3^*J*_HH_ = 5.1 Hz), 5.45 (AB spin system, 1H, SCH, ^3^*J*_HH_ = 5.1 Hz), 4.29 (q, 2H, OCH_2_CH_3_, ^3^*J*_HH_ = 7.1 Hz), 3.87 (s, 3H, OMe), 1.33 (t, 3H, OCH_2_CH_3_, ^3^*J*_HH_ = 7.1 Hz). ^13^C{^1^H} NMR (CDCl_3_, 75.5 MHz): δ = 170.9 (NCS), 170.4 (COO), 156.5 (C_2_-OMe, C_6_H_4_OMe), 132.9 (C_i_, Ph), 131.6 (C_p_, Ph), 129.2 (C_4_/C_5_, C_6_H_4_OMe), 128.8 (C_1_, C_6_H_4_OMe), 128.7 (C_o_, Ph), 128.5 (C_m_, Ph), 127.8 (C_6_, C_6_H_4_OMe), 121.0 (C_4_/C_5_, C_6_H_4_OMe), 110.7 (C_3_, C_6_H_4_OMe), 84.4 (CH, NCH), 61.7 (OCH_2_), 55.5 (CH_3_, OMe), 50.4 (CH, SCH), 14.2 (CH_3_). HRMS (ESI^+^) [*m*/*z*]: calcd for [C_19_H_19_NNaO_3_S]^+^ = [M + Na]^+^, 364.0983; found, 364.0978.

##### *trans*-(*RR*/*SS*) Ethyl 5-(3,4-Dimethylphenyl)-2-phenyl-4,5-dihydrothiazole-4-carboxylate **6m**

Following the general procedure, thiazolone **2m** (150 mg, 0.51 mmol) was reacted with NaOEt (10 mg, 0.09 mmol) for 4 h in refluxing EtOH (10 mL) to give, after chromatographic purification using silica gel as support and *n*-hexane/Et_2_O (8:1) as an eluent, *trans*-(*RR*/*SS*)-**6m** as a yellow oil. Obtained: 129 mg (75% yield). ^1^H NMR (CDCl_3_, 300.13 MHz): 7.94 (d, 2H, H_o_, Ph, ^3^*J*_HH_ = 6.8 Hz), 7.53–7.43 (m, 3H, H_p_ + 2H_m_, Ph), 7.19 (s, 1H, H_2_, C_6_H_3_(Me)_2_), 7.16, 7.12 (AB spin system, 2H, H_5_ + H_6_, ^3^*J*_HH_ = 7.5 Hz, C_6_H_3_(Me)_2_), 5.41 (AB spin system, CH, ^3^*J*_HH_ = 6.6 Hz), 5.36 (AB spin system, CH, ^3^*J*_HH_ = 6.6 Hz), 4.31 (q, 1H, OCH_2_CH_3_, ^3^*J*_HH_ = 7.1 Hz), 4.29 (q, 1H, OCH_2_CH_3_, ^3^*J*_HH_ = 7.1 Hz), 2.27 (s, 6H, 2CH_3_), 1.33 (t, 3H, OCH_2_CH_3_, ^3^*J*_HH_ = 7.1 Hz). ^13^C{^1^H} NMR (CDCl_3_, 75.5 MHz): 170.4 (NCS), 170.4 (COO), 137.8, 137.3, 136.7 (3C_q_, C_1_, C_3_, C_4_, C_6_H_3_(Me)_2_), 132.7 (C_i_, Ph), 131.8 (C_p_, Ph), 130.2 (CH, C_6_/C_5_, C_6_H_3_(Me)_2_), 128.7 (C_o_, Ph), 128.7 (CH, C_2_, C_6_H_3_(Me)_2_), 128.6 (C_m_, Ph), 124.9 (CH, C_6_/C_5_, C_6_H_3_(Me)_2_), 86.6 (NCH), 61.9 (OCH_2_), 56.5 (SCH), 19.8 (CH_3_), 19.5 (CH_3_), 14.2 (OCH_2_CH_3_). HRMS (ESI^+^) [*m*/*z*]: calcd for [C_20_H_22_NO_2_S]^+^ = [M + H]^+^, 340.1366; found, 340.1359.

##### *trans*-(*RR*/*SS*) Ethyl 5-(3,4-Dichlorophenyl)-2-phenyl-4,5-dihydrothiazole-4-carboxylate (**6n**) and Ethyl 5-(3,4-Dichlorophenyl)-2-phenylthiazole-4-carboxylate (**7n**)

Following the general procedure, thiazolone **2n** (270 mg, 0.81 mmol) was reacted with NaOEt (10 mg, 0.09 mmol) for 2 h in refluxing EtOH (10 mL) to give a waxy orange solid. This solid is composed of dihydrothiazole **6n** and thiazole **7n**. These compounds were separated and purified by column chromatography using silica gel as support. Using a mixture of *n*-hexane/Et_2_O (4:1) as an eluent, the thiazole **7n** eluted first. Evaporation of the solvent gave pure **7n** as white crystals. Obtained: 128 mg (42% yield). Further elution with the same mixture of solvents gave *trans*-(*RR*/*SS*)-**6n** as a yellow oil. Obtained: 70 mg (24% yield)

##### Ethyl 5-(3,4-Dichlorophenyl)-2-phenyl-4,5-dihydrothiazole-4-carboxylate (**6n**)

^1^H NMR (CDCl_3_, 300.13 MHz): δ = 7.93 (d, 2H, H_o_, Ph, ^3^*J*_HH_ = 7.0 Hz), 7.55–7.43 (m, 5H, H_p_+H_m_ (Ph) + H_5_ + H_6_(C_6_H_3_Cl_2_)), 7.28 (s, 1H, H_2_, C_6_H_3_Cl_2_), 5.39 (AB spin system, 1H, SCH, ^3^*J*_HH_ = 6.3 Hz), 5.31 (AB spin system, 1H, NCH, ^3^*J*_HH_ = 6.3 Hz), 4.31 (q, 1H, OCH_2_CH_3_, ^3^*J*_HH_ = 7.1 Hz), 4.29 (q, 1H, OCH_2_CH_3_, ^3^*J*_HH_ = 7.1 Hz), 1.34 (t, 3H, OCH_2_CH_3_, ^3^*J*_HH_ = 7.1 Hz). ^13^C{^1^H} NMR (CDCl_3_, 75.5 MHz): 173.6 (NCS), 169.9 (CO), 140.5 (C_1_, C_6_H_3_Cl_2_), 132.2 (CH, C_5_/C_6_, C_6_H_3_Cl_2_), 131.0 (CH, C_5_/C_6_, C_6_H_3_Cl_2_), 129.6 (C_p_, Ph), 128.8 (C_o_, Ph), 128.7 (C_m_, Ph), 126.6 (CH, C_2_, C_6_H_3_Cl_2_), 86.1 (NCH), 62.2 (OCH_2_), 55.4 (SCH), 14.1 (CH_3_). Signals due to C_3_ and C_4_ (C_6_H_3_Cl_2_) and to the C_ipso_ (Ph) were not observed. HRMS (ESI^+^) [*m*/*z*]: calcd for [C_18_H_16_Cl_2_NO_2_S]^+^ = [M + H]^+^, 380.0279; found, 380.0289.

##### Ethyl 5-(3,4-Dichlorophenyl)-2-phenylthiazole-4-carboxylate (**7n**)

^1^H NMR (CDCl_3_, 300.13 MHz): δ = 8.01 (m, 2H, H_o_, Ph), 7.67 (d, 1H, H_2_, C_6_H_3_Cl_2_, ^4^*J*_HH_ = 2 Hz), 7.54–7.48 (m, 4H, H_p_+H_m_ (Ph) + H_5_(C_6_H_3_Cl_2_)), 7.40 (dd, 1H, H_6_, C_6_H_3_Cl_2_, ^3^*J*_HH_ = 8.3 Hz, ^4^*J*_HH_ = 2.1 Hz), 4.35 (q, 2H, OCH_2_CH_3_, ^3^*J*_HH_ = 7.1 Hz), 1.31 (t, 3H, OCH_2_CH_3_, ^3^*J*_HH_ = 7.1 Hz). ^13^C{^1^H} NMR (CDCl_3_, 75.5 MHz): δ = 166.8 (NCS), 161.9 (CO), 142.6, 142.2 (S-C=C-N), 133.5 (C, C_6_H_3_Cl_2_), 132.5, 132.5 (2C, C-Cl, C_6_H_3_Cl_2_), 131.8 (CH, C_2_, C_6_H_3_Cl_2_), 130.9 (C_p_, Ph), 130.5 (C_i_, Ph), 130.1 (CH, C_5_, C_6_H_3_Cl_2_), 129.3 (CH, C_6_, C_6_H_3_Cl_2_), 129.0 (C_m_, Ph), 126.9 (C_o_, Ph), 61.5 (CH_2_), 14.1 (CH_3_). HRMS (ESI^+^) [*m*/*z*]: calcd for [C_18_H_13_Cl_2_NNaO_2_S]^+^ = [M + Na]^+^, 399.9942; found, 399.9945.

#### Microwave Synthesis of Methyl 5-(4-Nitrophenyl)-2-phenyl-4,5-thiazole-4-carboxylate **7g**

To a suspension of thiazolone **2g** (300 mg, 0.97 mmol) in methanol (5 mL) was added NaOMe (9 mg). The mixture was heated in a microwave oven (150 W, 70 °C) for 1 min. After the reaction time, the solvent was evaporated to dryness, and the solid residue was extracted with CH_2_Cl_2_ (10 mL). The resulting suspension was filtered through a Celite pad, and the Celite was washed with additional CH_2_Cl_2_ (20 mL). The clear solution was evaporated to dryness, and the crude was characterized by NMR and was shown to contain thiazole **7g**. Compound **7g** was purified by column chromatography (silica gel; *n*-hexane/Et_2_O = 5:1 as an eluent), giving pure **7g** as white crystals. Obtained: 64 mg (20% yield). ^1^H NMR (CDCl_3_, 300.13 MHz): δ = 8.33 (AB spin system, 2H, H_3_+H_5_, C_6_H_4_NO_2_), 8.02 (m, 2H, H_o_, Ph), 7.74 (AB spin system, 2H, H_2_+H_6_, C_6_H_4_NO_2_), 7.51 (m, 3H, H_p_+H_m_, Ph), 3.91 (s, 3H, OMe). ^13^C{^1^H} NMR (CDCl_3_, 75.5 MHz): 167.6 (NCS), 162.3 (CO), 148.1 (C, S-C = ), 143.1 (C_4_-N, C_6_H_4_NO_2_), 141.9 (C, N-C = ), 137.0 (C_1_, C_6_H_4_NO_2_), 132.3 (C_i_, Ph), 131.2 (C_p_, Ph), 131.0 (C_2_/C_6_, C_6_H_4_NO_2_), 129.2 (C_m_, Ph), 126.7 (C_o_, Ph), 123.4 (C_3_/C_5_, C_6_H_4_NO_2_), 52.6 (OCH_3_). HRMS (ESI^+^) [*m*/*z*]: calcd for [C_17_H_12_N_2_NaO_4_S]^+^ = [M + Na]^+^, 363.0415; found, 363.0410.

#### Synthesis of Dihydrothiazole Derivatives (*cis*/*trans*)-**5** through Ring-Opening Reaction without a Base in the Presence of BF_3_ (General Procedure)

All syntheses of (*cis*/*trans*)-methyl 5-aryl-2-phenyl-4,5-dihydrothiazole-4-carboxylates (*cis*/*trans*)-**5** were performed using the same experimental method, which is detailed here for the synthesis of **5a**. To a suspension of the thiazolone **2a** (100.7 mg, 0.38 mmol) in methanol (5 mL) was added BF_3_·OEt_2_ (200 μL, 1.621 mmol). The resulting mixture was heated in an oil bath at a reflux temperature with stirring for 18 h. After the reaction time, the solvent was evaporated to dryness, and the oily residue was dissolved in CH_2_Cl_2_ (5 mL). This solution was washed with H_2_O (3 × 2 mL), dried with anhydrous MgSO_4_, and evaporated to dryness, giving **5a** as the mixture of the two diastereoisomers *trans* (*RR*/*SS*) and *cis* (*RS*/*SR*) in a 1:1 molar ratio. Obtained: 83.04 mg (74% yield)

##### (*cis*/*trans*)-Methyl 2,5-Diphenyl-4,5-dihydrothiazole-4-carboxylate **5a**

^1^H NMR (CDCl_3_, 300.13 MHz): δ = 7.96 (m, H_o_, NCS-Ph), 7.90 (m, H_o_, NCS-Ph), 7.52–7.21 (m, H_m_+H_p_, NCS-Ph, H_o_+H_m_,+H_p_, Ph, both isomers), 5.55 (d, NCH, ^3^*J*_HH_ = 9.0 Hz, *cis*-isomer), 5.45 (d, SCH, ^3^*J*_HH_ = 6.5 Hz, *trans*-isomer), 5.37 (d, NCH, ^3^*J*_HH_ = 6.5 Hz, *trans*-isomer), 5.23 (d, SCH, ^3^*J*_HH_ = 9.0 Hz, *cis*-isomer), 3.79 (s, OMe, *trans*-isomer), 3.34 (s, OMe, *cis*-isomer). ^13^C{^1^H} NMR (CDCl_3_, 75.5 MHz): δ = 170.7 (SC = N), 170.7 (COO), 170.5 (SC = N), 169.2 (COO), 140.3, 138.1 (C_i_, Ph, both isomers), 132.5 (C_i_, NCS-Ph, overlapped), 132.0, 131.9 (2C_p_, NCS-Ph, both isomers), 129.0, 128.8, 128.7, 128.6, 128.6, 128.4, 127.9, 127.5 (C_o_, C_m_, NCS-Ph; C_o_, C_m_, Ph; both isomers), 128.4, 128.2 (C_p_, Ph both isomers), 86.5 (NCH, *trans*-isomer), 83.9 (NCH, *cis*-isomer), 56.6 (SCH, *trans*-isomer), 55.8 (SCH, *cis*-isomer), 52.9 (OMe, *trans*-isomer), 51.9 (OMe, *cis*-isomer). HRMS (ESI^+^) [*m*/*z*]: calcd for [C_17_H_16_NO_2_S]^+^ = [M + H]^+^, 298.0896; found, 298.0893.

##### (*cis*/*trans*)-Methyl 2-Phenyl-5-(*p*-tolyl)-4,5-dihydrothiazole-4-carboxylate 5**b**

Following the general procedure, thiazolone **2b** (101.4 mg, 0.363 mmol) was reacted with BF_3_·OEt_2_ (200 μL, 1.621 mmol) for 18 h in refluxing MeOH (5 mL) to give (*cis*/*trans*)-**5b** (1:1.1 molar ratio) as a yellow oil. Obtained: 80 mg (71% yield). ^1^H NMR (CDCl_3_, 300.13 MHz): δ = 7.98 (m, H_o_, NCS-Ph), 7.91 (m, H_o_, NCS-Ph), 7.55–7.41 (m, H_m_, H_p_, NCS-Ph, both isomers), 7.30 (d, H_o_, C_6_H_4_Me, ^3^*J*_HH_ = 8.1 Hz), 7.16 (d, H_o_, H_m_, C_6_H_4_Me, ^3^*J*_HH_ = 7.8 Hz), 7.06 (d, H_m_, C_6_H_4_Me, ^3^*J*_HH_ = 8.1 Hz), 5.55 (d, NCH, ^3^*J*_HH_ = 8.9 Hz, *cis*-isomer), 5.44 (d, SCH, ^3^*J*_HH_ = 6.6 Hz, *trans*-isomer), 5.36 (d, NCH, ^3^*J*_HH_ = 6.6 Hz, *trans*-isomer), 5.23 (d, SCH, ^3^*J*_HH_ = 8.9 Hz, *cis*-isomer), 3.82 (s, OMe, *trans*-isomer), 3.40 (s, OMe, *cis*-isomer), 2.34 (s, Me), 2.30 (s, Me). ^13^C{^1^H} NMR (CDCl_3_, 75.5 MHz): δ = 170.9 (SC = N), 170.8 (SC = N), 170.6 (COO), 169.5 (COO), 138.3 (C_p_, C_6_H_4_Me), 138.2 (C_p_, C_6_H_4_Me), 137.4 (C_i_, C_6_H_4_Me), 135.2 (C_i_, C_6_H_4_Me), 132.7 (C_i_, NCS-Ph, both isomers, overlapped), 132.0, 131.9 (2C_p_, NCS-Ph, both isomers), 129.8, 129.2, 128.9, 128.8, 128.7 (2C overlapped), 127.8, 127.5 (C_o_, C_m_, NCS-Ph + C_o_, C_m_, C_6_H_4_Me), 86.6 (NCH, *trans*-isomer), 84.0 (NCH, *cis*-isomer), 56.6 (SCH, *trans*-isomer), 55.8 (SCH, *cis*-isomer), 53.0 (OMe, *trans*-isomer), 52.0 (OMe, *cis*-isomer), 21.3 (Me), 21.2 (Me). HRMS (ESI^+^) [*m*/*z*]: calcd for [C_18_H_17_NO_2_SNa]^+^ = [M + Na]^+^, 334.0872; found, 334.0877.

##### (*cis*/*trans*)-Methyl 2-Phenyl-5-(4-methoxyphenyl)-4,5-dihydrothiazole-4-carboxylate **5c**

Following the general procedure, thiazolone **2c** (100.3 mg, 0.34 mmol) was reacted with BF_3_·OEt_2_ (200 μL, 1.621 mmol) for 18 h in refluxing MeOH (5 mL) to give (*cis*/*trans*)-**5c** (1:1.1 molar ratio) as a yellow oil. In this case, further chromatographic purification was necessary to separate **5c** from starting thiazolone **2c**. The chromatographic purification was started using silica as support and *n*-hexane/Et_2_O (9:1) as an eluent. Using these conditions, only the thiazolone **2c** was eluted. Then the solvent was changed, and 2-propanol was employed. Using these conditions, the dihydrothiazole **5c** was obtained as a yellow oil after solvent evaporation. Obtained: 42.7 mg (38% yield). ^1^H NMR (CDCl_3_, 300.13 MHz): δ = 7.96 (m, H_o_, NCS-Ph), 7.90 (m, H_o_, NCS-Ph), 7.54–7.41 (m, H_m_, H_p_, NCS-Ph, both isomers), 7.32 (d, H_o_, C_6_H_4_OMe, ^3^*J*_HH_ = 8.1 Hz), 7.19 (d, H_o_, C_6_H_4_OMe, ^3^*J*_HH_ = 8.1 Hz), 6.87–6.78 (d, H_m_, C_6_H_4_OMe, ^3^*J*_HH_ = 8.0 Hz), 5.53 (d, NCH, ^3^*J*_HH_ = 8.9 Hz, *cis*-isomer), 5.43 (d, SCH, ^3^*J*_HH_ = 6.5 Hz, *trans*-isomer), 5.34 (d, NCH, ^3^*J*_HH_ = 6.6 Hz, *trans*-isomer), 5.23 (d, SCH, ^3^*J*_HH_ = 8.9 Hz, *cis*-isomer), 3.81 (s, OMe, *trans*-isomer), 3.79, 3.76 (2s, OMe, both isomers), 3.41 (s, OMe, *cis*-isomer). ^13^C{^1^H} NMR (CDCl_3_, 75.5 MHz): δ = 171.0 (SC = N), 170.9 (SC = N), 170.8 (COO), 169.4 (COO), 159.5 (C_p_, C_6_H_4_OMe), 159.5 (C_p_, C_6_H_4_OMe), 132.6 (C_i_, NCS-Ph, both isomers), 132.2 (C_i_, C_6_H_4_OMe), 132.0 (C_p_, NCS-Ph), 131.9 (C_p_, NCS-Ph), 130.0 (C_i_, C_6_H_4_OMe), 129.1 (C_o_, C_6_H_4_OMe), 128.8 (C_o_, C_6_H_4_OMe), 128.7, 128.6 (C_o_, C_m_, NCS-Ph, both isomers), 114.4 (C_m_, C_6_H_4_OMe), 113.7 (C_m_, C_6_H_4_OMe), 86.3 (NCH, *trans*-isomer), 83.8 (NCH, *cis*-isomer), 56.2 (SCH, *trans*-isomer), 55.4 (SCH, *cis*-isomer), 55.3 (OMe), 55.2 (OMe), 52.9 (COOMe).), 52.0 (COOMe). HRMS (ESI^+^) [*m*/*z*]: calcd for [C_18_H_17_NO_3_SNa]^+^ = [M + Na]^+^, 350.0821; found, 350.0823.

##### (*cis*/*trans*)-Methyl 5-(4-Fluorophenyl)-2-phenyl-4,5-dihydrothiazole-4-carboxylate **5d**

Following the general procedure, thiazolone **2d** (102.0 mg, 0.360 mmol) was reacted with BF_3_·OEt_2_ (200 μL, 1.621 mmol) for 18 h in refluxing MeOH (5 mL) to give (*cis*/*trans*)-**5d** (1:1.1 molar ratio) as a yellow oil. In this case, further chromatographic purification was necessary to separate **5d** from starting thiazolone **2d**. The chromatographic purification was started using silica as support and *n*-hexane/Et_2_O (9:1) as an eluent. Using these conditions, only the thiazolone **2d** was eluted. Then the solvent was changed, and 2-propanol was employed. Using these conditions, the dihydrothiazole **5d** was obtained as a yellow oil after solvent evaporation. Obtained: 81 mg (71% yield). ^1^H NMR (CDCl_3_, 300.13 MHz): δ = 7.99 (m, H_o_, NCS-Ph), 7.93 (m, H_o_, NCS-Ph), 7.55–7.43 (m, H_p_+H_m_, NCS-Ph, both isomers), 7.40 (m, H_o_, C_6_H_4_F), 7.28 (m, H_o_, C_6_H_4_F), 7.06 (tt, H_m_, C_6_H_4_F, ^3^J_HF_ = 8.6 Hz, ^4^*J*_HH_ = 2.1 Hz), 6.97 (tt, H_m_, C_6_H_4_F, ^3^J_HF_ = 8.7 Hz, ^4^*J*_HH_ = 2.0 Hz), 5.57 (d, NCH, ^3^*J*_HH_ = 8.9 Hz, *cis*-isomer), 5.47 (d, SCH, ^3^*J*_HH_ = 6.5 Hz, *trans*-isomer), 5.35 (d, NCH, ^3^*J*_HH_ = 6.5 Hz, *trans*-isomer), 5.26 (d, SCH, ^3^*J*_HH_ = 8.9 Hz, *cis*-isomer), 3.85 (s, OMe, *trans*-isomer). 3.43 (s, OMe, *cis*-isomer). ^13^C{^1^H} NMR (CDCl_3_, 75.5 MHz): δ = 170.7 (COO), 170.5 (SC = N), 170.4 (SC = N), 169.3 (COO), 162.6 (d, C_p_-F, C_6_H_4_F, ^1^*J*_CF_ = 247.4 Hz), 162.6 (d, C_p_-F, C_6_H_4_F, ^1^*J*_CF_ = 247.7 Hz), 136.2 (d, C_i_, C_6_H_4_F, ^4^*J*_CF_ = 3.3 Hz), 134.1 (d, C_i_, C_6_H_4_F, ^4^*J*_CF_ = 3.5 Hz), 132.6 (2C_i_, NCS-Ph, both isomers ovelapped), 132.1, 132.0 (2C_p_, NCS-Ph, both isomers), 129.8(d, C_o_, C_6_H_4_F, ^3^*J*_CF_ = 8.3 Hz), 129.3(d, C_o_, C_6_H_4_F, ^3^*J*_CF_ = 8.3 Hz), 128.9, 128.8, 128.7, 128.7 (C_o_, C_m_, NCS-Ph, both isomers), 116.0 (d, C_m_, C_6_H_4_F, ^2^*J*_CF_ = 21.7 Hz), 115.4 (d, C_m_, C_6_H_4_F, ^2^*J*_CF_ = 21.7 Hz), 86.8 (NCH, *trans*-isomer), 84.1 (NCH, *cis*-isomer), 56.0 (SCH, *trans*-isomer), 55.2 (SCH, *cis*-isomer), 53.0 (OMe). 52.0 (OMe). ^19^F NMR(CDCl_3_, 282.40 MHz): δ = −113.67 (tt, ^3^*J*_FH_ = 8.6 Hz, ^4^*J*_FH_ = 3.3 Hz), −113.23 (tt, ^3^*J*_FH_ = 8.5 Hz, ^4^*J*_FH_ = 3.3 Hz). HRMS (ESI^+^) [*m*/*z*]: calcd for [C_17_H_15_FNO_2_S]^+^ = [M + H]^+^, 316.0802; found, 316.0796.

##### (*cis*/*trans*)-Methyl 5-(4-Chlorophenyl)-2-phenyl-4,5-dihydrothiazole-4-carboxylate **5e**

Following the general procedure, thiazolone **2e** (300.6 mg, 1.00 mmol) was reacted with BF_3_·OEt_2_ (600 μL, 4.863 mmol) for 18 h in refluxing MeOH (15 mL) to give (*cis*/*trans*)-**5e** (1:1.7 molar ratio) as a yellow oil. In this case, chromatographic purification was carried out to separate (*cis*/*trans*)-**5e** from thiazolone **2e** and to further separate *cis*-**5e** from *trans*-**5e**. The chromatographic purification was started using silica as support and *n*-hexane/Et_2_O (9:1) as an eluent. Using these conditions, only the thiazolone **2e** was eluted. Then the solvent was changed, and a mixture, *n*-hexane/ethyl acetate (8:2), was used as an eluent. Compound *trans*-**5e** eluted first and was obtained as a yellow oil after solvent evaporation (obtained: 90 mg, 27% yield). Compound *cis*-**5e** eluted in a second fraction and was obtained as a yellow oil after solvent evaporation (obtained: 54 mg, 16% yield). ^1^H NMR (CDCl_3_, 300.13 MHz): δ = 7.95 (m, H_o_, NCS-Ph), 7.89 (m, H_o_, NCS-Ph), 7.55–7.40 (m, H_m_, H_p_, NCS-Ph both isomers), 7.33 (d, H_o_, H_m,_ C_6_H_4_Cl), 7.22 (d, H_o_, H_m_, C_6_H_4_Cl), 5.56 (d, NCH, ^3^*J*_HH_ = 8.9 Hz, *cis*-isomer), 5.42 (d, SCH, ^3^*J*_HH_ = 6.4 Hz, *trans*-isomer), 5.32 (d, NCH, ^3^*J*_HH_ = 6.4 Hz, *trans*-isomer), 5.21 (d, SCH, ^3^*J*_HH_ = 8.9 Hz, *cis*-isomer), 3.82 (s, OMe, *trans*-isomer), 3.42 (s, OMe, *cis*-isomer). ^13^C{^1^H} NMR (CDCl_3_, 75.5 MHz): δ = 170.7 (SC = N), 170.7 (SC = N), 170.6 (COO), 169.3 (COO), 138.2 (C_i_, C_6_H_4_Cl), 136.9 (C_i_, C_6_H_4_Cl), 134.4 (C_p_, C_6_H_4_Cl), 134.2 (C_p_, C_6_H_4_Cl), 132.5 (C_i_, NCS-Ph, both isomers), 132.2(C_p_, NCS-Ph), 132.1 (C_p_, NCS-Ph), 129.4, 128.9 (C_o_, C_m_ C_6_H_4_Cl), 129.3, 129.0 (C_o_, C_m_ C_6_H_4_Cl), 128.8, 128.8 (C_o_, C_m_, NCS-Ph), 128.8, 128.8 (C_o_, C_m_, NCS-Ph), 86.6 (NCH, *trans*-isomer), 83.9 (NCH, *cis*-isomer), 56.0 (SCH, *trans*-isomer), 55.2 (SCH, *cis*-isomer), 53.1 (OMe, *trans*-isomer), 52.2 (OMe, *cis*-isomer). HRMS (ESI^+^) [*m*/*z*]: calcd for [C_17_H_14_ClNO_2_SNa]^+^ = [M + Na]^+^, 354.0326; found, 354.0325.

##### (*cis*/*trans*)-Methyl 5-(4-Trifluoromethylphenyl)-2-phenyl-4,5-dihydrothiazole-4-carboxylate **5h**

Following the general procedure, thiazolone **2h** (103.60 mg, 0.311 mmol) was reacted with BF_3_·OEt_2_ (200 μL, 1.621 mmol) for 18 h in refluxing MeOH (5 mL) to give (*cis*/*trans*)-**5h** (1:2.1 molar ratio) as a yellow oil. In this case, further chromatographic purification was necessary to separate **5h** from starting thiazolone **2h**. The chromatographic purification was started using silica as support and *n*-hexane/Et_2_O (9:1) as an eluant. Using these conditions, only the thiazolone **2h** was eluted. Then the solvent was changed, and 2-propanol was employed. Using these conditions, the dihydrothiazole **5h** was obtained as a yellow oil after solvent evaporation. Obtained: 38 mg (34% yield). ^1^H NMR (CDCl_3_, 300.13 MHz): δ = 7.95 (m, H_o_, NCS-Ph), 7.91 (m, H_o_, NCS-Ph), 7.61 (d, H_m_, C_6_H_4_-CF_3,_^3^*J*_HH_ = 8.1 Hz, both isomers), 7.54–7.50 (m, H_p_, NCS-Ph both isomers, H_o_, C_6_H_4_-CF_3_), 7.40–7.42 (m, H_m_, NCS-Ph, both isomers), 7.39 (d, 2H, H_o_, C_6_H_4_-CF_3,_^3^*J*_HH_ = 8.2 Hz), 5.60 (d, NCH, ^3^*J*_HH_ = 8.9 Hz, *cis*-isomer), 5.49 (d, SCH, ^3^*J*_HH_ = 6.4 Hz, *trans*-isomer), 5.35 (d, NCH, ^3^*J*_HH_ = 6.4 Hz, *trans*-isomer), 5.28 (d, SCH, ^3^*J*_HH_ = 8.9 Hz, *cis*-isomer), 3.83 (s, OMe, *trans*-isomer), 3.39 (s, OMe, *cis*-isomer). ^13^C{^1^H} NMR (CDCl_3_, 75.5 MHz): δ = 173.4 (SC = N), 172.4 (SC = N), 170.1 (COO), 168.5 (COO), 144.0 (C_i_, C_6_H_4_-CF_3_), 141.5 (C_i_, C_6_H_4_-CF_3_), 133.2 (C_p_, NCS-Ph), 132.9 (C_p_, NCS-Ph), 131.5 (C_i_, NCS-Ph), 131.3 (C_i_, NCS-Ph), 130.9 (q, C_p_-CF_3_, ^2^*J*_CF_ = 32.9 Hz), 130.8 (q, C_p_-CF_3_, ^2^*J*_CF_ = 32.9 Hz), 129.1, 129.1 (C_o_, C_m_, NCS-Ph), 129.0, 129.0 (C_o_, C_m_, NCS-Ph), 128.6 (C_o_, C_6_H_4_-CF_3_), 128.1 (C_o_, C_6_H_4_-CF_3_), 126.3 (q, C_m_, C_6_H_4_-CF_3_, ^3^*J*_CF_ = 3.7 Hz), 125.7 (q, C_m_, C_6_H_4_-CF_3_, ^3^*J*_CF_ = 3.7 Hz), 122.2 (q, CF_3_, ^1^*J*_CF_ = 272 Hz), 122.1 (q, CF_3_, ^1^*J*_CF_ = 272 Hz), 85.1 (NCH, *trans*-isomer), 82.2 (NCH, *cis*-isomer), 55.8 (SCH, *trans*-isomer), 55.0 (SCH, *cis*-isomer), 53.4 (OMe, *trans*-isomer), 52.3 (OMe, *cis*-isomer). ^19^F NMR(CDCl_3_, 282.40 MHz): δ = −62.76 (s), −62.70 (s). HRMS (ESI^+^) [*m*/*z*]: calcd for [C_18_H_14_F_3_NO_2_SNa]^+^ = [M + Na]^+^, 388.0590; found, 388.0584.

##### (*cis*/*trans*)-Methyl 5-(2-Bromophenyl)-2-phenyl-4,5-dihydrothiazole-4-carboxylate **5k**

Following the general procedure, thiazolone **2k** (100.4 mg, 0.293 mmol) was reacted with BF_3_·OEt_2_ (200 μL, 1.621 mmol) for 18 h in refluxing MeOH (5 mL) to give (*cis*/*trans*)-**5k** (1:0.8 molar ratio) as a yellow oil. In this case, further chromatographic purification was necessary to separate **5k** from starting thiazolone **2k**. The chromatographic purification was started using silica as support and *n*-hexane/Et_2_O (9:1) as an eluent. Using these conditions, only the thiazolone **2k** was eluted. Then the solvent was changed, and 2-propanol was employed. Using these conditions, the dihydrothiazole **5k** was obtained as a yellow oil after solvent evaporation. Obtained: 30 mg (27% yield). ^1^H NMR (CDCl_3_, 300.13 MHz): δ = 7.93 (m, H_o_, NCS-Ph), 7.90 (m, H_o_, NCS-Ph), 7.59 (dd, H_3_, C_6_H_4_Br, ^3^*J*_HH_ = 8.0 Hz, ^4^*J*_HH_ = 1.2 Hz), 7.55–7.52 (m, H_p_, NCS-Ph, both isomers, H_3_, C_6_H_4_Br), 7.49–7.42 (m, H_m_, NCS-Ph, H_6_, C_6_H_4_Br; both isomers), 7.30 (td, H_5_, C_6_H_4_Br, ^3^*J*_HH_ = 7.5 Hz, ^4^*J*_HH_ = 1.2 Hz), 7.22 (td, H_5_, C_6_H_4_Br, ^3^*J*_HH_ = 7.4 Hz, ^4^*J*_HH_ = 1.2 Hz), 7.15 (td, H_4_, C_6_H_4_Br, ^3^*J*_HH_ = 8.0 Hz, ^4^*J*_HH_ = 1.7 Hz), 7.10 (td, H_4_, C_6_H_4_Br, ^3^*J*_HH_ = 7.4 Hz, ^4^*J*_HH_ = 1.7 Hz), 5.91 (d, SCH, ^3^*J*_HH_ = 9.1 Hz, *cis*-isomer), 5.89 (d, SCH, ^3^*J*_HH_ = 4.5 Hz, *trans*-isomer), 5.66 (d, NCH, ^3^*J*_HH_ = 9.1 Hz, *cis*-isomer), 5.47 (d, NCH, ^3^*J*_HH_ = 4.5 Hz, *trans*-isomer), 3.83 (s, OMe, *trans*-isomer), 3.40 (s, OMe, *cis*-isomer). ^13^C{^1^H} NMR (CDCl_3_, 75.5 MHz): δ = 170.9 (SC = N), 170.8 (SC = N), 170.4 (COO), 169.2 (COO), 140.2 (C_1_, C_6_H_4_Br), 137.9 (C_1_, C_6_H_4_Br), 133.2 (C_3_, C_6_H_4_Br), 132.8 (C_3_, C_6_H_4_Br), 132.7 (C_i_, NCS-Ph), 132.6 (C_i_, NCS-Ph), 132.1 (C_p_, NCS-Ph), 132.1 (C_p_, NCS-Ph), 129.8 (C_4,_C_6_H_4_Br), 129.7 (C_4,_C_6_H_4_Br), 129.2, 128.9 (C_o_, C_m_, NCS-Ph, both isomers), 128.8 (C_6_, C_6_H_4_Br), 128.8 (C_6_, C_6_H_4_Br), 128.5 (C_5_, C_6_H_4_Br), 128.1 (C_5_, C_6_H_4_Br), 123.8 (C_2_-Br, C_6_H_4_Br), 123.7 (C_2_-Br, C_6_H_4_Br), 85.1 (NCH, *trans*-isomer), 82.6 (NCH, *cis*-isomer), 55.3 (SCH, *trans*-isomer), 54.5 (SCH, *cis*-isomer), 53.1 (OMe, *trans*-isomer), 52.0 (OMe, *cis*-isomer). HRMS (ESI^+^) [*m*/*z*]: calcd for [C_17_H_14_BrNO_2_SNa]^+^ = [M + Na]^+^, 397.9821; found, 397.9808.

##### (*cis*/*trans*)-Methyl 5-(3,4-Dimethylphenyl)-2-phenyl-4,5-dihydrothiazole-4-carboxylate **5m**

Following the general procedure, thiazolone **2m** (101.73 mg, 0.347 mmol) was reacted with BF_3_·OEt_2_ (200 μL, 1.621 mmol) for 18 h in refluxing MeOH (5 mL) to give (*cis*/*trans*)-**5m** (1:0.8 molar ratio) as a yellow oil. Obtained: 95 mg (84% yield). ^1^H NMR (CDCl_3_, 300.13 MHz): δ = 7.98 (m, 2H, NCS-Ph), 7.92 (m, H_o_, NCS-Ph), 7.55–7.41 (m, H_m_, H_p_, NCS-Ph, both isomers), 7.18 (s, H_2_, C_6_H_3_(Me)_2_), 7.14–7.12 (m, H_5_, H_6_, C_6_H_3_(Me)_2_), 7.03 (s, H_2_, C_6_H_3_(Me)_2_), 7.02 (m, H_5_, H_6_, C_6_H_3_(Me)_2_), 5.55 (d, NCH, ^3^*J*_HH_ = 9.0 Hz, *cis*-isomer), 5.43 (d, SCH, ^3^*J*_HH_ = 6.7 Hz, *trans*-isomer), 5.38 (d, NCH, ^3^*J*_HH_ = 6.7 Hz, *trans*-isomer), 5.22 (d, SCH, ^3^*J*_HH_ = 9.0 Hz, *cis*-isomer), 3.82 (s, OMe, *trans*-isomer), 3.42 (s, OMe, *cis*-isomer), 2.25 (s, Me, *trans*-isomer). 2.20 (s, Me, *cis*-isomer). ^13^C{^1^H} NMR (CDCl_3_, 75.5 MHz): δ = 170.9 (COO + SC = N, overlapped), 170.6 (SC = N), 169.4 (COO), 137.7 (C_i_, C_6_H_3_(Me)_2_), 137.4, 136.9, 136.8, 136.7 (C_q_, C_3_, C_4_, C_6_H_3_(Me)_2_ both isomers), 135.5 (C_i_, C_6_H_3_(Me)_2_), 132.7 (C_i_, NCS-Ph), 132.7 (C_i_, NCS-Ph), 131.9 (C_p_, NCS-Ph), 131.9 (C_p_, NCS-Ph), 130.3, 129.7, 125.3, 124.3 (C_5_, C_6_, C_6_H_3_(Me)_2_, both isomers), 129.0, 128.9, 128.6 (C_o_, C_m_, NCS-Ph, both isomers), 128.7 (C_2_, C_6_H_3_(Me)_2_), 128.7 (C_2_, C_6_H_3_(Me)_2_), 86.5 (NCH, *trans*-isomer), 83.8 (NCH, *cis*-isomer), 56.5 (SCH, *trans*-isomer), 55.7 (SCH, *cis*-isomer), 52.9 (OMe, *trans*-isomer), 51.9 (OMe, *cis*-isomer), 19.9, 19.8, 19.5, 19.5 (Me). HRMS (ESI^+^) [*m*/*z*]: calcd for [C_19_H_19_NO_2_SNa]^+^ = [M + Na]^+^, 348.1029; found, 348.1032.

## Supporting Information Available

Copies of ^1^H and ^13^C NMR spectra for all new compounds, copies of the absorption (UV–vis) spectra of thiazolones **2** and selected cyclobutanes **3**, NMR spectra, and UV–vis spectra of **2b** in the presence of BF_3_ (PDF)

## Accession Codes

CCDC 1912941–1912942, 1958992–1958993, and 1999650 contain the supplementary crystallographic data for this paper. These data can be obtained free of charge via www.ccdc.cam.ac.uk/data_request/cif, or by emailing data_request@ccdc.cam.ac.uk, or by contacting The Cambridge Crystallographic Data Centre, 12 Union Road, Cambridge CB2 1EZ, UK; fax: +44 1223 336033.
