# Supplementary material for: Reactivity of (Z)-4-Aryliden-5(4H)-thiazolones: [2 + 2]-Photocycloaddition, Ring-Opening Reactions, and Influence of the Lewis Acid BF3
Source: J Org Chem. 2021 Aug 16;86(17):12119–40. doi: 10.1021/acs.joc.1c01458 (PMC9129068; doi:10.1021/acs.joc.1c01458)
Supplement: Supplementary file 1 — jo1c01458_si_001.pdf [file jo1c01458_si_001.pdf]

Reactivity of (*Z*)-4-Aryliden-5(4*H*)-Thiazolones: [2+2]-photocycloaddition, ring-opening reactions and influence of the Lewis Acid BF_3

Sonia Sierra,† David Dalmau,† Sheila Higuera,† Darío Cortés,† Olga Crespo,† Ana I. Jimenez,† Alexandra Pop,‡ Cristian Silvestru,‡ Esteban P. Urriolabeitia*†

† Instituto de Síntesis Química y Catálisis Homogénea, ISQCH (CSIC-Universidad de Zaragoza), Pedro Cerbuna 12, E-50009 Zaragoza (Spain)

‡ Supramolecular Organic and Organometallic Chemistry Centre, Departament of Chemistry, Faculty of Chemistry and Chemical Engineering, Babeş-Bolyai University, Str. Arany Janos 11, RO-400028 Cluj–Napoca, (Romania)

Corresponding author e-mail address: esteban@unizar.es

Supporting Information

Index of this supporting information

Copies of ^1H and ^{13}C NMR spectra of all synthesized species

1.- NMR spectra of (*Z*)-4-arylidene-5(4*H*)-thiazolones **2** **S1**

2.- NMR spectra of [2+2]-photocycloaddition products **3** **S28**

3.- NMR spectra of [2+2]-photocycloaddition products **4** **S57**

4.- NMR spectra of dihydrothiazoles **5** and **6**, and thiazoles **7** (absence of BF_3) **S66**

5.- NMR spectra of dihydrothiazoles **5** (presence of BF_3) **S84**

6.- UV-Vis spectra of thiazolones **2** and selected cyclobutanes **3** **S103**

7.- NMR spectra and UV-Vis spectra of **2b** in presence of BF_3 **S111**

8.- ORTEP of compounds **3g**, **3h**, **3m**, **4b**·CH_2Cl_2 and **7n** and crystallographic data **S112**

Copies of ^{1}H and ^{13}C NMR spectra of all synthesized species

1. NMR of 4-arylidene-5(4*H*)-thiazolones 2

(*Z*)-4-(4-benzylidene)-2-phenyl-5(4*H*)-thiazolone 2a

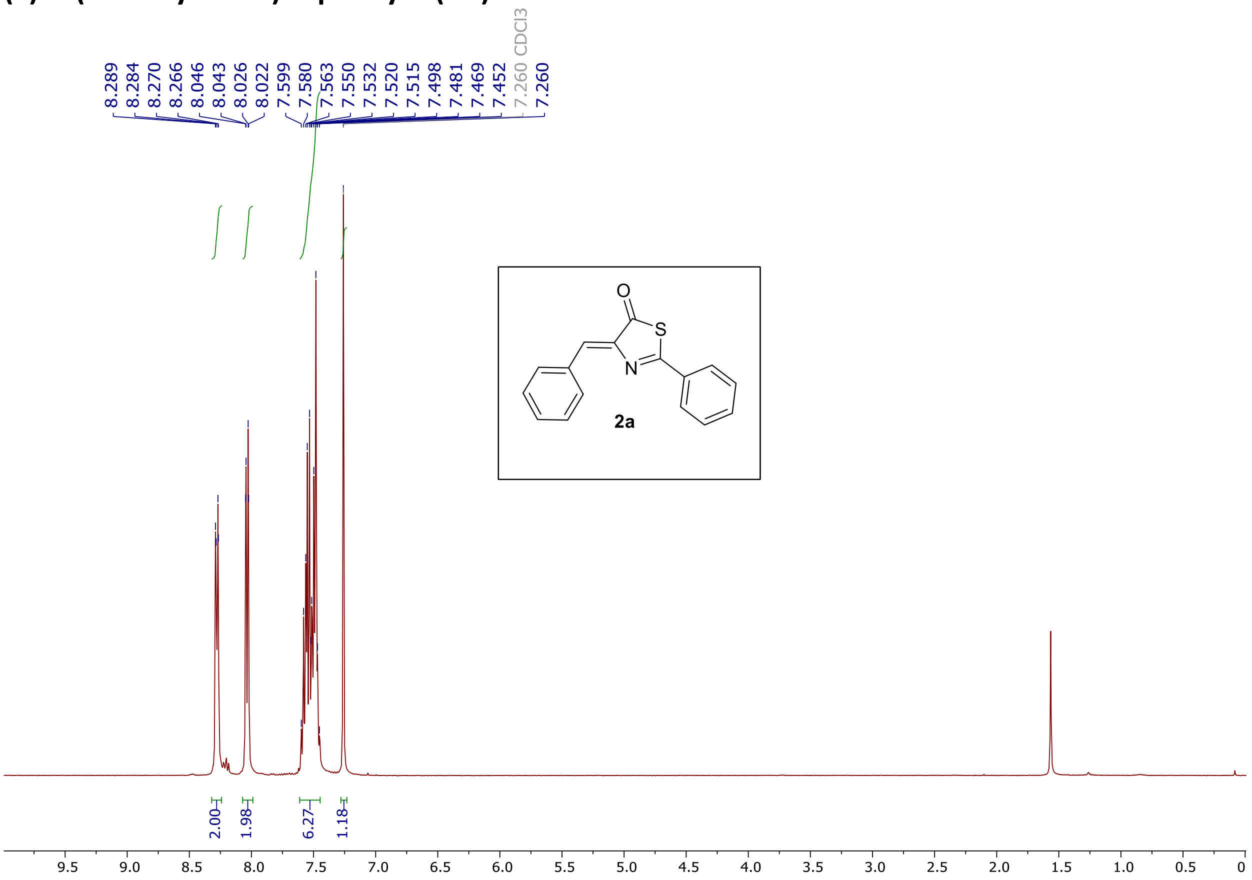

^{1}H NMR spectrum ($CDCl_3$, 300.13 MHz) of **2a**

(*Z*)-4-(4-methylbenzylidene)-2-phenyl-5(4*H*)-thiazolone 2b

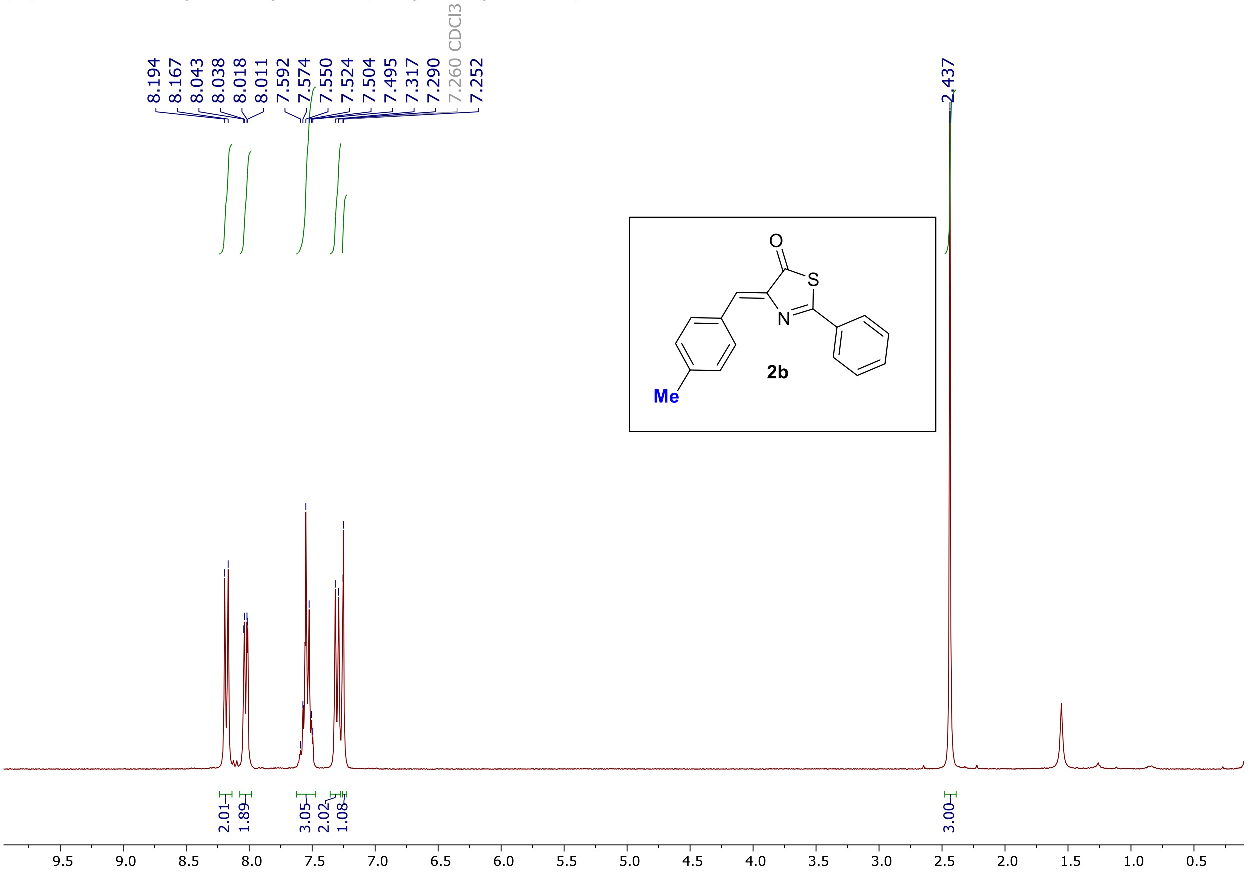

^{1}H NMR spectrum (CD_2Cl_2, 300.13 MHz) of **2b**

(*Z*)-4-(4-methoxybenzylidene)-2-phenyl-5(4*H*)-thiazolone 2c

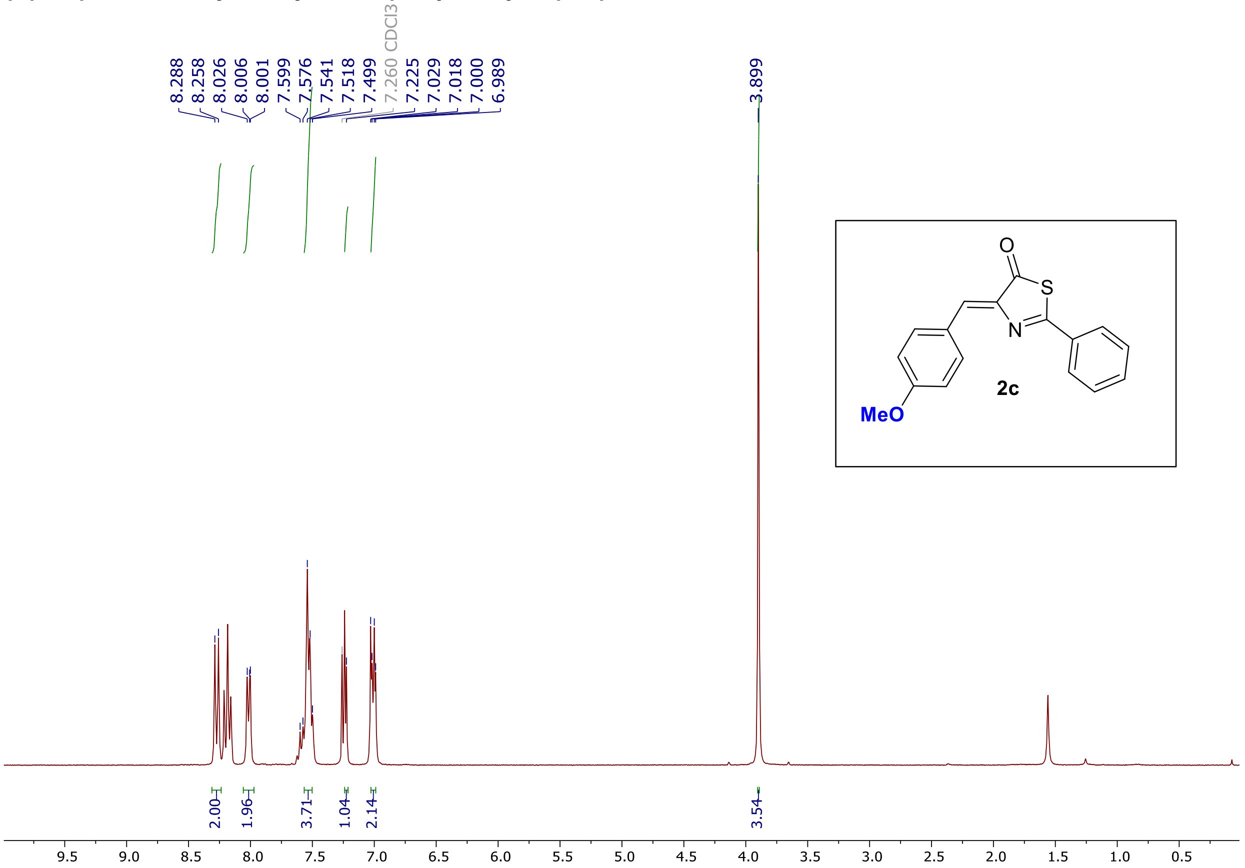

^{1}H NMR spectrum (CD_2Cl_2, 300.13 MHz) of **2c**

(*Z*)-4-(4-fluorobenzylidene)-2-phenyl-5(4*H*)-thiazolone 2d

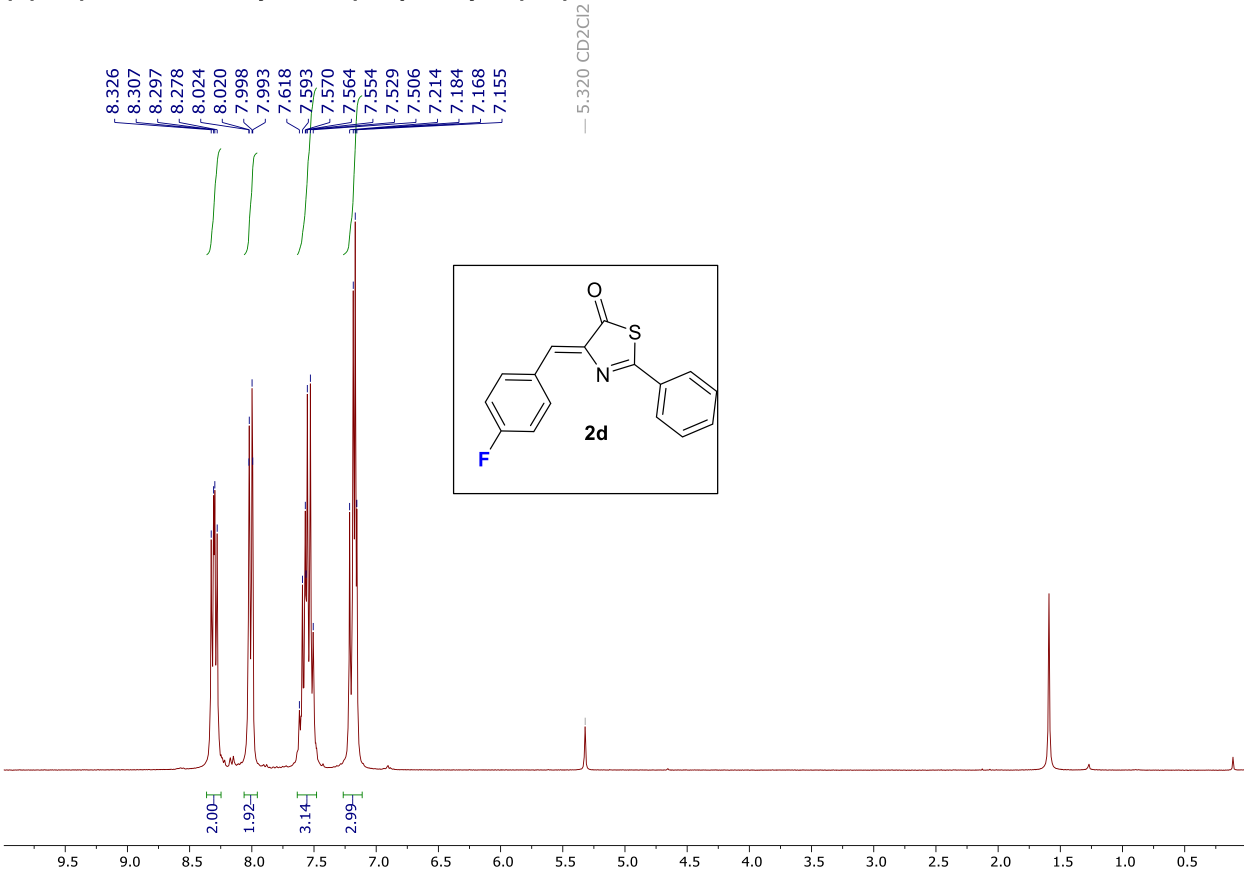

^{1}H NMR spectrum (CD_2Cl_2, 300.13 MHz) of **2d**

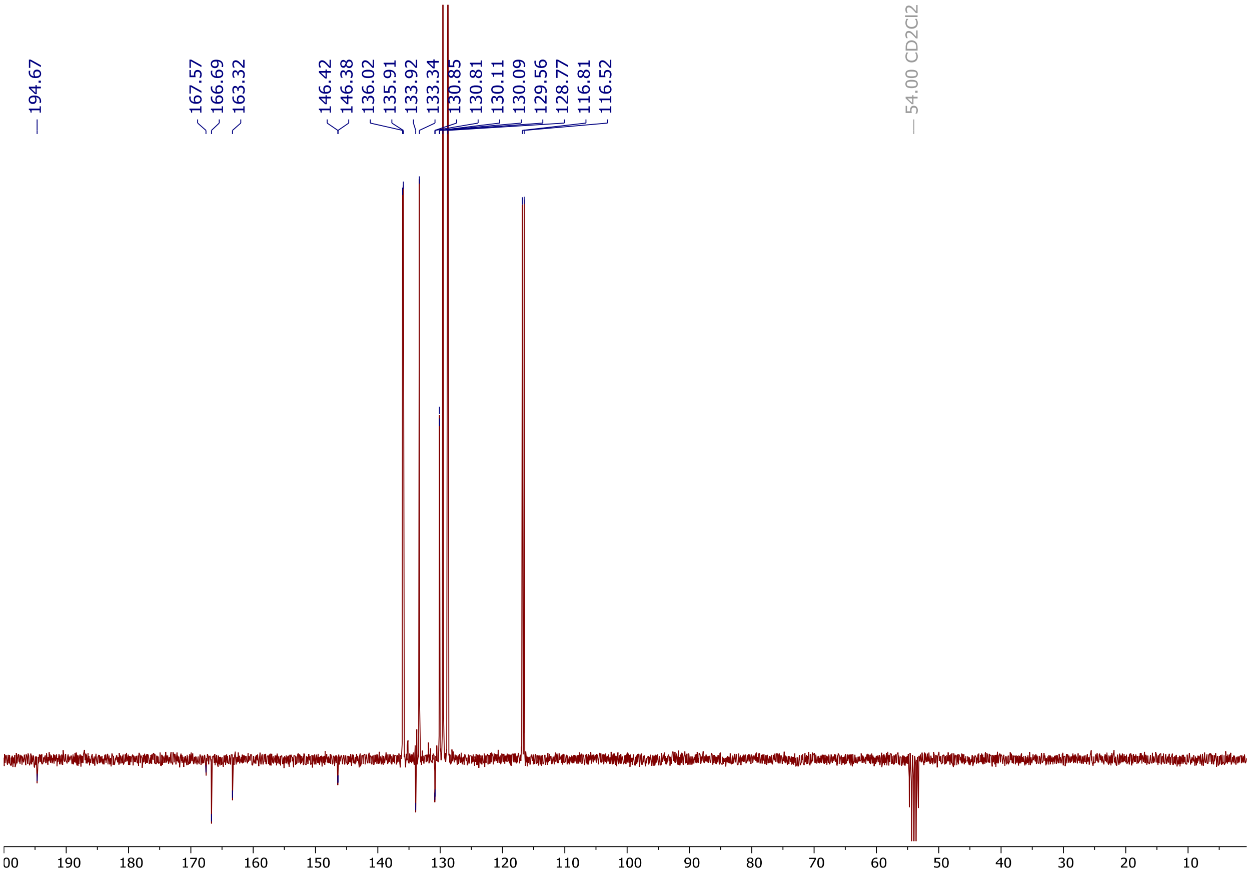

$^{13}C\{^{1}H\}$ NMR (APT) spectrum (CD_2Cl_2, 75.5 MHz) of **2d**

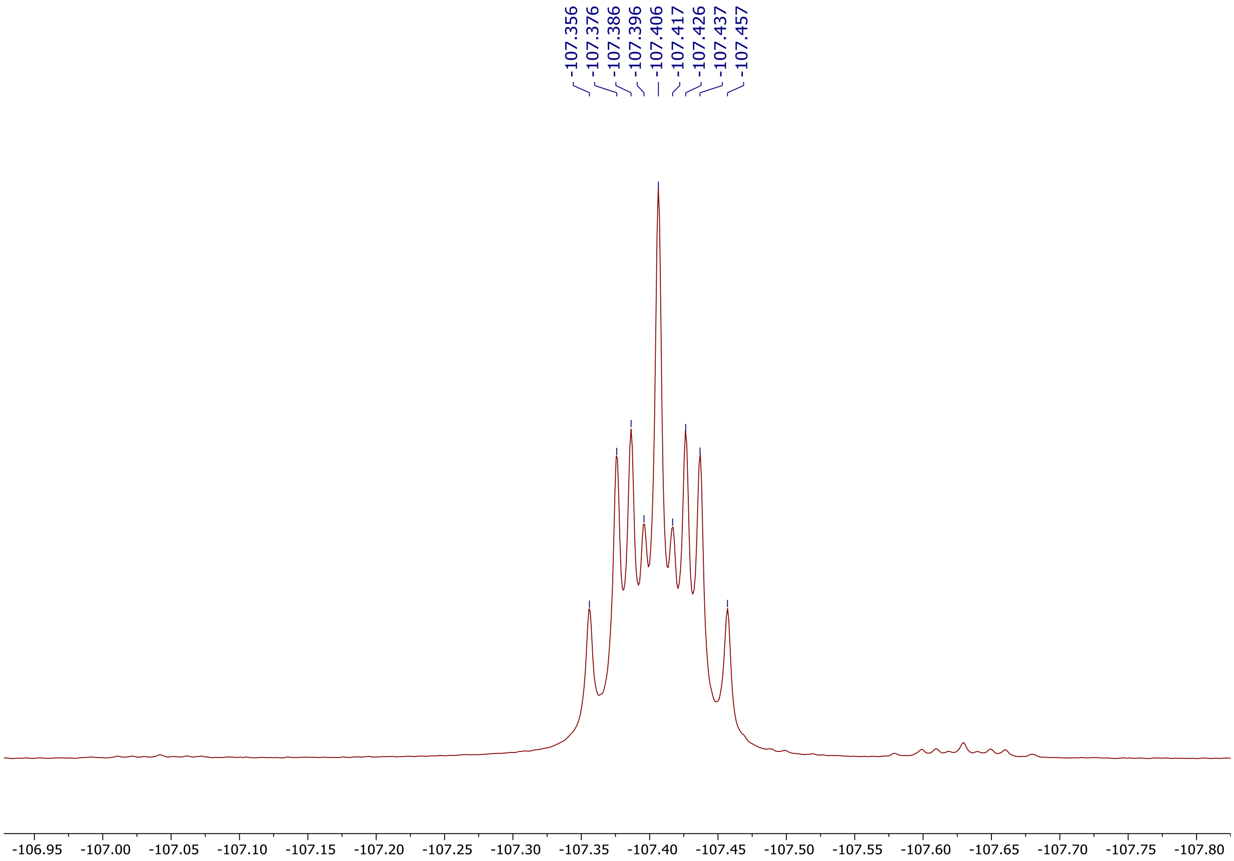

^{19}F NMR spectrum (CD_2Cl_2, 282.4 MHz) of **2d**

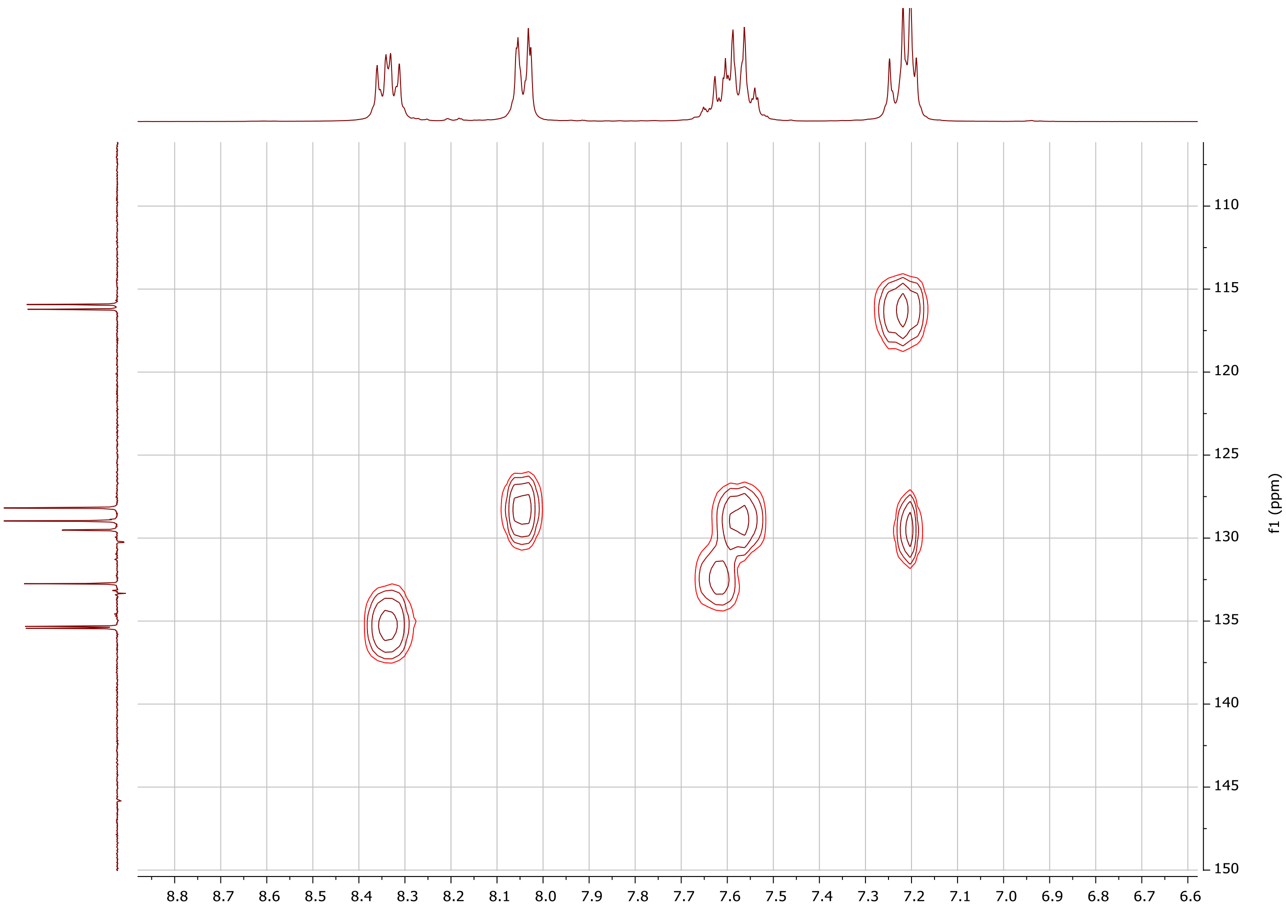

^1H-^{13}C HSQC correlation spectrum of **2d**

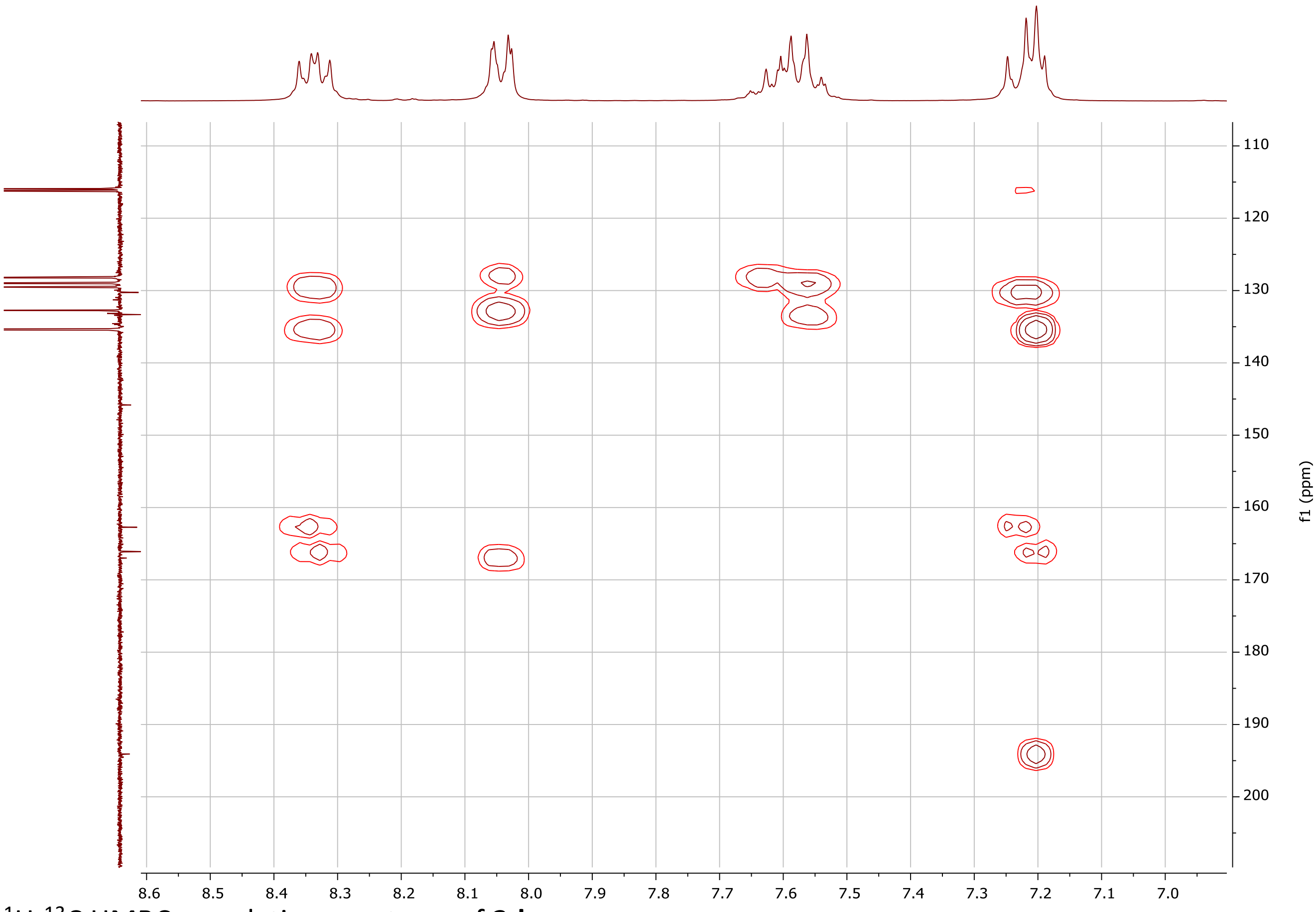

^1H-^{13}C HMBC correlation spectrum of **2d**

(*Z*)-4-(4-chlorobenzylidene)-2-phenyl-5(4*H*)-thiazolone 2e

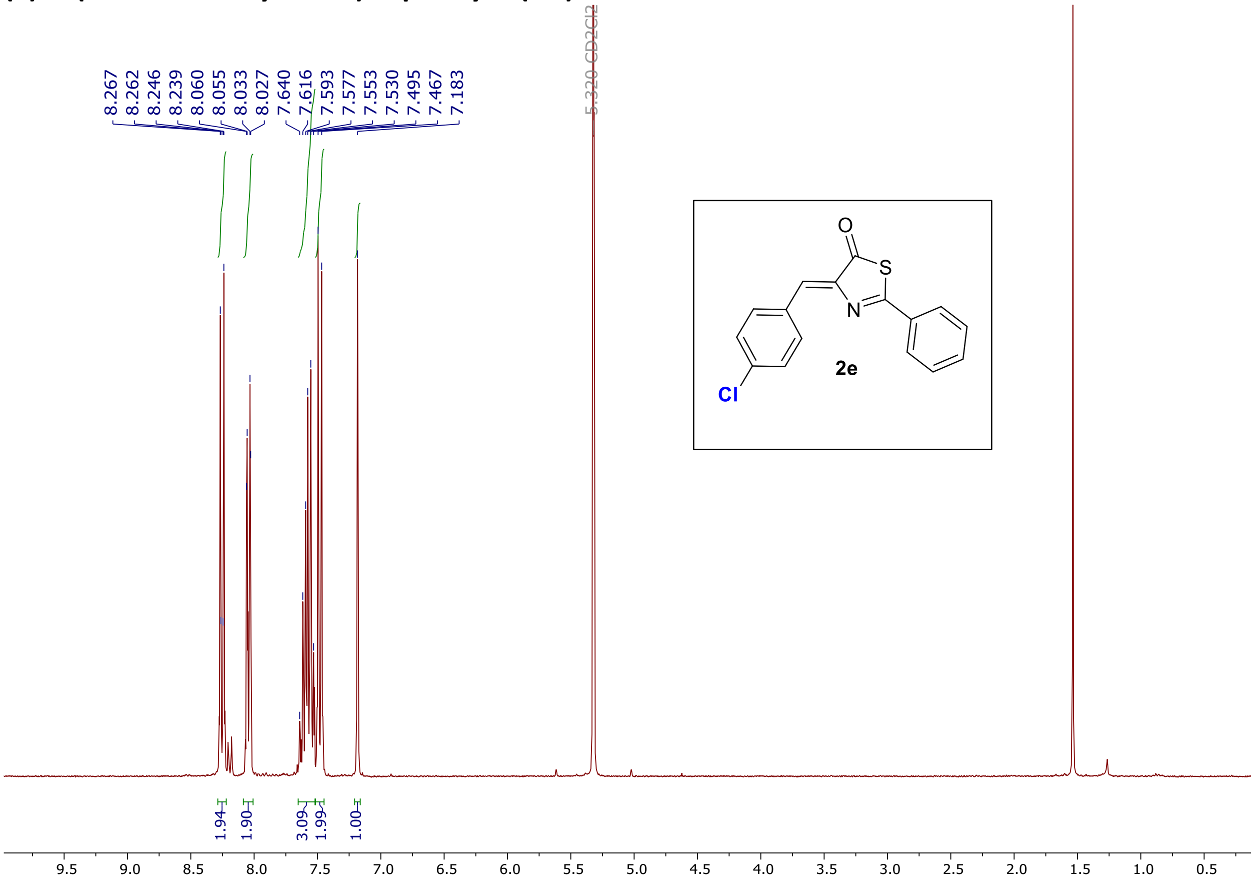

^{1}H NMR spectrum (CD_2Cl_2, 300.13 MHz) of **2e**

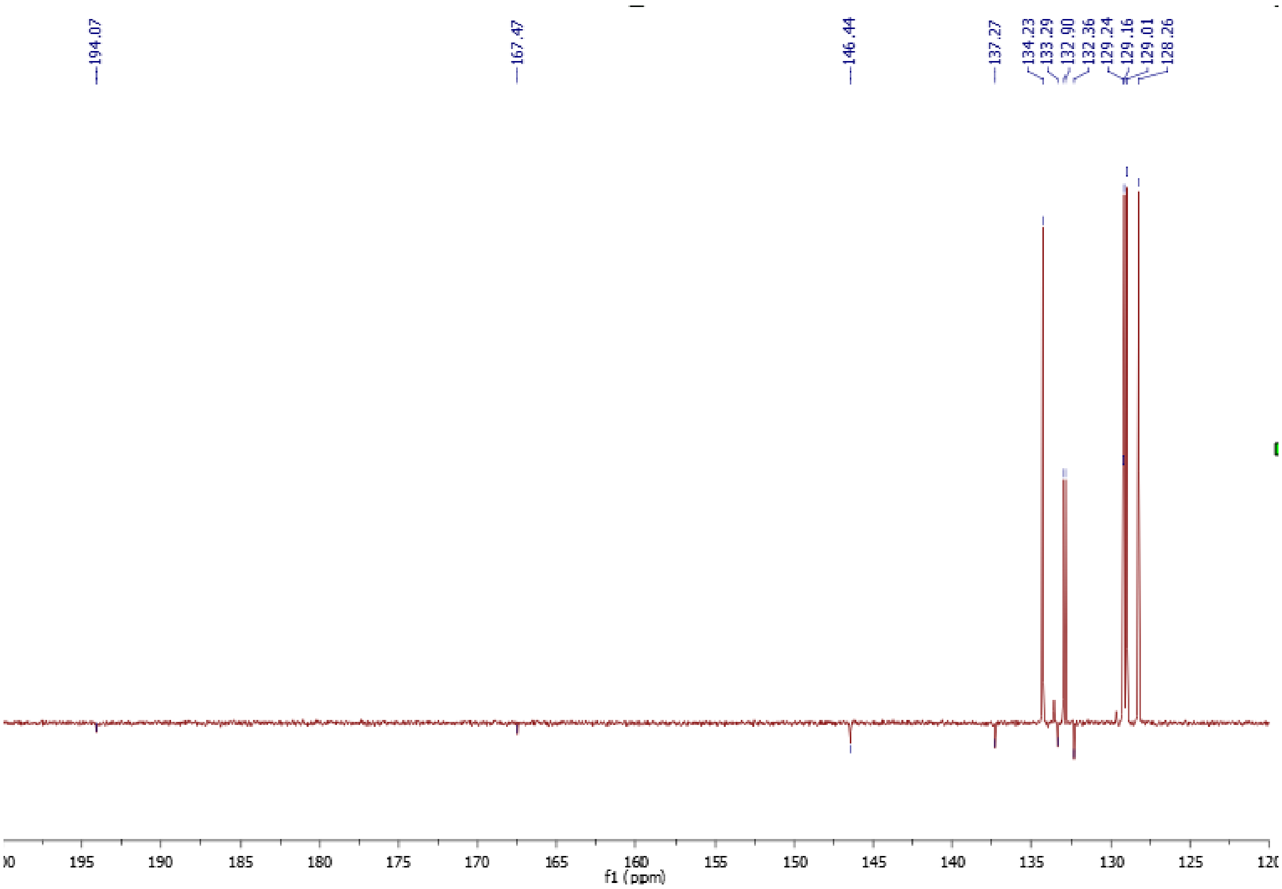

$^{13}C\{^1H\}$ NMR (APT) spectrum (CD_2Cl_2, 75.5 MHz) of **2e**

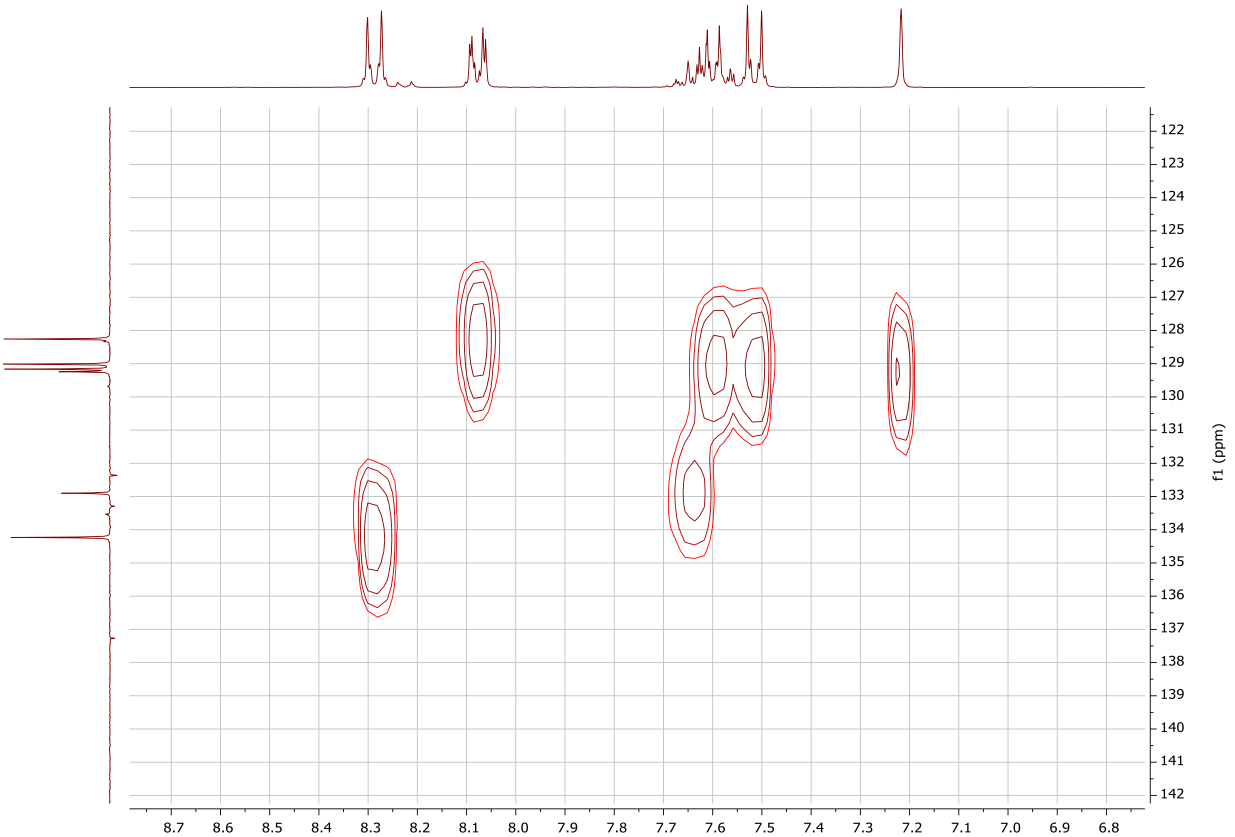

^{1}H-^{13}C HSQC correlation spectrum of **2e**

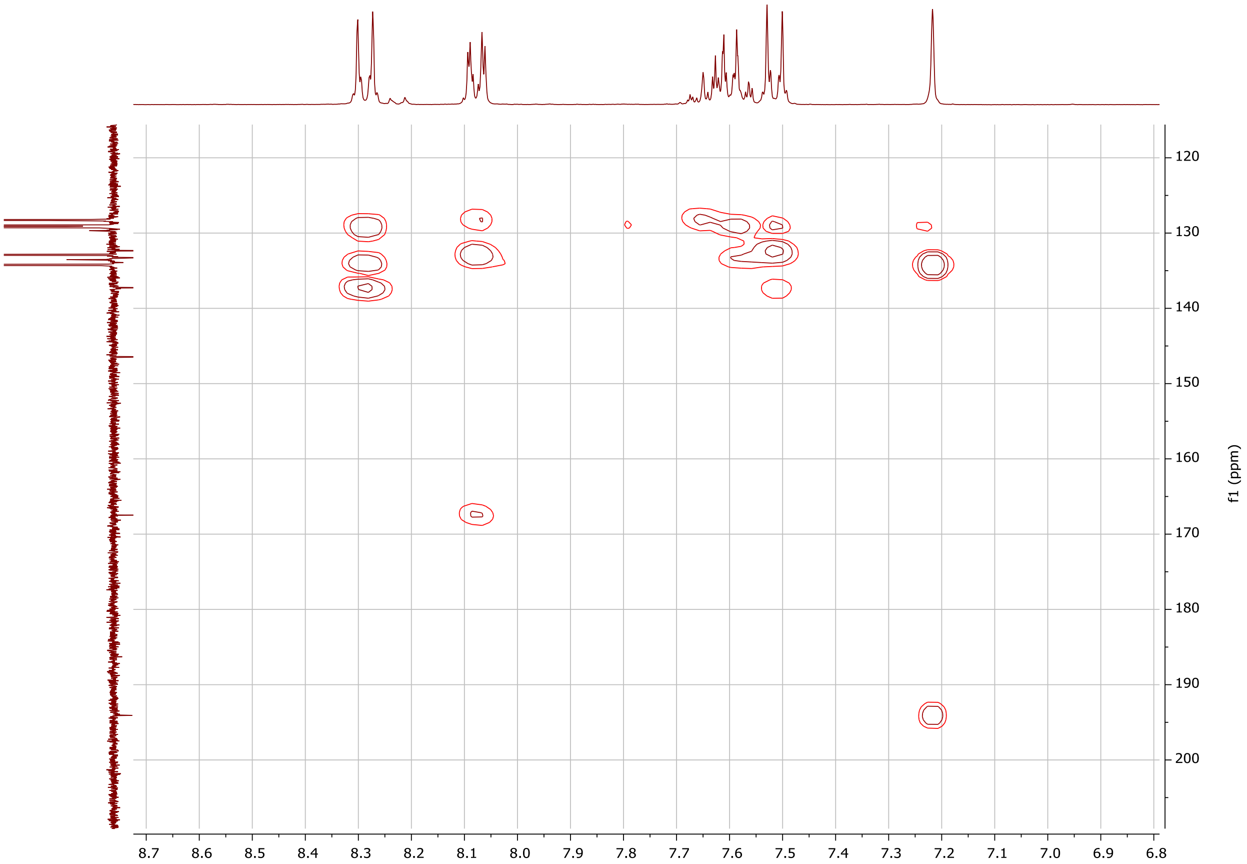

^{1}H-^{13}C HMBC correlation spectrum of **2e**

(*Z*)-4-(4-bromobenzylidene)-2-phenyl-5(4*H*)-thiazolone 2f

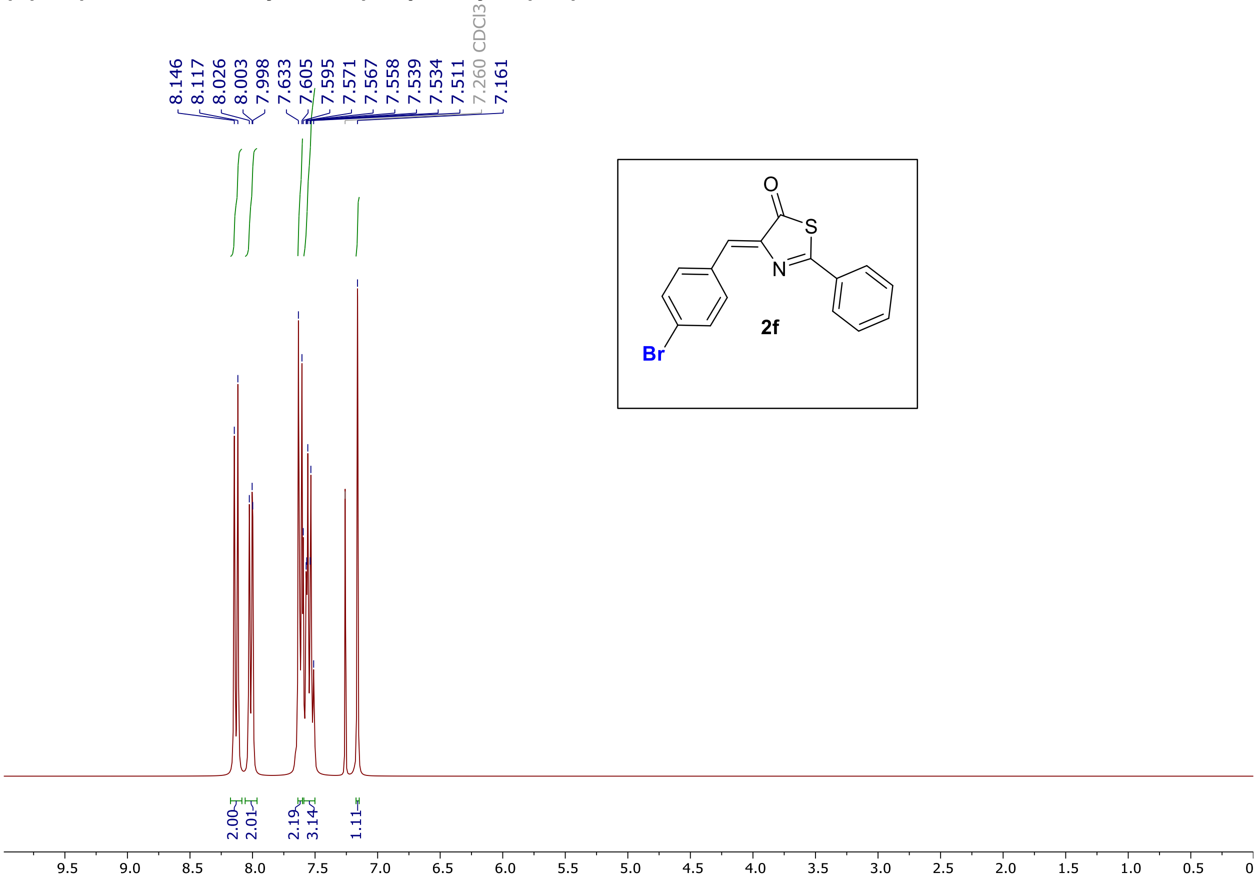

^{1}H NMR spectrum ($CDCl_3$, 300.13 MHz) of **2f**

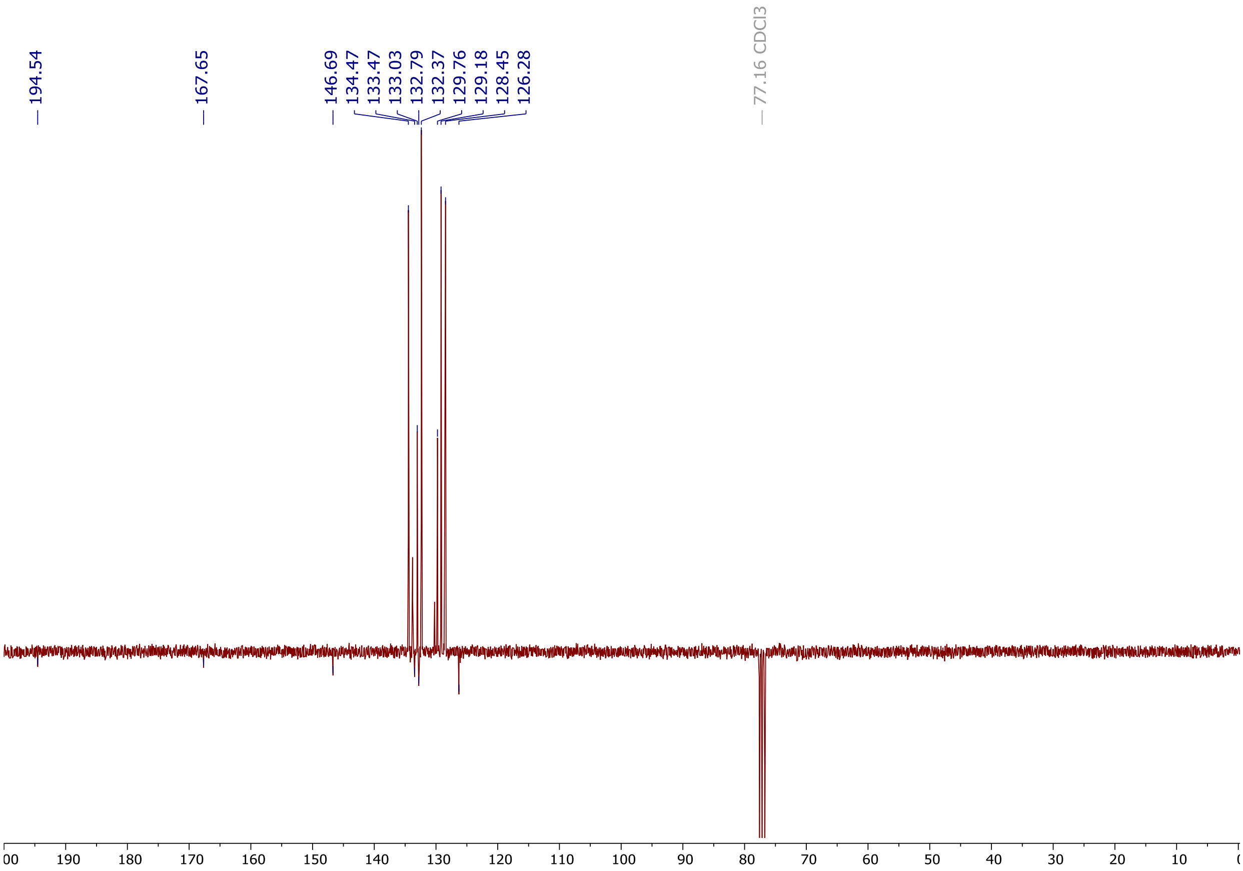

$^{13}C\{^{1}H\}$ NMR (APT) spectrum ($CDCl_3$, 75.5 MHz) of **2f**

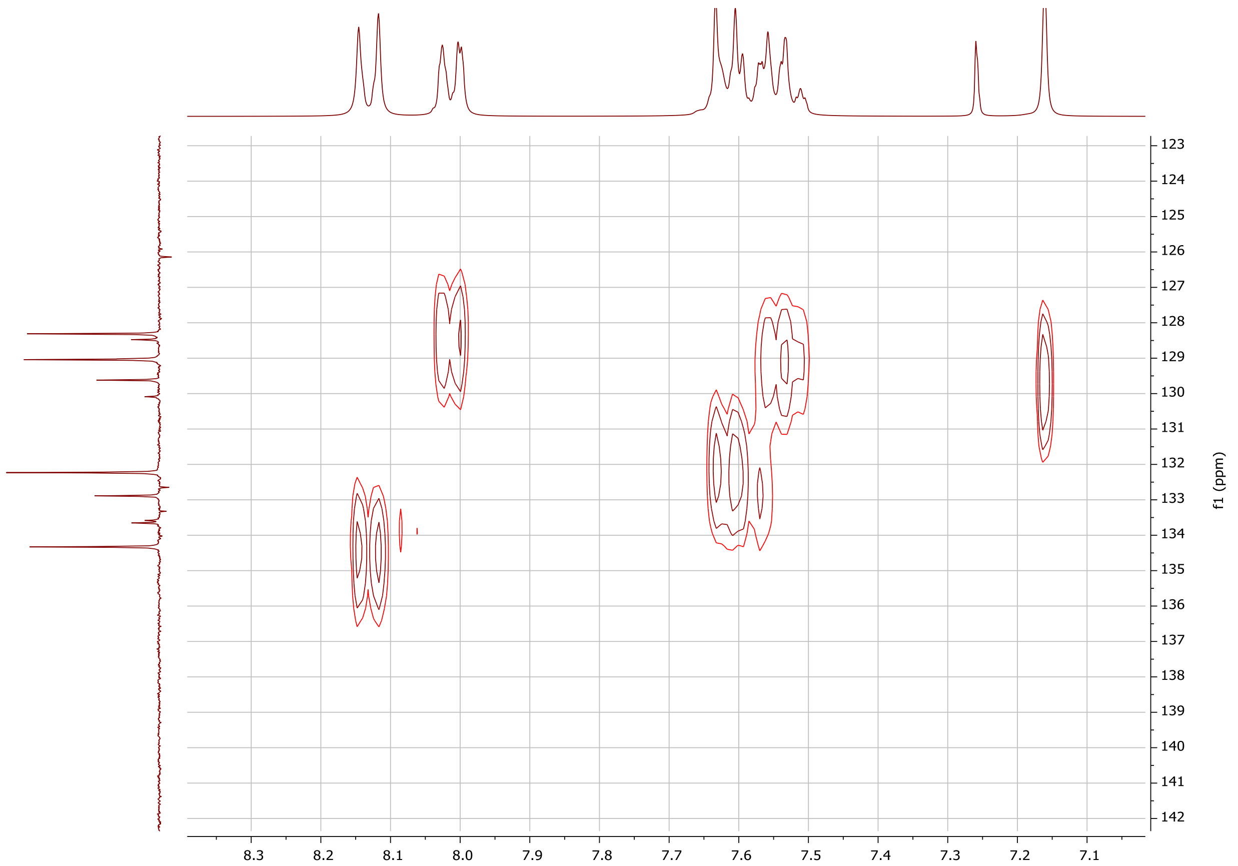

^{1}H-^{13}C HSQC correlation spectrum of **2f**

(*Z*)-4-(4-nitrobenzylidene)-2-phenyl-5(4*H*)-thiazolone 2g

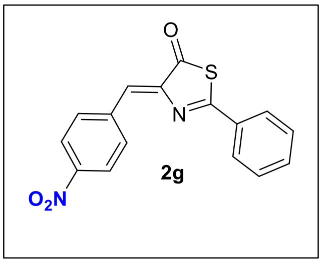

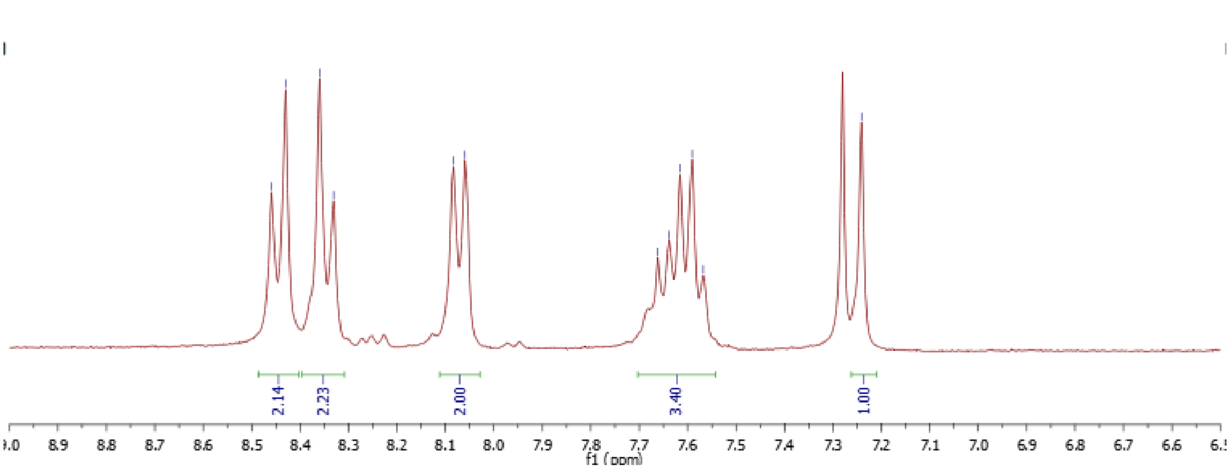

^{1}H NMR spectrum ($CDCl_3$, 300.13 MHz) of **2g**

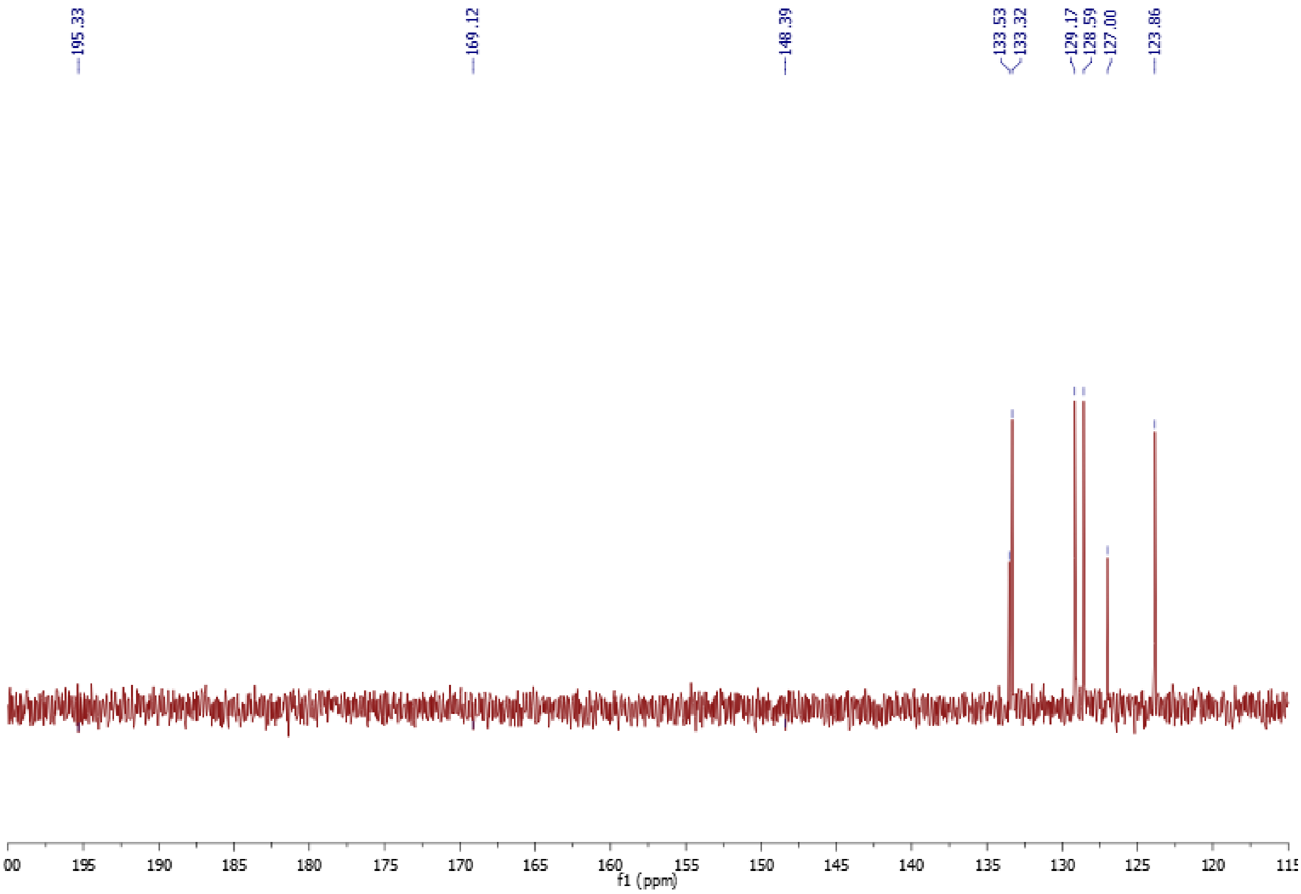

$^{13}C\{^{1}H\}$ NMR (APT) spectrum ($CDCl_3$, 75.5 MHz) of **2g**

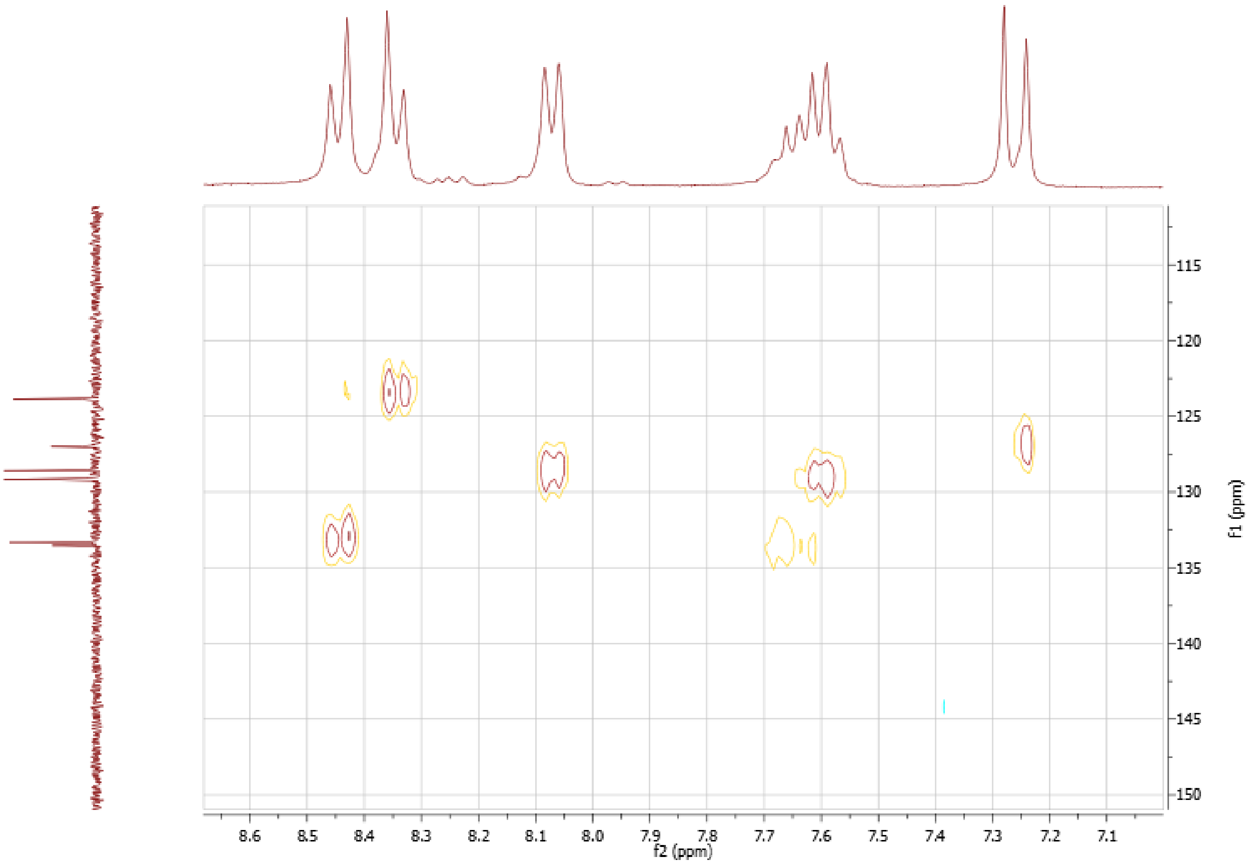

^{1}H-^{13}C HSQC correlation spectrum of **2g**

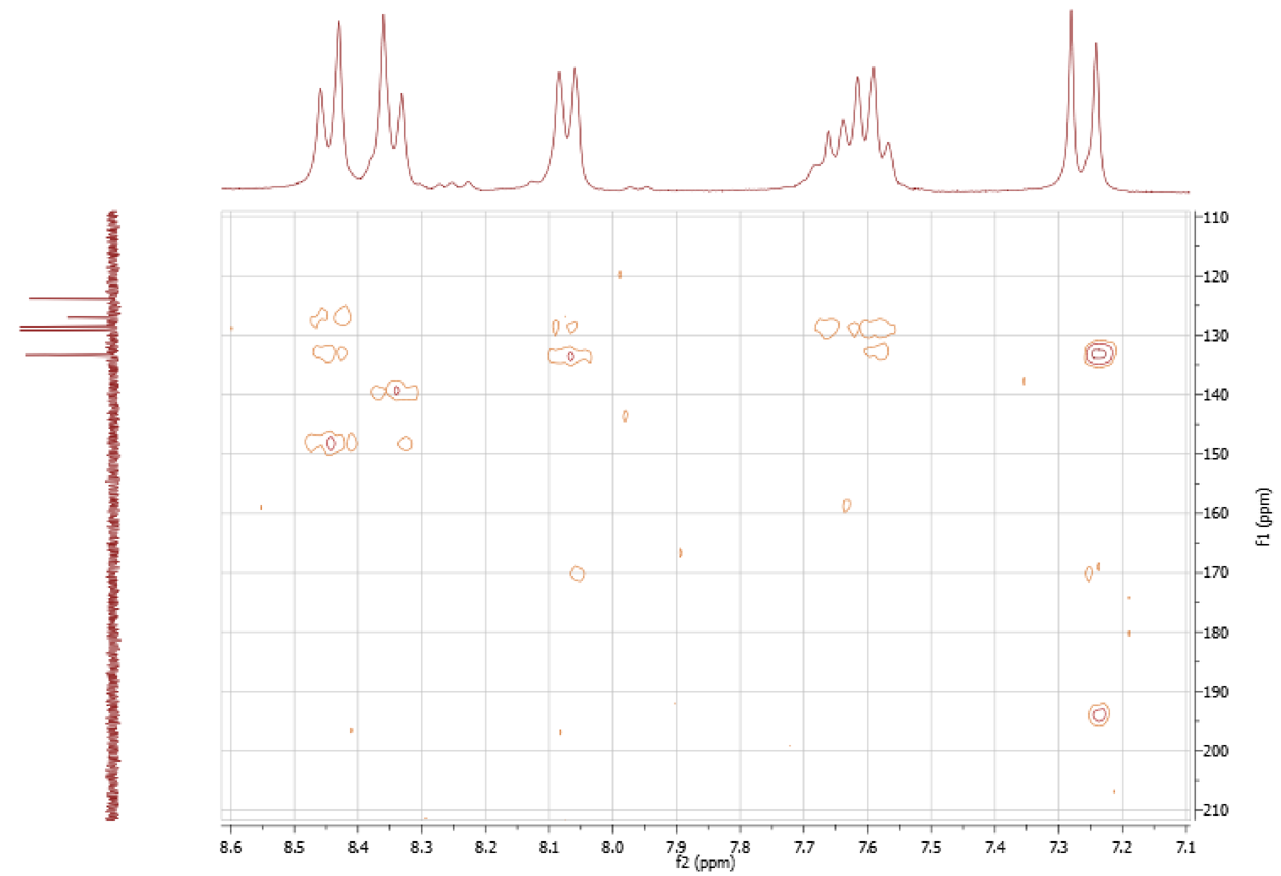

^{1}H-^{13}C HMBC correlation spectrum of **2g**

(*Z*)-4-(4-trifluoromethylbenzylidene)-2-phenyl-5(4*H*)-thiazolone 2h

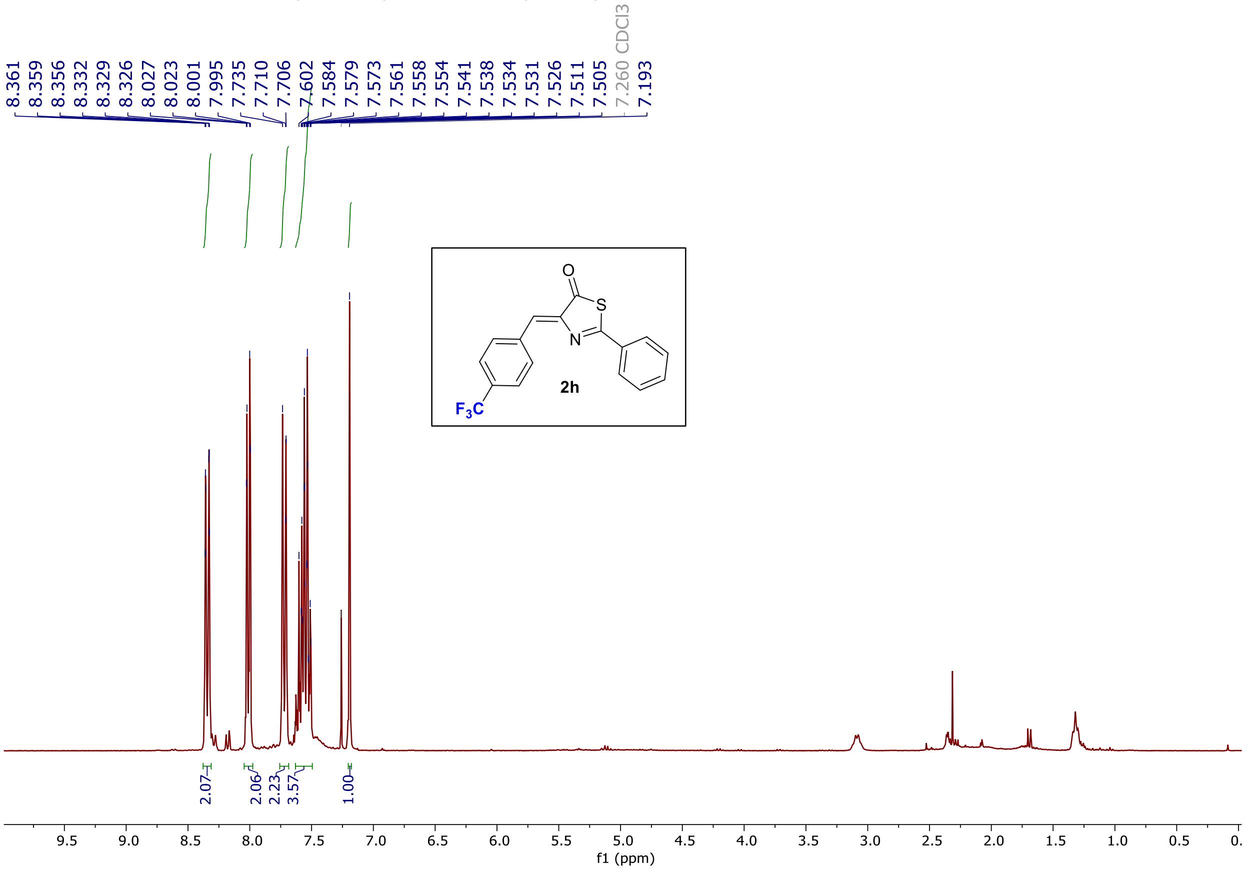

^{1}H NMR spectrum ($CDCl_3$, 300.13 MHz) of **2h**

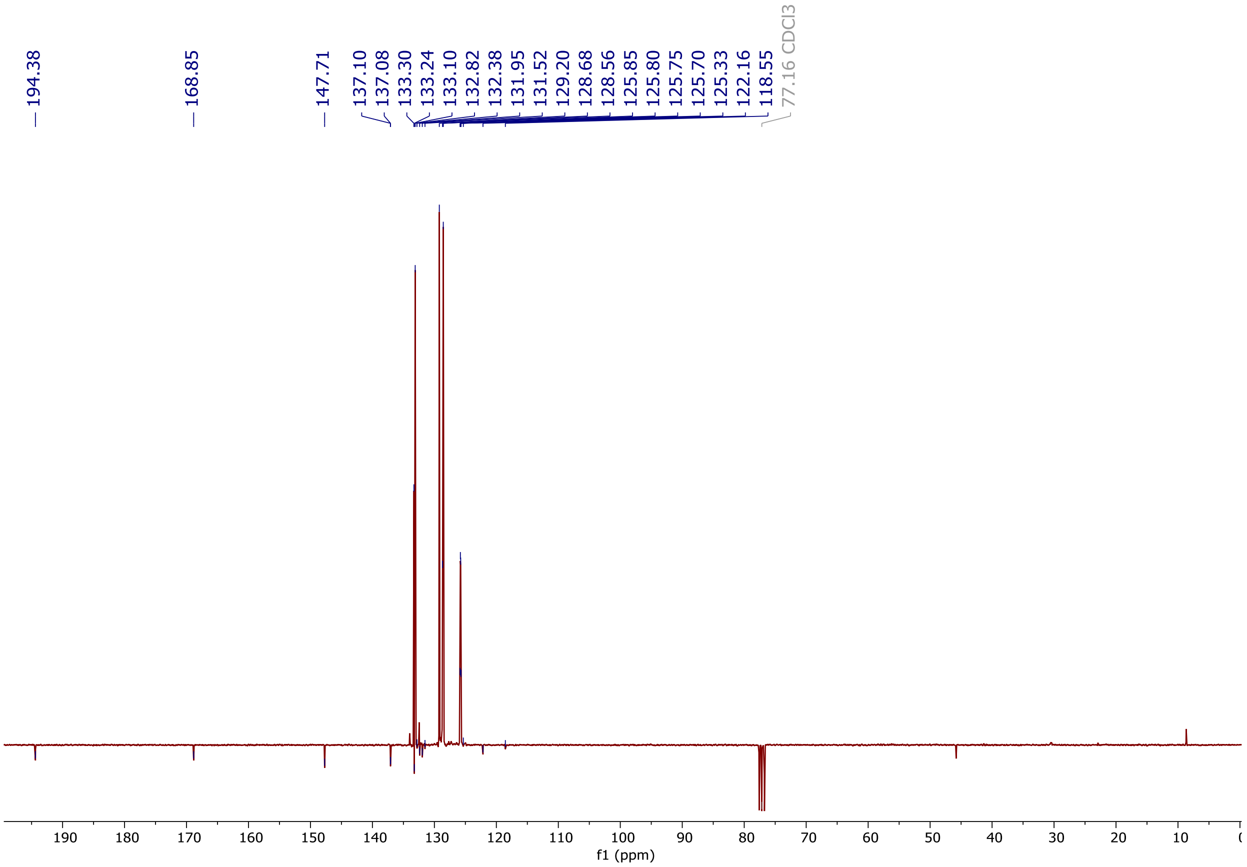

$^{13}C\{^{1}H\}$ NMR (APT) spectrum ($CDCl_3$, 75.5 MHz) of **2h**

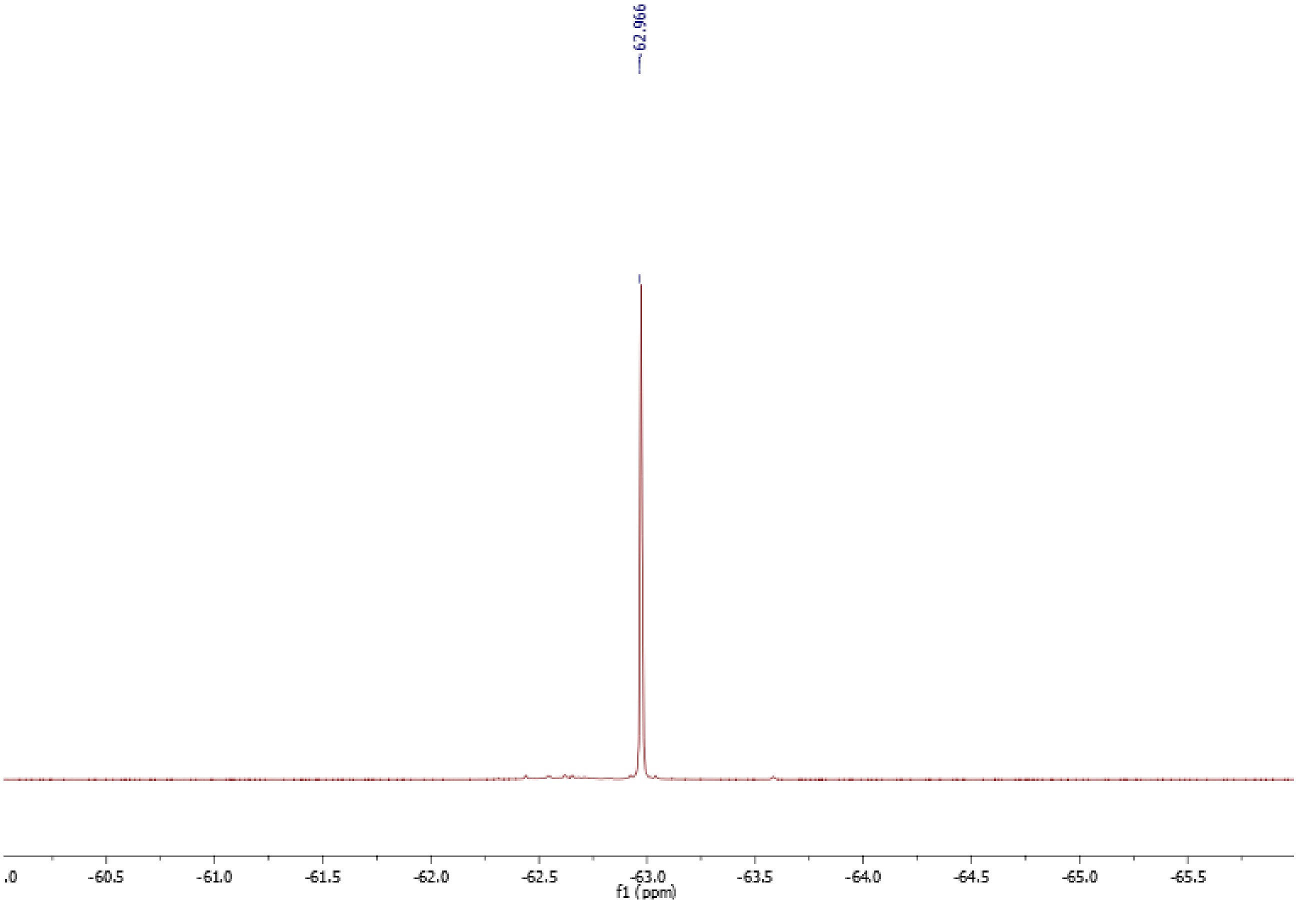

^{19}F NMR spectrum ($CDCl_3$, 282.4 MHz) of **2h**

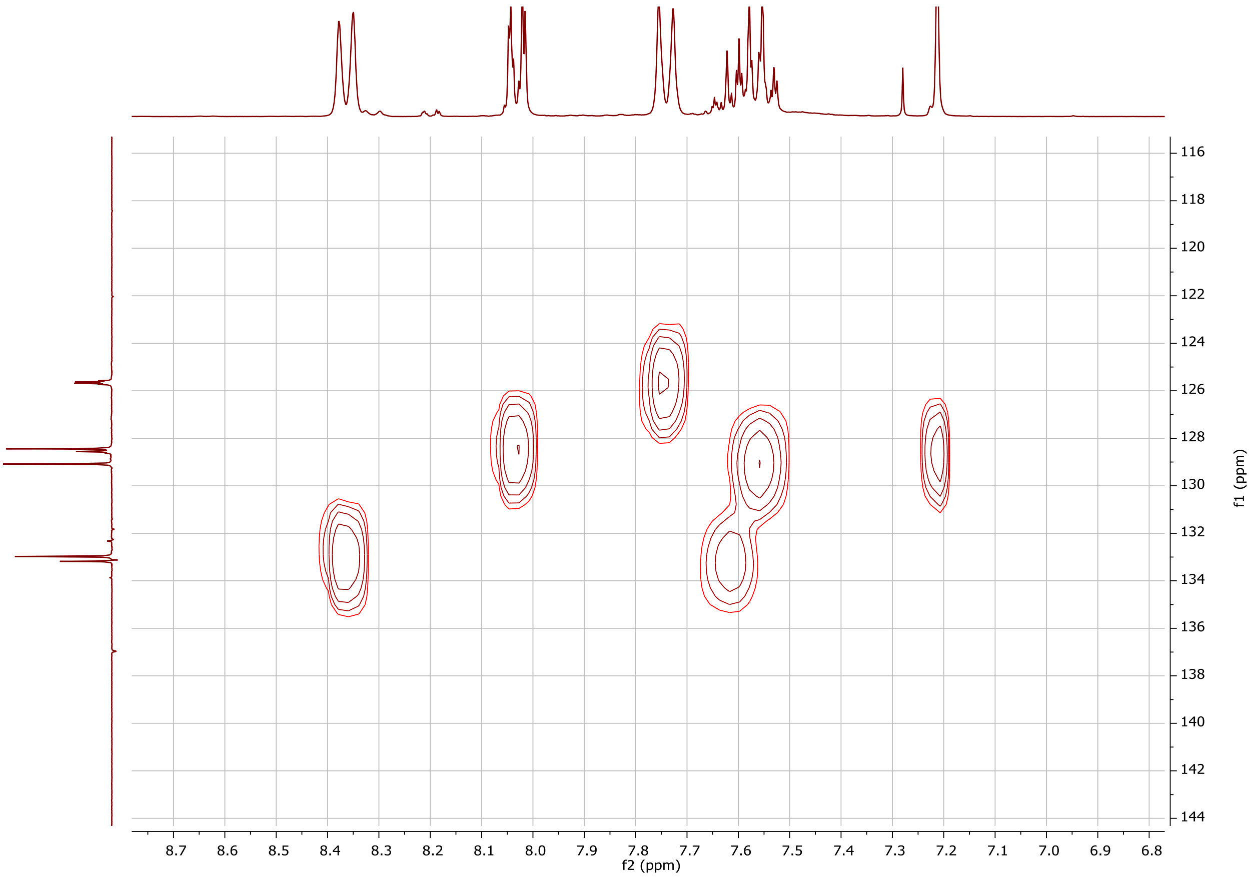

^{1}H-^{13}C HSQC correlation spectrum of **2h**

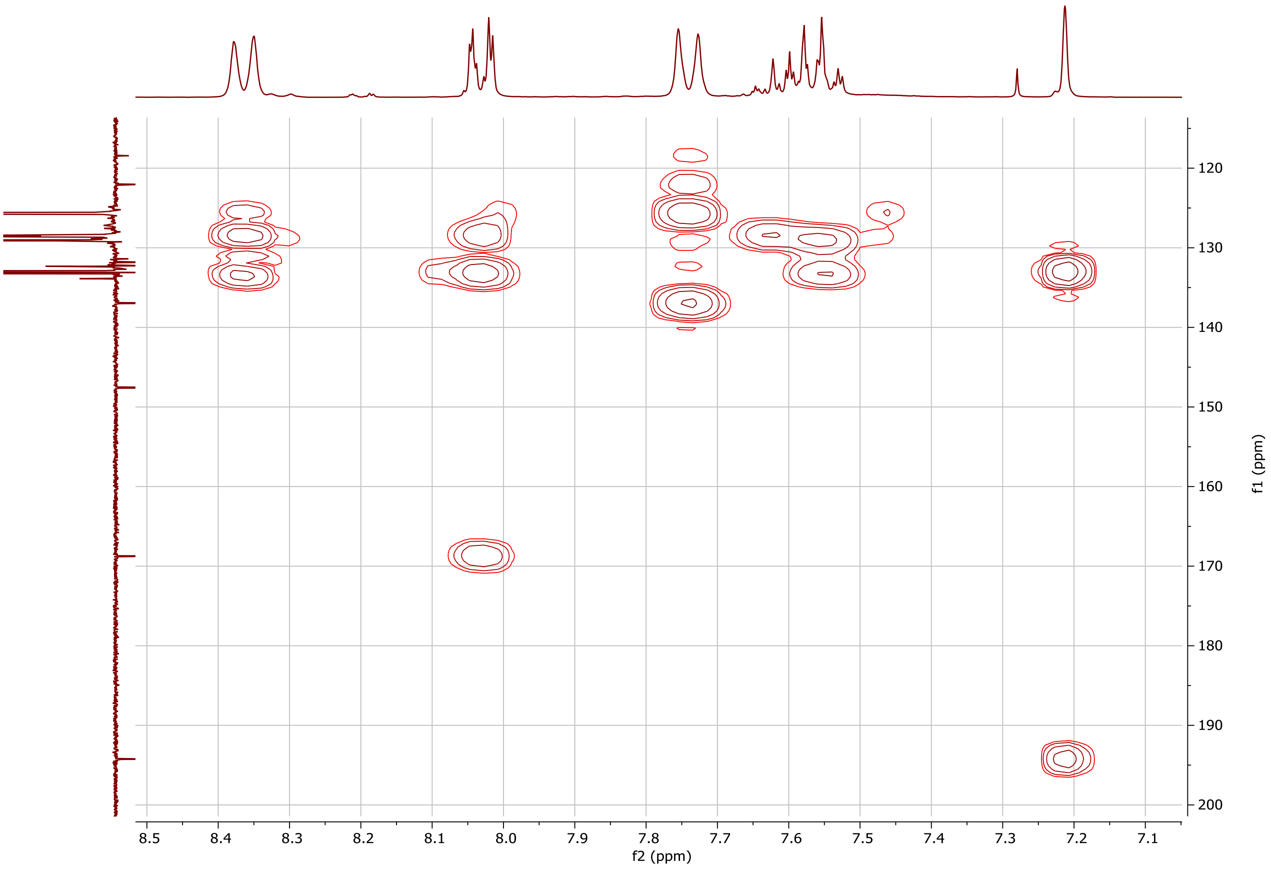

^{1}H-^{13}C HMBC correlation spectrum of **2h**

(*Z*)-4-(2-methoxylbenzylidene)-2-phenyl-5(4*H*)-thiazolone 2i

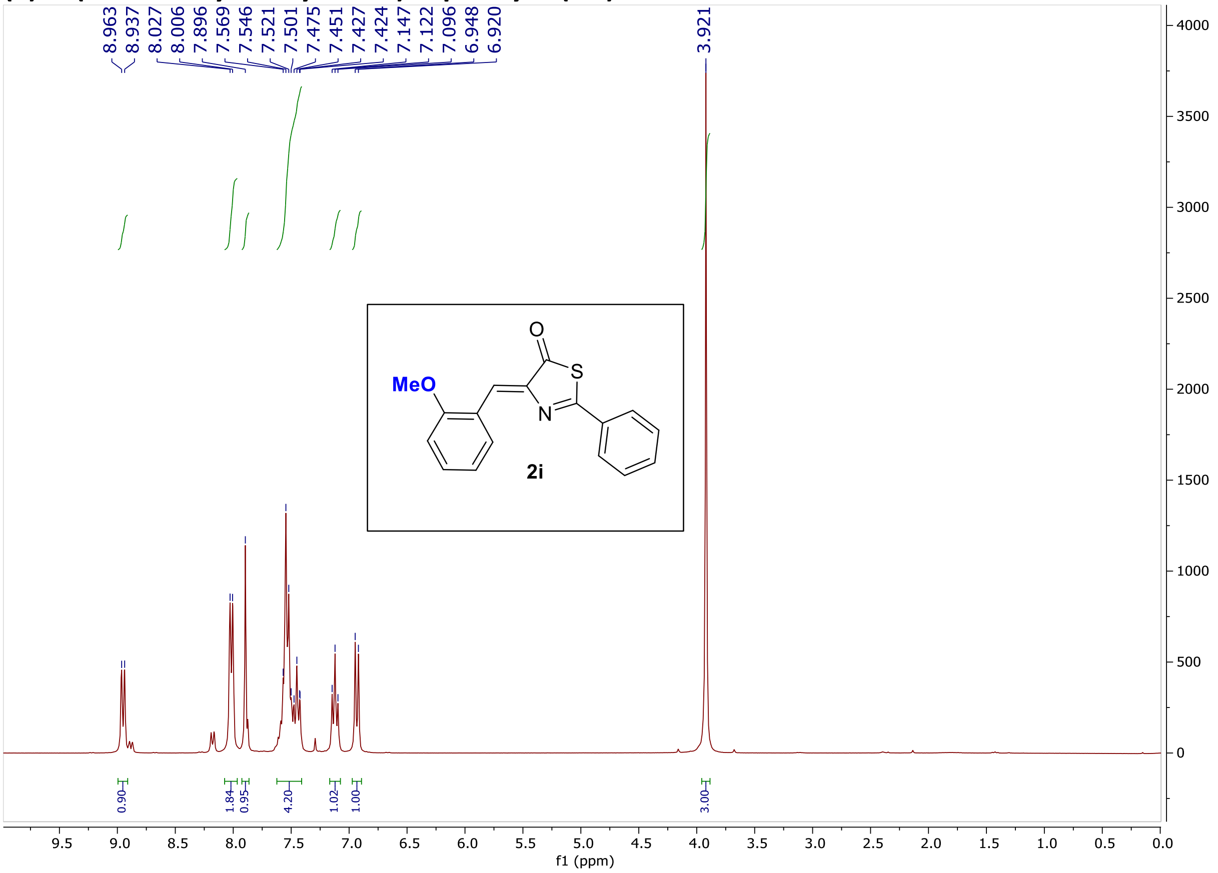

^{1}H NMR spectrum ($CDCl_3$, 300.13 MHz) of **2i**

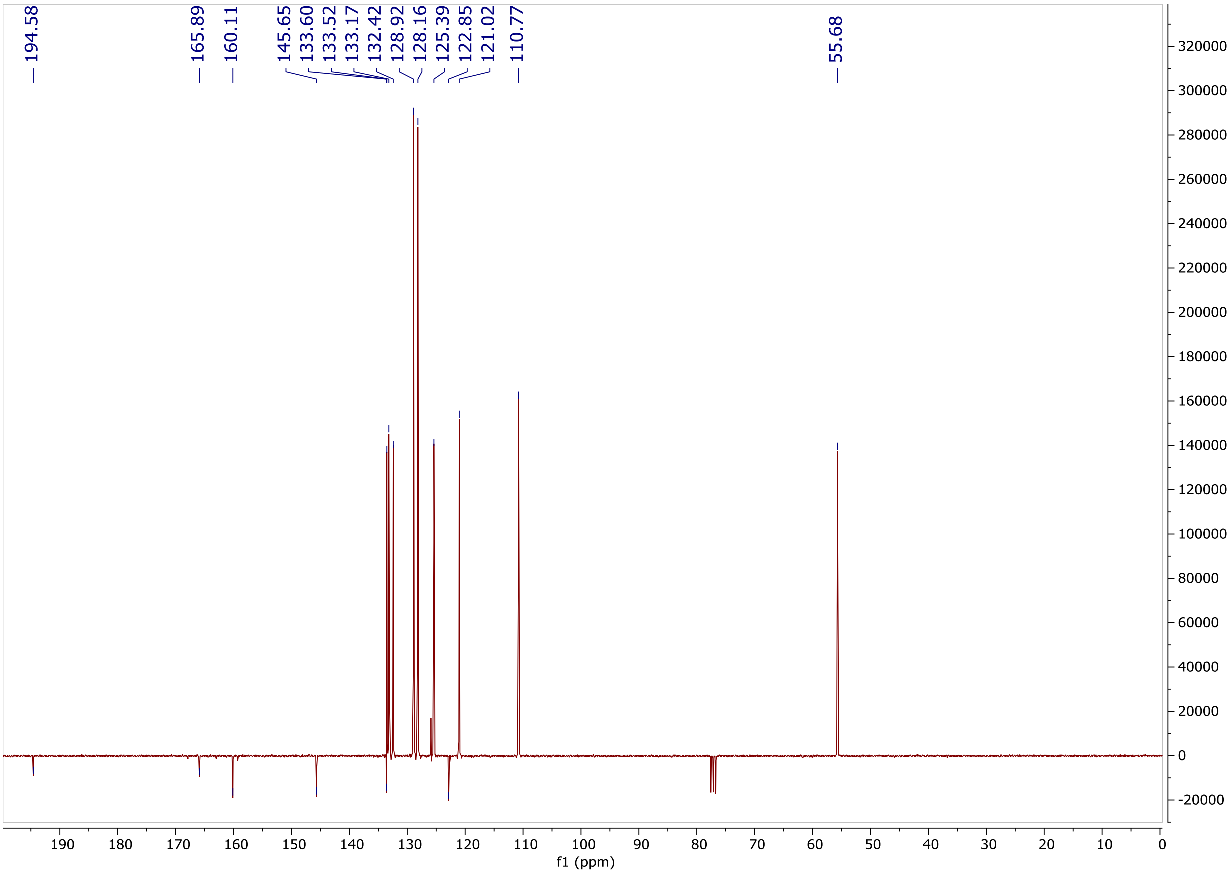

$^{13}C\{^{1}H\}$ NMR (APT) spectrum ($CDCl_3$, 75.5 MHz) of **2i**

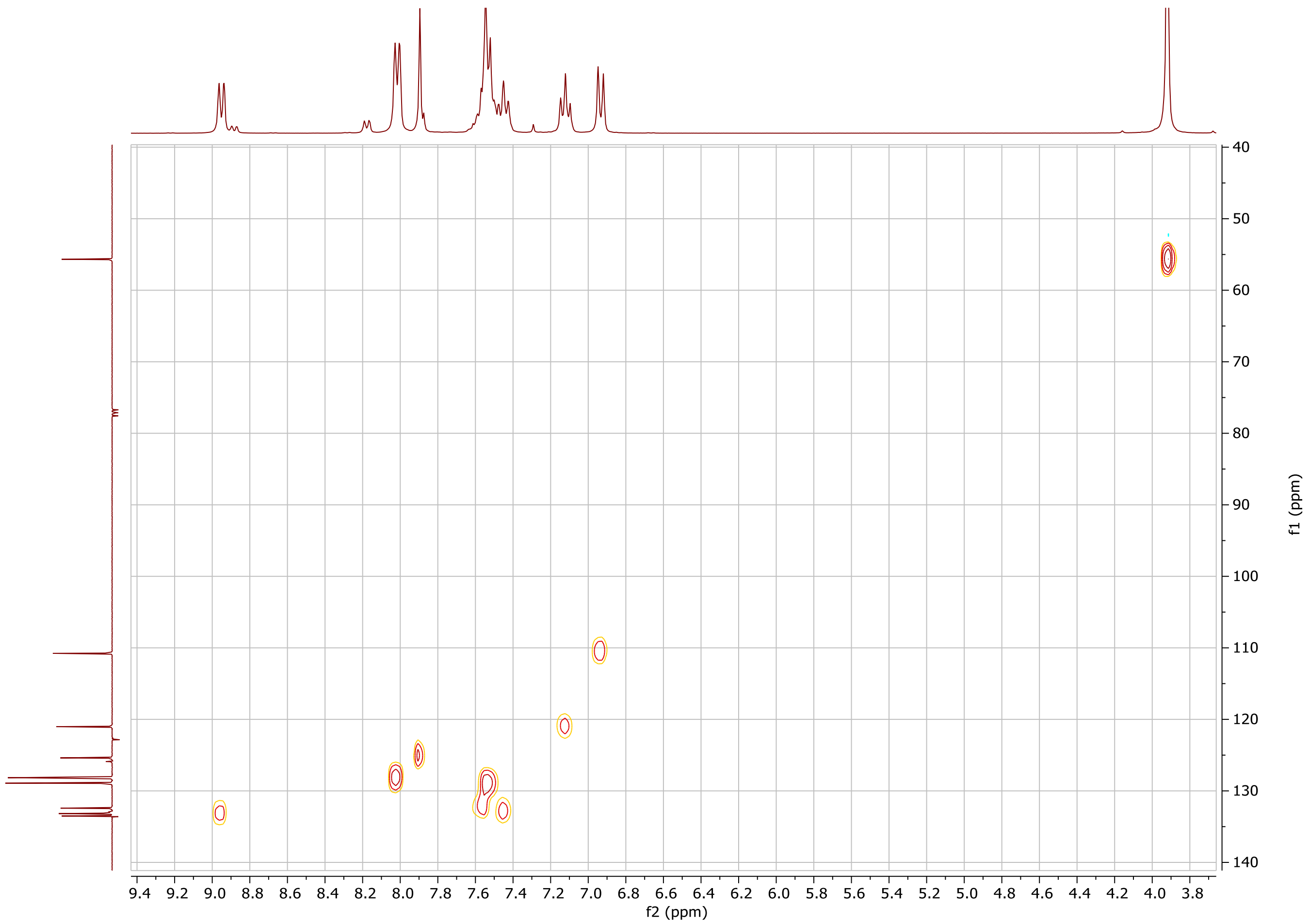

^{1}H-^{13}C HSQC correlation spectrum of **2i**

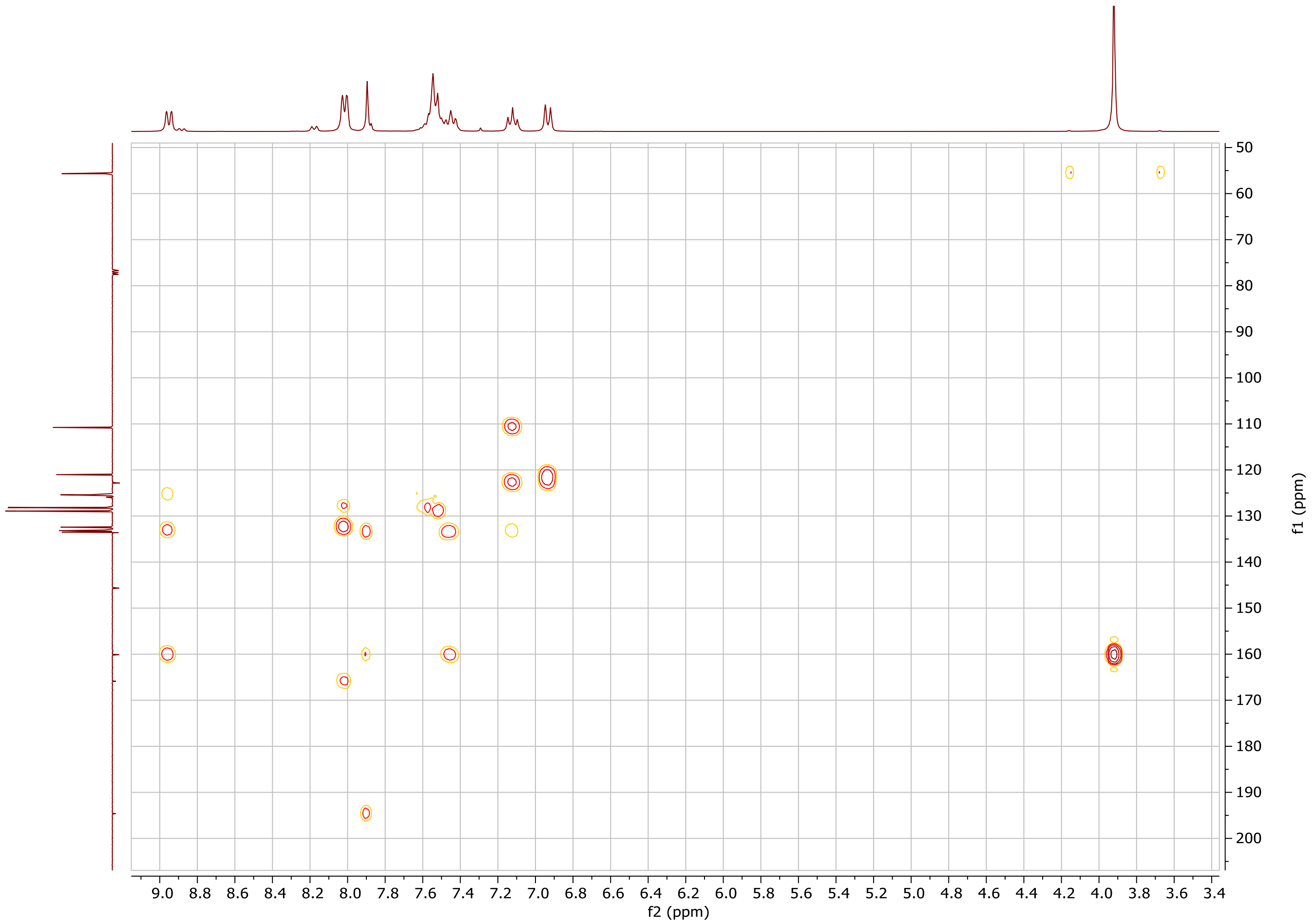

^{1}H-^{13}C HMBC correlation spectrum of **2i**

(*Z*)-4-(2-chlorobenzylidene)-2-phenyl-5(4*H*)-thiazolone 2j

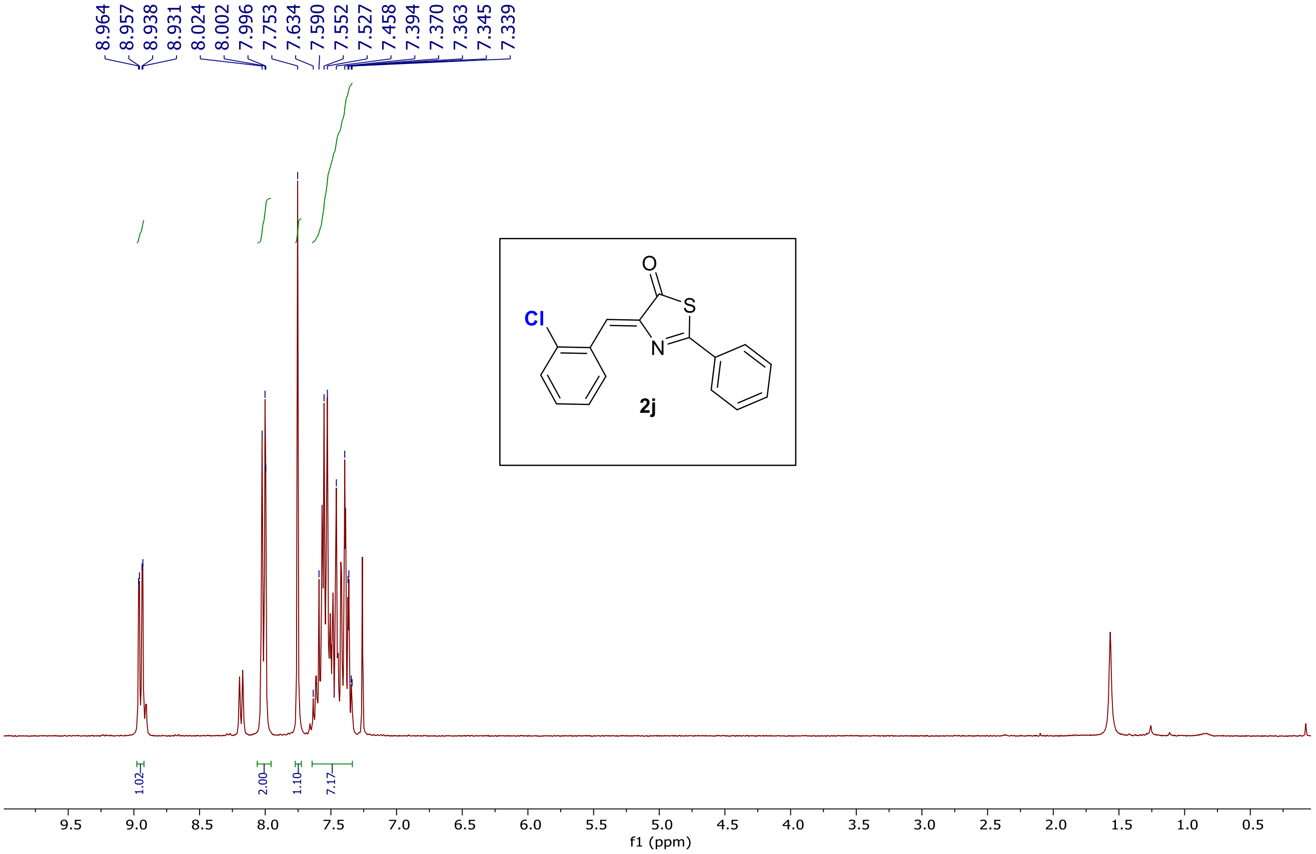

^{1}H NMR spectrum ($CDCl_3$, 300.13 MHz) of **2j**

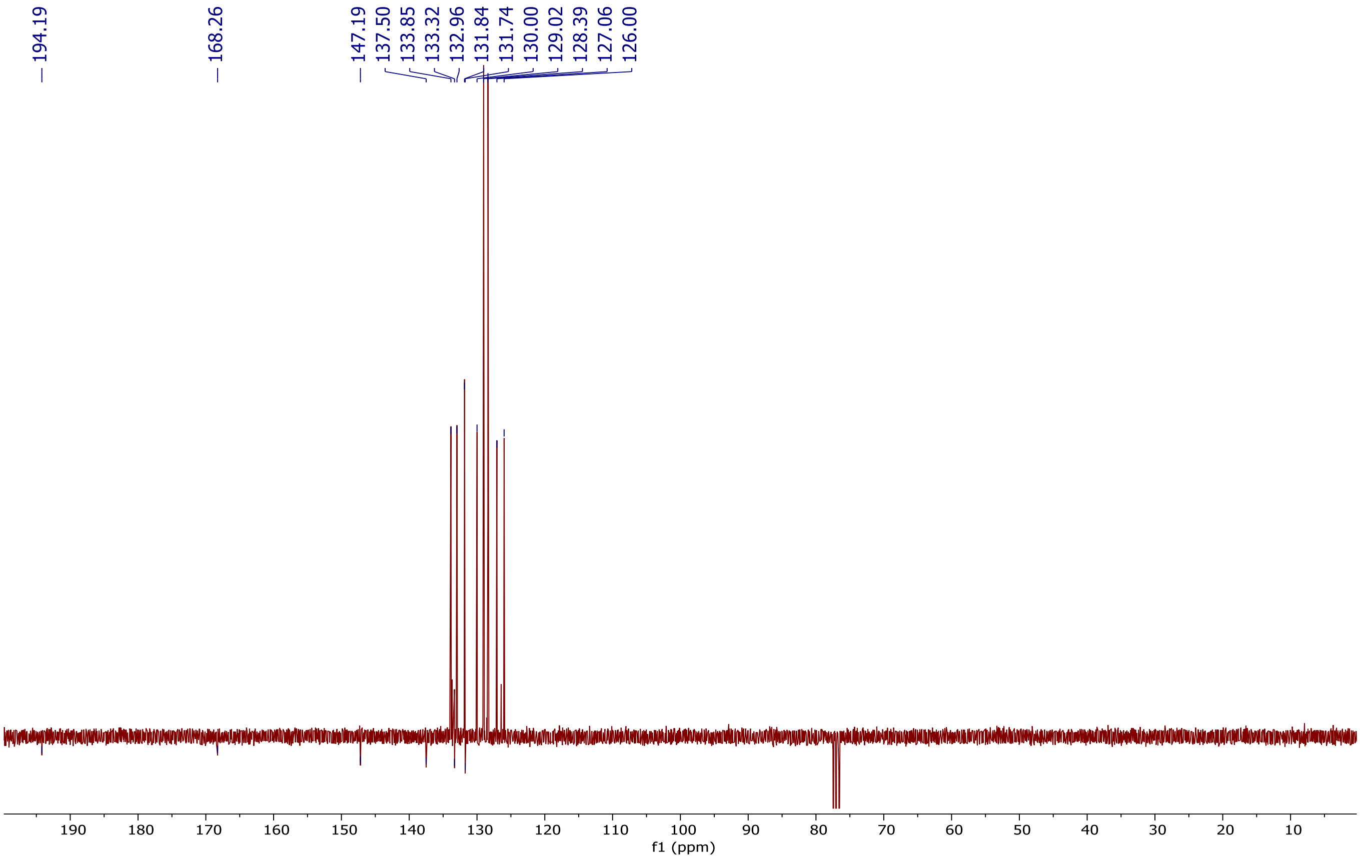

^{13}C{^{1}H} NMR (APT) spectrum ($CDCl_3$, 75.5 MHz) of **2j**

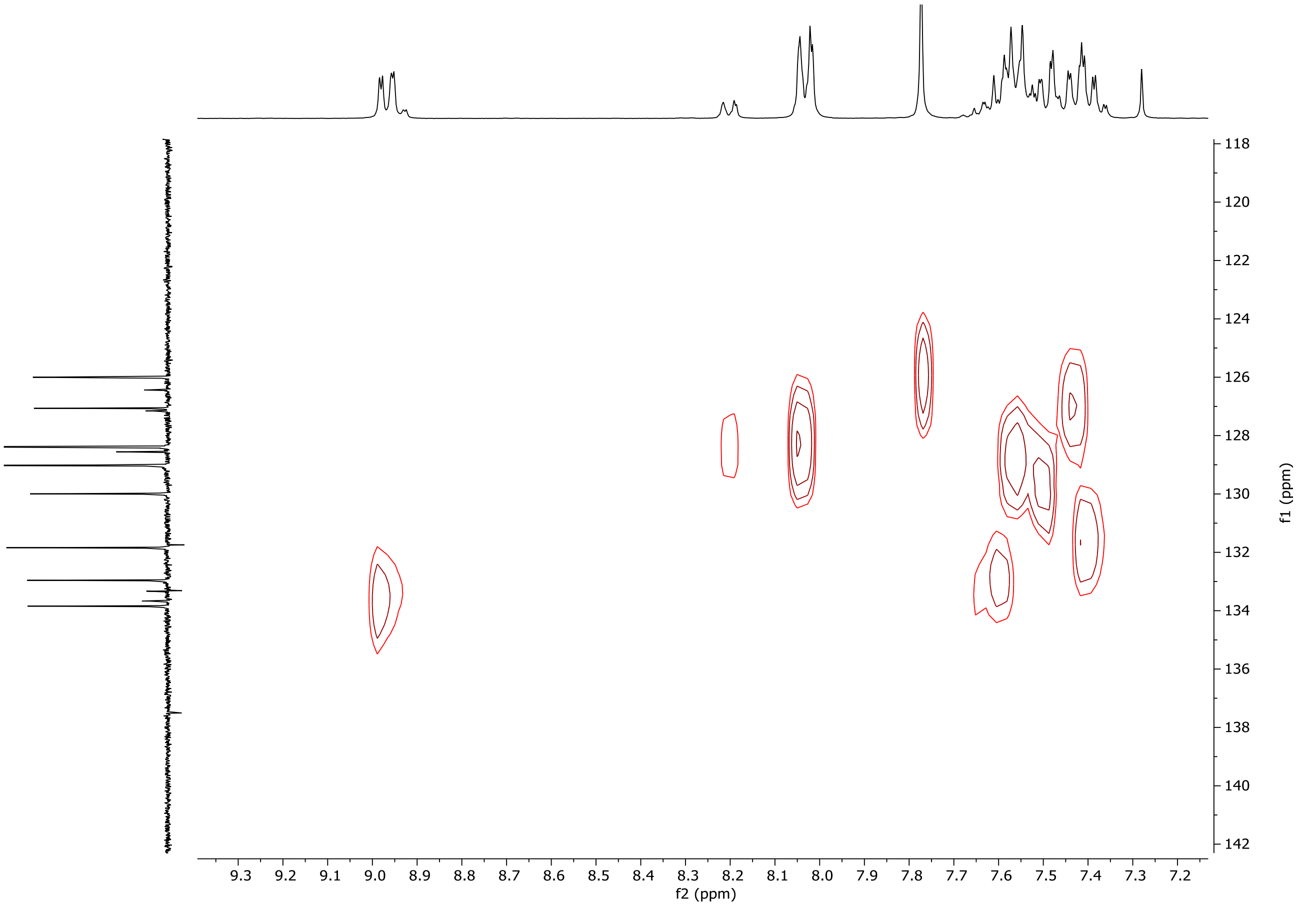

^{1}H-^{13}C HSQC correlation spectrum of **2j**

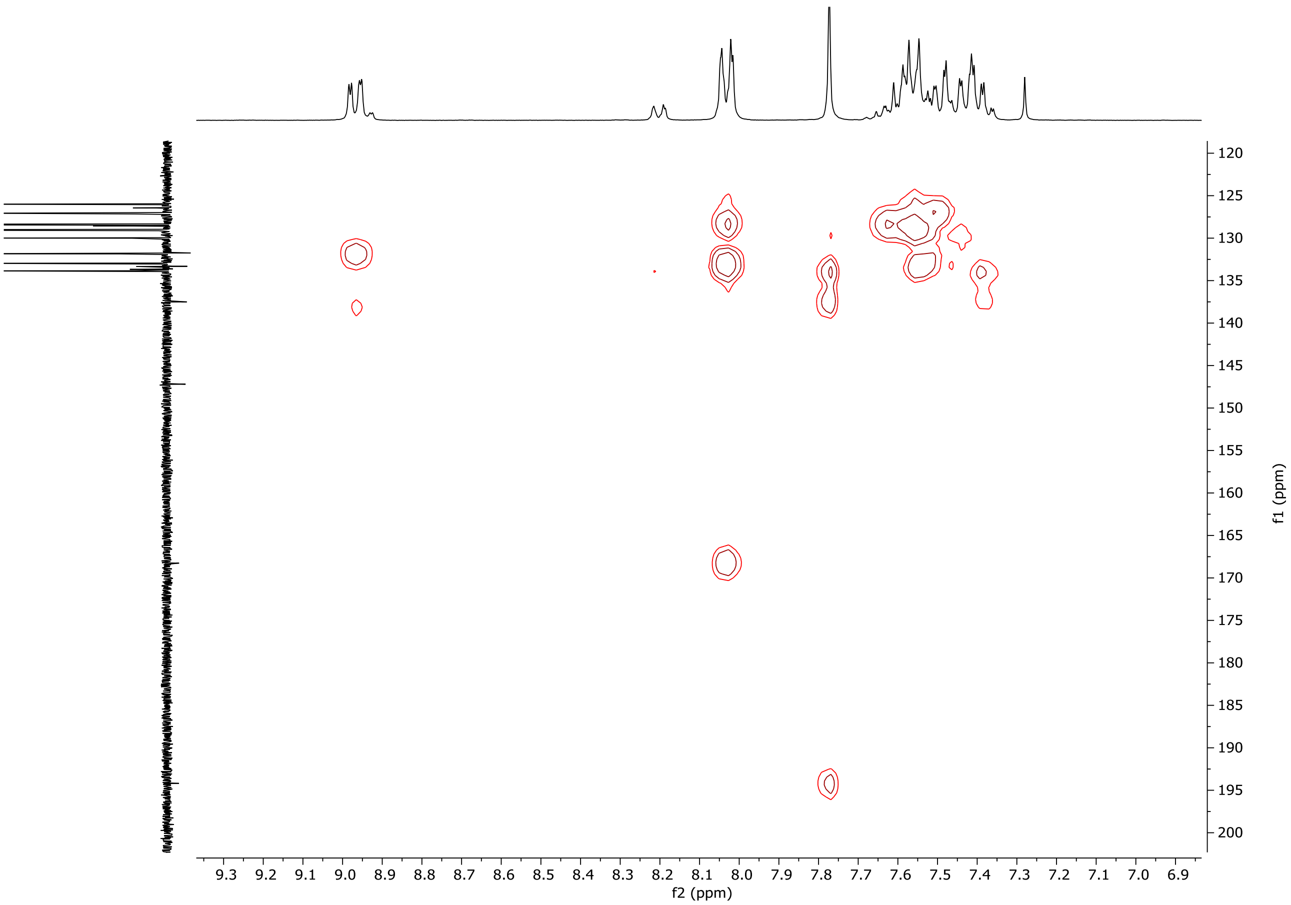

^{1}H-^{13}C HMBC correlation spectrum of **2j**

(*Z*)-4-(2-bromobenzylidene)-2-phenyl-5(4*H*)-thiazolone 2k

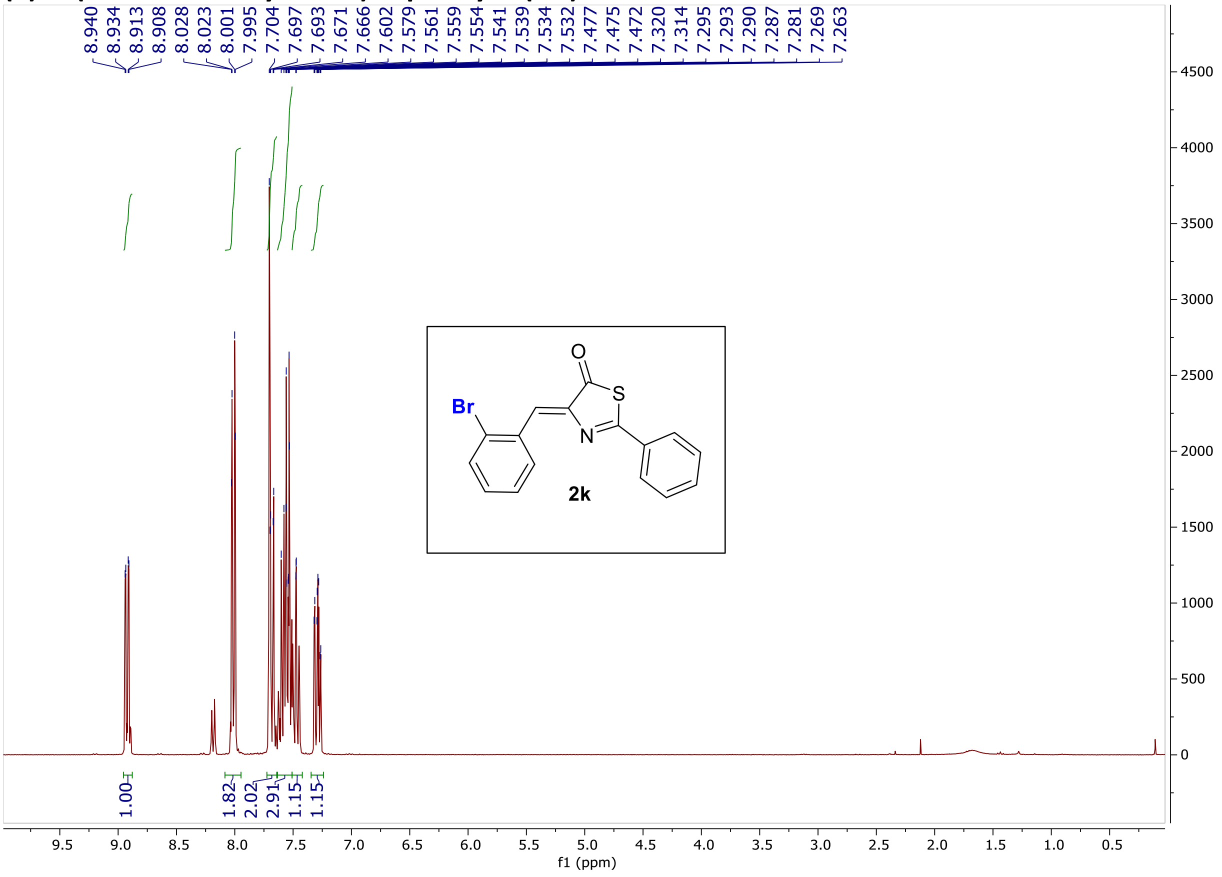

^{1}H NMR spectrum ($CDCl_3$, 300.13 MHz) of **2k**

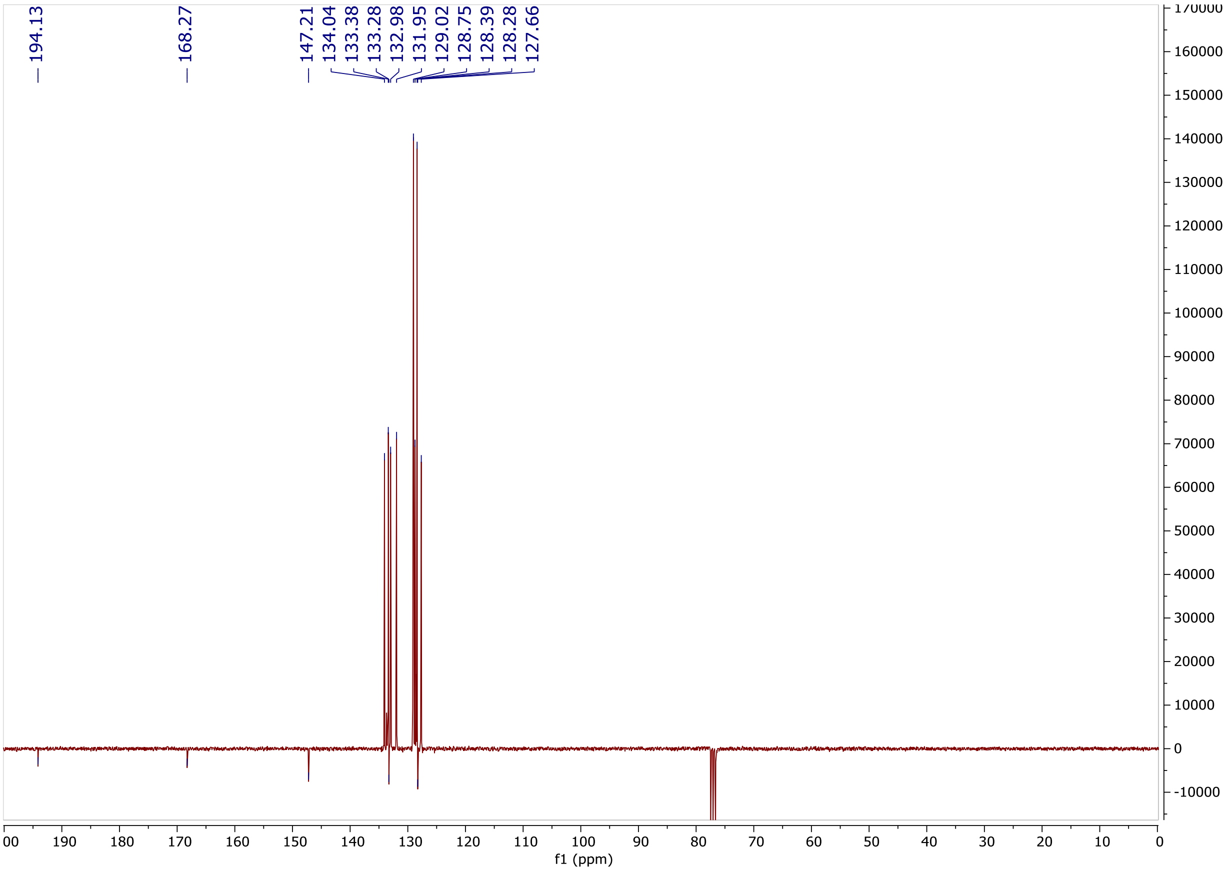

^{13}C{^{1}H} NMR (APT) spectrum ($CDCl_3$, 75.5 MHz) of **2k**

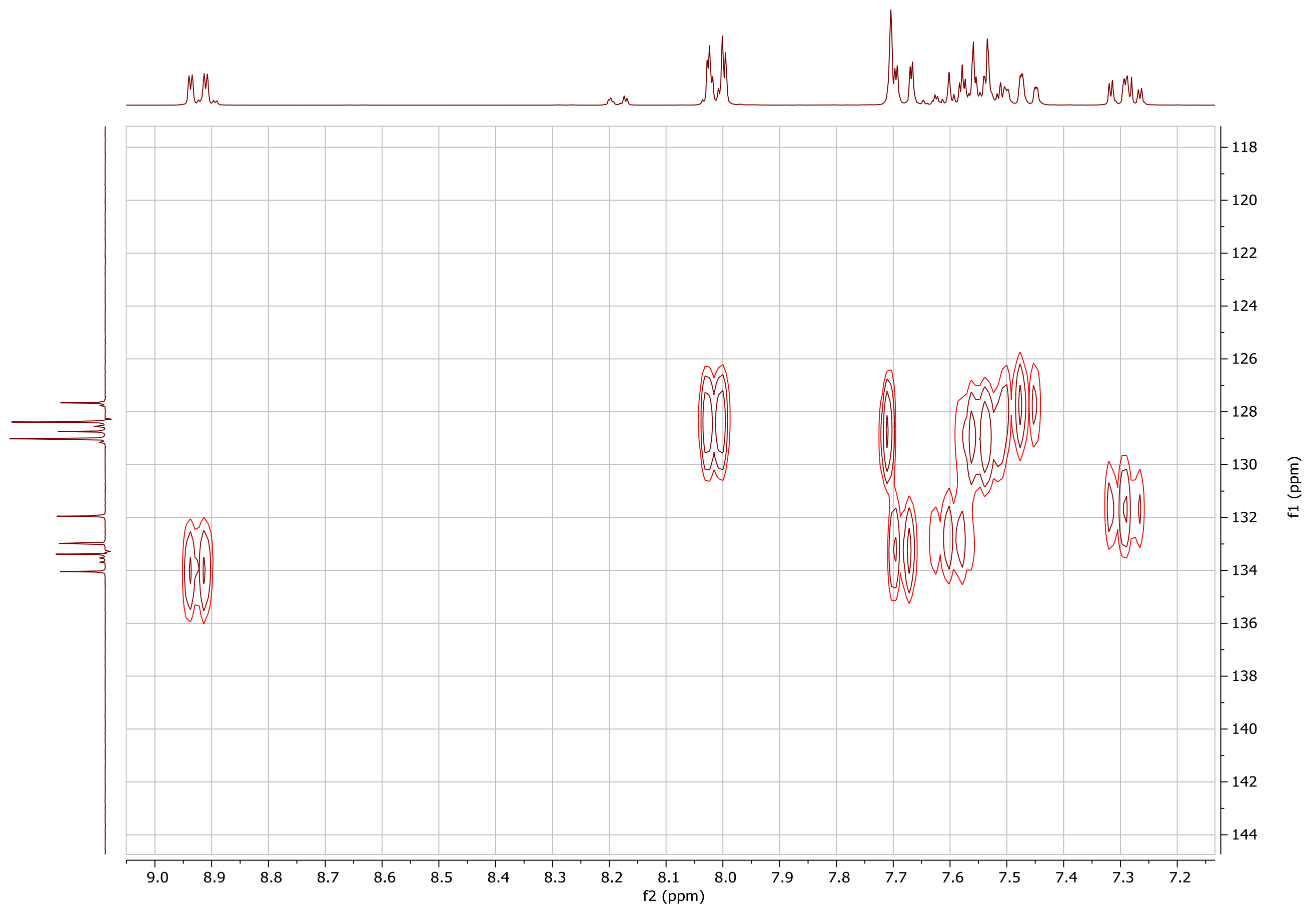

^{1}H-^{13}C HSQC correlation spectrum of **2k**

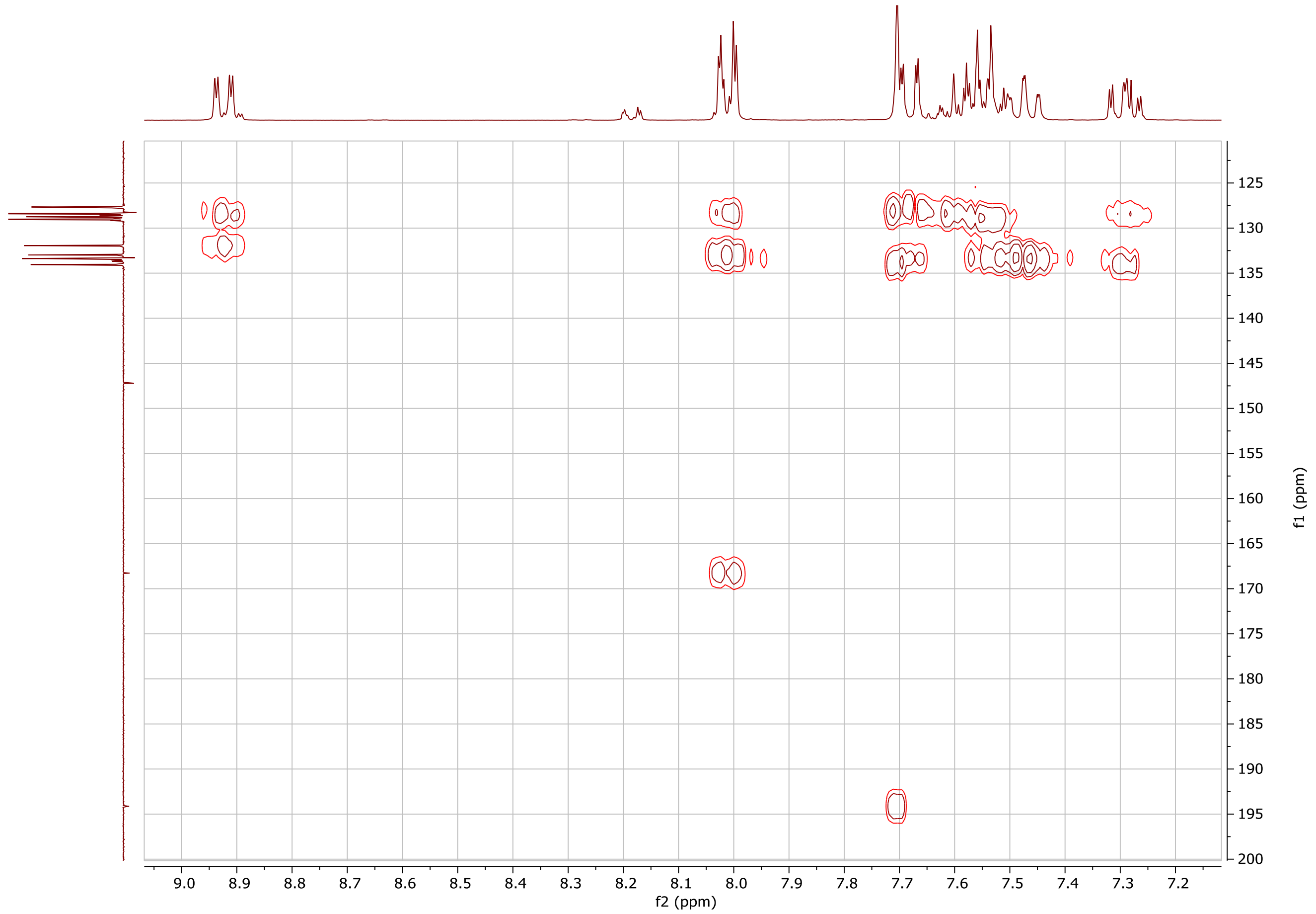

^{1}H-^{13}C HMBC correlation spectrum of **2k**

(*Z*)-4-(3,4-dimethoxybenzylidene)-2-phenyl-5(4*H*)-thiazolone 2l

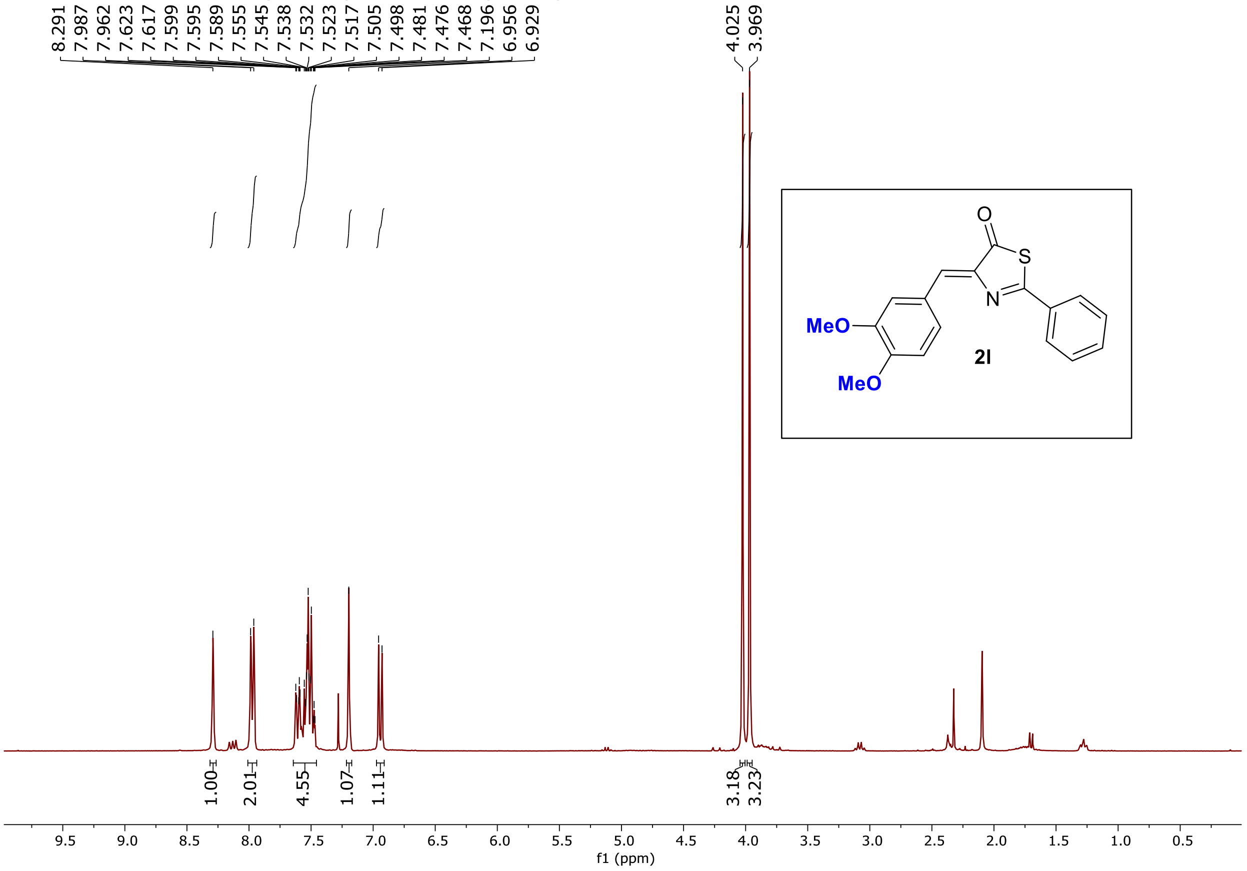

^{1}H NMR spectrum ($CDCl_3$, 300.13 MHz) of **2l**

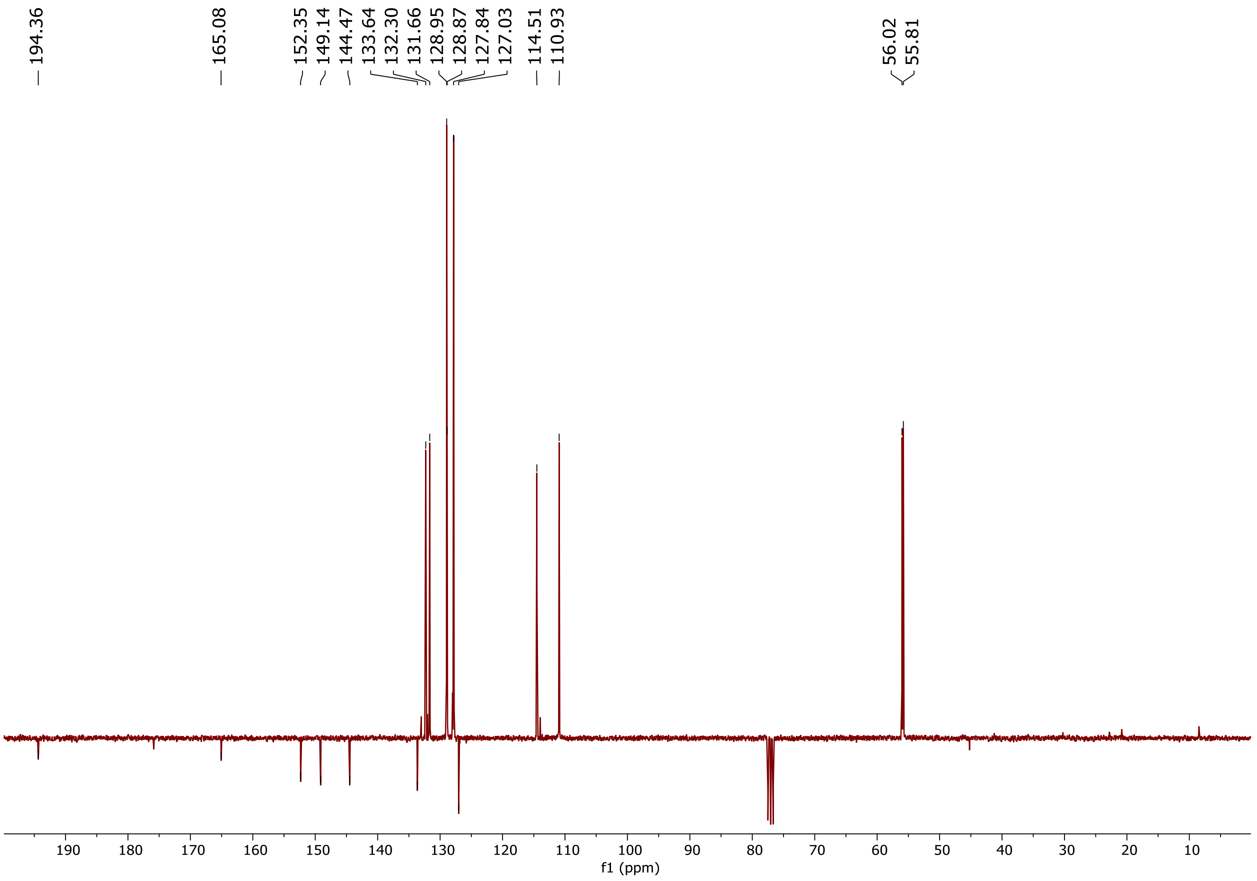

$^{13}C\{^{1}H\}$ NMR (APT) spectrum ($CDCl_3$, 75.5 MHz) of **2l**

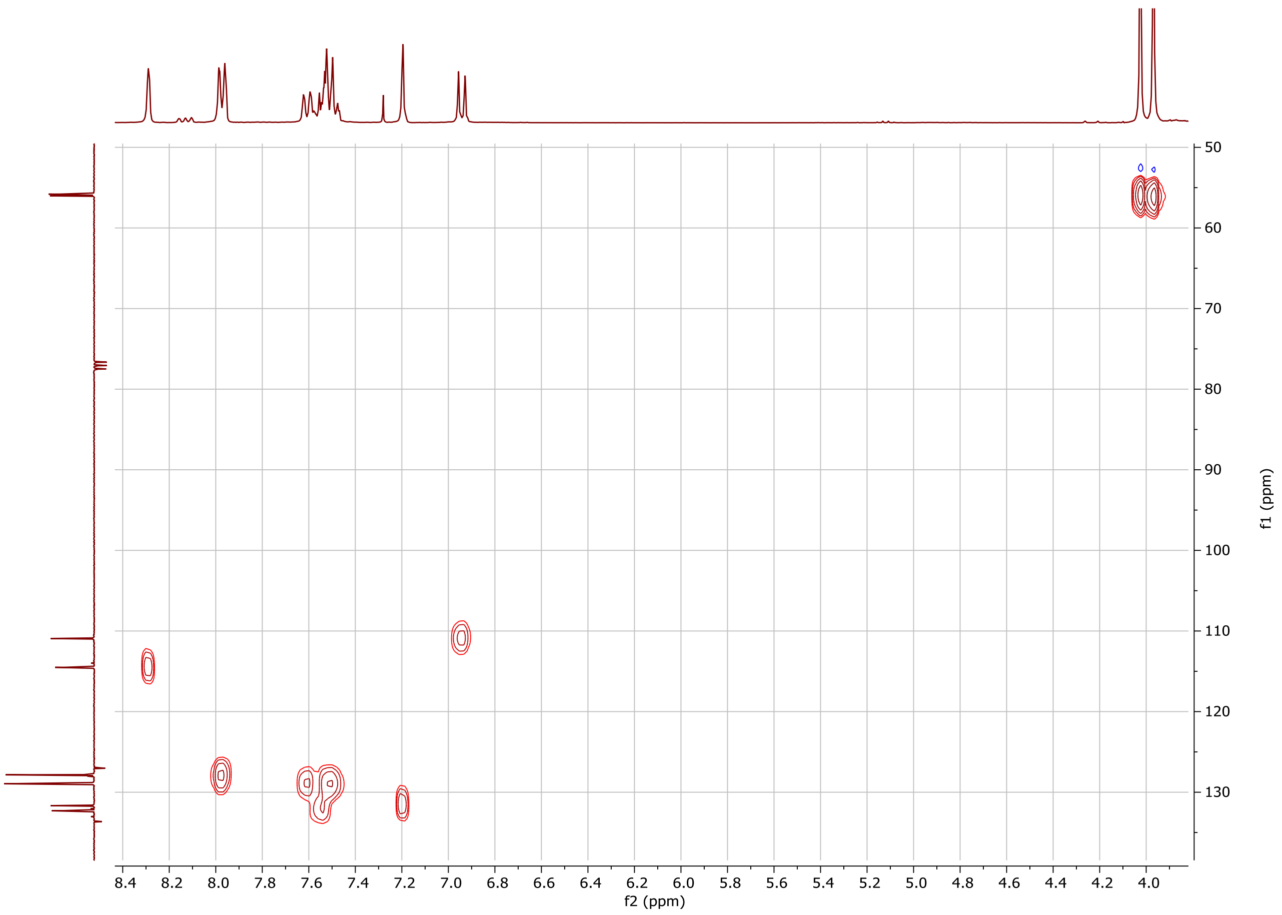

^{1}H-^{13}C HSQC correlation spectrum of **2l**

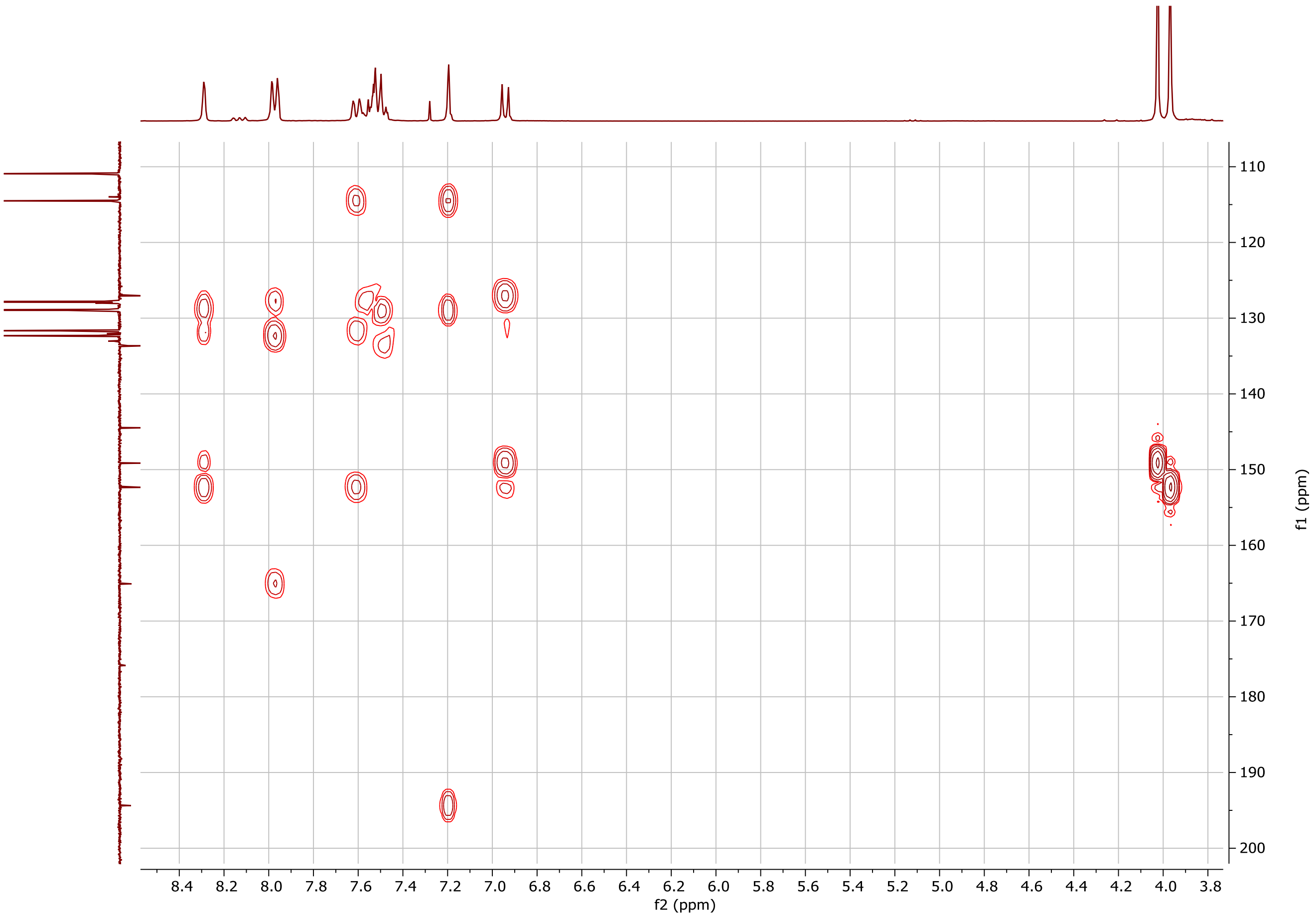

^{1}H-^{13}C HMBC correlation spectrum of **2l**

(*Z*)-4-(3,4-dimethylbenzylidene)-2-phenyl-5(4*H*)-thiazolone 2m

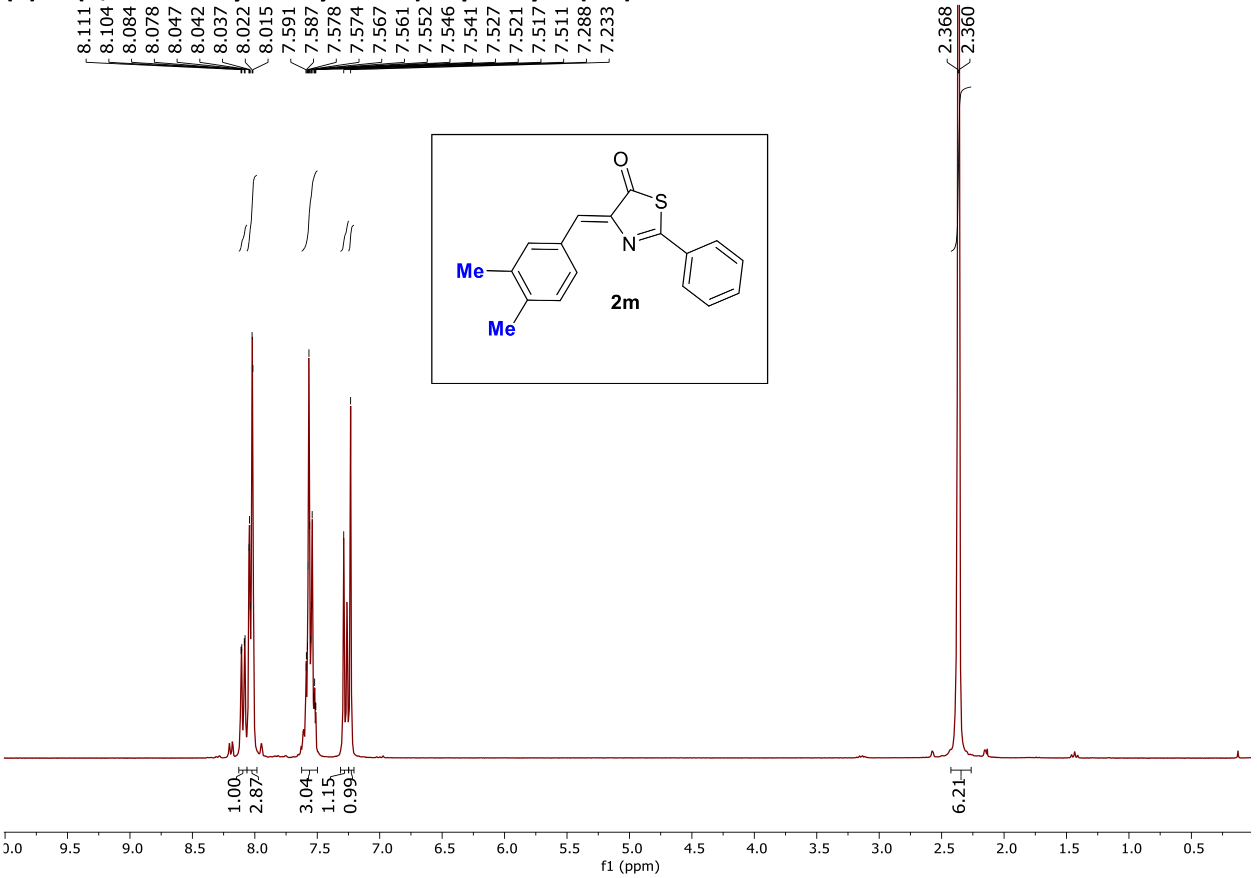

^{1}H NMR spectrum ($CDCl_3$, 300.13 MHz) of **2m**

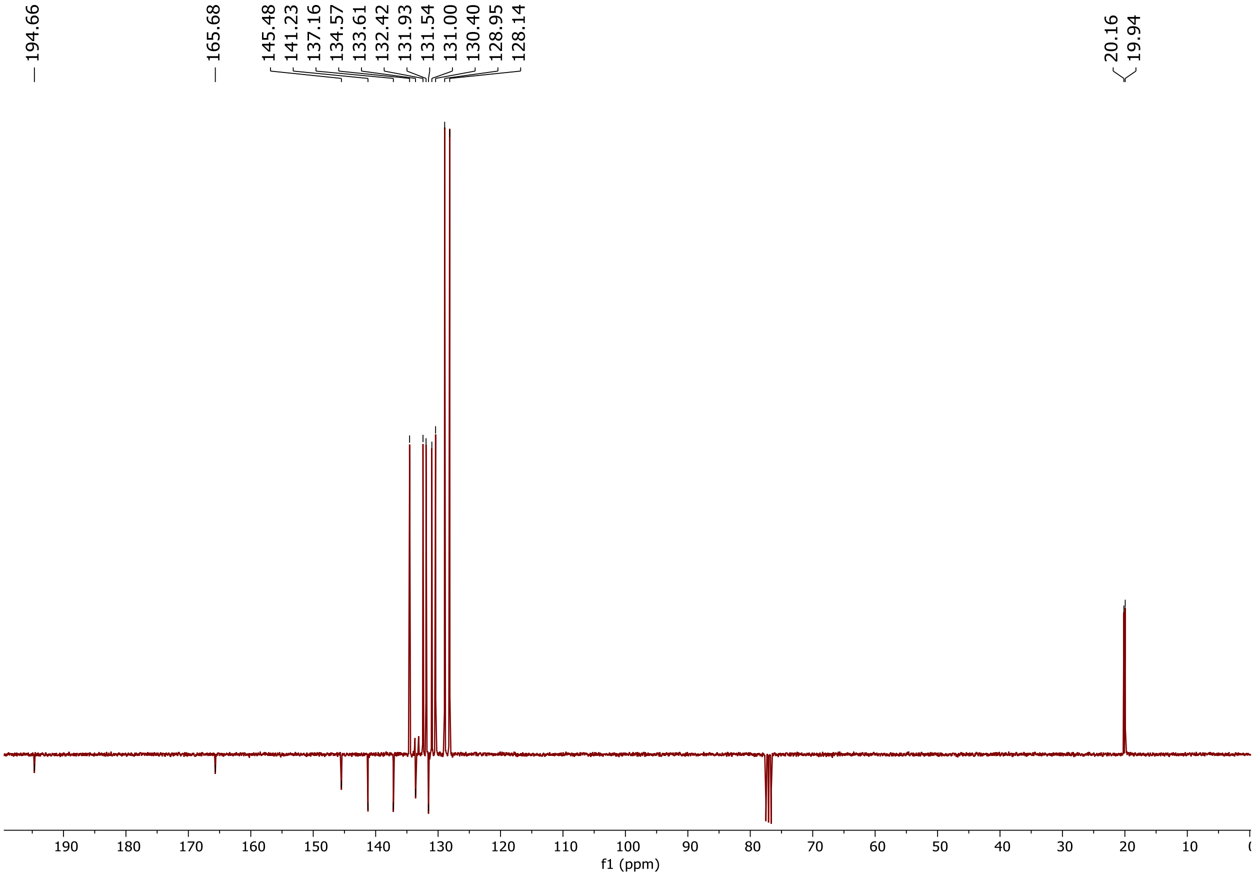

^{13}C{^{1}H} NMR (APT) spectrum ($CDCl_3$, 75.5 MHz) of **2m**

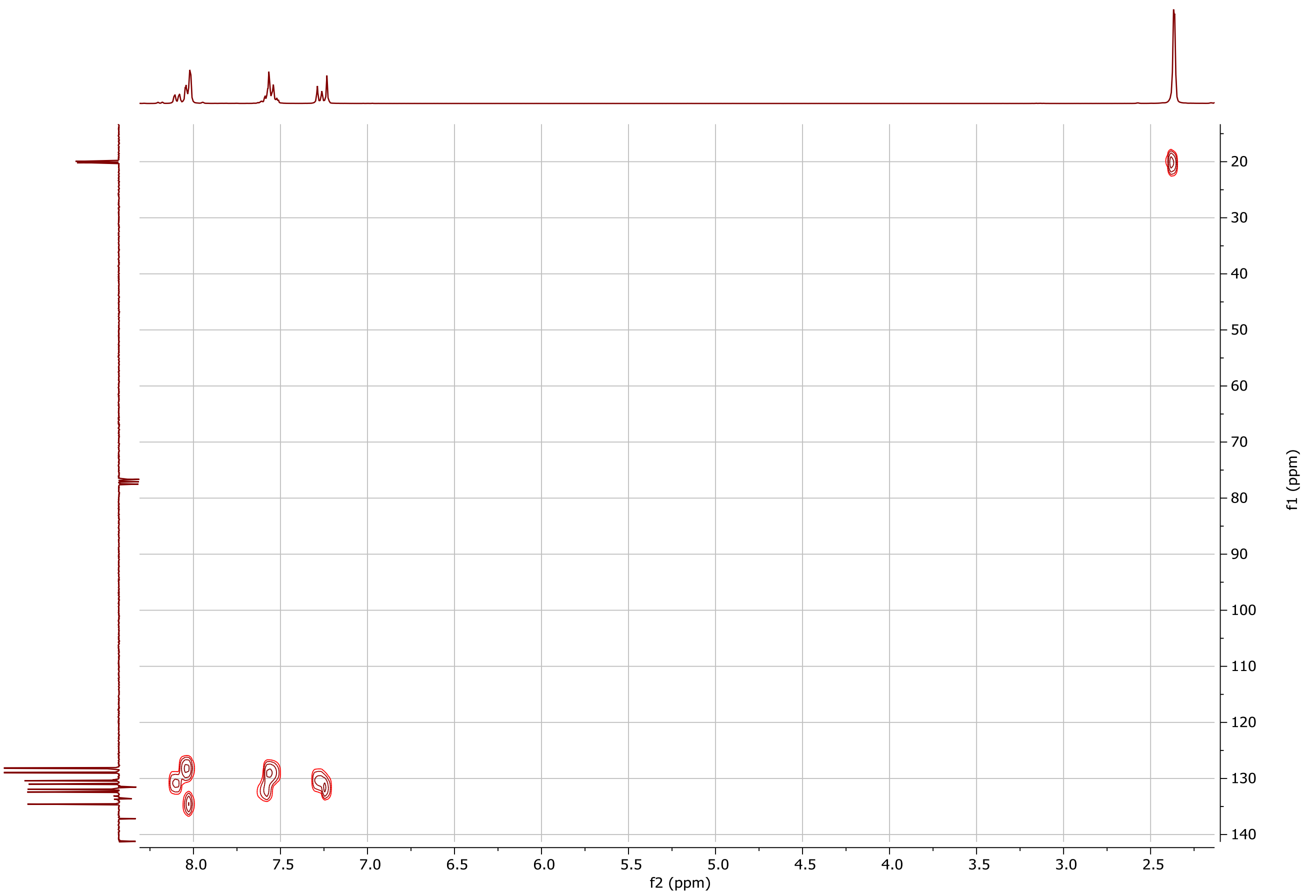

^{1}H-^{13}C HSQC correlation spectrum of **2m**

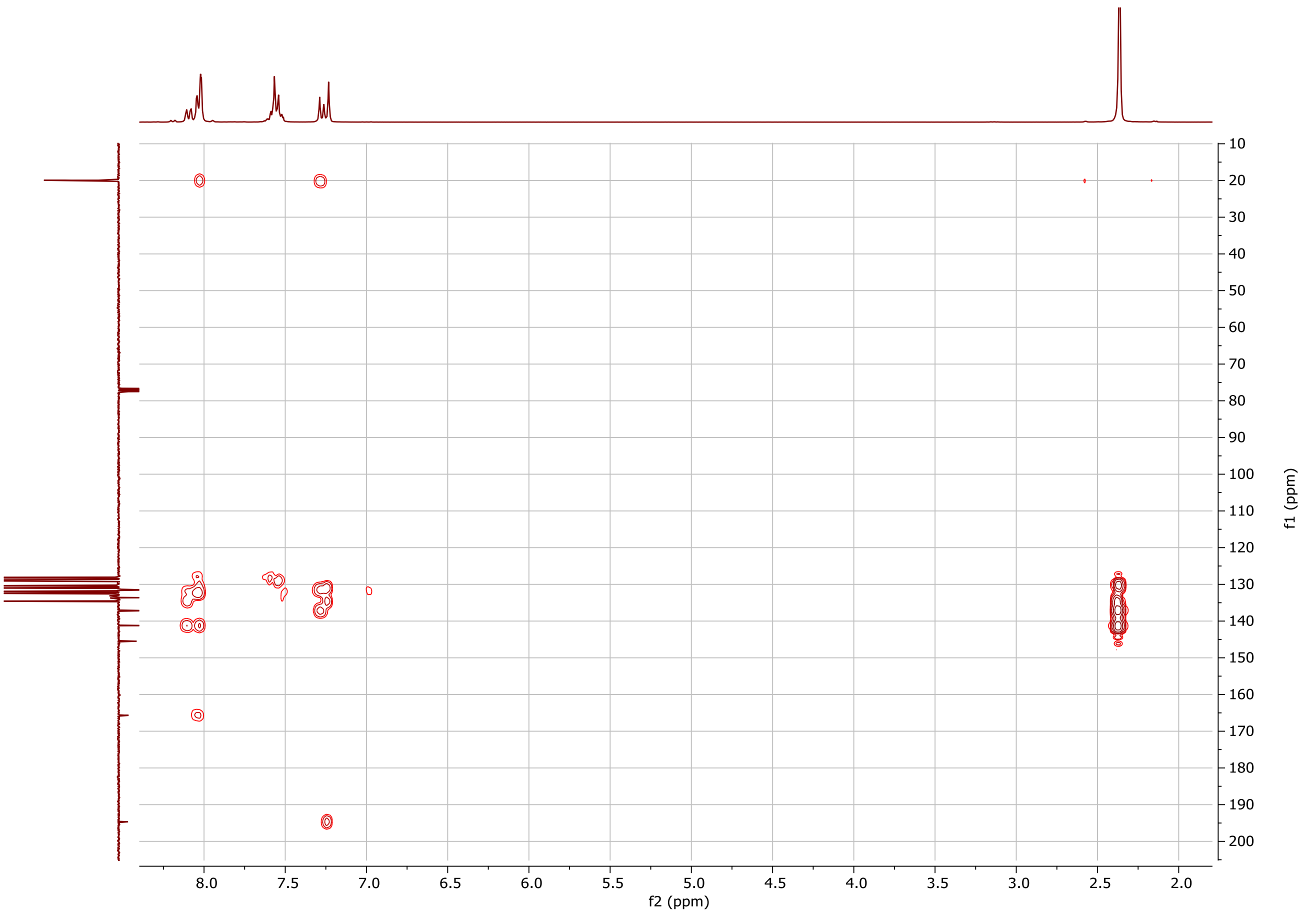

^{1}H-^{13}C HMBC correlation spectrum of **2m**

(*Z*)-4-(3,4-dichlorobenzylidene)-2-phenyl-5(4*H*)-thiazolone 2n

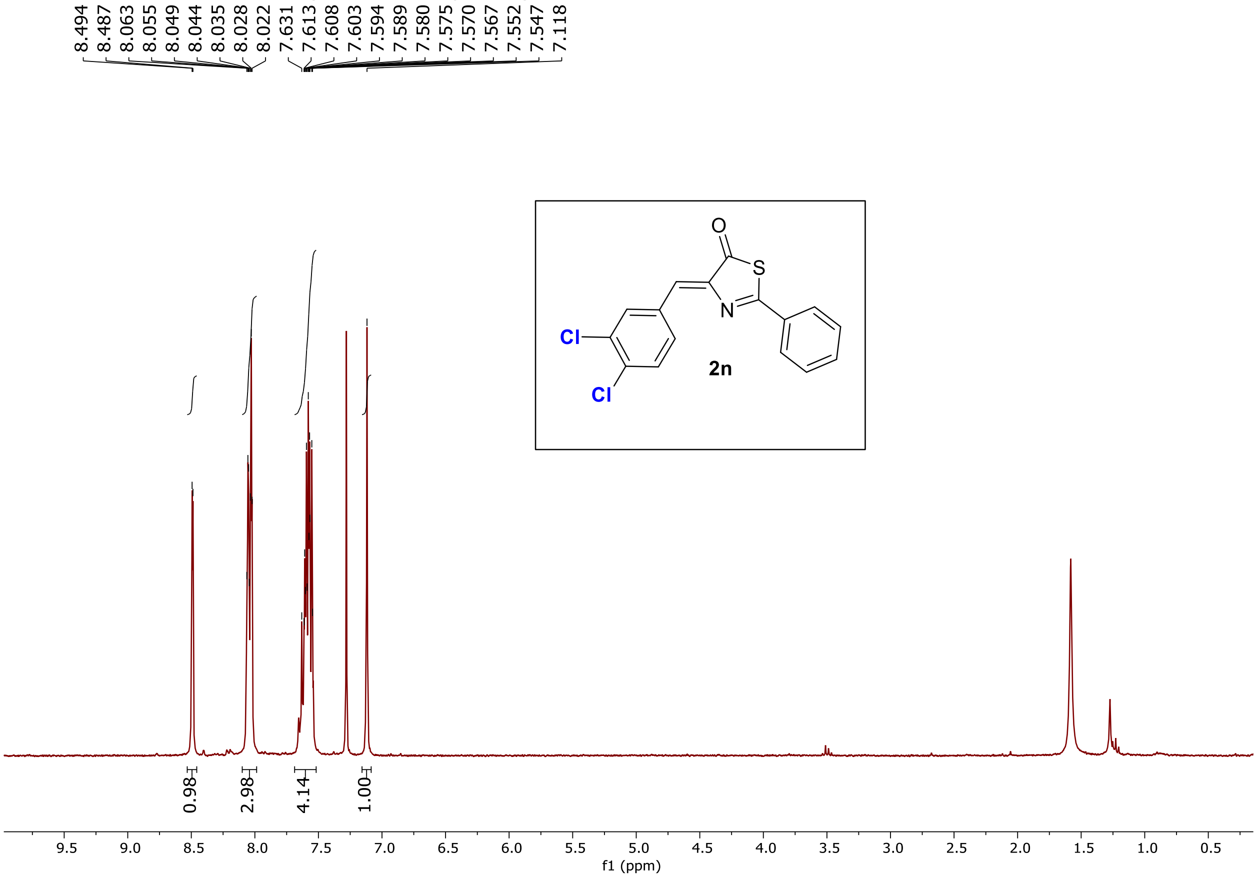

^{1}H NMR spectrum ($CDCl_3$, 300.13 MHz) of **2n**

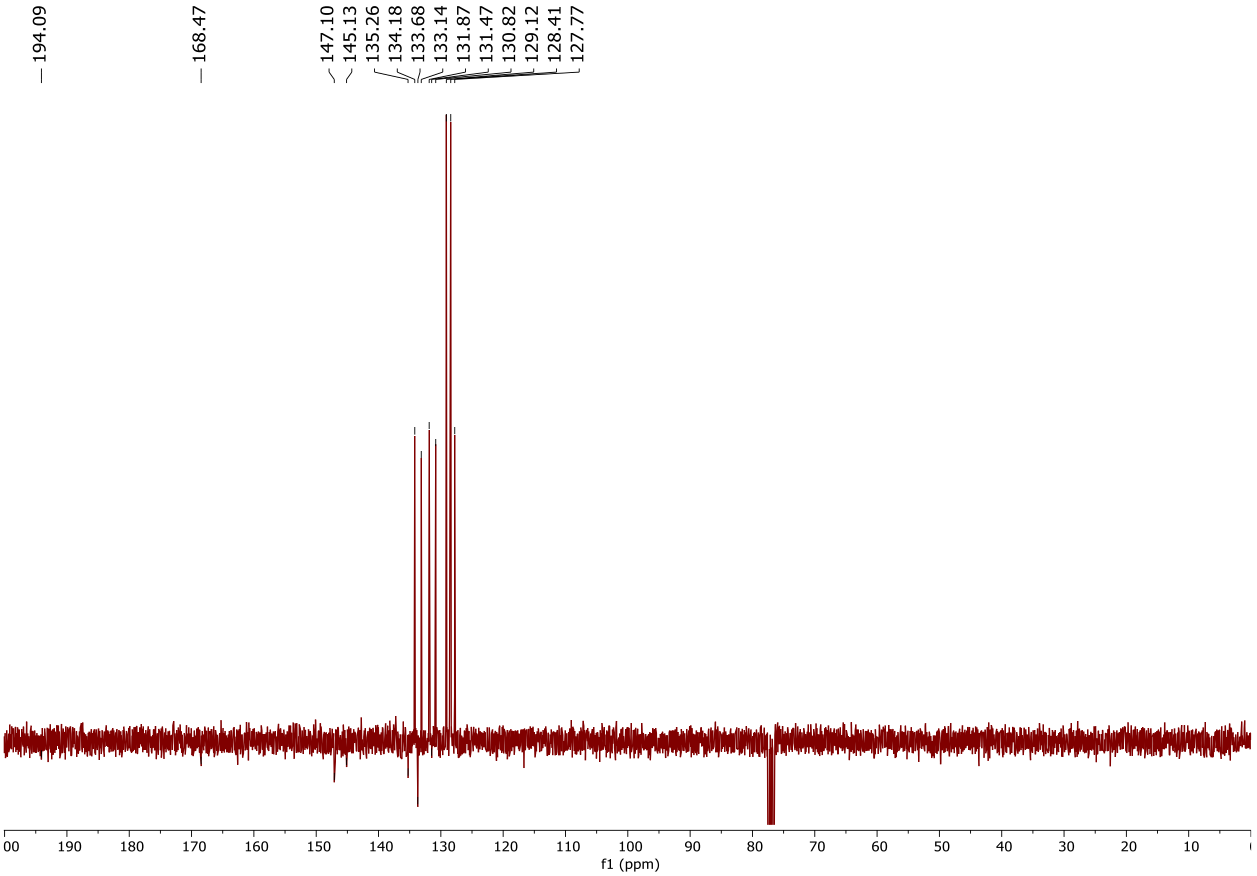

^{13}C{^{1}H} NMR (APT) spectrum (CD_2Cl_2, 75.5 MHz) of **2n**

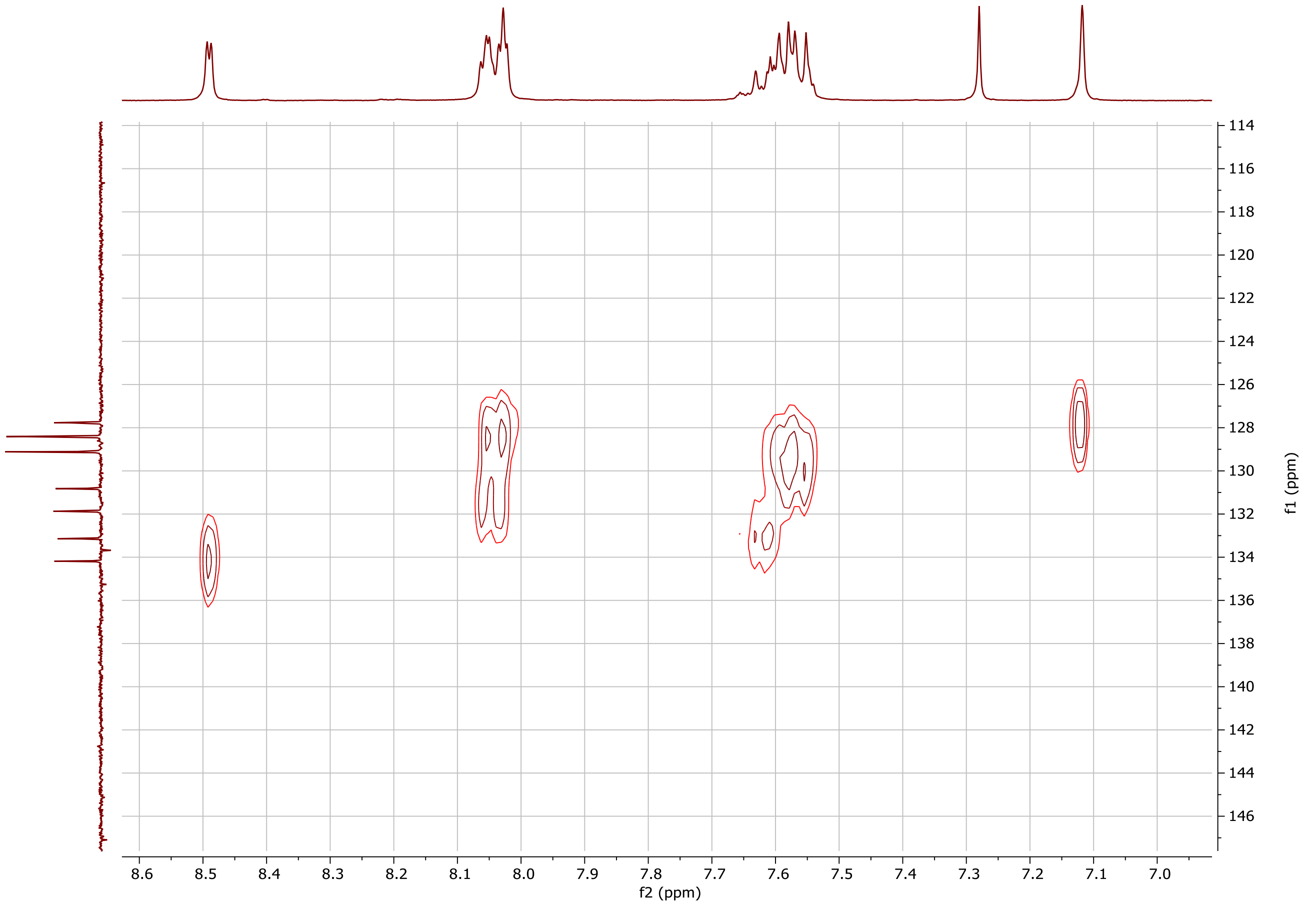

^{1}H-^{13}C HSQC correlation spectrum of **2n**

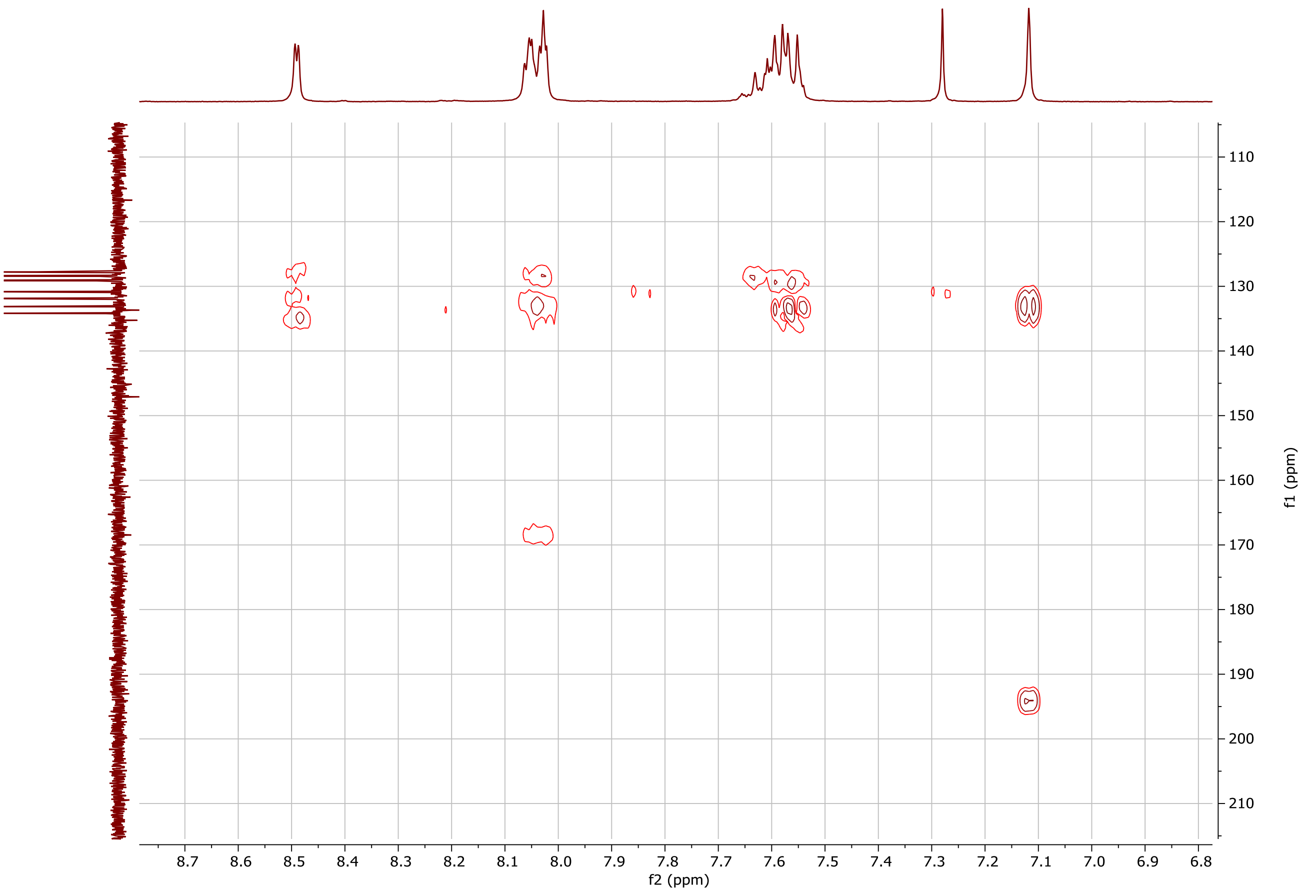

^{1}H-^{13}C HMBC correlation spectrum of **2n**

(*Z*)-4-(3,4-difluorobenzylidene)-2-phenyl-5(4*H*)-thiazolone 2o

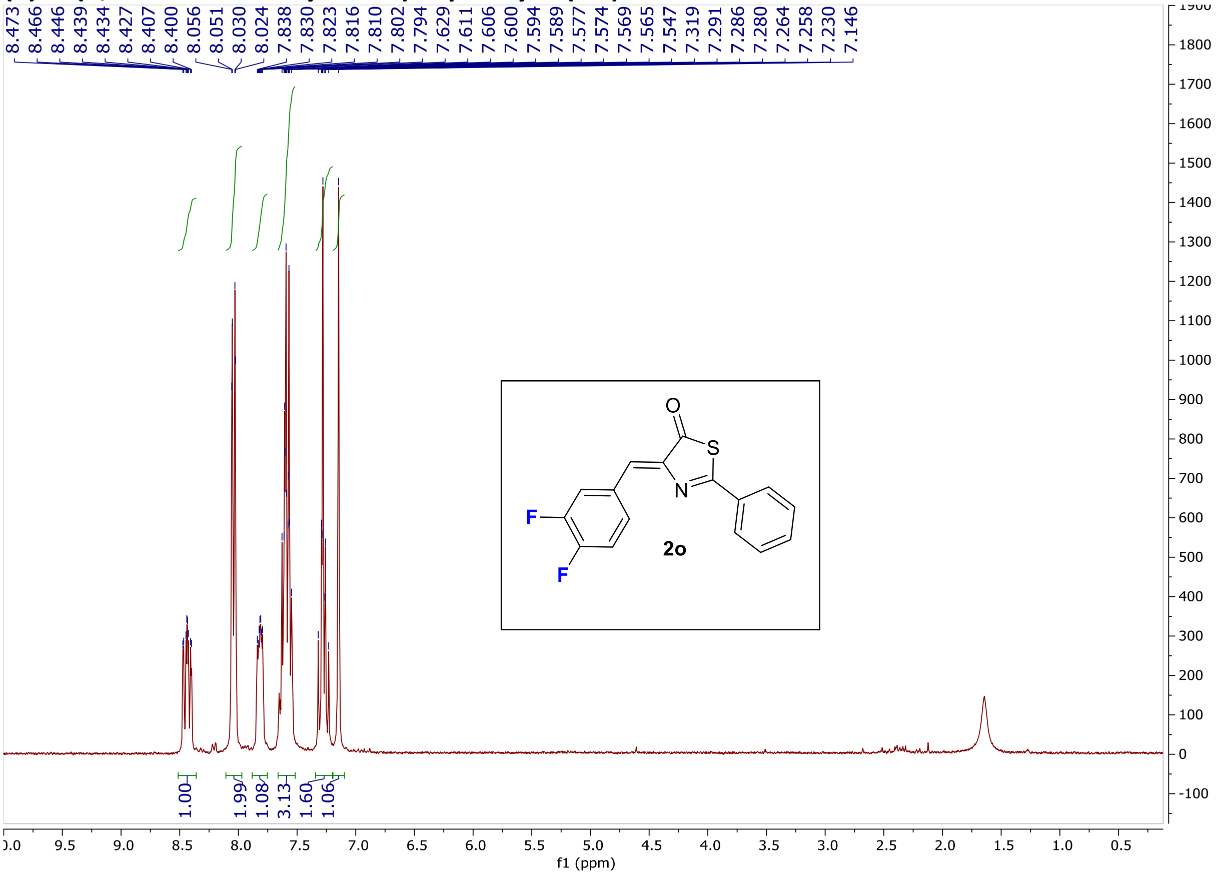

^{1}H NMR spectrum ($CDCl_3$, 300.13 MHz) of **2o**

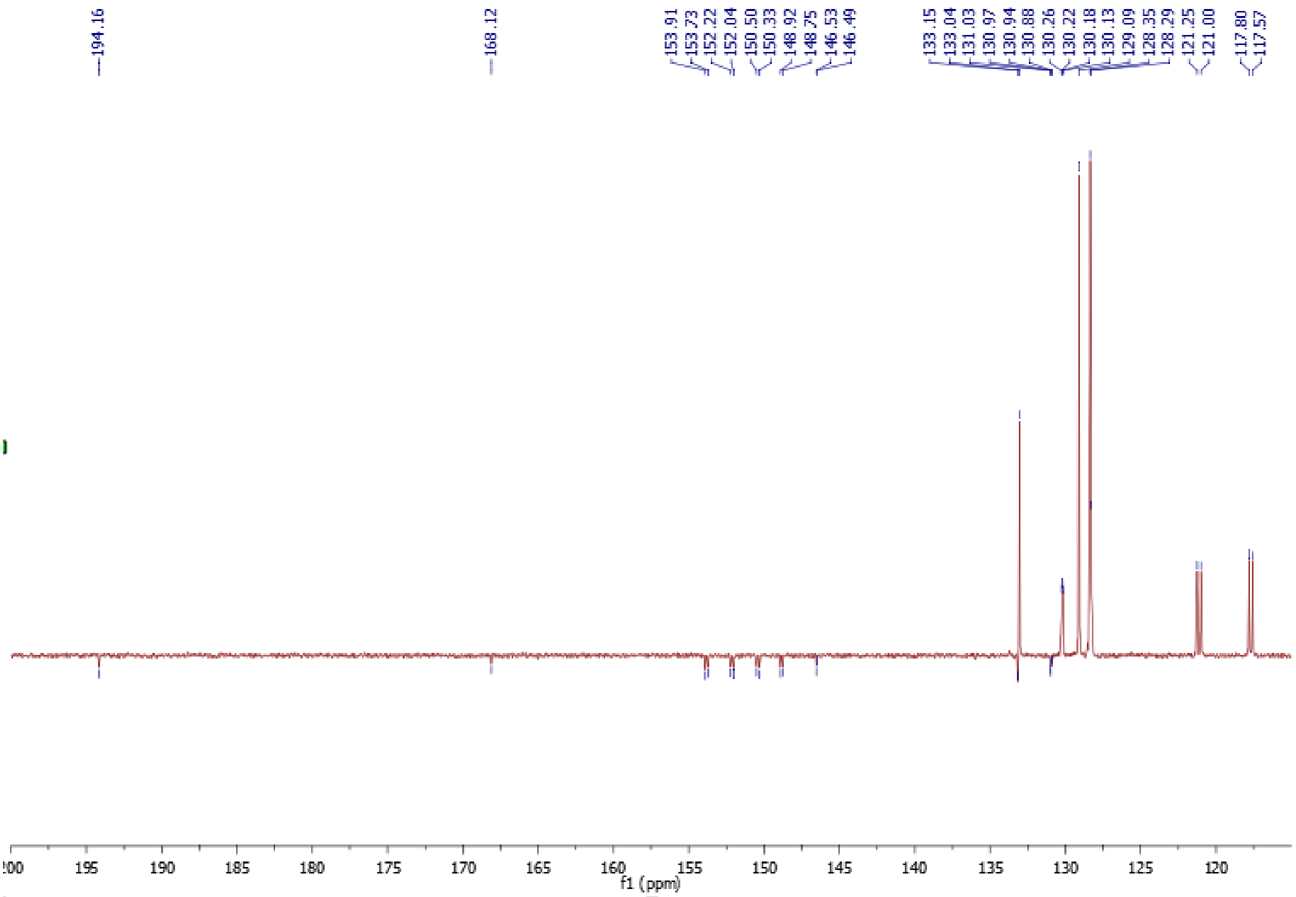

$^{13}C\{^{1}H\}$ NMR (APT) spectrum ($CDCl_3$, 75.5 MHz) of **2o**

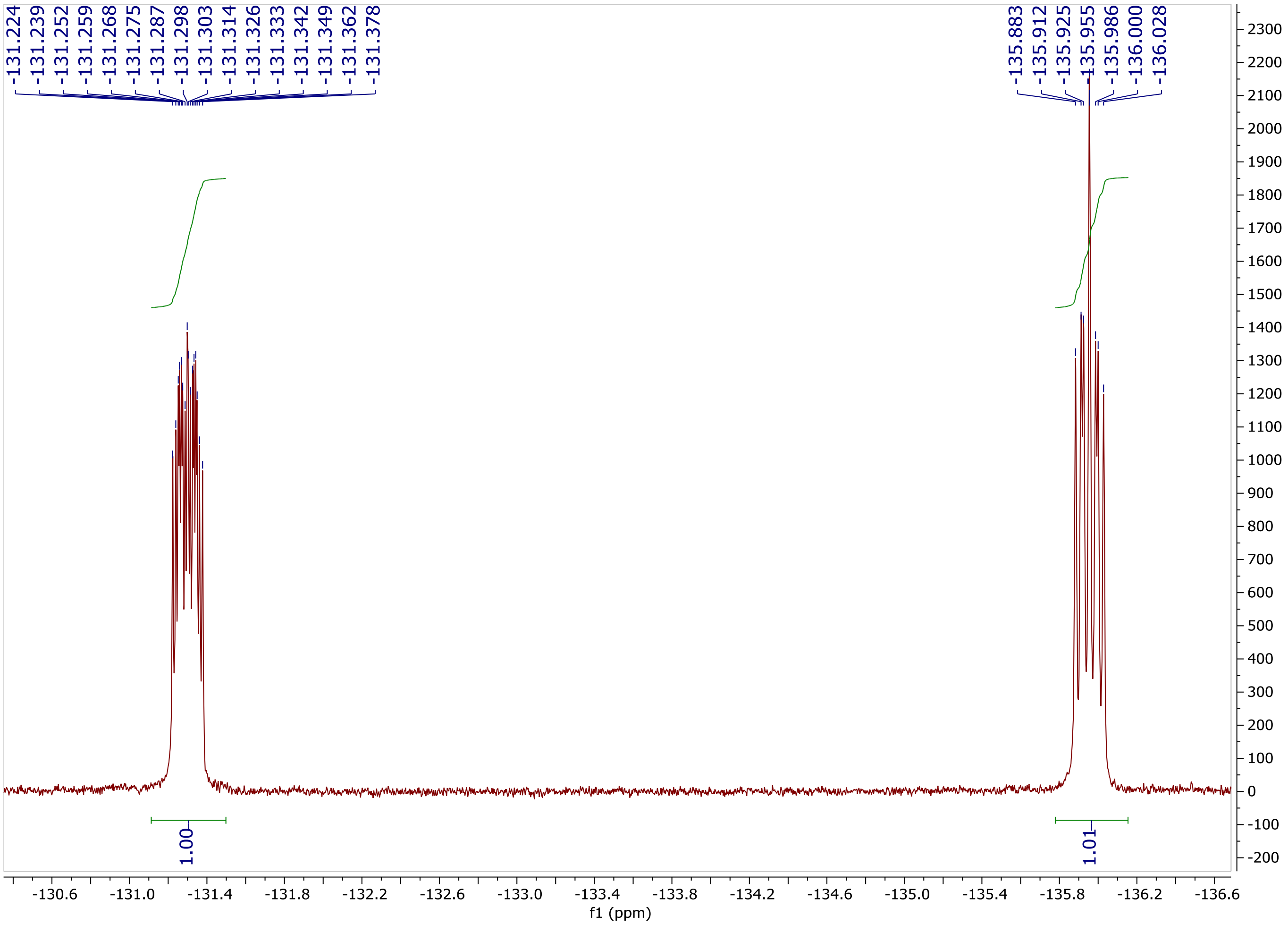

^{19}F NMR spectrum ($CDCl_3$, 75.5 MHz) of **2o**

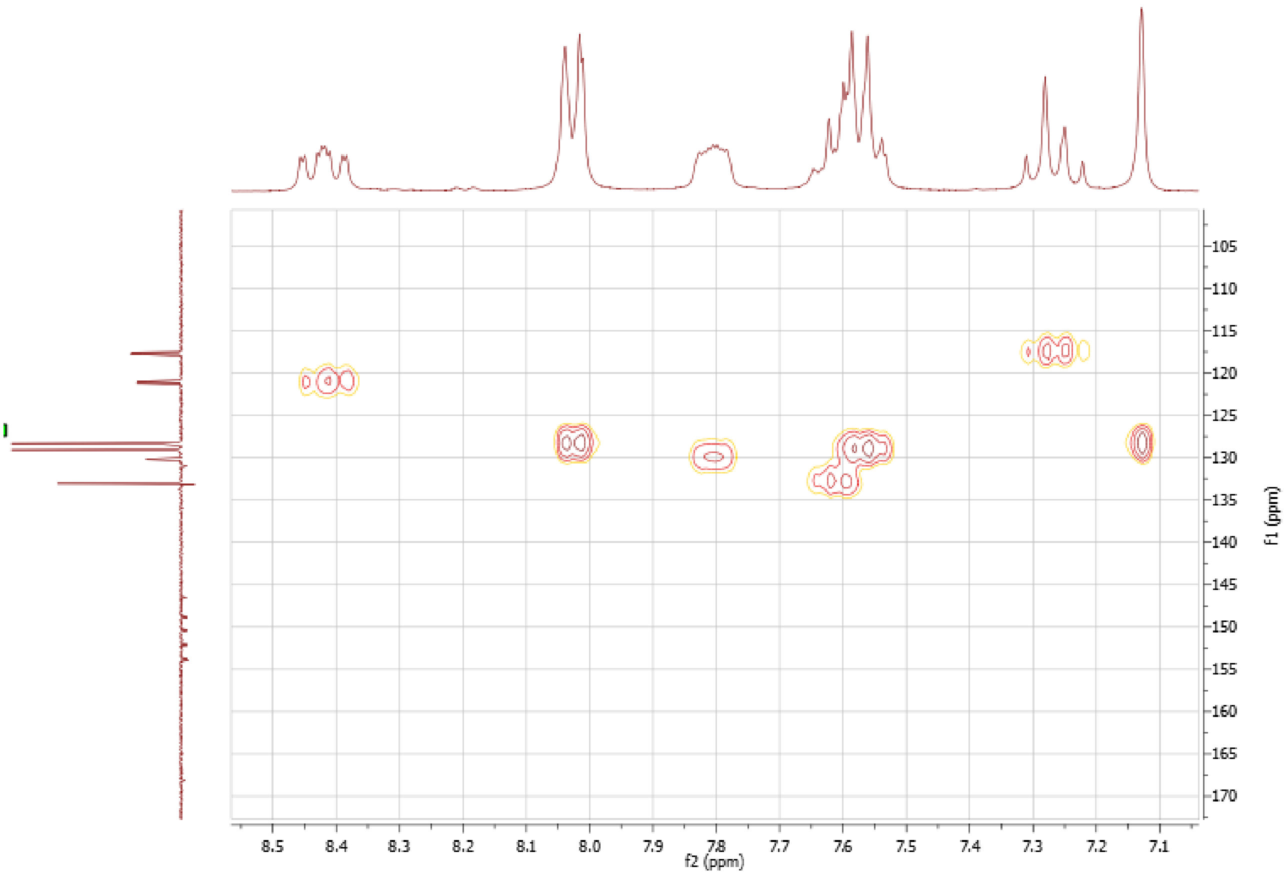

^{1}H-^{13}C HSQC correlation spectrum of **2o**

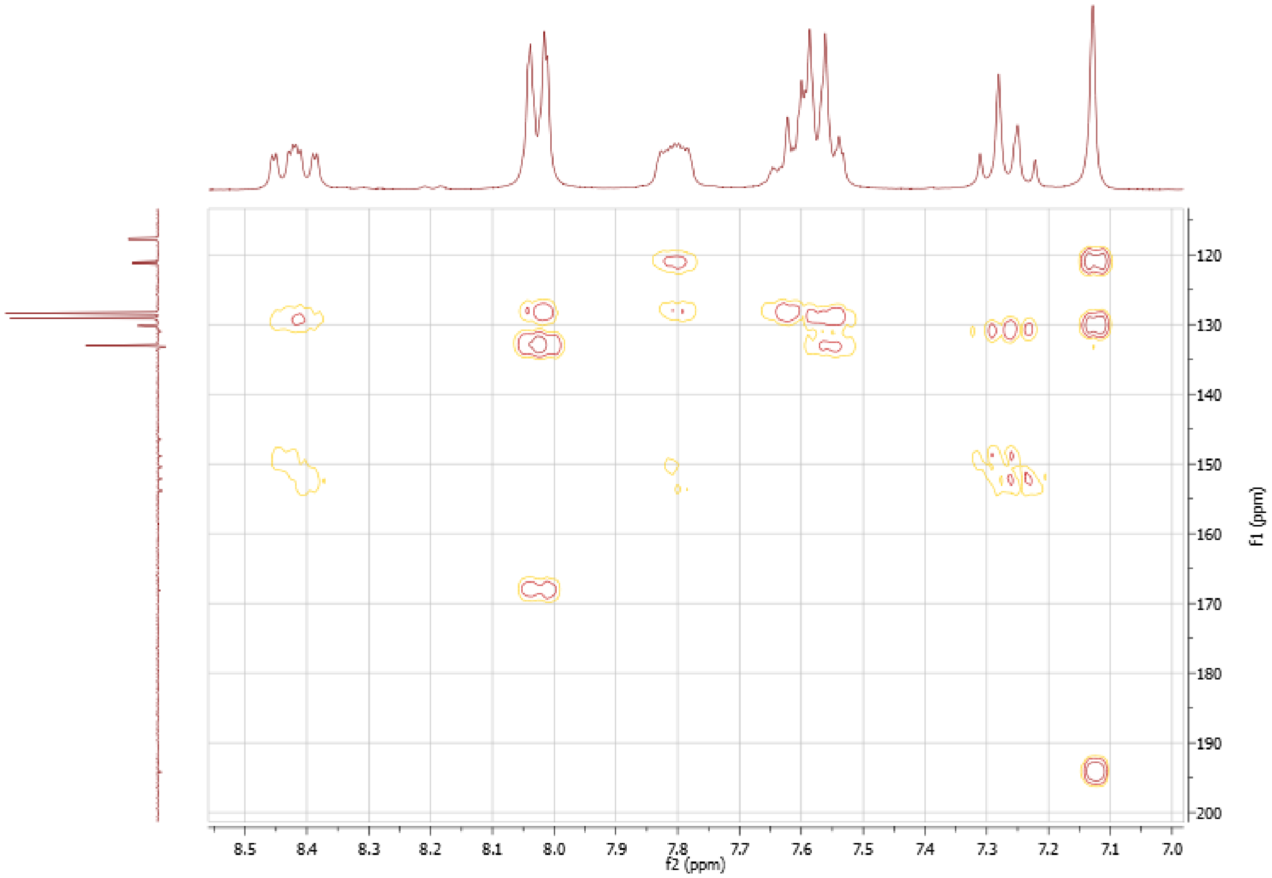

^{1}H-^{13}C HMBC correlation spectrum of **2o**

2.- NMR spectra of [2+2]-photocycloaddition products 3 (photocycloaddition in absence of BF_3)

2,6,9,12-tetraphenyl-3,10-dithia-1,8-diazadispiro[4.1.4^7.1^5]dodeca-1,8-diene-4,11-dione 3a

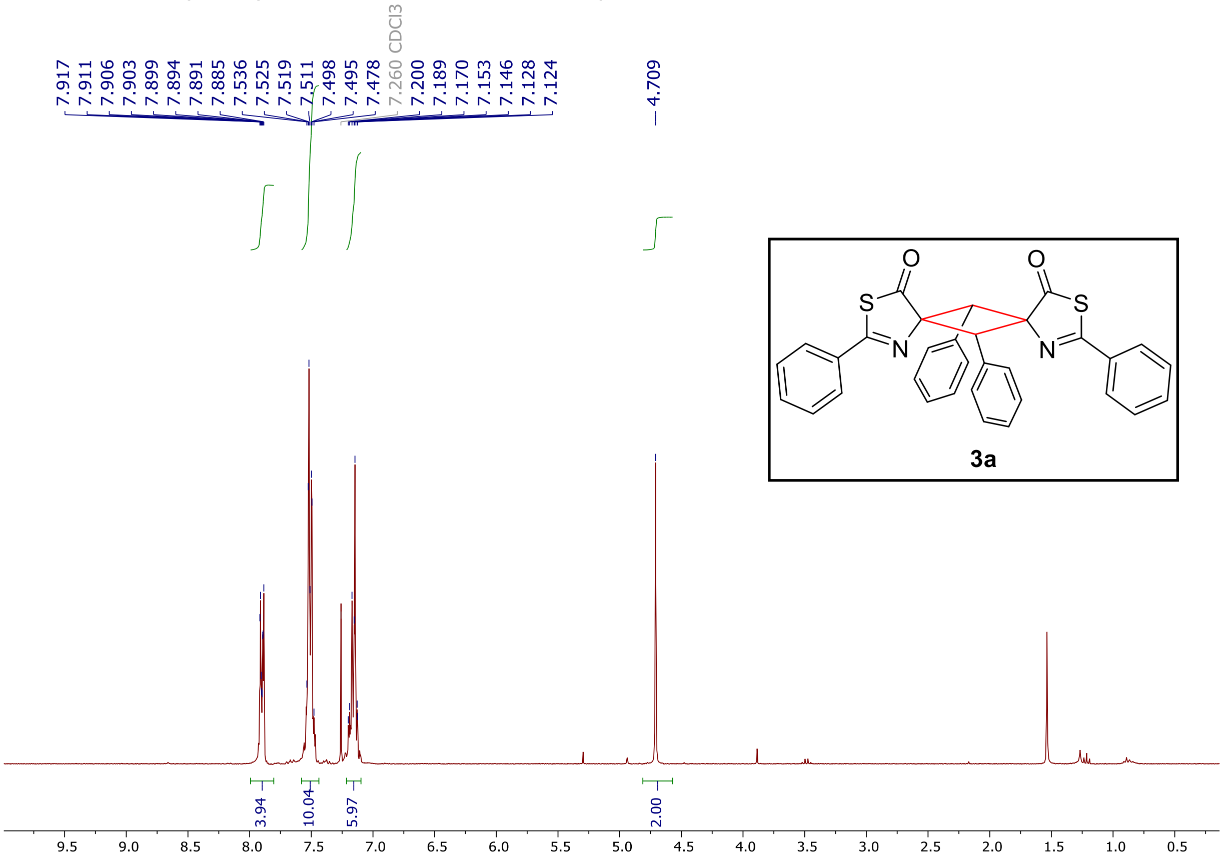

^{1}H NMR spectrum ($CDCl_3$, 300.13 MHz) of **3a**

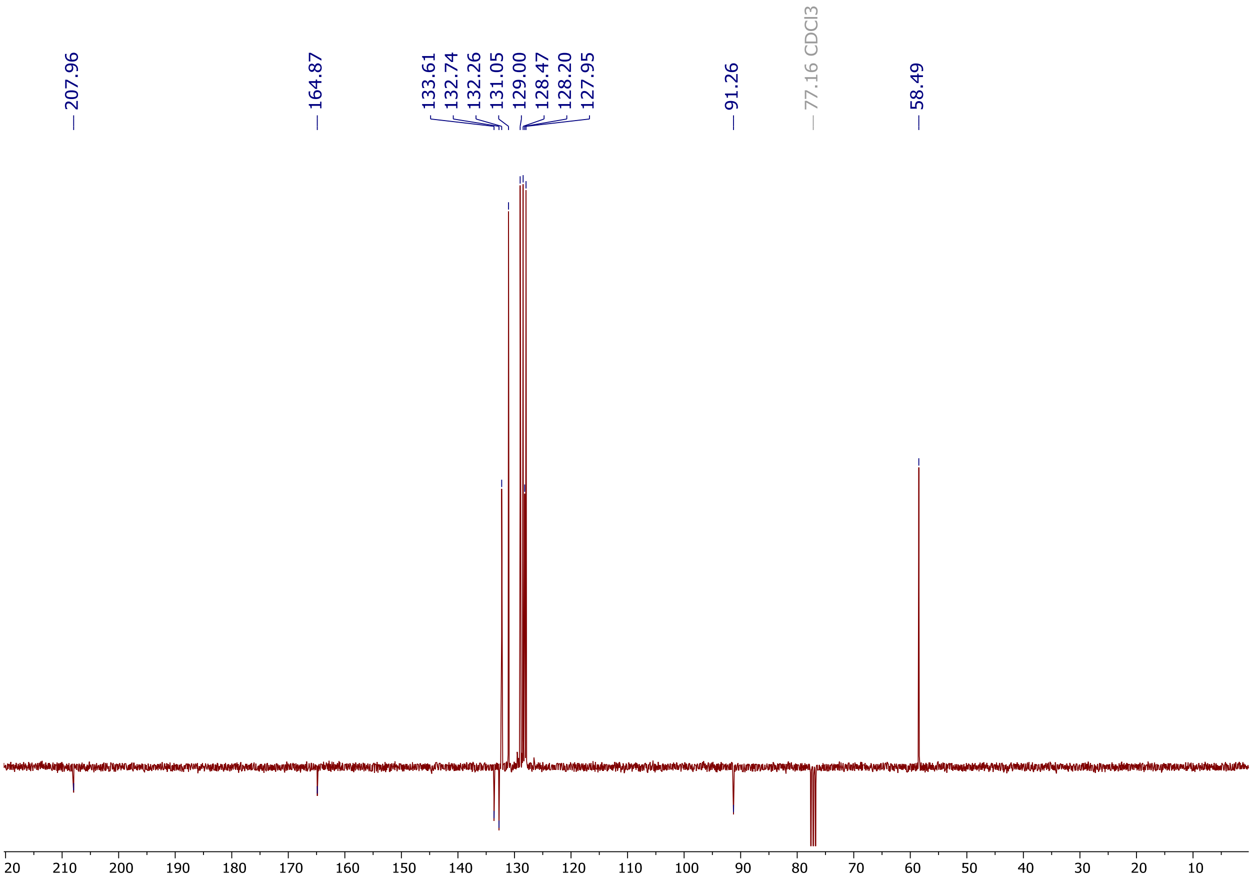

$^{13}C\{^1H\}$ NMR (APT) spectrum ($CDCl_3$, 75.5 MHz) of **3a**

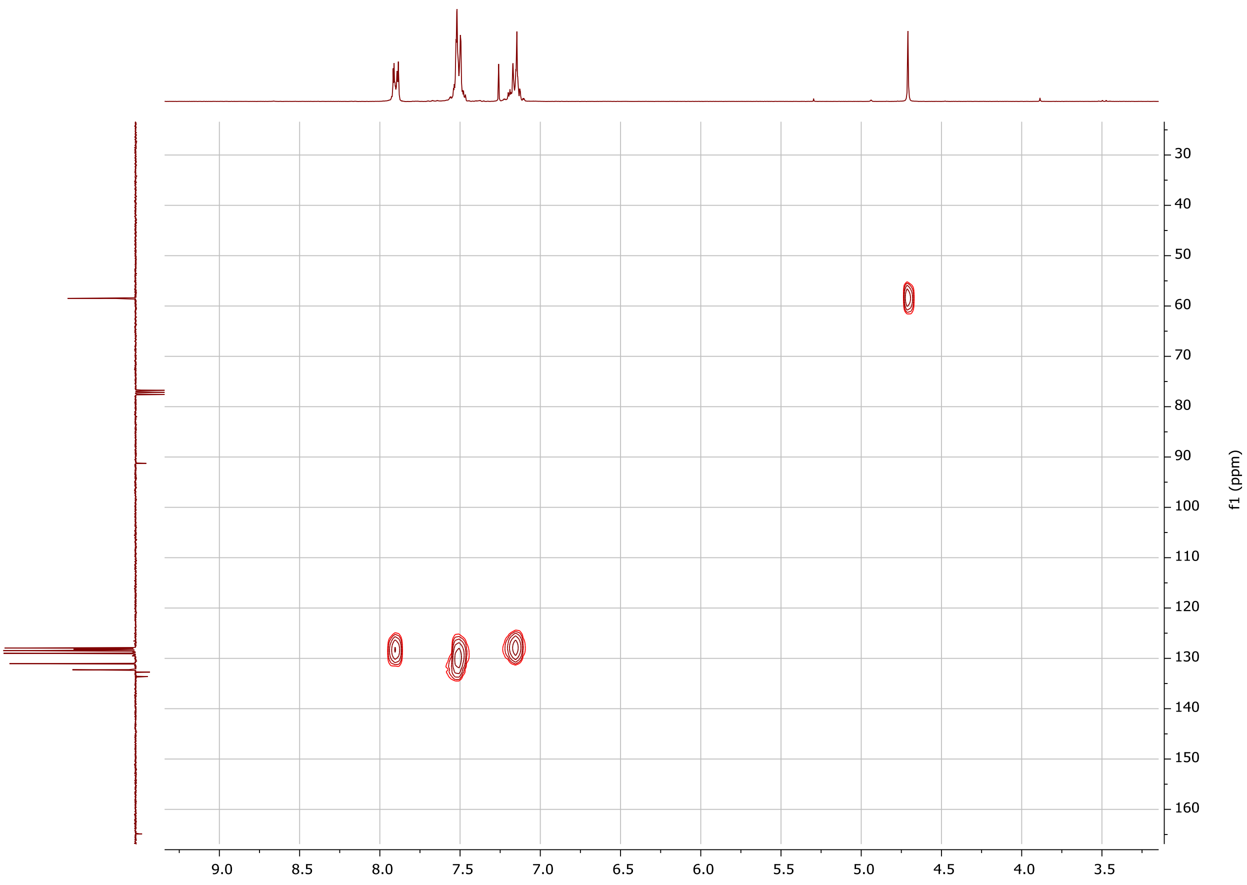

^{1}H-^{13}C HSQC spectrum of **3a**

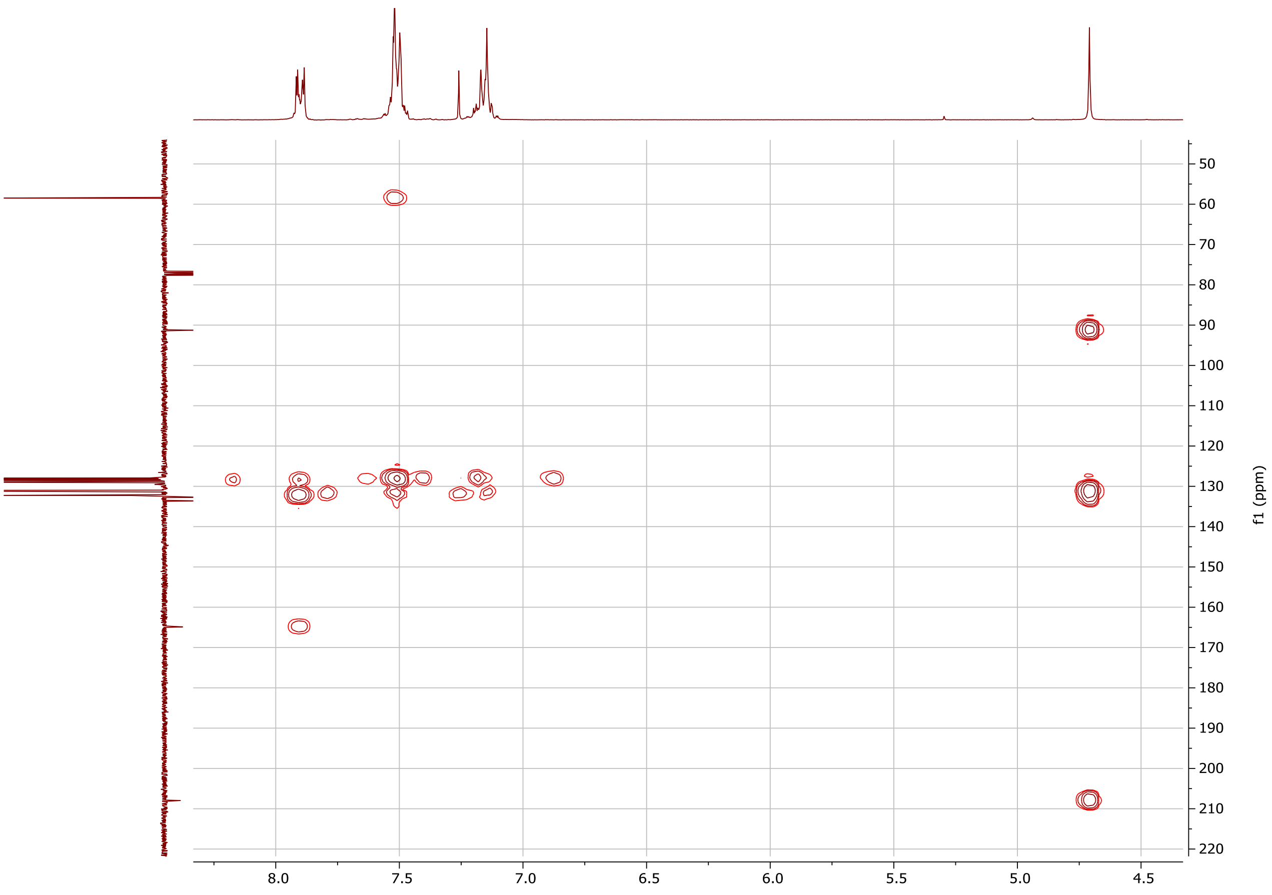

^{1}H-^{13}C HMBC spectrum of **3a**

2,9-diphenyl-6,12-di-*p*-tolyl-3,10-dithia-1,8-diazadispiro[4.1.4^{7}.1^{5}]dodeca-1,8-diene-4,11-dione 3b

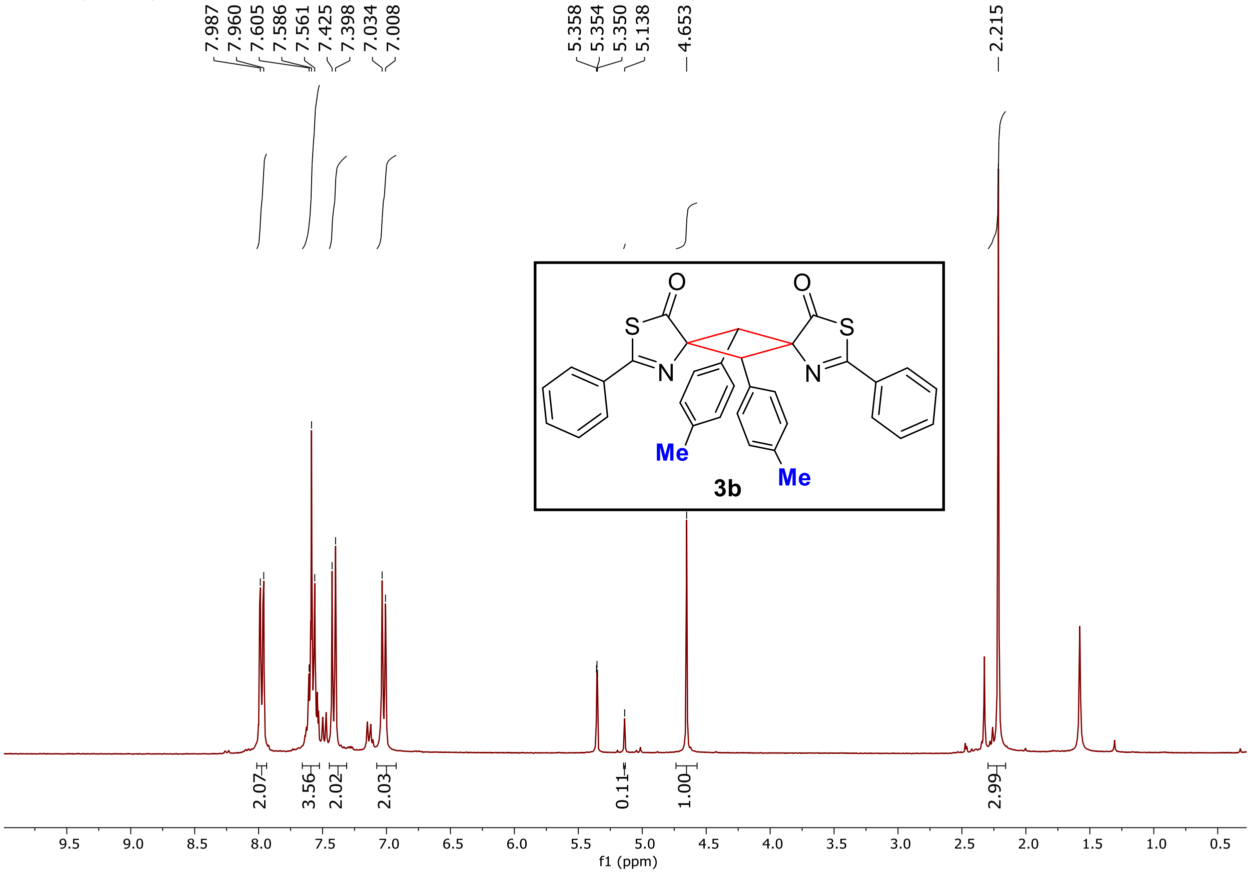

^{1}H NMR spectrum (CD_2Cl_2, 300.13 MHz) of **3b**

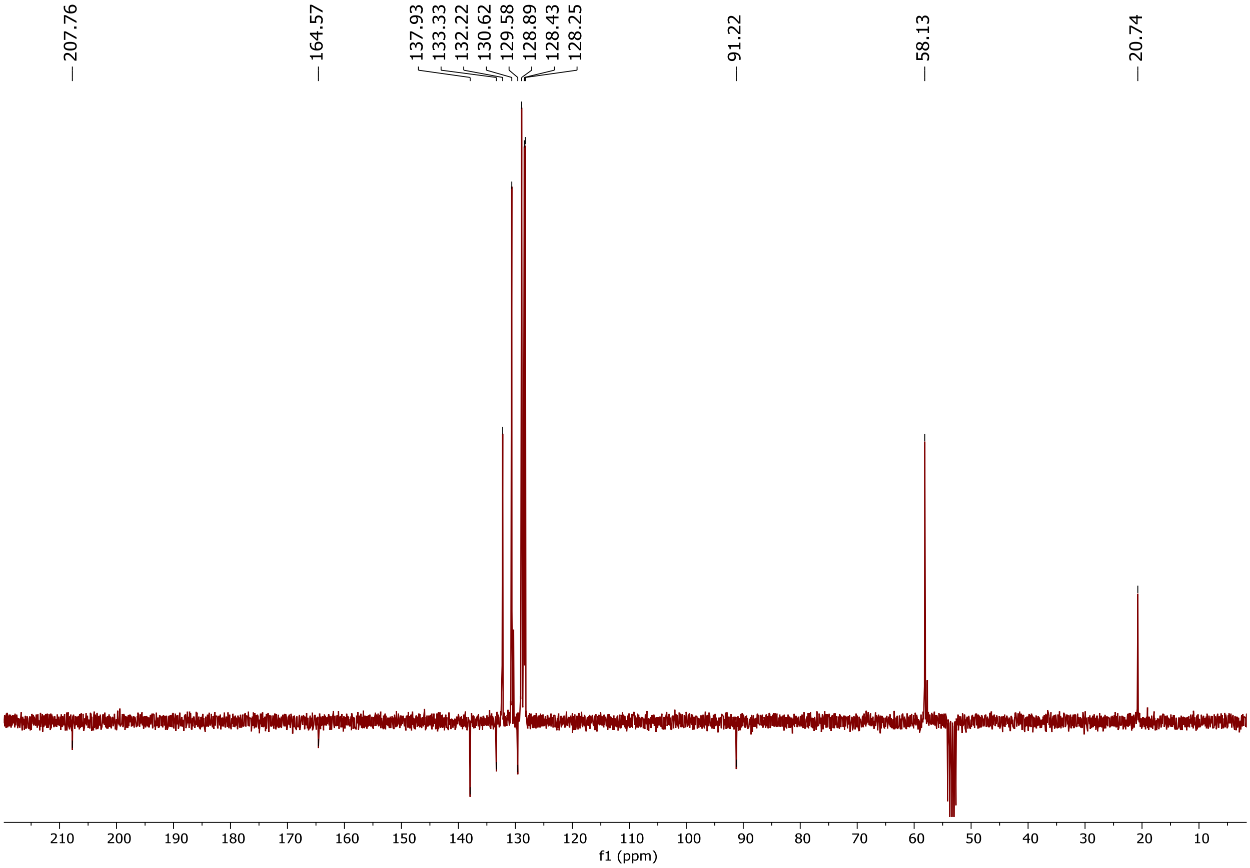

^{13}C{^{1}H} NMR (APT) spectrum (CD_2Cl_2, 75.5 MHz) of **3b**

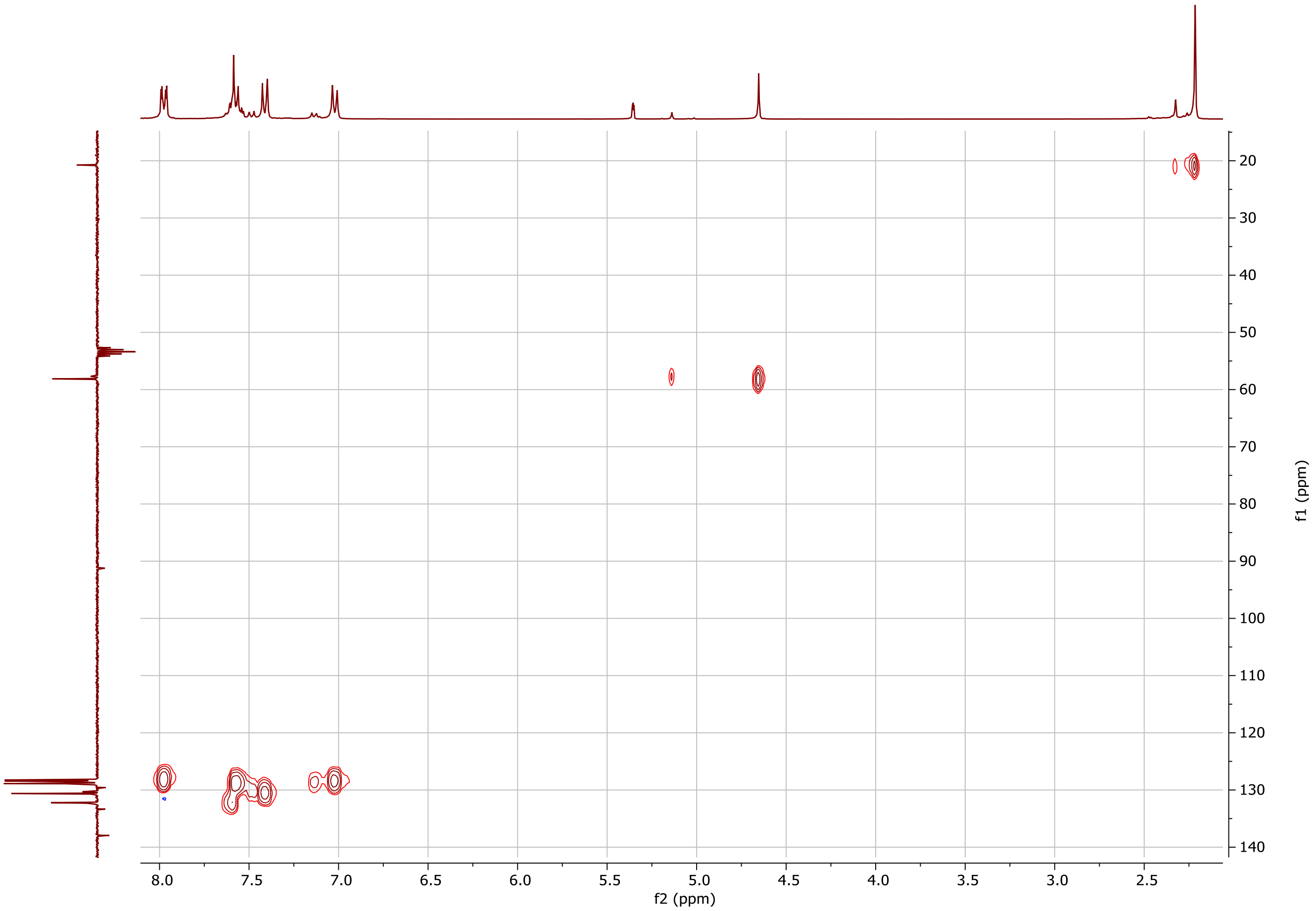

^{1}H-^{13}C HSQC correlation spectrum of **3b**

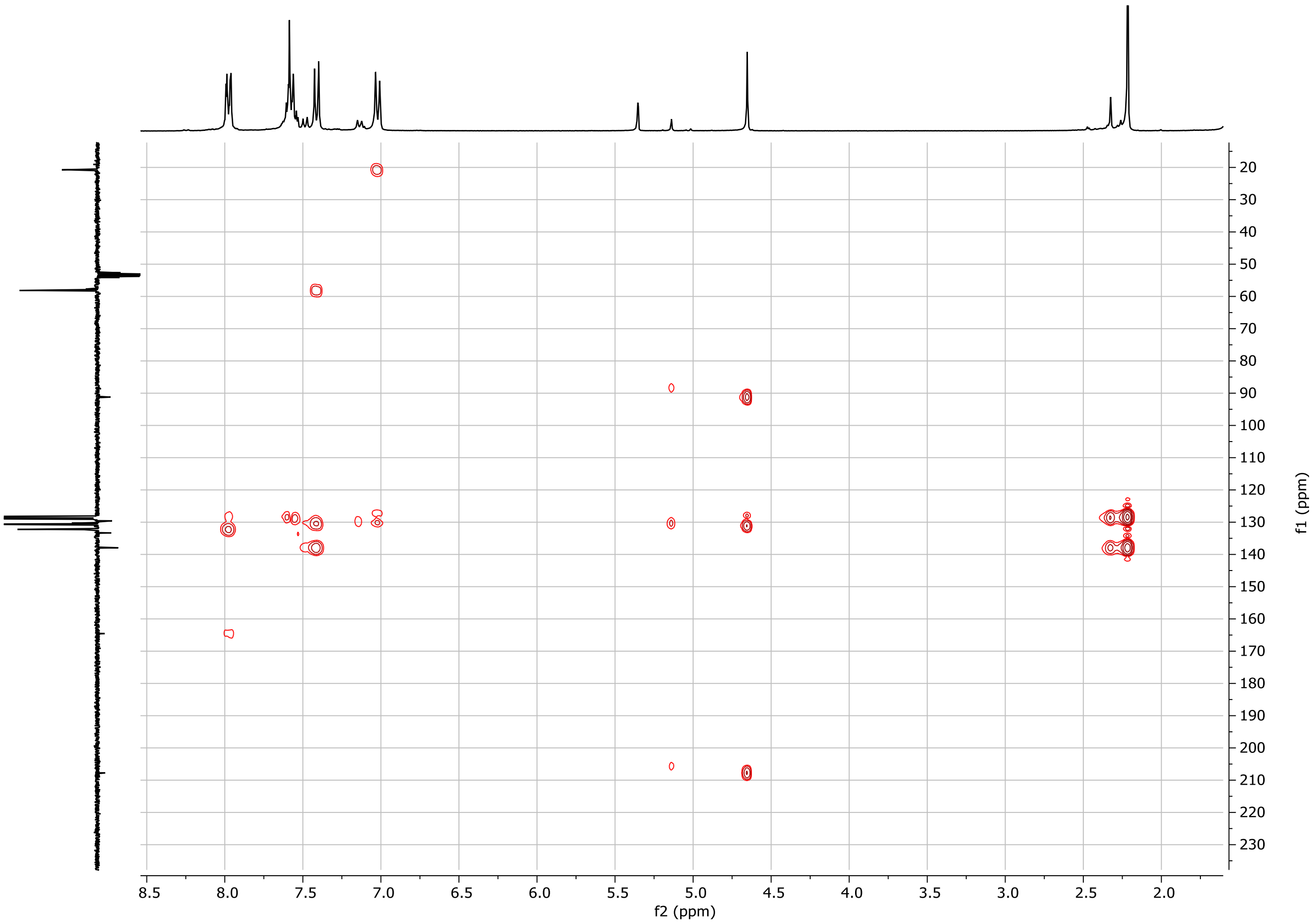

^{1}H-^{13}C HMBC correlation spectrum of **3b**

6,12-bis(4-methoxyphenyl)-2,9-diphenyl-3,10-dithia-1,8-diazadispiro[4.1.4^7.1^5]dodeca-1,8-diene-4,11-dione 3c

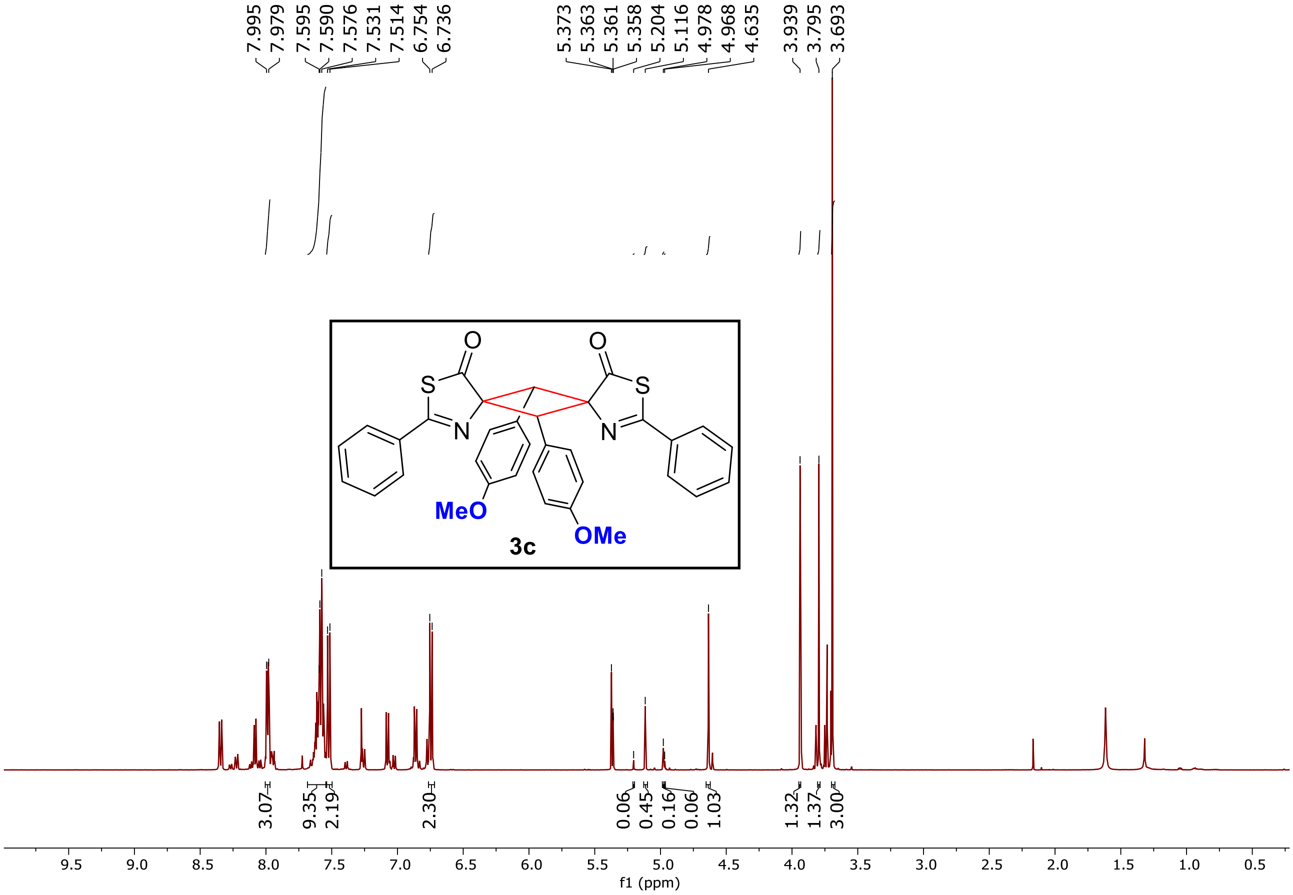

^{1}H NMR spectrum (CD_2Cl_2, 300.13 MHz) of **3c**

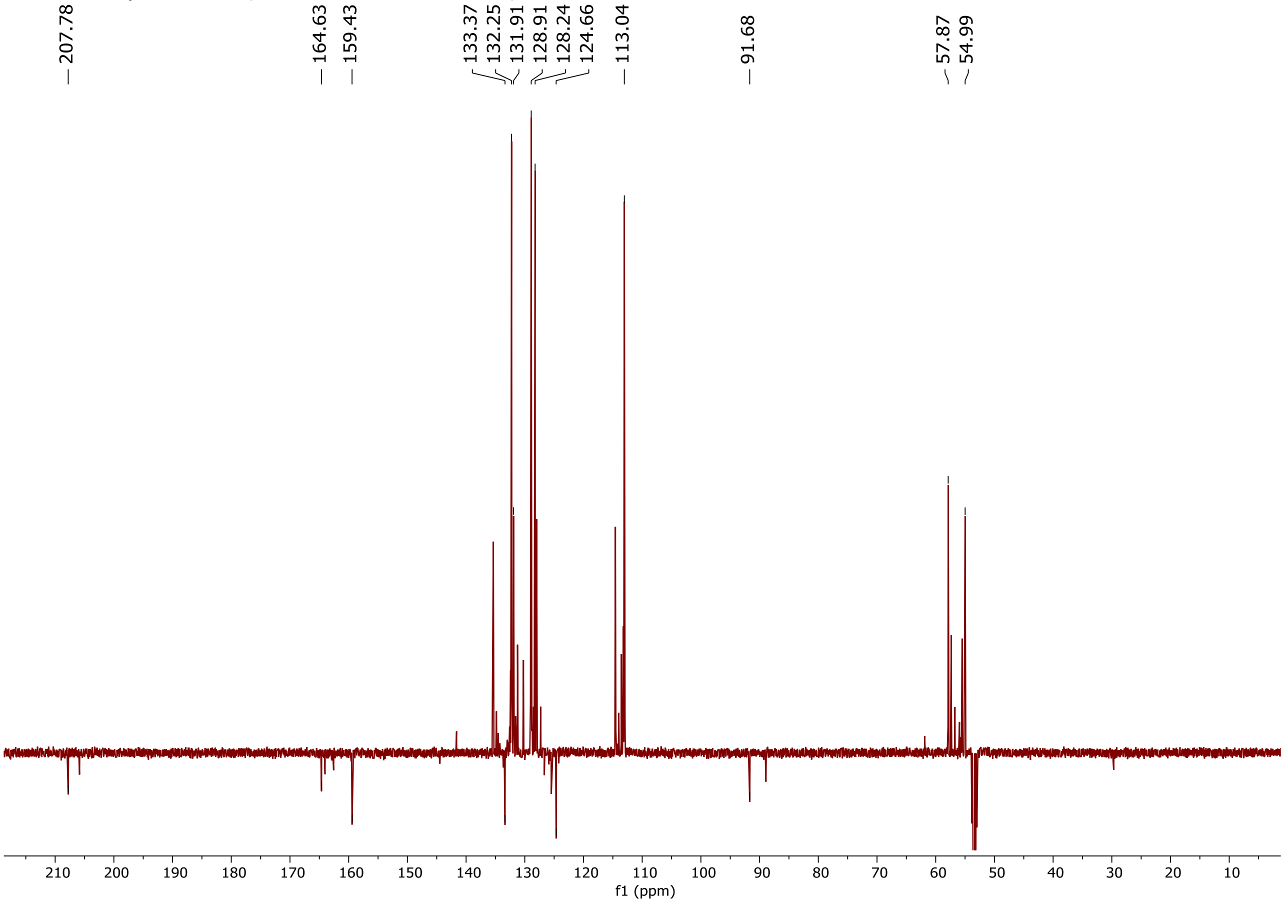

$^{13}C\{^1H\}$ NMR (APT) spectrum (CD_2Cl_2, 75.5 MHz) of **3c**

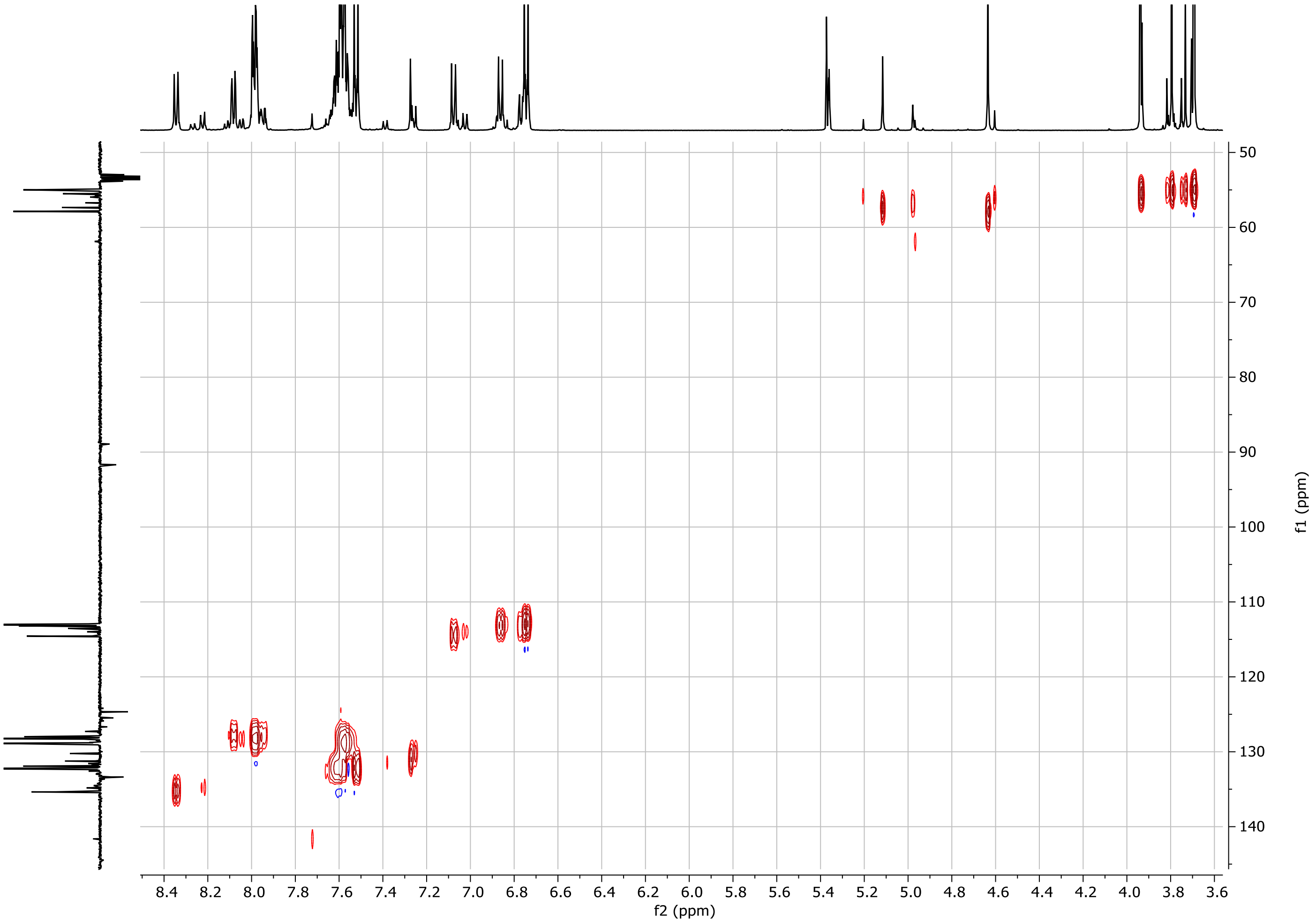

^{1}H-^{13}C HSQC correlation spectrum of **3c**

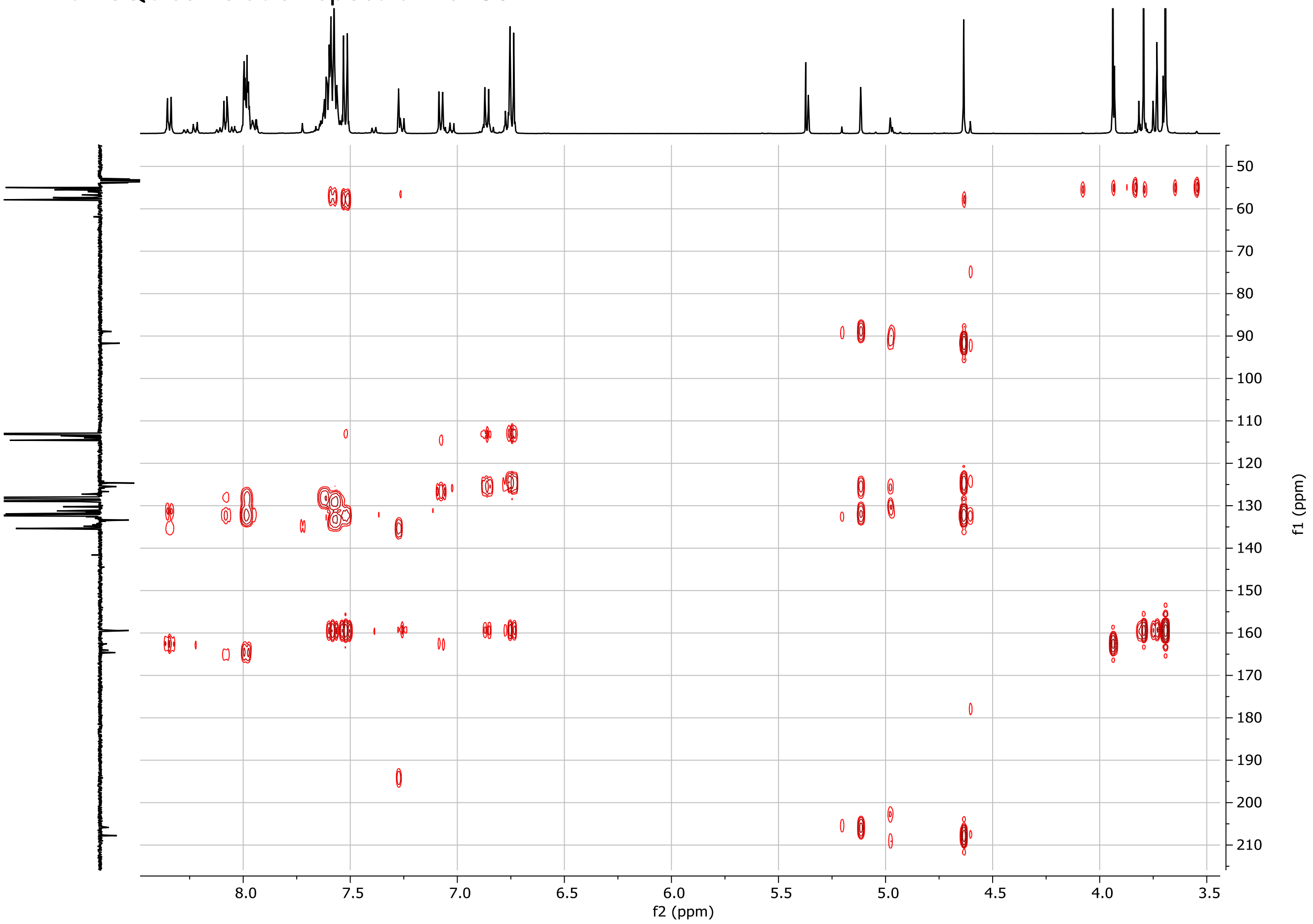

^{1}H-^{13}C HMBC correlation spectrum of **3c**

6,12-bis(4-fluorophenyl)-2,9-diphenyl-3,10-dithia-1,8-diazadispiro[4.1.4^7.1^5]dodeca-1,8-diene-4,11-dione 3d

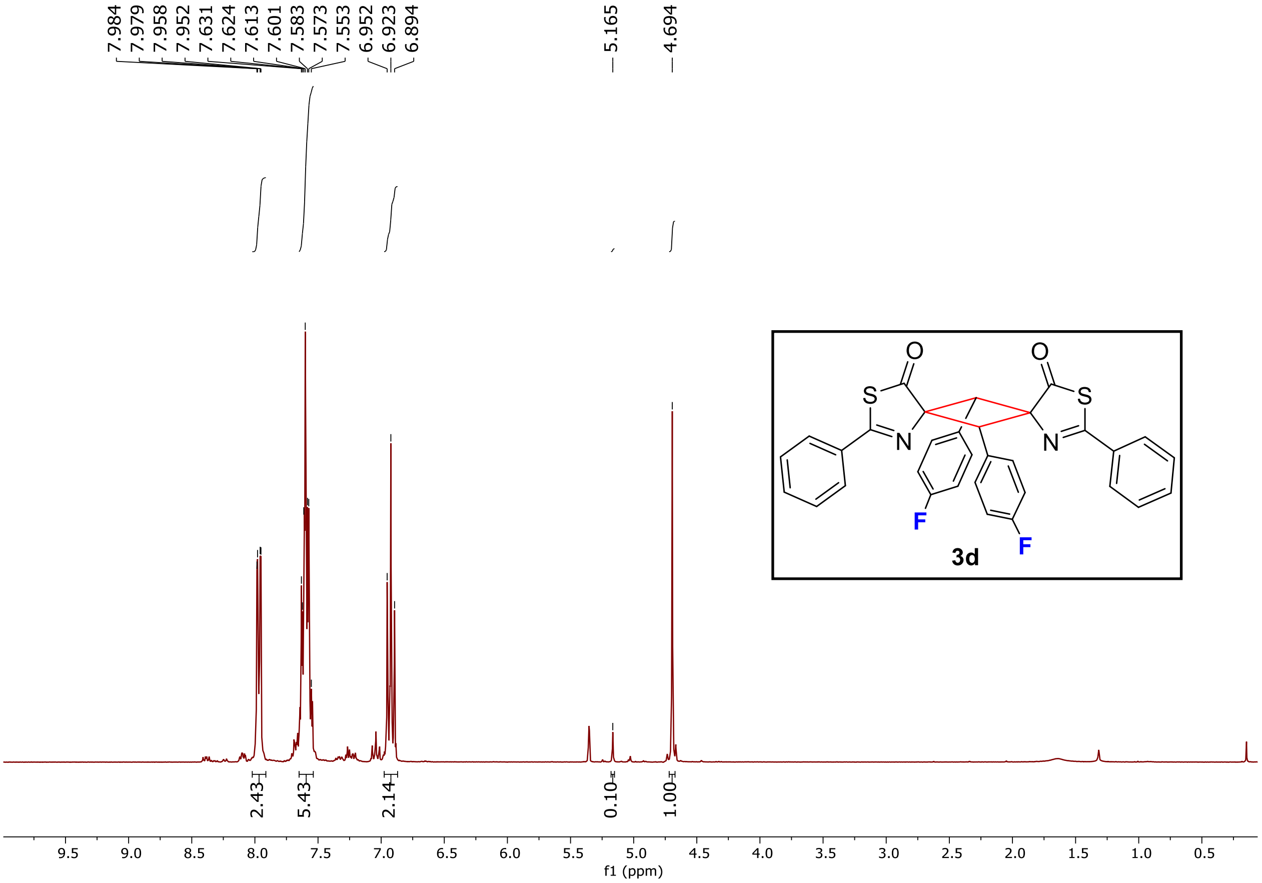

^{1}H NMR spectrum (CD_2Cl_2, 300.13 MHz) of **3d**

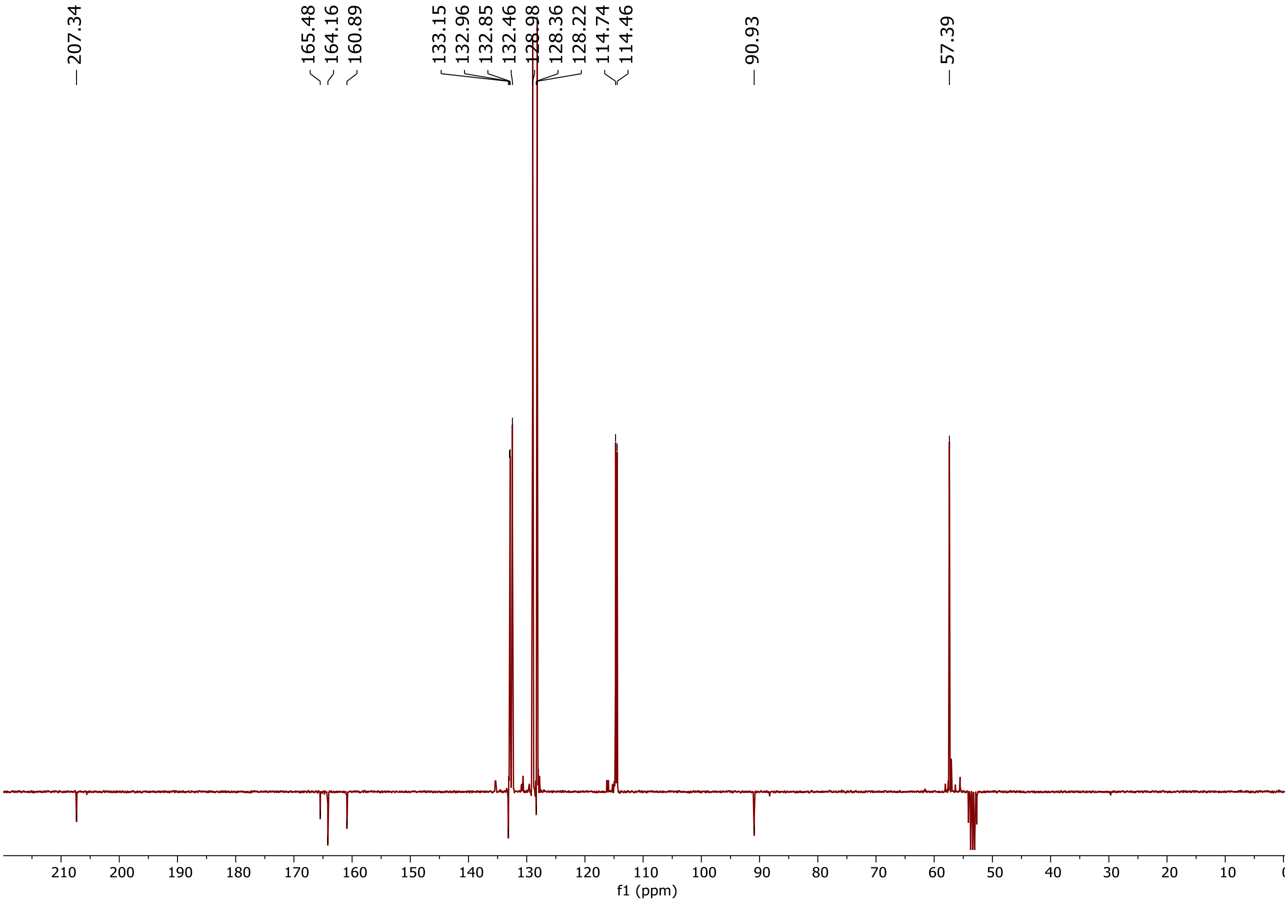

$^{13}C\{^1H\}$ NMR (APT) spectrum (CD_2Cl_2, 75.5 MHz) of **3d**

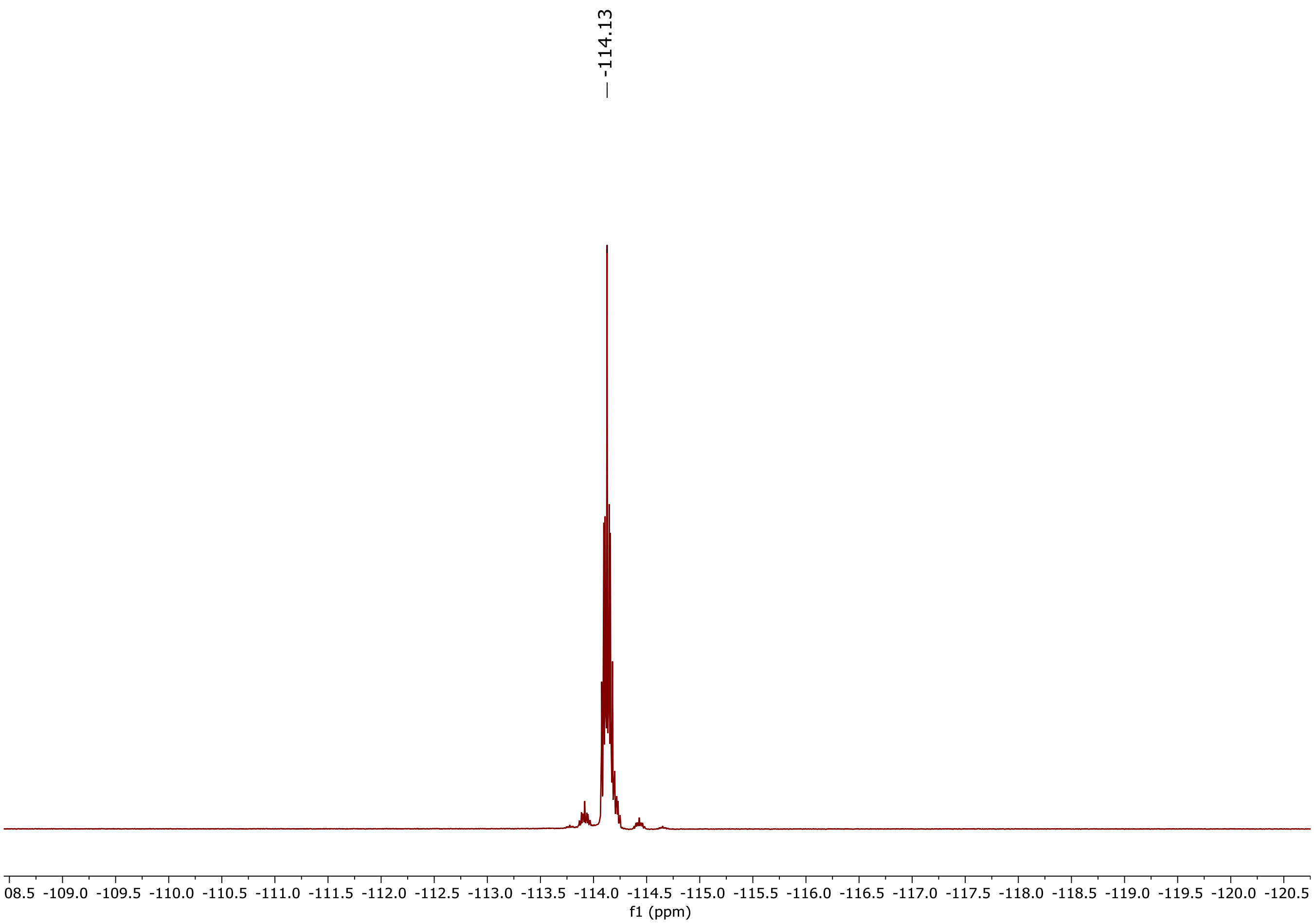

^{19}F NMR spectrum (CD_2Cl_2, 282.4 MHz) of **3d**

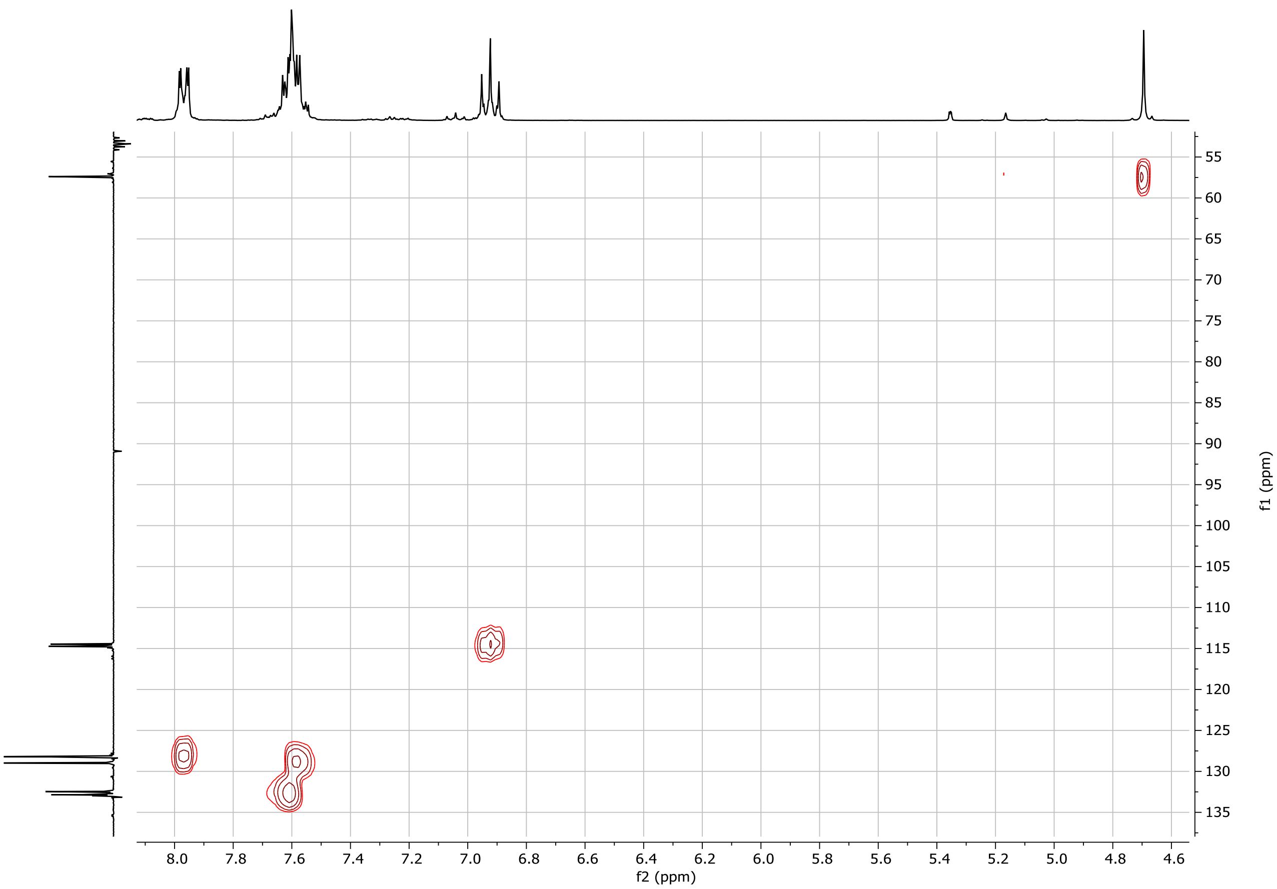

^{1}H-^{13}C HSQC correlation spectrum of **3d**

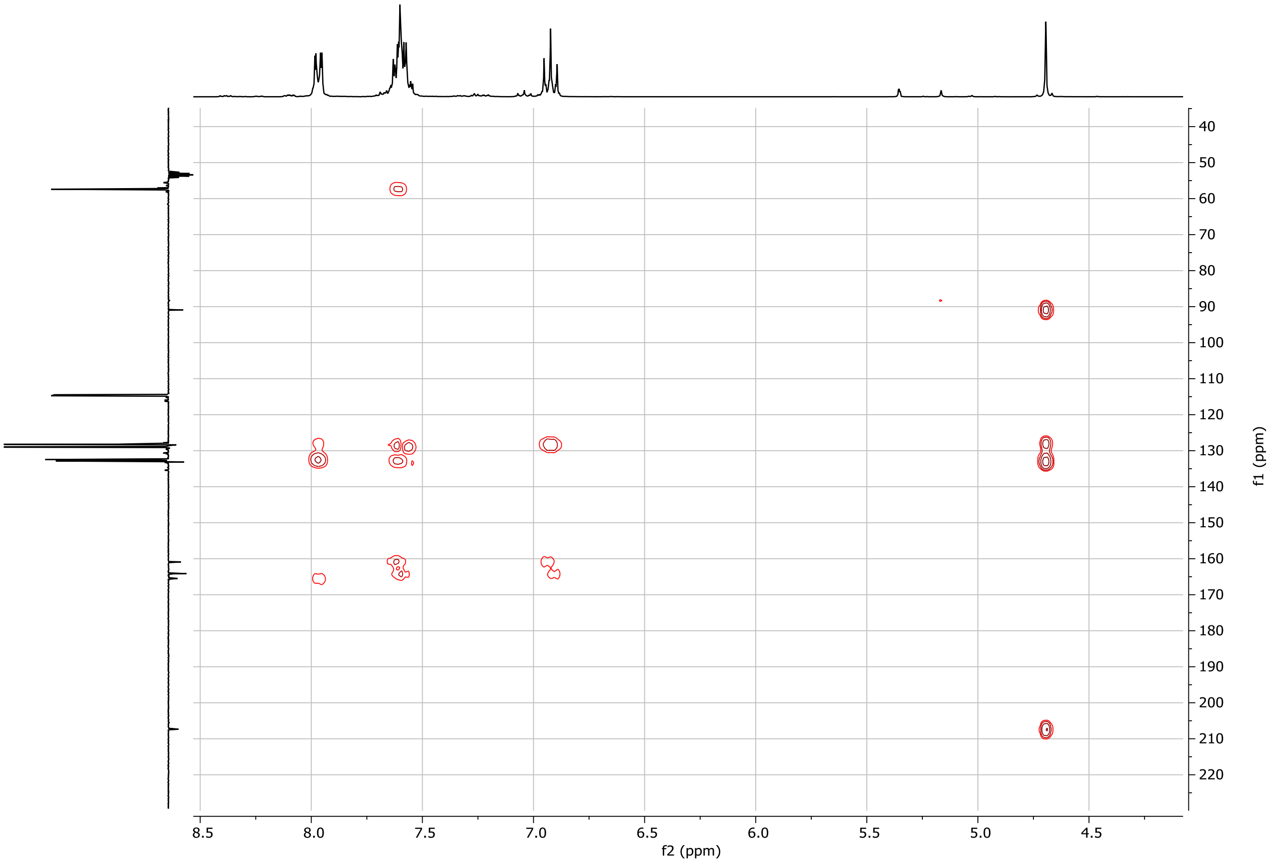

^{1}H-^{13}C HMBC correlation spectrum of **3d**

6,12-bis(4-chlorophenyl)-2,9-diphenyl-3,10-dithia-1,8-diazadispiro[4.1.4^7.1^5]dodeca-1,8-diene-4,11-dione 3e

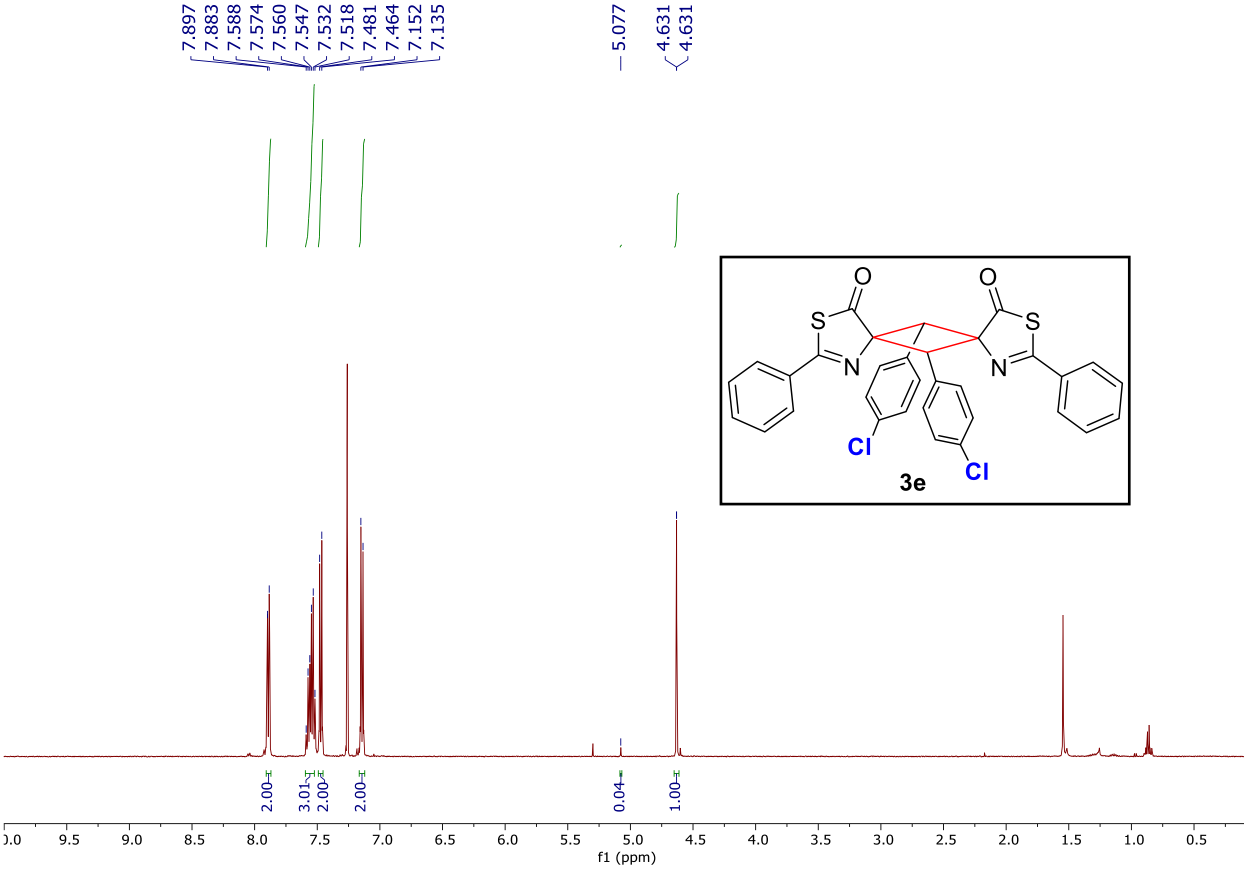

^{1}H NMR spectrum ($CDCl_3$, 500.13 MHz) of **3e**

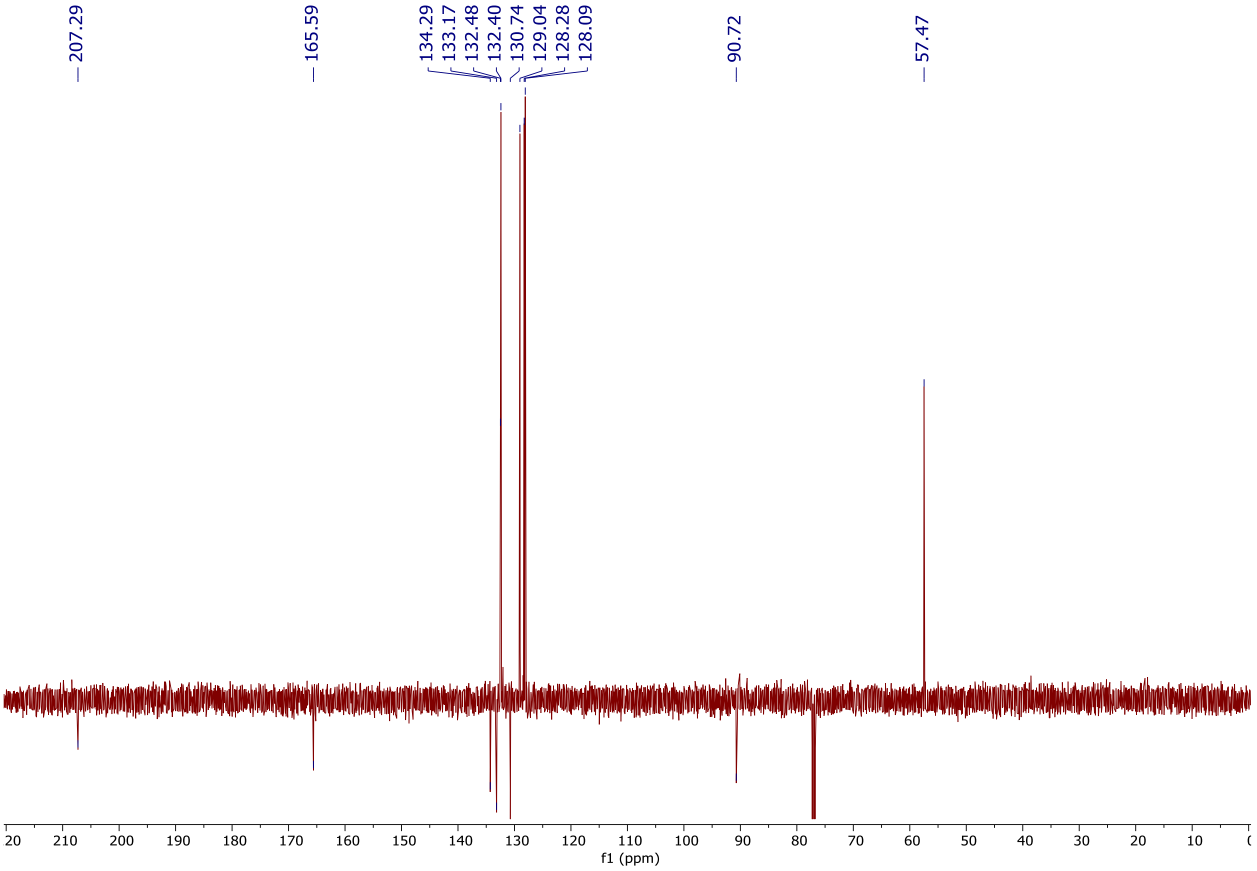

$^{13}C\{^{1}H\}$ NMR (APT) spectrum ($CDCl_3$, 125.76 MHz) of **3e**

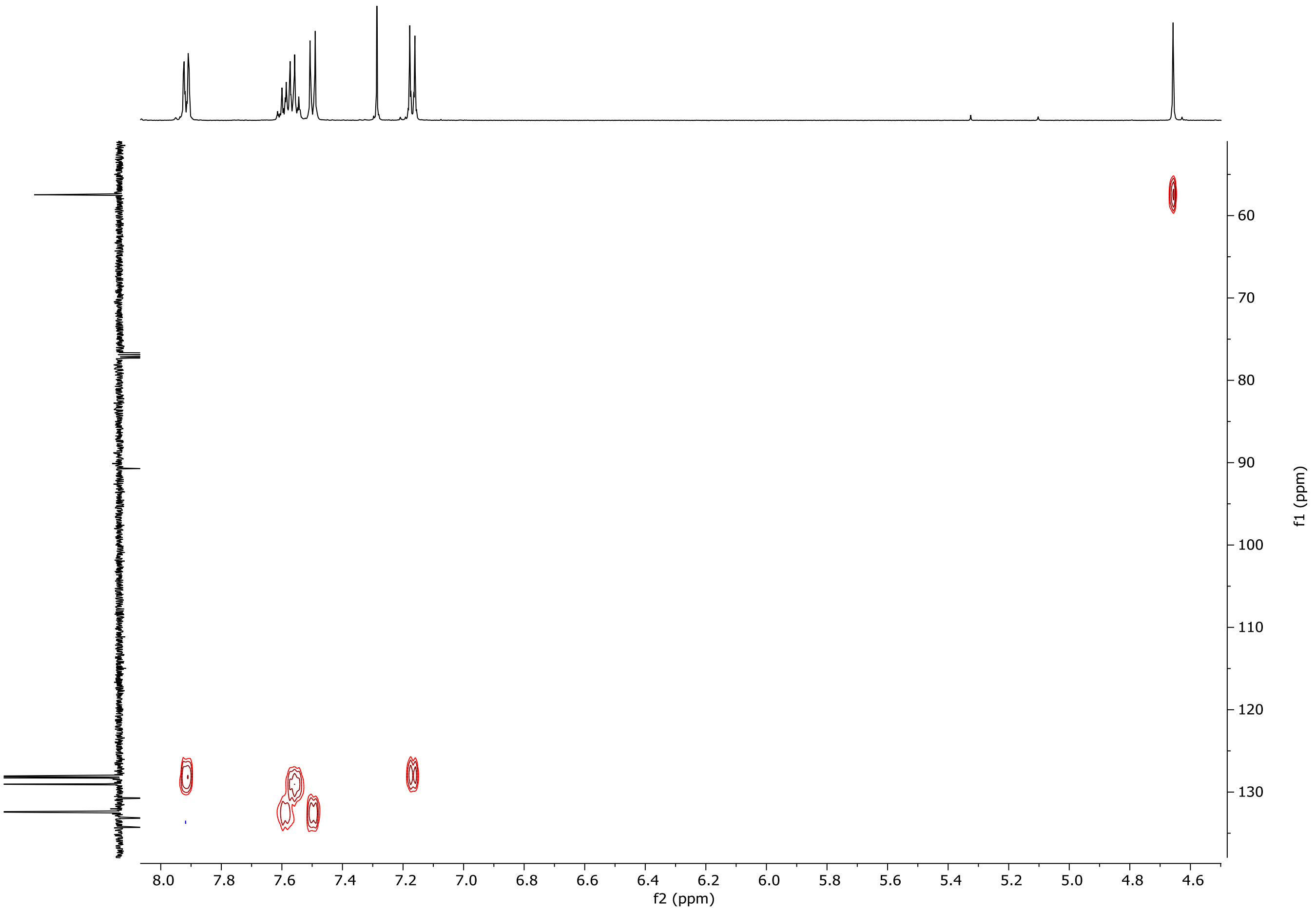

^{1}H-^{13}C HSQC correlation spectrum of **3e**

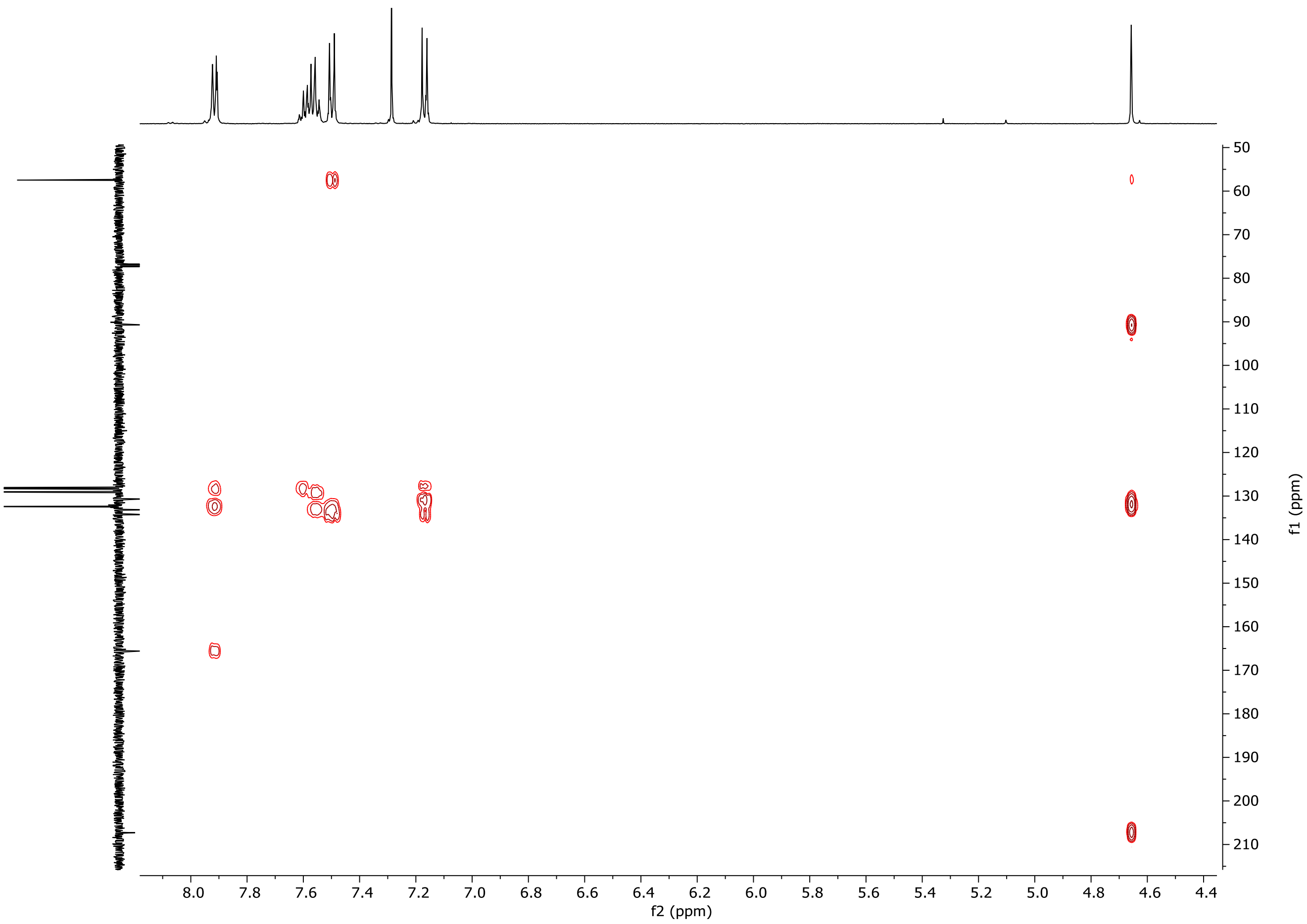

^1H-^{13}C HMBC correlation spectrum of **3e**

6,12-bis(4-bromophenyl)-2,9-diphenyl-3,10-dithia-1,8-diazadispiro[4.1.4^7.1^5]dodeca-1,8-diene-4,11-dione 3f

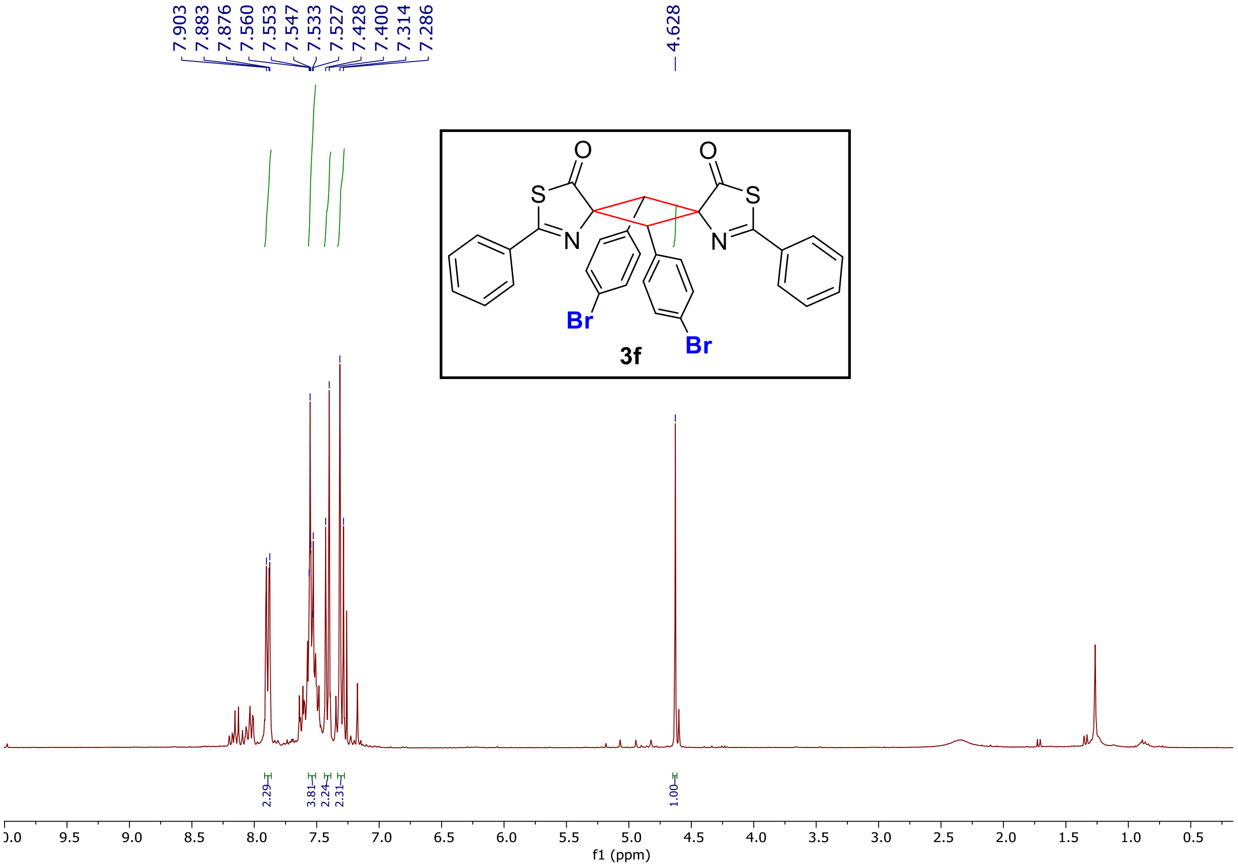

^1H NMR spectrum ($CDCl_3$, 300.13 MHz) of **3f**

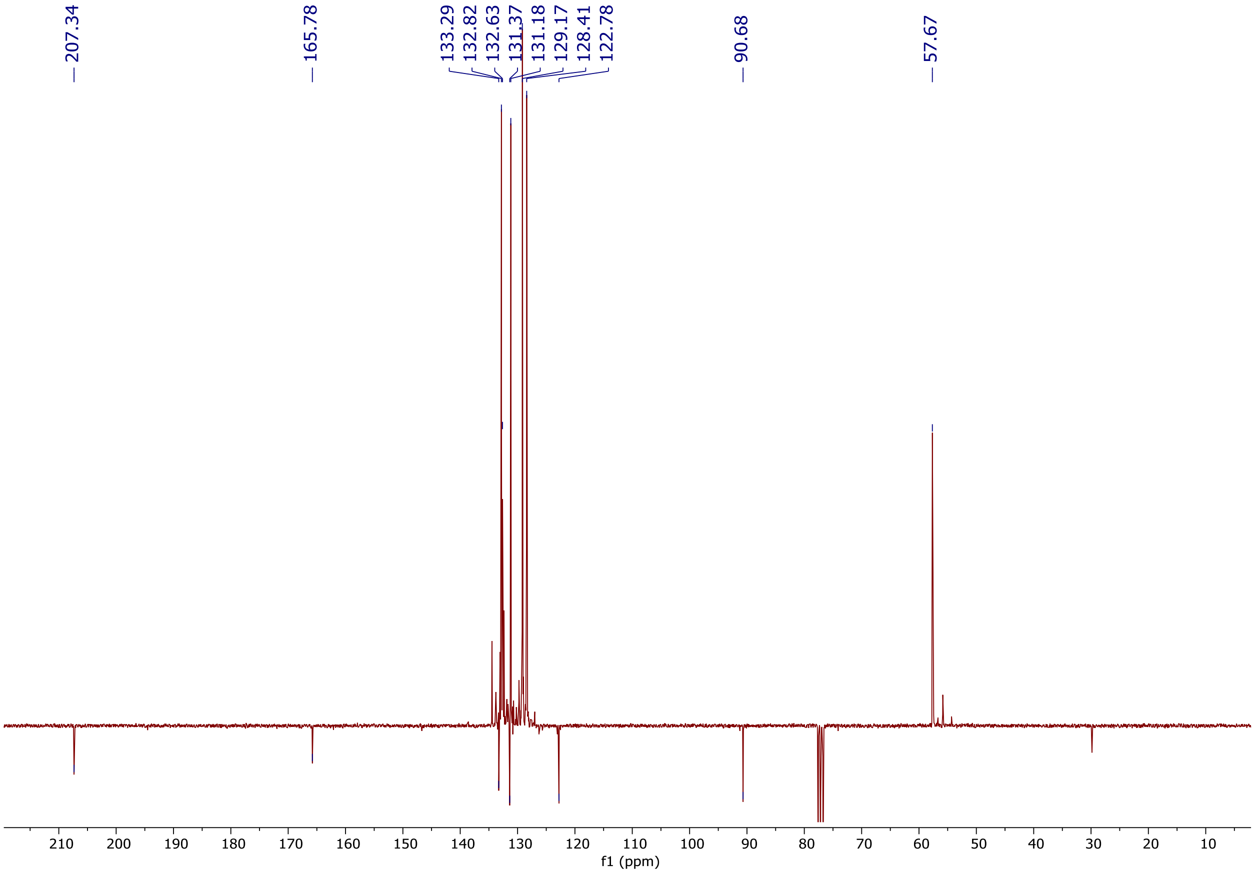

$^{13}C\{^{1}H\}$ NMR (APT) spectrum ($CDCl_3$, 75.47 MHz) of **3f**

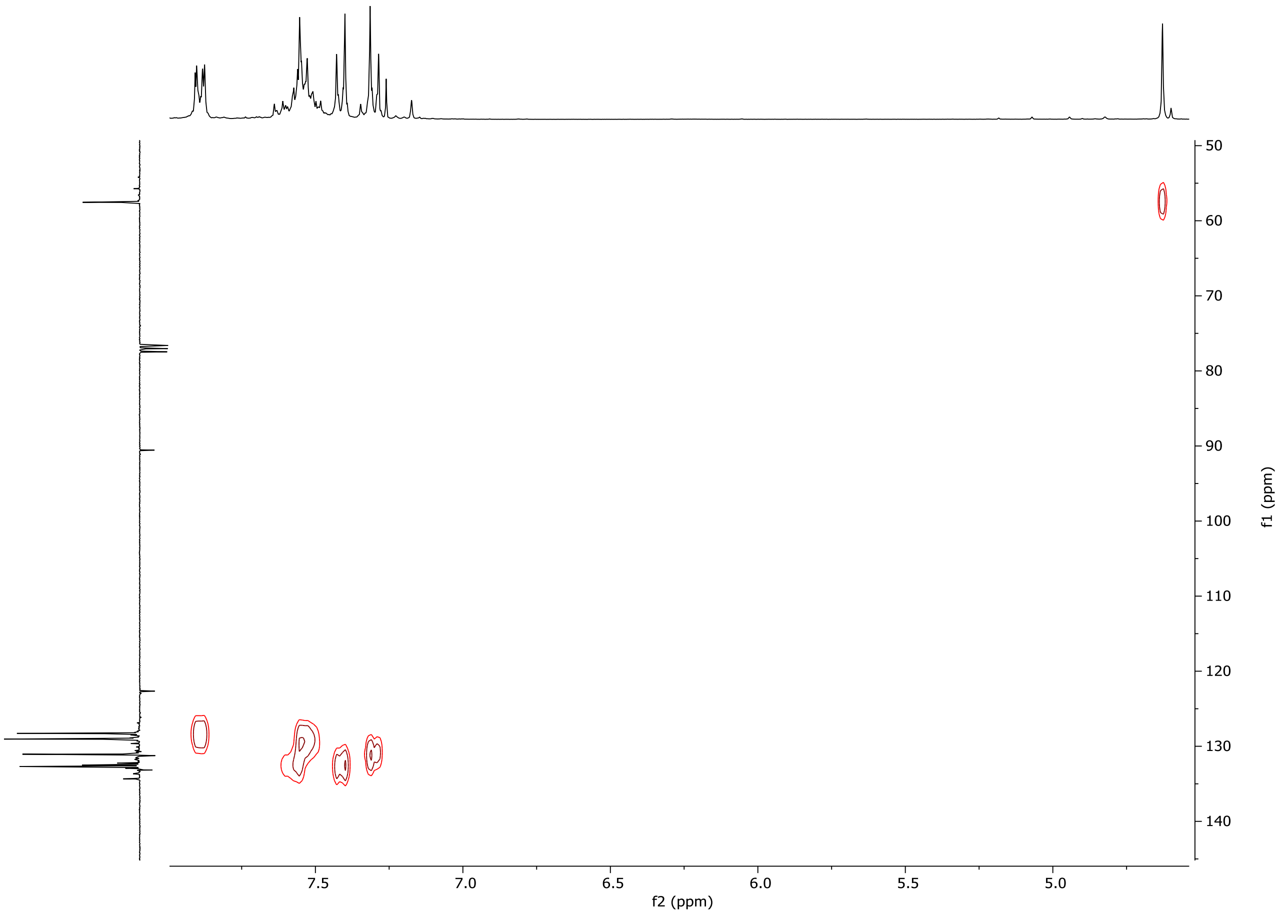

^{1}H-^{13}C HSQC correlation spectrum of **3f**

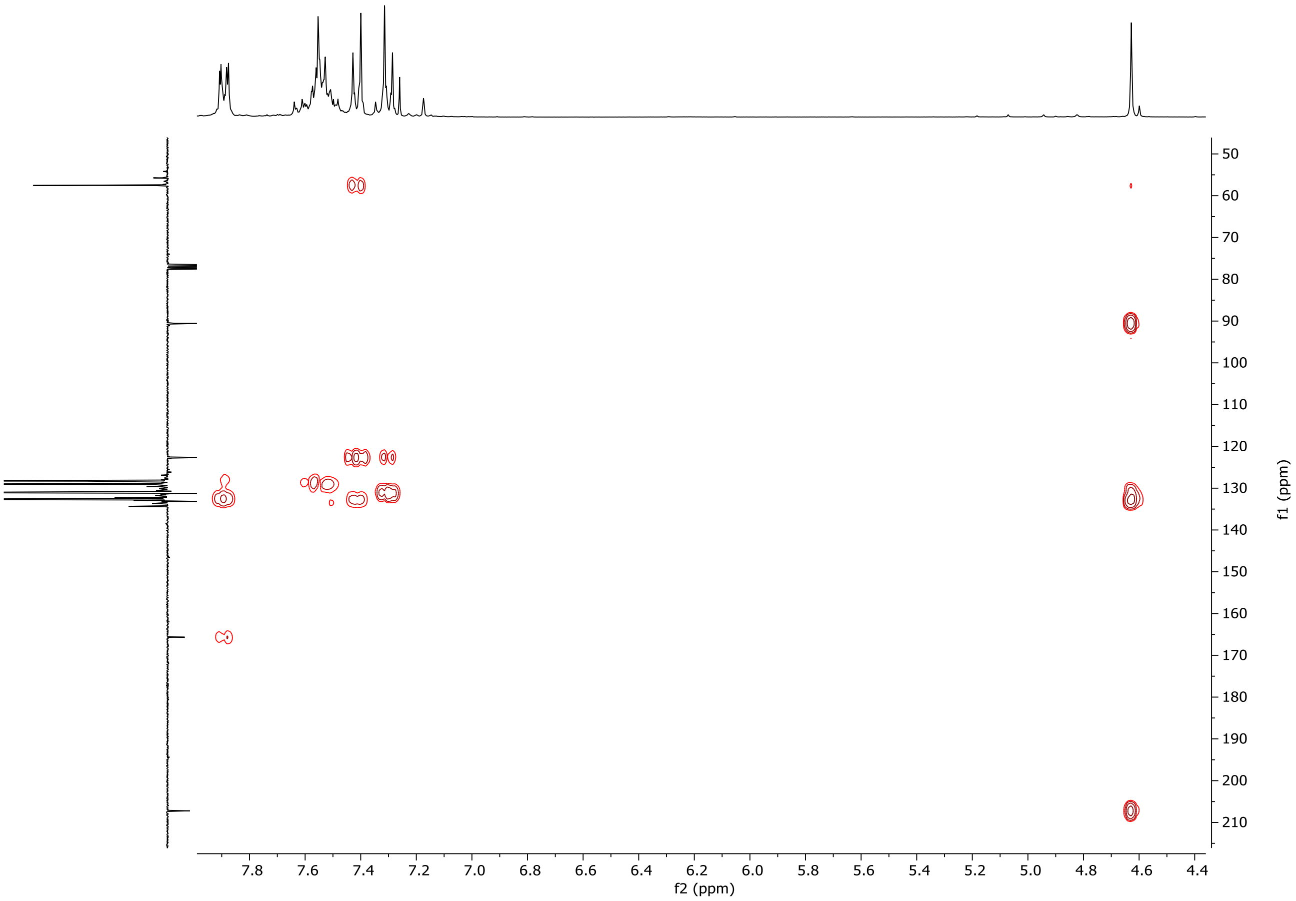

^{1}H-^{13}C HMBC correlation spectrum of **3f**

6,12-bis(4-nitrophenyl)-2,9-diphenyl-3,10-dithia-1,8-diazadispiro[4.1.4^7.1^5]dodeca-1,8-diene-4,11-dione 3g

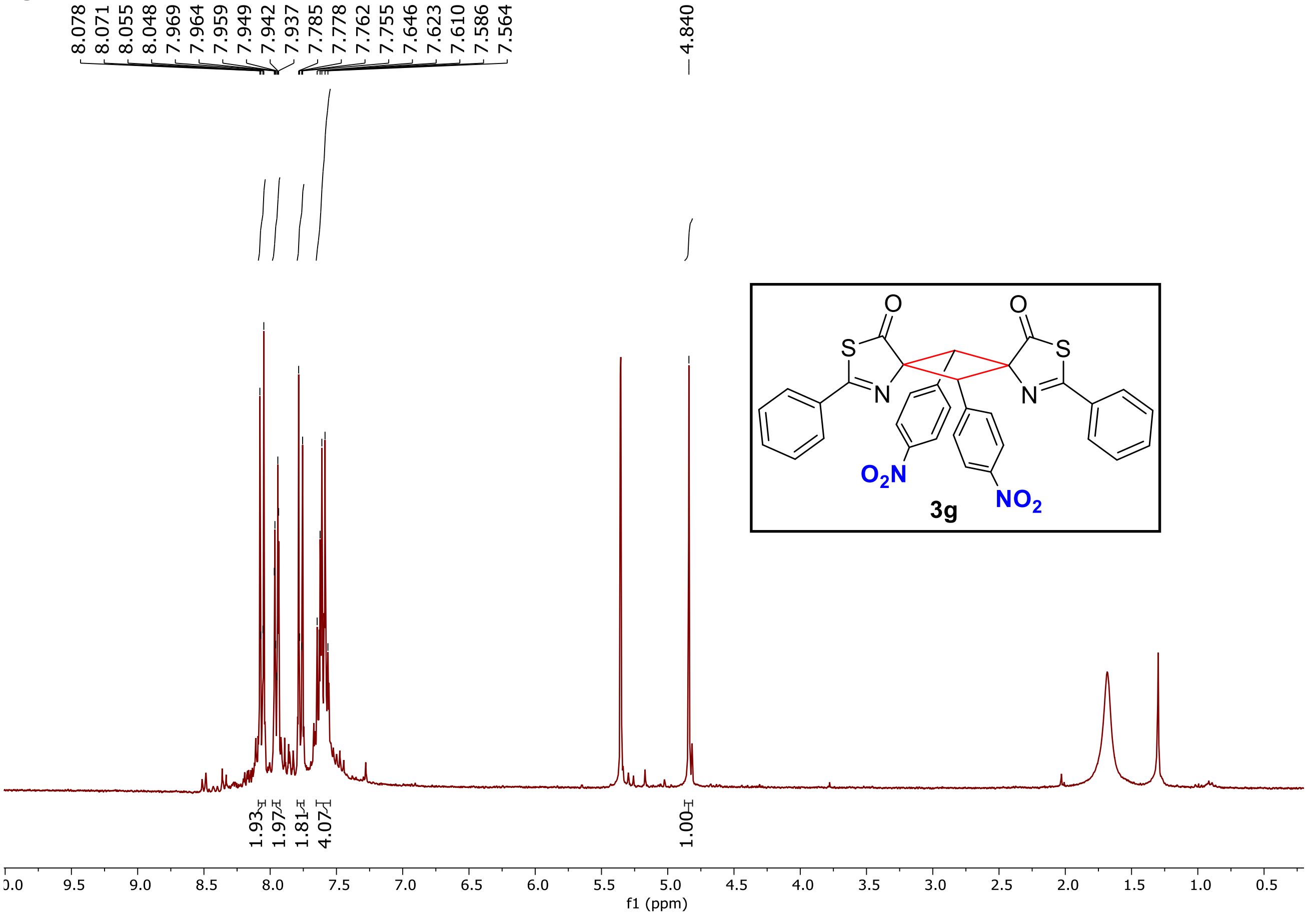

^{1}H NMR spectrum (CD_2Cl_2, 300.13 MHz) of **3g**

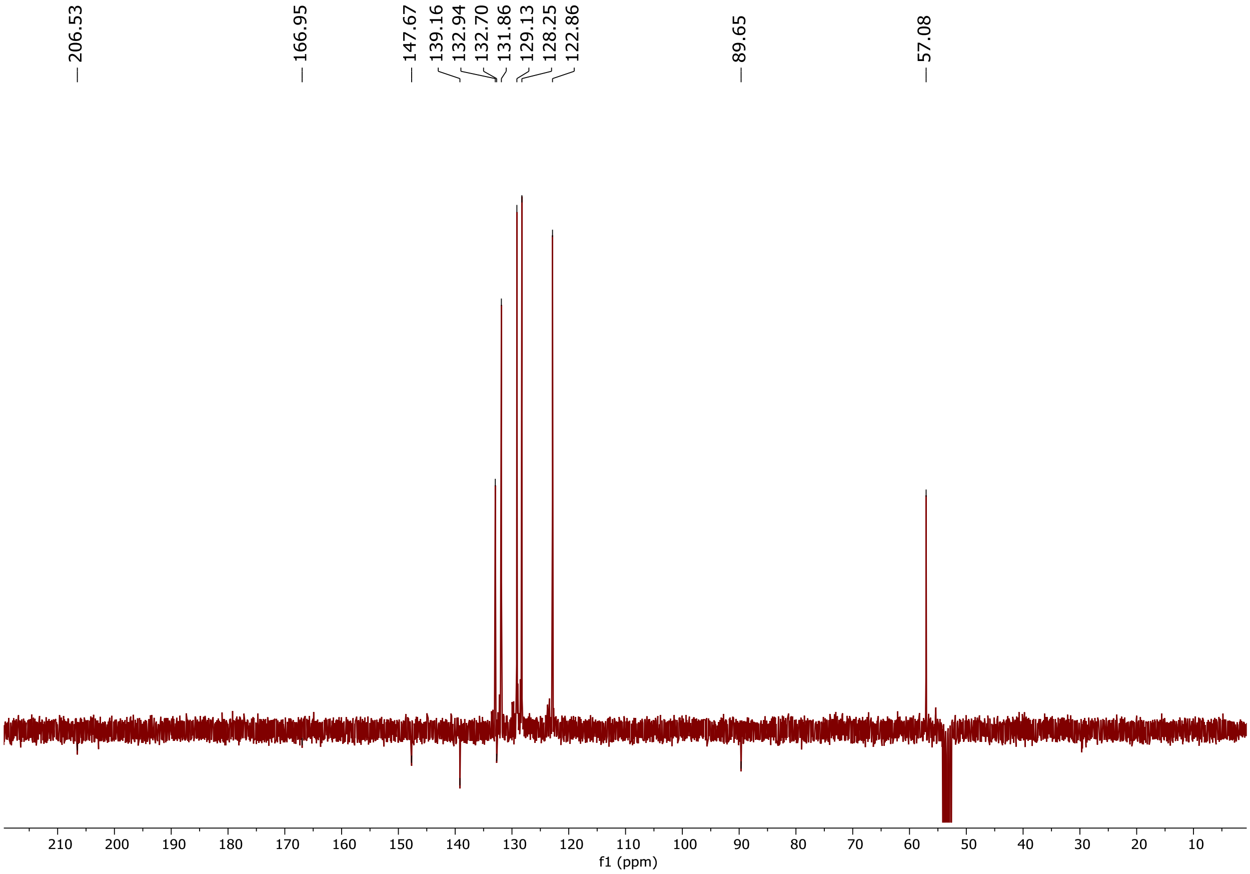

$^{13}C\{^{1}H\}$ NMR (APT) spectrum (CD_2Cl_2, 75.5 MHz) of **3g**

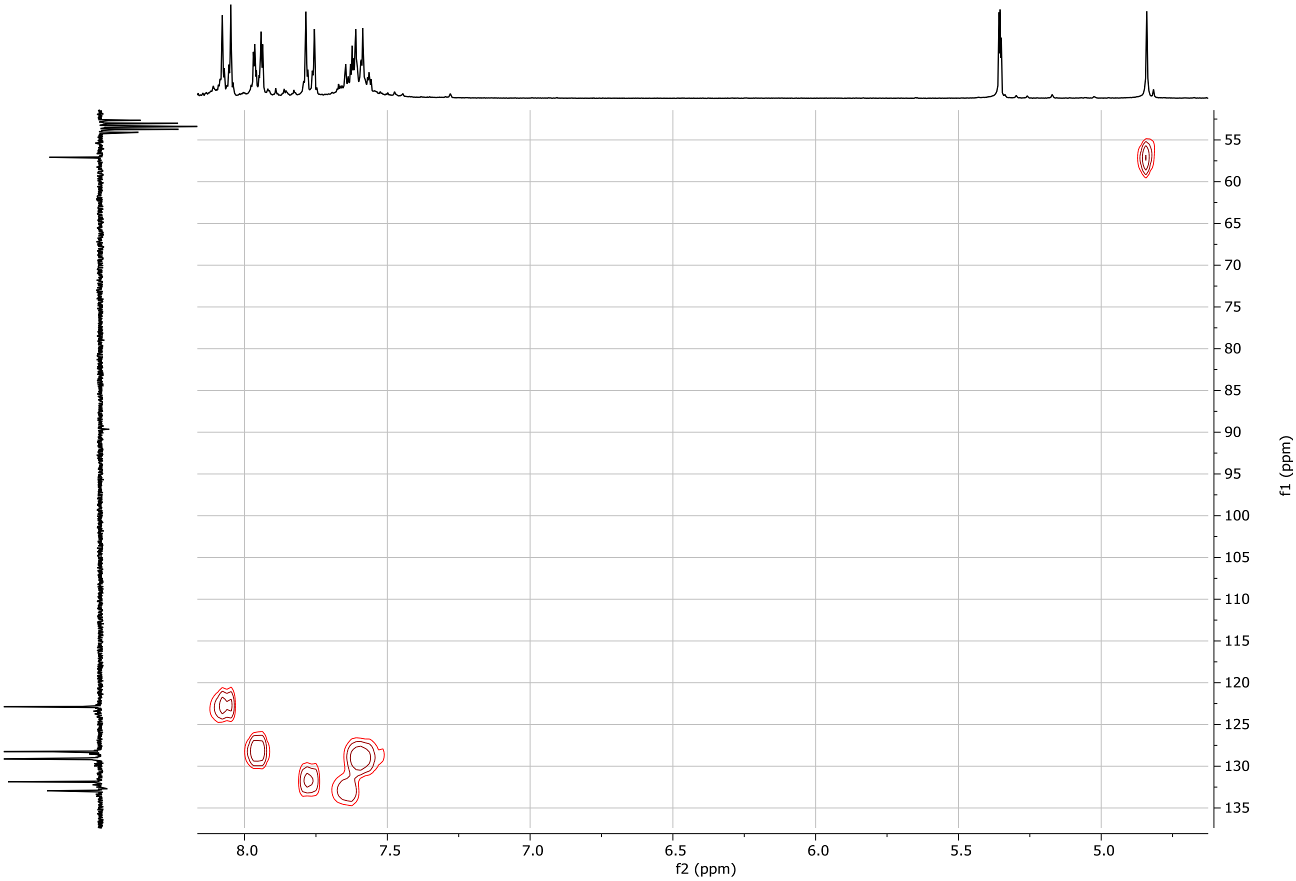

^{1}H-^{13}C HSQC correlation spectrum of **3g**

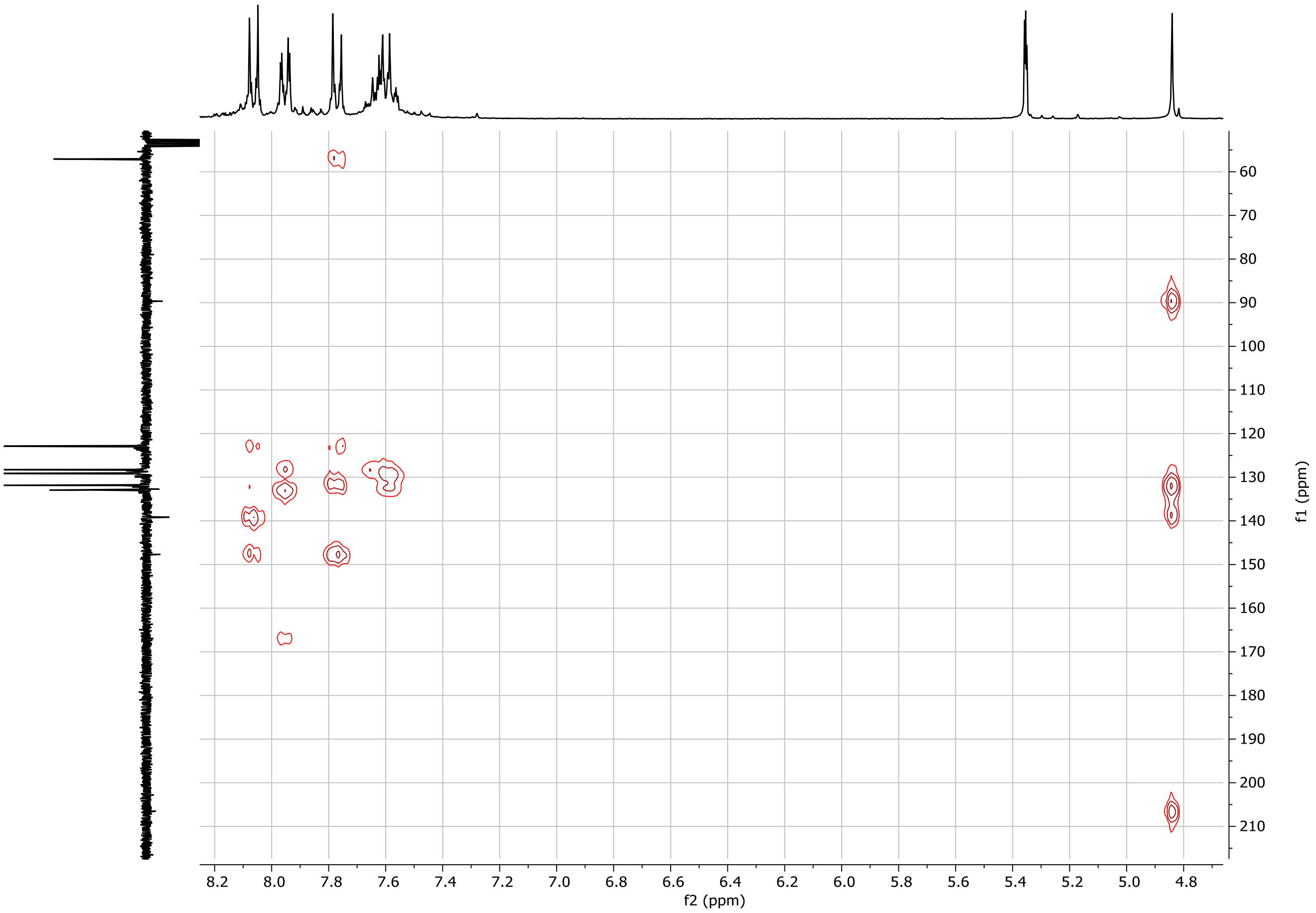

^1H-^{13}C HMBC correlation spectrum of **3g**

2,9-diphenyl-6,12-bis(4-(trifluoromethyl)phenyl)-3,10-dithia-1,8-diazadispiro[4.1.4^7.1^5]dodeca-1,8-diene-4,11-dione 3h

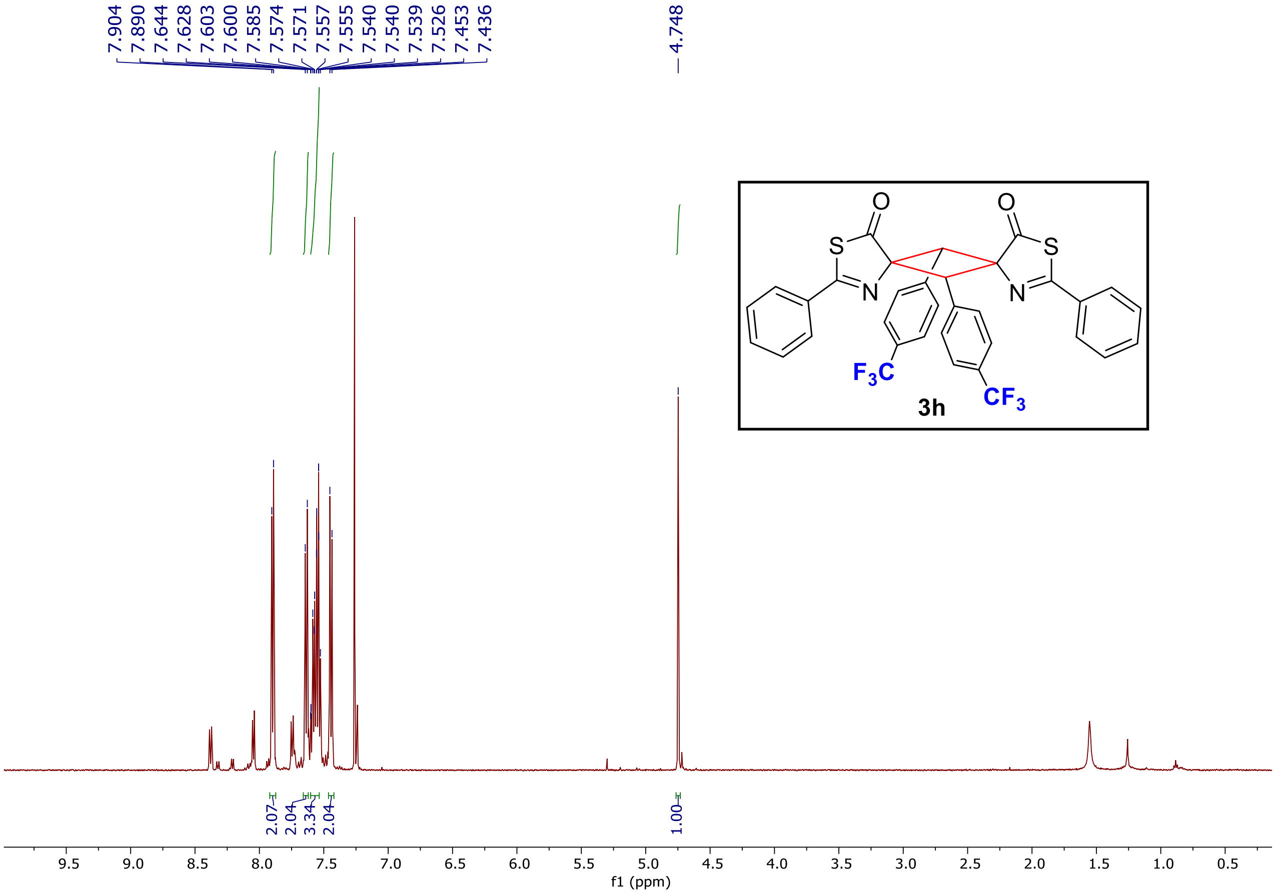

^1H NMR spectrum ($CDCl_3$, 500.13 MHz) of **3h**

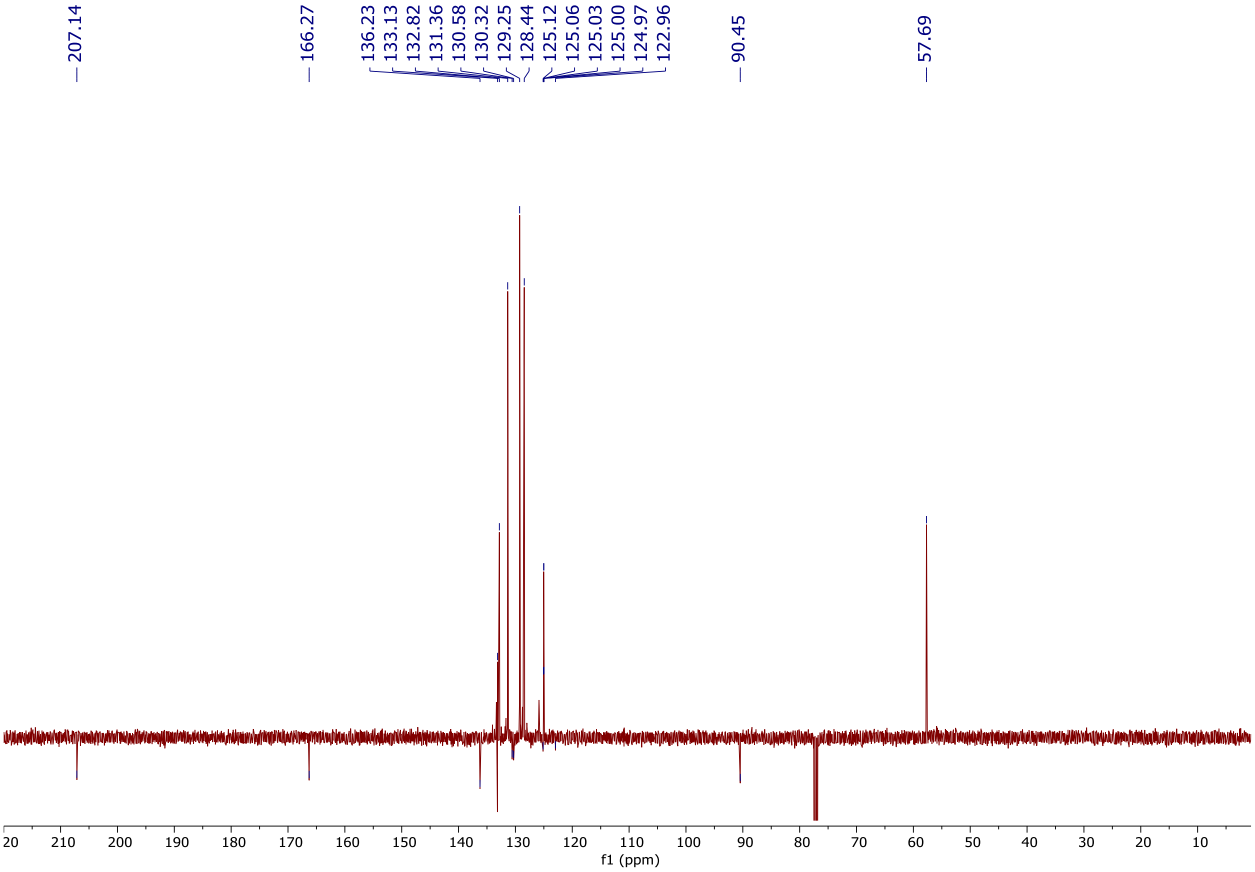

$^{13}C\{^1H\}$ NMR (APT) spectrum ($CDCl_3$, 125.76 MHz) of **3h**

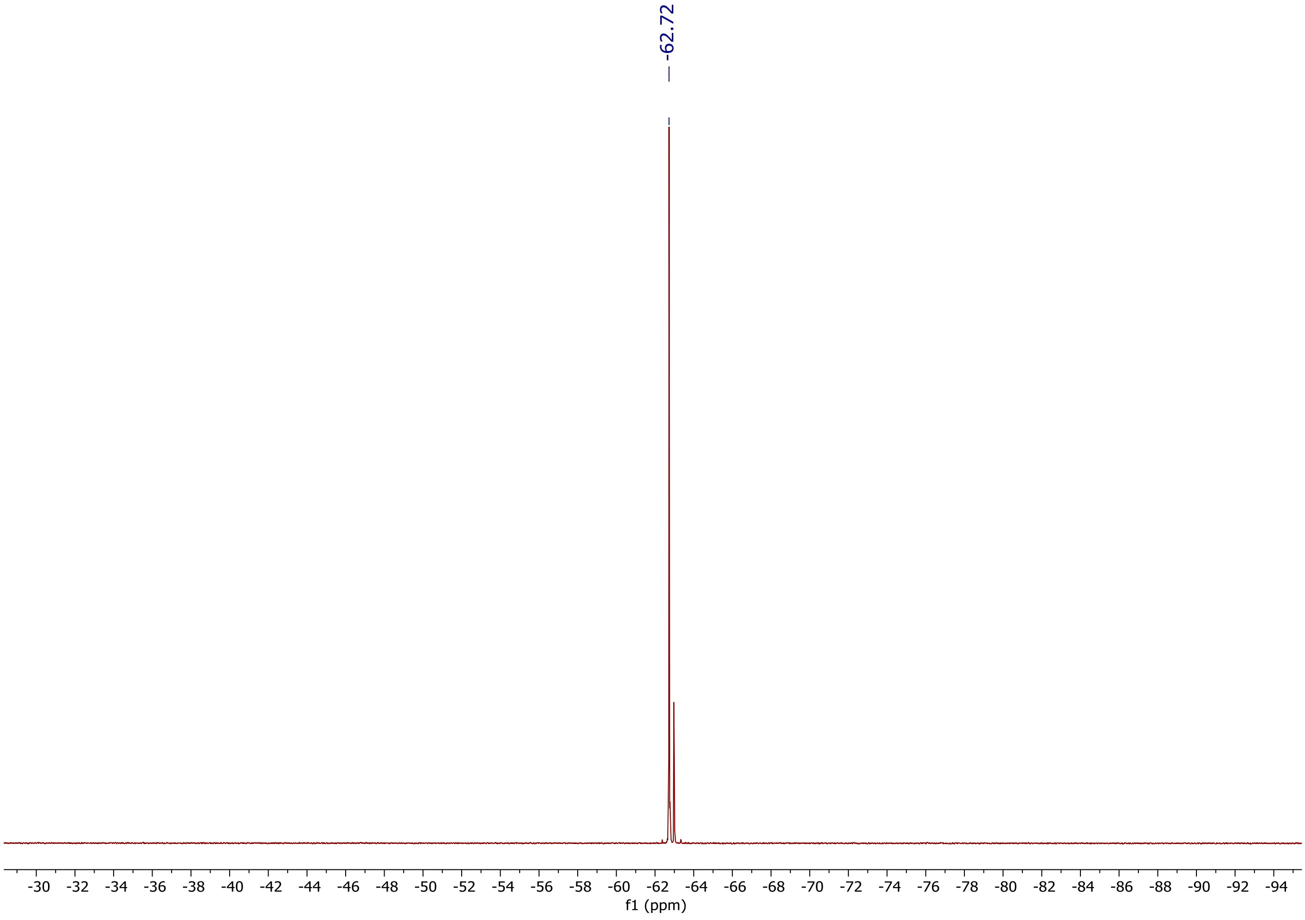

^{19}F NMR spectrum ($CDCl_3$, 282.4 MHz) of **3h**

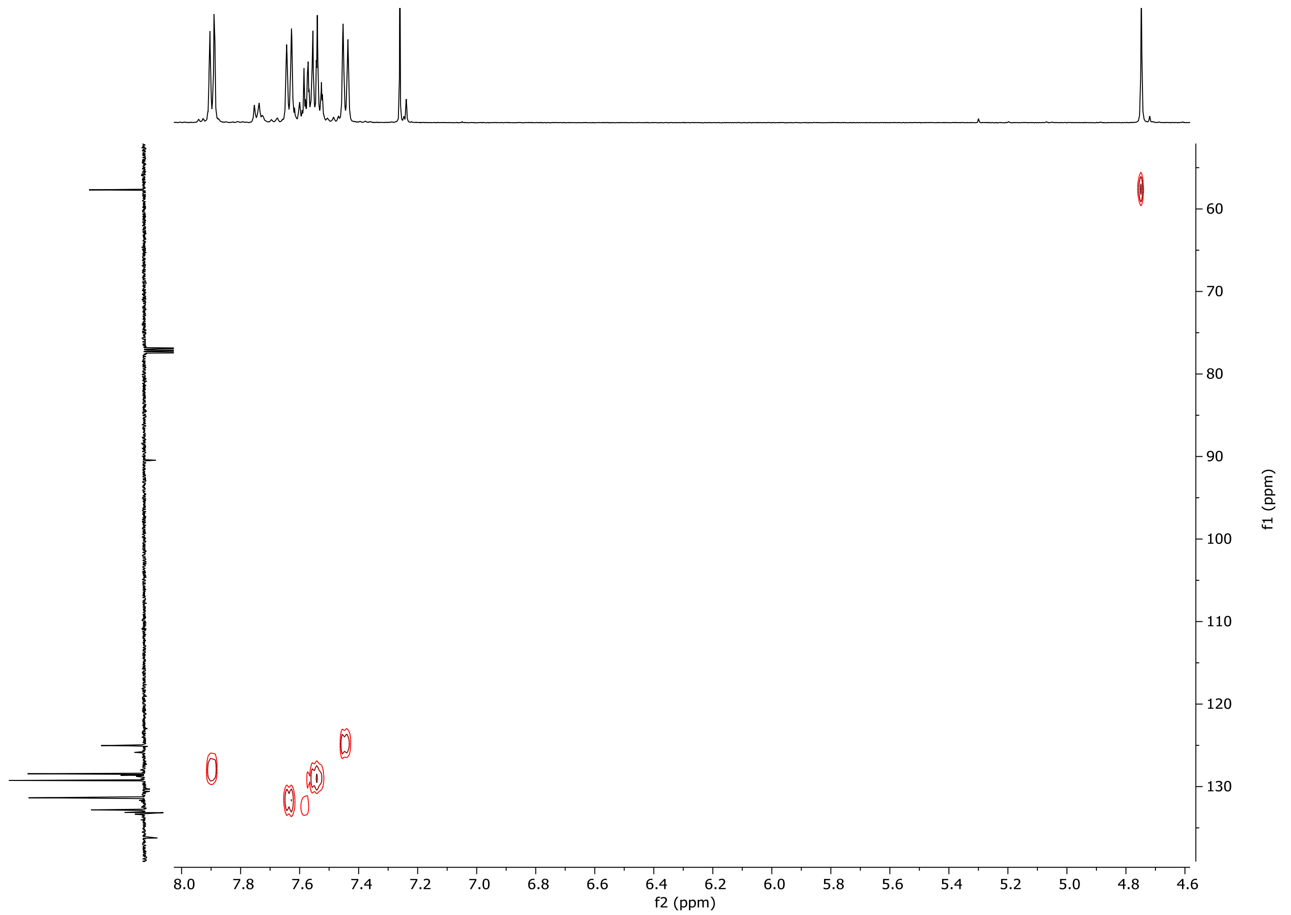

^{1}H-^{13}C HSQC correlation spectrum of **3h**

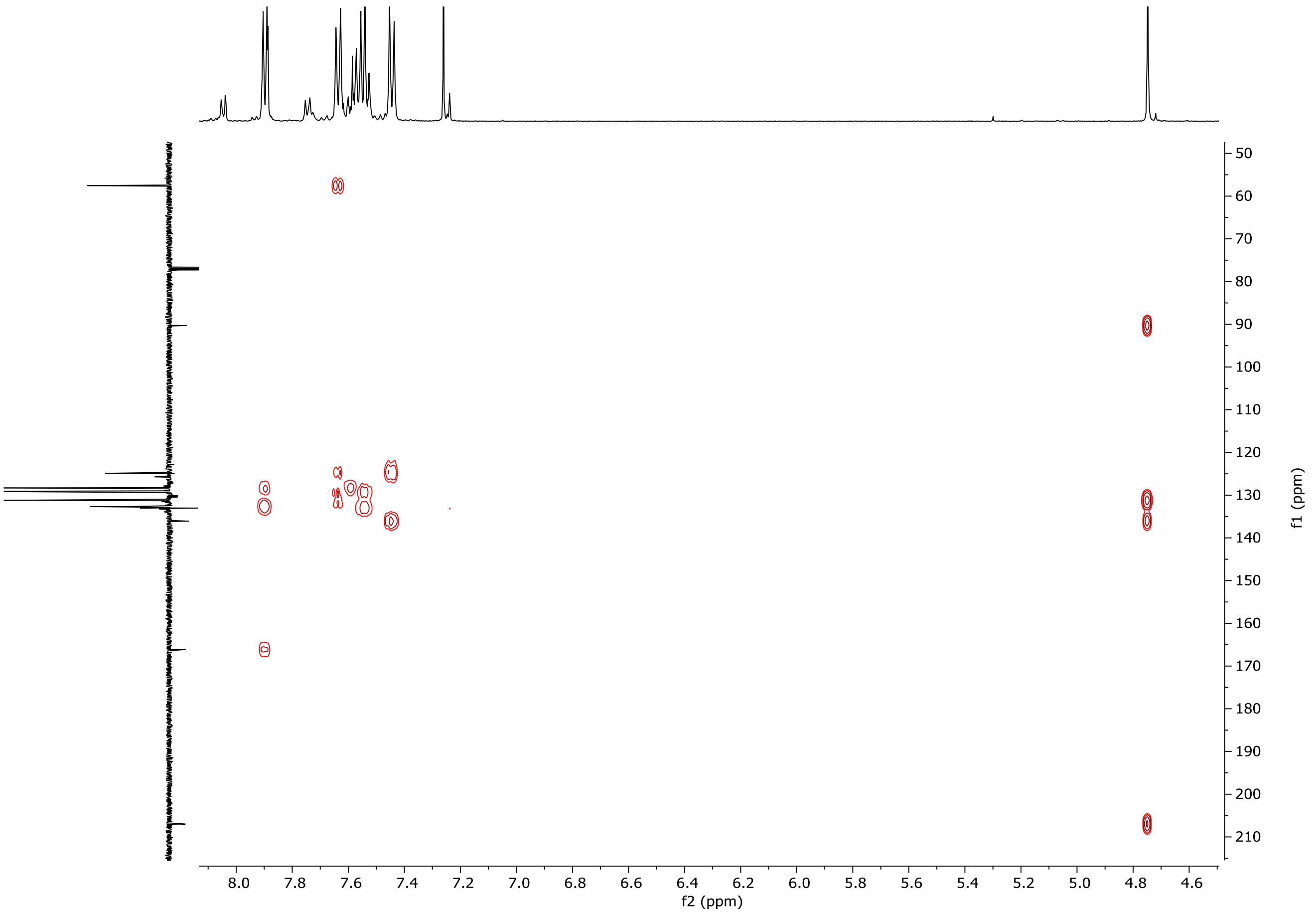

^{1}H-^{13}C HMBC correlation spectrum of **3h**

6,12-bis(2-chlorophenyl)-2,9-diphenyl-3,10-dithia-1,8-diazadispiro[4.1.4^{7}.1^{5}]dodeca-1,8-diene-4,11-dione 3j

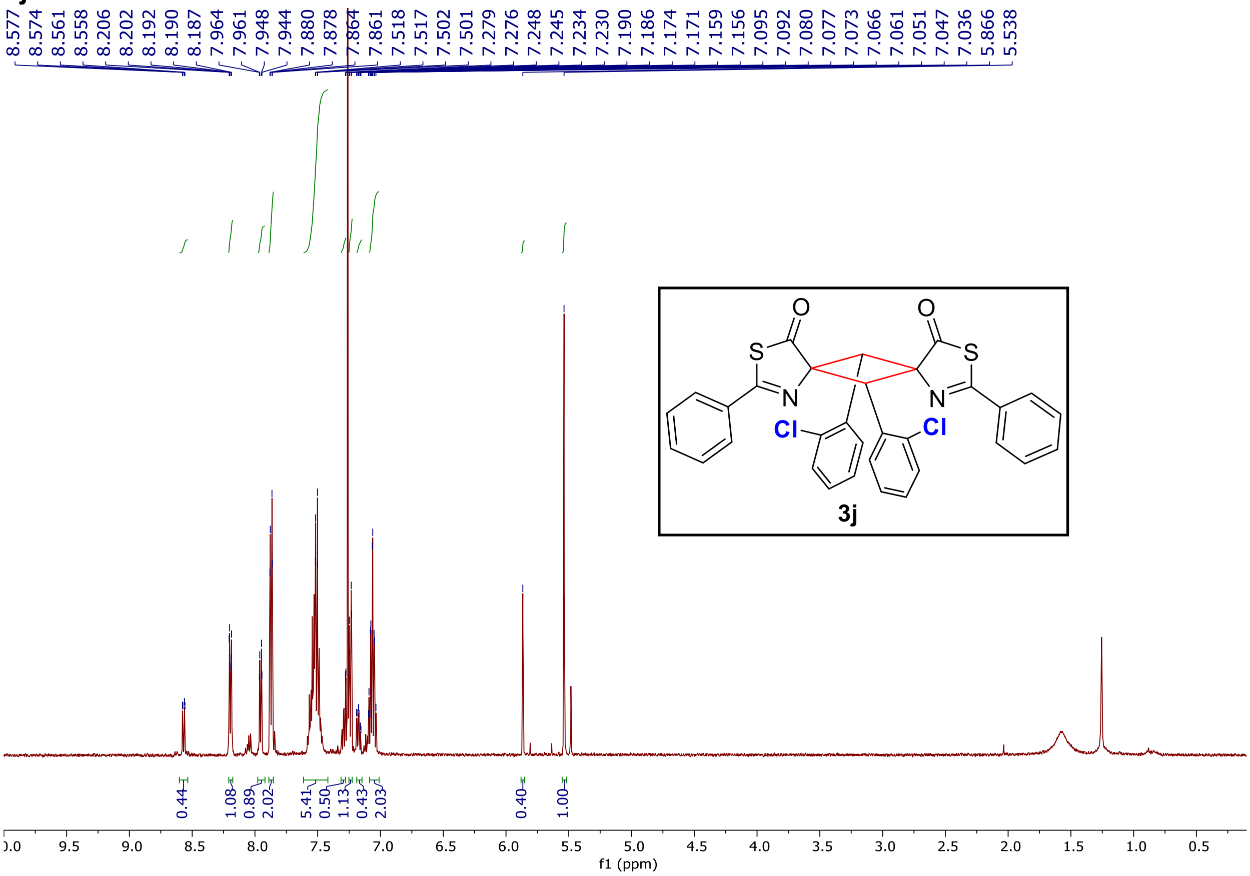

^{1}H NMR spectrum ($CDCl_3$, 500.13 MHz) of **3j**

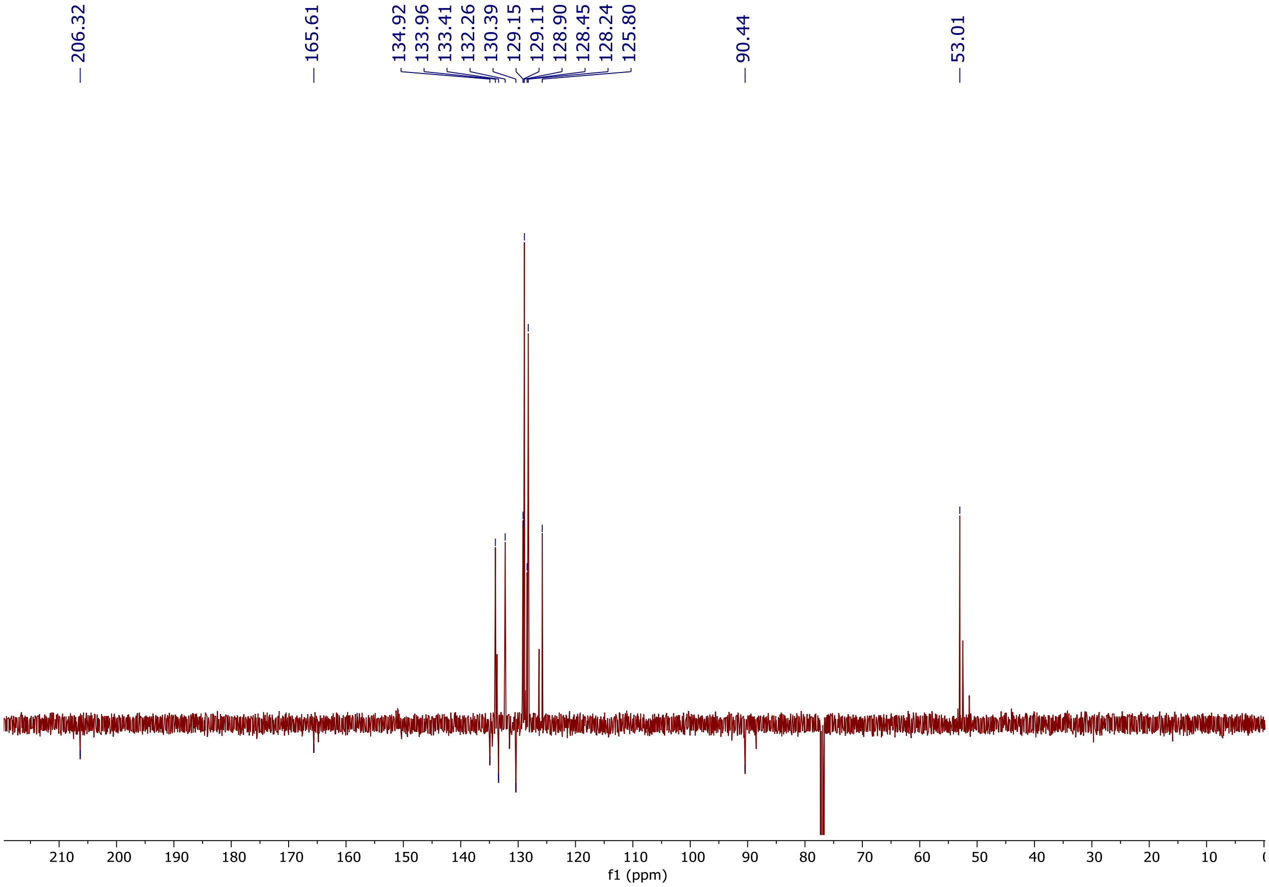

^{13}C{^{1}H} NMR (APT) spectrum ($CDCl_3$, 125.76 MHz) of **3j**

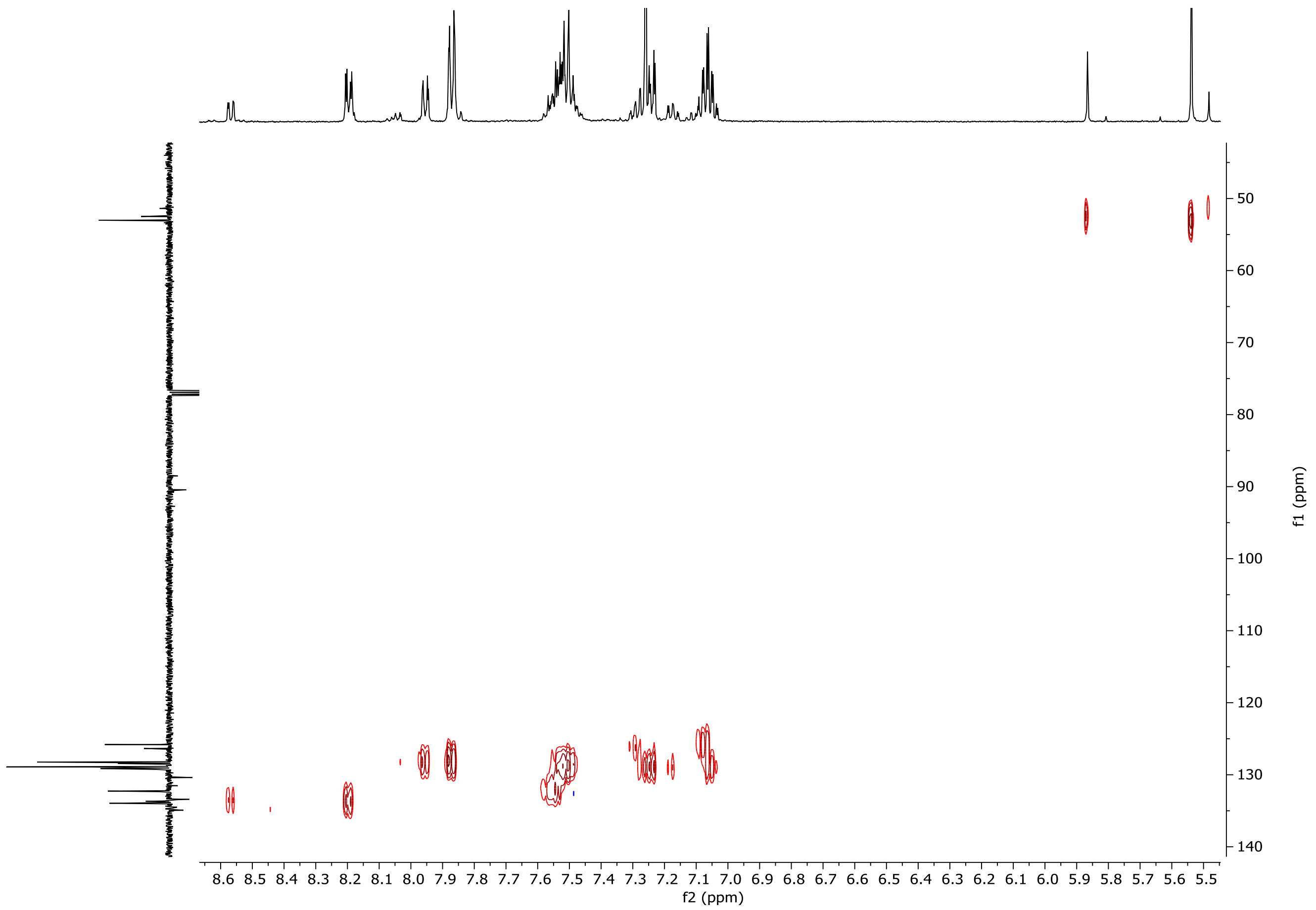

^{1}H-^{13}C HSQC correlation spectrum of **3j**

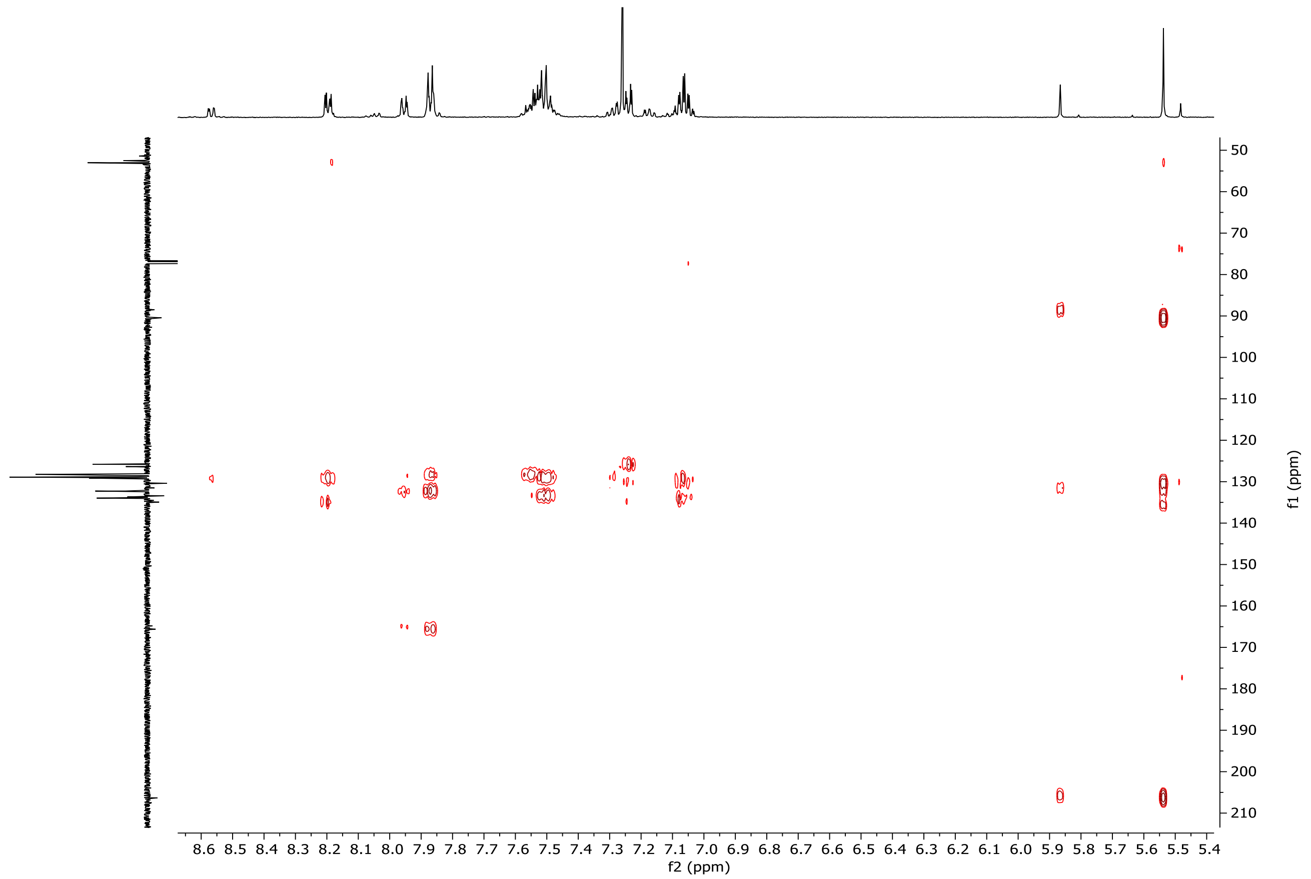

^{1}H-^{13}C HMBC correlation spectrum of **3j**

6,12-bis(2-bromophenyl)-2,9-diphenyl-3,10-dithia-1,8-diazadispiro[4.1.4^{7}.1^{5}]dodeca-1,8-diene-4,11-dione 3k

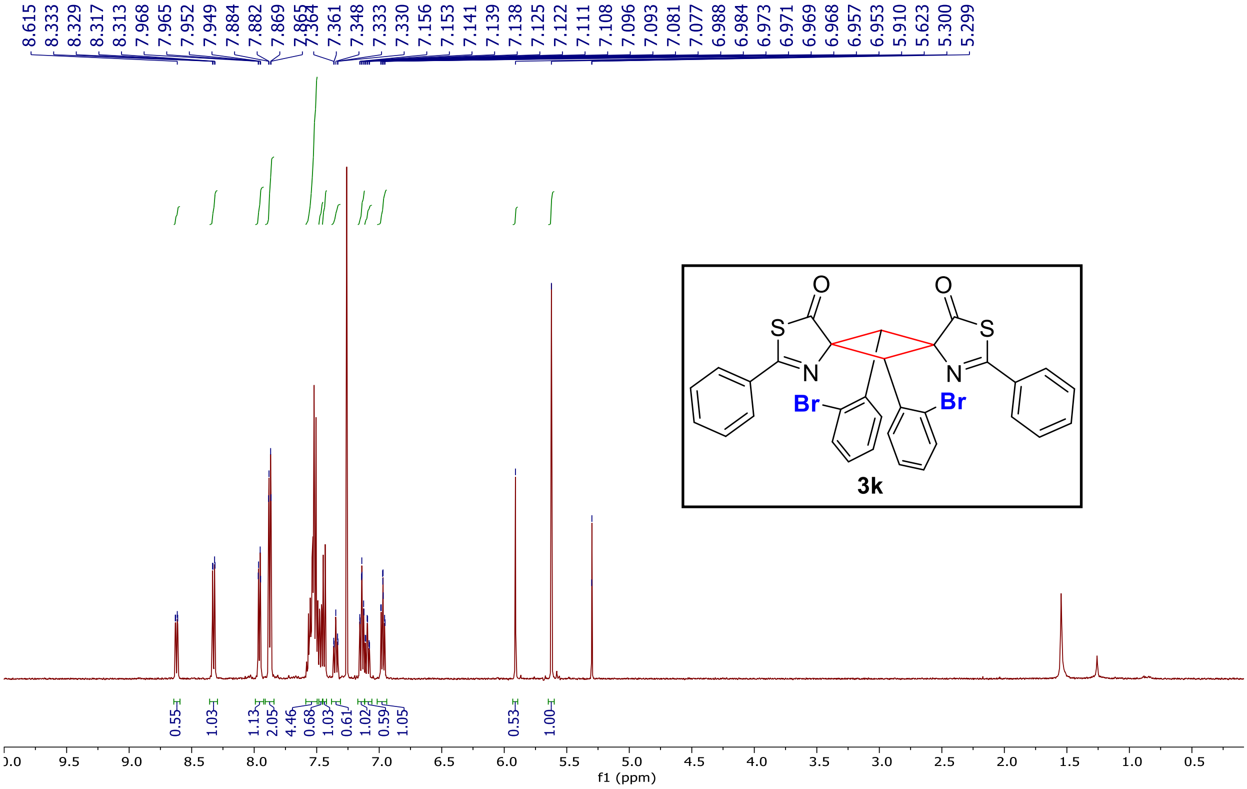

^{1}H NMR spectrum ($CDCl_3$, 500.13 MHz) of **3k**

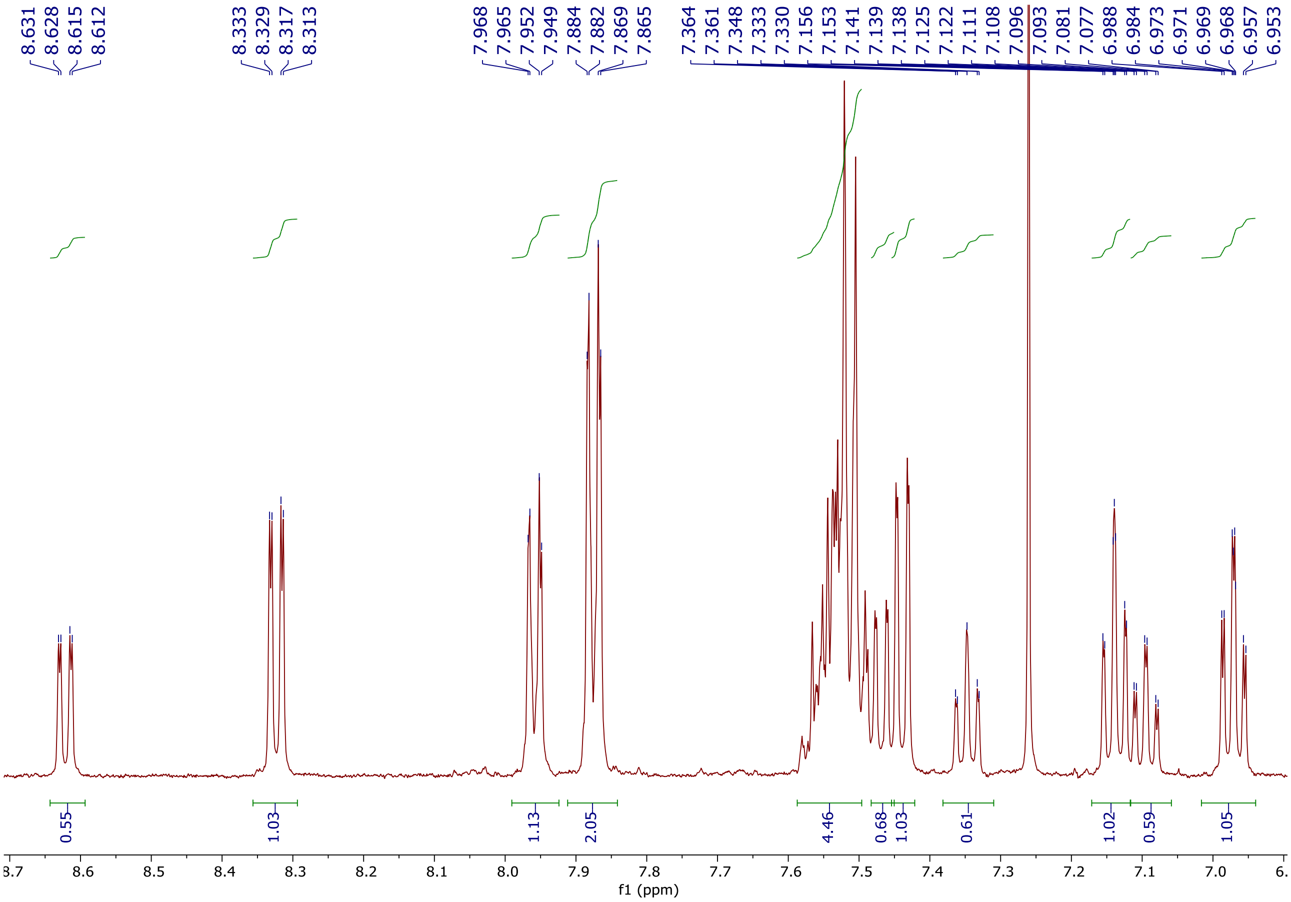

^{1}H NMR spectrum ($CDCl_3$, 500.13 MHz) of **3k** (zoom aromatic region)

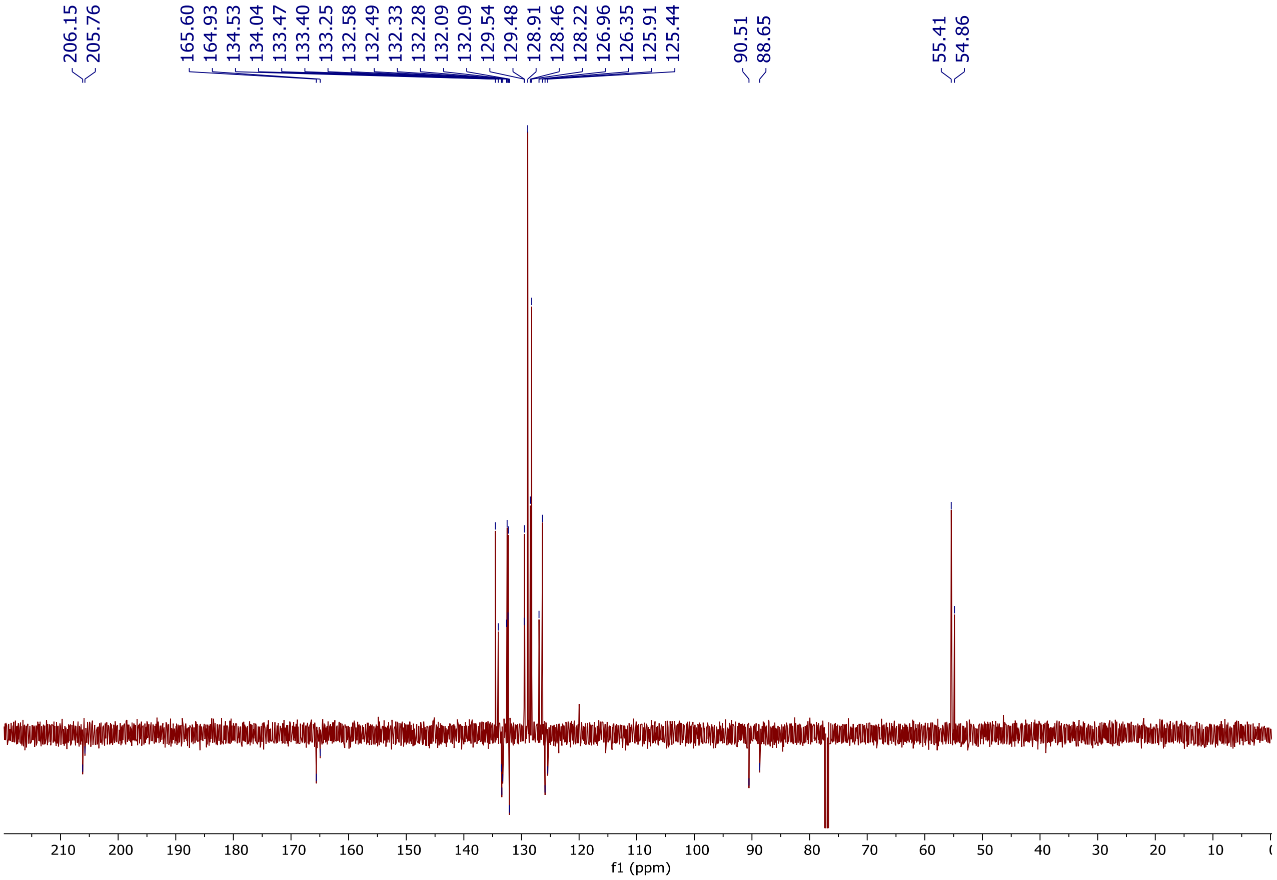

$^{13}C\{^1H\}$ NMR (APT) spectrum ($CDCl_3$, 125.76 MHz) of **3k**

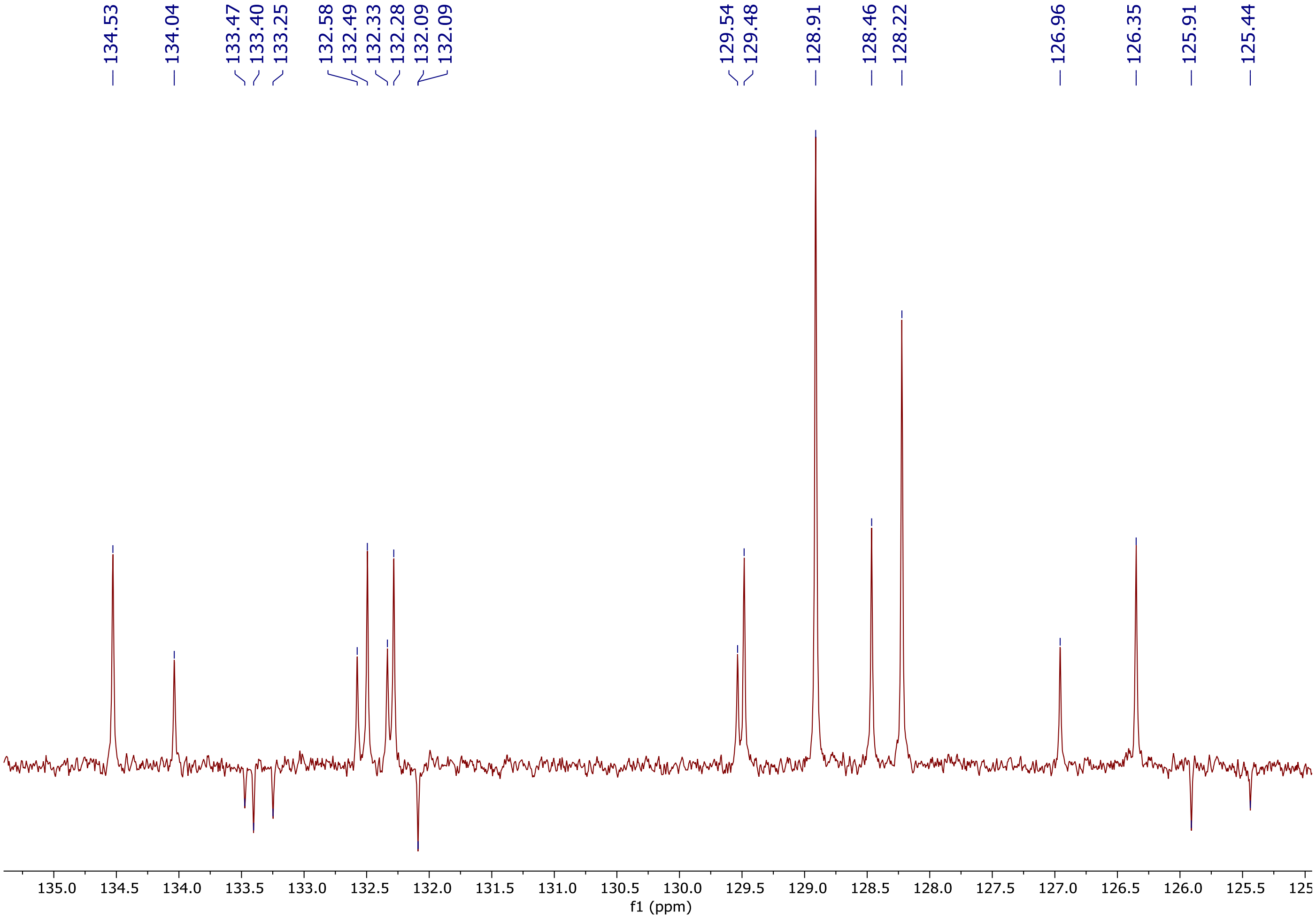

$^{13}C\{^1H\}$ NMR (APT) spectrum ($CDCl_3$, 125.76 MHz) of **3k** (zoom aromatic region)

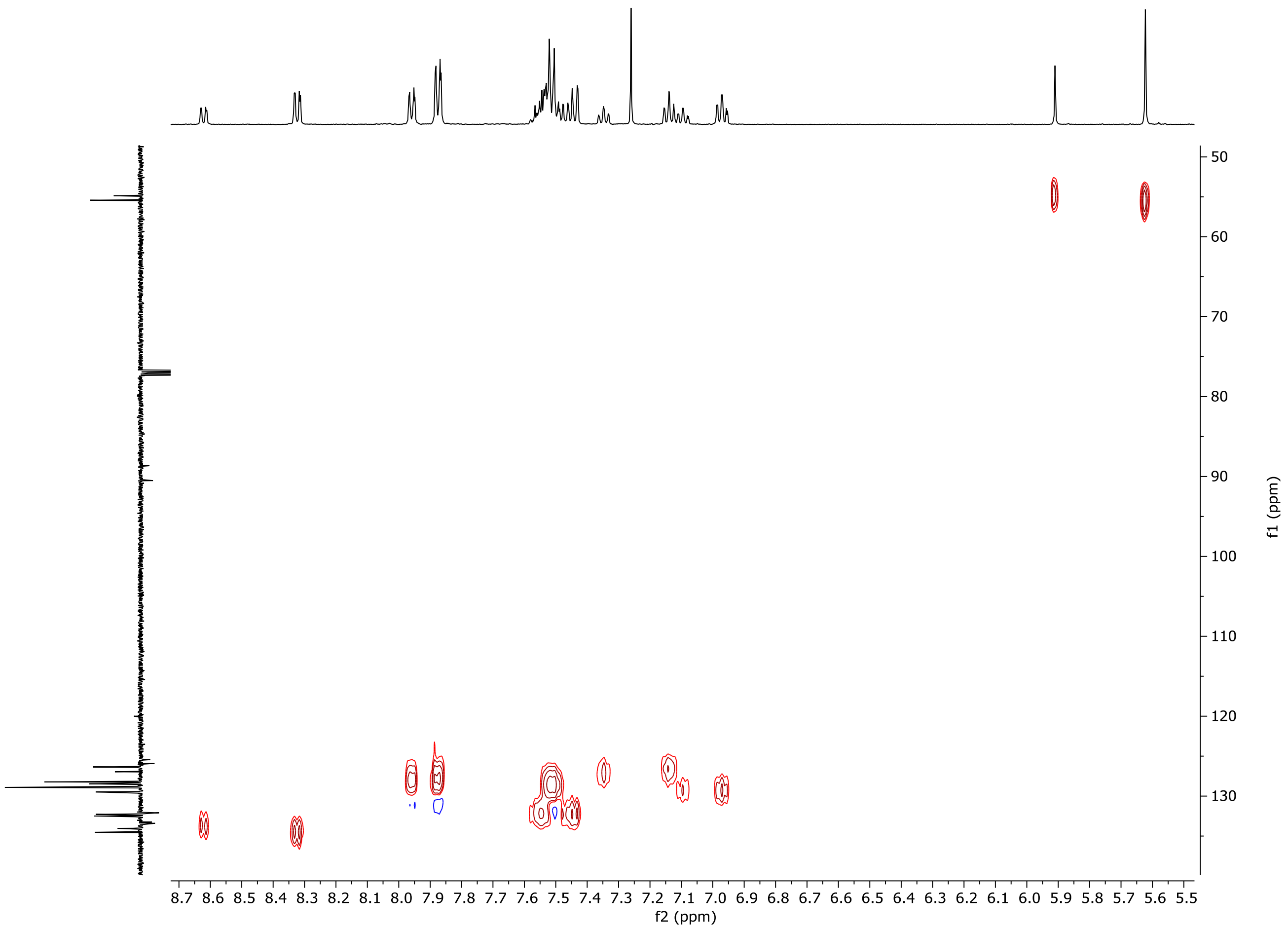

^{1}H-^{13}C HSQC correlation spectrum of **3k**

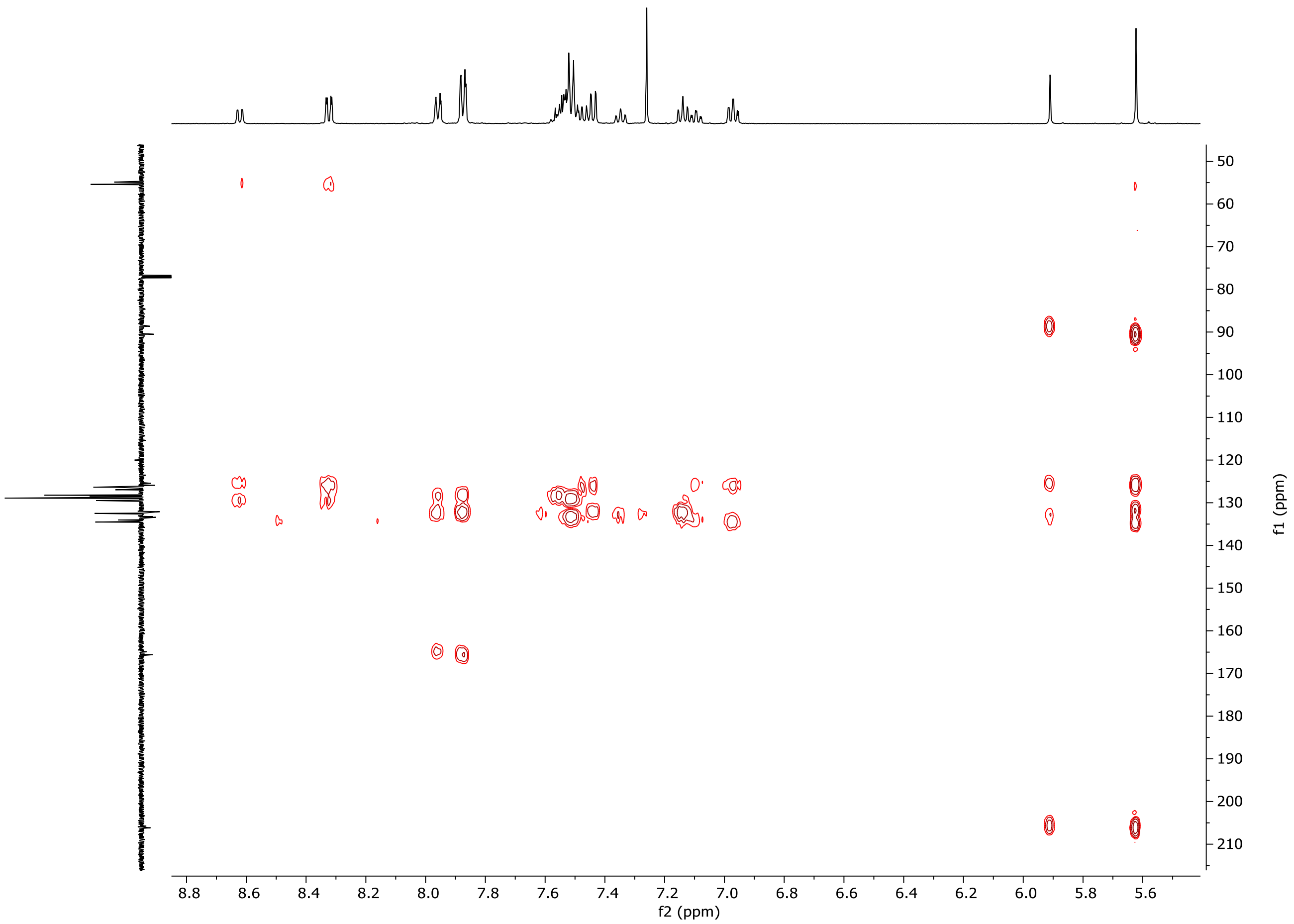

^{1}H-^{13}C HMBC correlation spectrum of **3k**

6,12-bis(3,4-dimethylphenyl)-2,9-diphenyl-3,10-dithia-1,8-diazadispiro[$4.1.4^{7}.1^{5}$]dodeca-1,8-diene-4,11-dione 3m

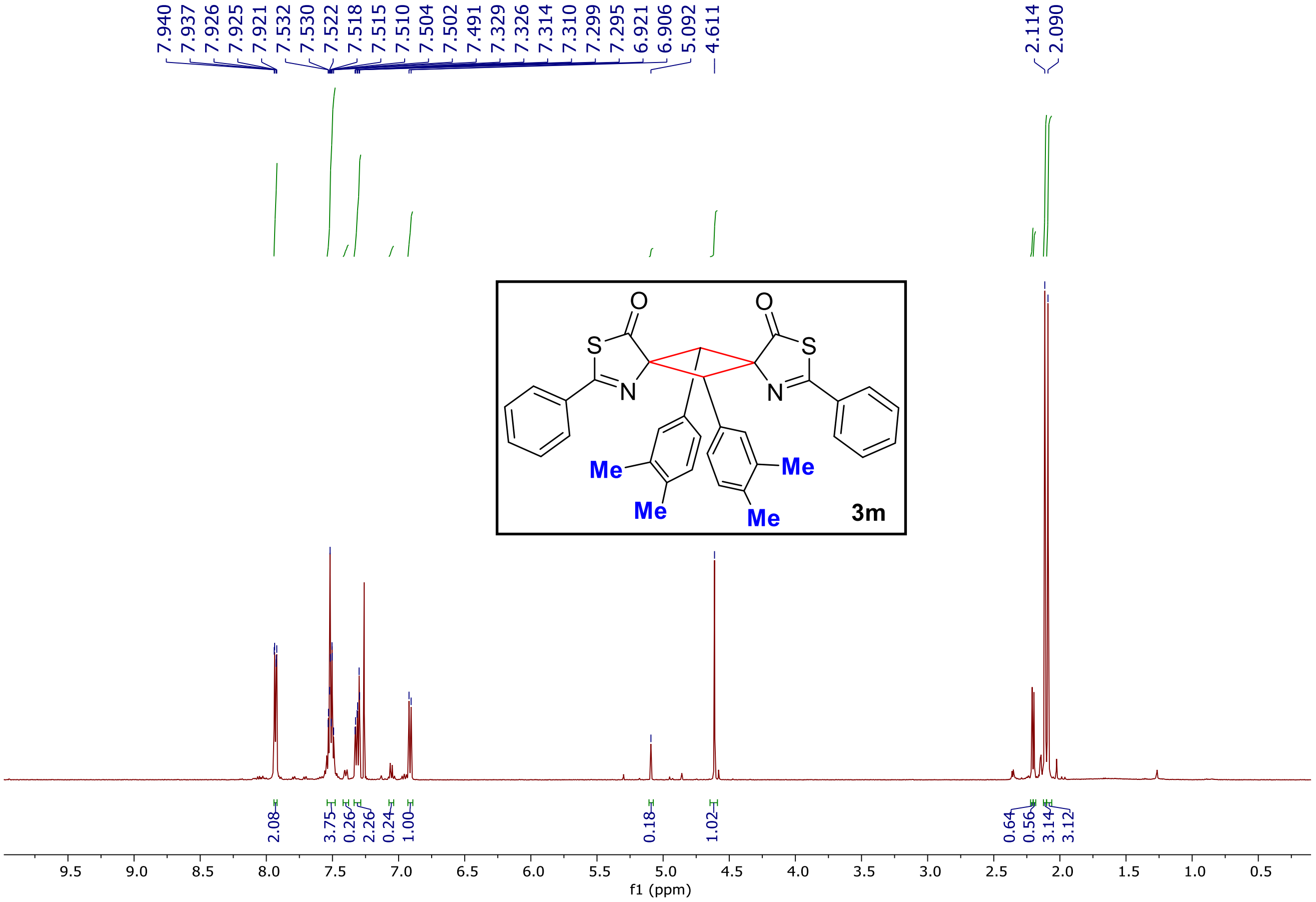

^{1}H NMR spectrum ($CDCl_3$, 500.13 MHz) of **3m**

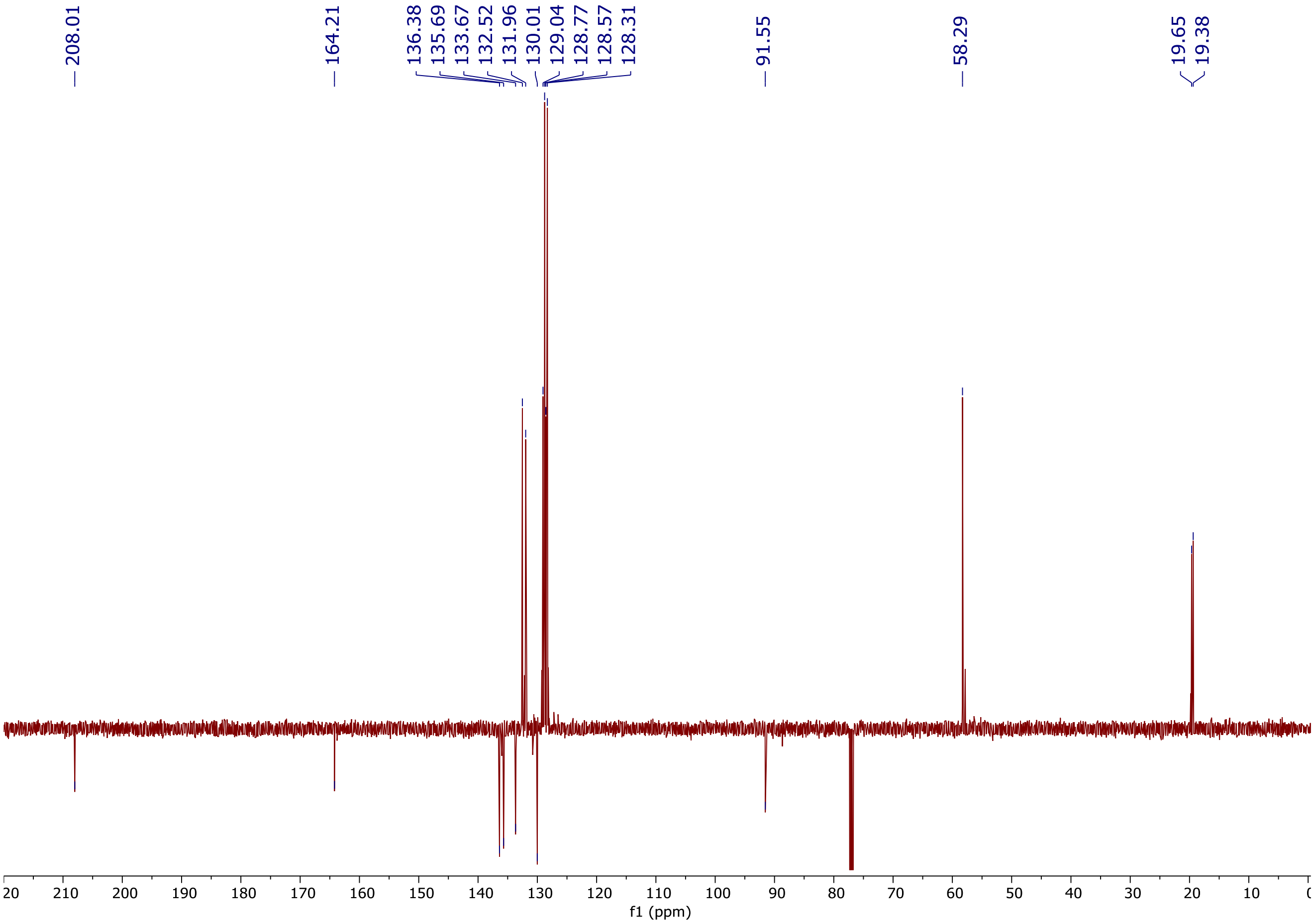

^{13}C{^{1}H} NMR (APT) spectrum ($CDCl_3$, 125.76 MHz) of **3m**

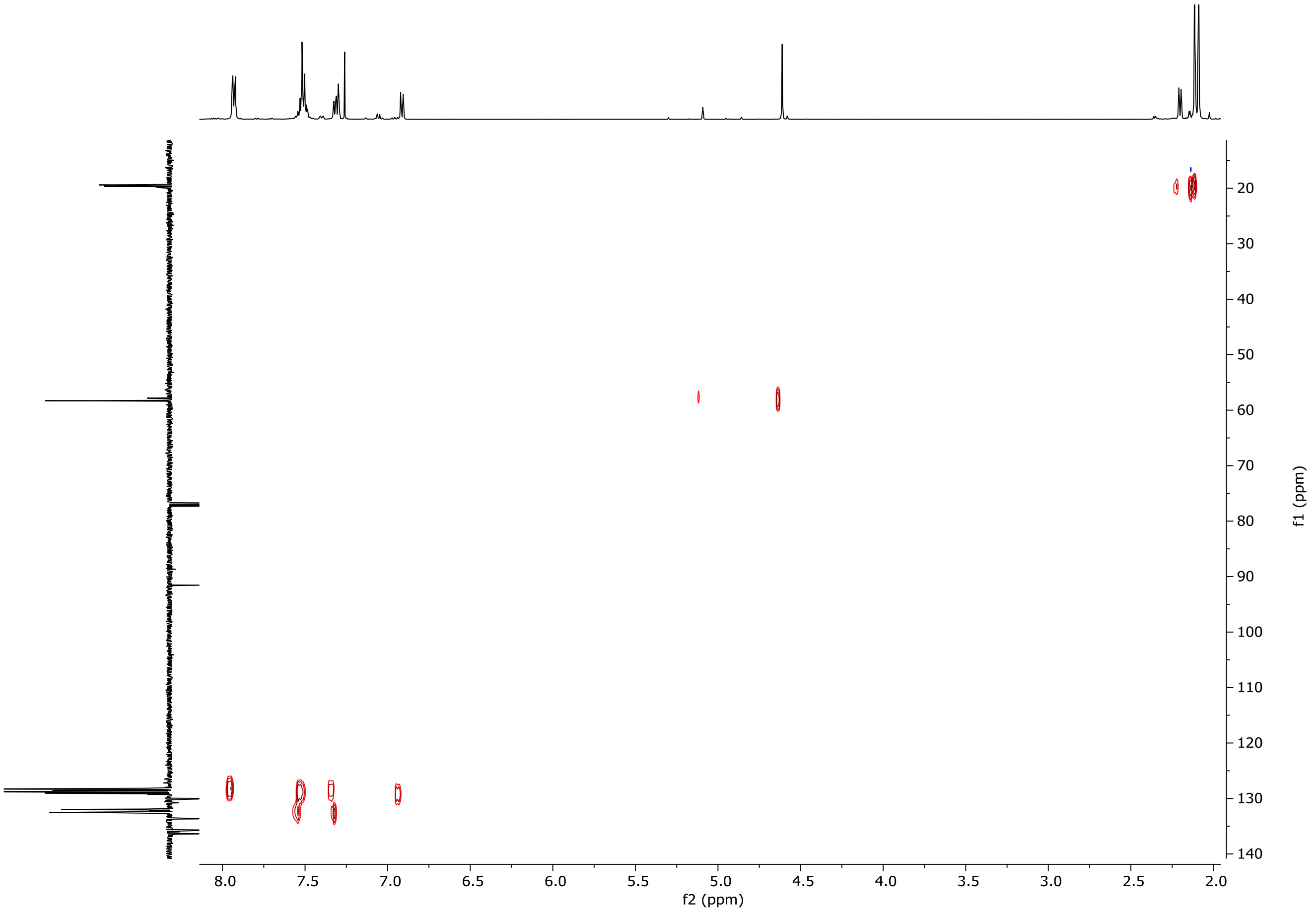

^{1}H-^{13}C HSQC correlation spectrum of **3m**

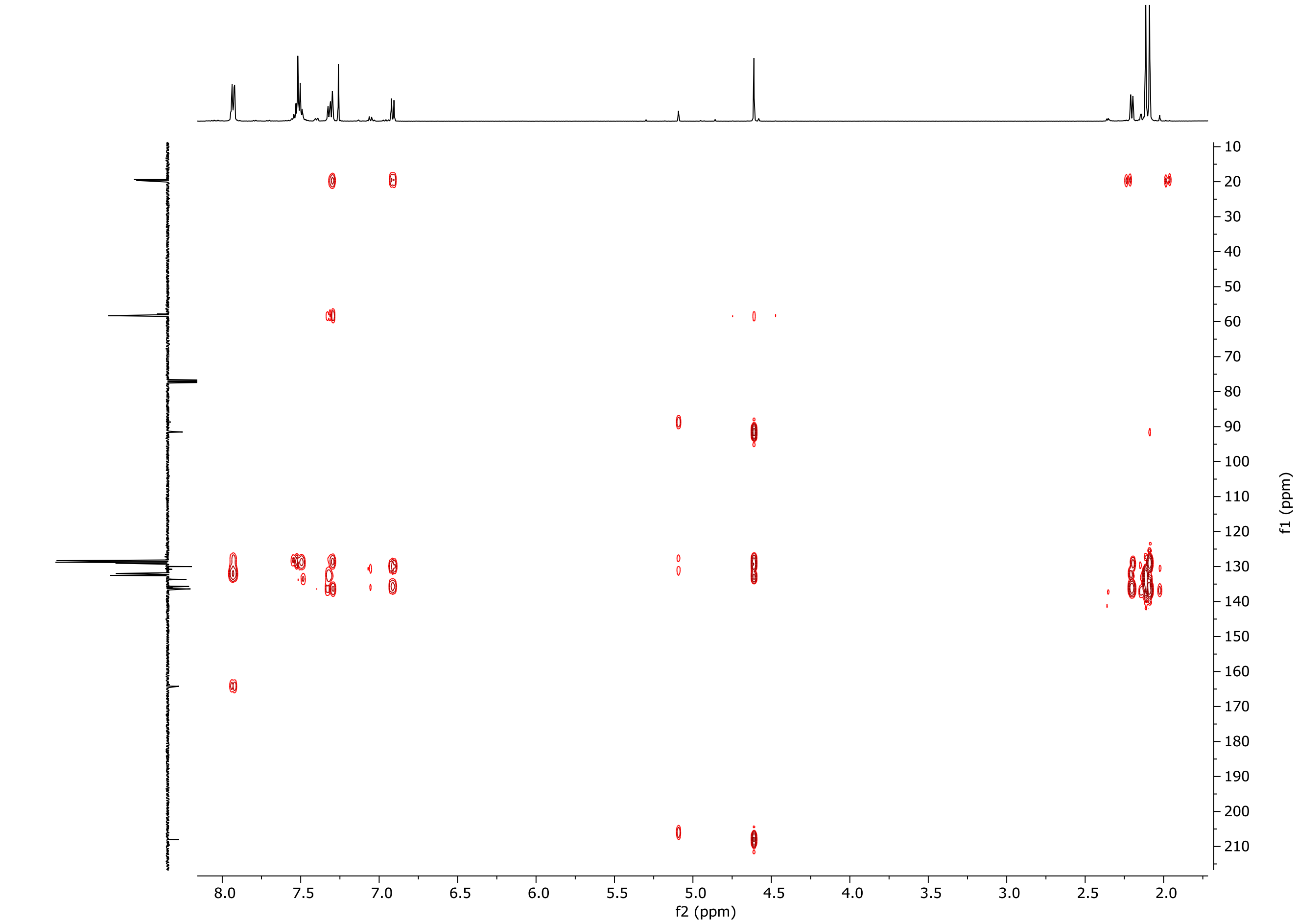

^{1}H-^{13}C HMBC correlation spectrum of **3m**

6,12-bis(3,4-dichlorophenyl)-2,9-diphenyl-3,10-dithia-1,8-diazadispiro[4.1.4^7.1^5]dodeca-1,8-diene-4,11-dione 3n

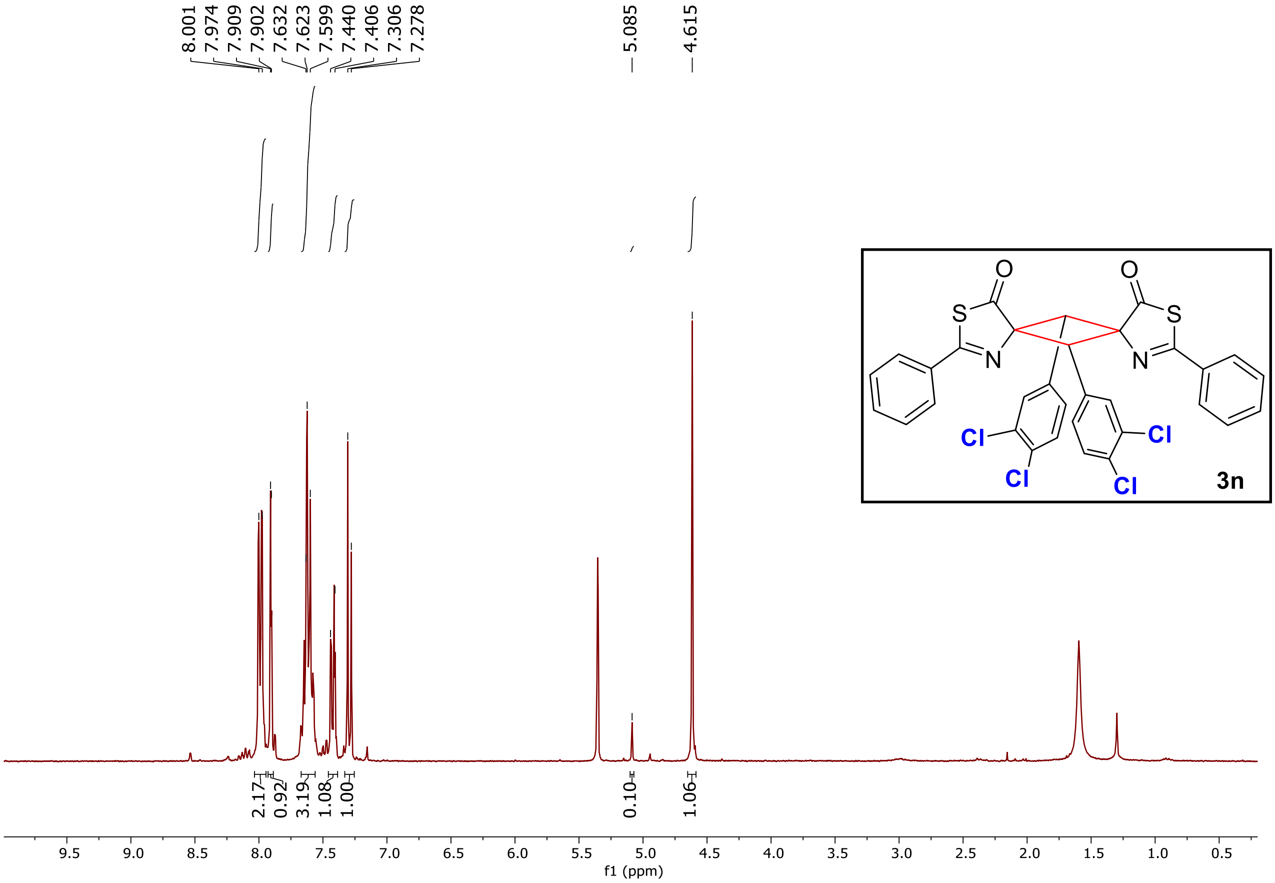

^{1}H NMR spectrum (CD_2Cl_2, 300.13 MHz) of **3n**

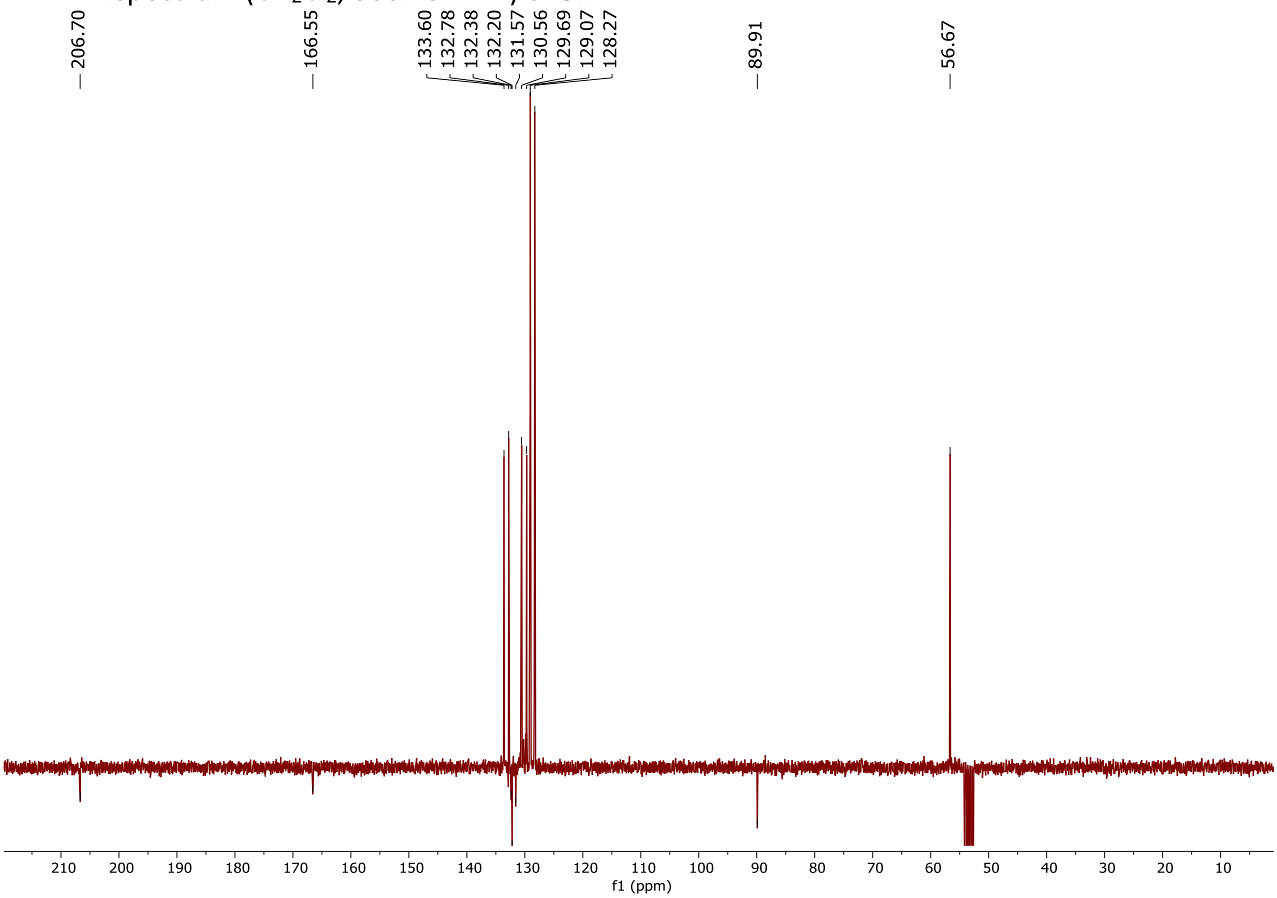

$^{13}C\{^{1}H\}$ NMR (APT) spectrum (CD_2Cl_2, 75.5 MHz) of **3n**

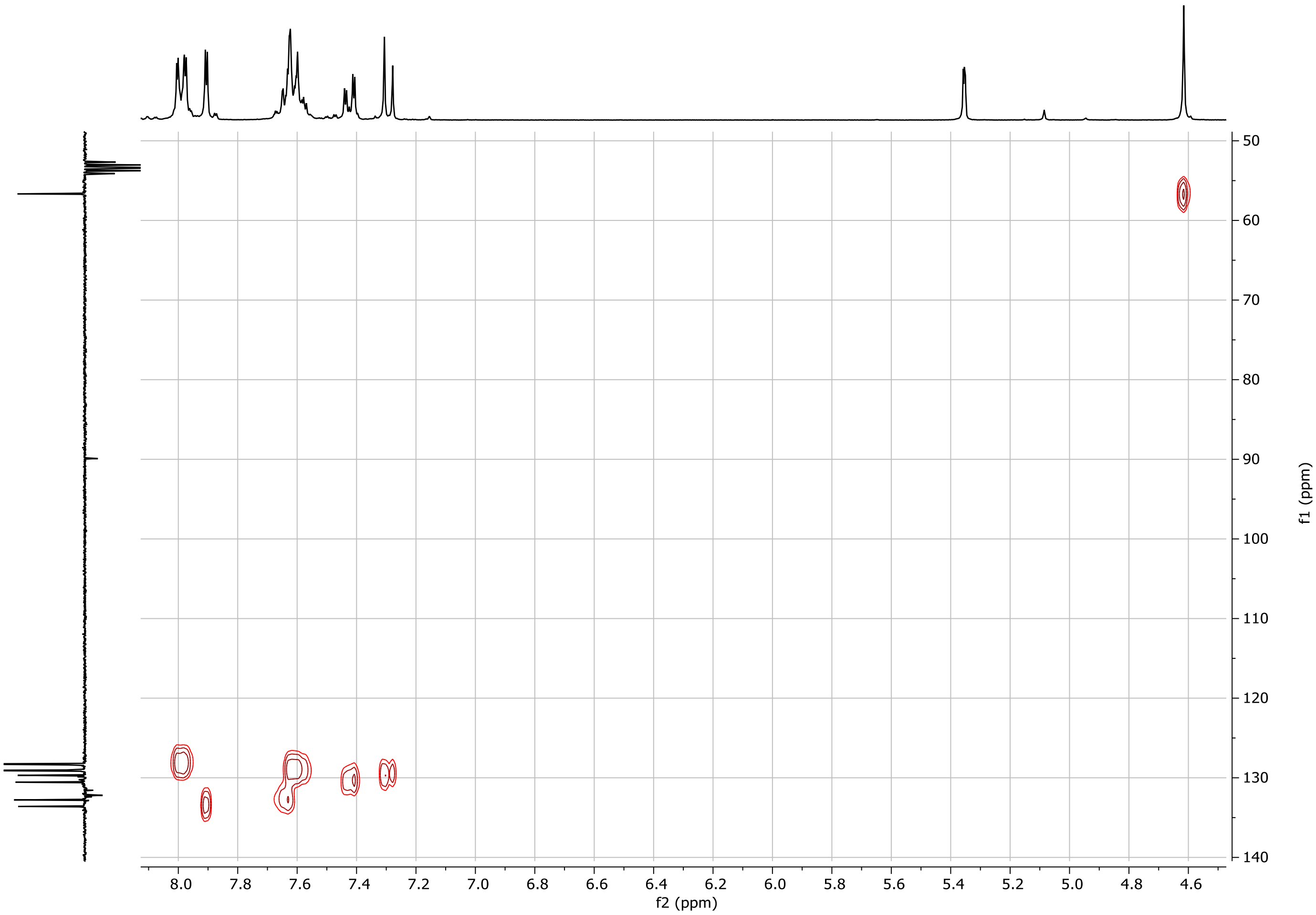

^{1}H-^{13}C HSQC correlation spectrum of **3n**

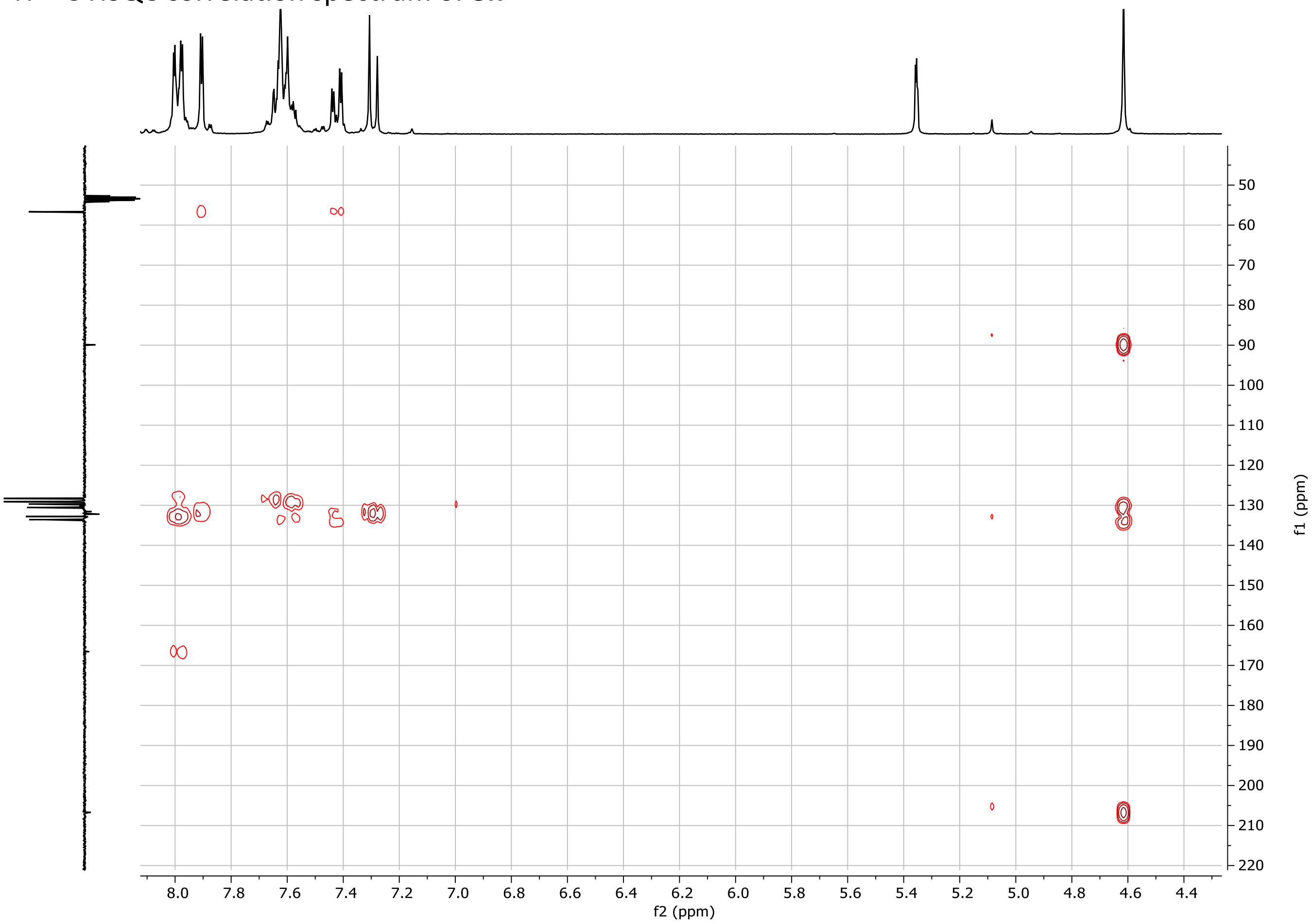

^{1}H-^{13}C HSQC correlation spectrum of **3n**

6,12-bis(3,4-difluorophenyl)-2,9-diphenyl-3,10-dithia-1,8-diazadispiro[4.1.4^{7}.1^{5}]dodeca-1,8-diene-4,11-dione 3o

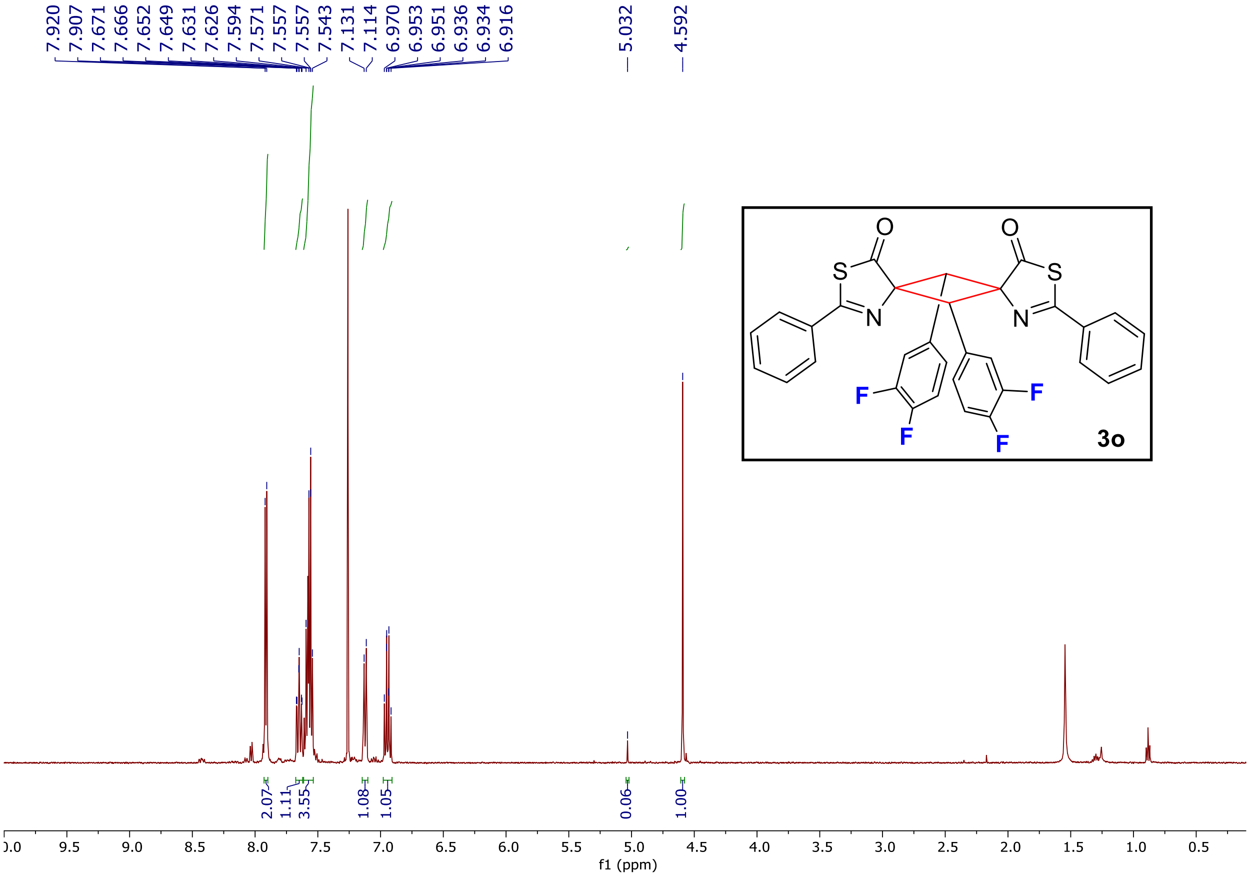

^{1}H NMR spectrum ($CDCl_3$, 500.13 MHz) of **3o**

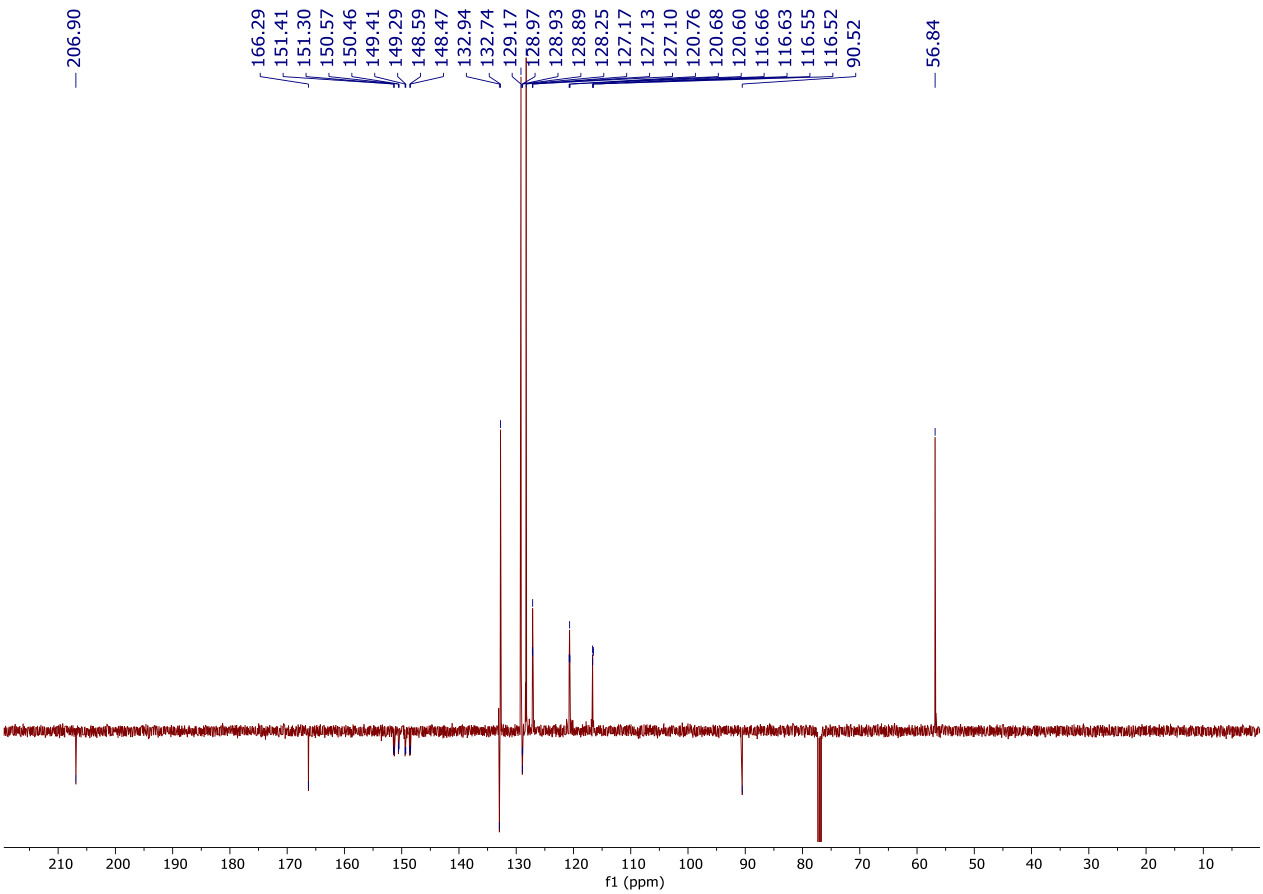

^{13}C{^{1}H} NMR spectrum ($CDCl_3$, 125.76 MHz) of **3o**

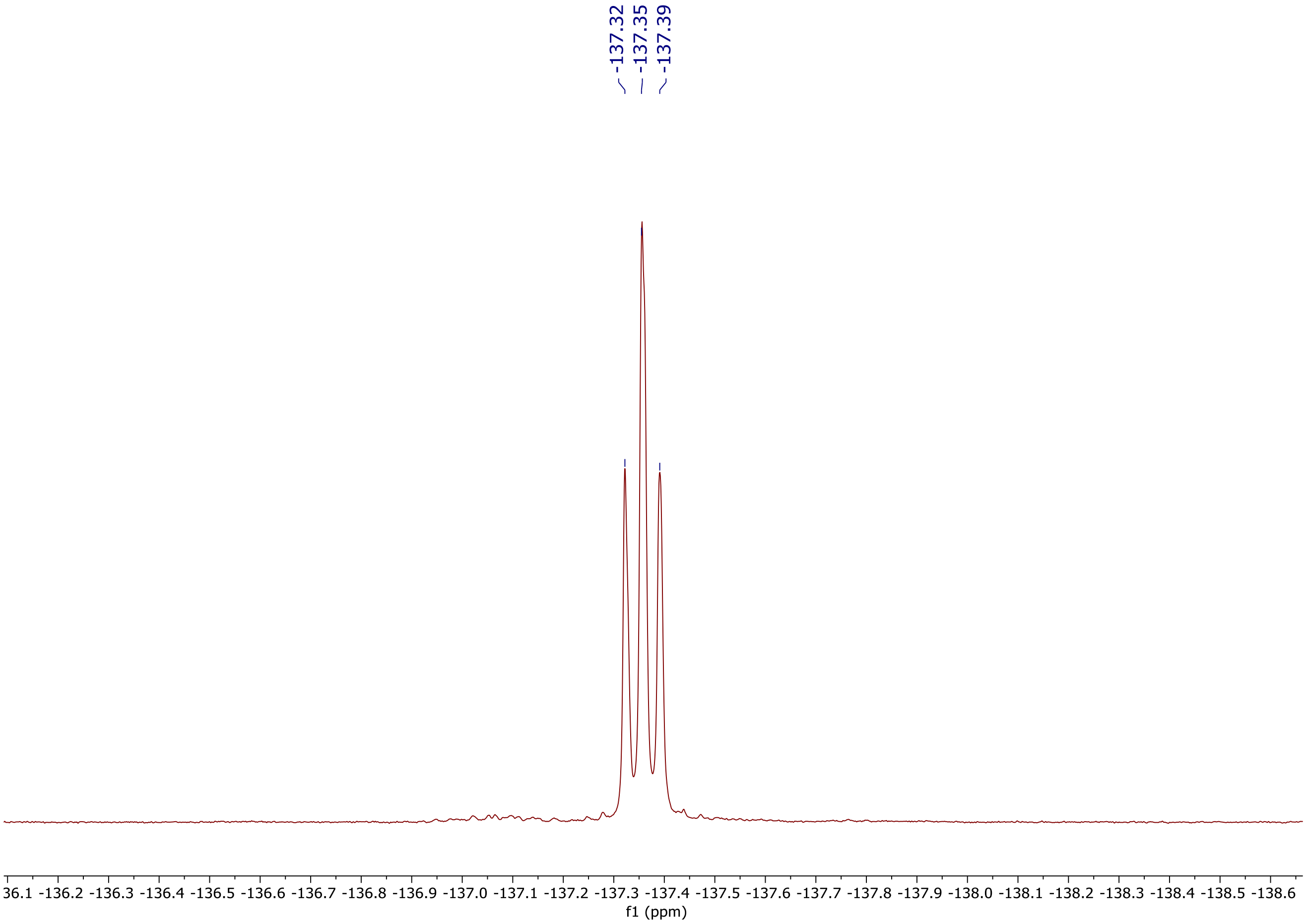

^{19}F NMR spectrum ($CDCl_3$, 282.4 MHz) of **3o**

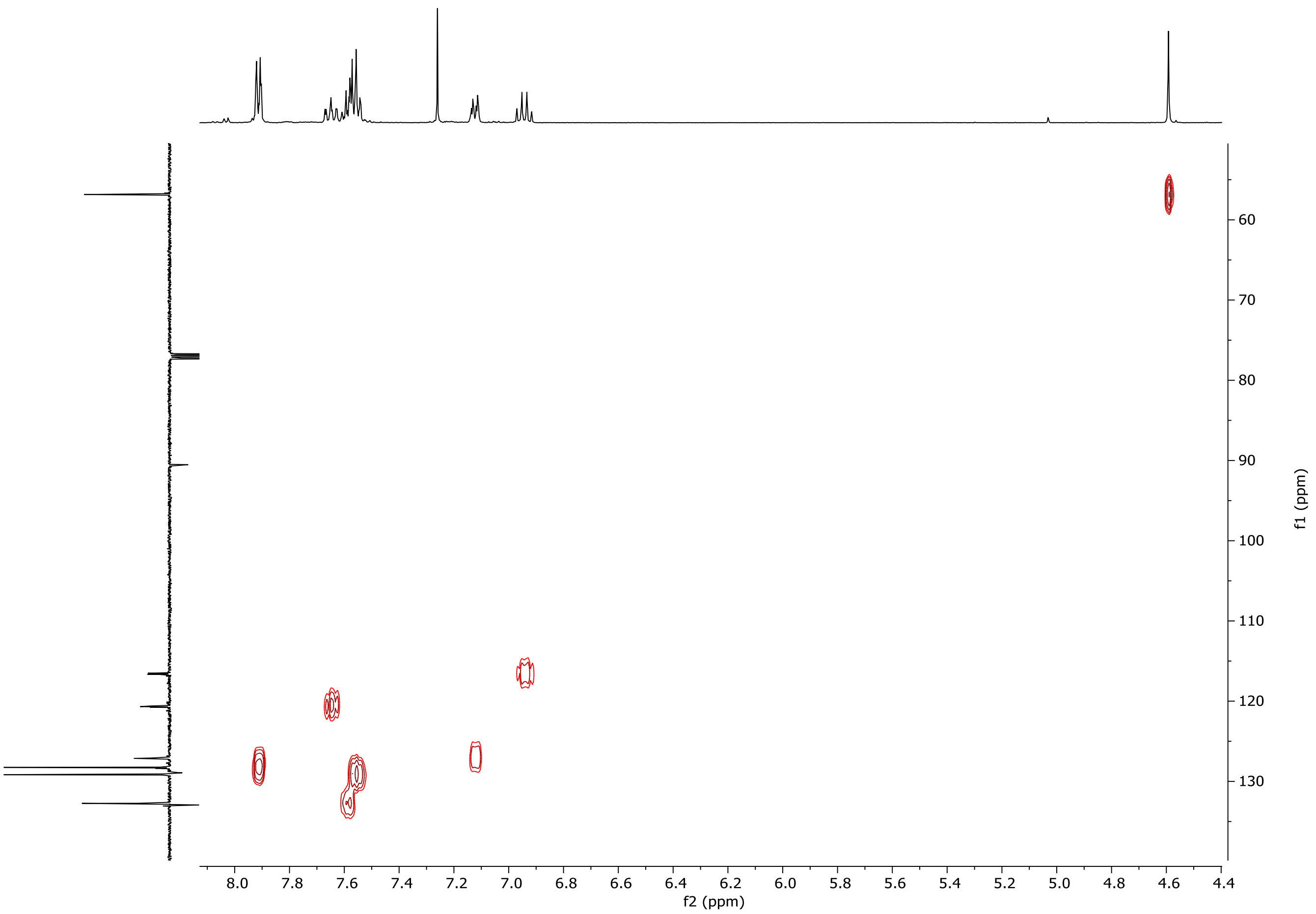

^{1}H-^{13}C HSQC correlation spectrum of **3o**

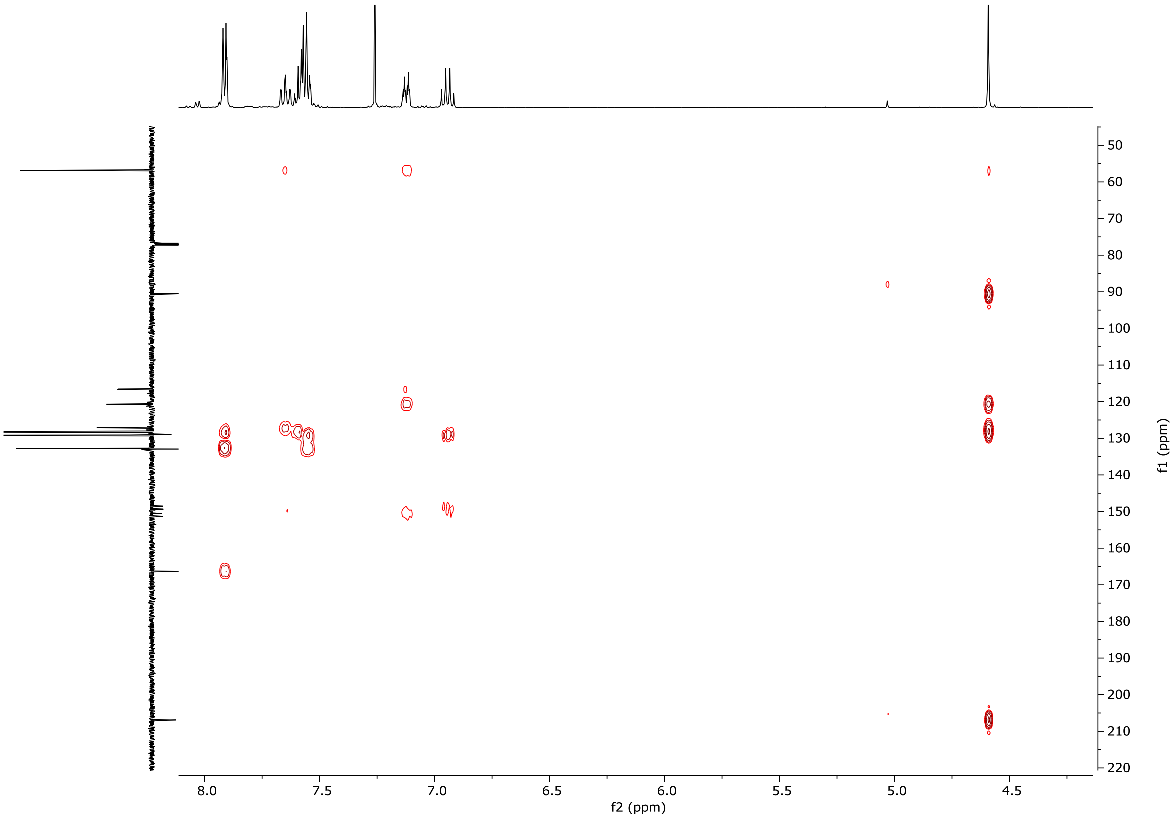

^{1}H-^{13}C HMBC correlation spectrum of **3o**

3.- NMR spectra of [2+2]-photocycloaddition products 4 (photocycloaddition in presence of BF_3)
Methyl 8-oxo-1,3,6-triphenyl-2-phenylthioamido-7-thia-5-azaspiro[3.4]-oct-5-ene-2-carboxylate 4a

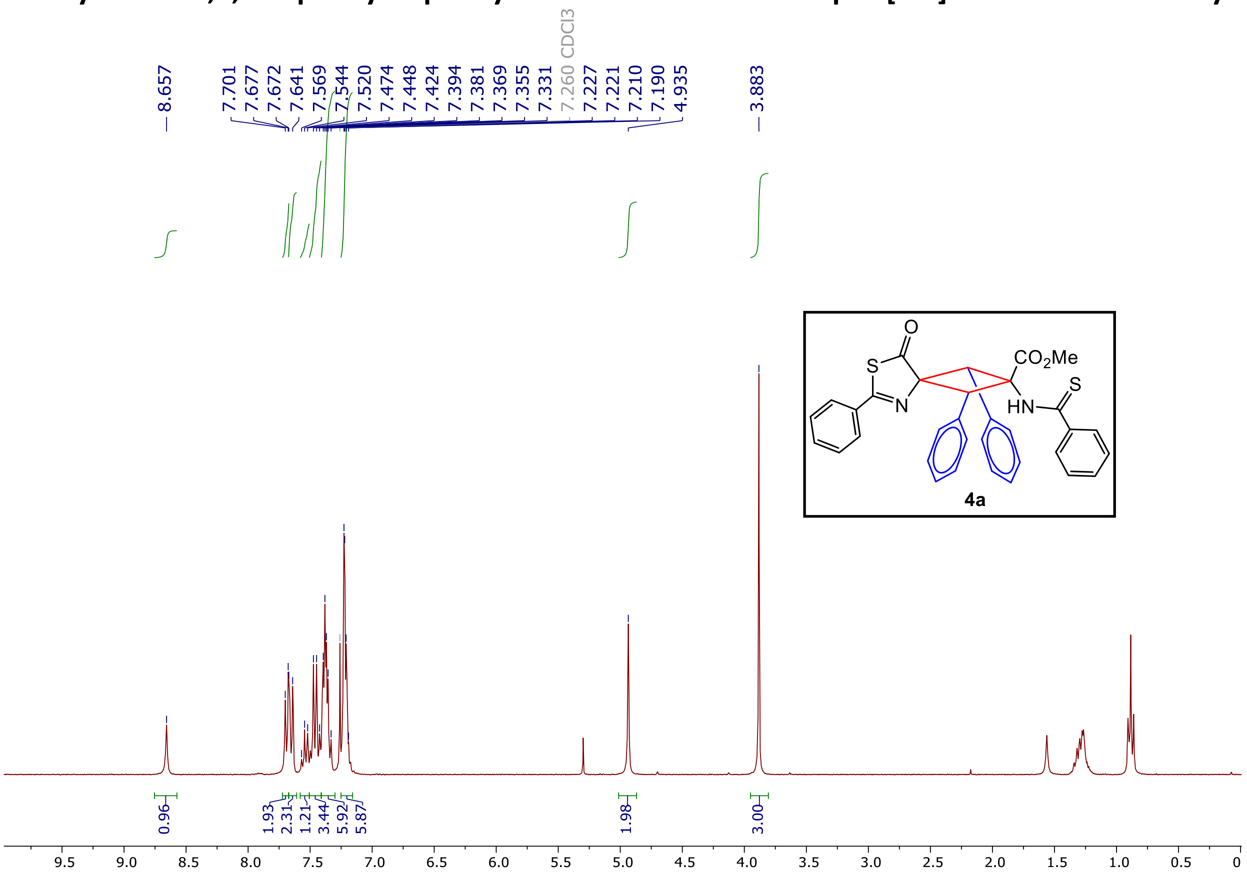

^{1}H NMR spectrum ($CDCl_3$, 300.13 MHz) of **4a**

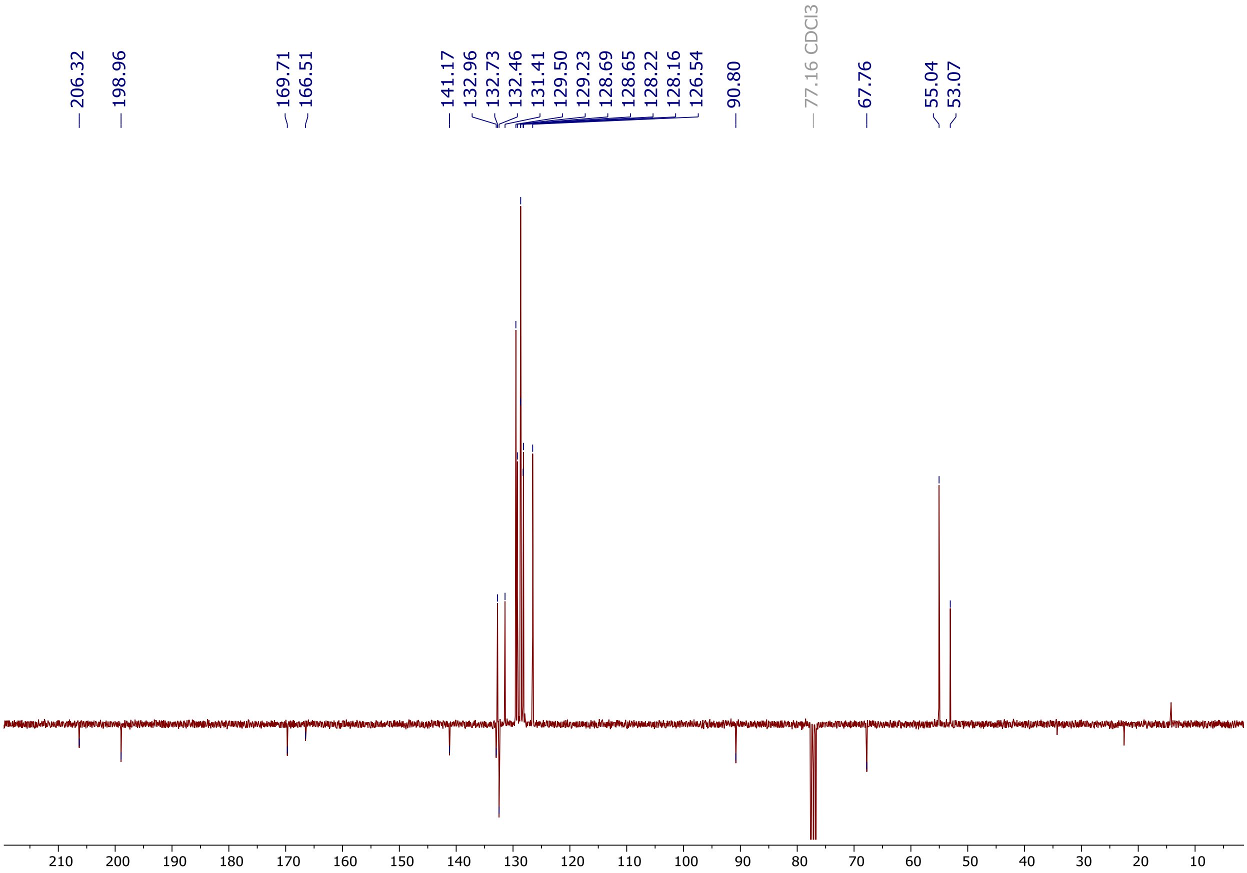

$^{13}C\{^{1}H\}$ NMR (APT) spectrum ($CDCl_3$, 75.5 MHz) of **4a**

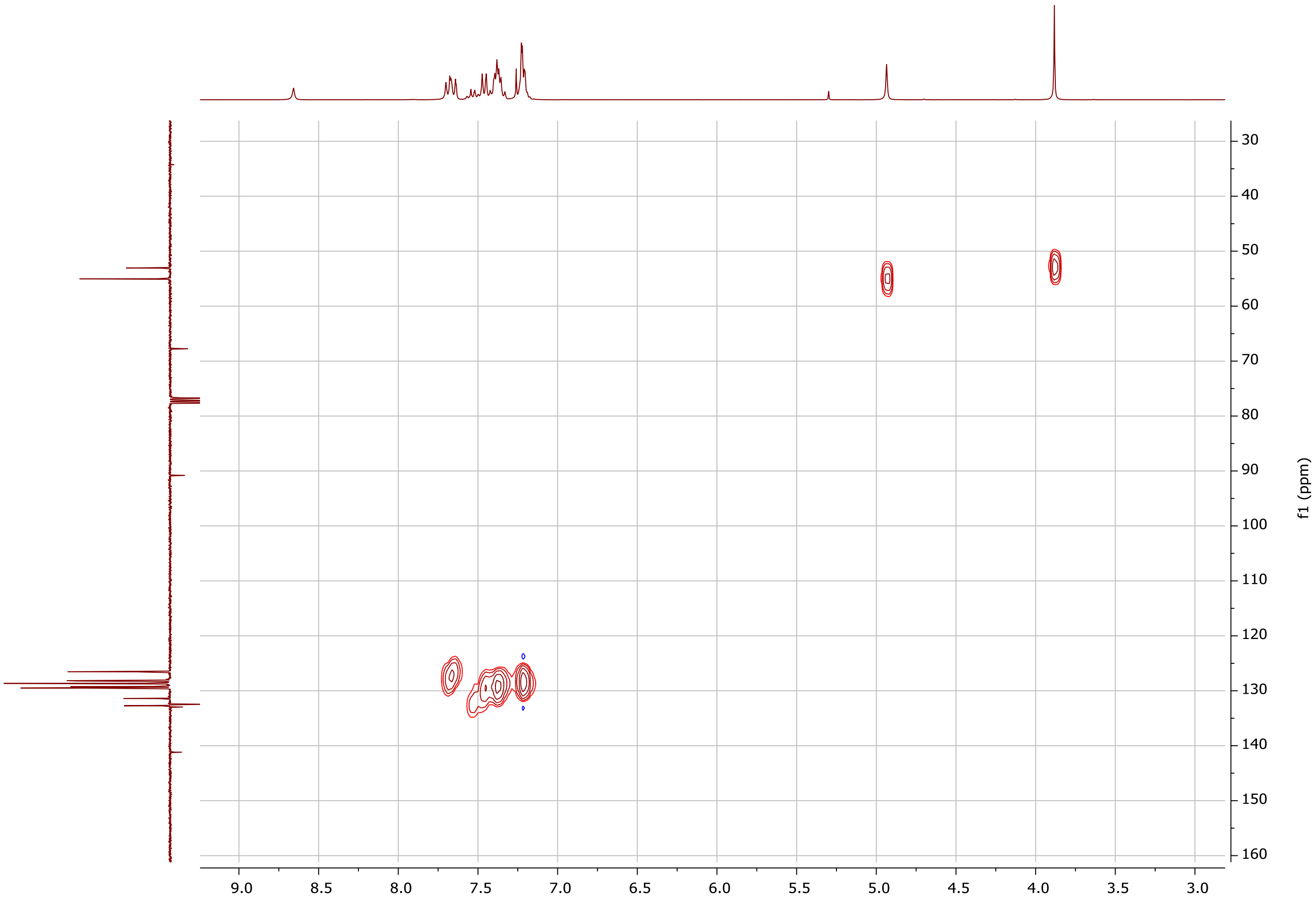

^1H-^{13}C HSQC correlation spectrum of **4a**

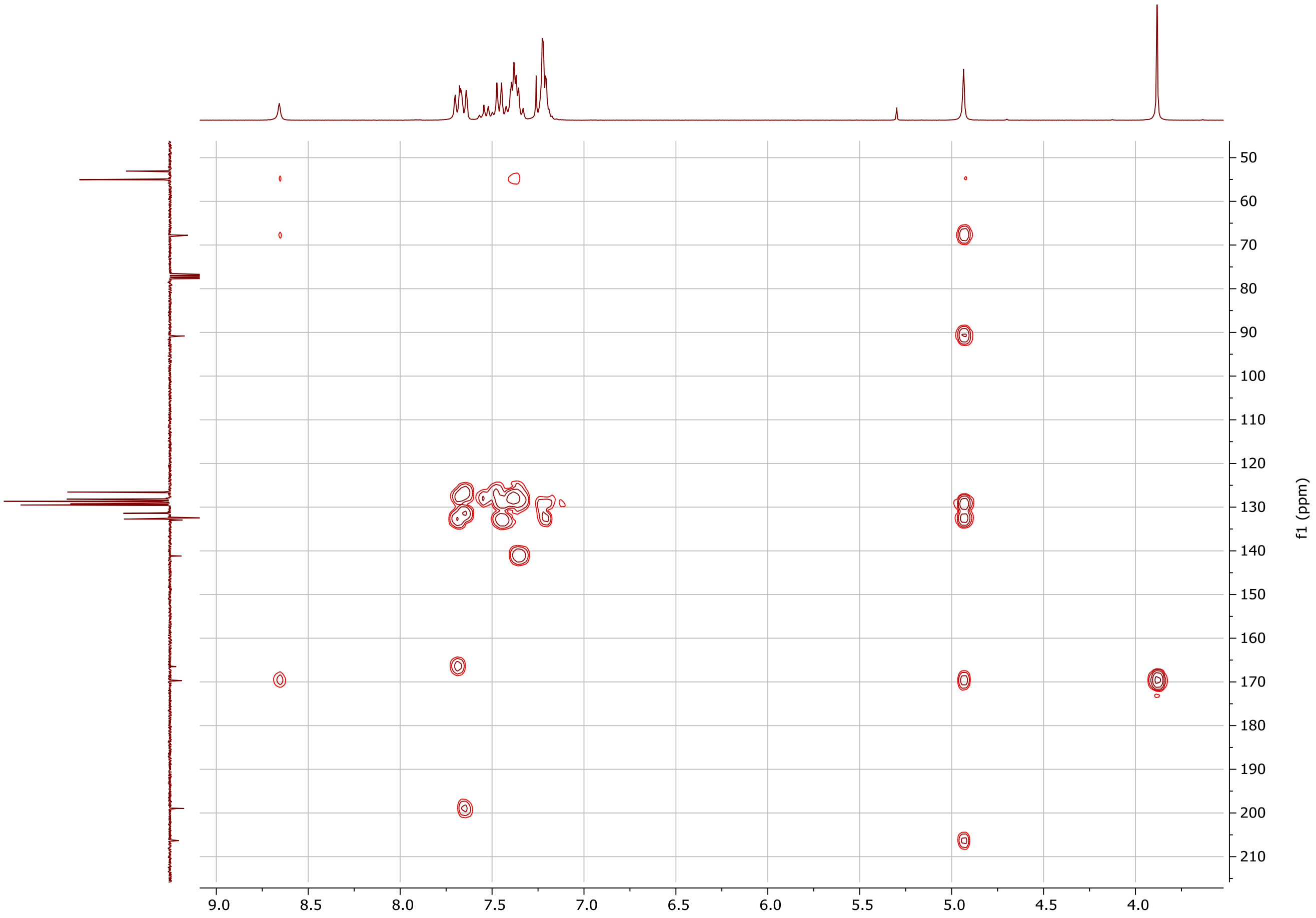

^1H-^{13}C HMBC correlation spectrum of **4a**

Methyl 8-oxo-6-phenyl-2-phenylthioamido-1,3-di-*p*-tolyl-7-thia-5-azaspiro[3.4]oct-5-ene-2-carboxylate 4b

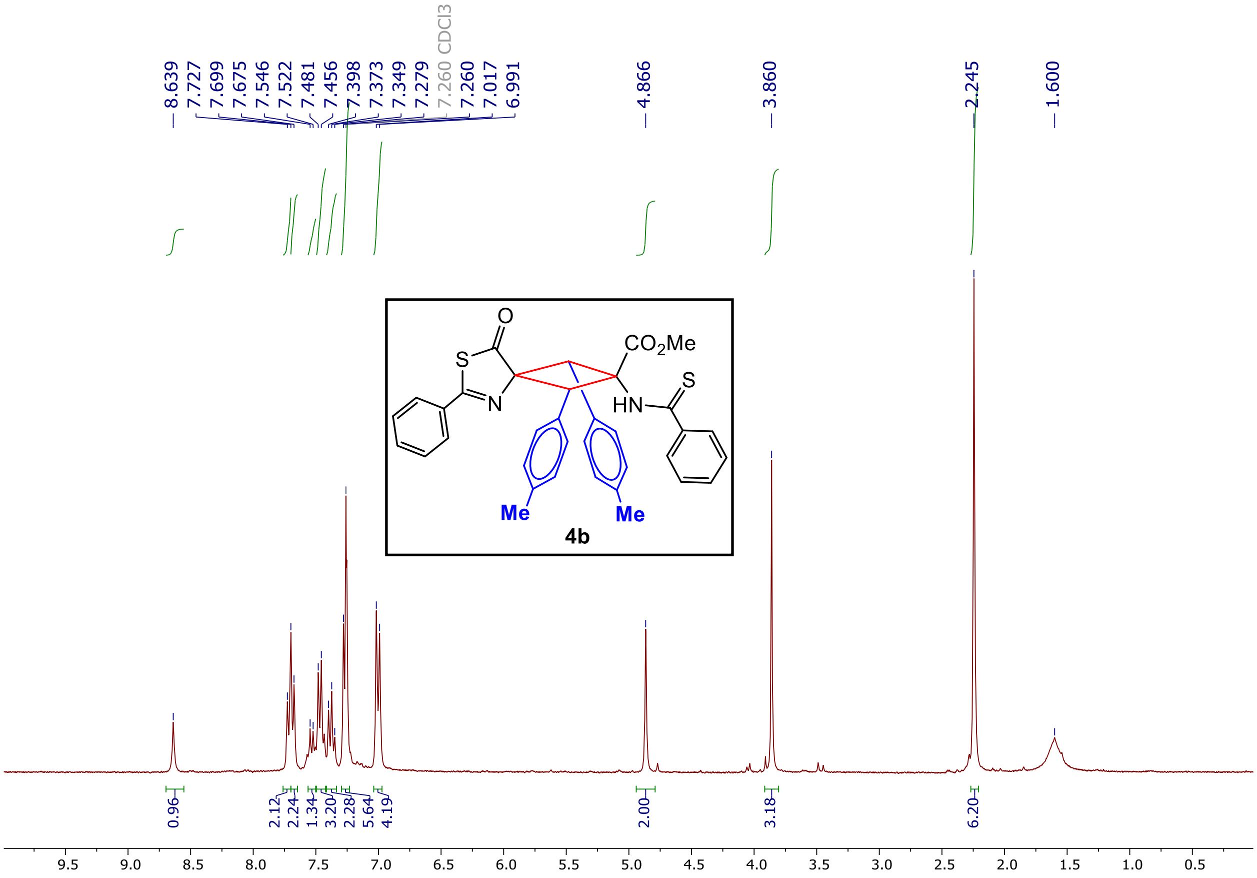

^{1}H NMR spectrum ($CDCl_3$, 300.13 MHz) of **4b**

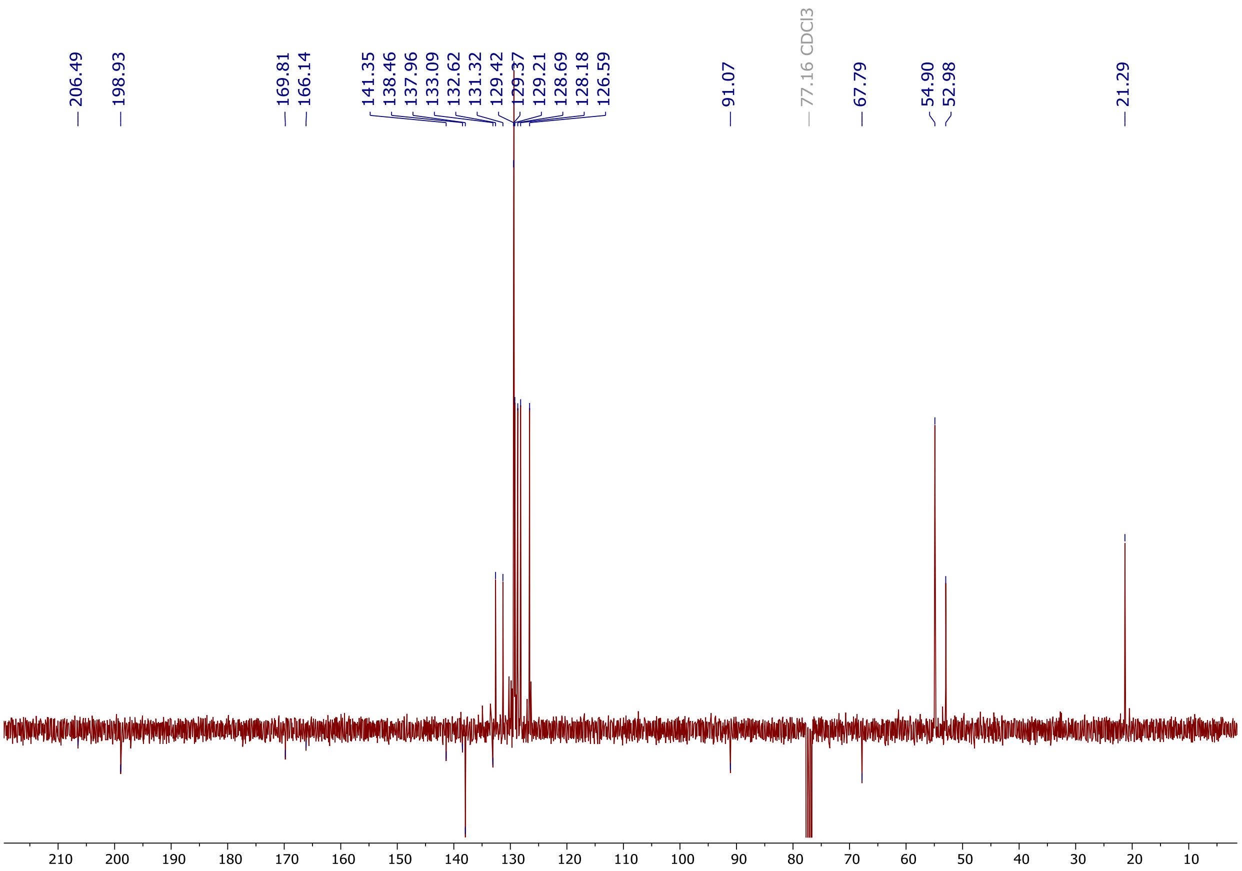

^{13}C{^{1}H} NMR (APT) spectrum ($CDCl_3$, 75.5 MHz) of **4b**

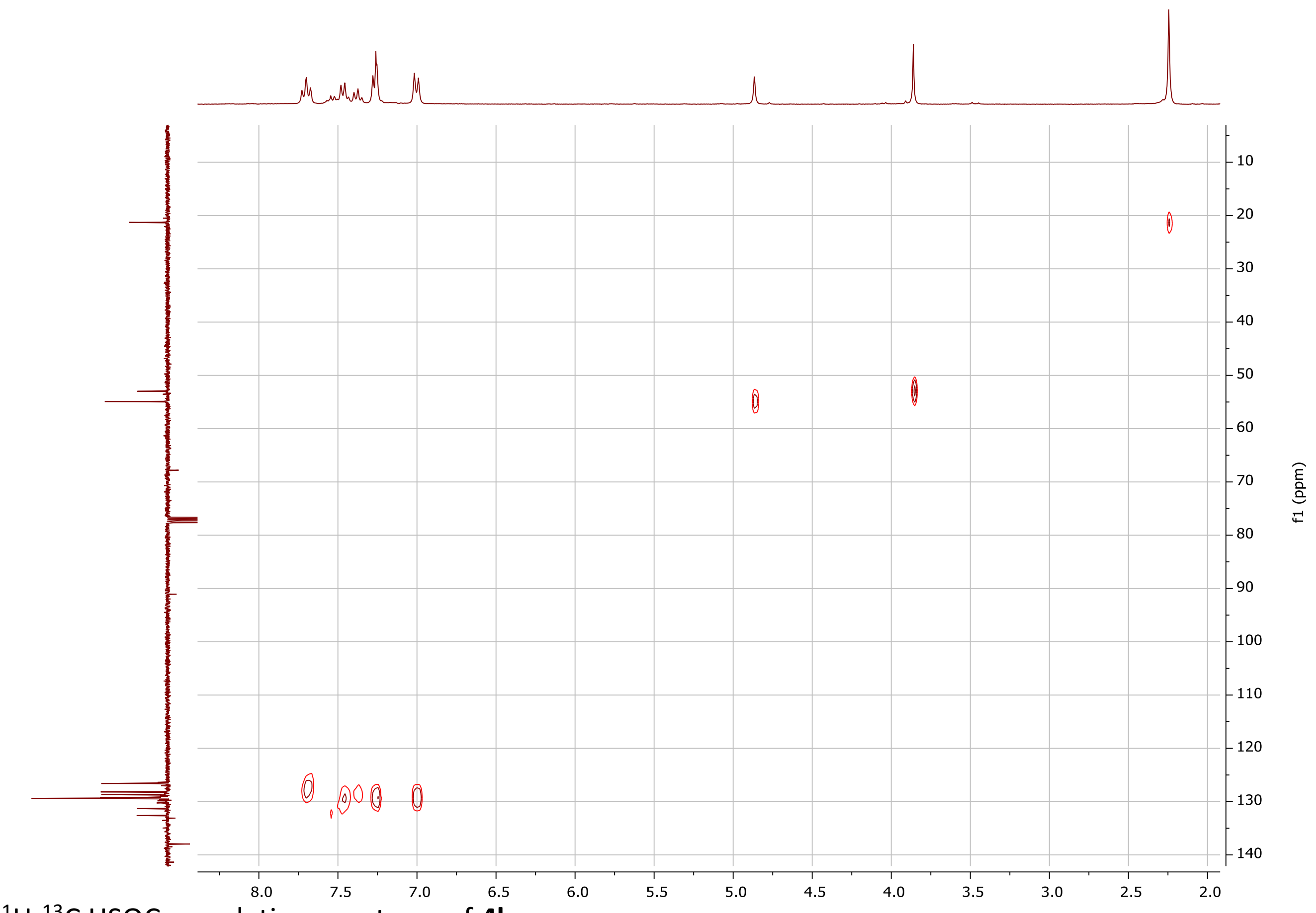

^{1}H-^{13}C HSQC correlation spectrum of **4b**

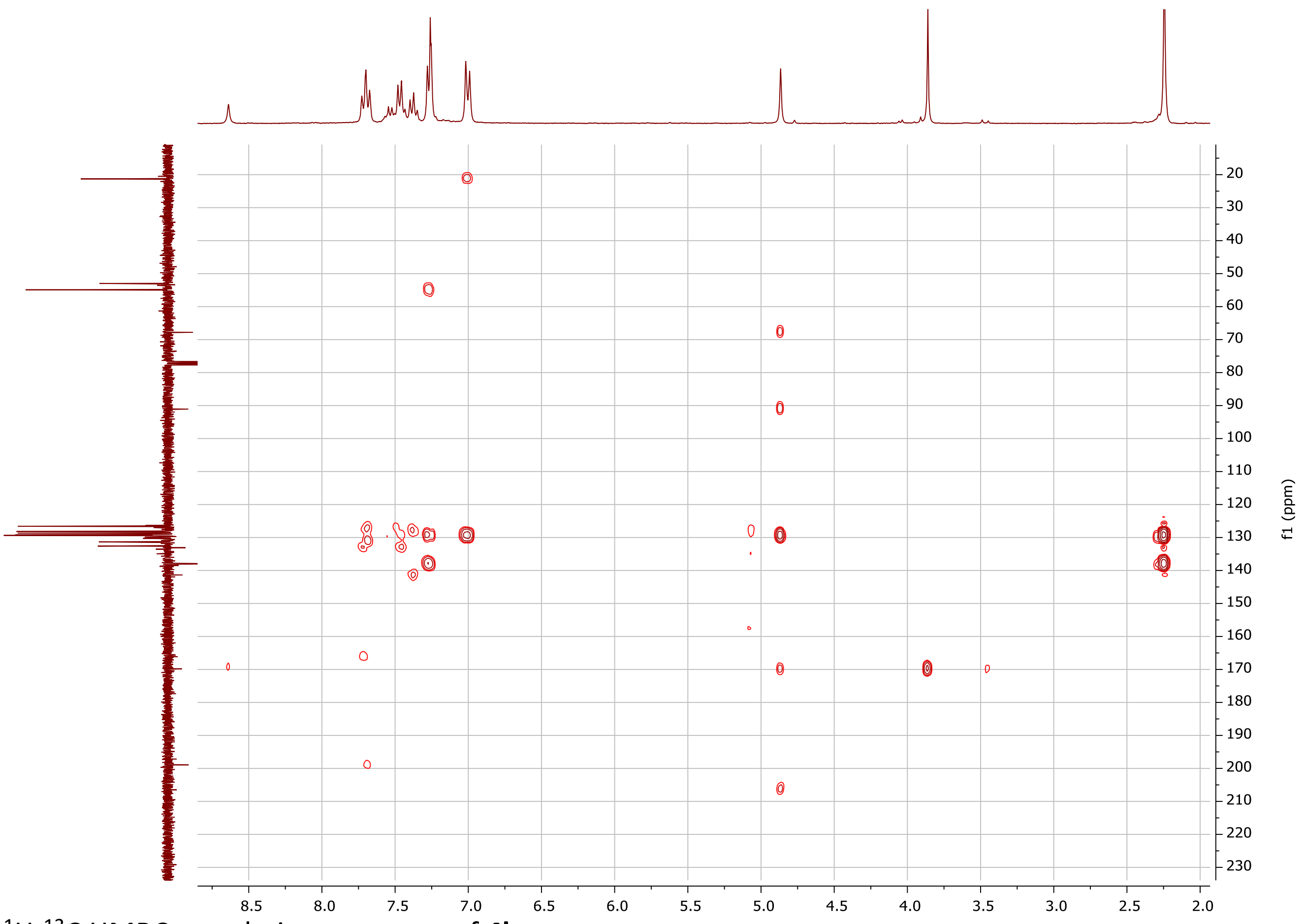

^{1}H-^{13}C HMBC correlation spectrum of **4b**

Methyl 1,3-bis(4-fluorophenyl)-8-oxo-6-phenyl-2-phenylthioamido-7-thia-5-azaspiro[3.4]oct-5-ene-2-carboxylate 4d

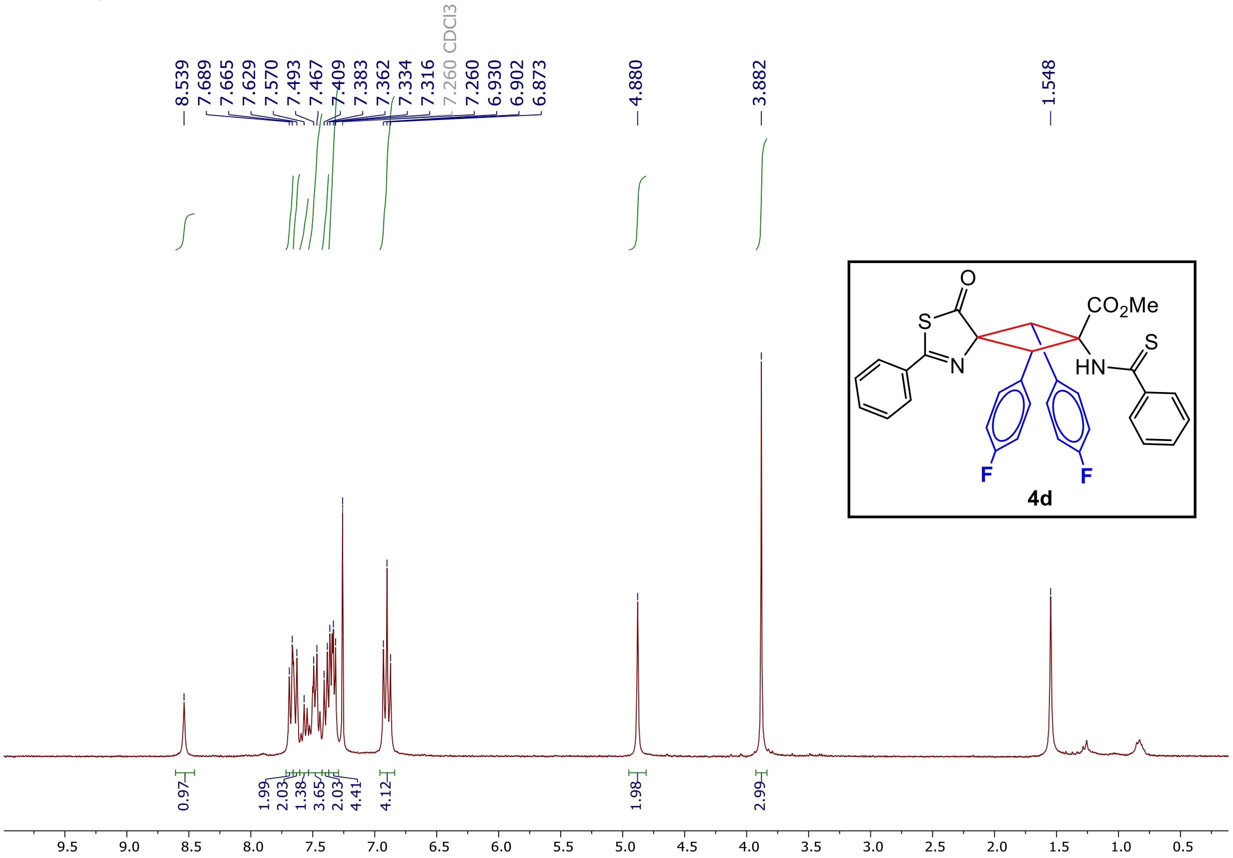

^{1}H spectrum ($CDCl_3$, 300.13 MHz) of **4d**

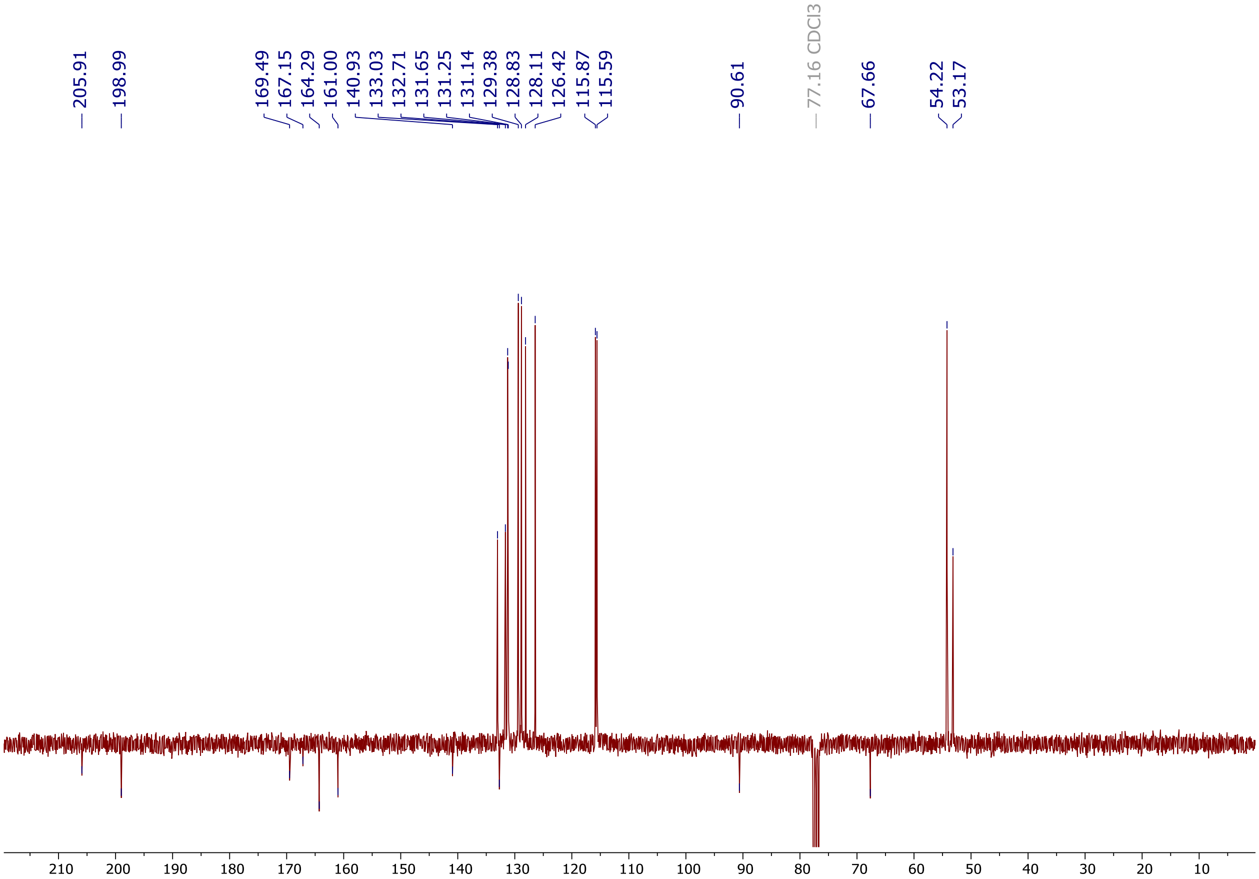

$^{13}C\{^{1}H\}$ NMR (APT) spectrum ($CDCl_3$, 75.5 MHz) de **4d**

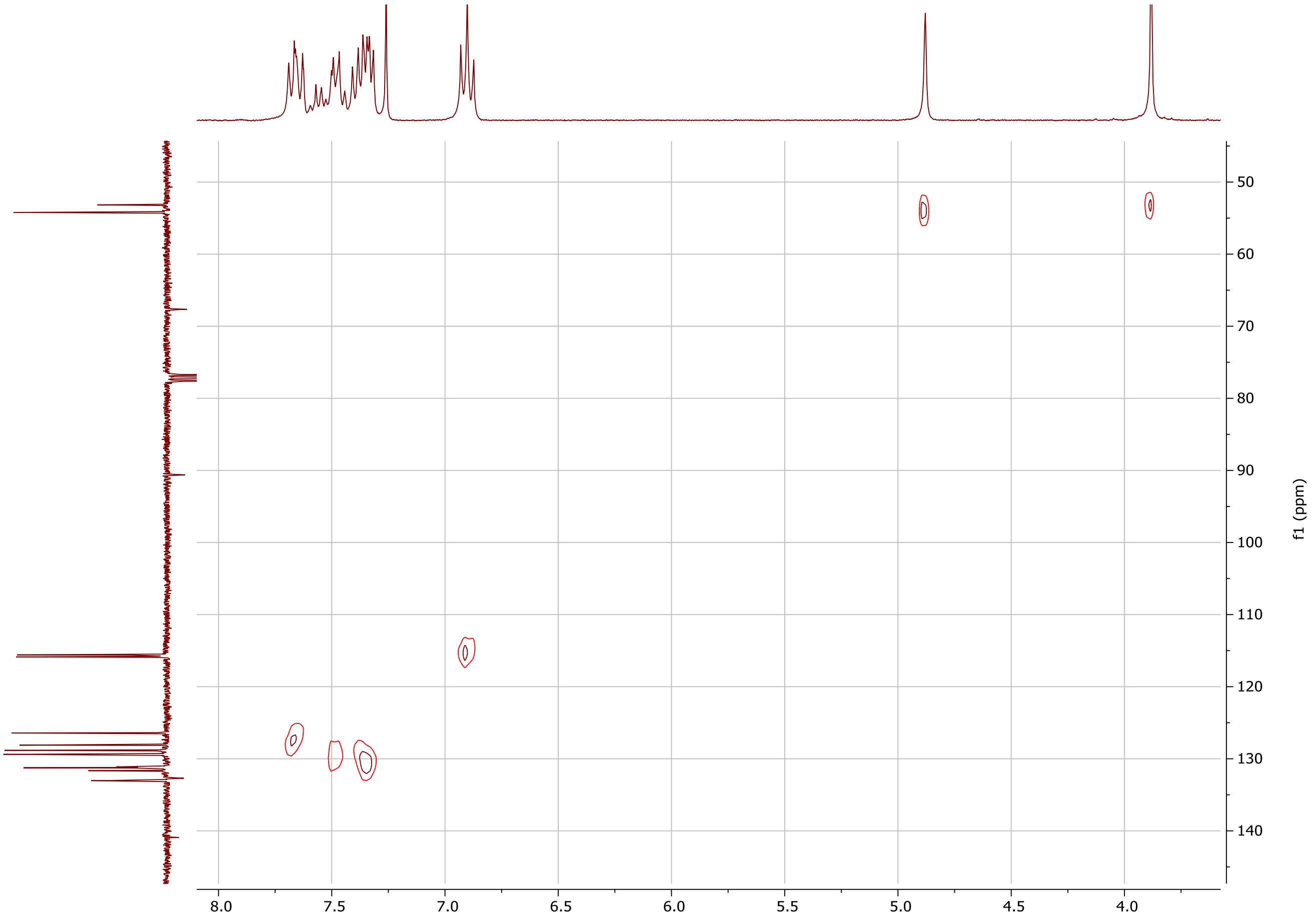

^{1}H-^{13}C HSQC correlation spectrum of **4d**

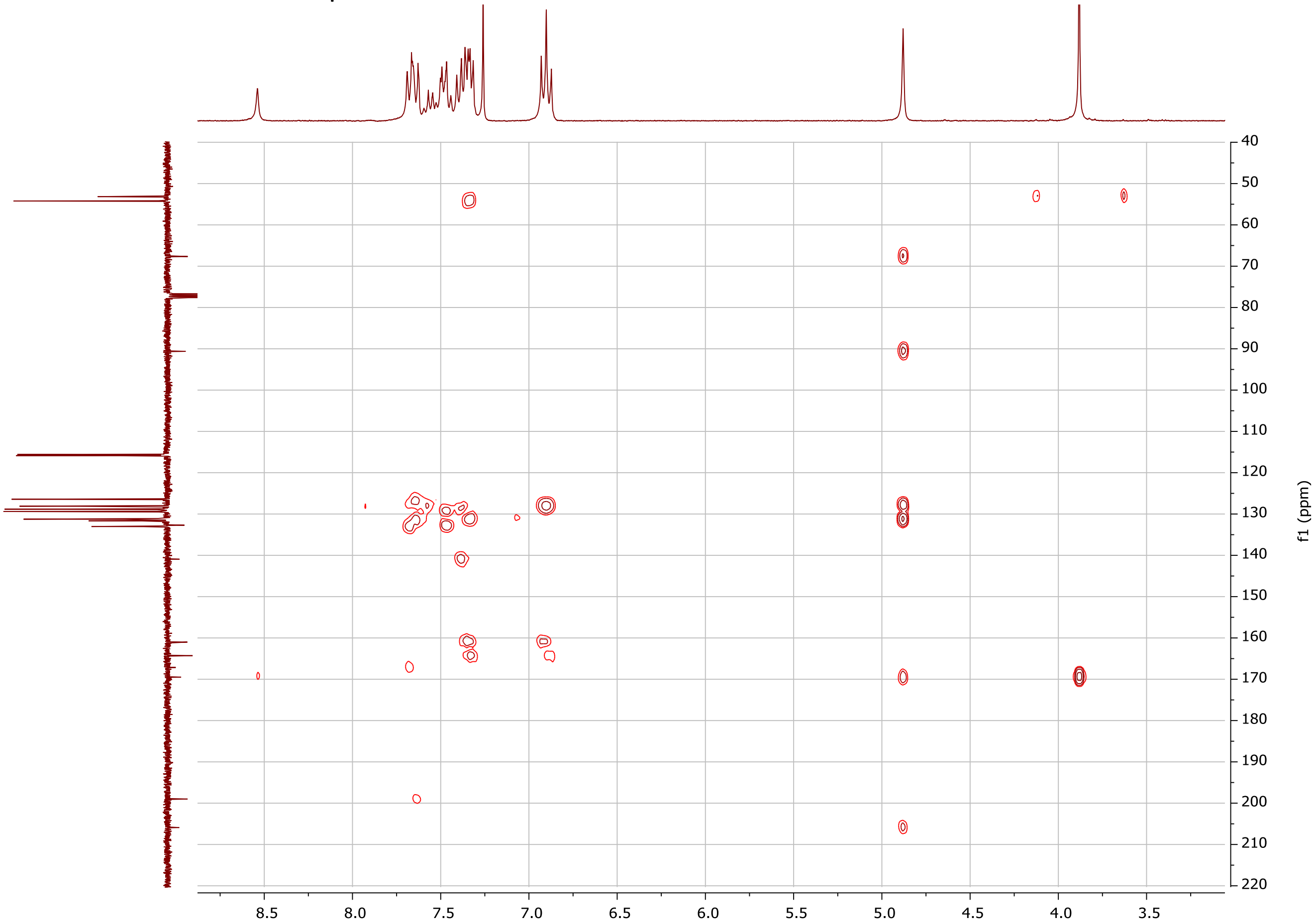

^{1}H-^{13}C HMBC correlation spectrum of **4d**

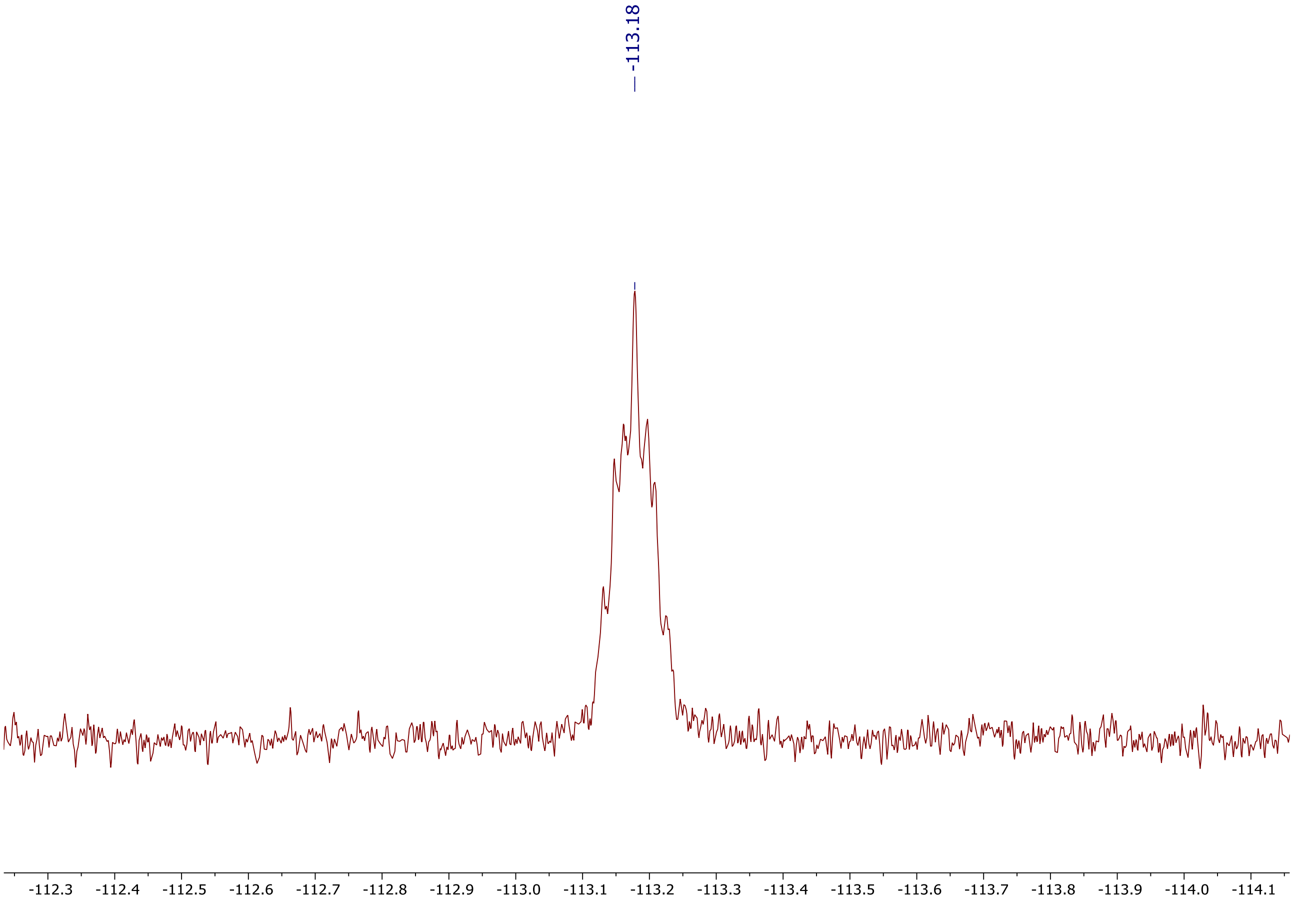

^{19}F NMR spectrum ($CDCl_3$, 282.40 MHz) of **4d**

Methyl 1,3-bis(4-chlorophenyl)-8-oxo-6-phenyl-2-phenylthioamido-7-thia-5-azaspiro[3.4]oct-5-ene-2-carboxylate 4e

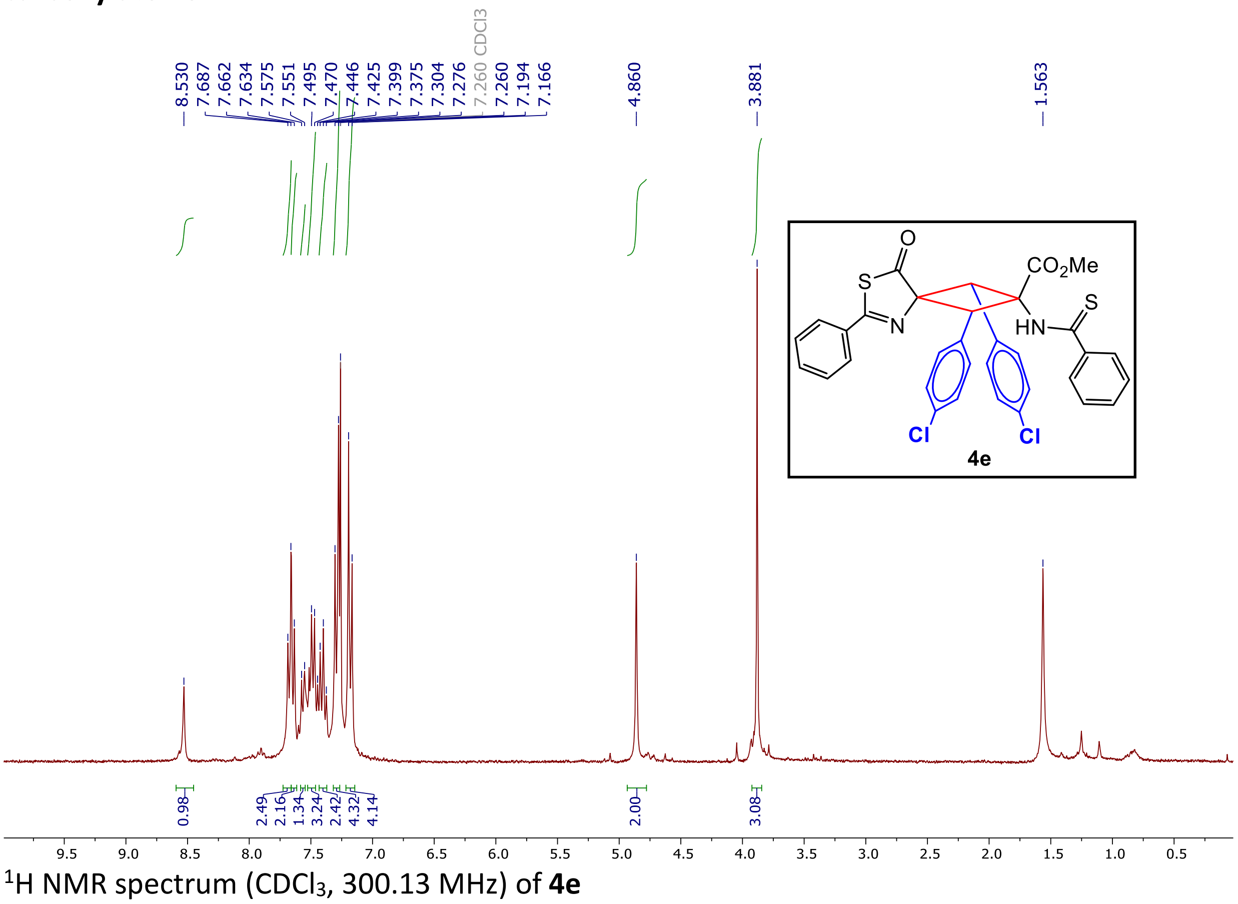

^{1}H NMR spectrum ($CDCl_3$, 300.13 MHz) of **4e**

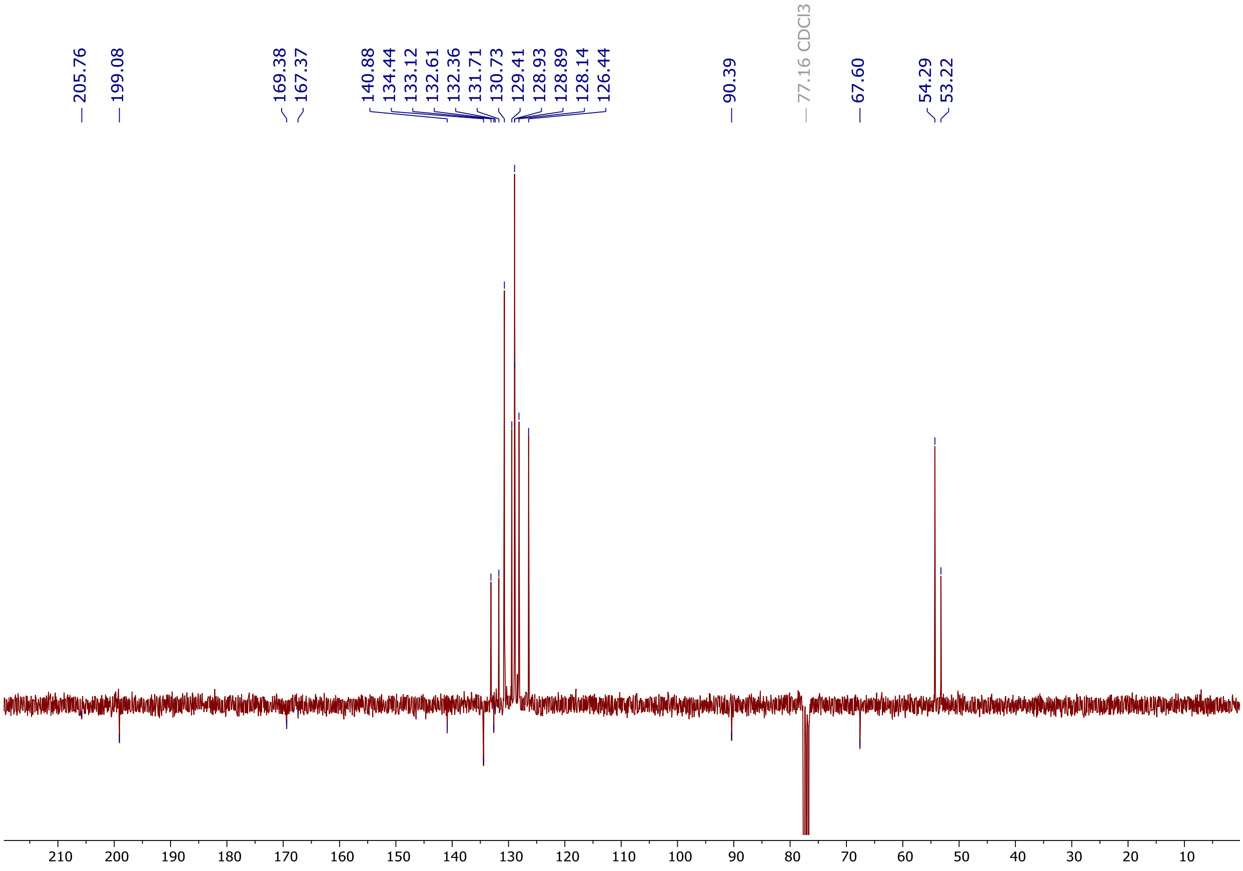

$^{13}C\{^1H\}$ NMR (APT) spectrum ($CDCl_3$, 75.5 MHz) of **4e**

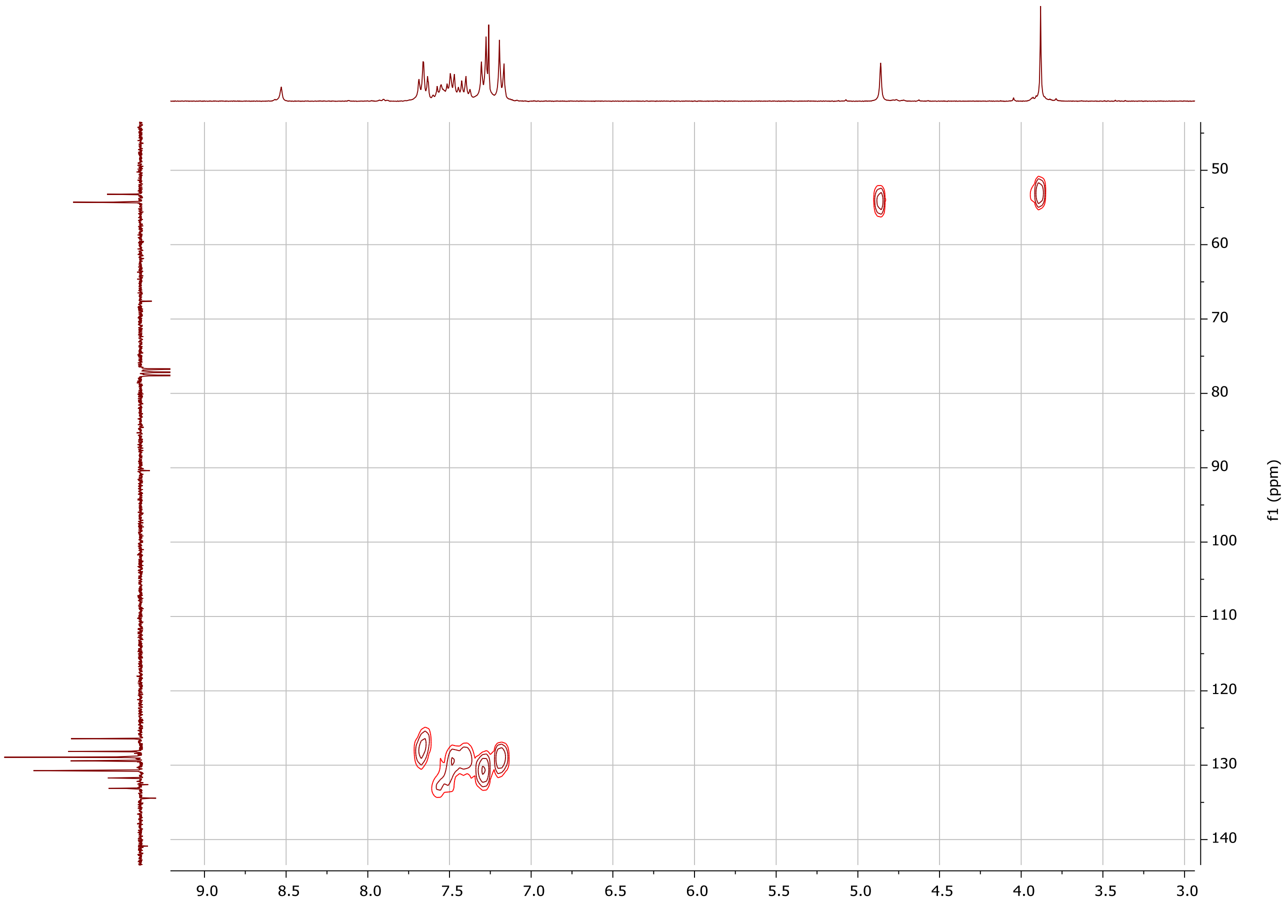

^1H-^{13}C HSQC correlation spectrum of **4e**

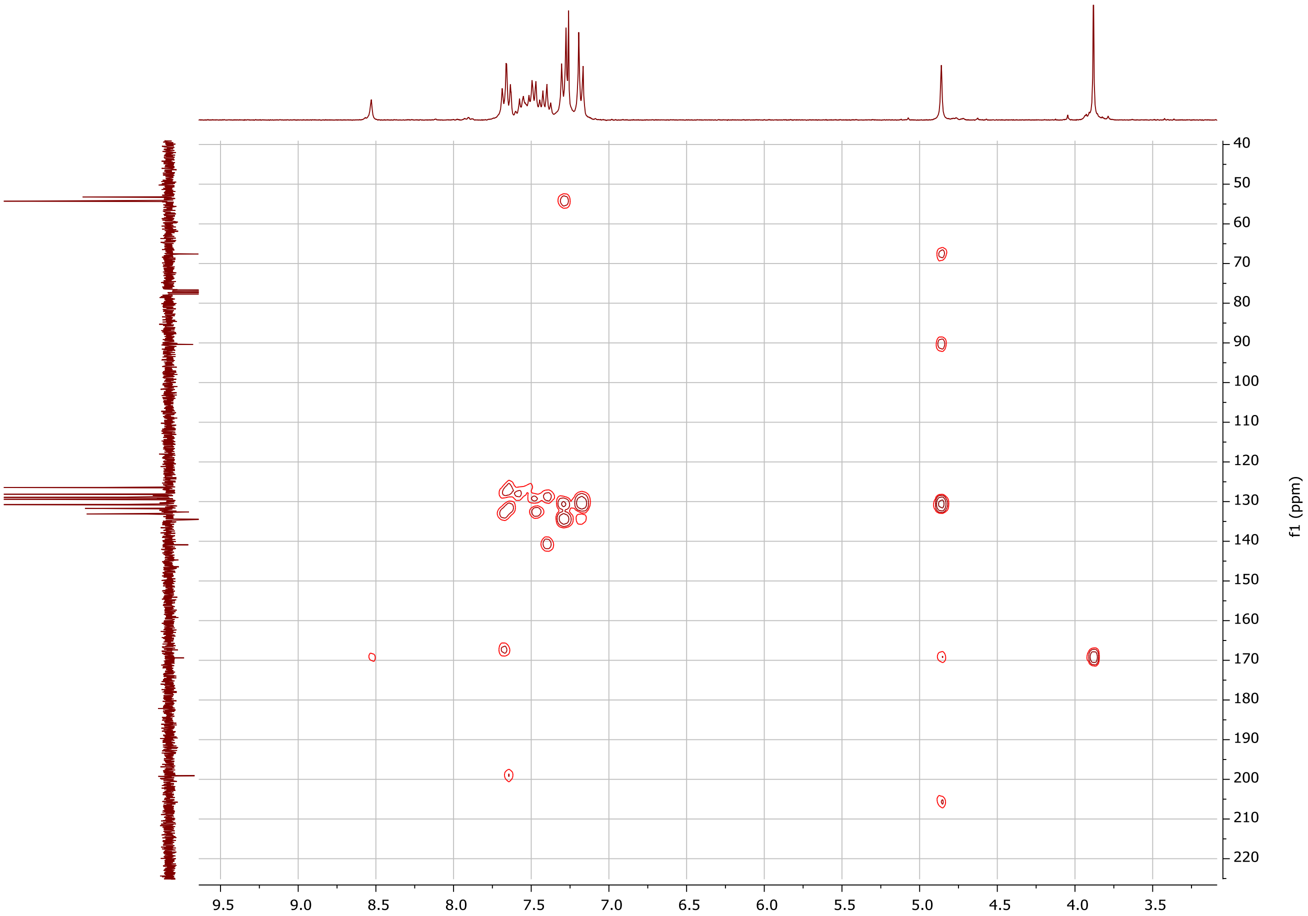

^{1}H-^{13}C HMBC correlation spectrum of **4e**

4.- NMR spectra of dihydrothiazole derivatives 5 and 6, and thiazoles 7 (ring opening with base in absence of BF_3)

Methyl 5-(4-methoxyphenyl)-2-phenyl-4,5-dihydrothiazole-4-carboxylate 5c

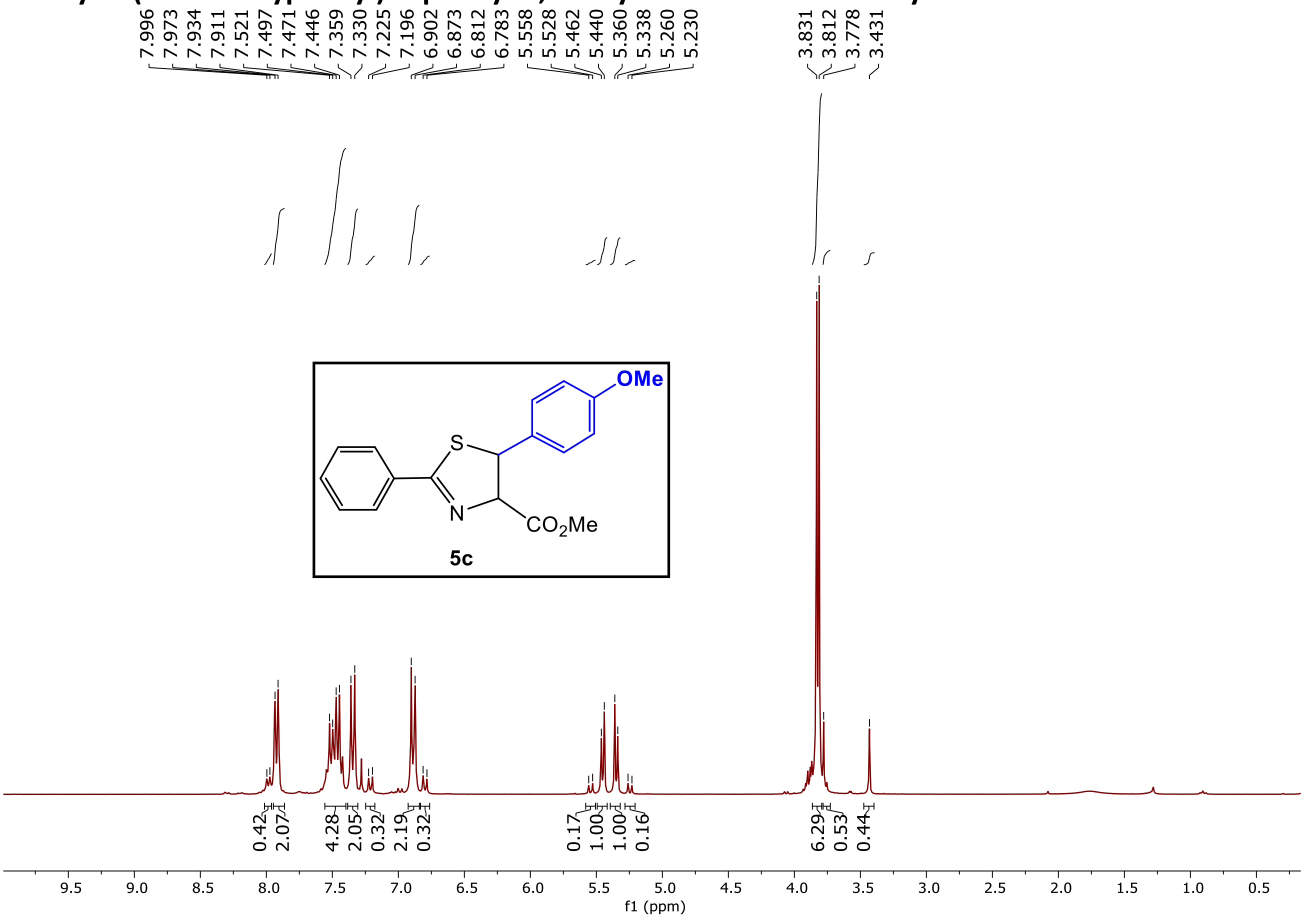

^{1}H NMR spectrum ($CDCl_3$, 300.13 MHz) of **5c**

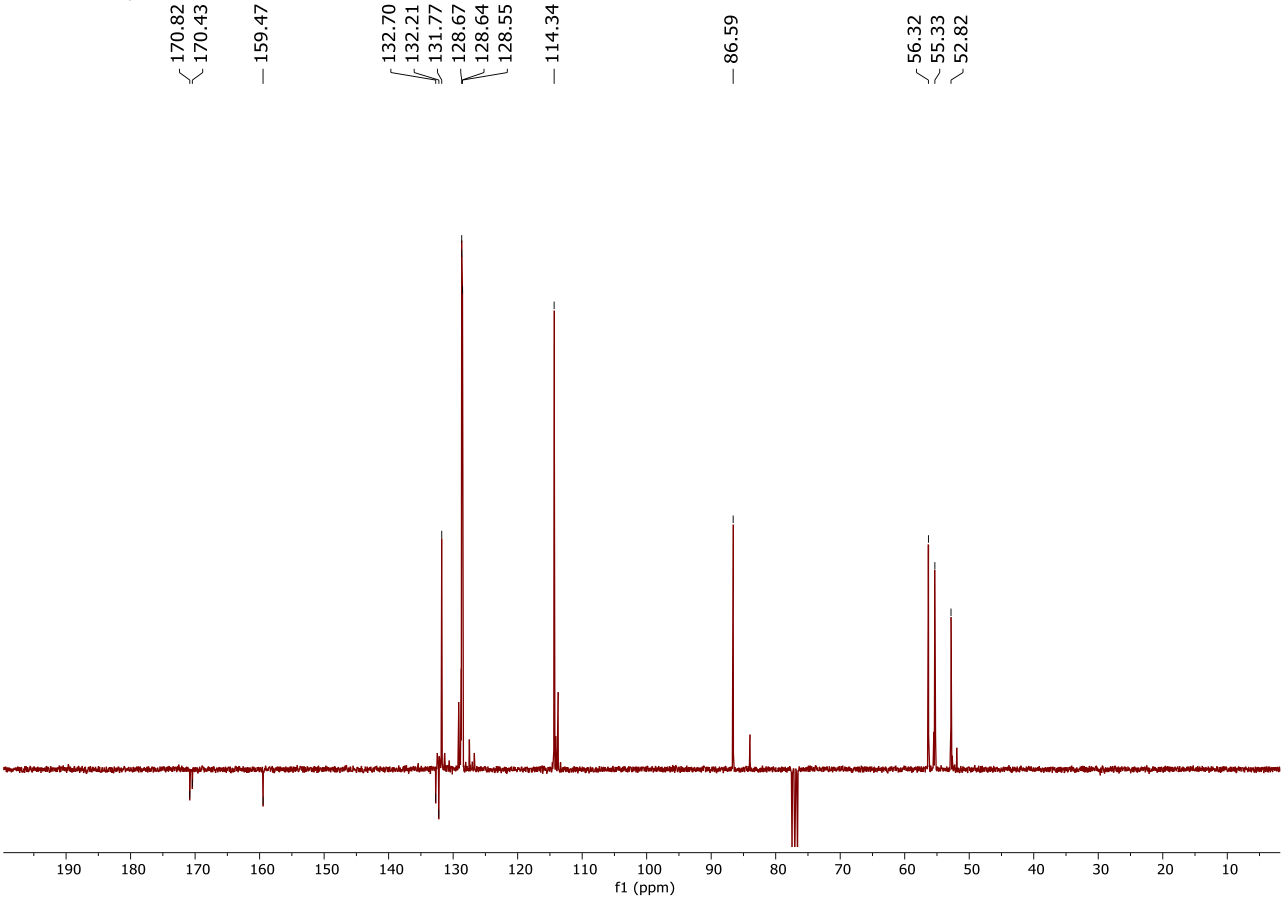

^{13}C{^{1}H} (APT) NMR spectrum ($CDCl_3$, 75.47 MHz) of **5c**

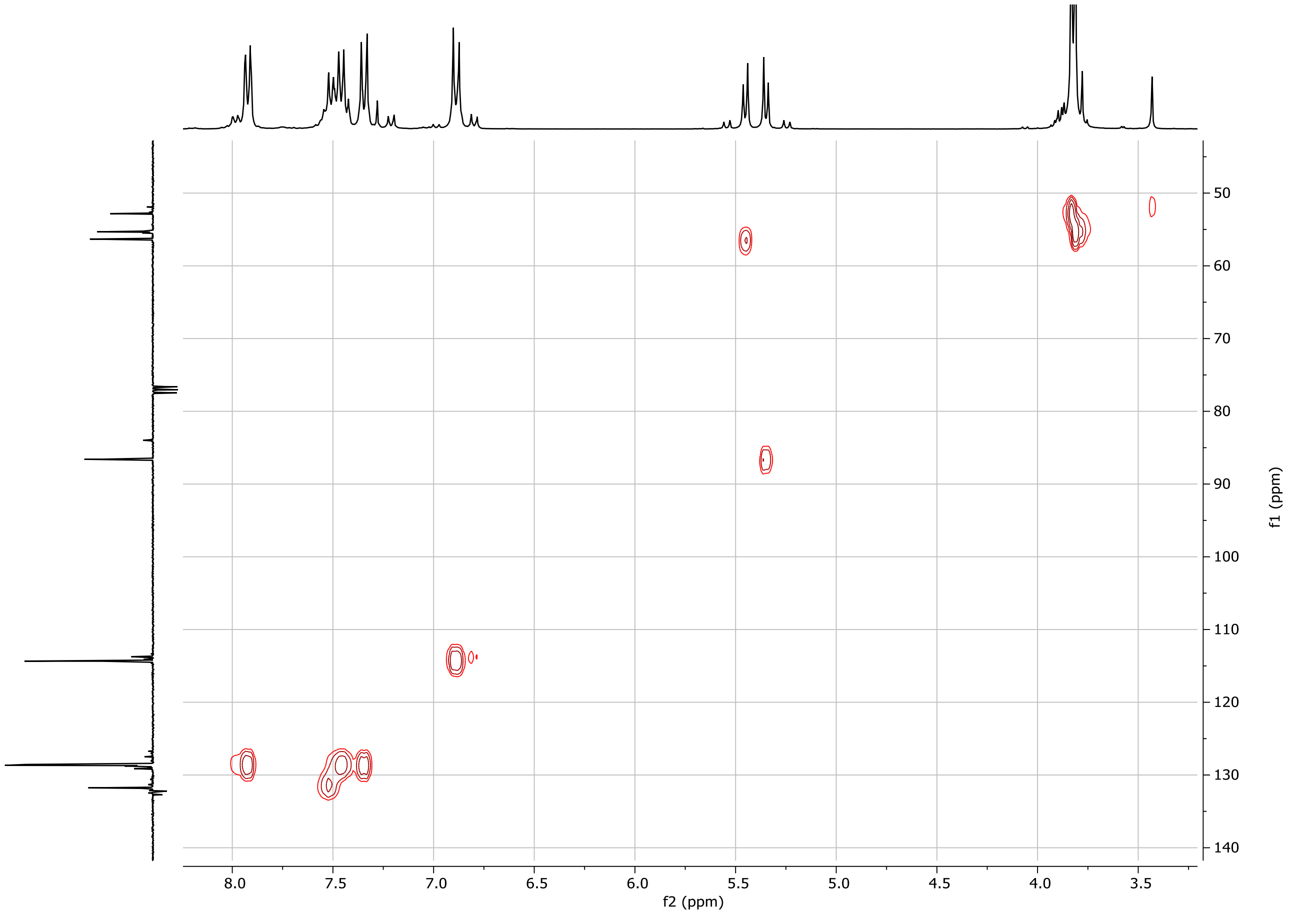

^{1}H-^{13}C HSQC correlation spectrum of **5c**

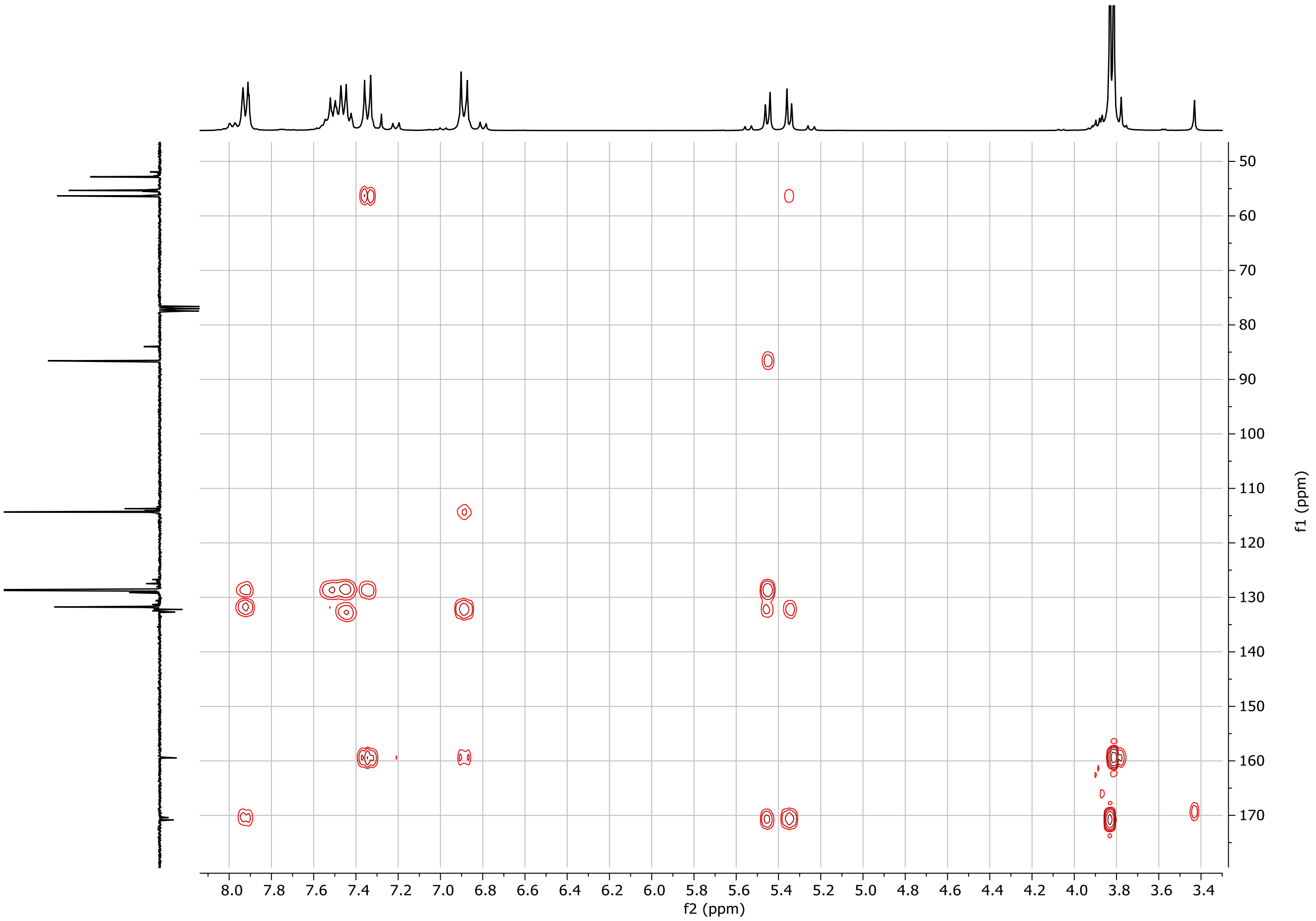

^{1}H-^{13}C HMBC correlation spectrum of **5c**

Methyl 5-(4-fluorophenyl)-2-phenyl-4,5-dihydrothiazole-4-carboxylate 5d

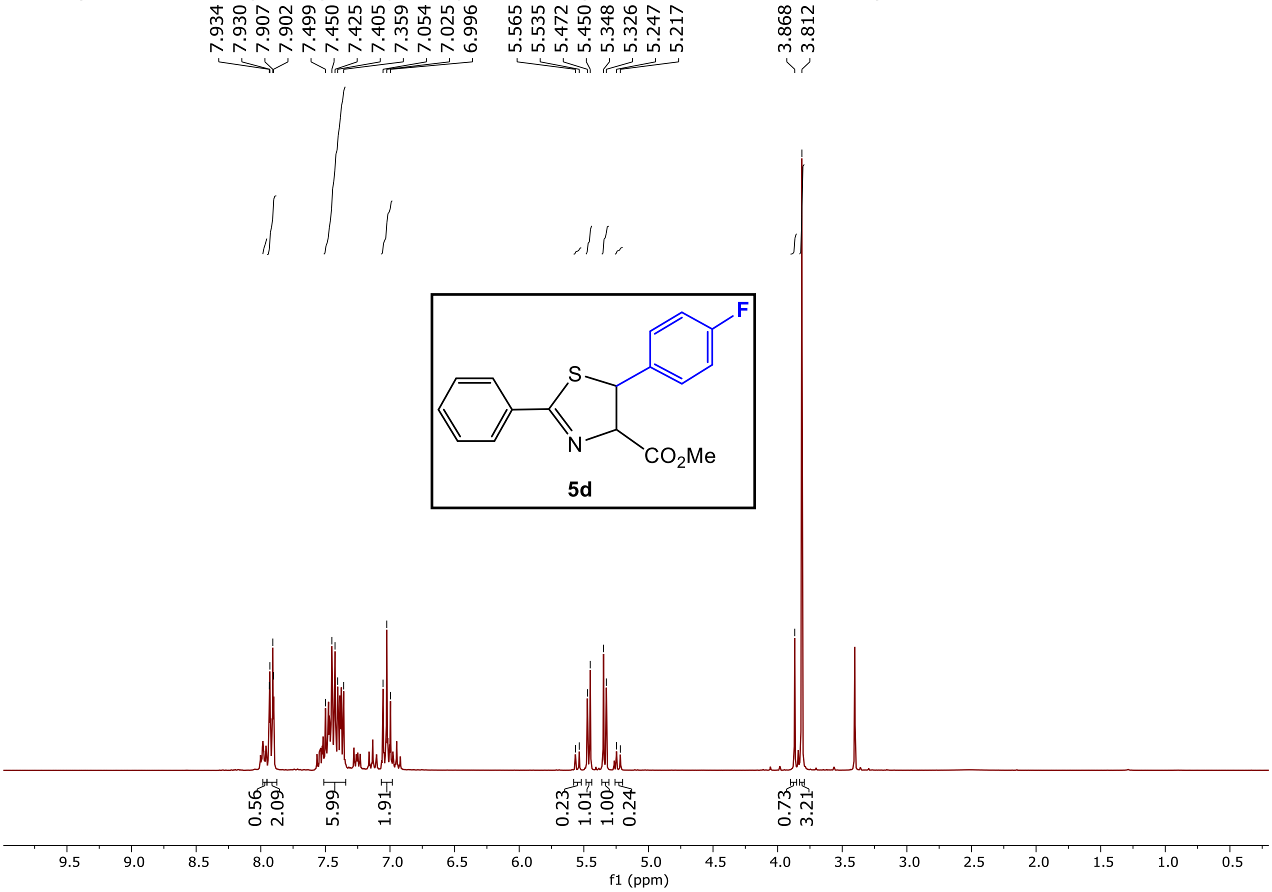

^{1}H NMR spectrum ($CDCl_3$, 300.13 MHz) of **5d**

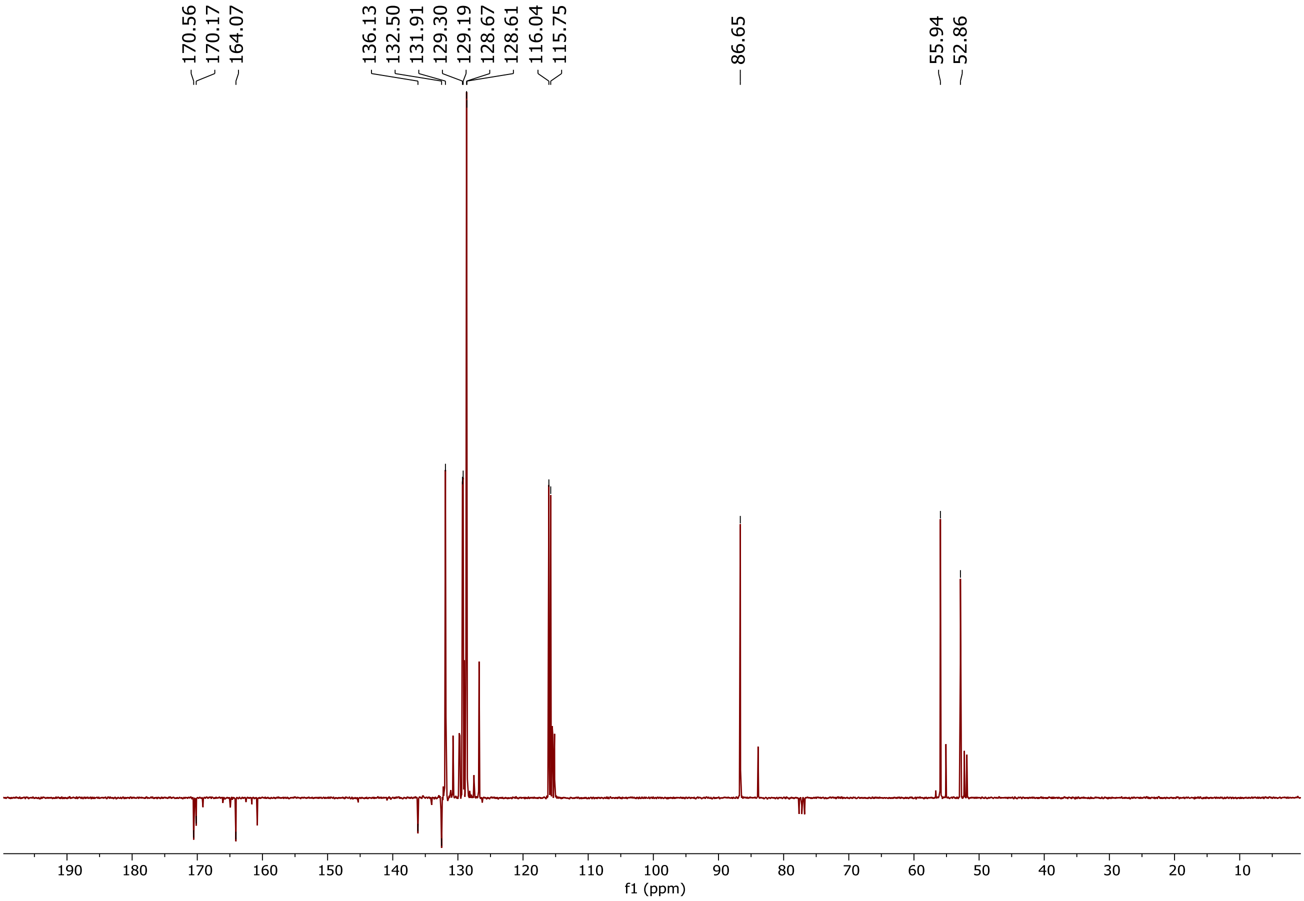

$^{13}C\{^{1}H\}$ (APT) NMR spectrum ($CDCl_3$, 75.47 MHz) of **5d**

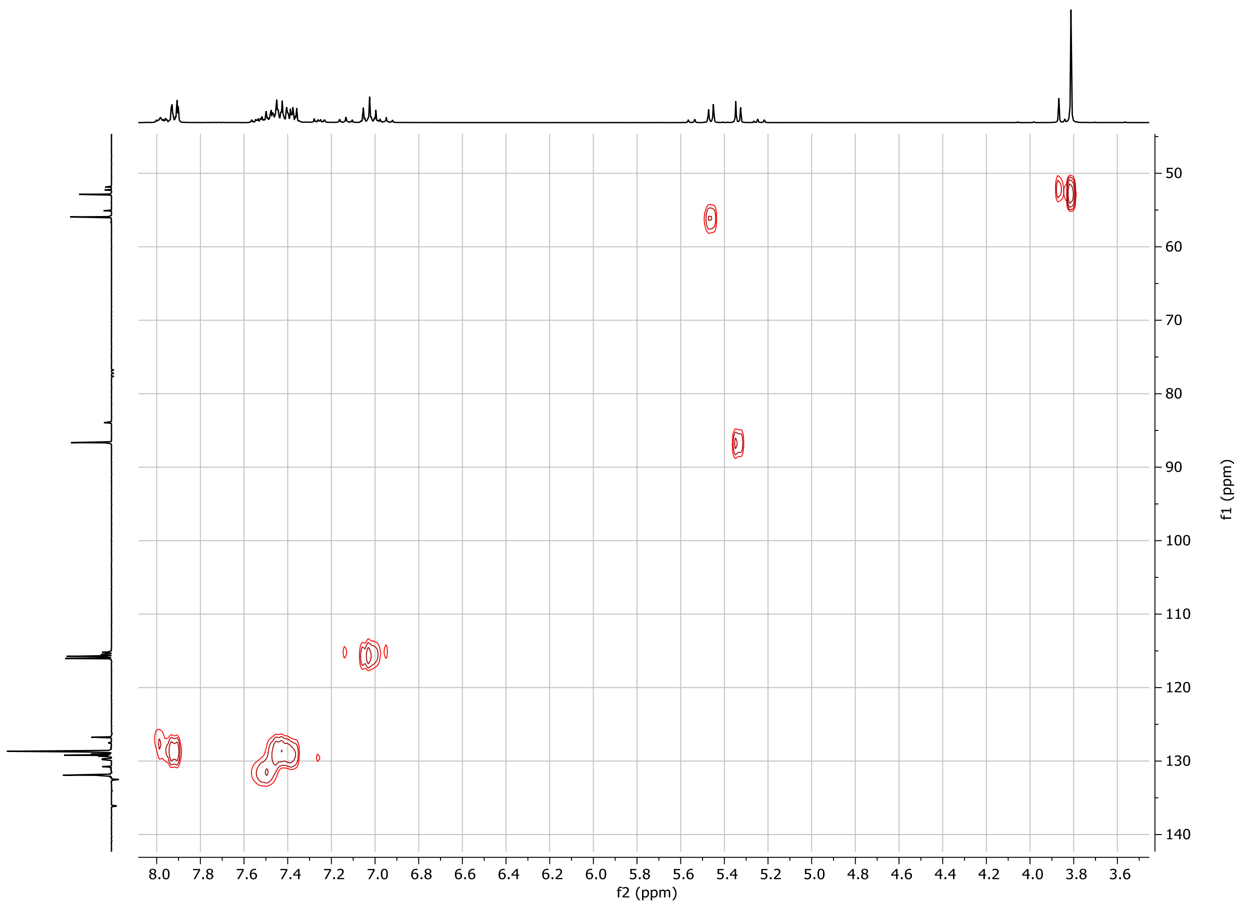

^{1}H-^{13}C HSQC correlation spectrum of **5d**

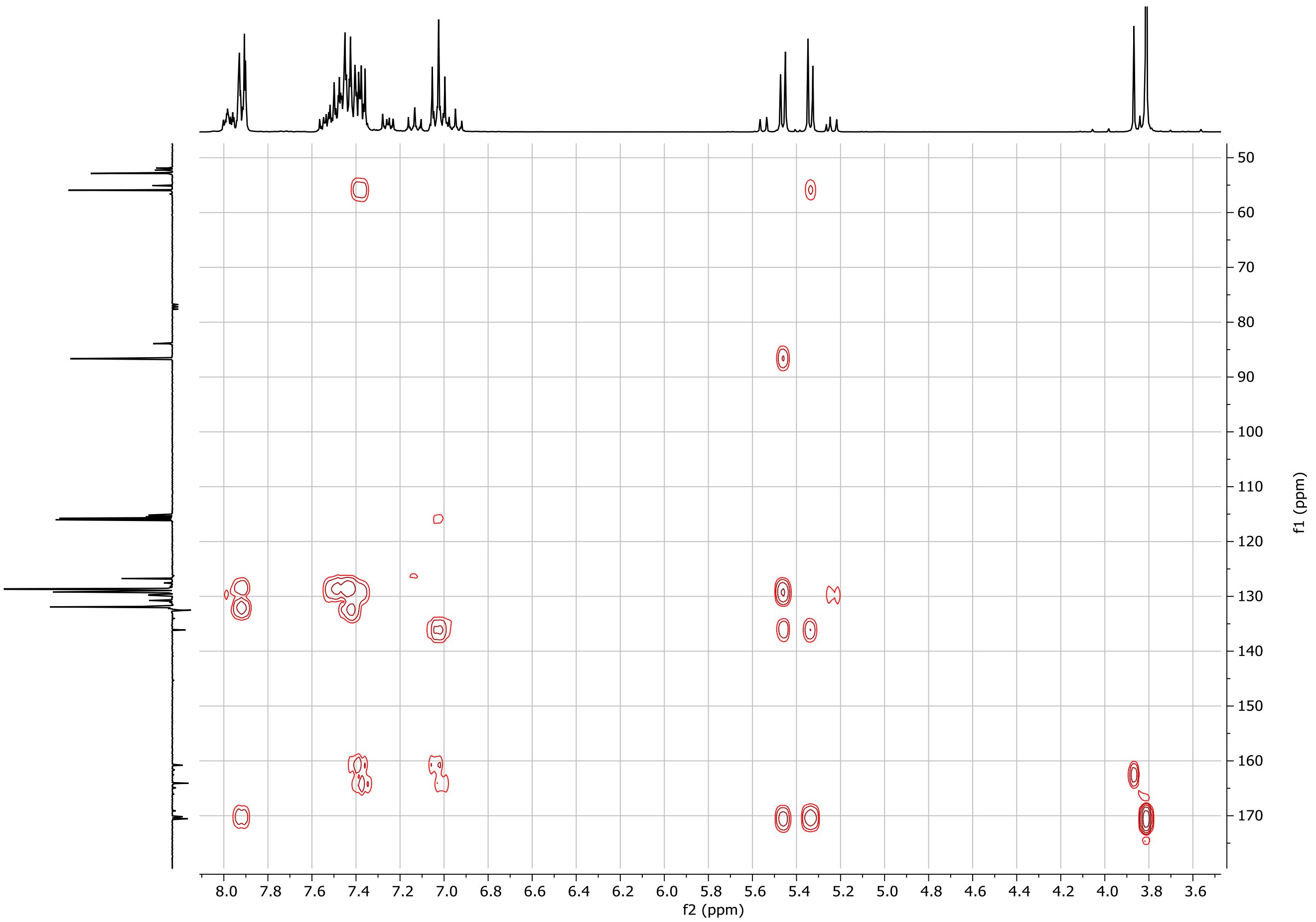

^{1}H-^{13}C HMBC correlation spectrum of **5d**

Ethyl 5-(4-methoxyphenyl)-2-phenyl-4,5-dihydrothiazole-4-carboxylate 6c

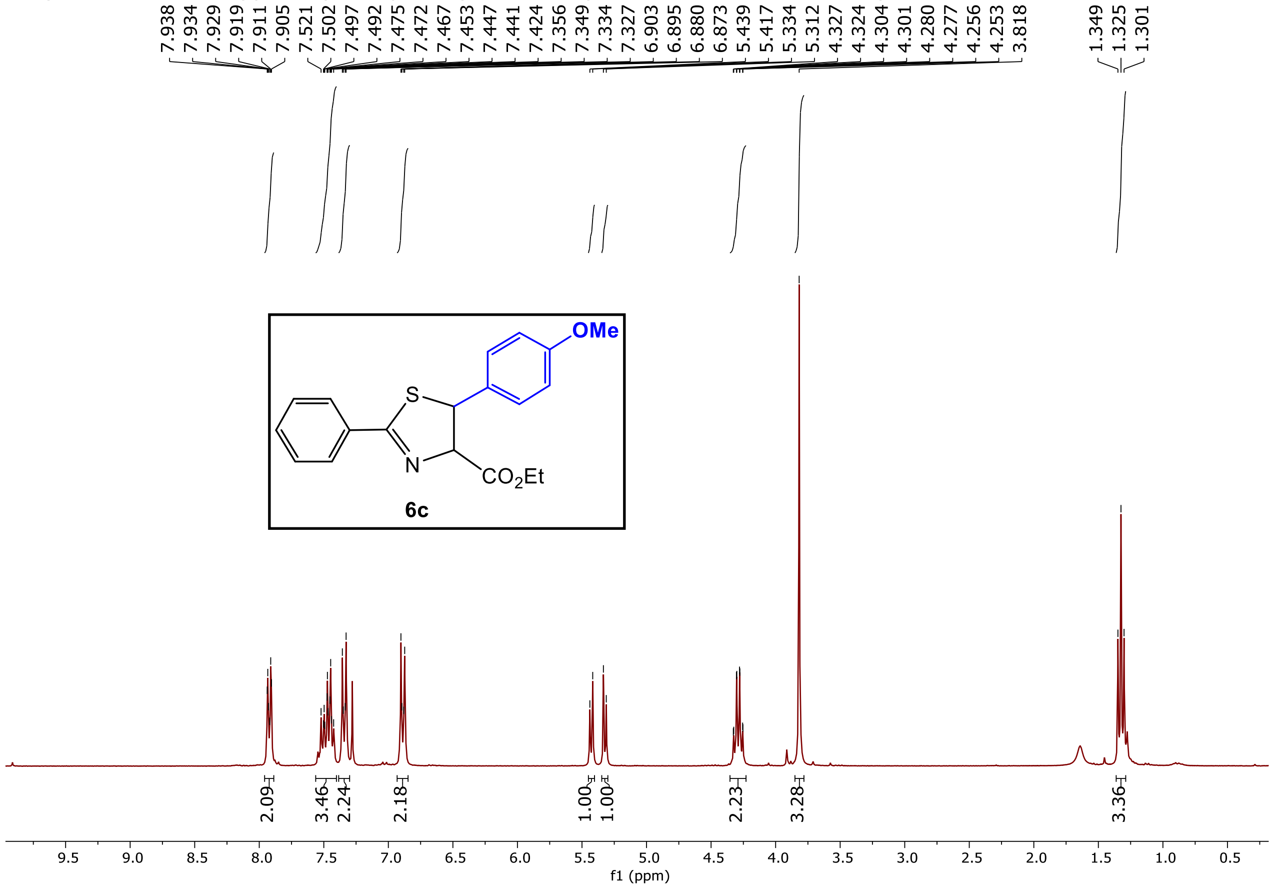

^{1}H NMR spectrum ($CDCl_3$, 300.13 MHz) of **6c**

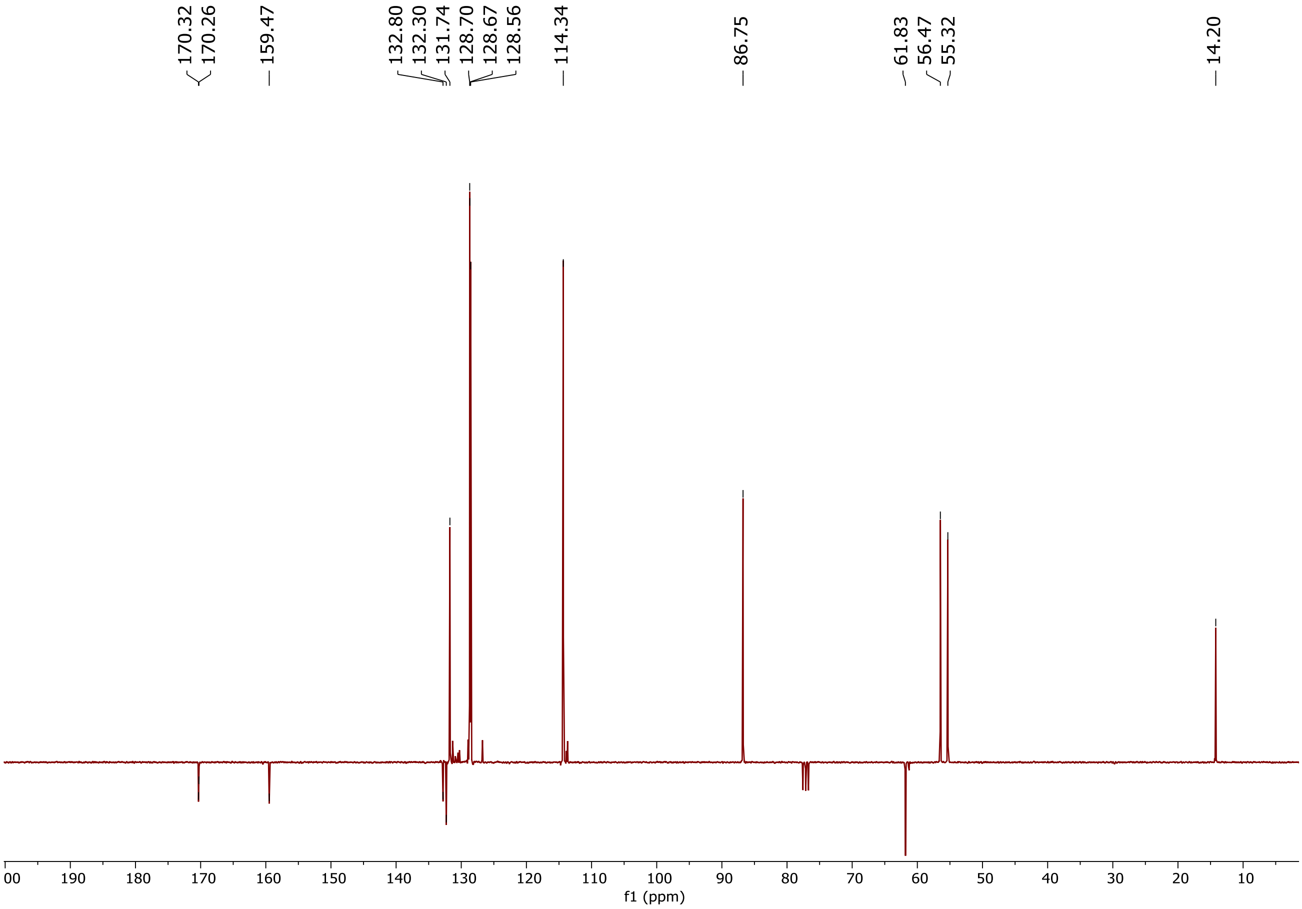

$^{13}C\{^{1}H\}$ (APT) NMR spectrum ($CDCl_3$, 75.47 MHz) of **6c**

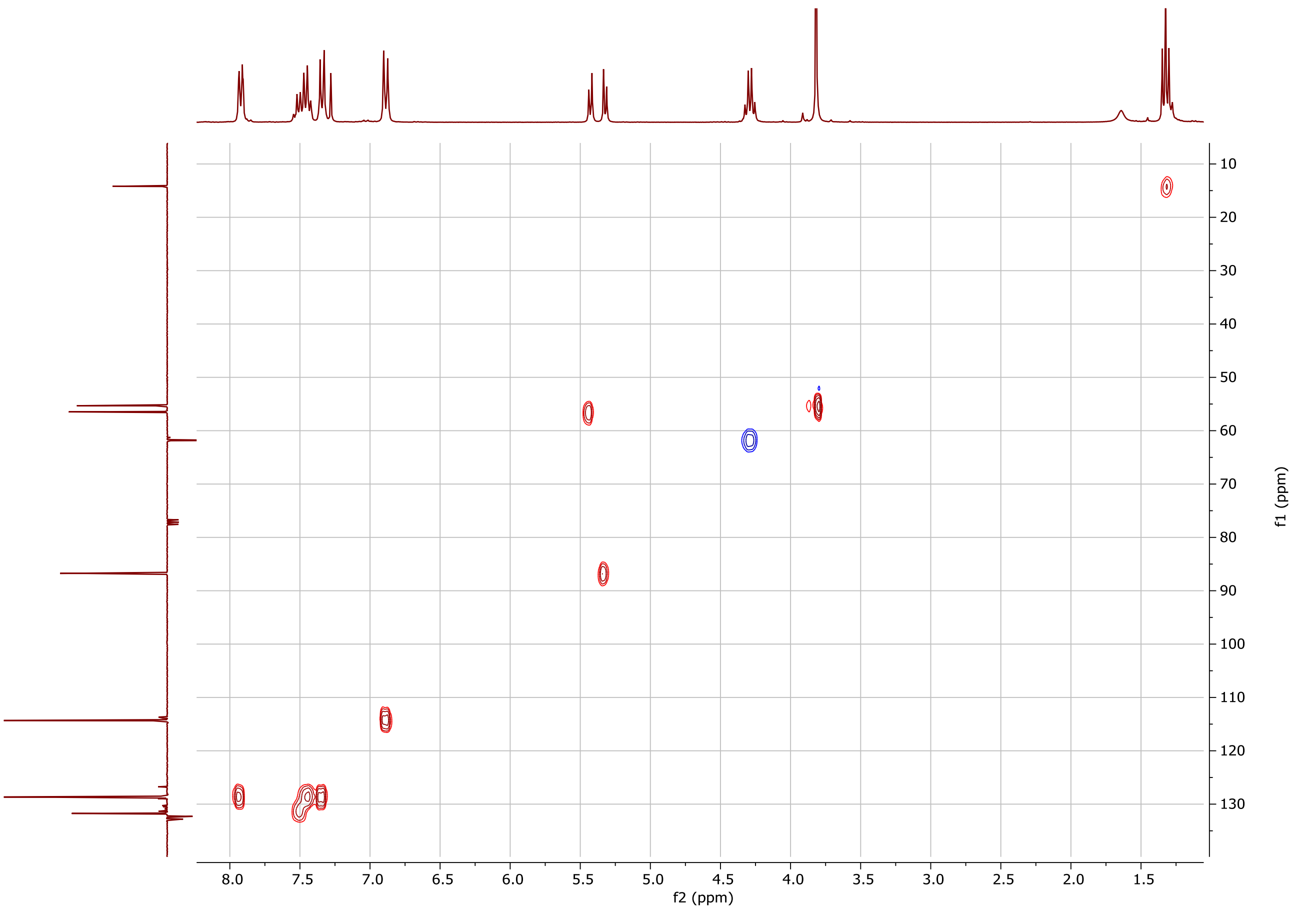

^{1}H-^{13}C HSQC correlation spectrum of **6c**

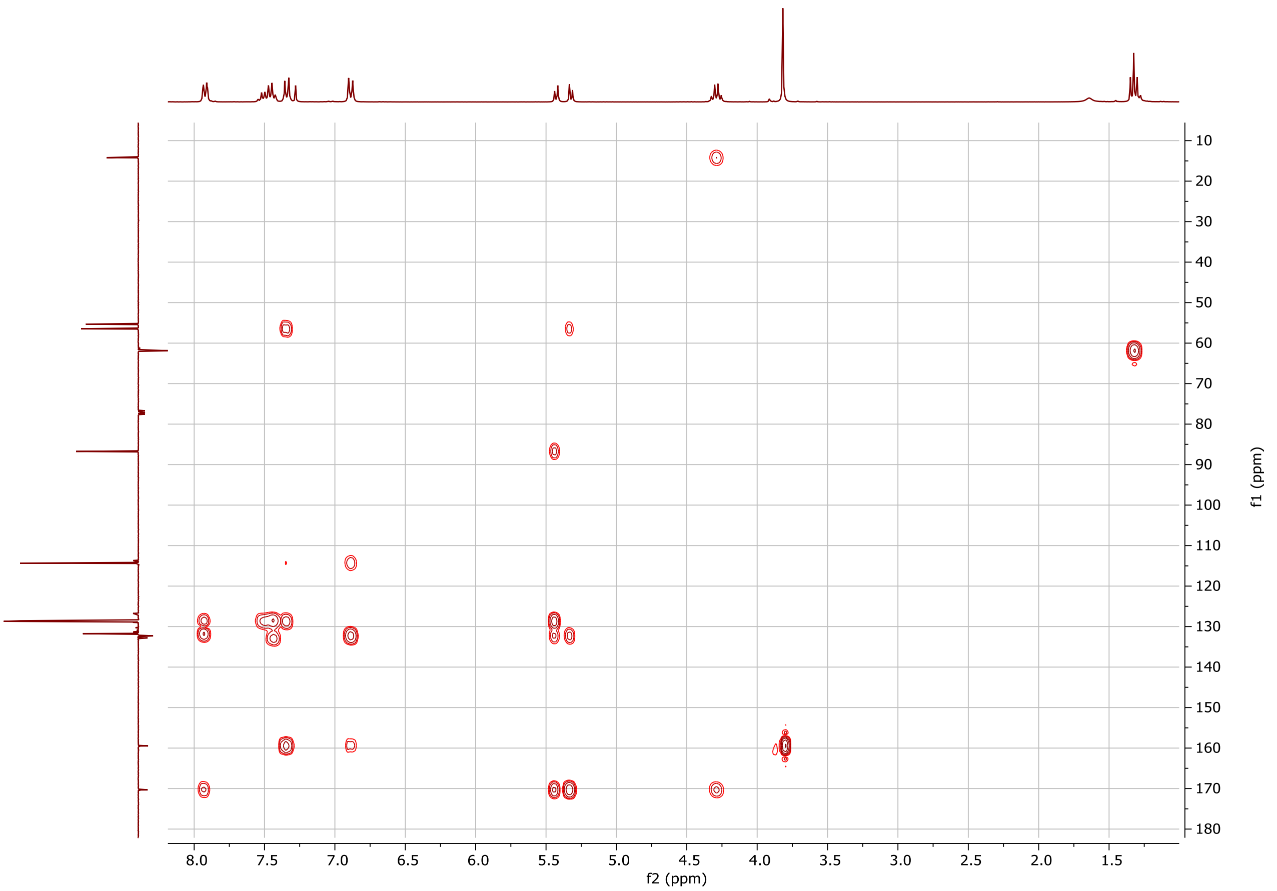

^{1}H-^{13}C HMBC correlation spectrum of **6c**

Ethyl 5-(4-(trifluoromethyl)phenyl)-2-phenyl-4,5-dihydrothiazole-4-carboxylate 6h

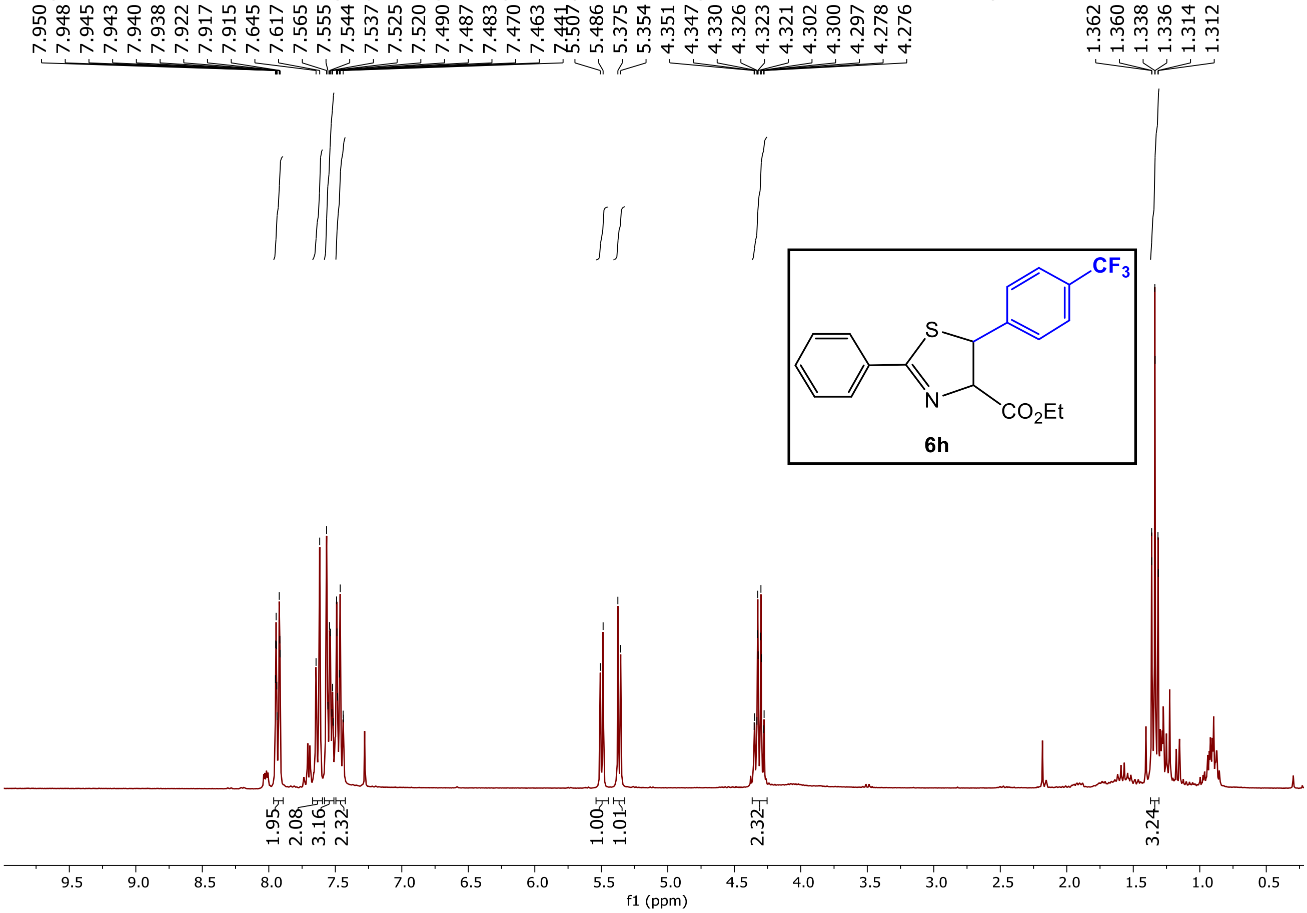

^{1}H NMR spectrum ($CDCl_3$, 300.13 MHz) of **6h**

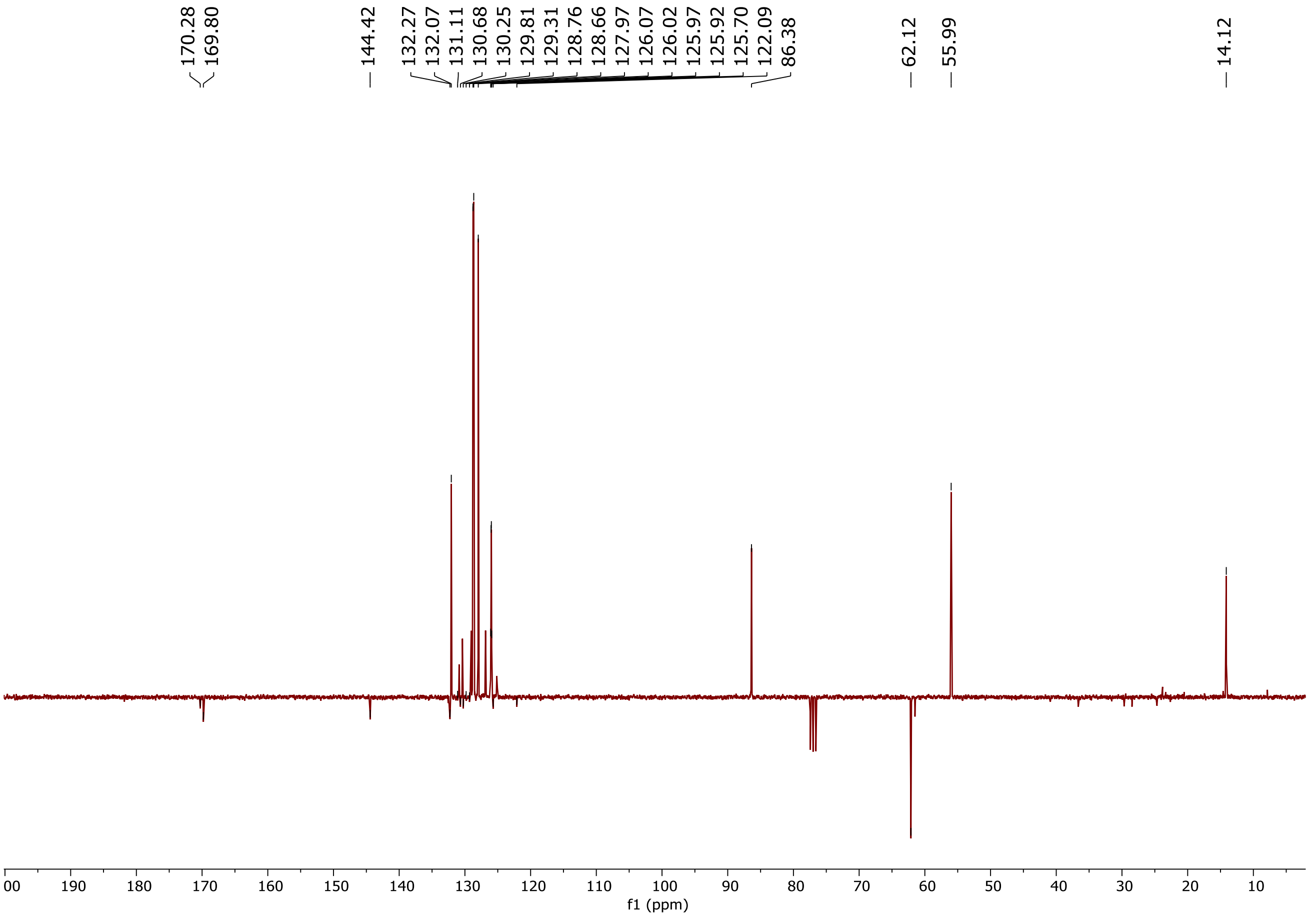

^{13}C{^{1}H} (APT) NMR spectrum ($CDCl_3$, 75.47 MHz) of **6h**

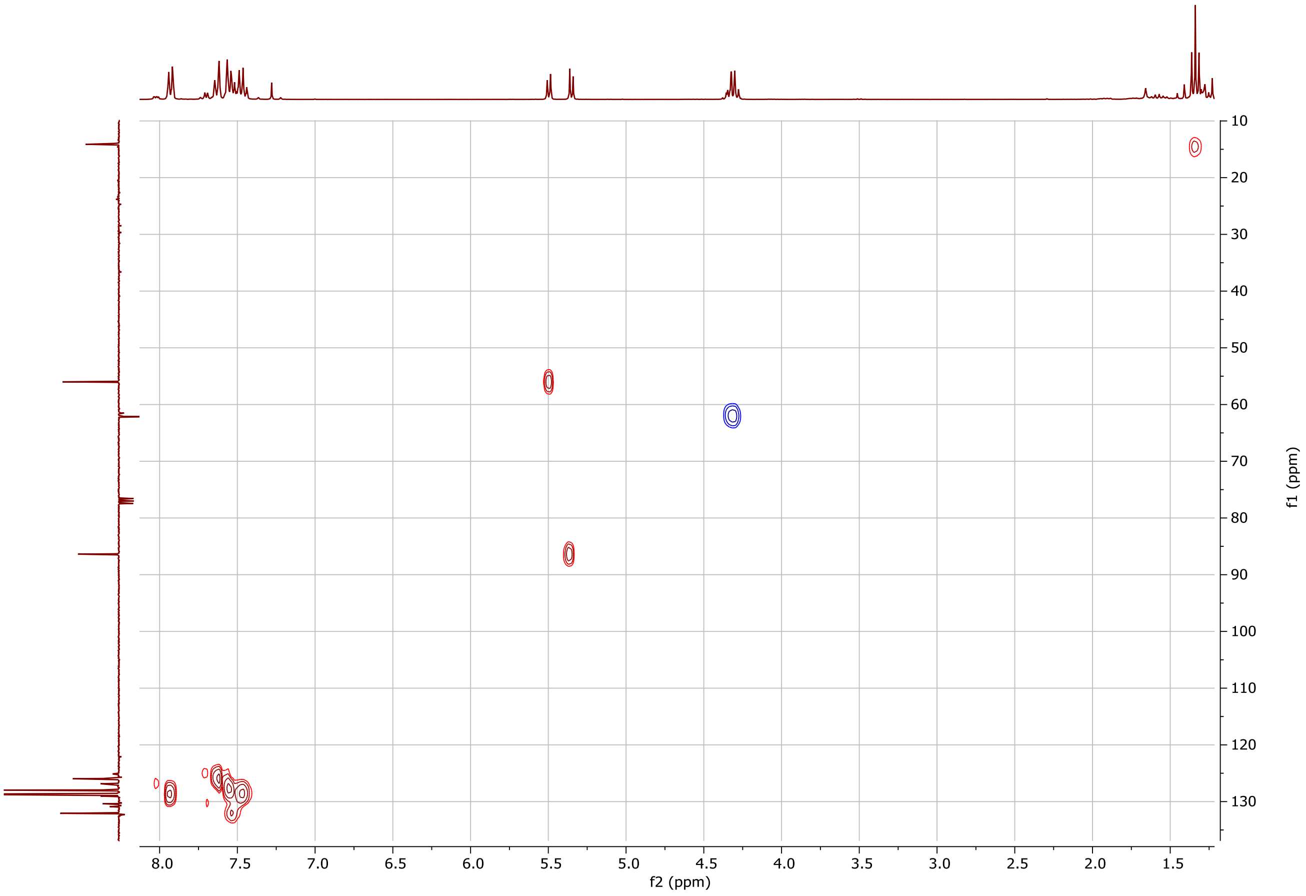

^{1}H-^{13}C HSQC correlation spectrum of **6h**

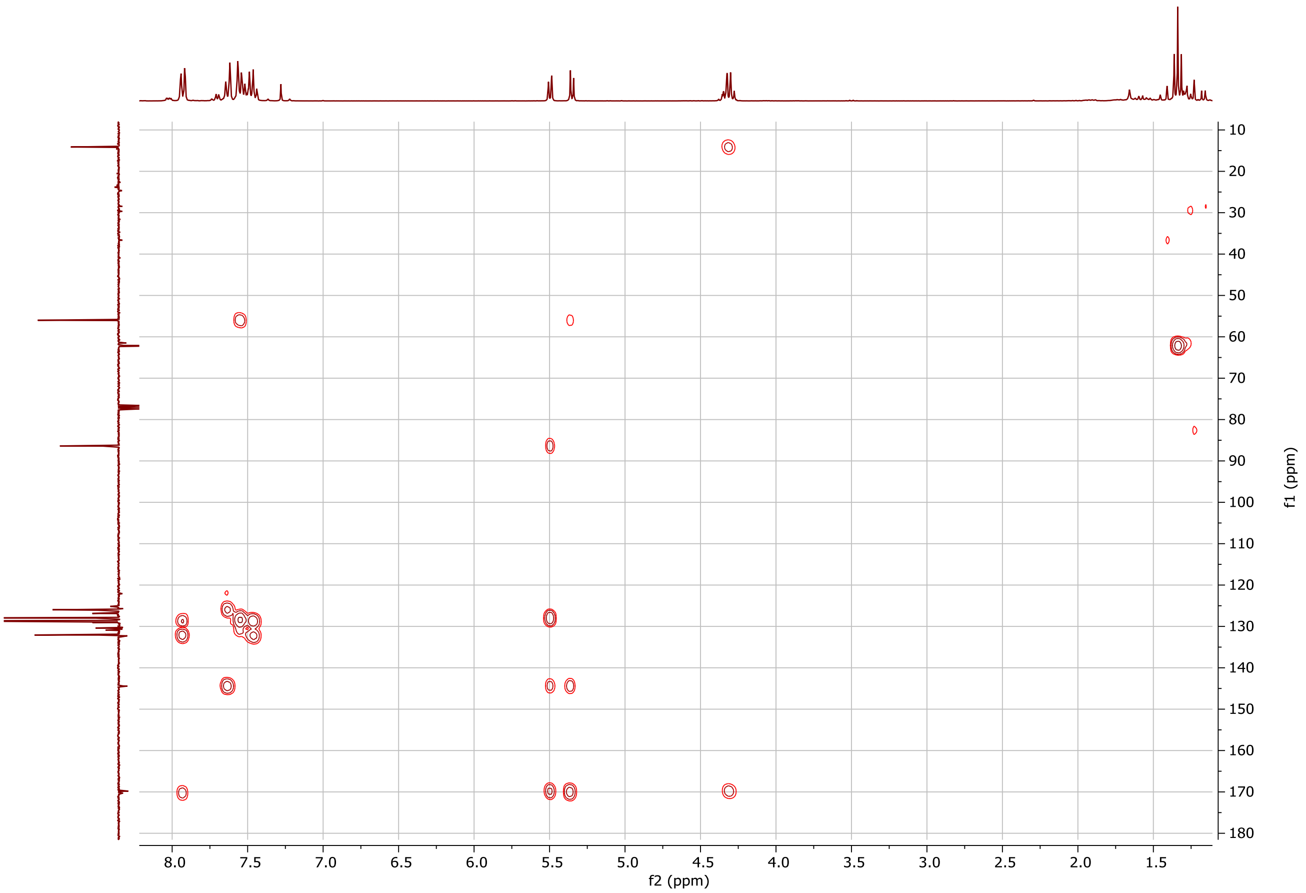

^{1}H-^{13}C HMBC correlation spectrum of **6h**

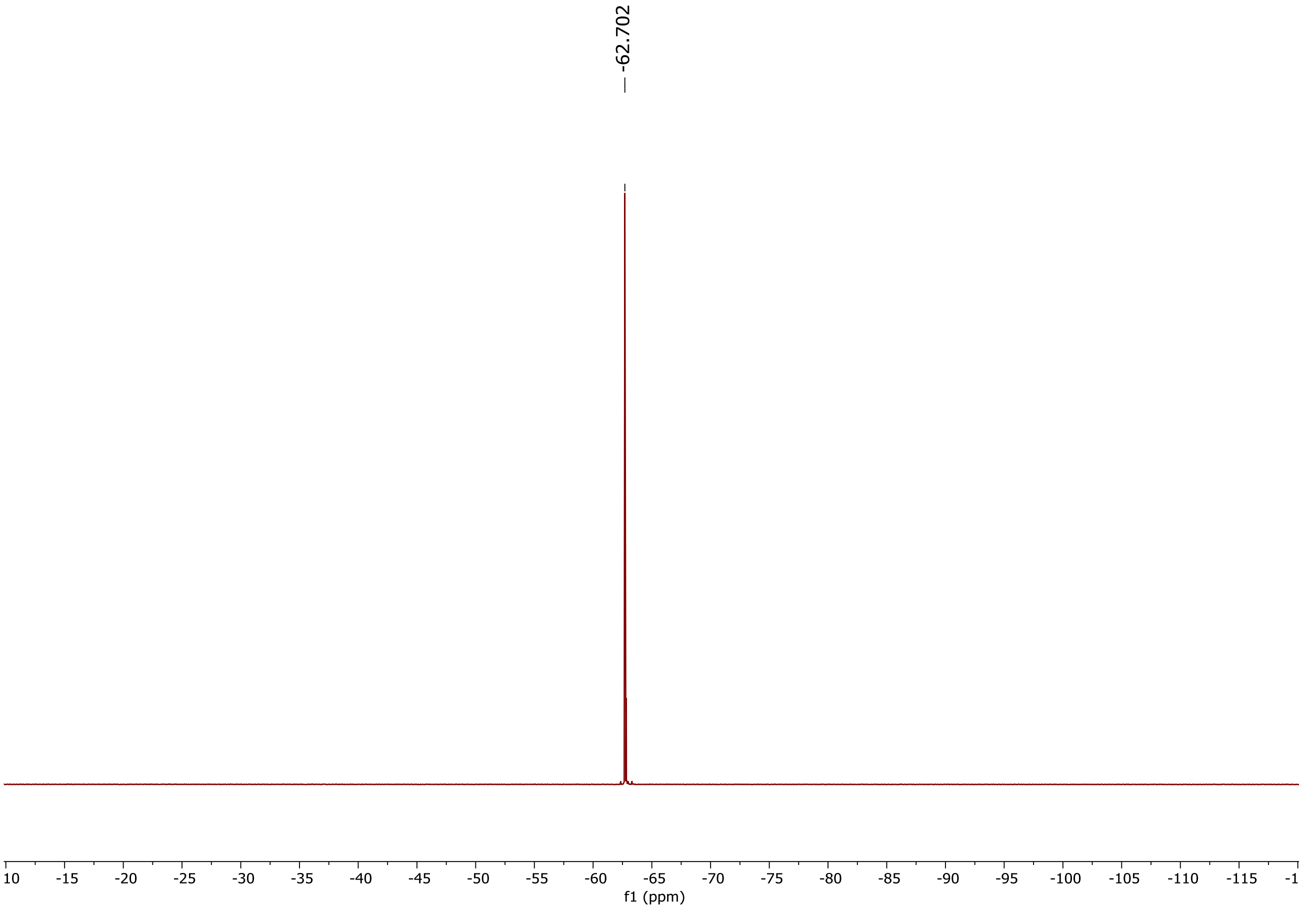

^{19}F NMR spectrum ($CDCl_3$, 282.4 MHz) of **6h**

Ethyl 5-(2-methoxyphenyl)-2-phenyl-4,5-dihydrothiazole-4-carboxylate 6i

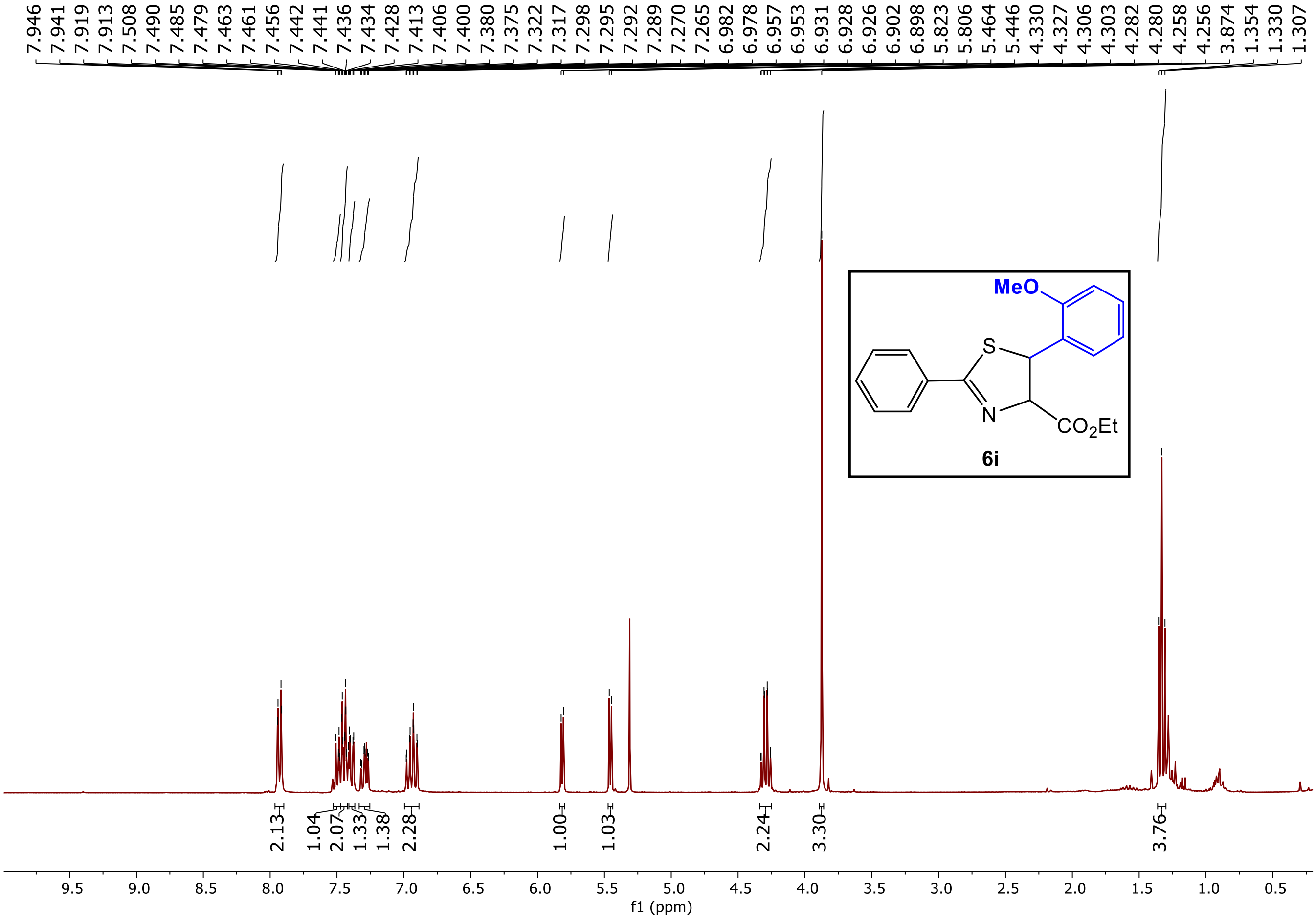

^{1}H NMR spectrum ($CDCl_3$, 300.13 MHz) of **6i**

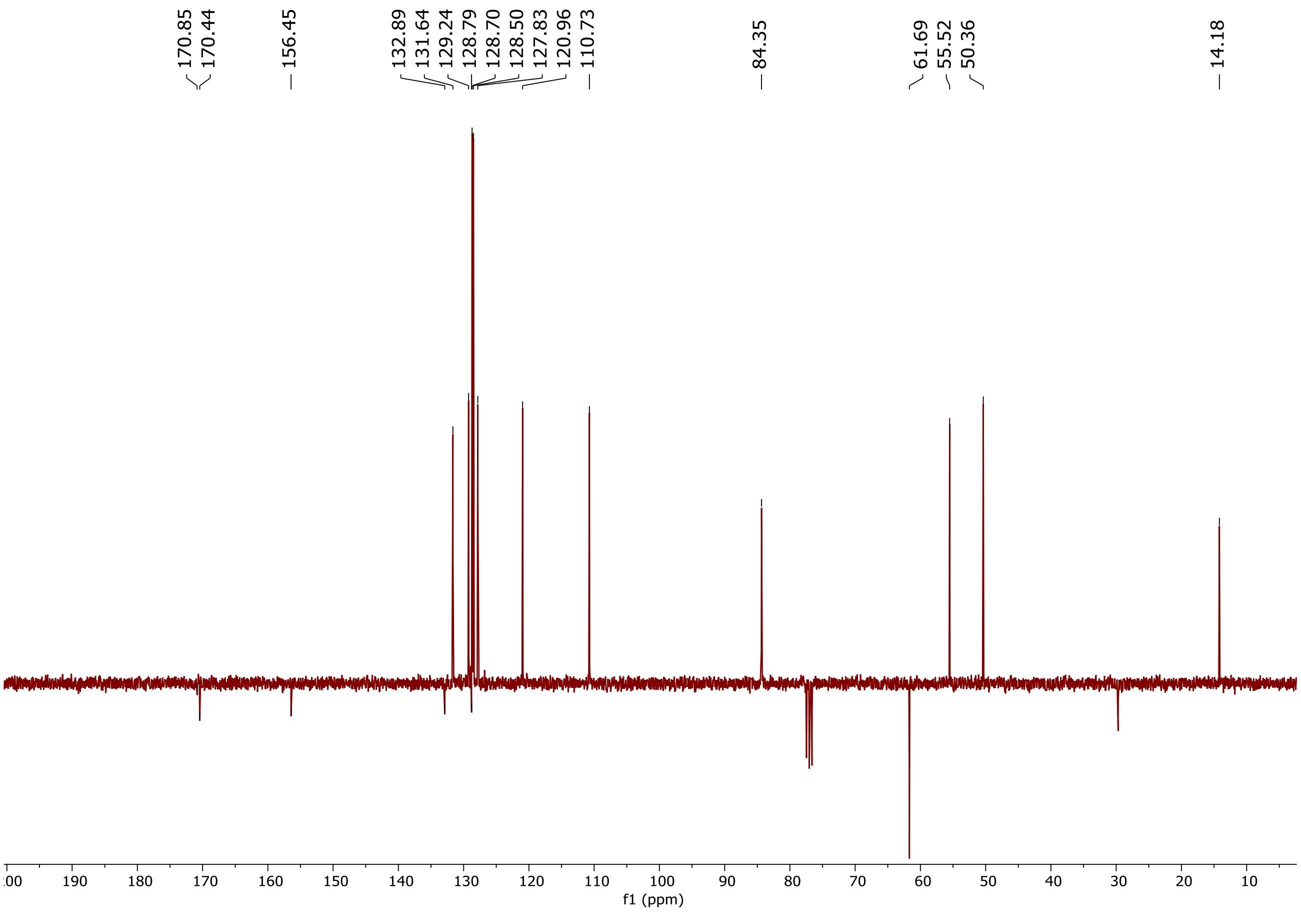

$^{13}C\{^{1}H\}$ (APT) NMR spectrum ($CDCl_3$, 75.47 MHz) of **6i**

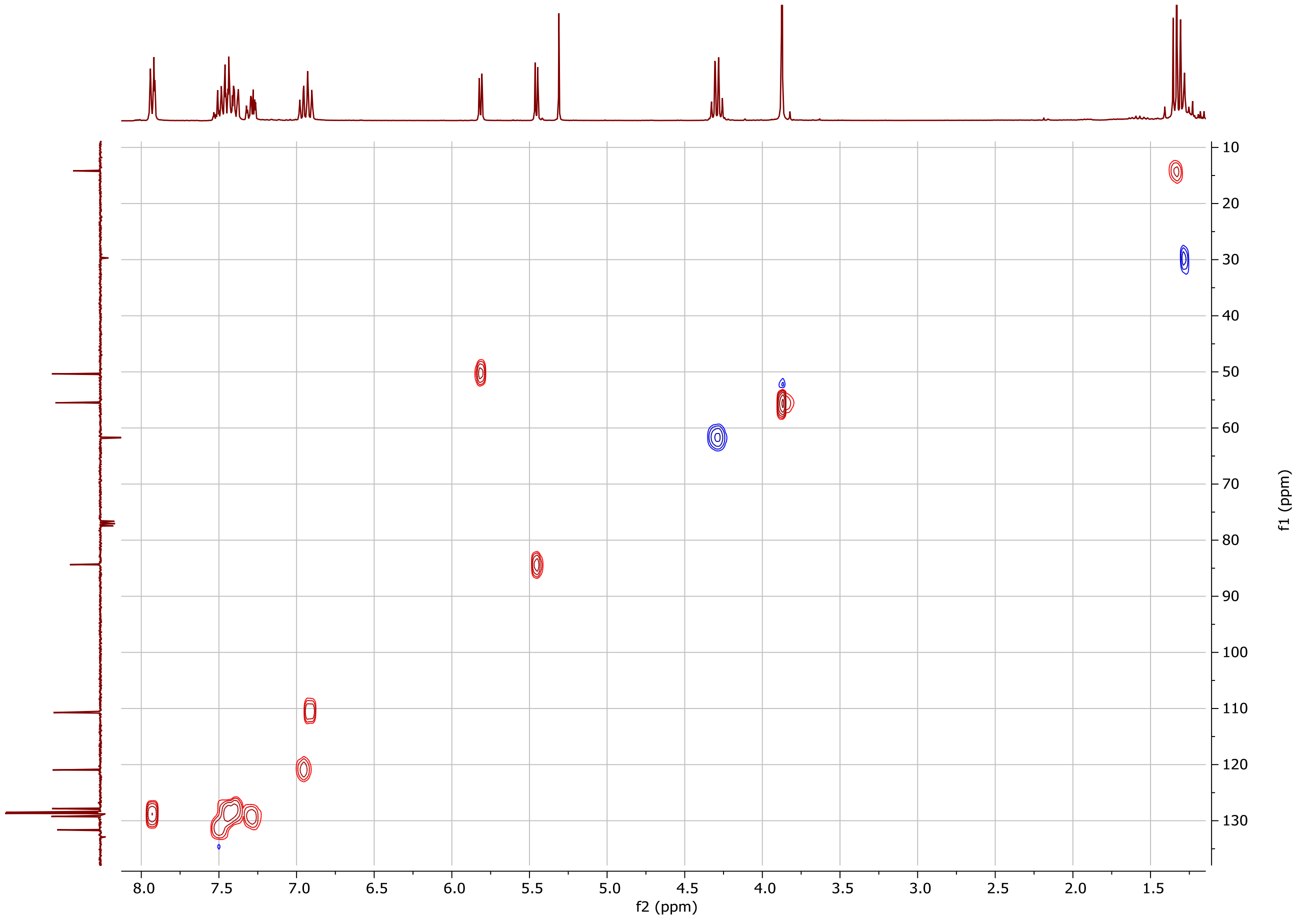

^{1}H-^{13}C HSQC correlation spectrum of **6i**

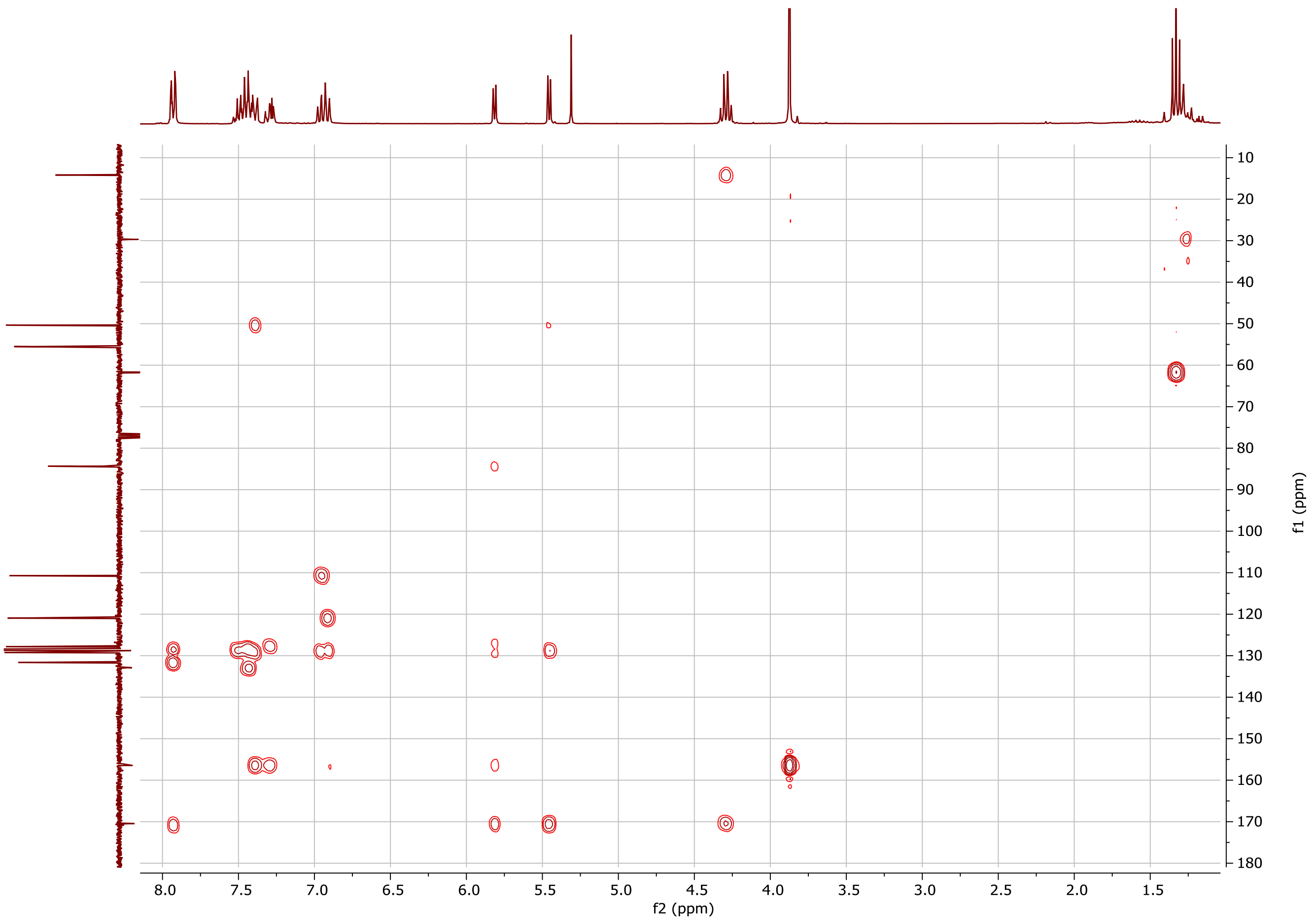

^1H-^{13}C HMBC correlation spectrum of **6i**

Ethyl 5-(3,4-dimethylphenyl)-2-phenyl-4,5-dihydrothiazole-4-carboxylate 6m

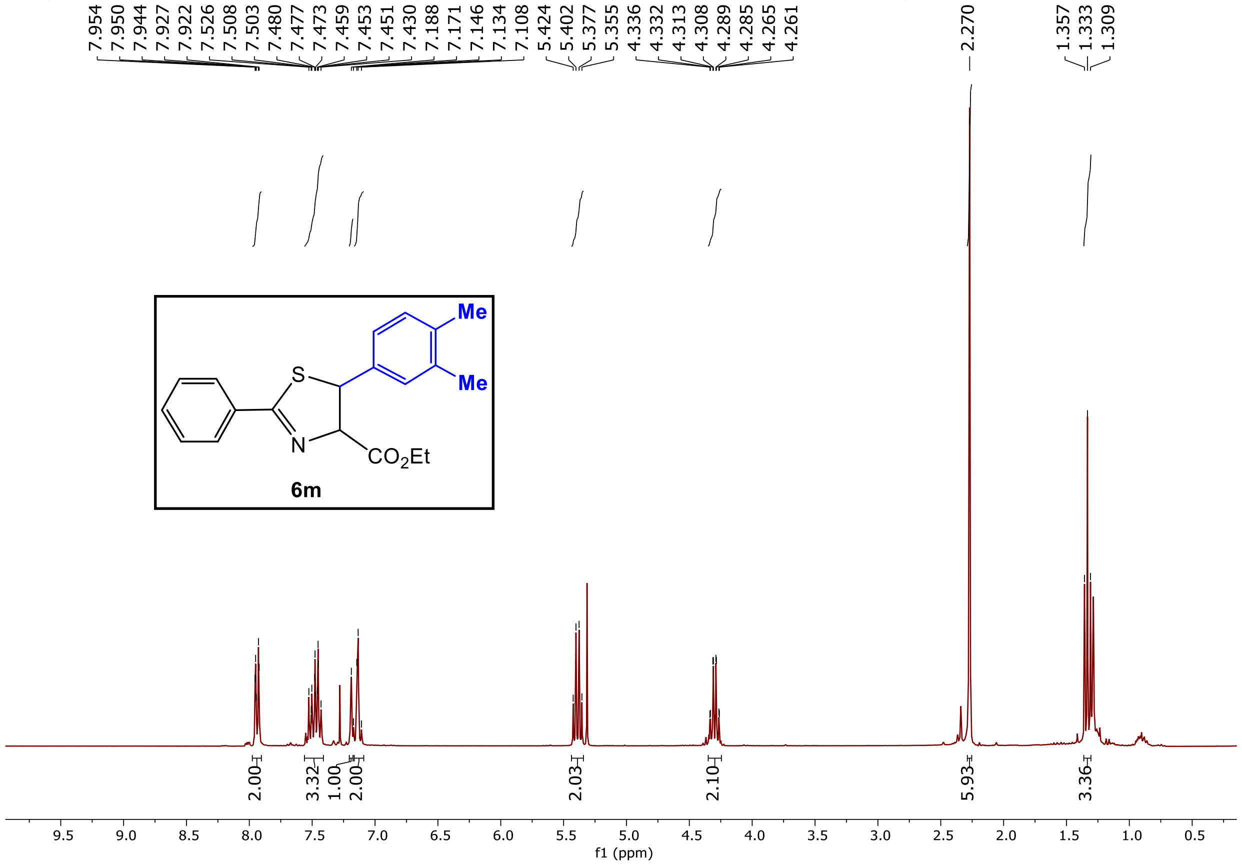

^1H NMR spectrum ($CDCl_3$, 300.13 MHz) of **6m**

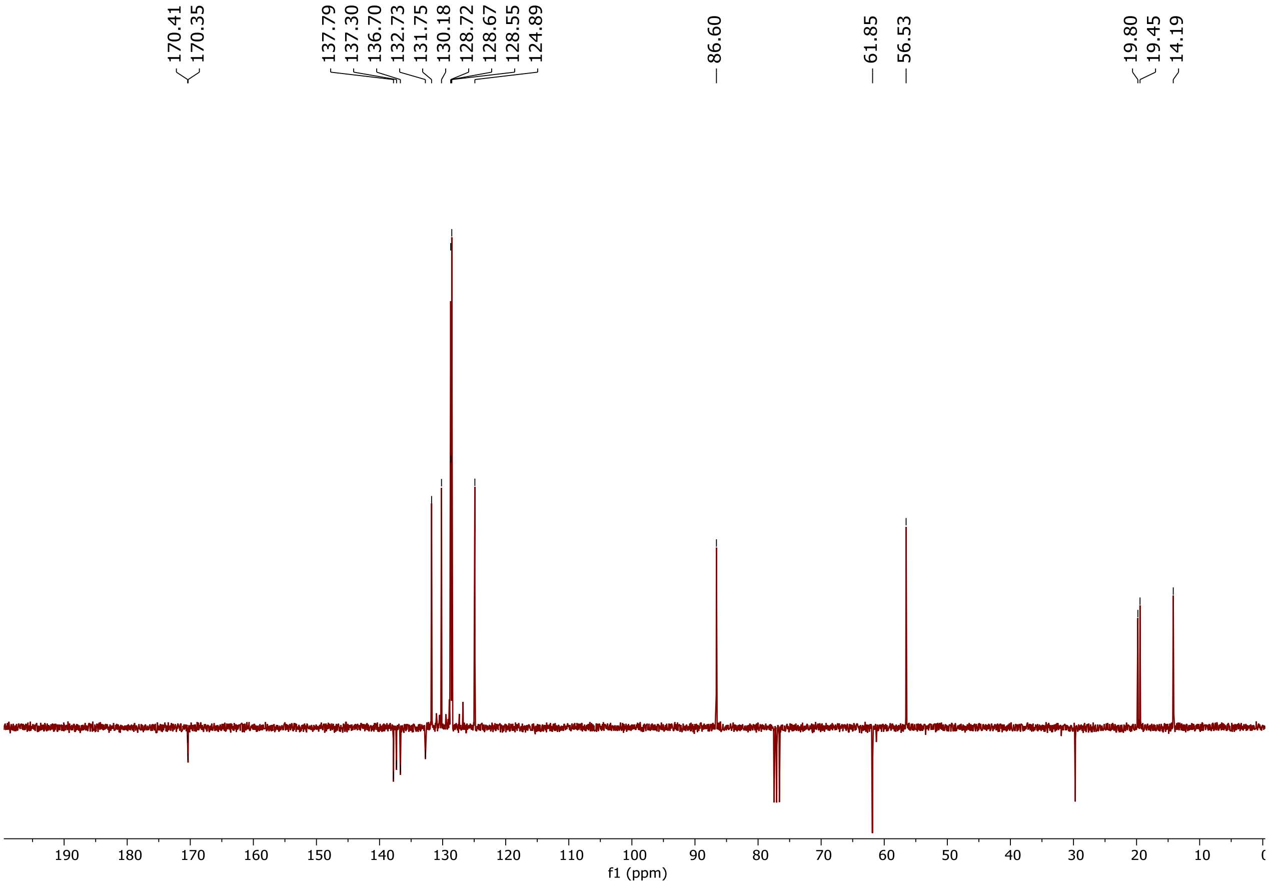

$^{13}C\{^{1}H\}$ NMR spectrum ($CDCl_3$, 75.47 MHz) of **6m**

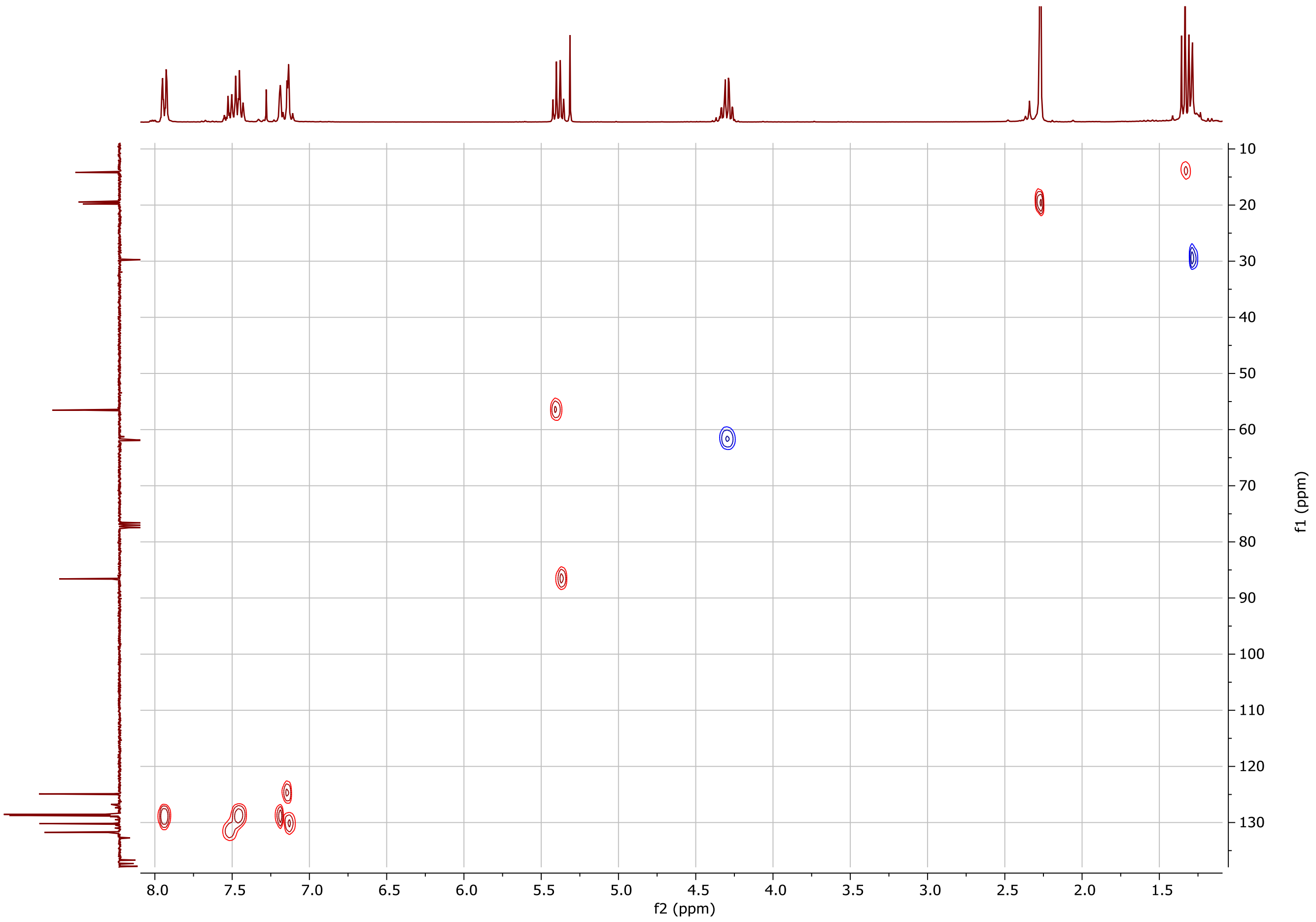

^{1}H-^{13}C HSQC correlation spectrum of **6m**

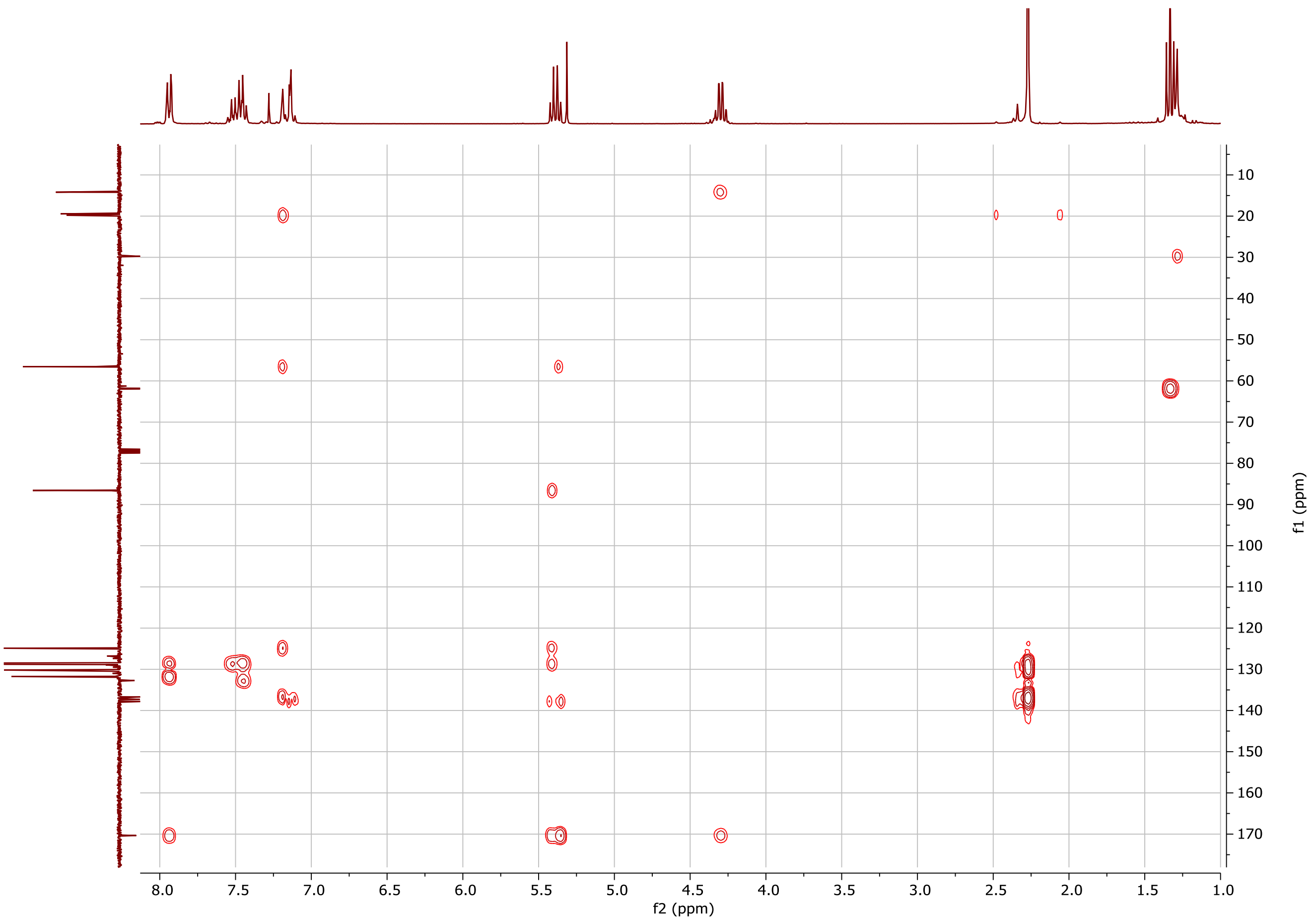

^1H-^{13}C HMBC correlation spectrum of **6m**

Ethyl 5-(3,4-dichlorophenyl)-2-phenyl-4,5-dihydrothiazole-4-carboxylate 6n

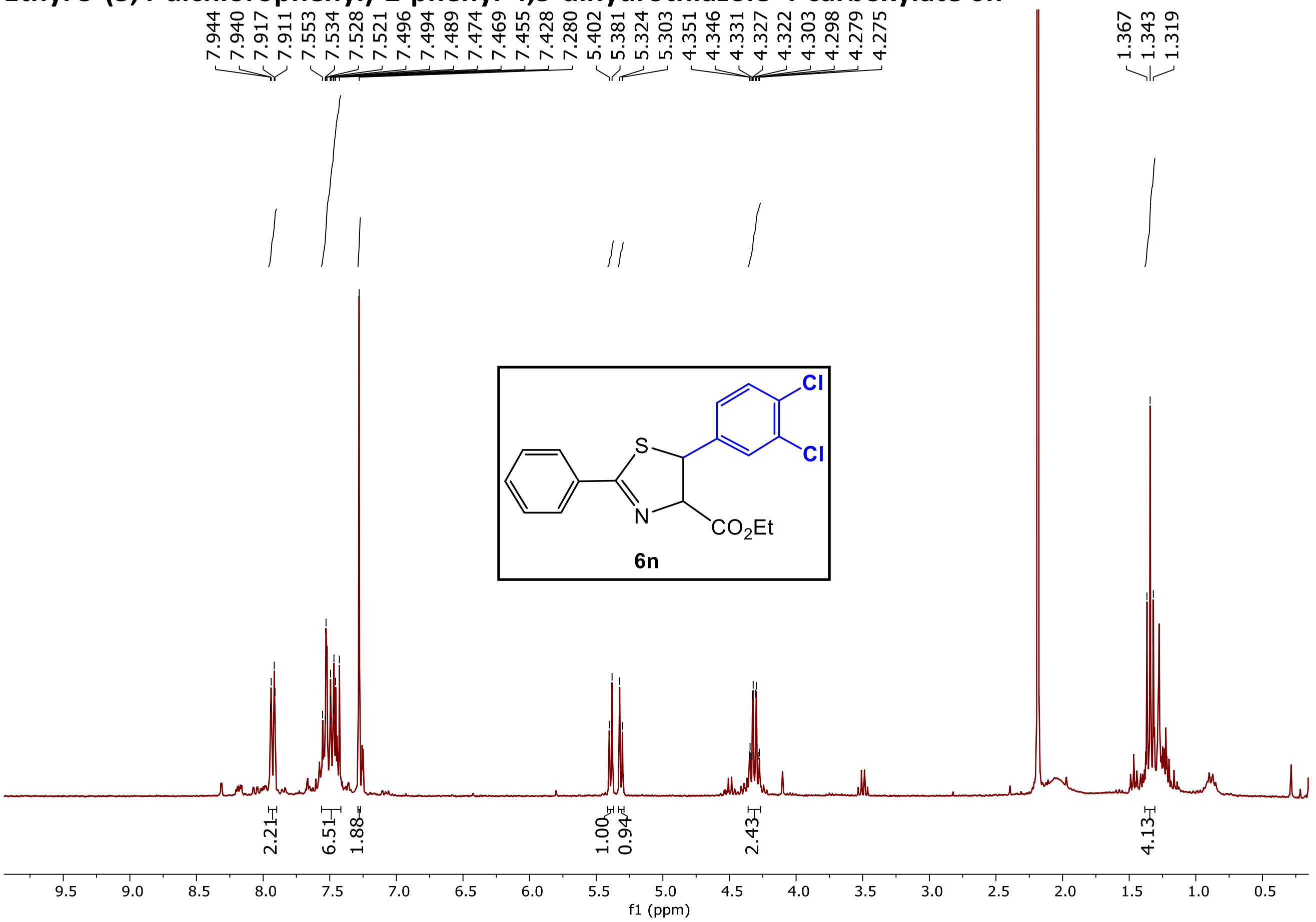

^1H NMR spectrum ($CDCl_3$, 300.13 MHz) of **6n**

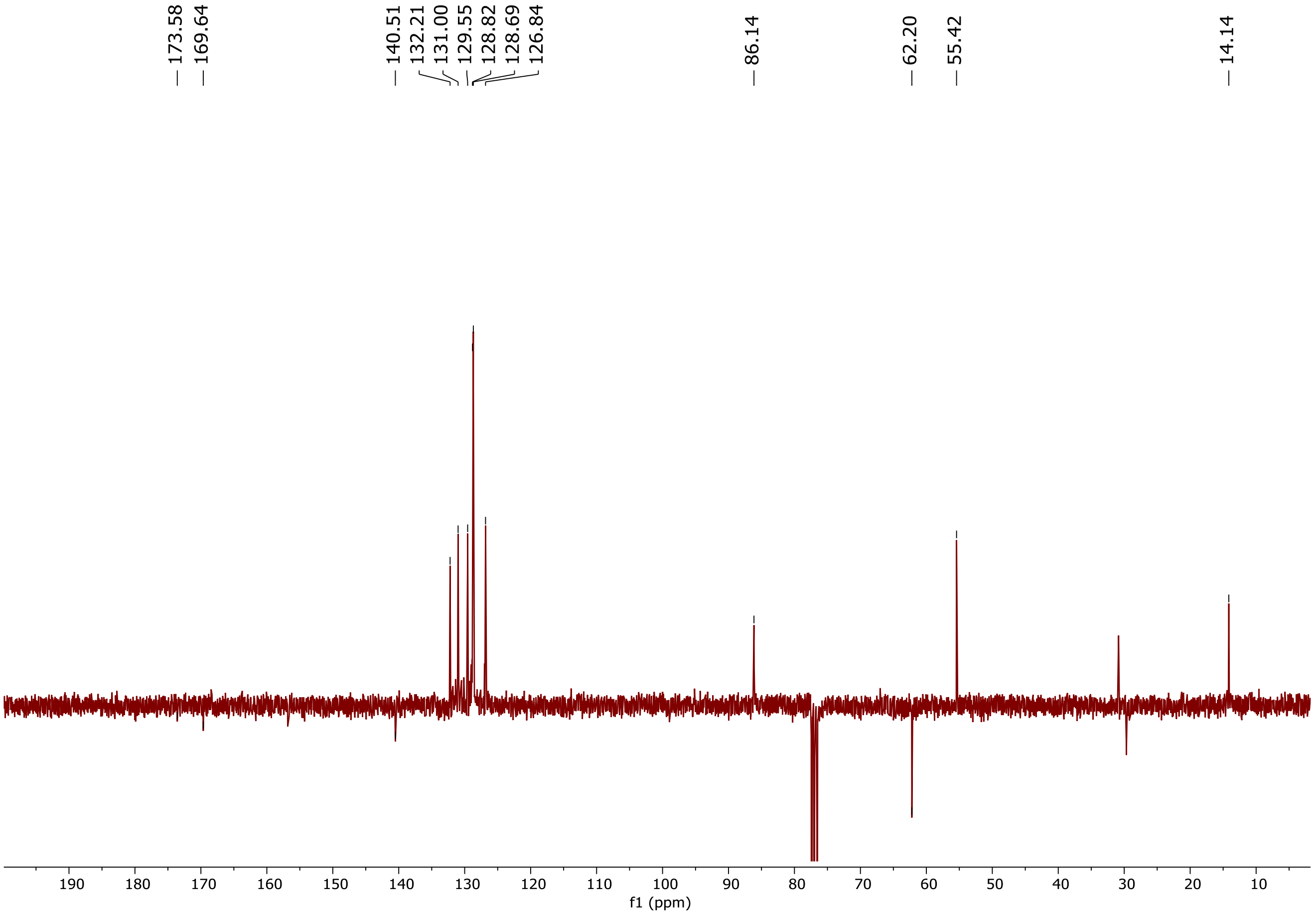

$^{13}C\{^{1}H\}$ NMR spectrum ($CDCl_3$, 75.47 MHz) of **6n**

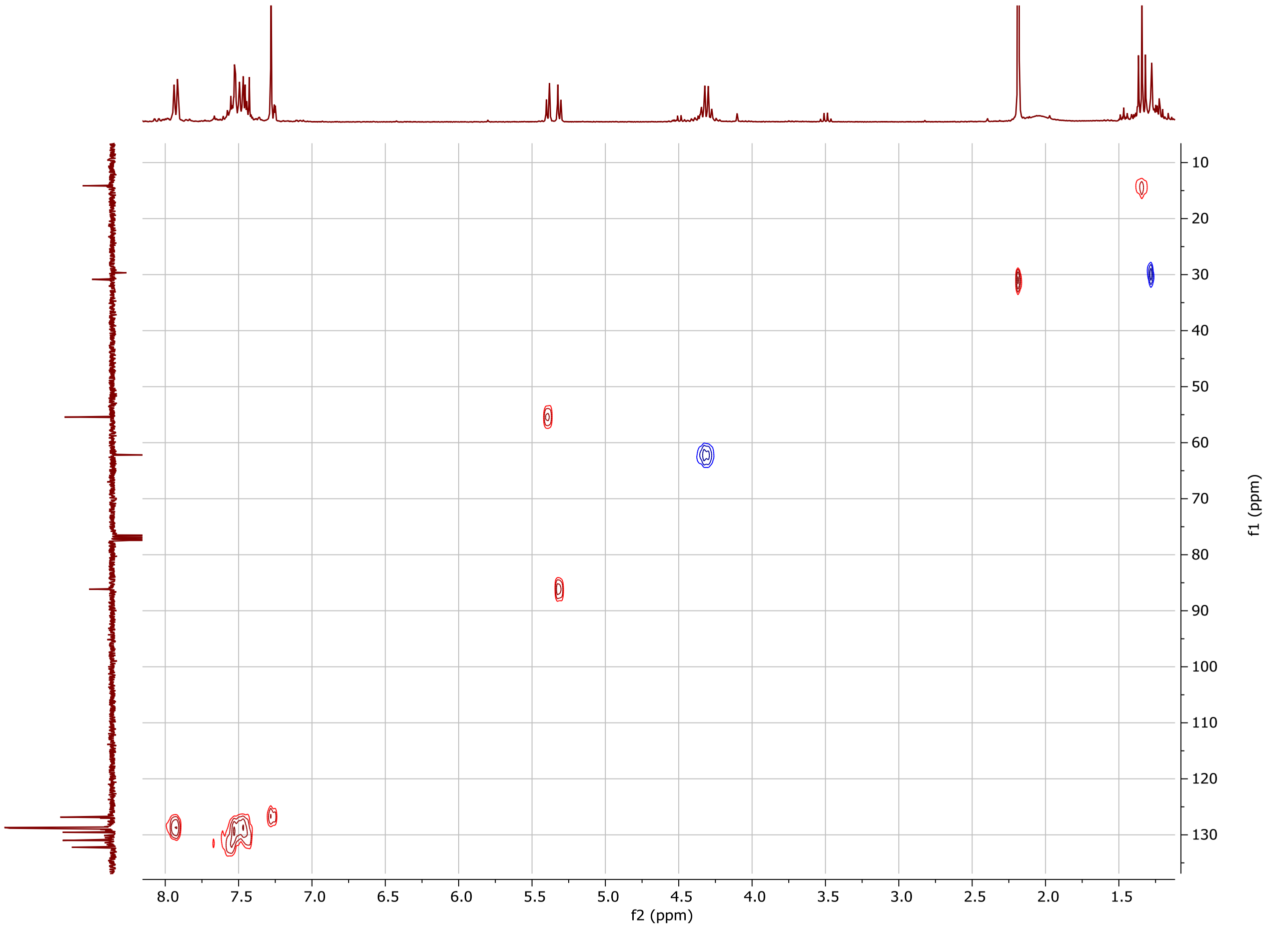

^{1}H-^{13}C HSQC correlation spectrum of **6n**

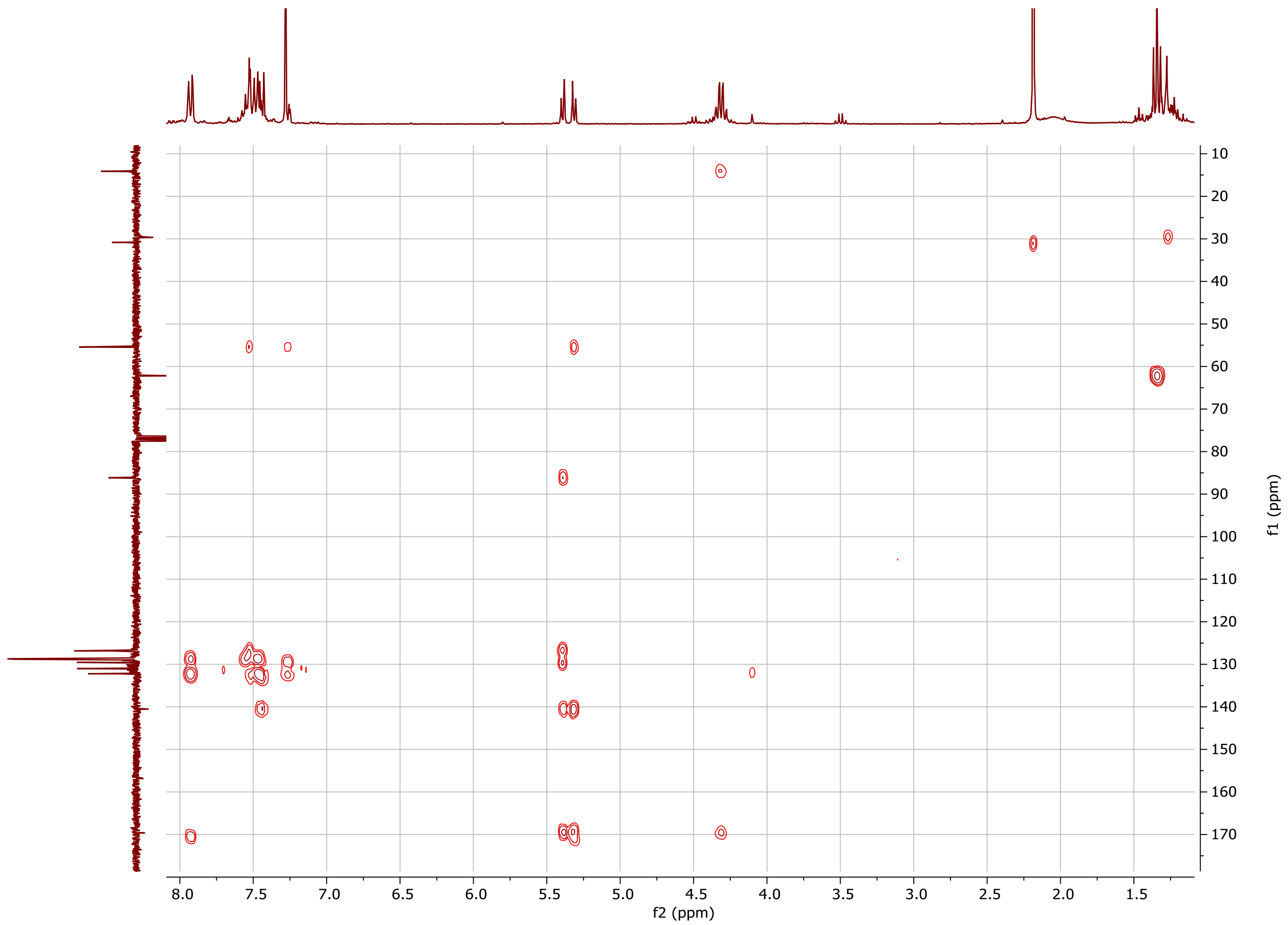

^1H-^{13}C HMBC correlation spectrum of **6n**

Ethyl 5-(3,4-dichlorophenyl)-2-phenyl-4,5-thiazole-4-carboxylate 7n

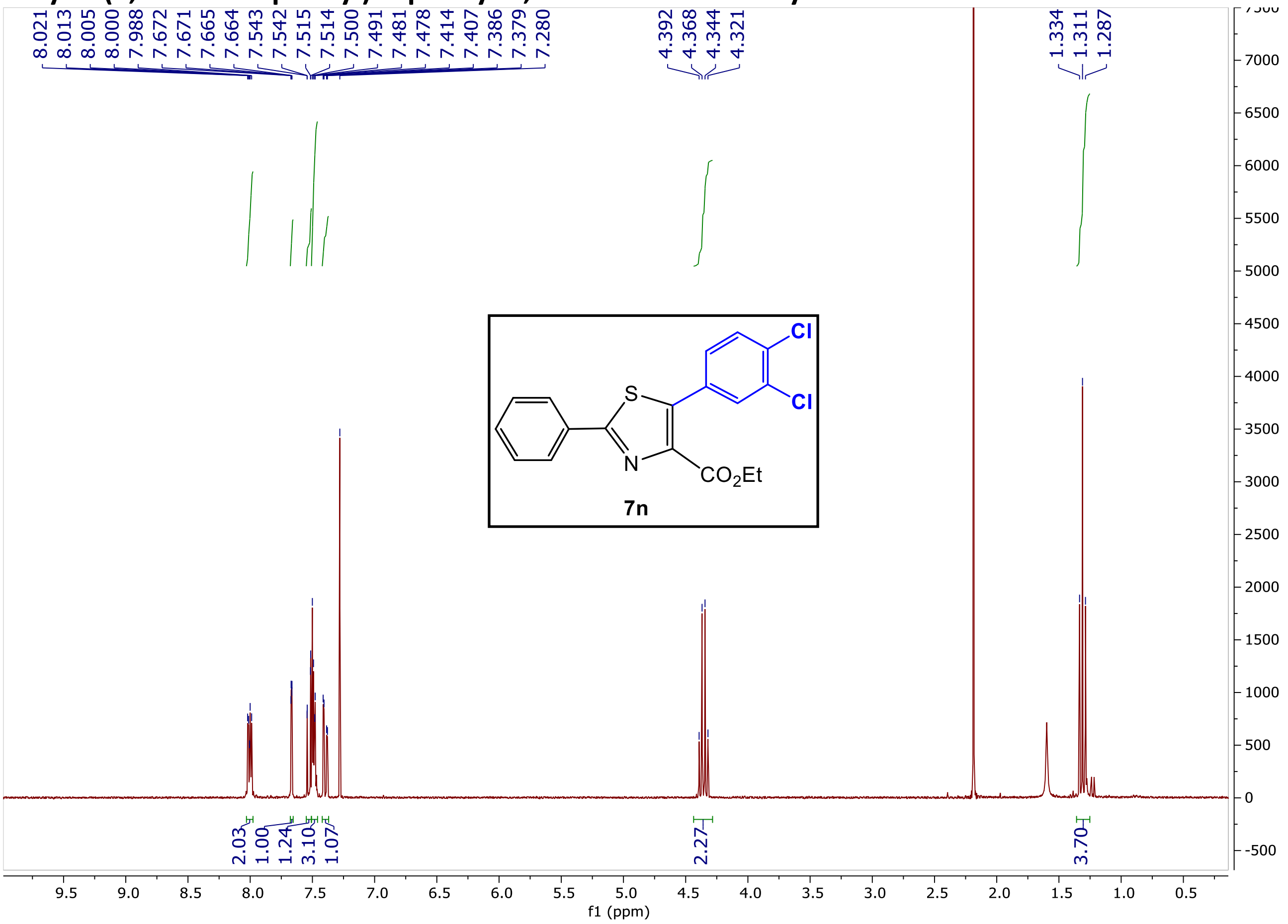

^1H NMR spectrum ($CDCl_3$, 300.13 MHz) of **7n**

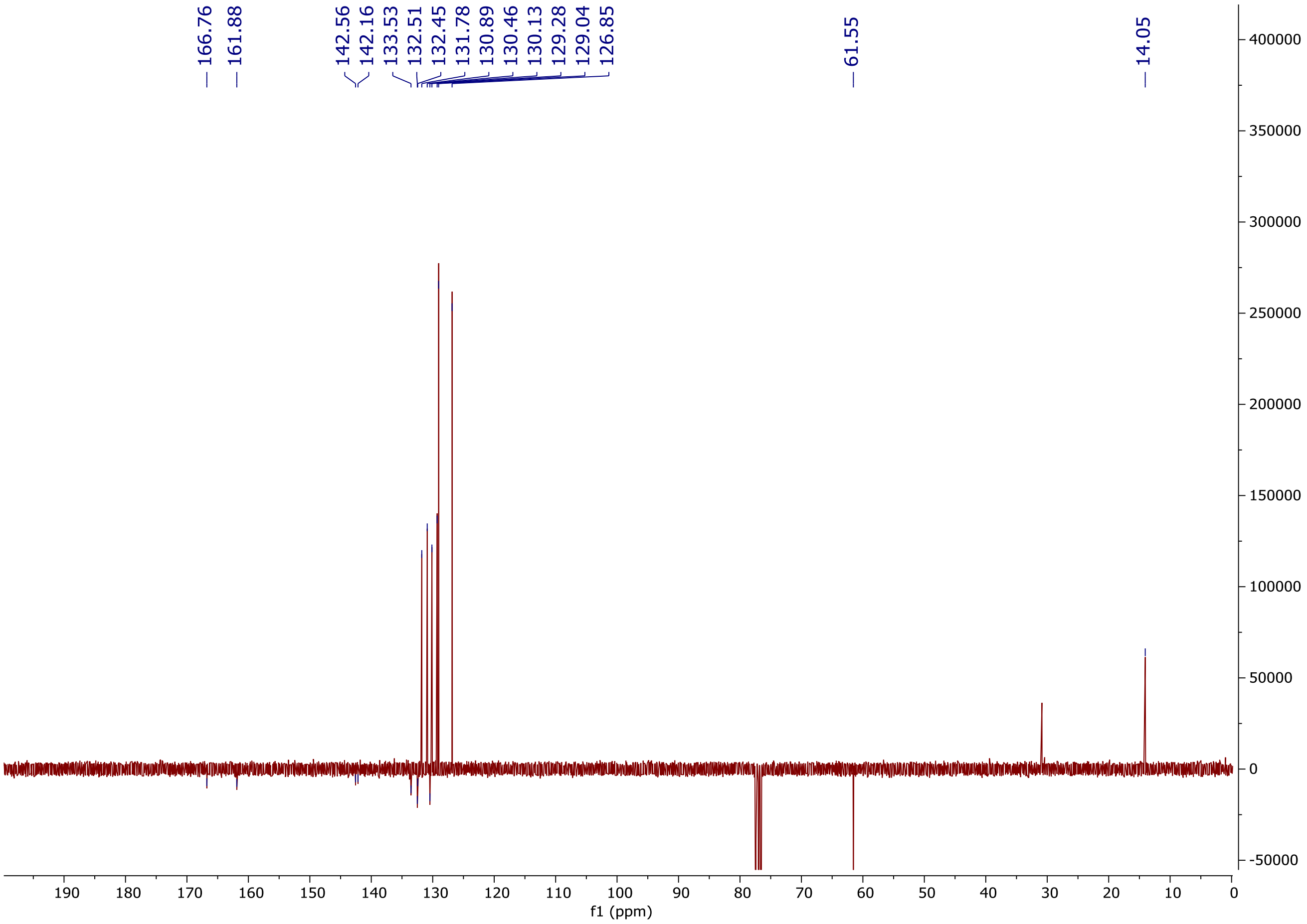

$^{13}C\{^{1}H\}$ NMR spectrum ($CDCl_3$, 75.47 MHz) of **7n**

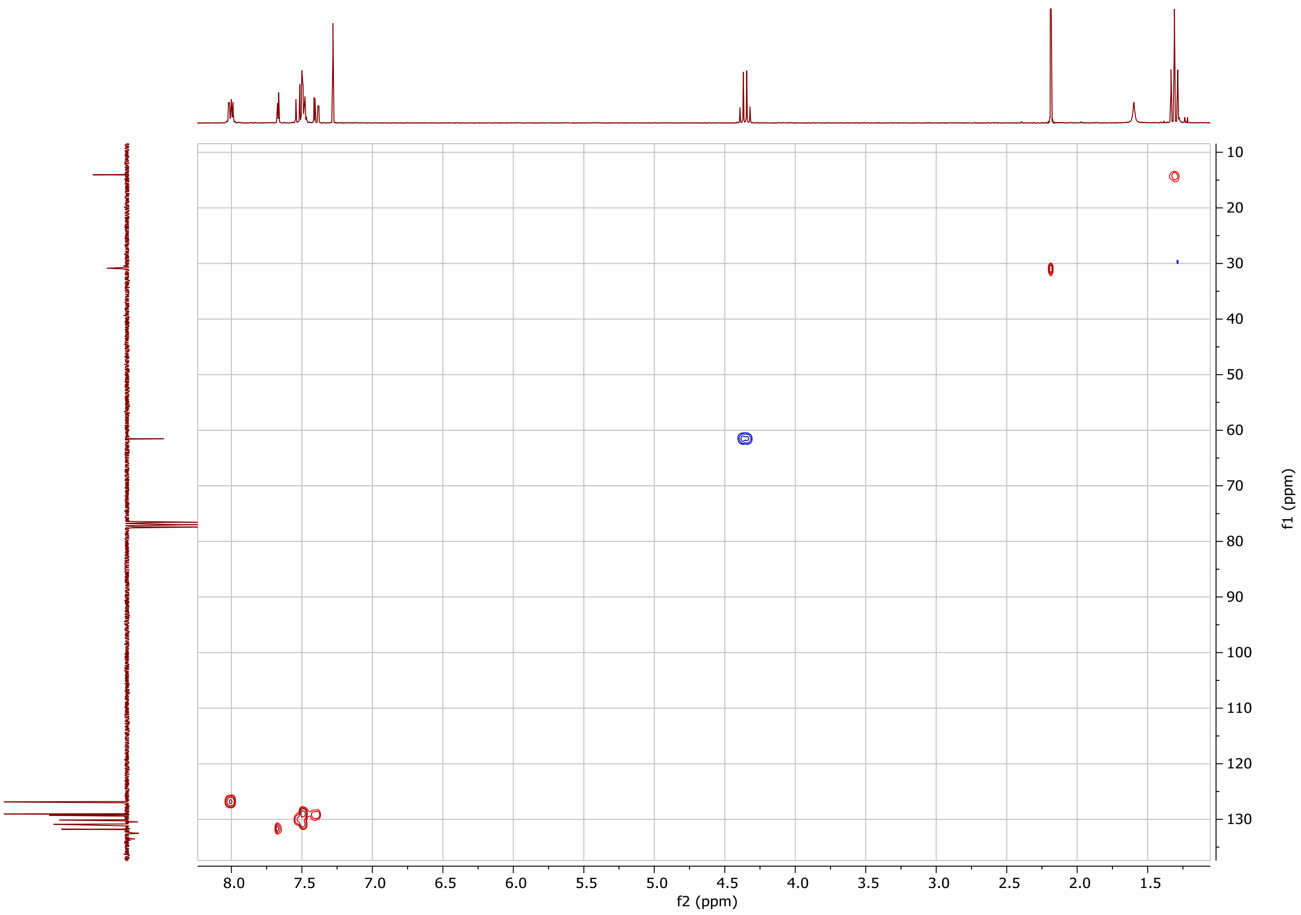

^{1}H-^{13}C HSQC correlation spectrum of **7n**

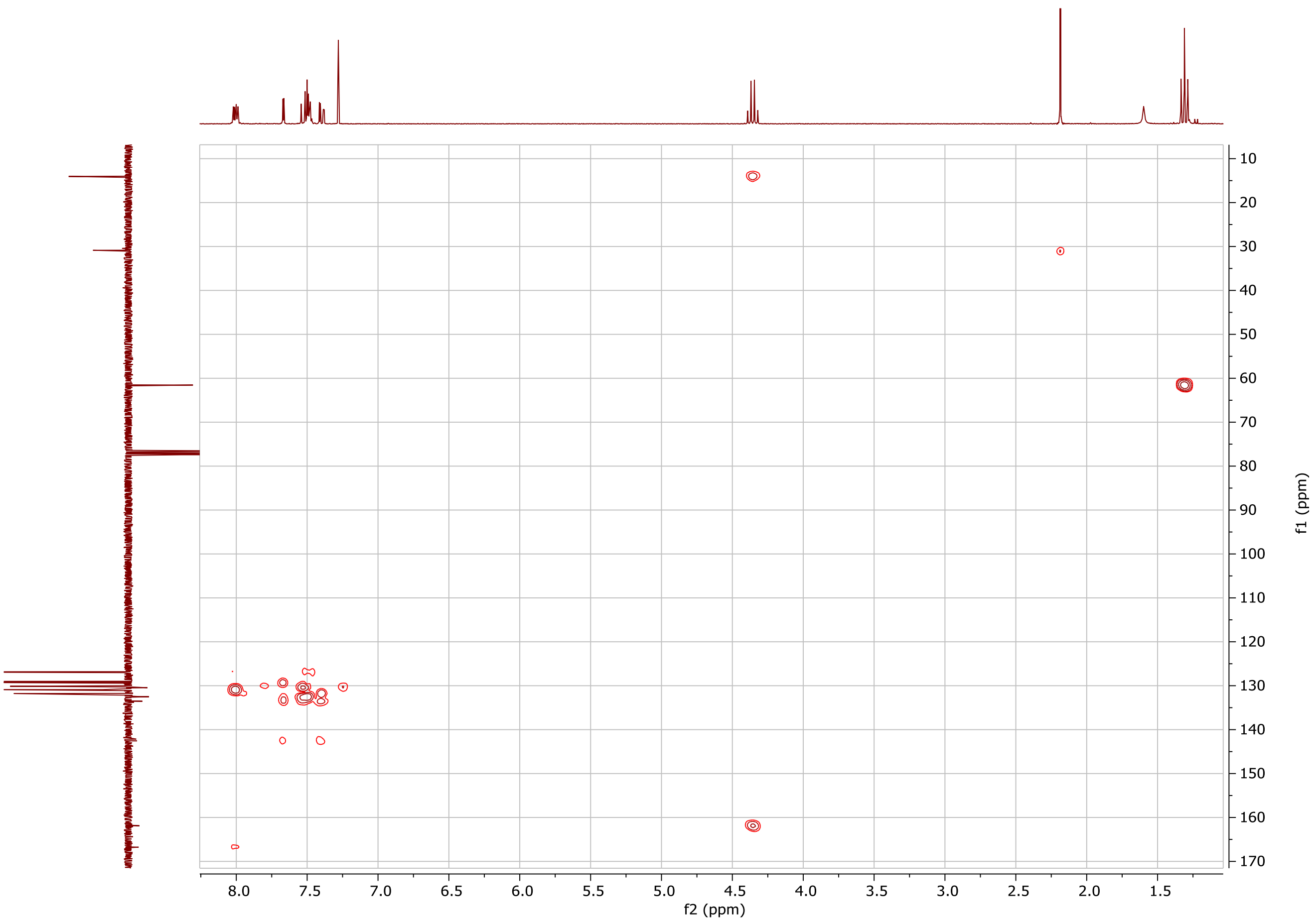

^1H-^{13}C HMBC correlation spectrum of **7n**

Methyl 5-(4-nitrophenyl)-2-phenyl-4,5-thiazole-4-carboxylate 7g

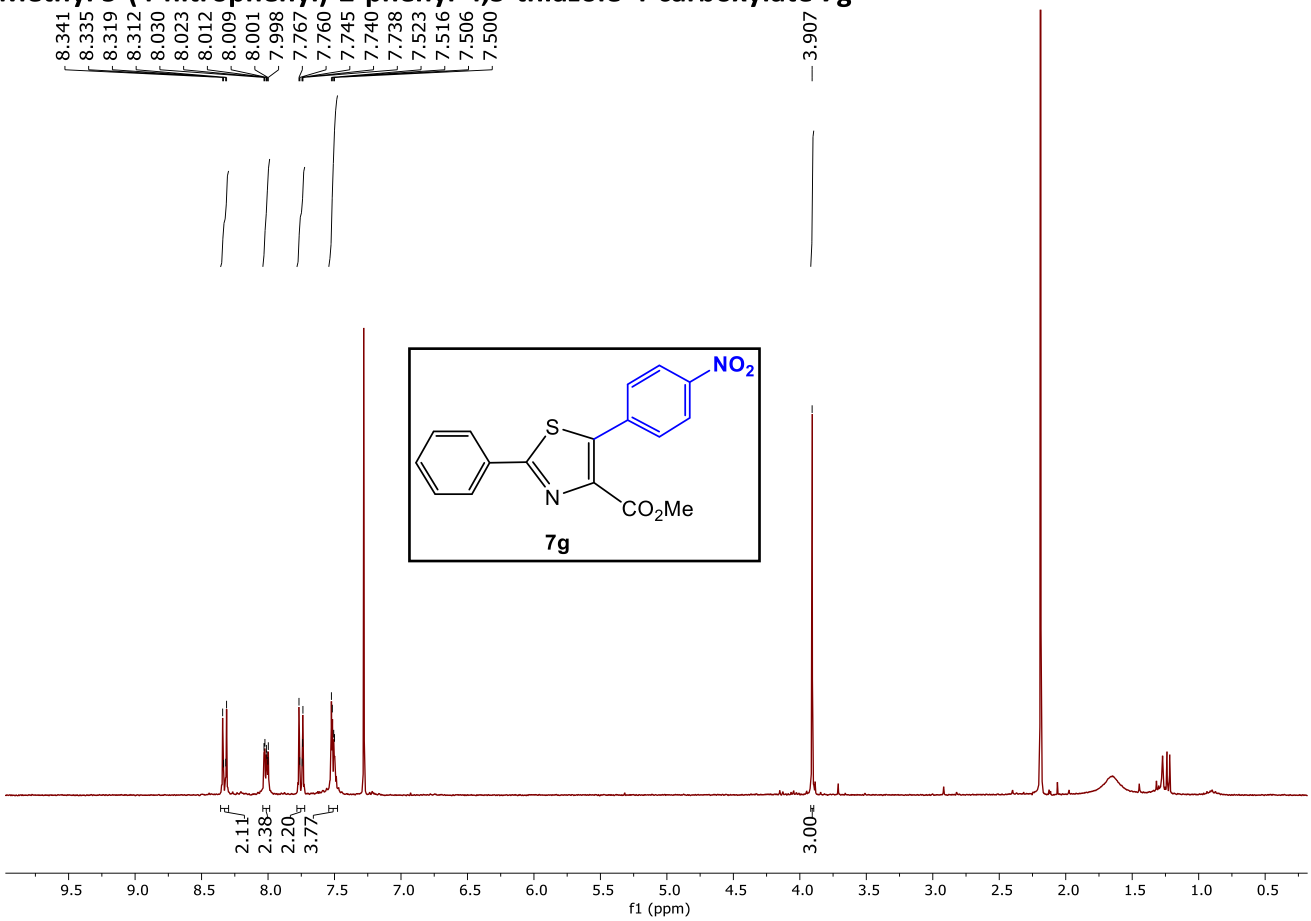

^1H NMR spectrum ($CDCl_3$, 300.13 MHz) of **7g**

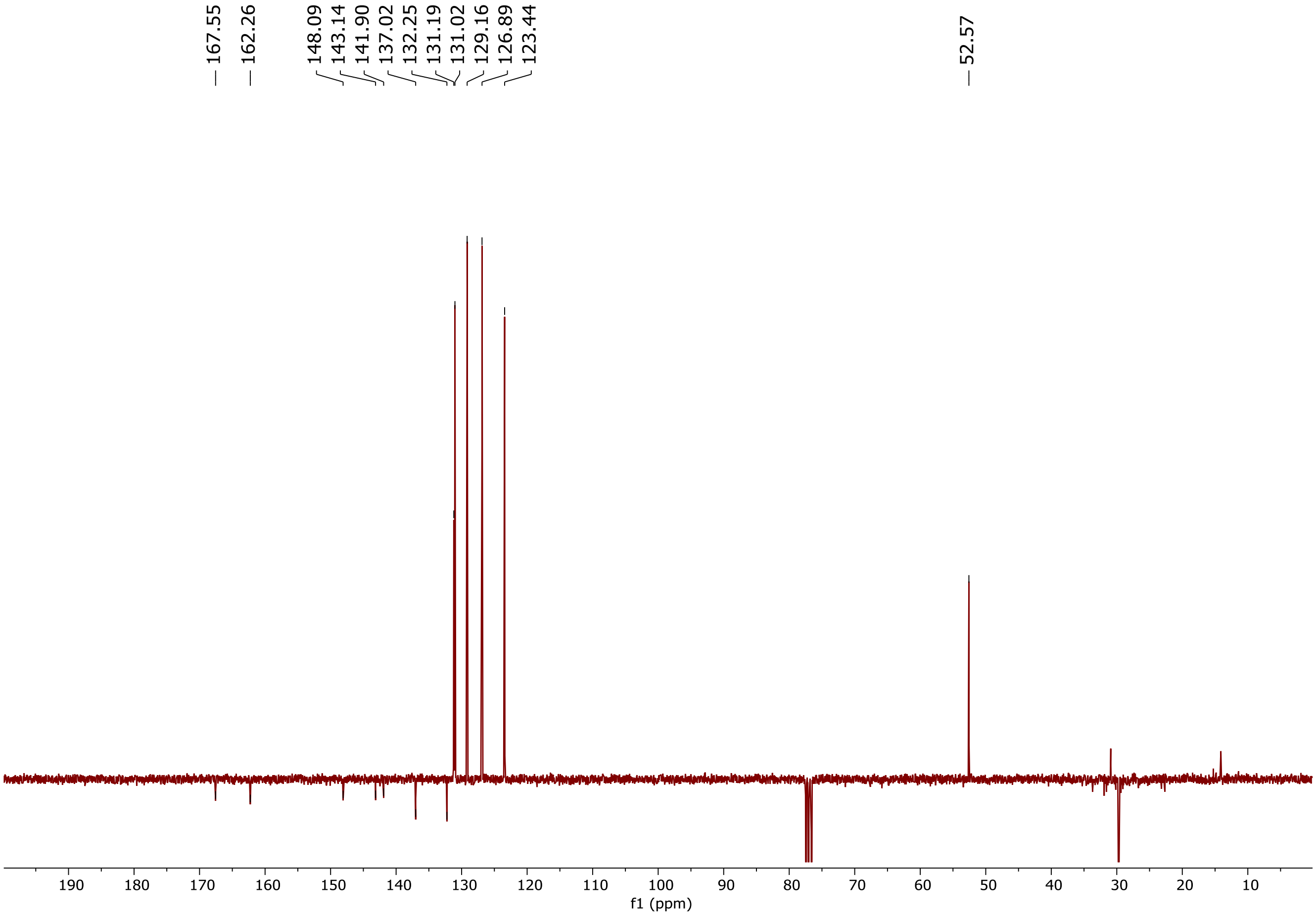

$^{13}C\{^1H\}$ NMR spectrum ($CDCl_3$, 75.47 MHz) of **7g**

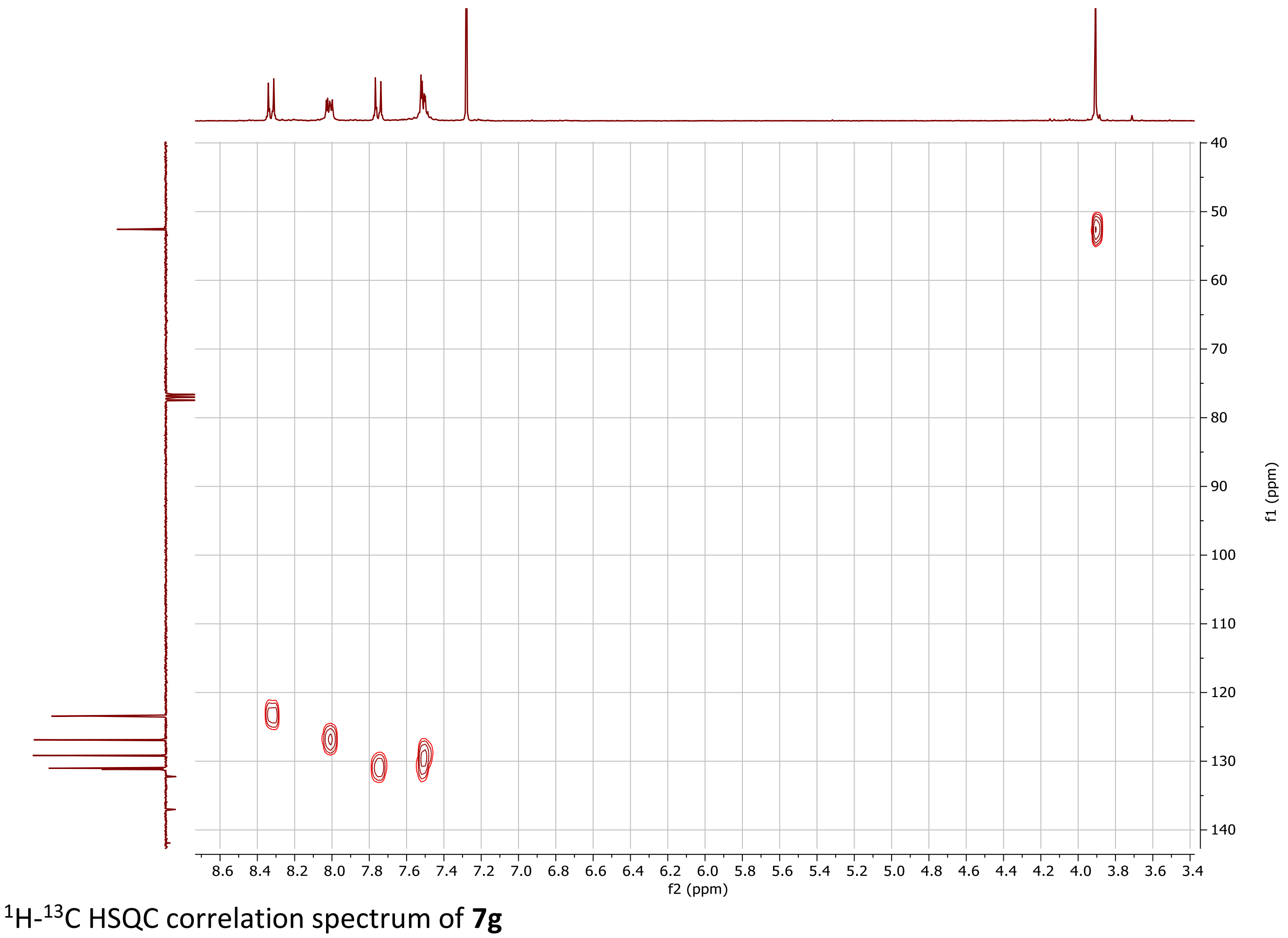

^1H-^{13}C HSQC correlation spectrum of **7g**

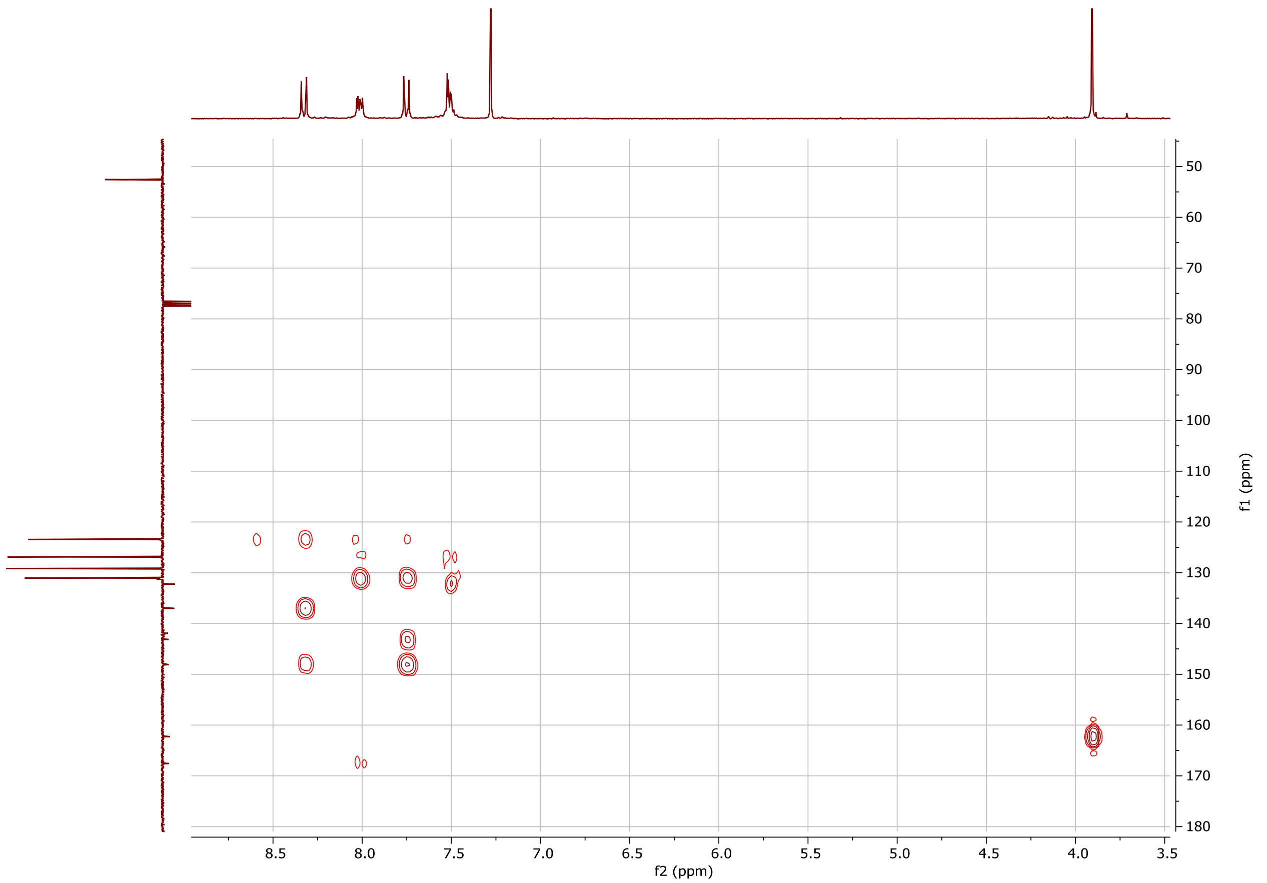

^1H-^{13}C HMBC correlation spectrum of **7g**

5.- NMR spectra of dihydrothiazole derivatives 5 (ring opening without base in presence of BF_3)

(*cis/trans*)-methyl 2,5-diphenyl-4,5-dihydrothiazole-4-carboxylate 5a

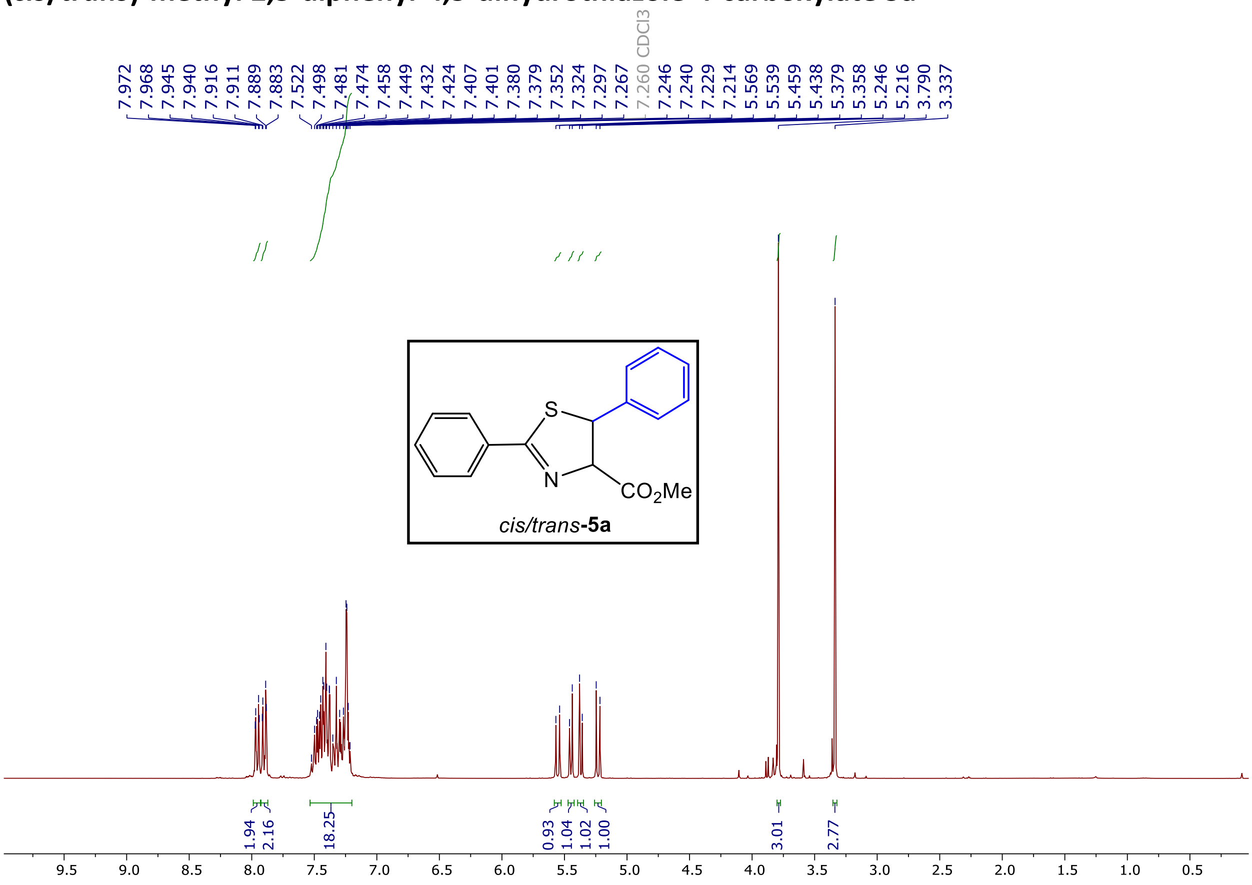

^1H NMR spectrum ($CDCl_3$, 300.13 MHz) of **5a**

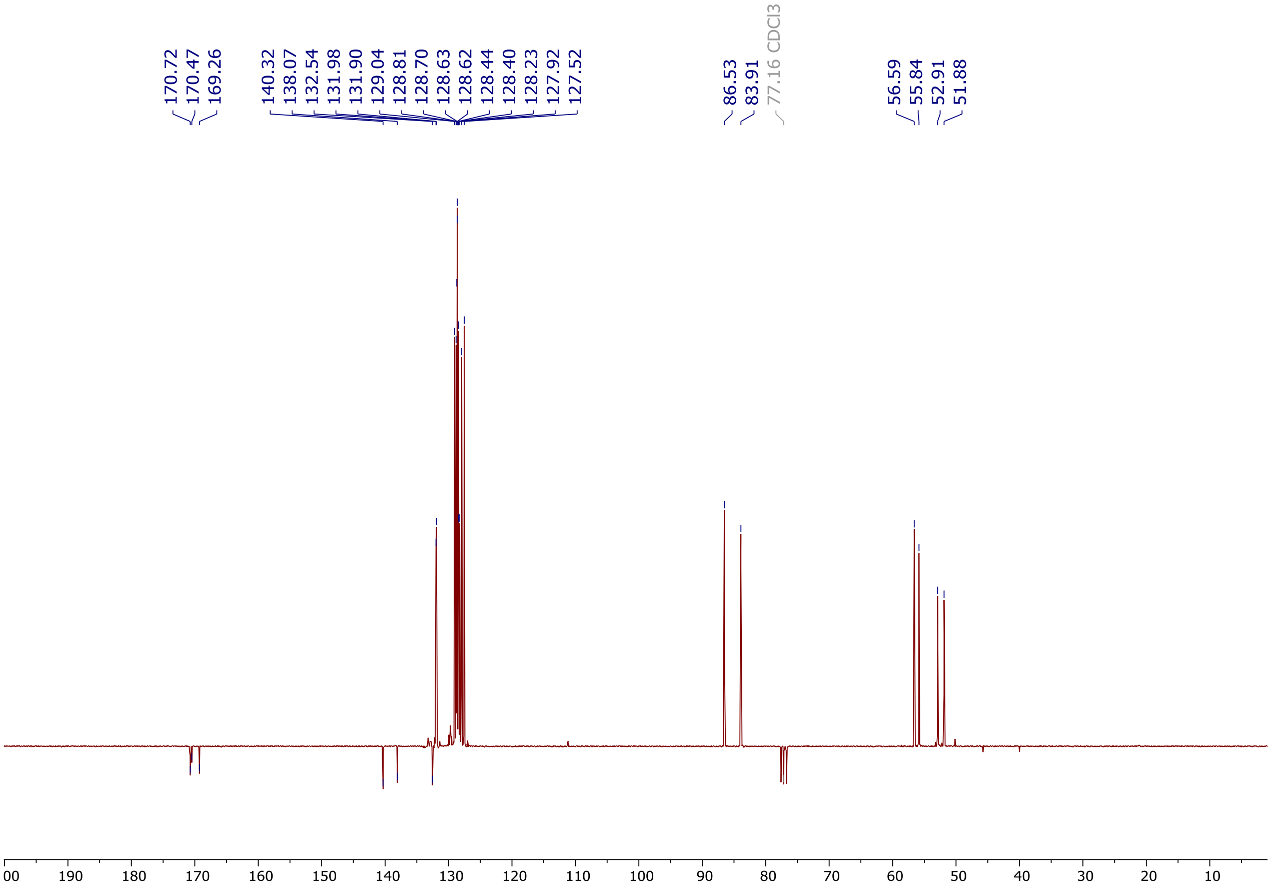

$^{13}C\{^{1}H\}$ NMR (APT) spectrum ($CDCl_3$, 75.5 MHz) of **5a**

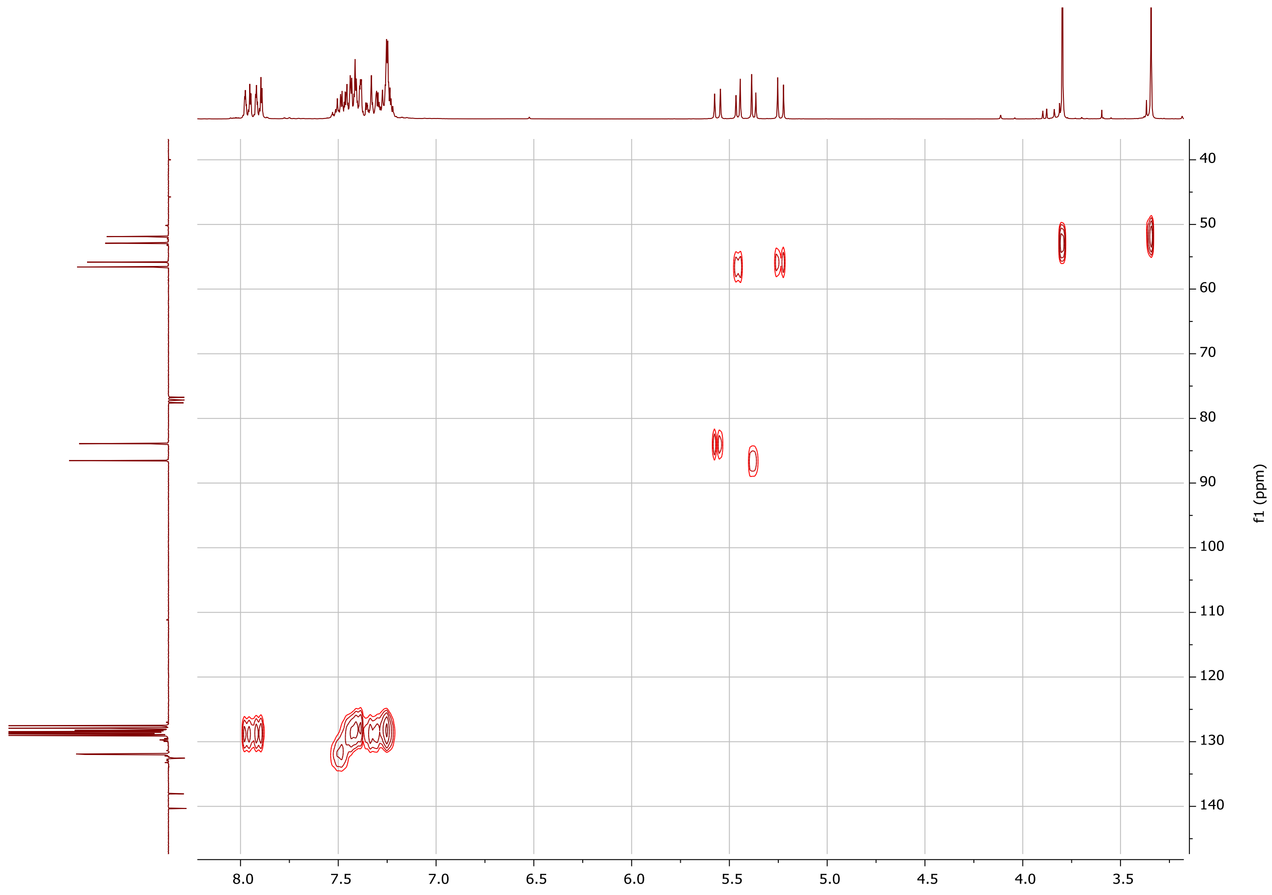

^{1}H-^{13}C HSQC correlation spectrum of **5a**

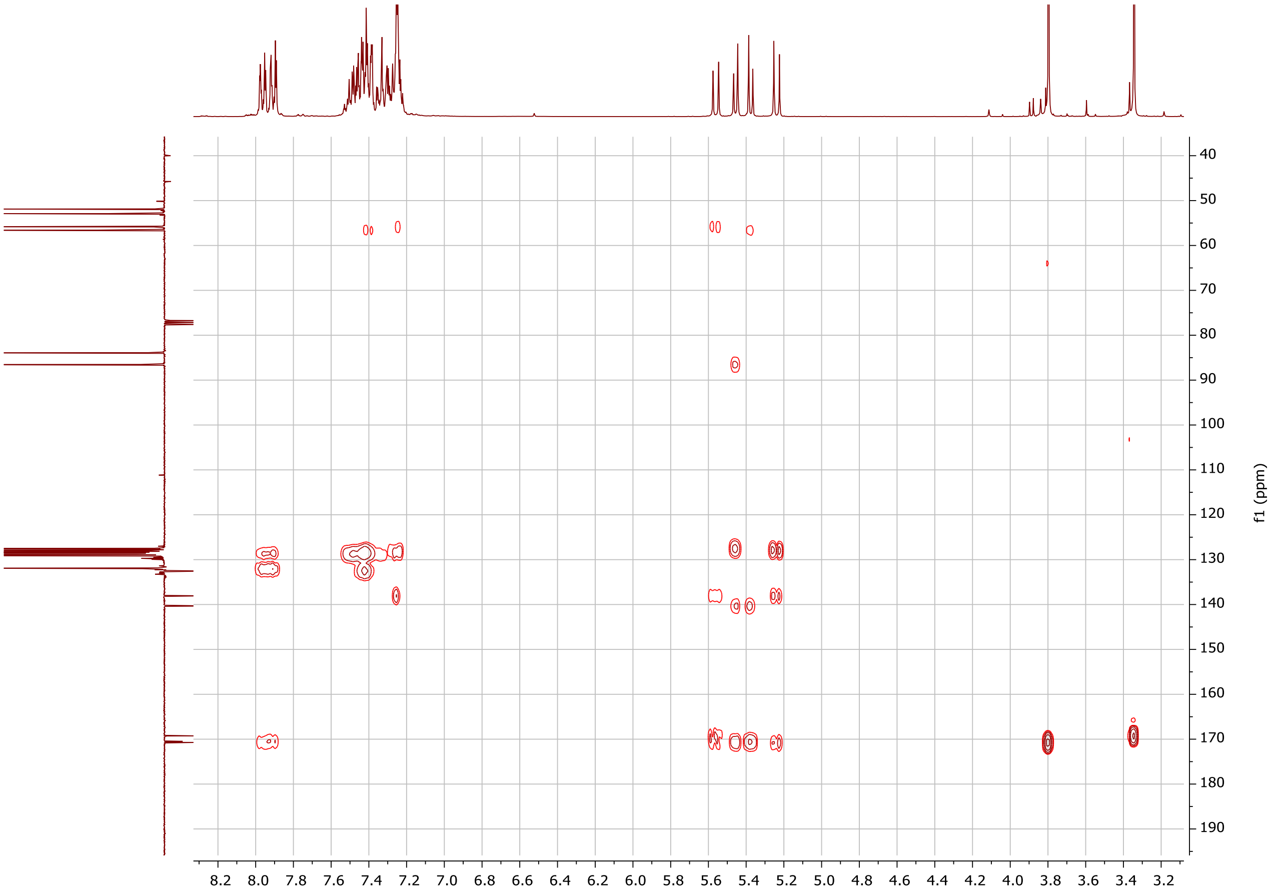

^{1}H-^{13}C HMBC correlation spectrum of **5a**

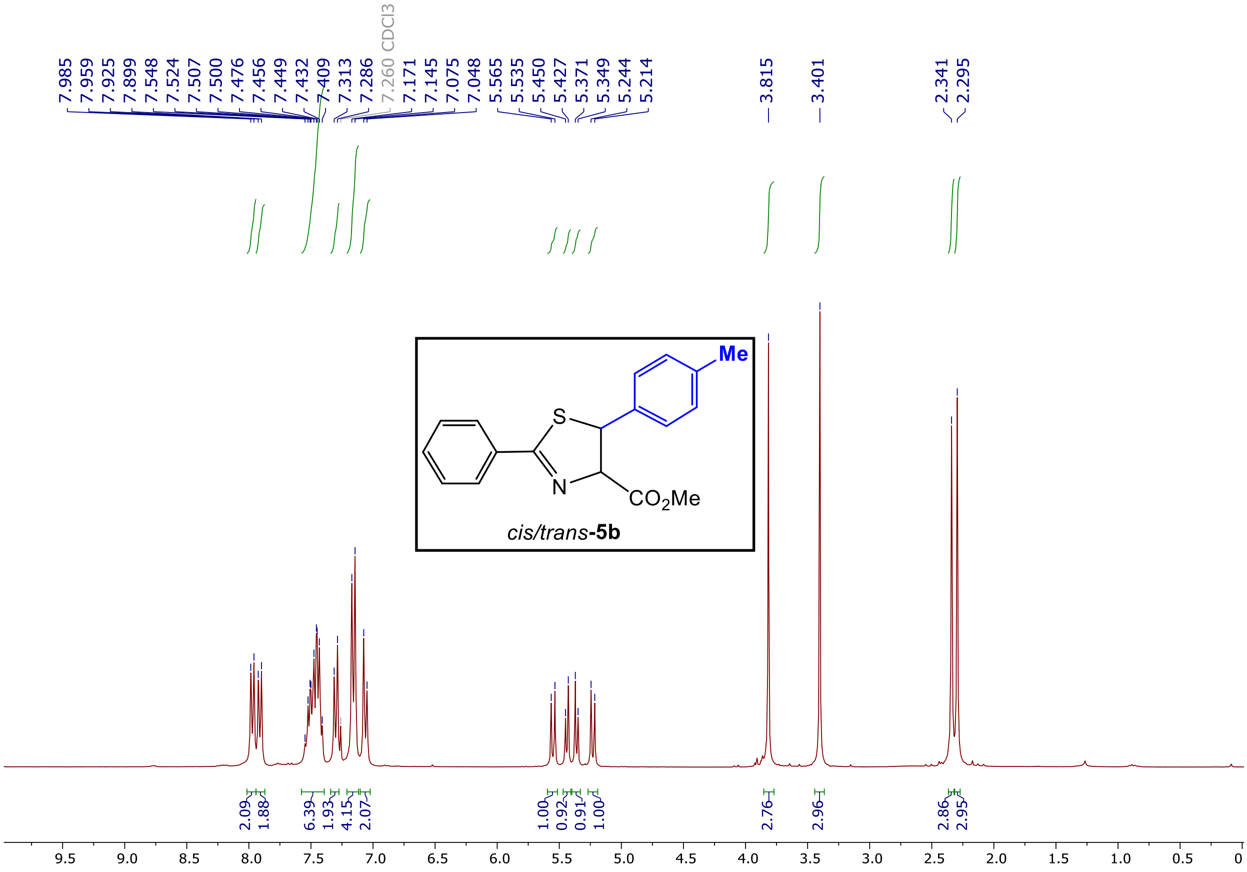

^{1}H NMR spectrum ($CDCl_3$, 300.13 MHz) of **5b**

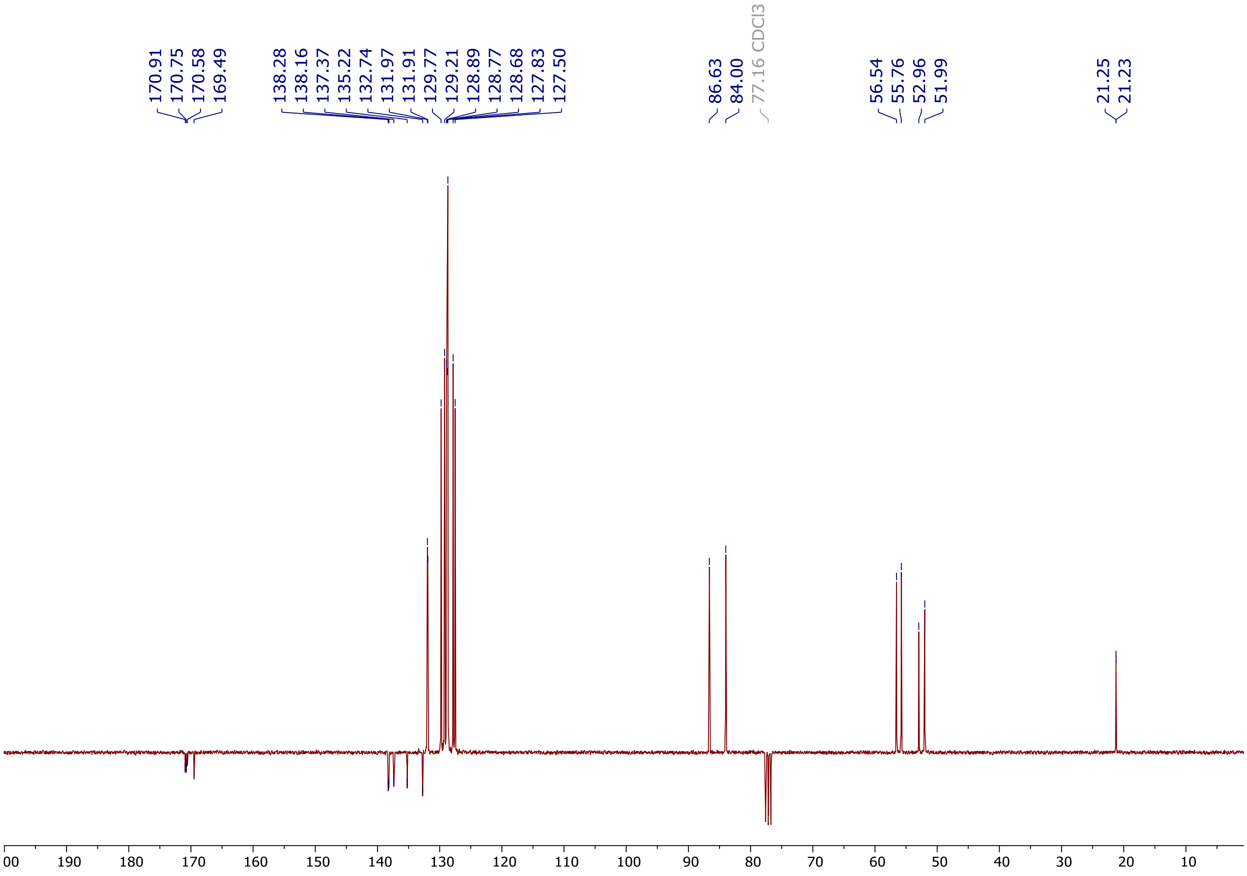

$^{13}C\{^{1}H\}$ NMR (APT) spectrum ($CDCl_3$, 75.5 MHz) of **5b**

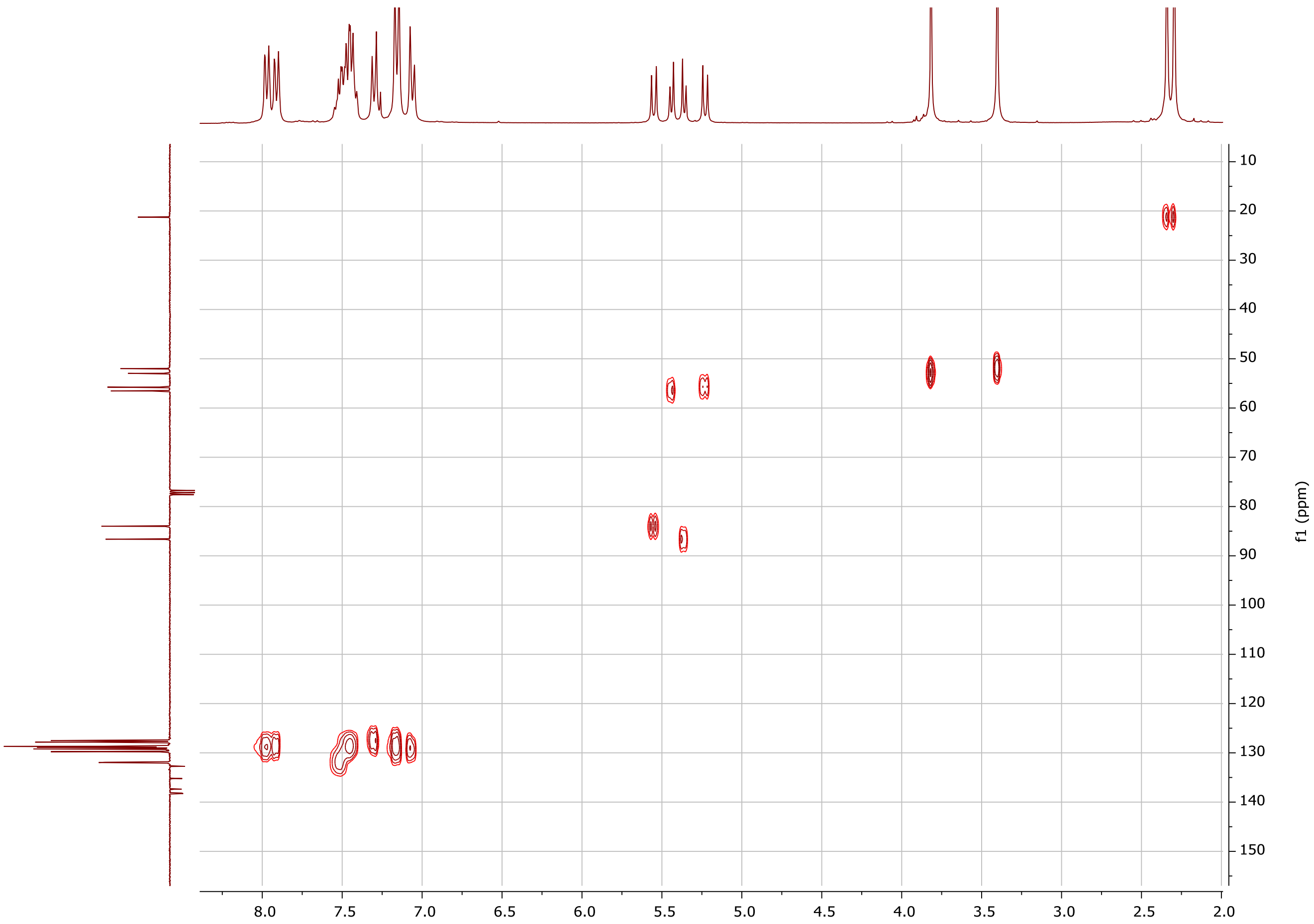

^{1}H-^{13}C HSQC correlation spectrum of **5b**

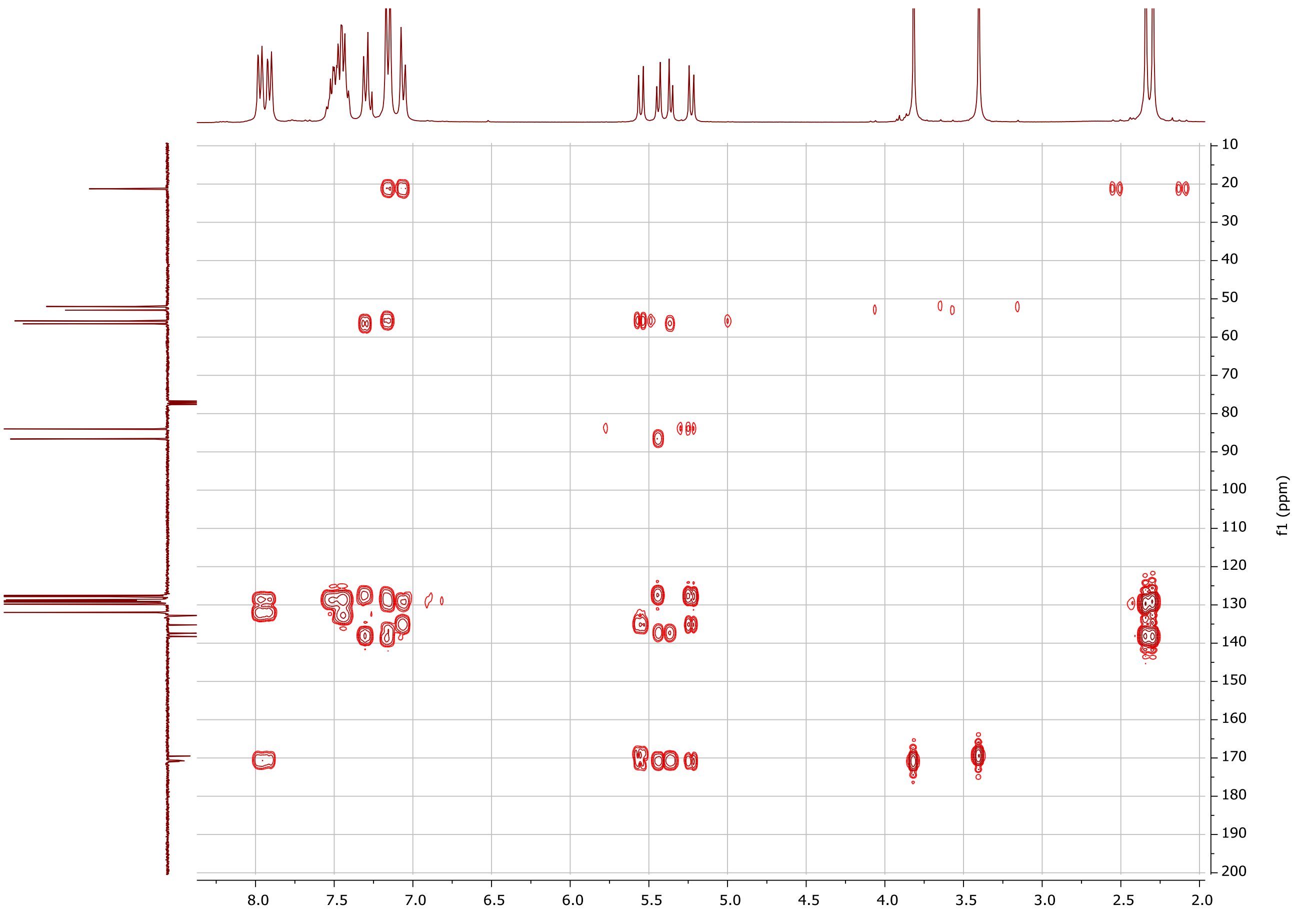

^{1}H-^{13}C HMBC correlation spectrum of **5b**

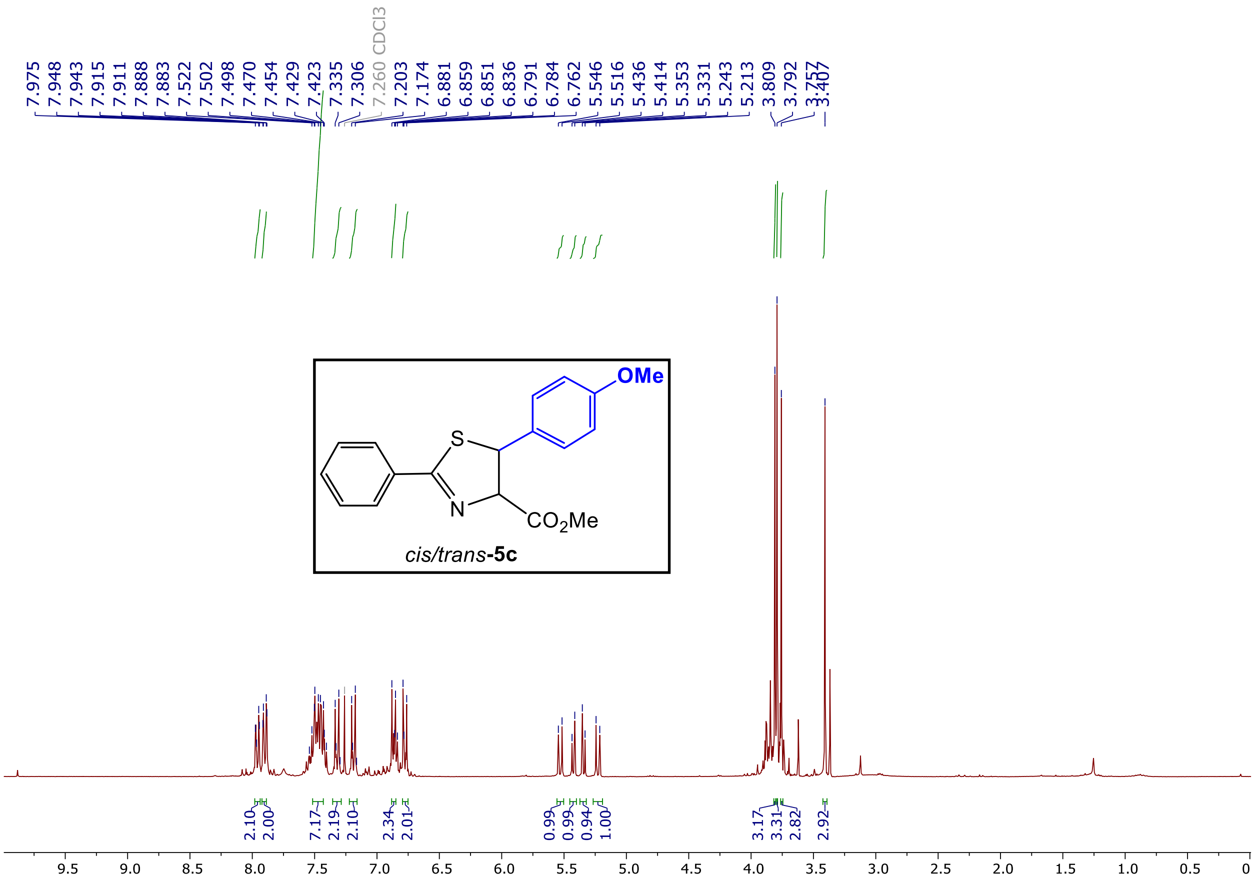

^{1}H NMR spectrum ($CDCl_3$, 300.13 MHz) of **5c**

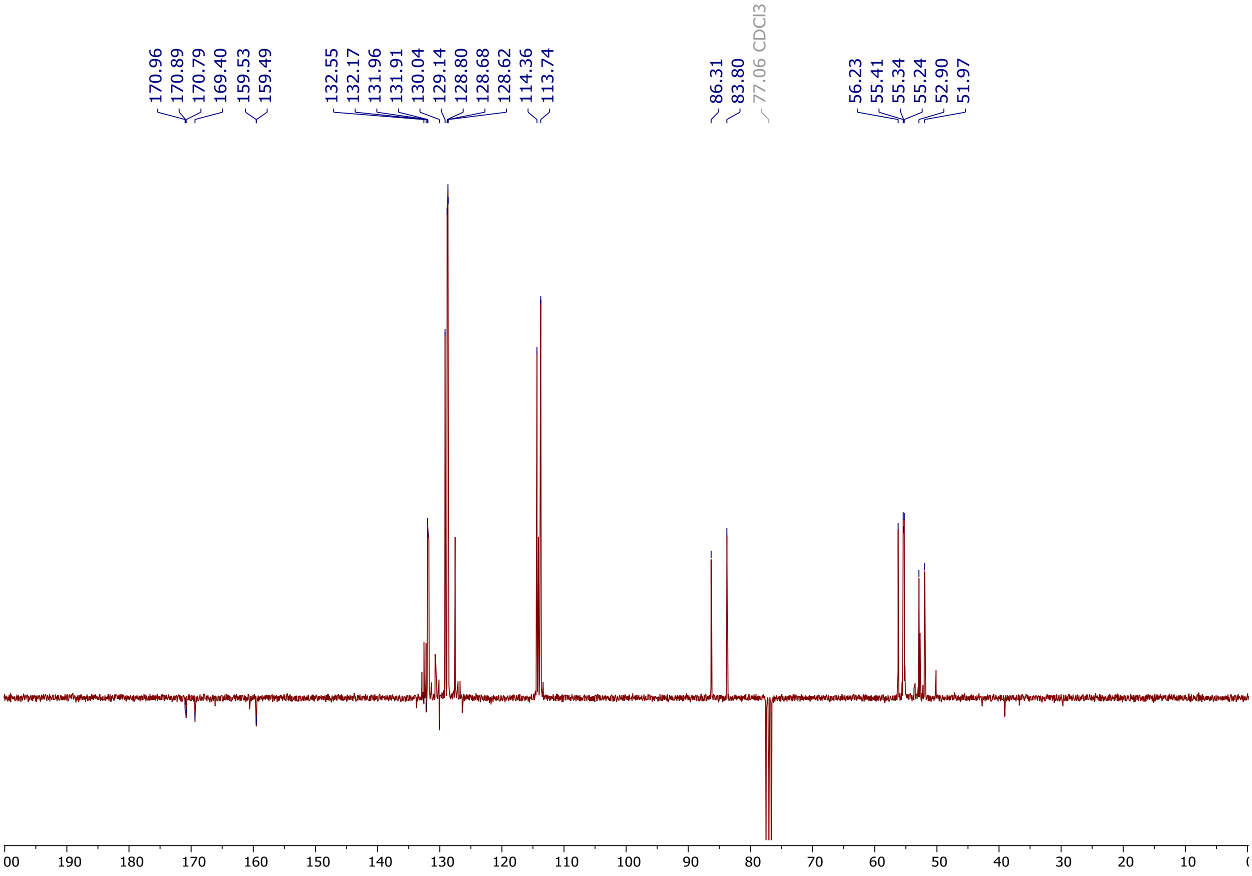

$^{13}C\{^{1}H\}$ NMR (APT) spectrum ($CDCl_3$, 75.5 MHz) of **5c**

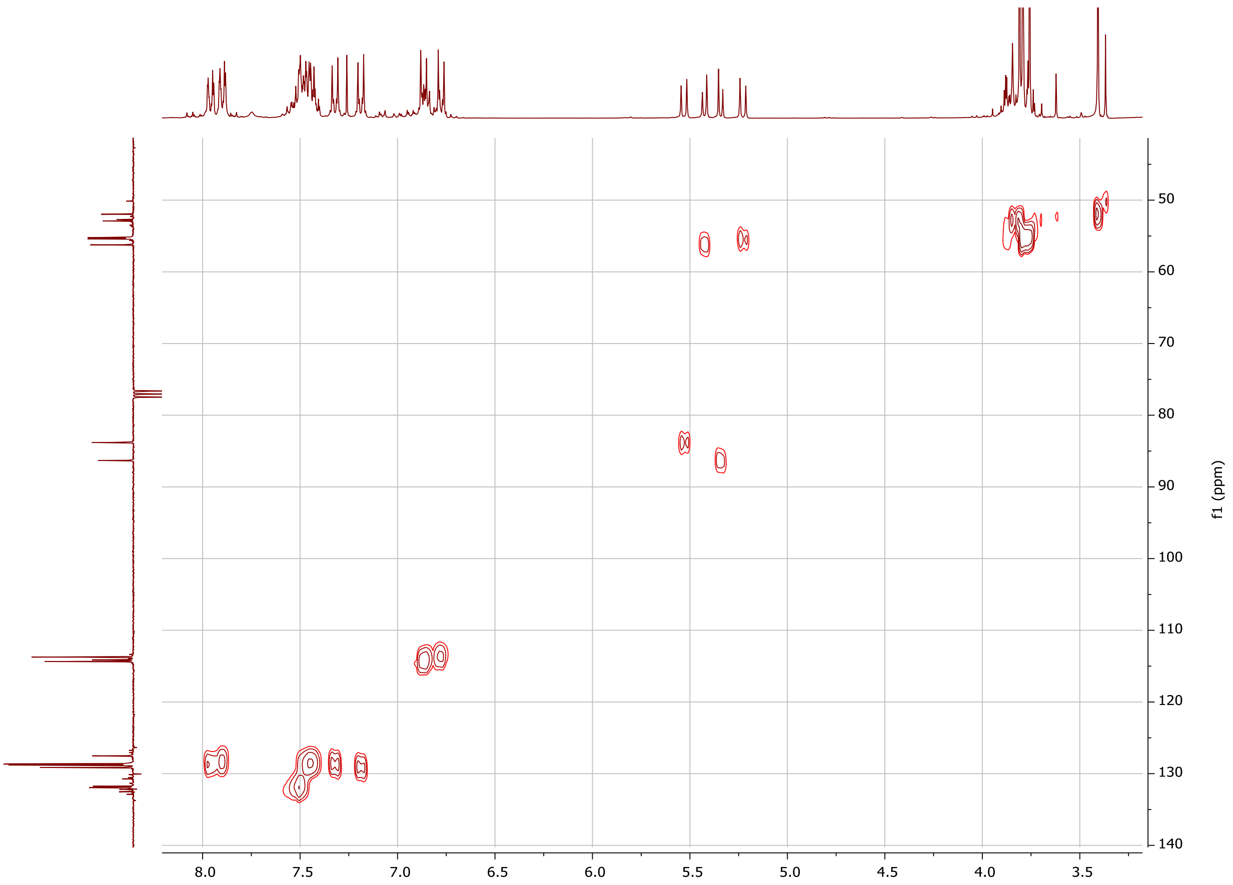

^{1}H-^{13}C HSQC correlation spectrum of **5c**

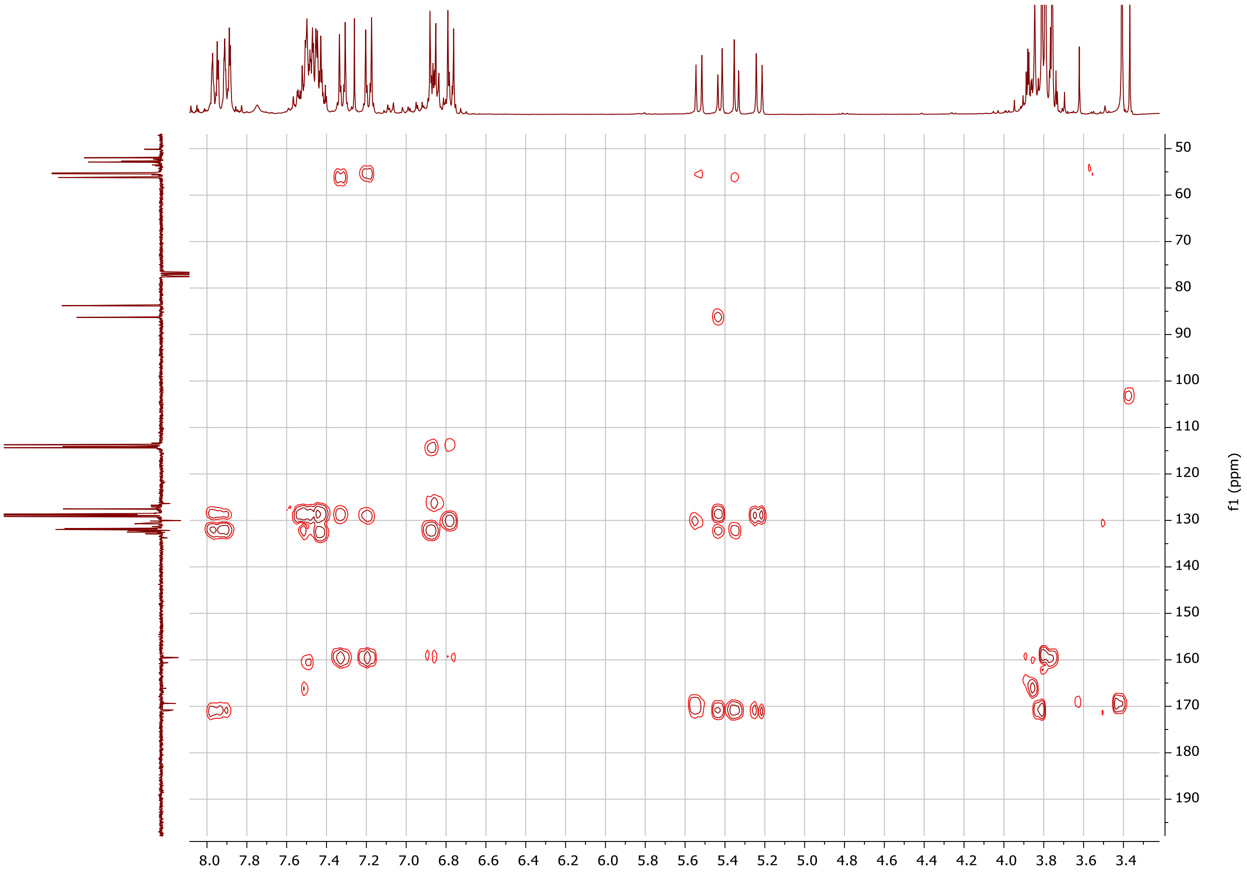

^1H-^{13}C HMBC correlation spectrum of **5c**

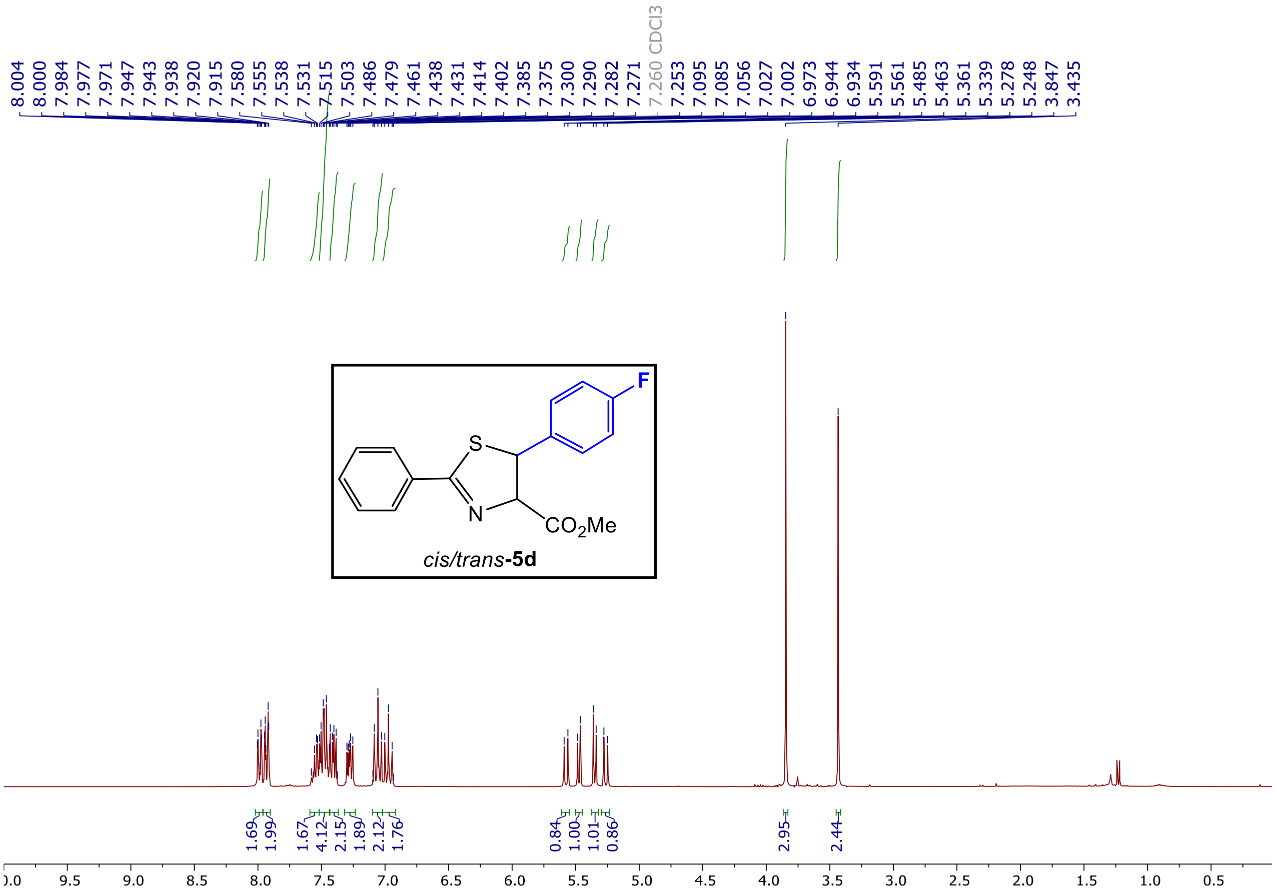

^1H NMR spectrum ($CDCl_3$, 300.13 MHz) of **5d**

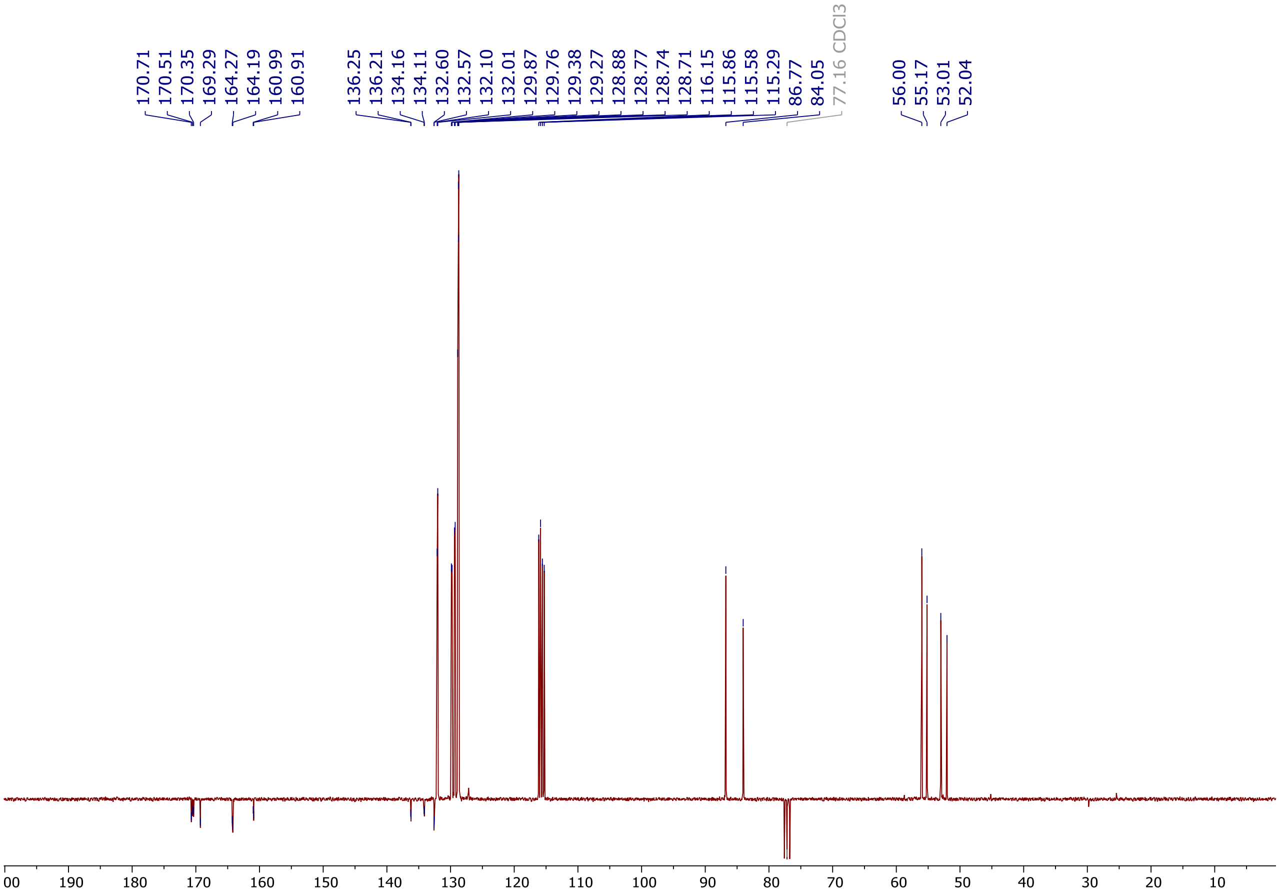

$^{13}C\{^{1}H\}$ NMR (APT) spectrum ($CDCl_3$, 75.5 MHz) of **5d**

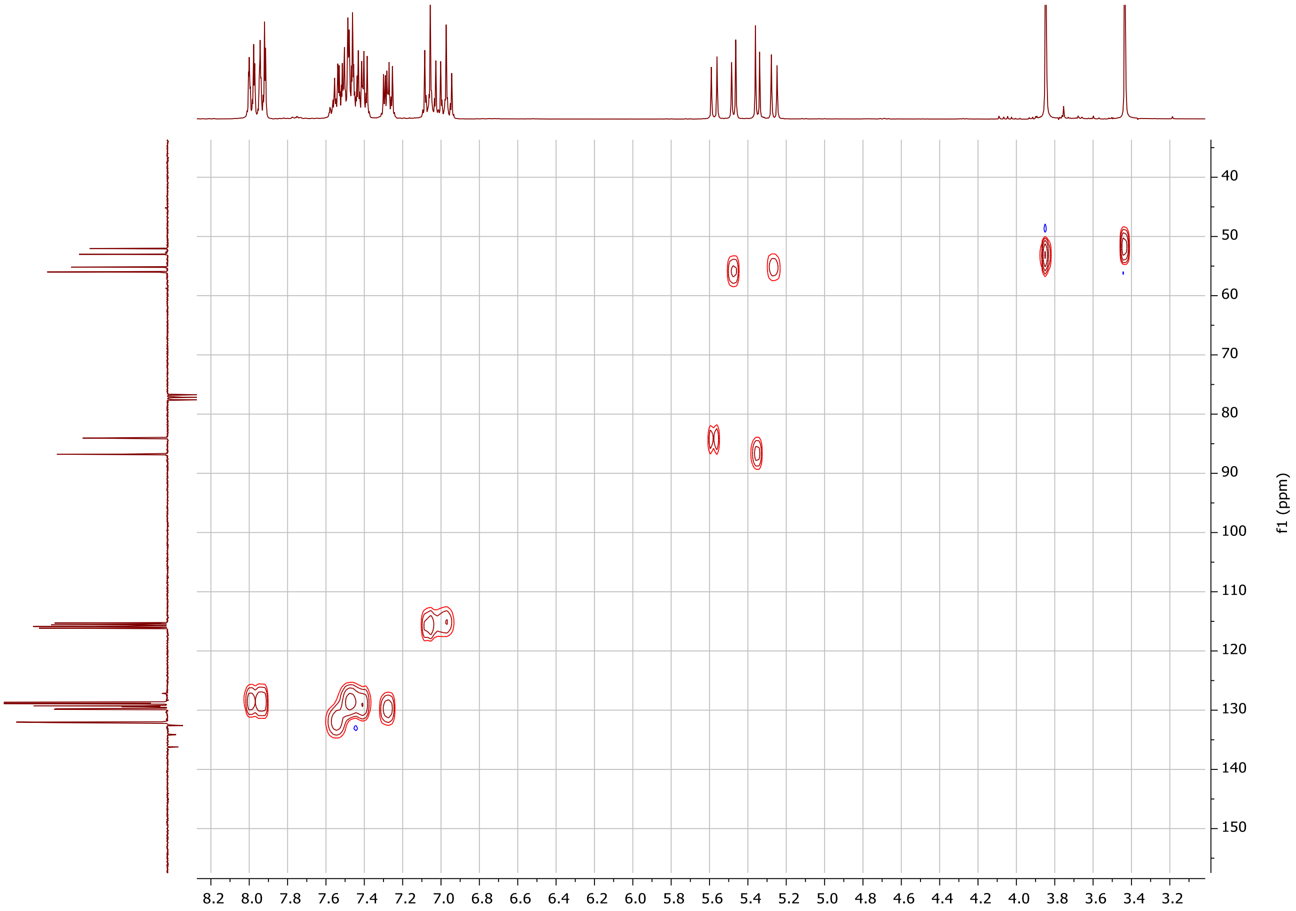

^{1}H-^{13}C HSQC correlation spectrum of **5d**

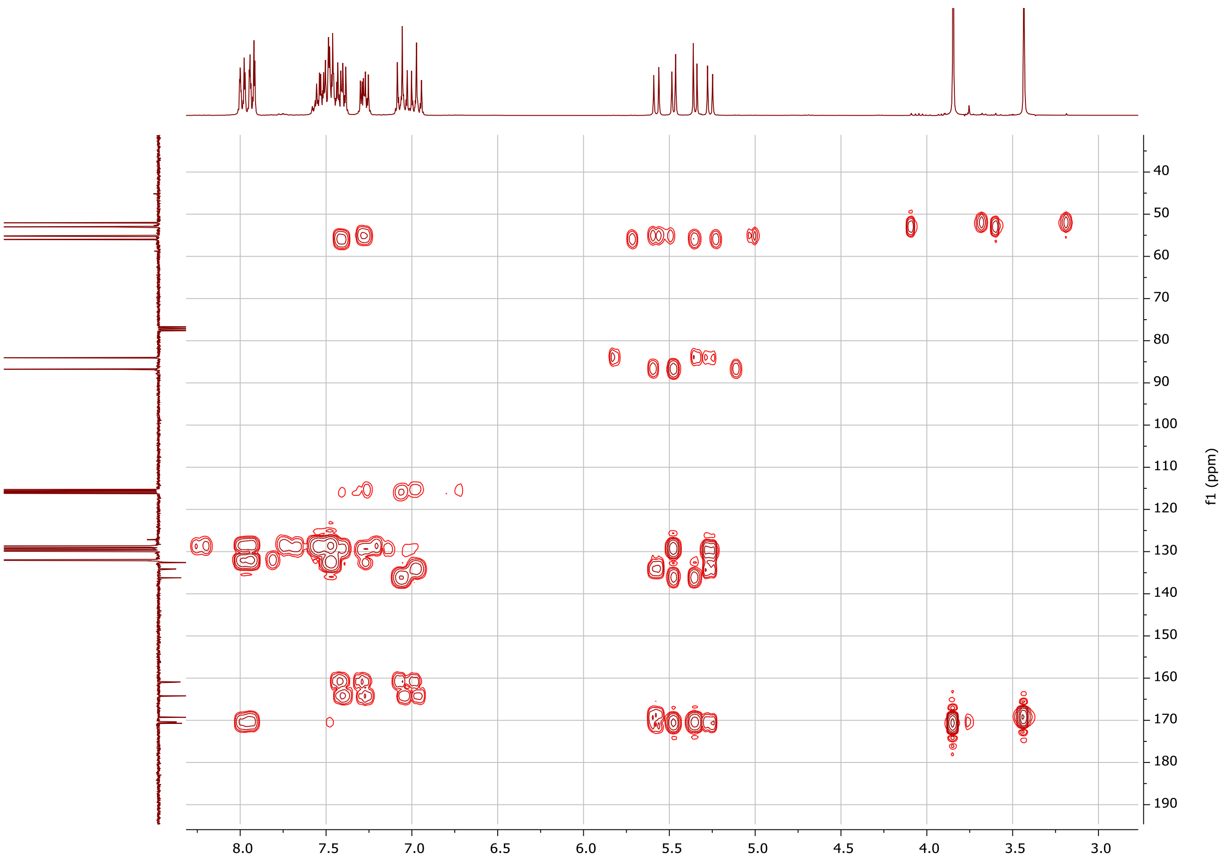

^{1}H-^{13}C HMBC correlation spectrum of **5d**

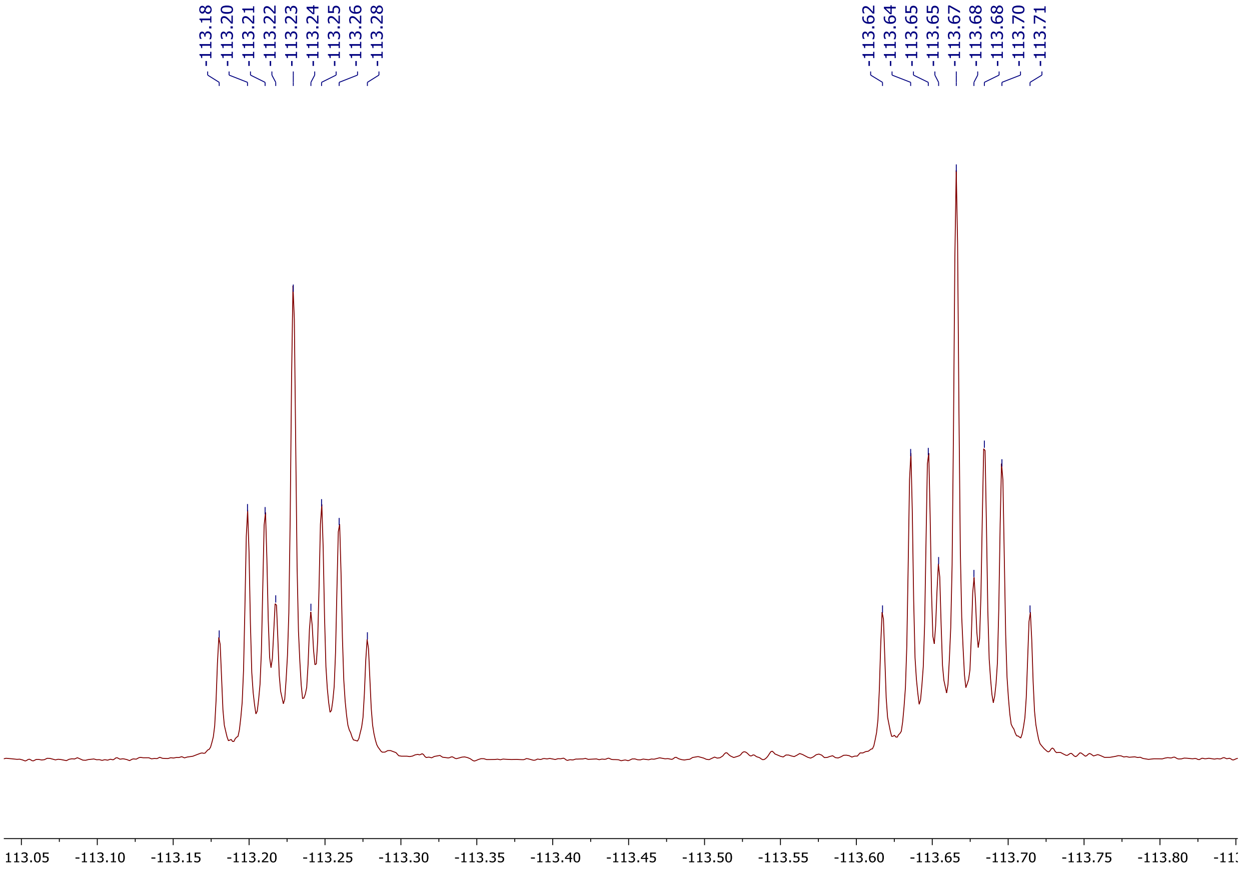

^{19}F NMR spectrum ($CDCl_3$, 282.40 MHz) of **5d**

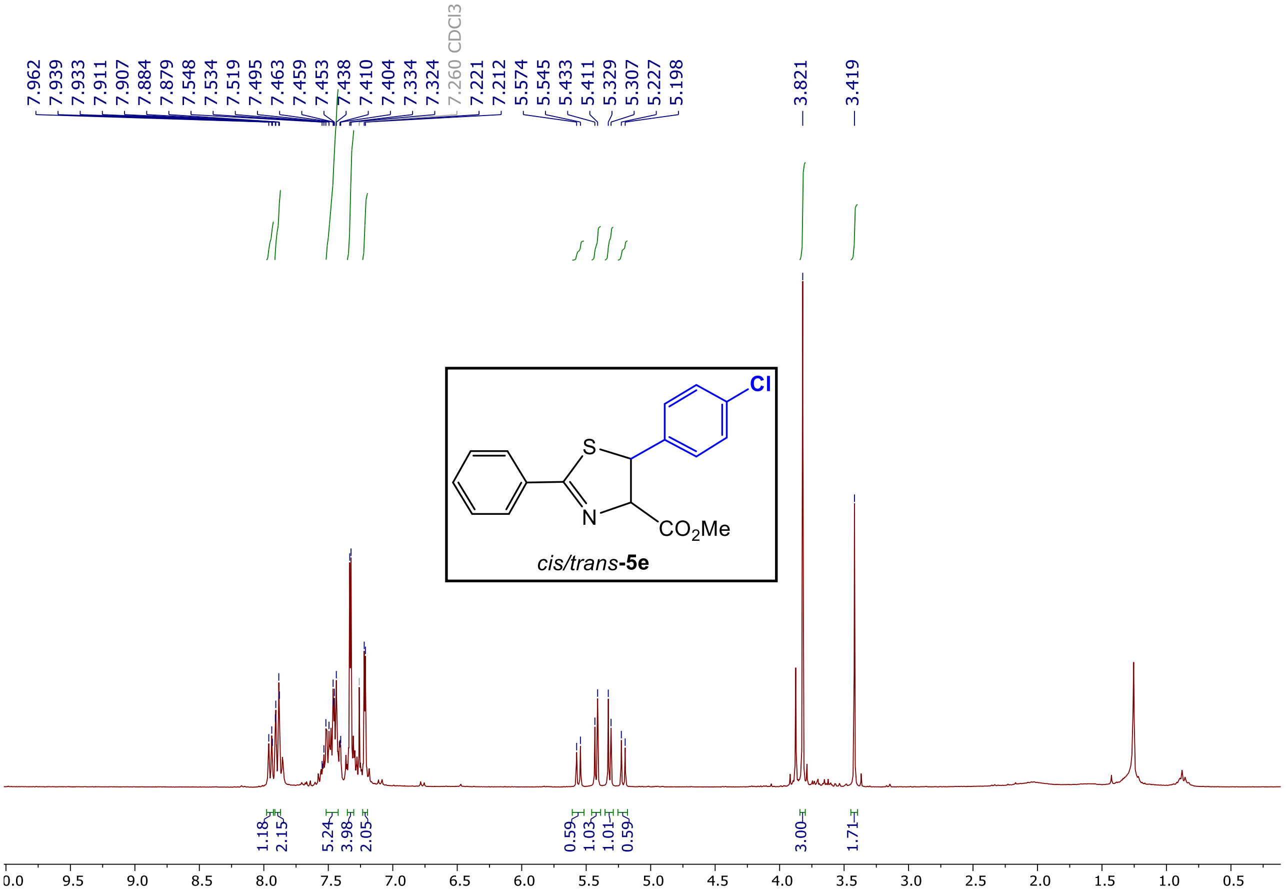

^{1}H NMR spectrum ($CDCl_3$, 300.13 MHz) of **5e**

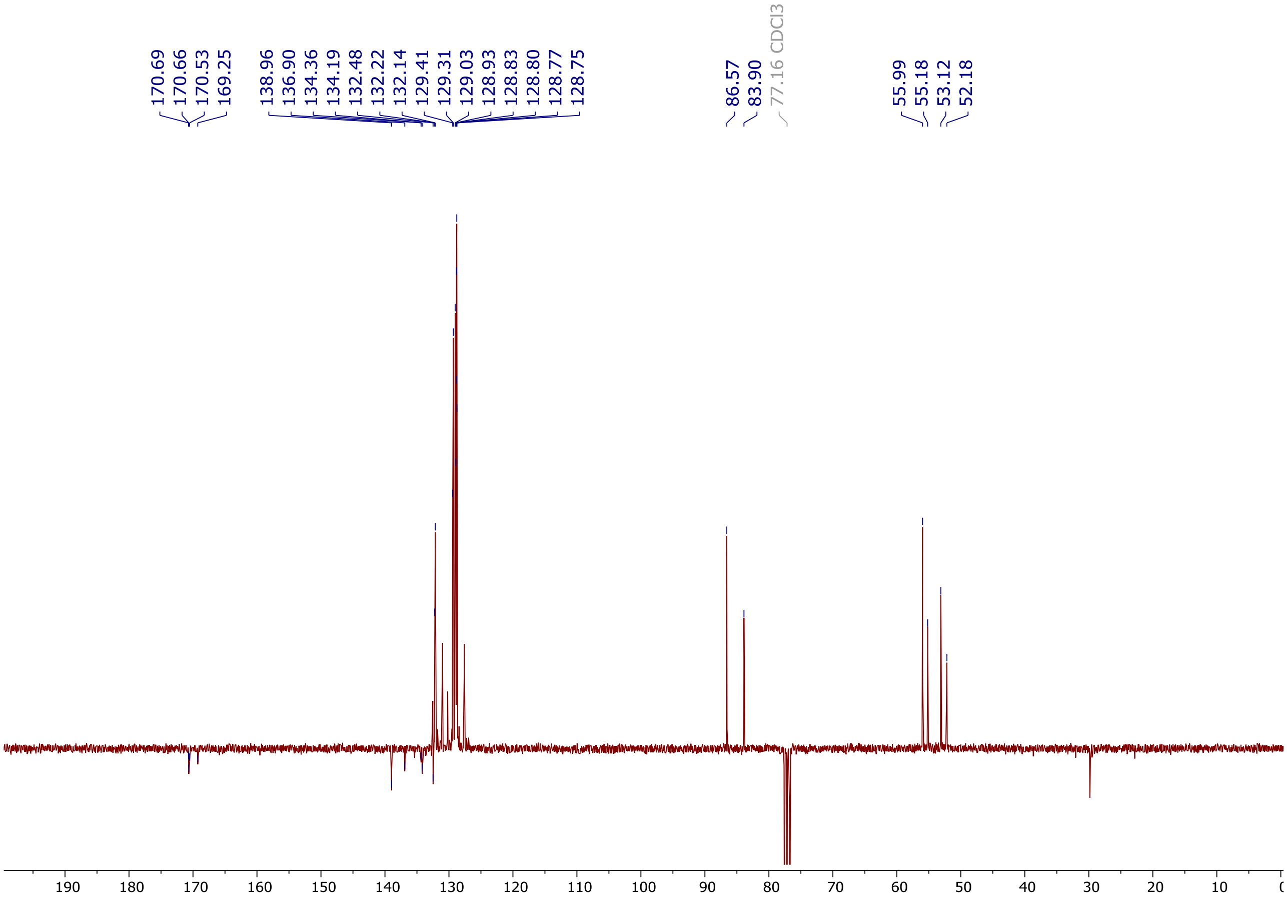

^{13}C{^{1}H} NMR (APT) spectrum ($CDCl_3$, 75.5 MHz) of **5e**

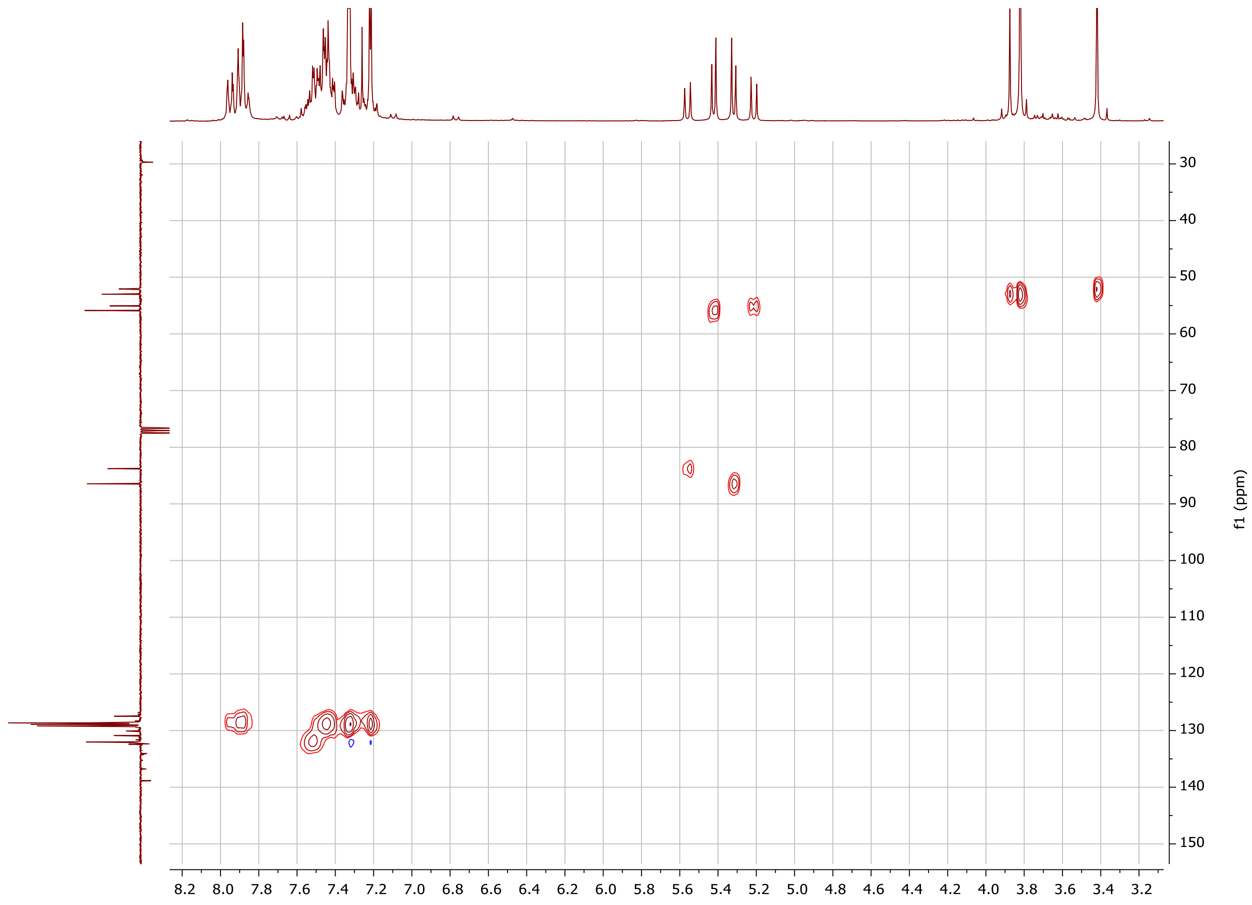

^{1}H-^{13}C HSQC correlation spectrum of **5e**

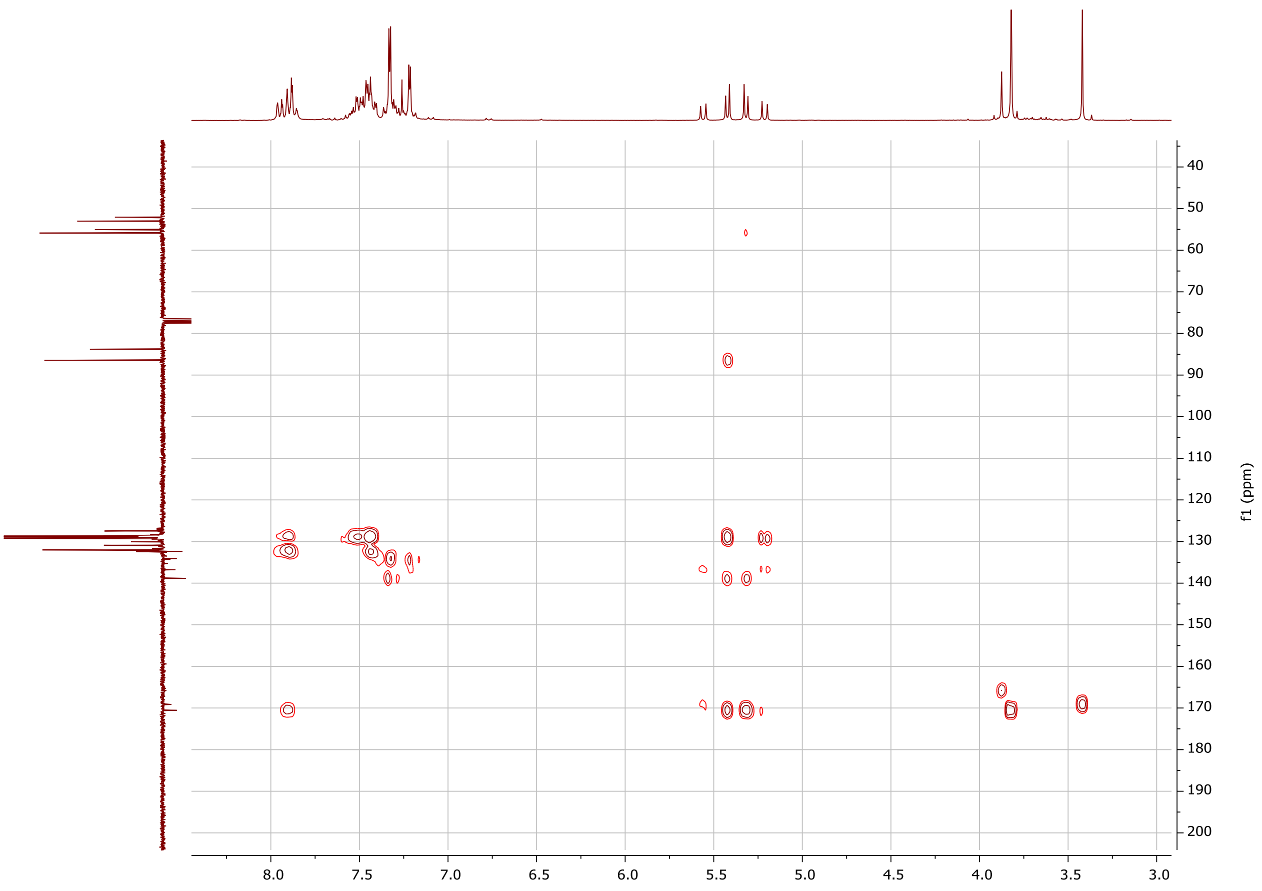

^{1}H-^{13}C HMBC correlation spectrum of **5e**

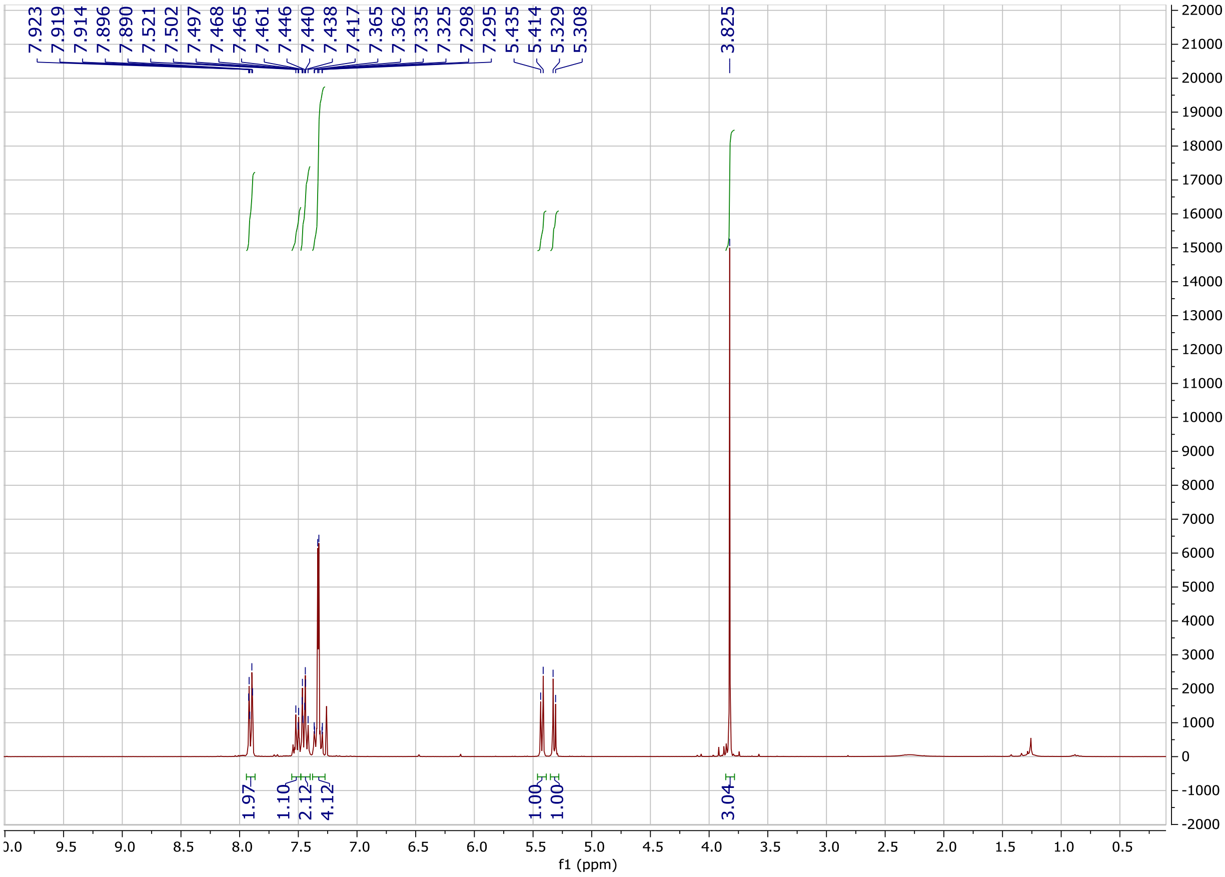

^{1}H NMR spectrum ($CDCl_3$, 300.13 MHz) of *trans*-**5e**

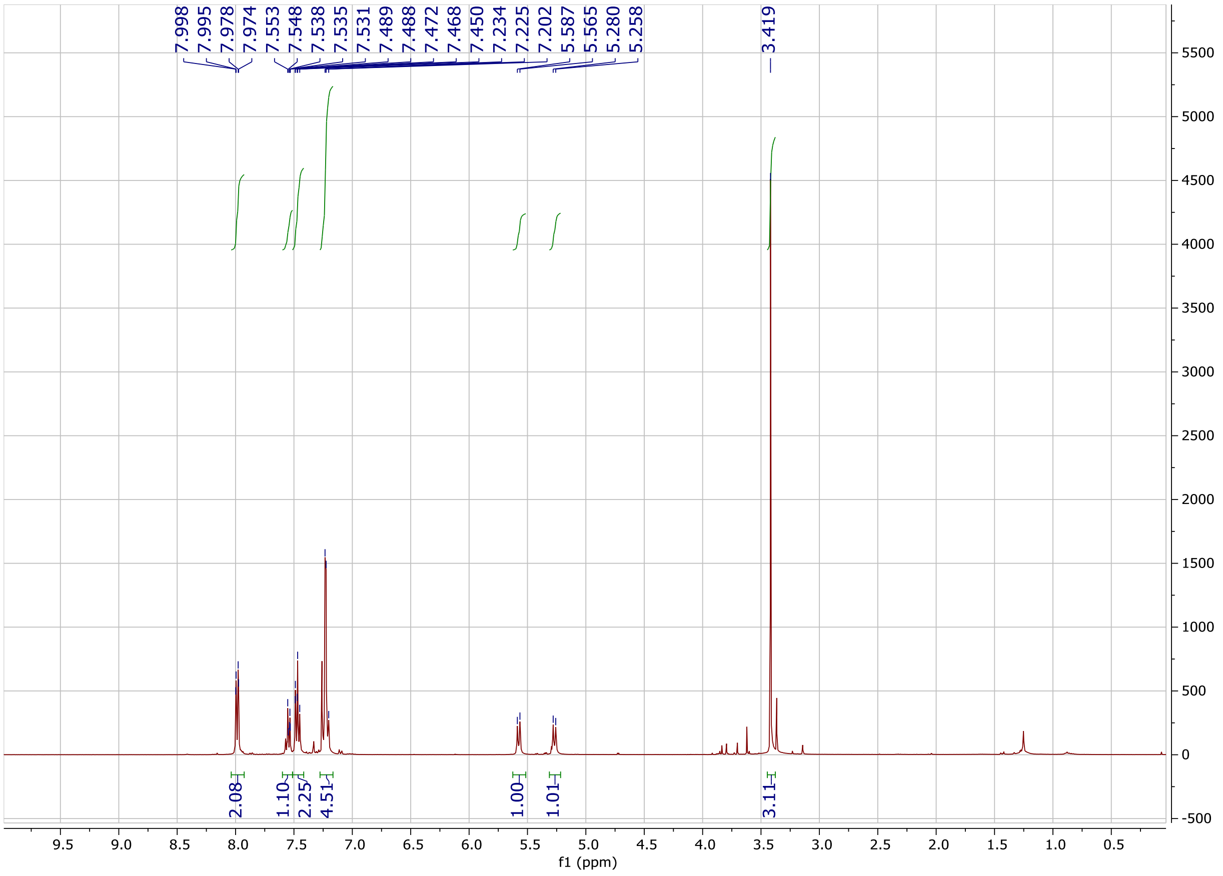

^{1}H NMR spectrum ($CDCl_3$, 400.13 MHz) of *cis*-**5e**

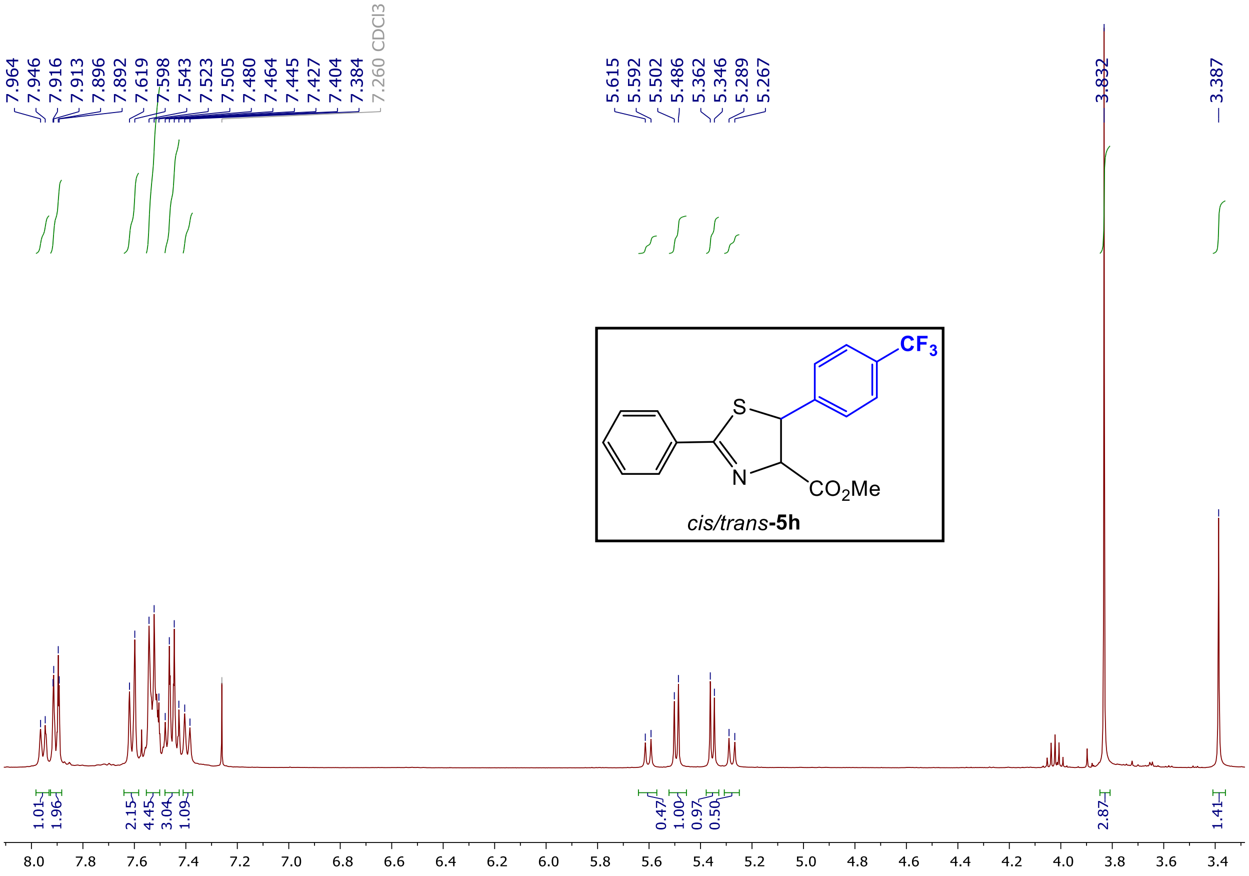

^{1}H NMR spectrum ($CDCl_3$, 300.13 MHz) of **5h**

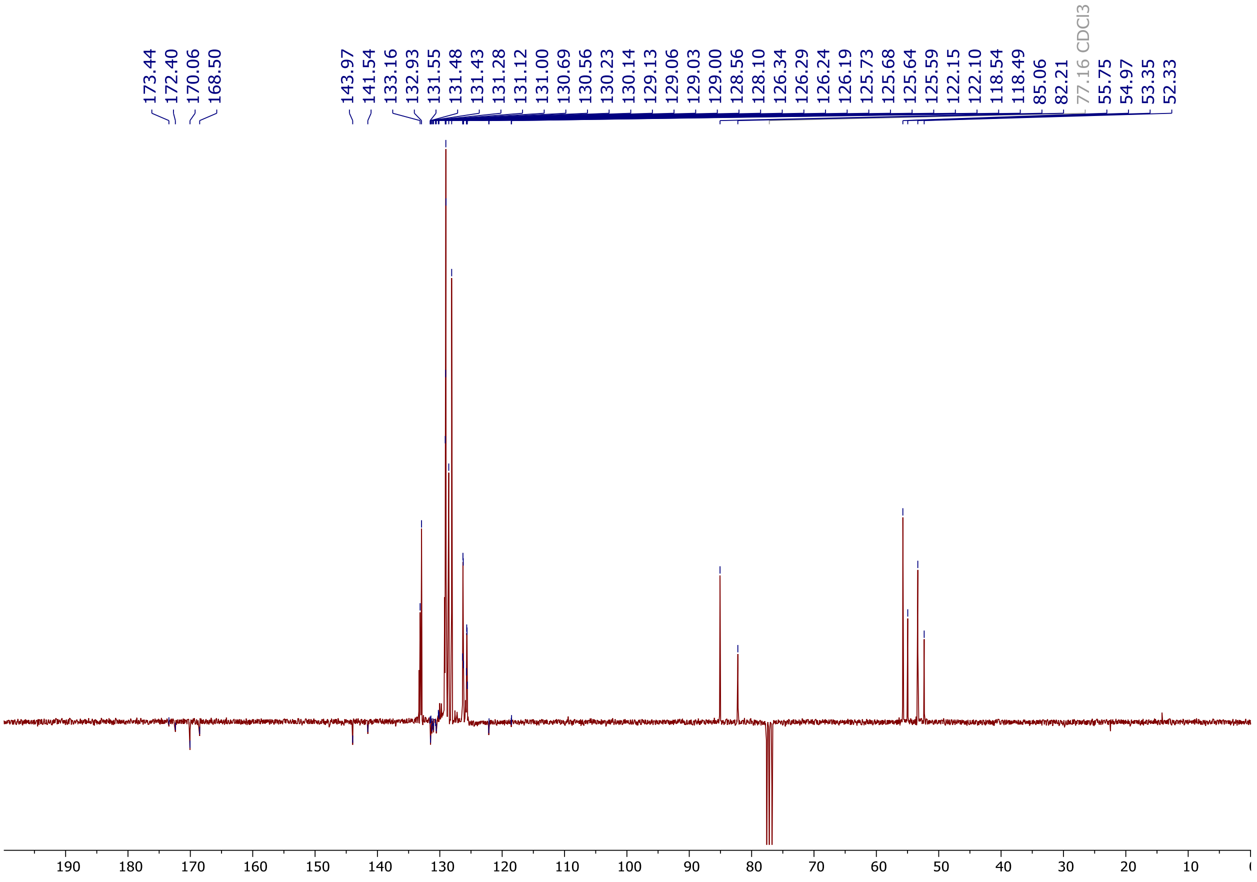

$^{13}C\{^{1}H\}$ NMR (APT) spectrum ($CDCl_3$, 75.5 MHz) of **5h**

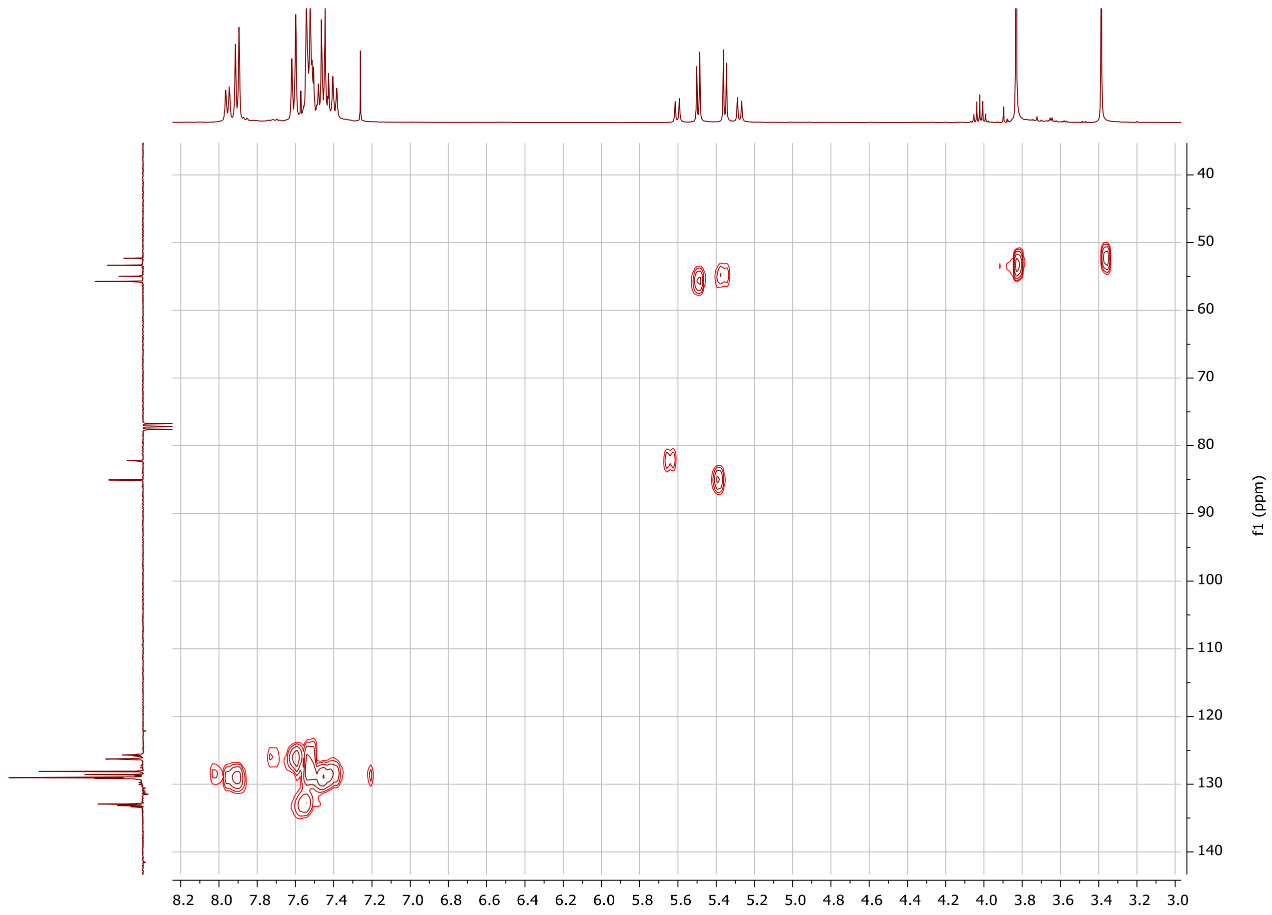

^{1}H-^{13}C HSQC correlation spectrum of **5h**

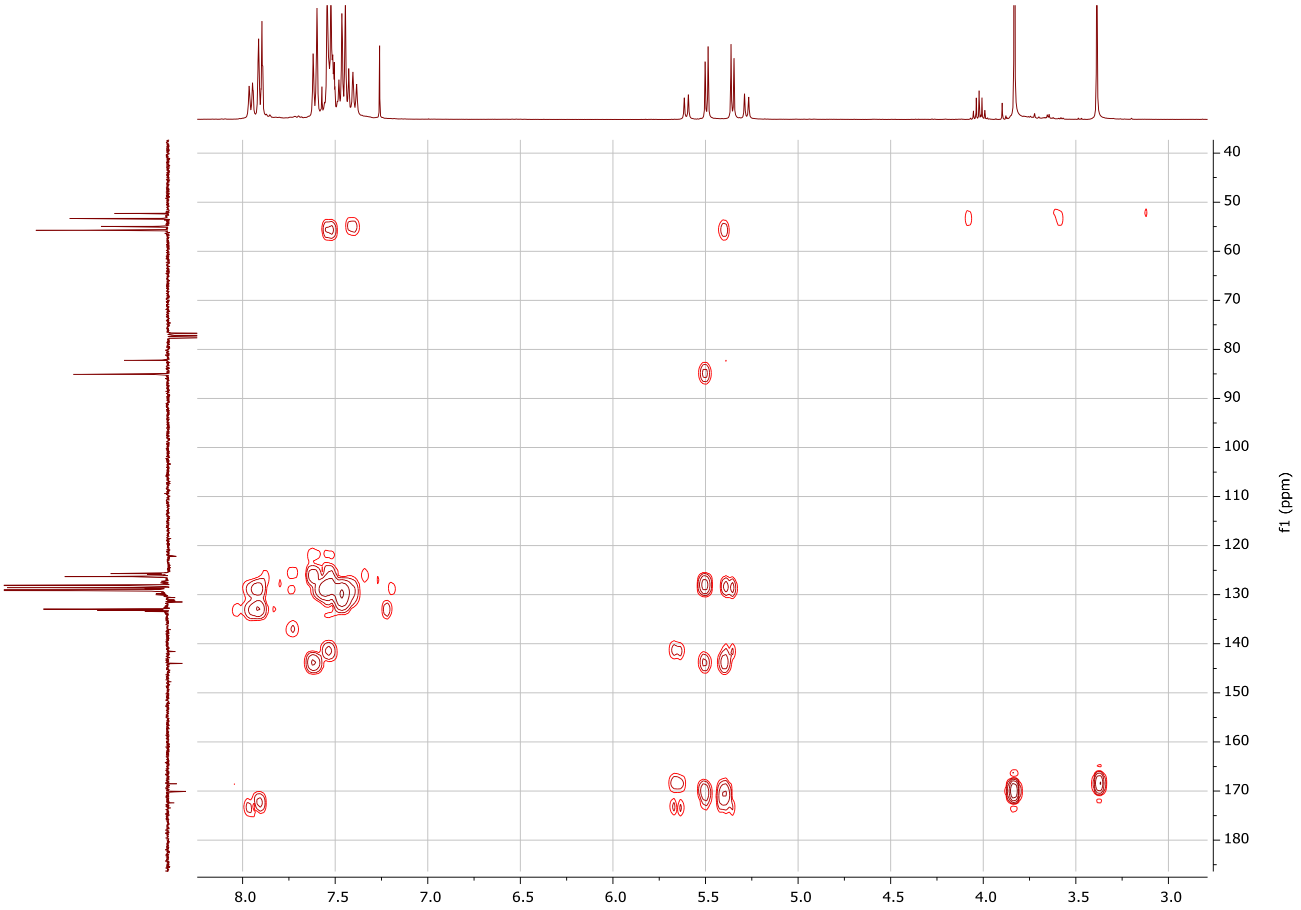

^{1}H-^{13}C HMBC correlation spectrum of **5h**

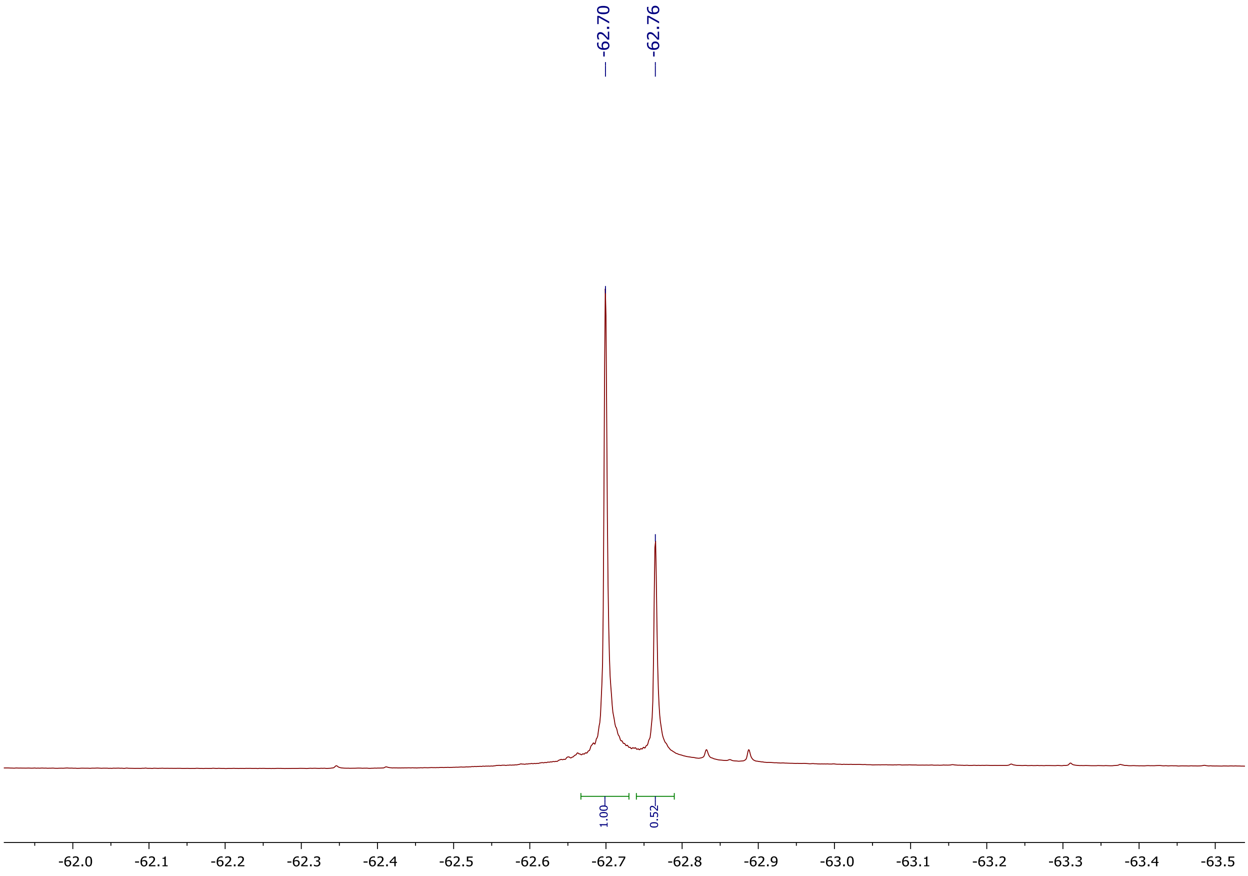

^{19}F NMR spectrum ($CDCl_3$, 282.40 MHz) of **5h**

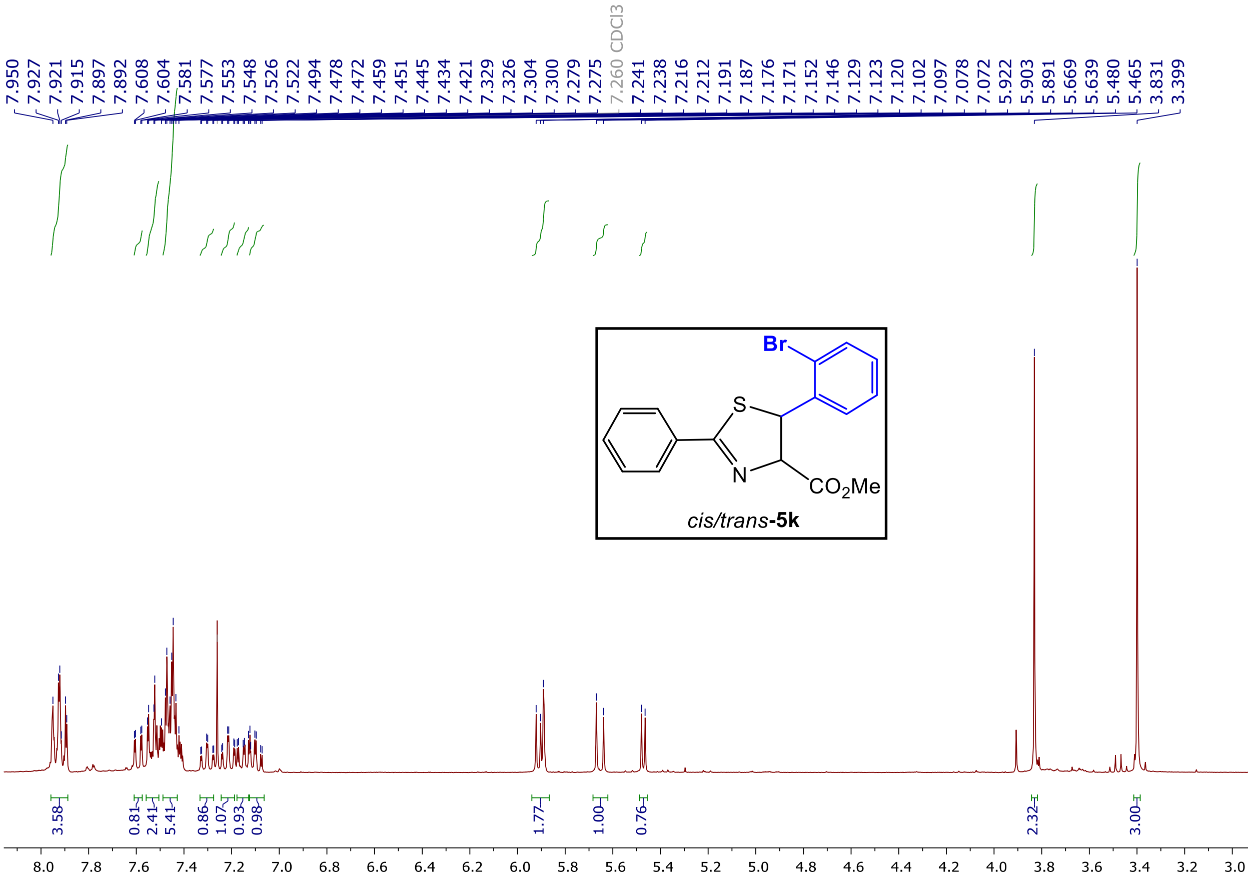

^{1}H NMR spectrum ($CDCl_3$, 300.13 MHz) of **5k**

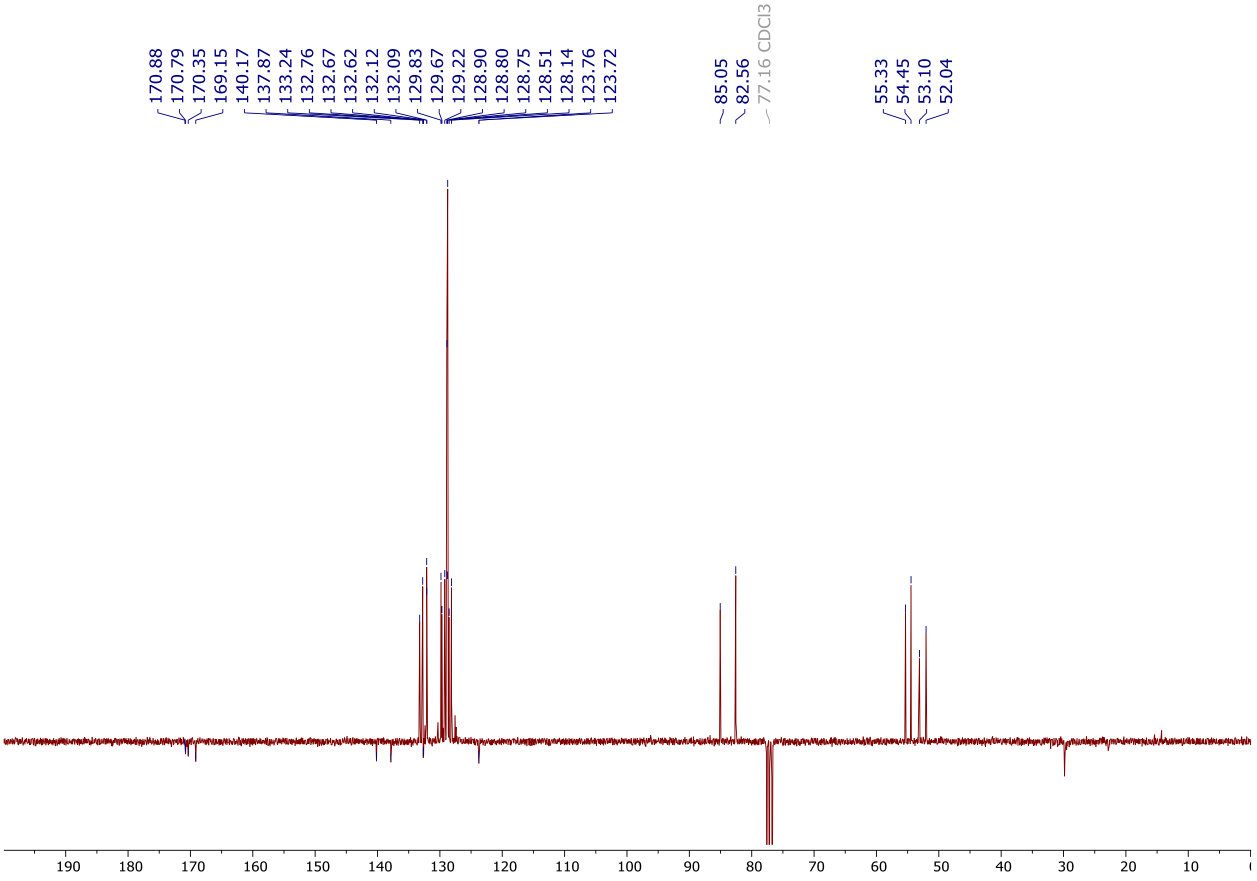

$^{13}C\{^{1}H\}$ NMR (APT) spectrum ($CDCl_3$, 75.5 MHz) of **5k**

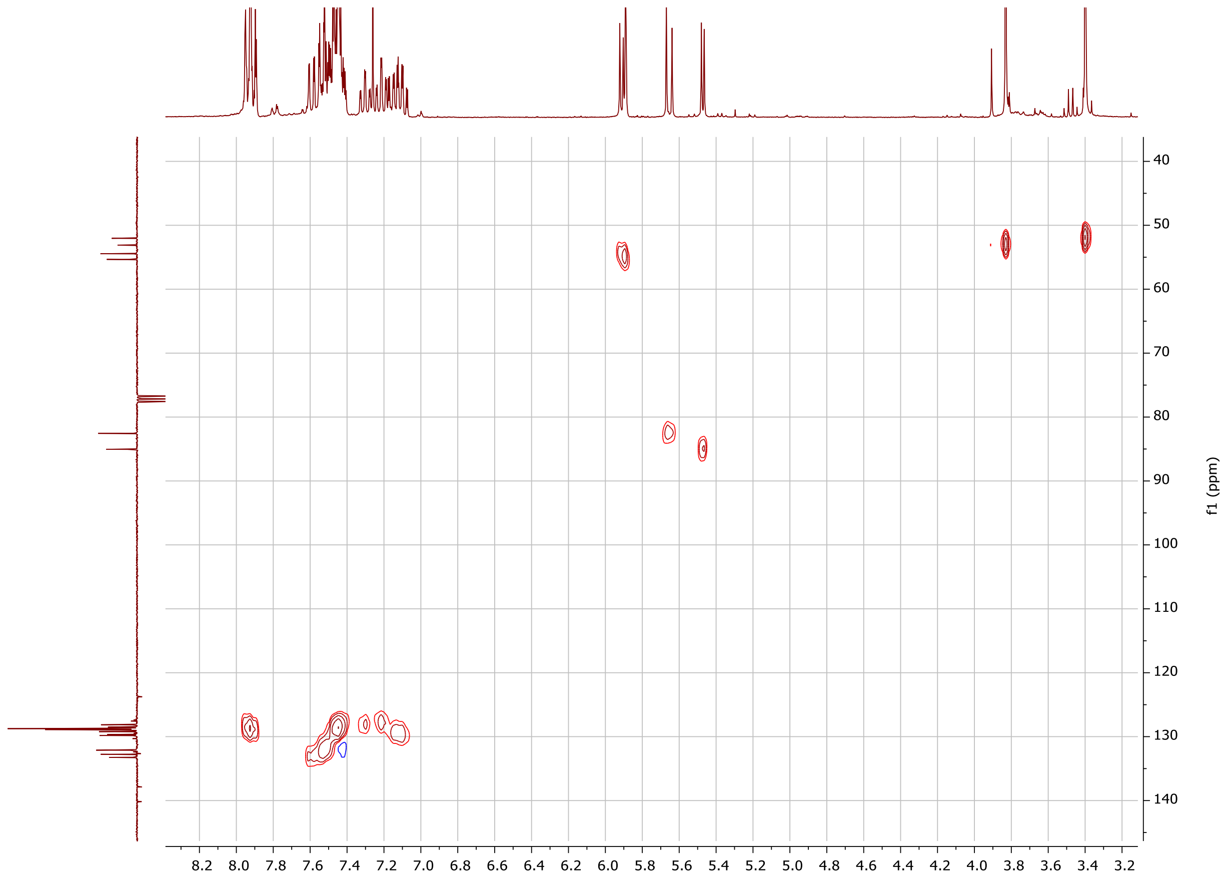

^{1}H-^{13}C HSQC correlation spectrum of **5k**

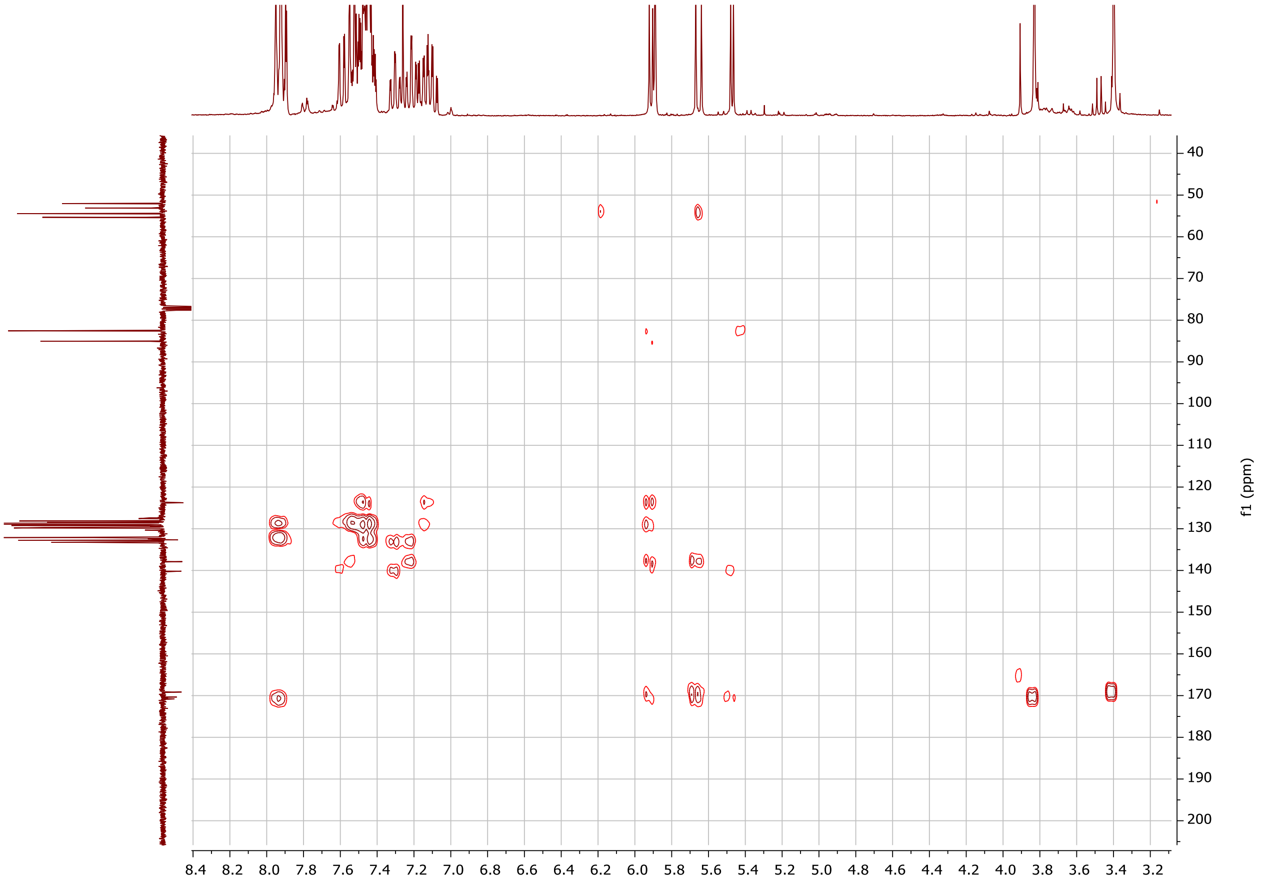

^{1}H-^{13}C HMBC correlation spectrum of **5k**

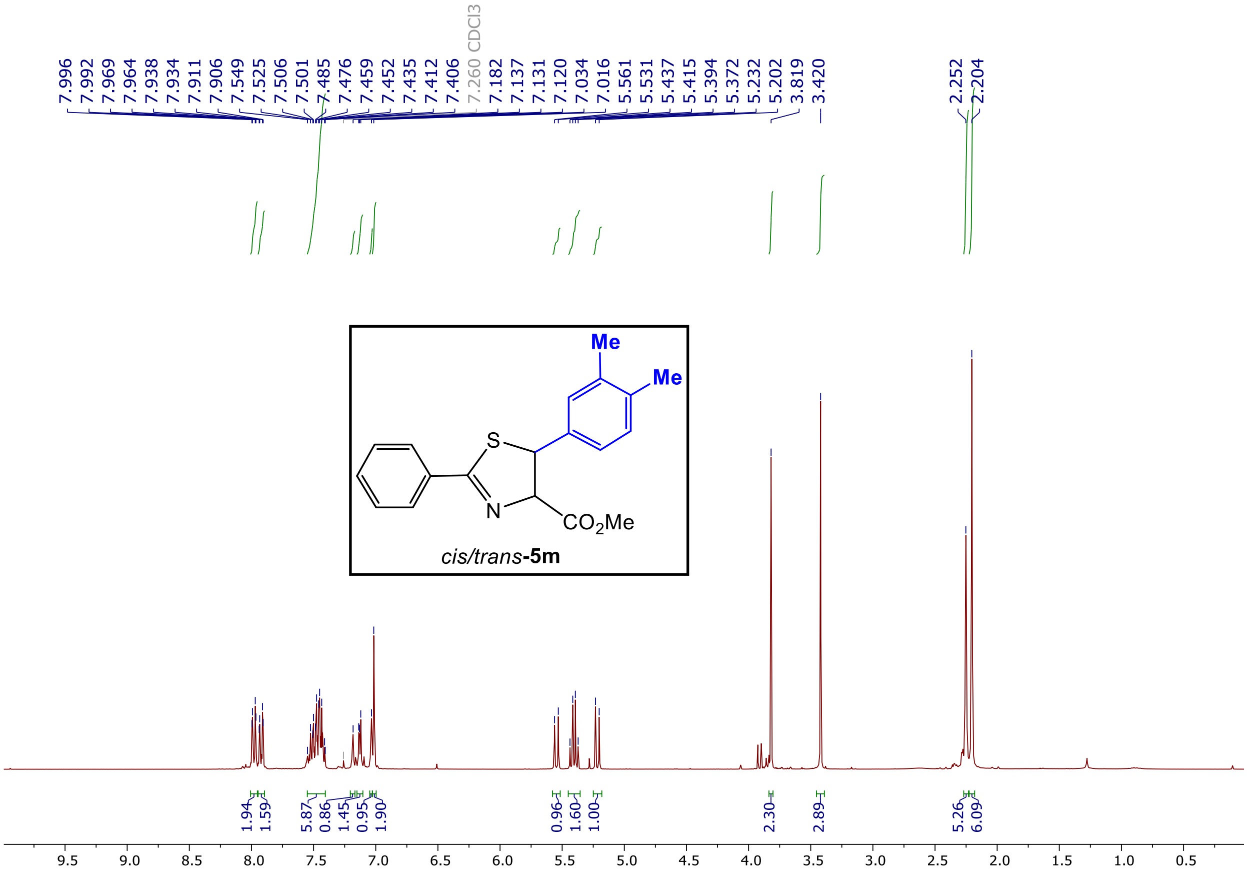

^{1}H NMR spectrum ($CDCl_3$, 300.13 MHz) of **5m**

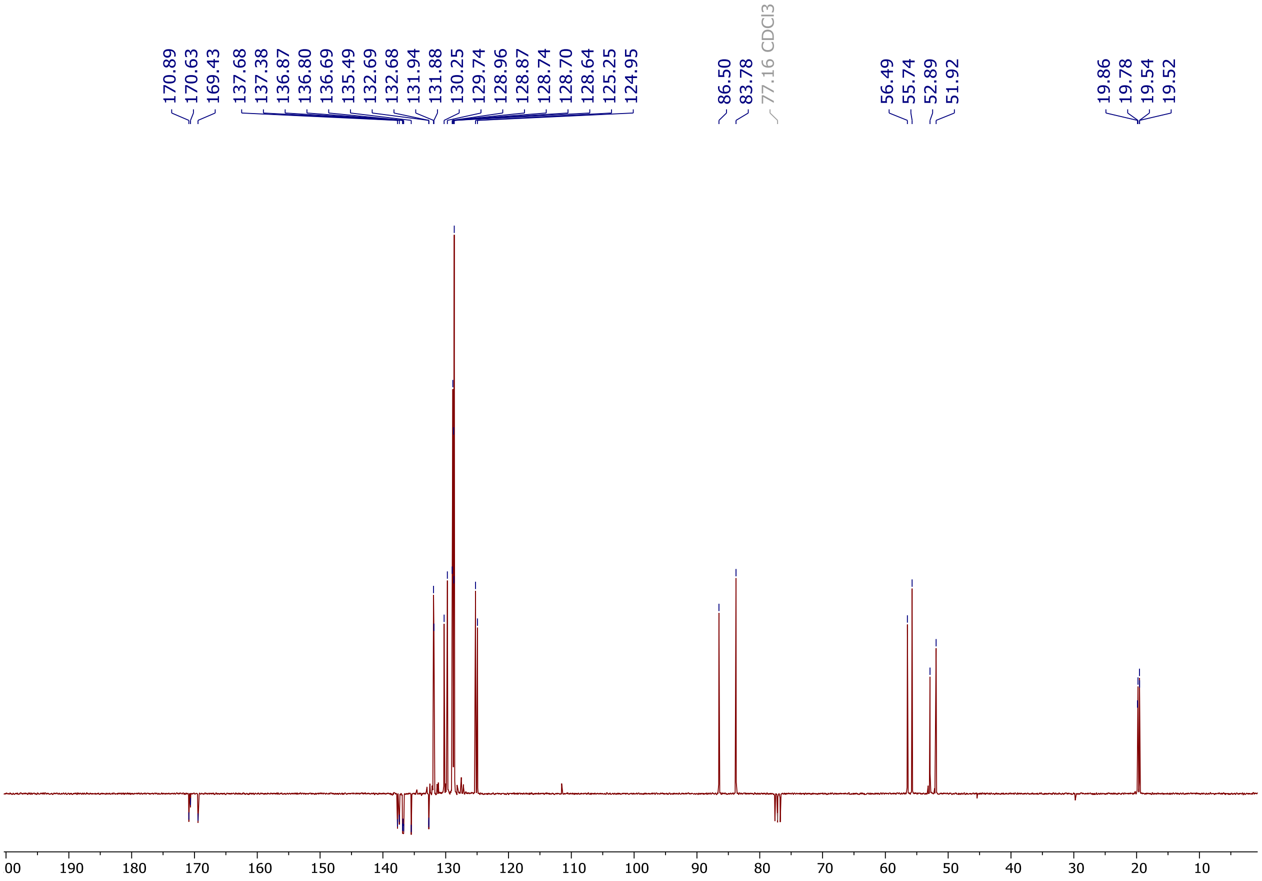

$^{13}C\{^{1}H\}$ NMR (APT) spectrum ($CDCl_3$, 75.5 MHz) of **5m**

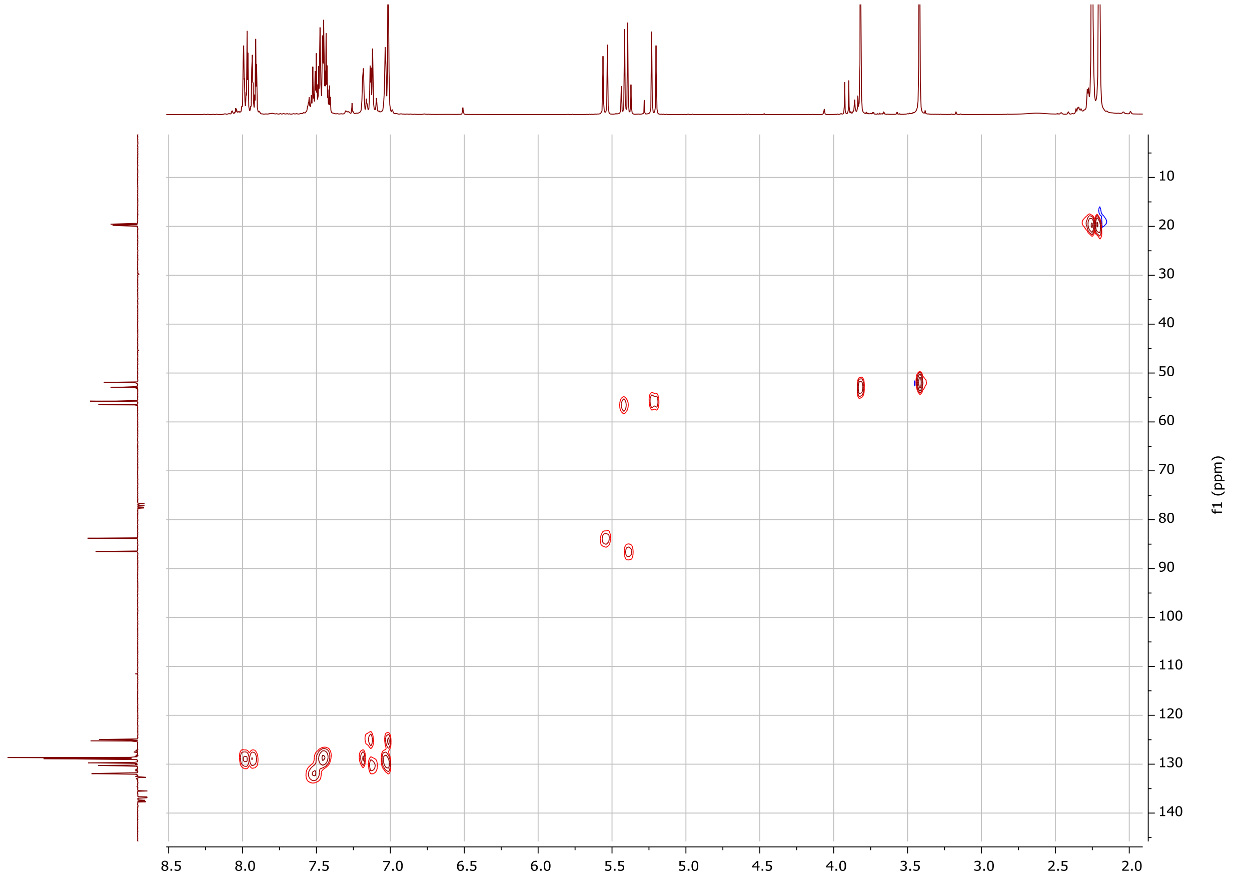

^{1}H-^{13}C HSQC correlation spectrum of **5m**

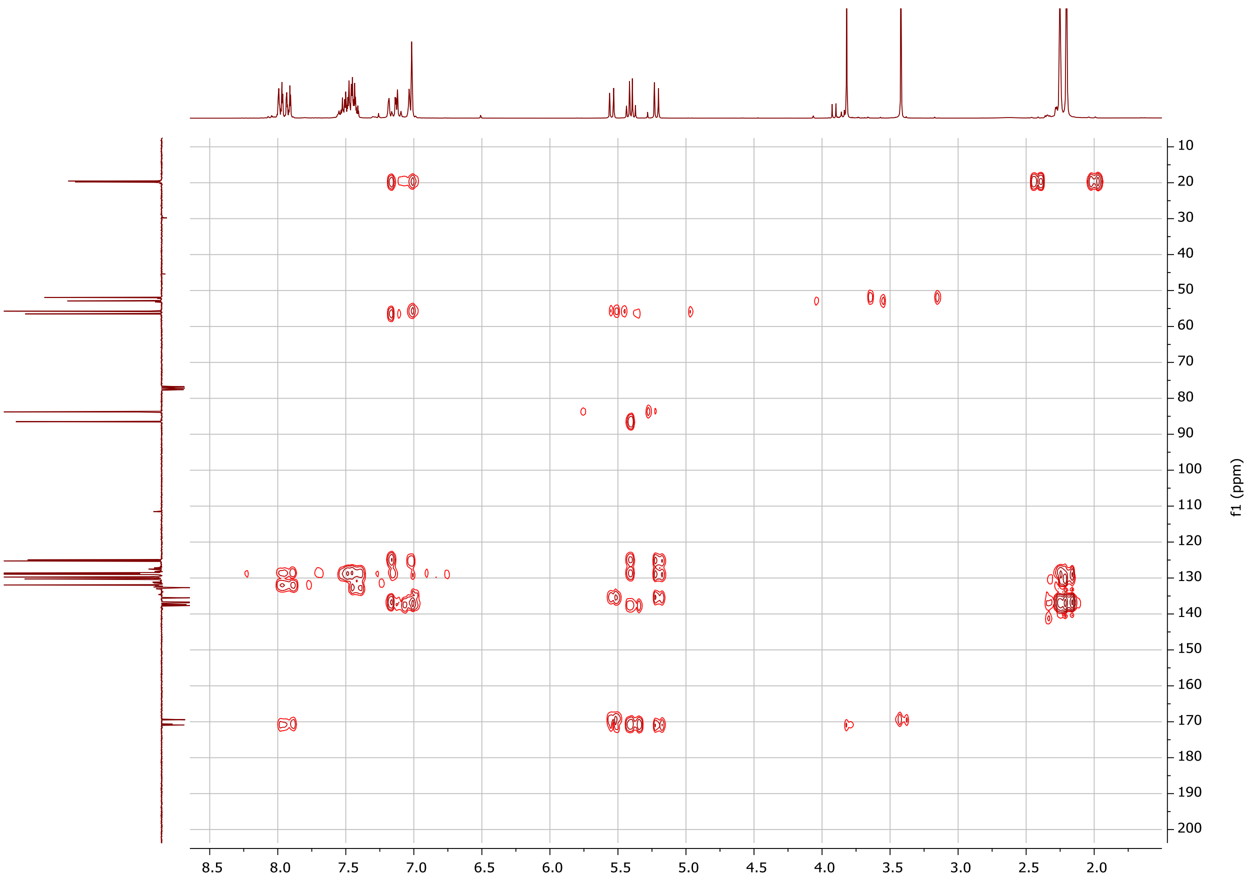

^{1}H-^{13}C HMBC correlation spectrum of **5m**

6.- UV-Vis spectra of thiazolones 2 and selected cyclobutanes 3

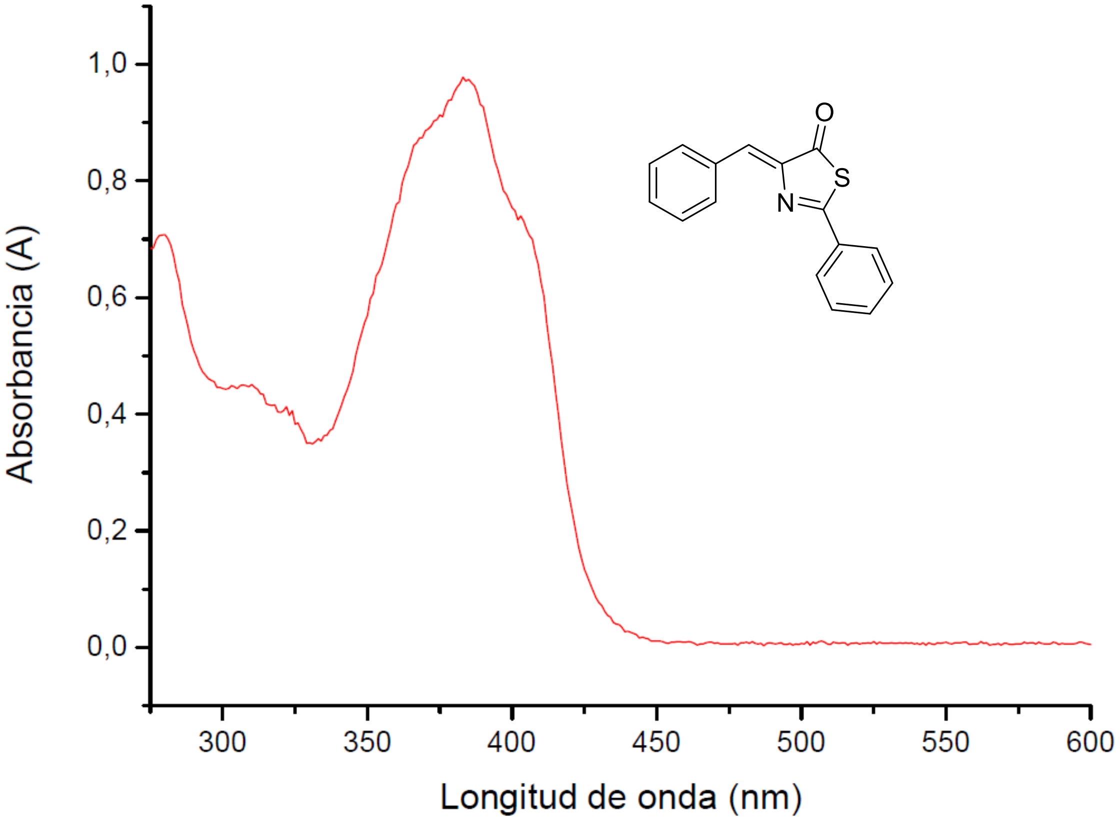

UV-Vis spectrum (CH_2Cl_2, 2×10^{-5} M) of **2a**

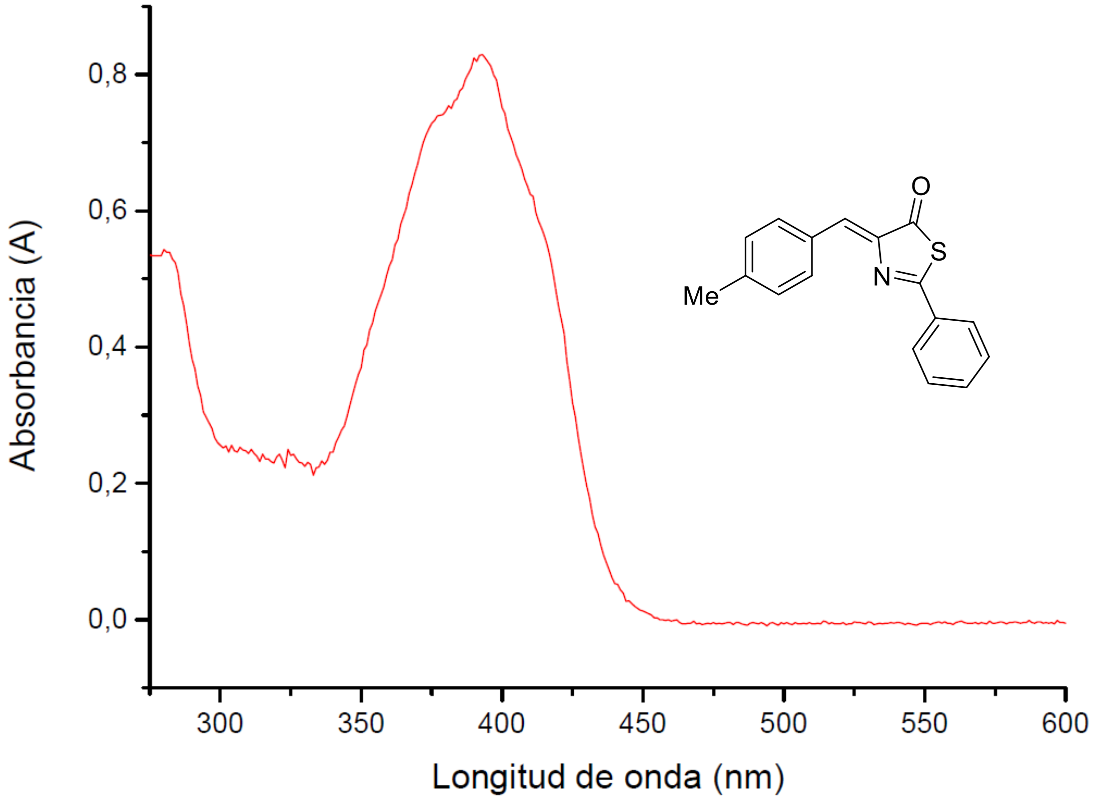

UV-Vis spectrum (CH_2Cl_2, 2×10^{-5} M) of **2b**

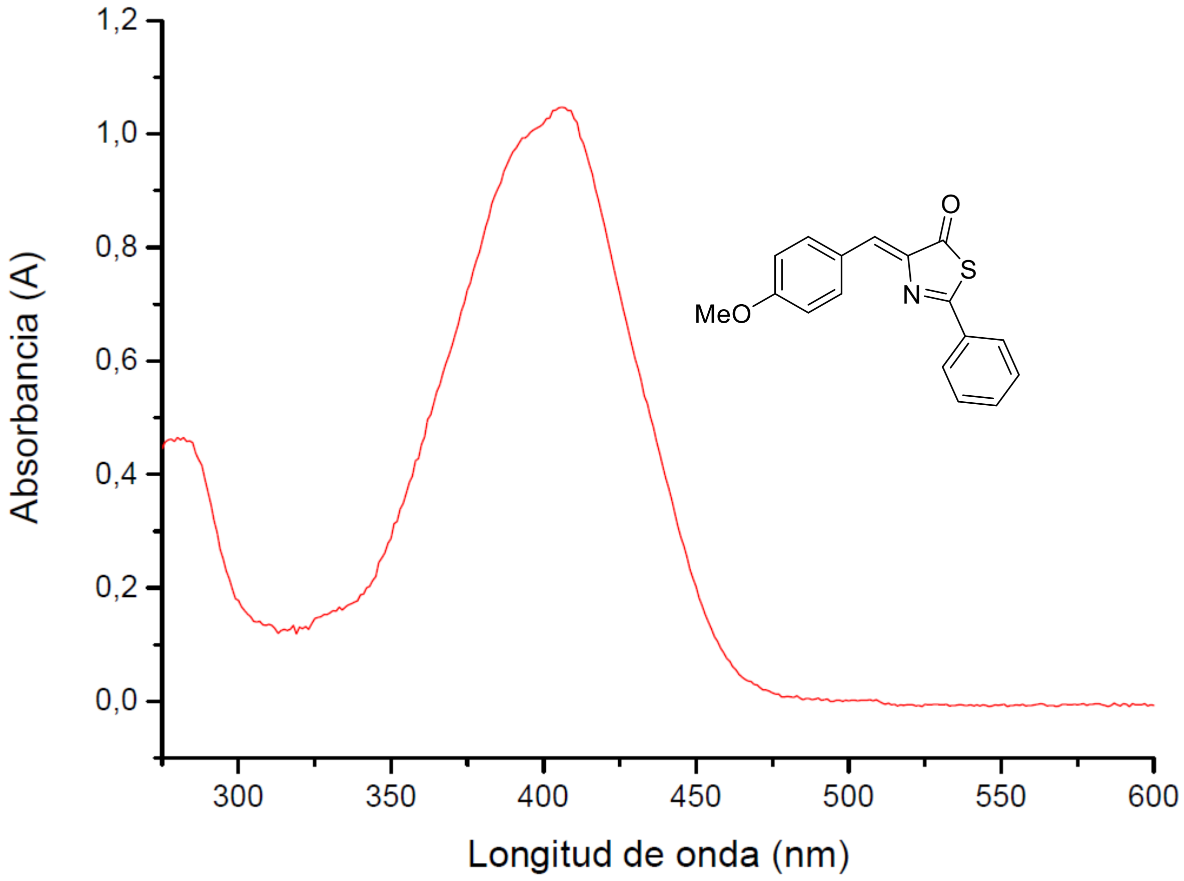

UV-Vis spectrum (CH_2Cl_2, $2{\times}10^{-5}$ M) of **2c**

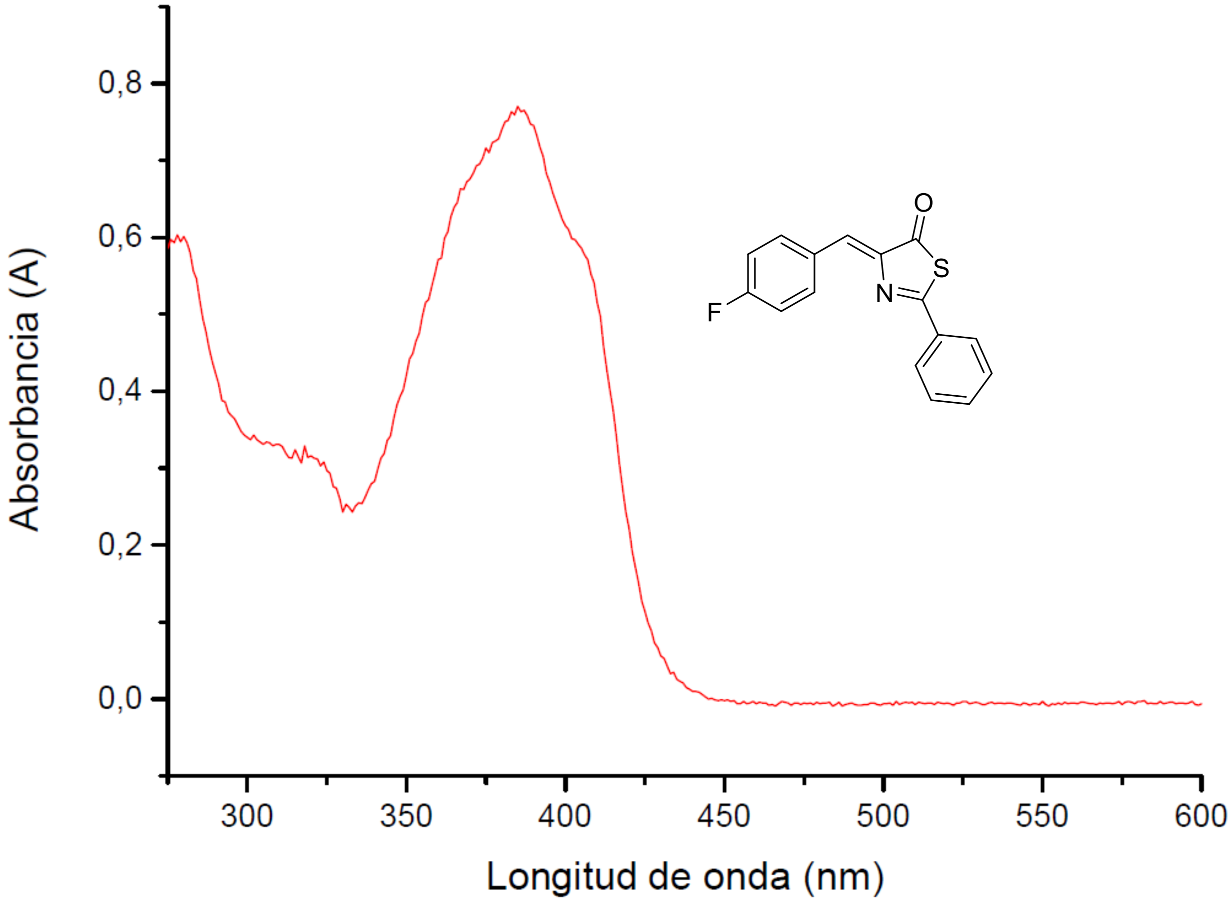

UV-Vis spectrum (CH_2Cl_2, $2{\times}10^{-5}$ M) of **2d**

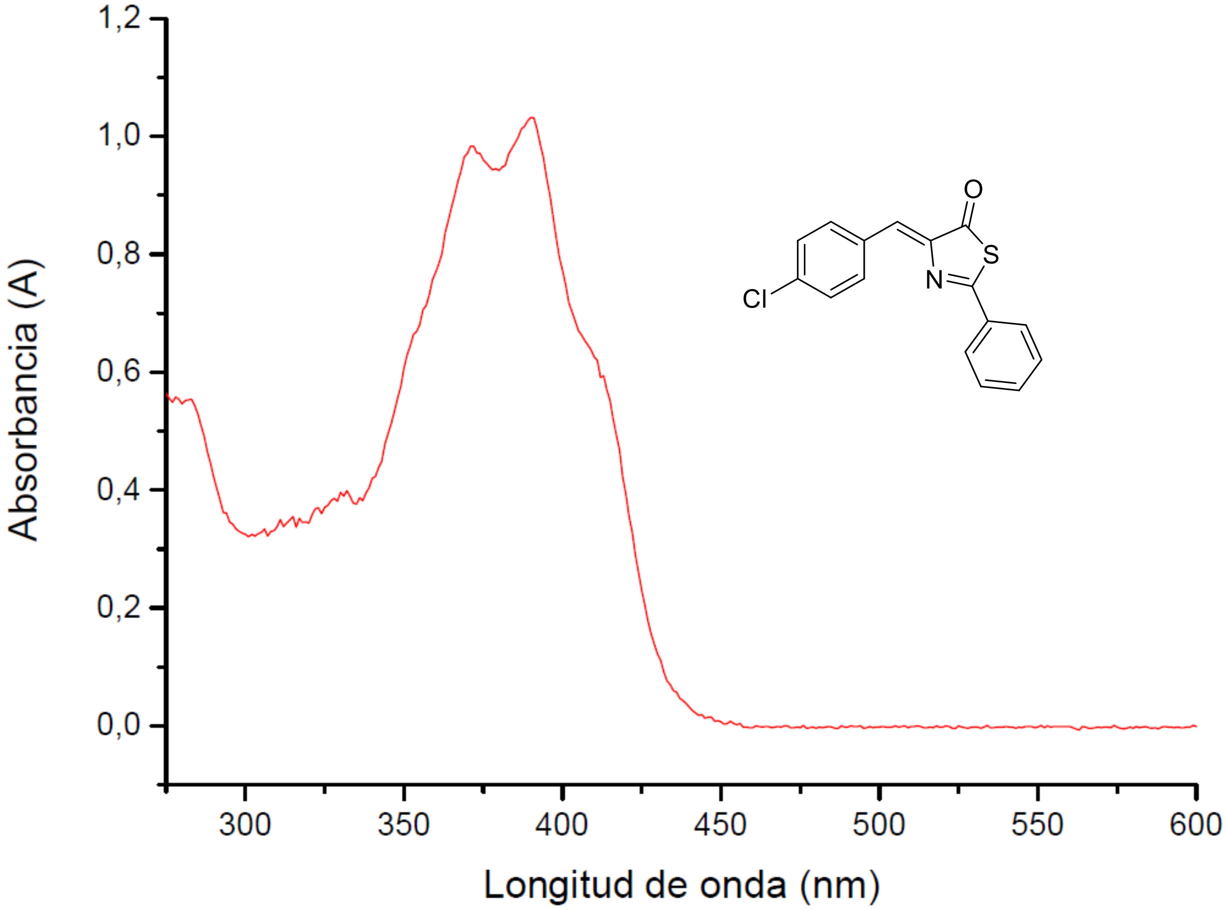

UV-Vis spectrum (CH_2Cl_2, 2×10^{-5} M) of **2e**

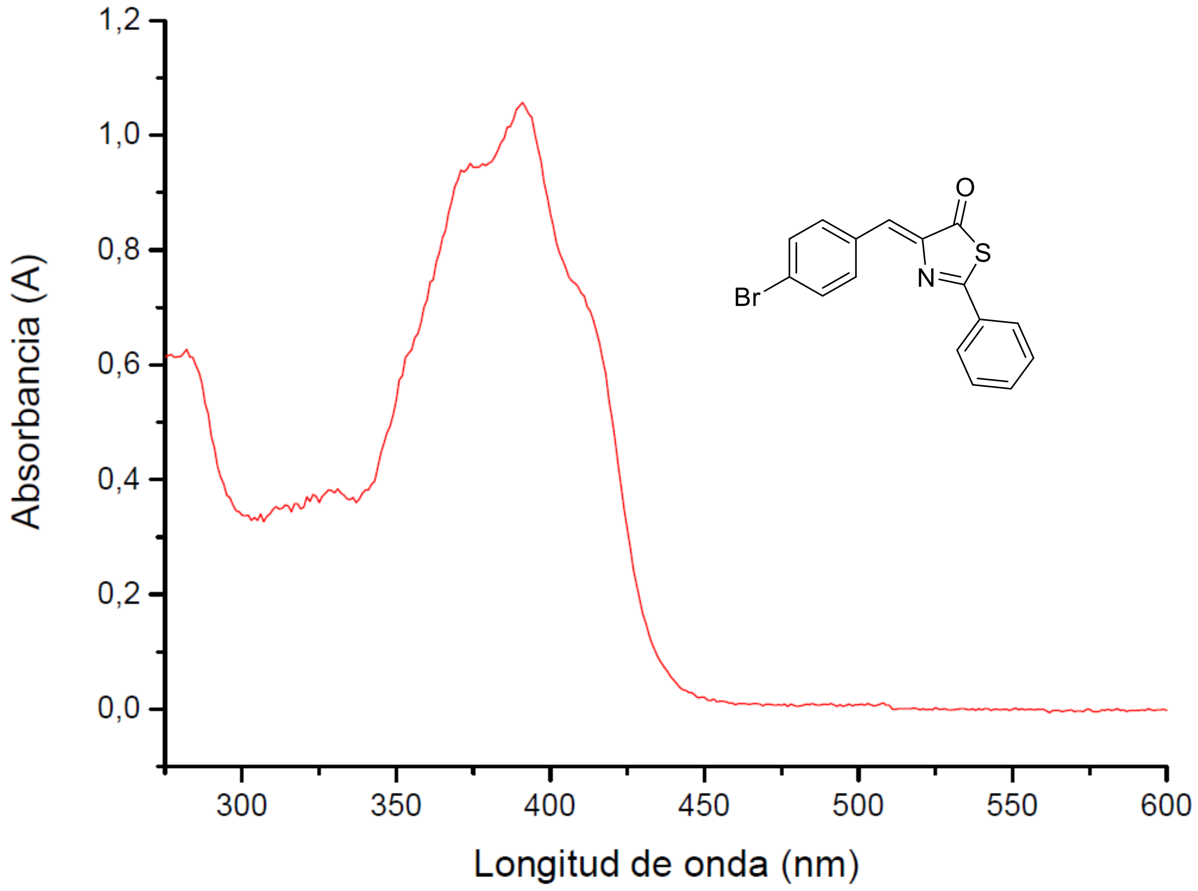

UV-Vis spectrum (CH_2Cl_2, 2×10^{-5} M) of **2f**

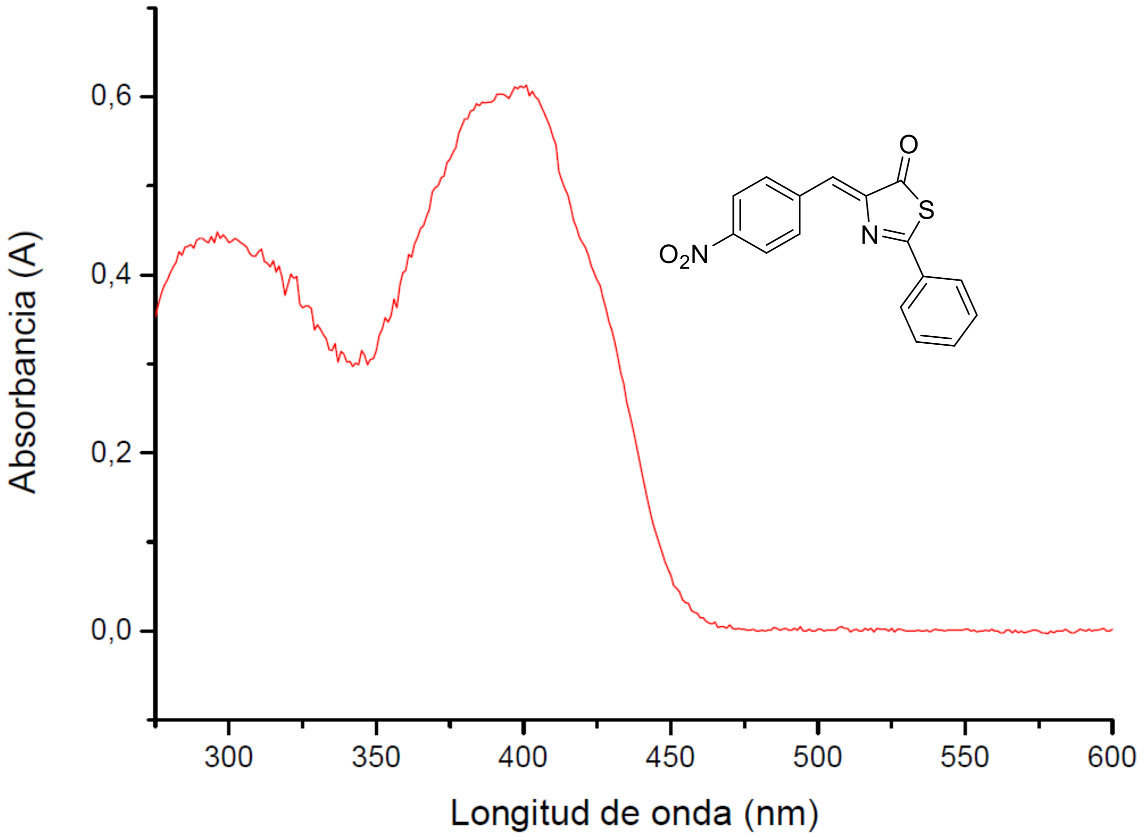

UV-Vis spectrum (CH_2Cl_2, 2×10^{-5} M) of **2g**

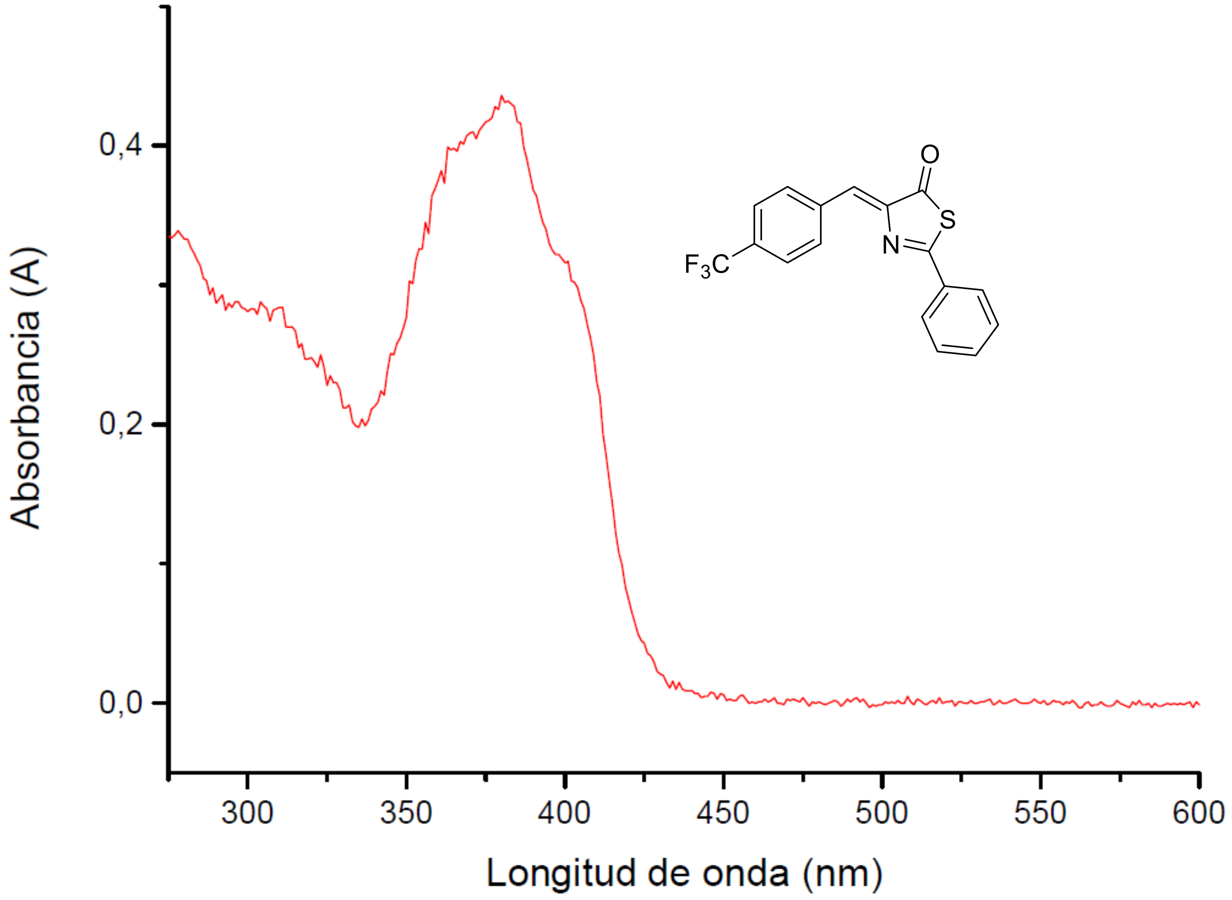

UV-Vis spectrum (CH_2Cl_2, 2×10^{-5} M) of **2h**

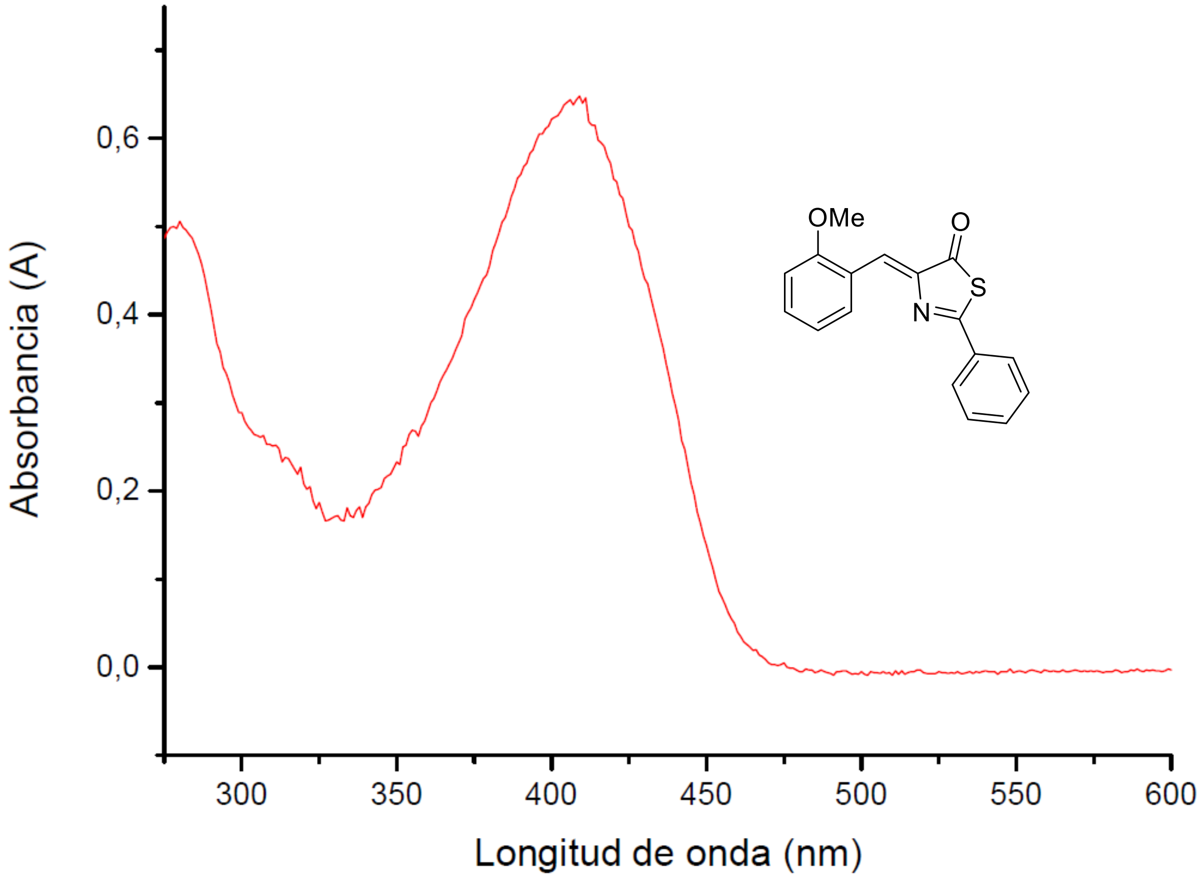

UV-Vis spectrum (CH_2Cl_2, 2×10^{-5} M) of **2i**

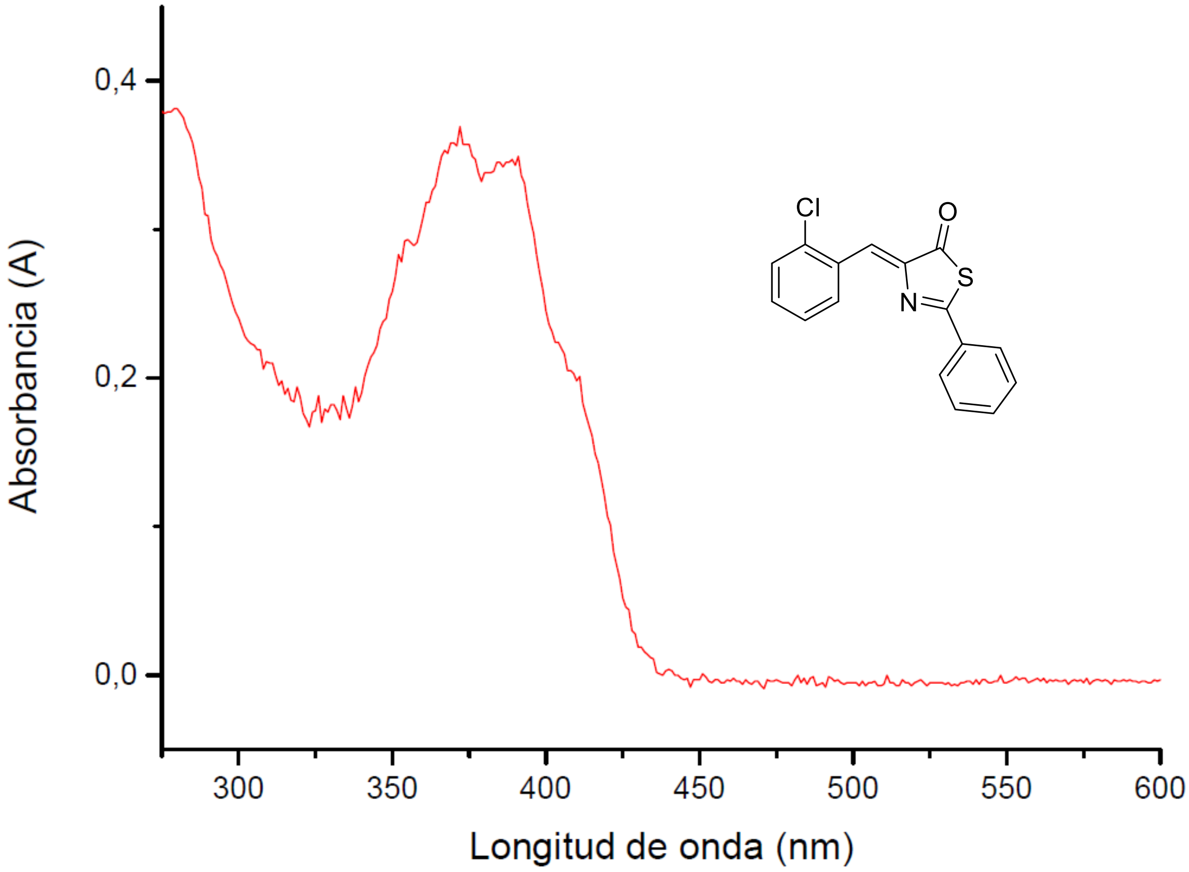

UV-Vis spectrum (CH_2Cl_2, 2×10^{-5} M) of **2j**

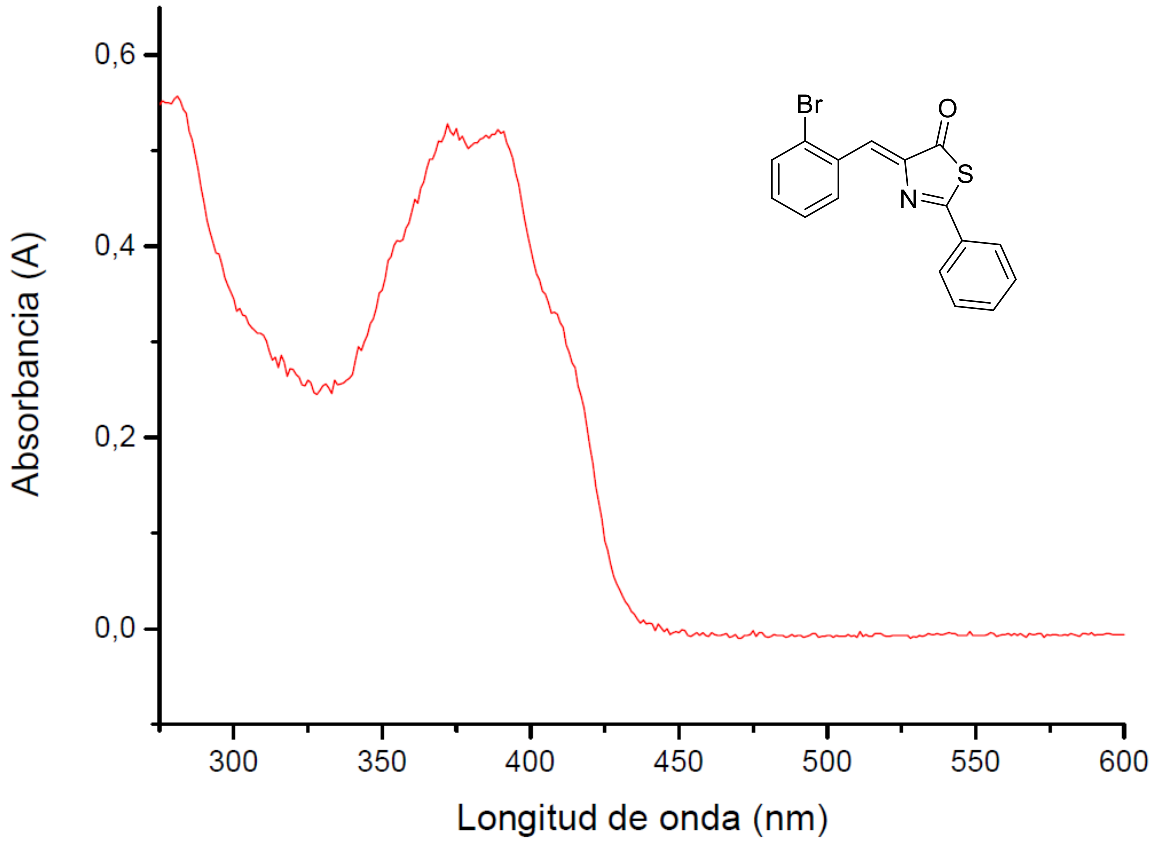

UV-Vis spectrum (CH_2Cl_2, 2×10^{-5} M) of **2k**

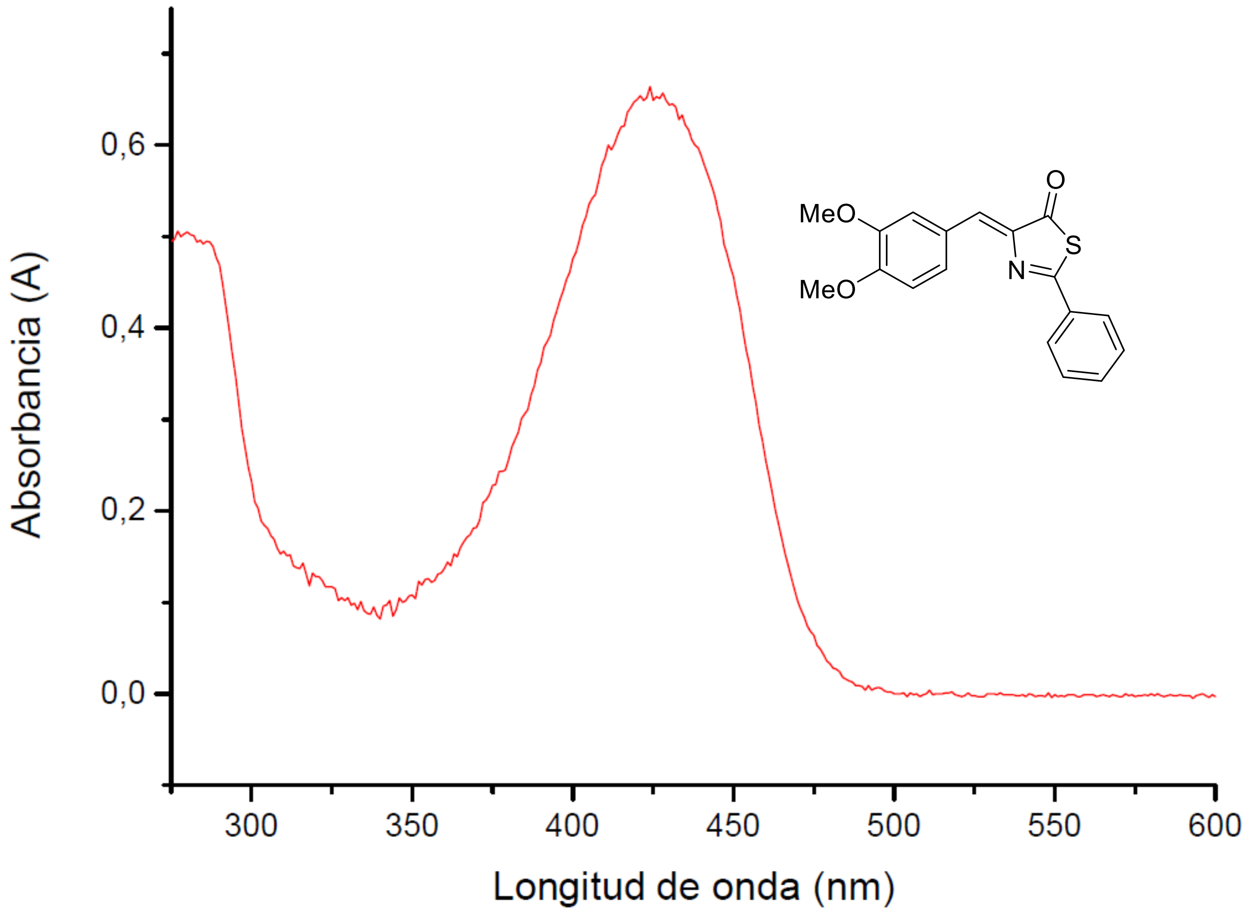

UV-Vis spectrum (CH_2Cl_2, 2×10^{-5} M) of **2l**

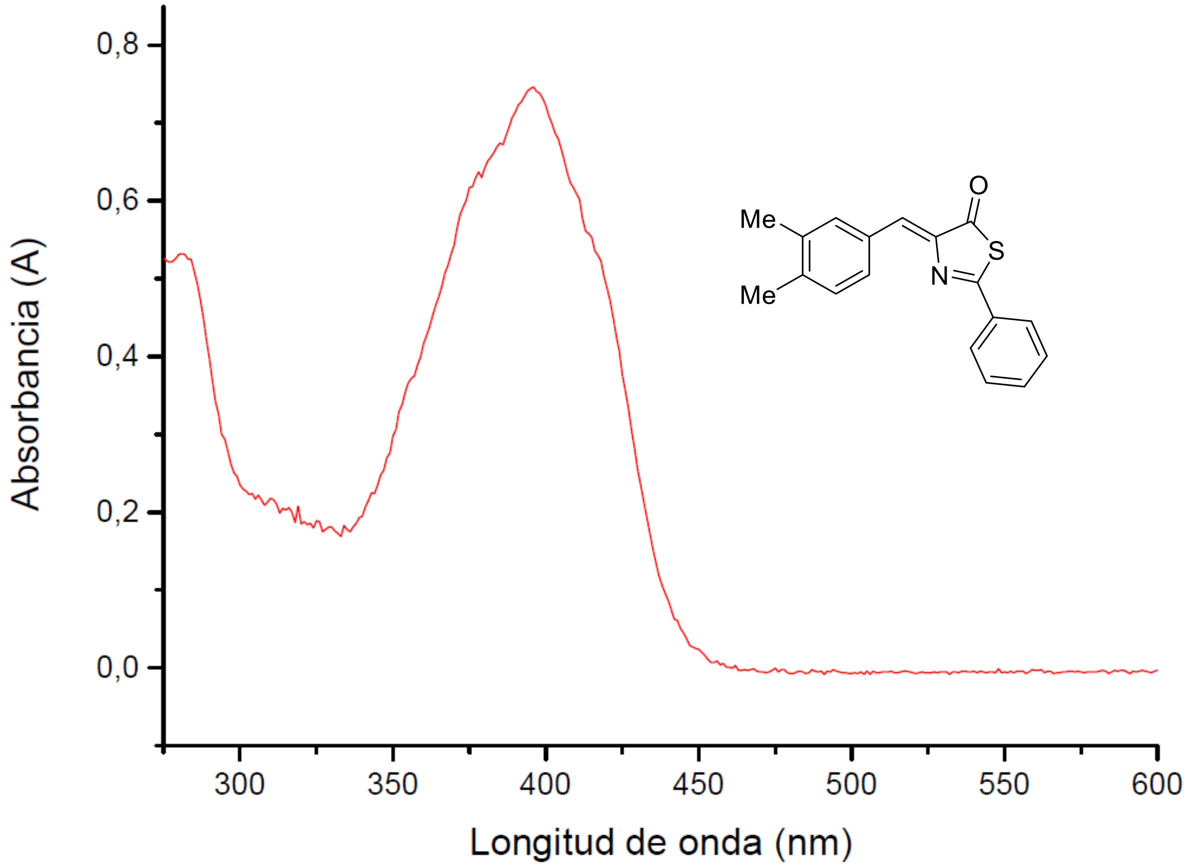

UV-Vis spectrum (CH_2Cl_2, 2×10^{-5} M) of **2m**

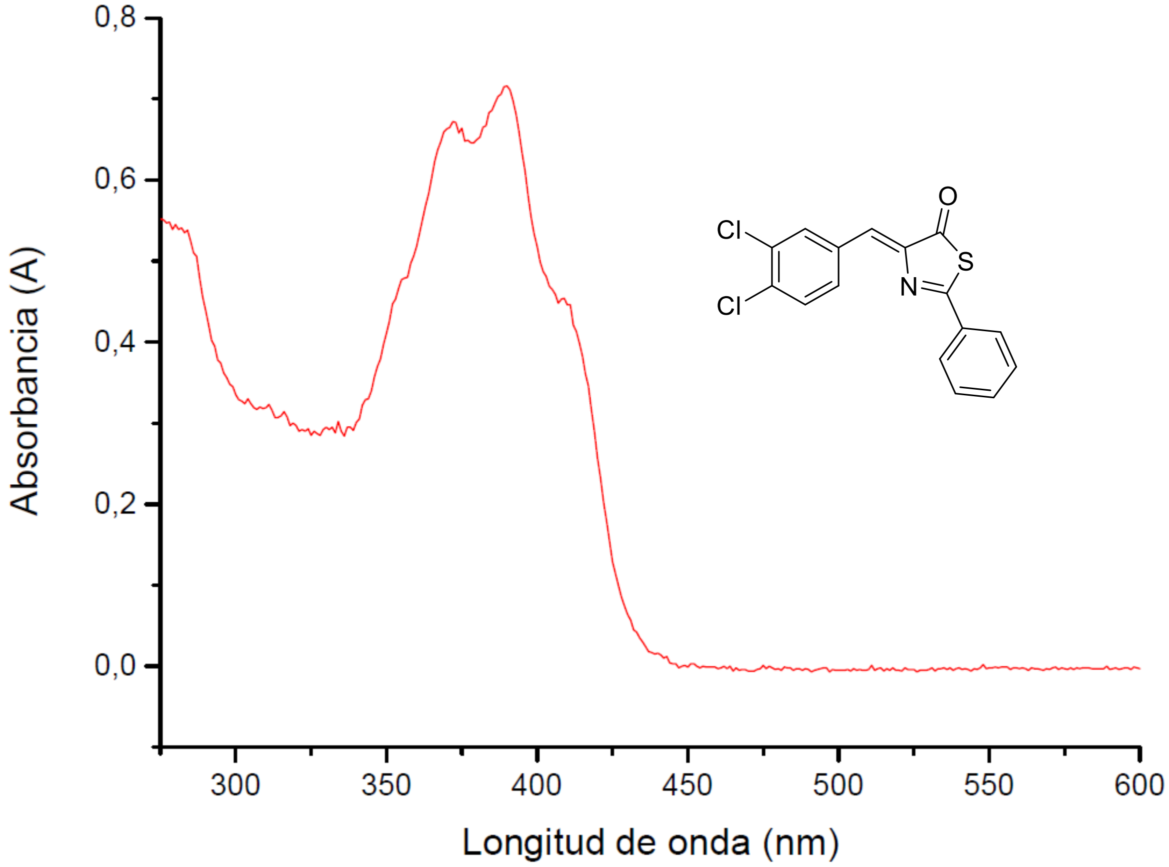

UV-Vis spectrum (CH_2Cl_2, 2×10^{-5} M) of **2n**

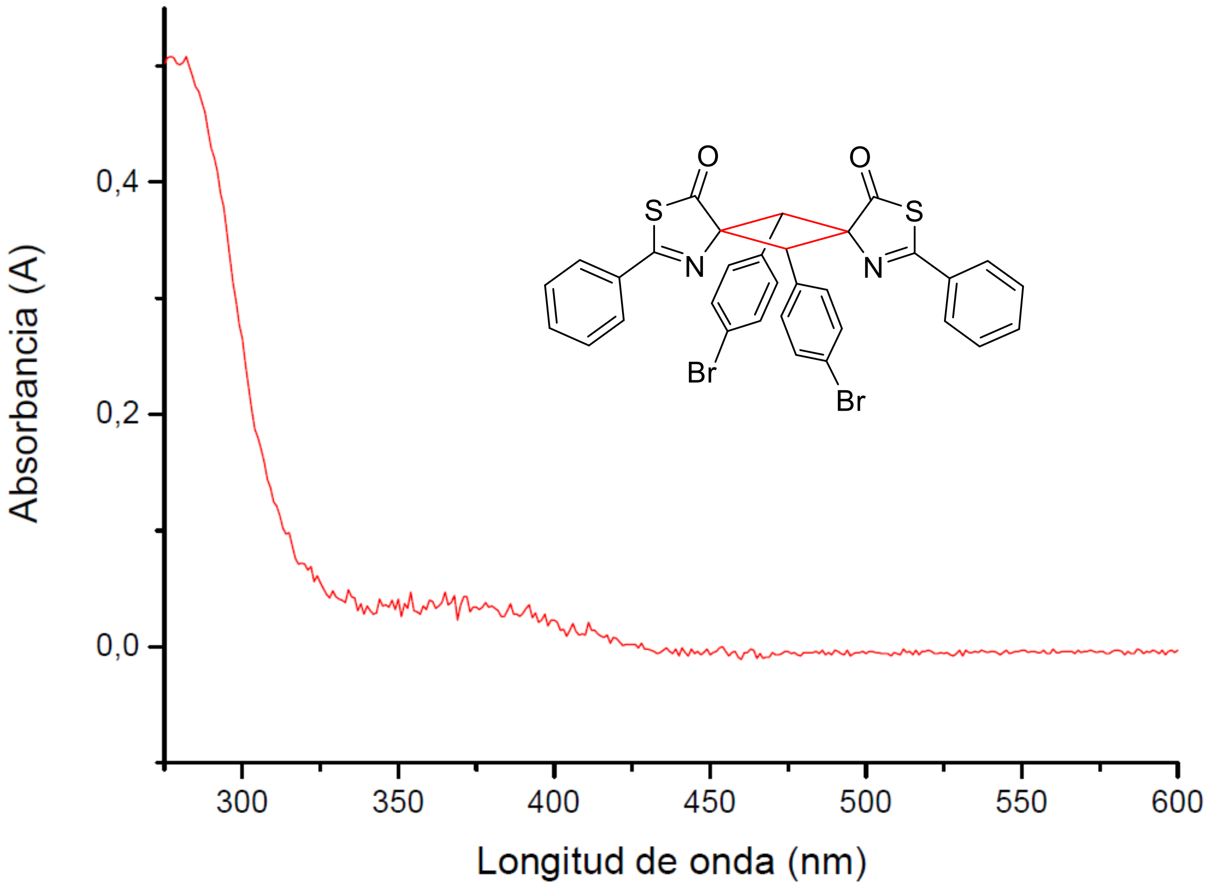

UV-Vis spectrum (CH_2Cl_2, 2×10^{-5} M) of **3f**

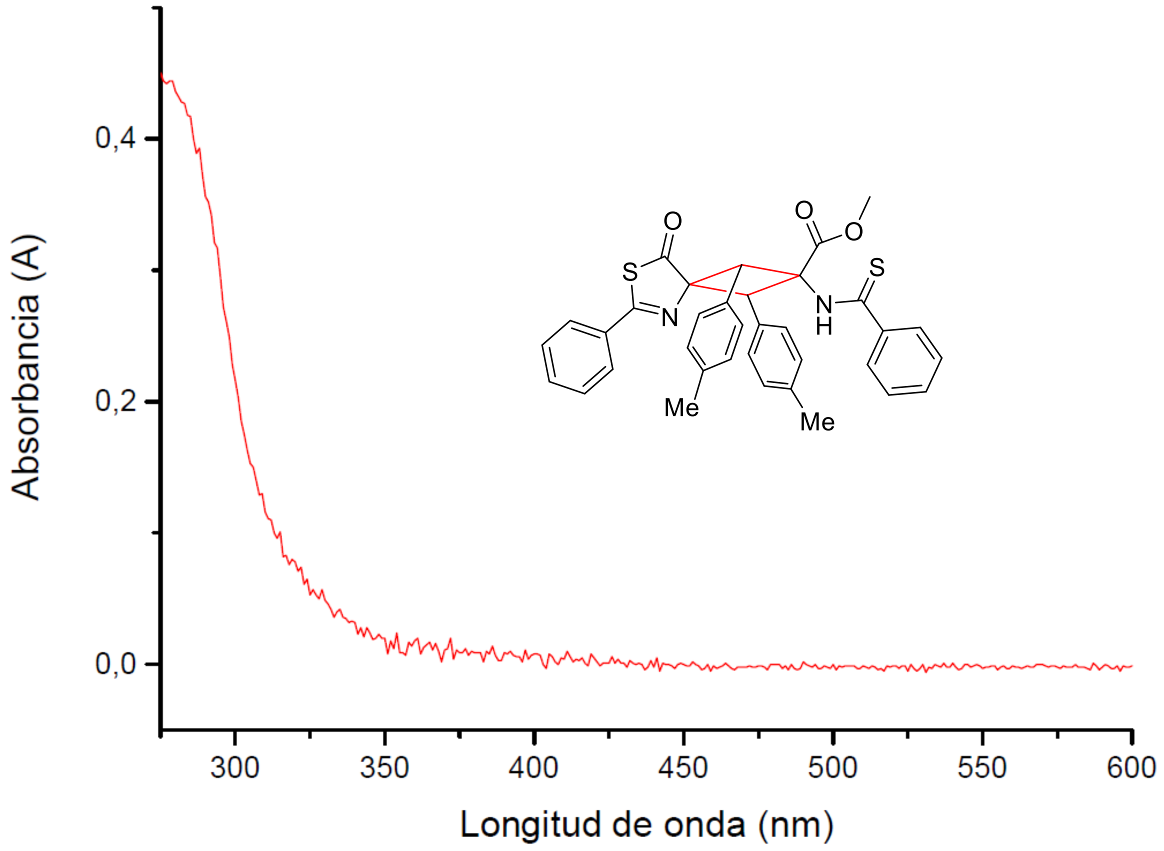

UV-Vis spectrum (CH_2Cl_2, 2×10^{-5} M) of **4b**

7.- NMR spectra and UV-Vis spectra of 2b in CD_3OD in presence of increasing amounts of BF_3

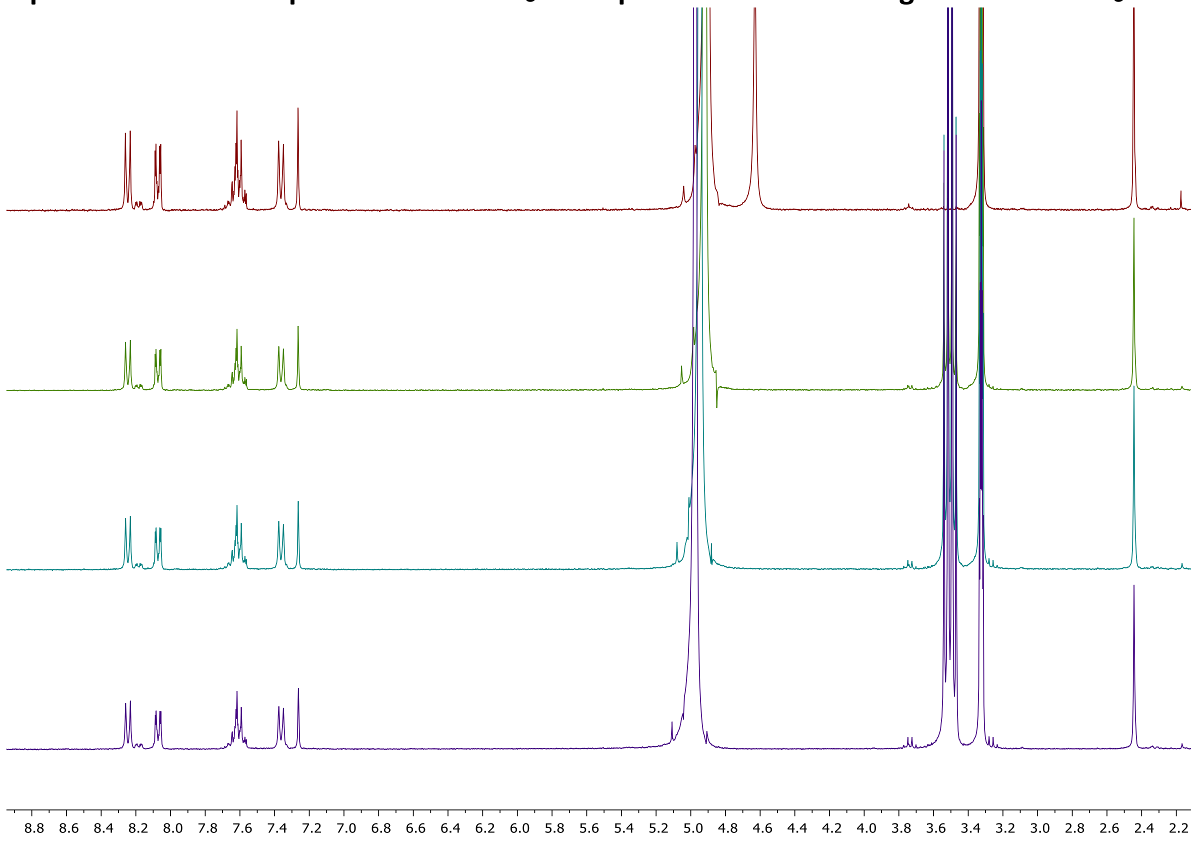

^1H NMR spectra of **2b** in CD_3OD (top); after addition of 1 equiv of $BF_3{\cdot}Et_2O$ (top middle); after addition of 2 equivs of $BF_3{\cdot}Et_2O$ (bottom middle); after addition of 4 equivs of $BF_3{\cdot}Et_2O$ (bottom).

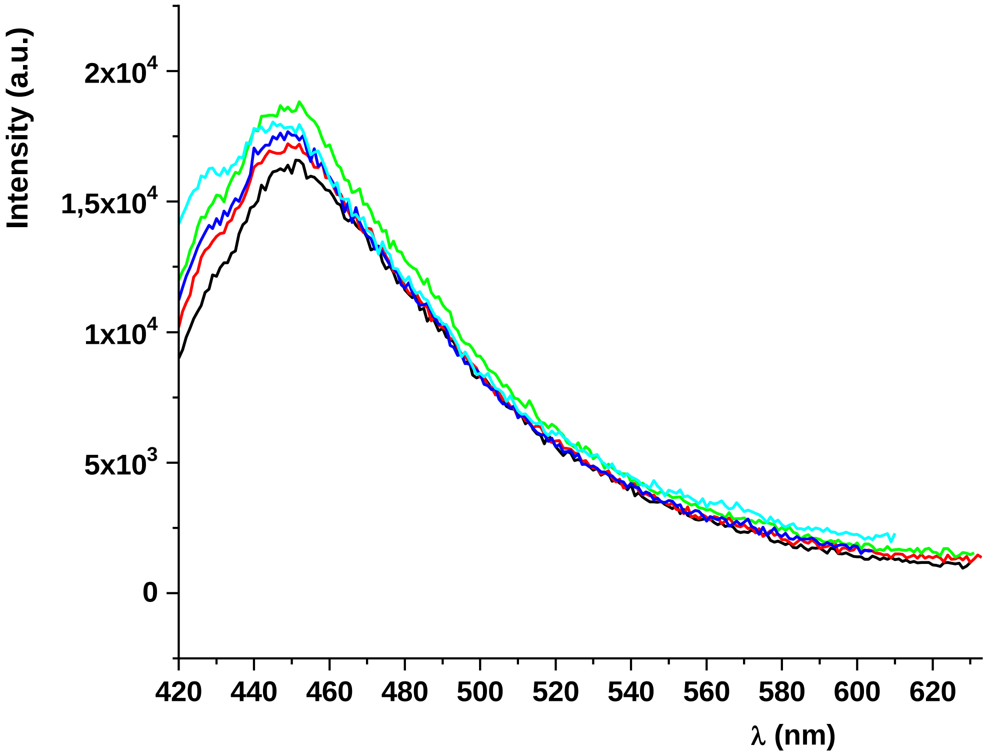

Emission spectrum of **2b** in CH_3OH (λ_{exc} = 390 nm; pale blue line); overlapped are the emission spectra of **2b** after addition of 1 (blue), 2 (green), 3 (red) and 4 equivs (black line) of $BF_3{\cdot}Et_2O$.

8.- ORTEP of compounds **3g**, **3h**, **3m**, **4b**·CH_2Cl_2 and **7n**

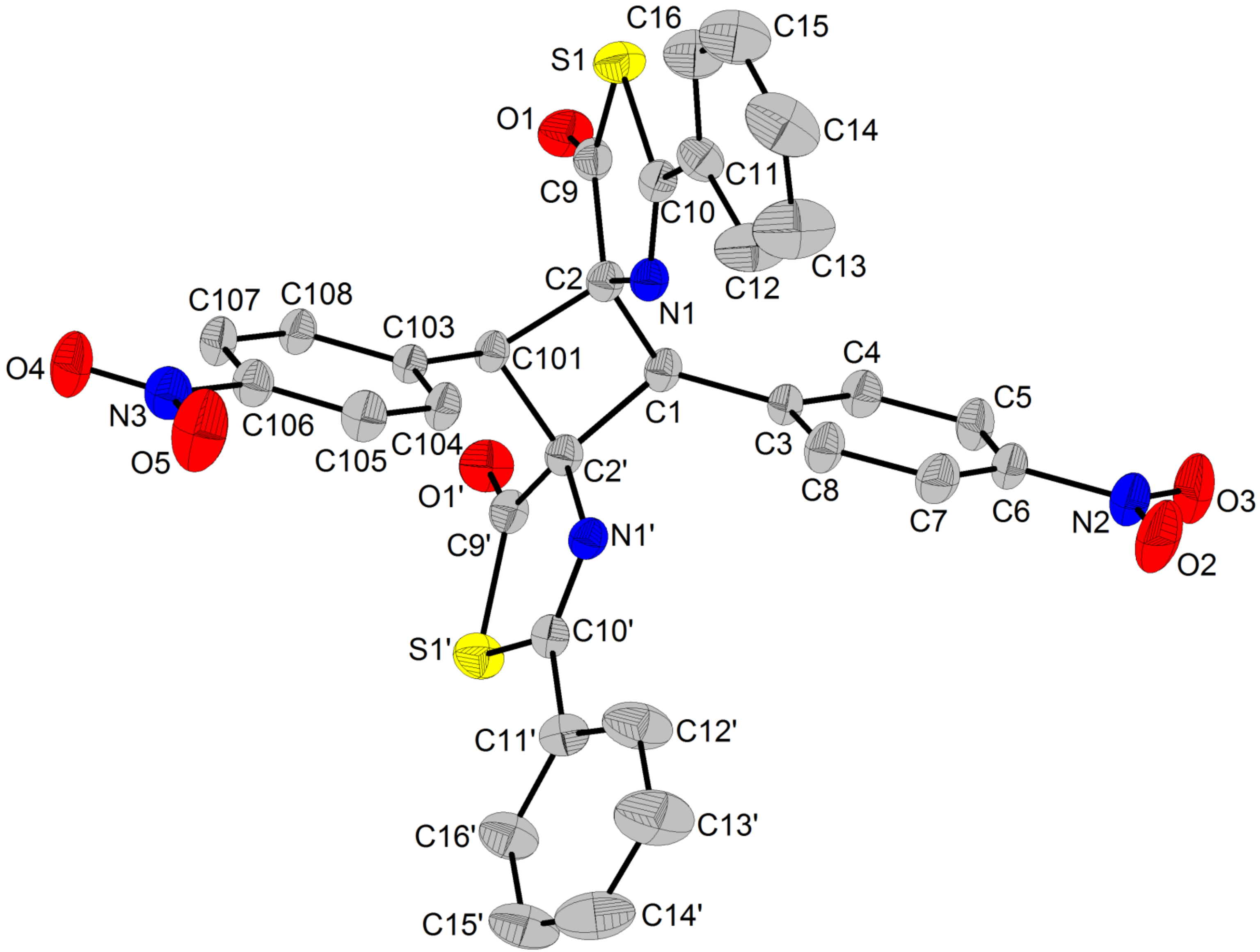

Molecular structure of **3g**. Thermal ellipsoids are drawn at 30% probability. Hydrogen atoms are omitted for clarity.

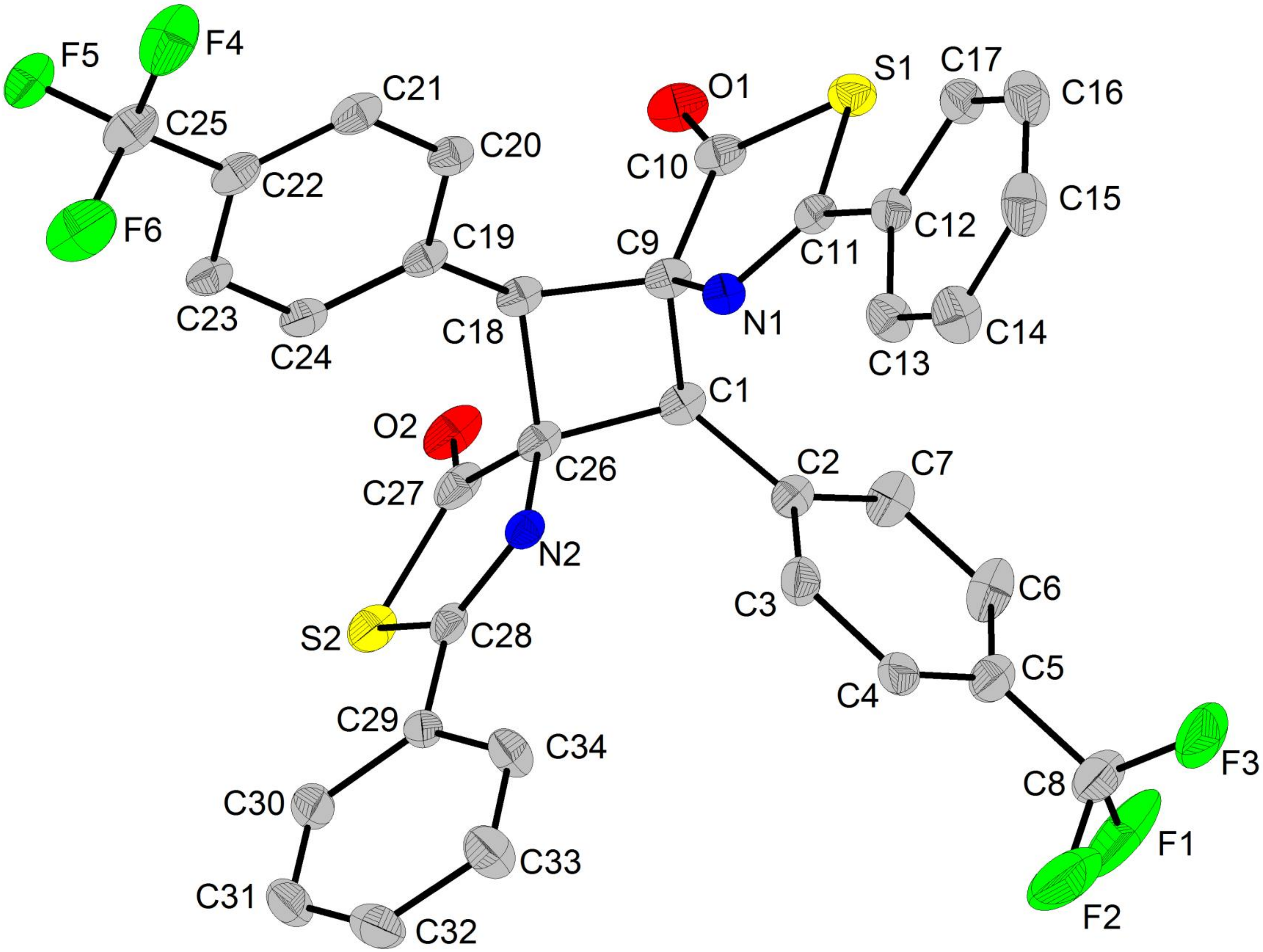

Molecular structure of **3h**. Thermal ellipsoids are drawn at 30% probability. Hydrogen atoms are omitted for clarity.

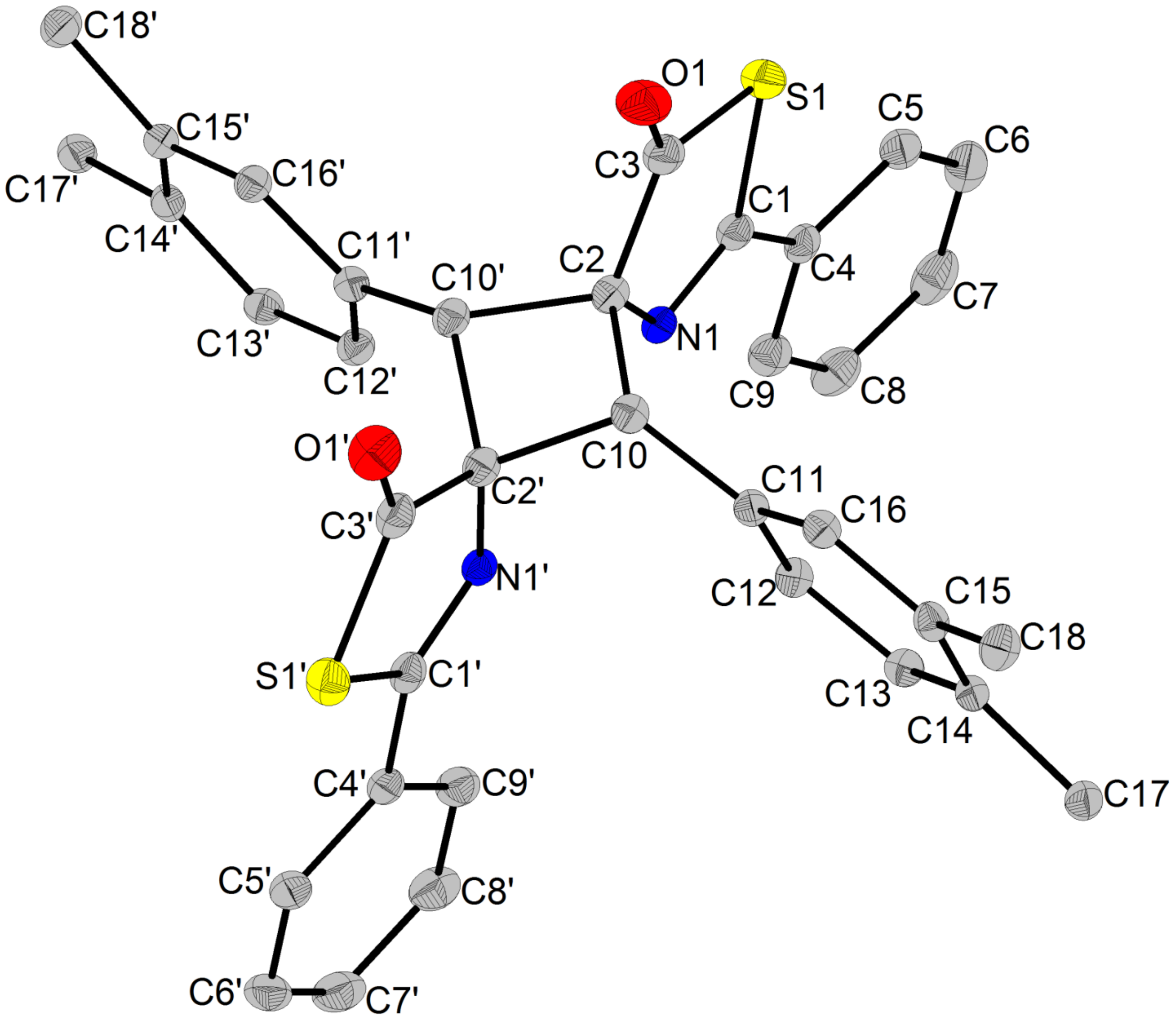

Molecular structure of **3m**. Thermal ellipsoids are drawn at 30% probability. Hydrogen atoms are omitted for clarity.

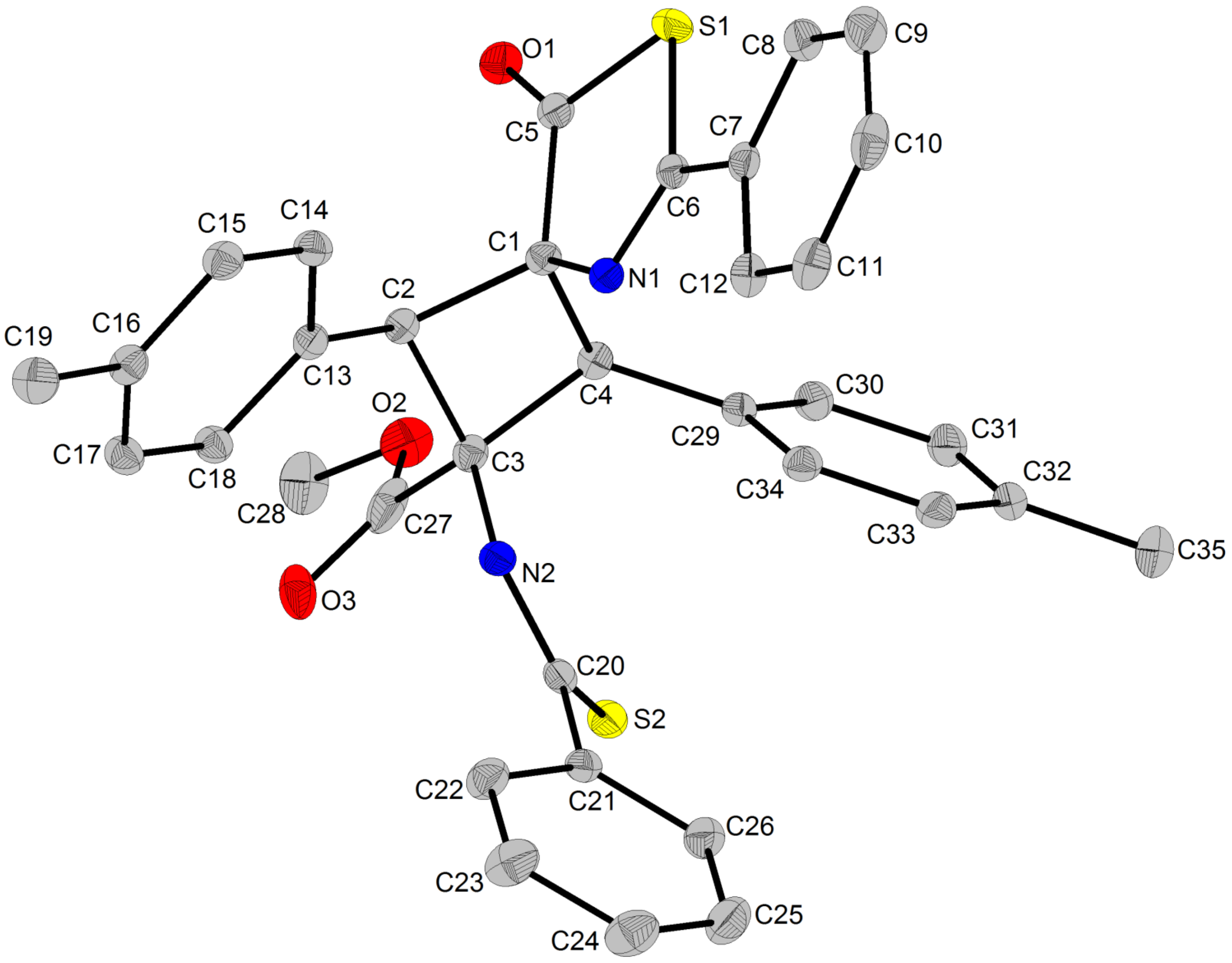

Molecular structure of **4b**·CH_2Cl_2. Thermal ellipsoids are drawn at 30% probability. Hydrogen atoms are omitted for clarity and solvent molecule not shown

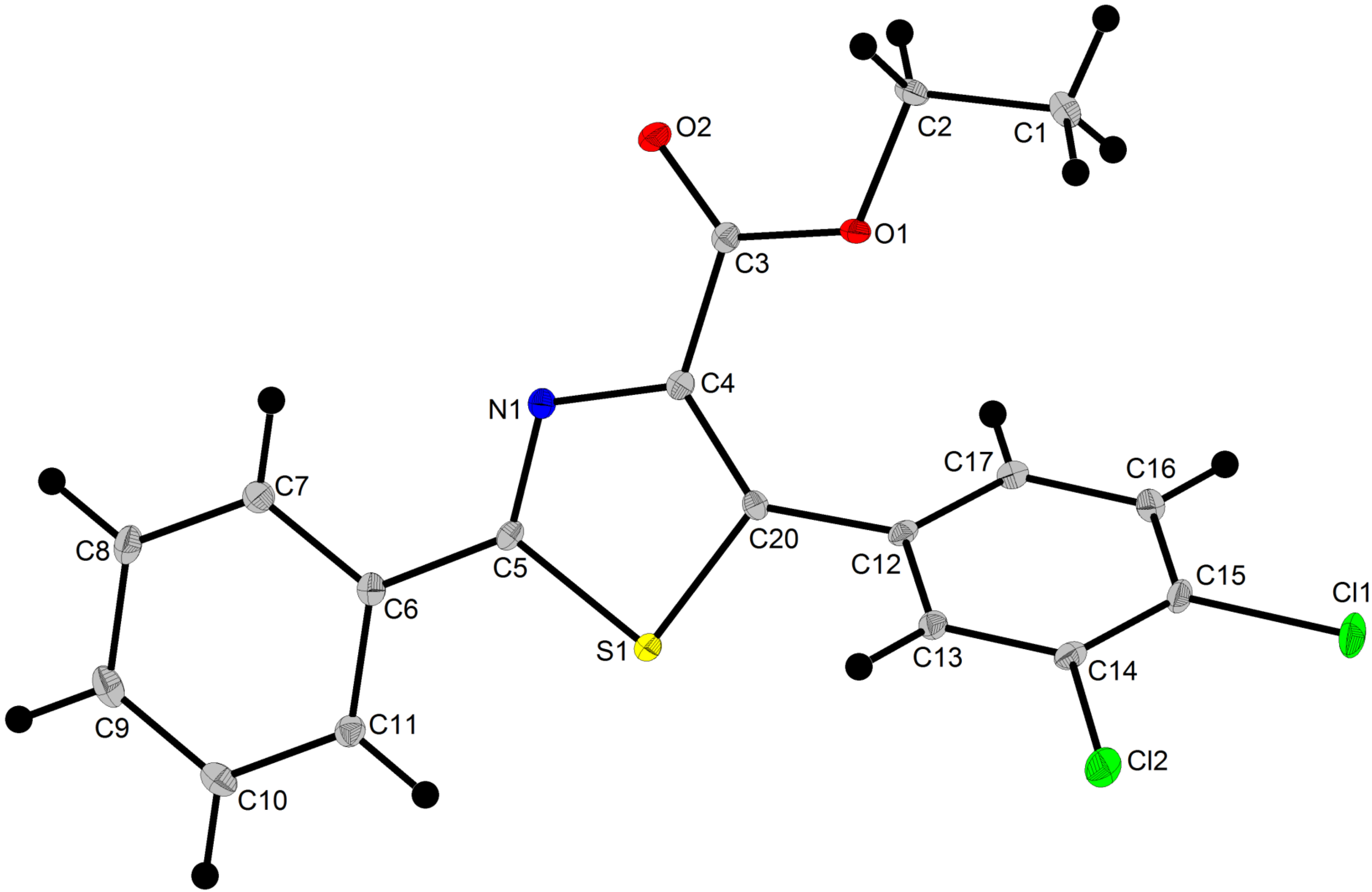

Molecular structure of **7n**. Thermal ellipsoids are drawn at 30% probability

Crystallographic Data

Table S1. Crystal data and details of data collection for **3g**, **3h**, **3m**, **4b**·CH_2Cl_2 and **7n**.

Compound	**3g**	**3h**	**3m**	**4b**·CH_2Cl_2	**7n**
Empirical formula	$C_{32}H_{20}N_4O_6S_2$	$C_{34}H_{20}F_6N_2O_2S_2$	$C_{36}H_{30}N_2O_2S_2$	$C_{36}H_{32}Cl_2N_2O_3S_2$	$C_{18}H_{13}Cl_2NO_2S$
Formula weight	620.64	666.64	586.74	675.65	378.25
T [K]	293(2) K	100(2)	100(2)	100(2)	100(2)
Crystal system	Orthorhombic	Monoclinic	Tetragonal	Monoclinic	Triclinic
Space group	Pnma	$P2_1/c$	$P4_32_12$	$P2_1/c$	P-1
a [Å]	13.6825(5)	10.0174(4)	15.02(3)	9.4339(4)	7.0885(7)
b [Å]	16.3580(7)	11.2247(5)	15.02(3)	20.0162(9)	8.9789(9)
c [Å]	16.1301(7)	26.0949(12)	13.08(3)	17.5781(8)	14.1650(15)
α [°]	90.00	90.00	90.00	90.00	77.999(2)
β [°]	90.00	95.3550(10)	90.00	92.363(1)	76.623(2)
γ [°]	90.00	90.00	90.00	90.00	72.151(2)
V [$Å^3$]	3610.2(3)	2921.4(2)	2949(14)	3316.5(3)	825.69(15)
Z	4	4	4	4	2
ρ_{calcd} [g cm^{-3}]	1.142	1.516	1.322	1.353	1.521
Absorption coefficient [mm^{-1}]	0.190	0.258	0.217	0.361	0.530
Crystal size [mm]	0.11 x 0.07 x 0.06	0.117 x 0.111 x 0.080	0.320 x 0.103 x 0.094	0.420 x 0.370 x 0.270	0.200 x 0.090 x 0.060
Θ range for data collection	3.165 to 28.062	2.398 to 28.303	2.471 to 28.418	1.542 to 28.681	2.410 to 28.288
Reflections collected	53261	46941	43659	29004	11320
Independent reflections	4331 [R(int) = 0.0859]	7247 [R(int) = 0.0475]	3707 [R(int) = 0.0721]	7954 [R(int) = 0.0276]	9328 [R(int) = 0.0356]
Data/restraints/parameters	4331/0/229	7247/18/415	3707/0/192	7954/0/416	9328 / 0 / 219
Final R indices [I>2sigma(I)]	R1 = 0.0625 wR2 = 0.2074	R1 = 0.0605 wR2 = 0.1483	R1 = 0.0384 wR2 = 0.0804	R1 = 0.0523 wR2 = 0.1304	R1 = 0.0405 wR2 = 0.0817
R indices (all data)	R1 = 0.1313 wR2 = 0.2703	R1 = 0.0886 wR2 = 0.1687	R1 = 0.0508 wR2 = 0.0868	R1 = 0.0674 wR2 = 0.1411	R1 = 0.0524 wR2 = 0.0882
Goodness-of-fit on F^2	0.853	1.032	1.059	1.096	1.049
Largest diff. peak/hole / e $Å^{-3}$	0.241 / -0.257	1.168 / -0.603	0.226 /-0.285	1.089 / -0.685	0.323 / -0.340
